# Reactivity of Nitroarenes toward Alkyl Radicals Enables Direct C–H Alkylation

**DOI:** 10.1021/jacs.6c10599

**Published:** 2026-07-30

**Authors:** Kousik Das, Sayonika Chakraborty, Eno Paenurk, Burkhard König

**Affiliations:** † Fakultät für Chemie und Pharmazie, 9147 Universität Regensburg, 93053 Regensburg, Germany

## Abstract

Friedel–Crafts alkylation is a cornerstone transformation for arene functionalization; however, strongly electron-deficient nitroarenes fail to undergo alkylation under conventional conditions. This long-standing limitation is notable given the prevalence of nitroarenes in pharmaceuticals and agrochemicals and their central role as synthetic building blocks. Here we report a photocatalytic strategy that overcomes this challenge, enabling direct C–H alkylation of nitroarenes using readily available carboxylic acids as alkyl radical precursors. Under mild conditions, alkyl radicals generated through a decarboxylative process add preferentially to the aromatic π-system while preserving the nitro functionality, suppressing competing oxygen-atom transfer and reduction pathways. This transformation is compatible with a wide range of nitroarenes and carboxylic acids and tolerates diverse functional groups. Mechanistic studies support a pathway involving decarboxylative radical addition, followed by oxidation and rearomatization mediated by an organophotocatalyst. The photocatalytic strategy is also applicable to alkyltrifluoroborates as alternative alkyl radical precursors. This method enables late-stage functionalization of complex nitroarenes and bioactive molecules, providing a modular platform for sequential C–H diversification and expanding the synthetic utility of nitroarenes.

## Graphical abstract

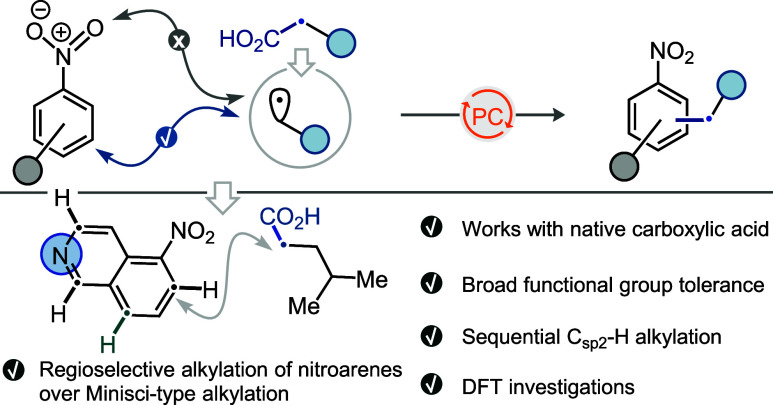

## Introduction

The Friedel–Crafts reaction^1^ remains one of the most straightforward and widely taught methods for introducing alkyl groups onto aromatic rings. However, its effectiveness is fundamentally limited by the electronic nature of the substrate. Electron-deficient arenes, in particular, exhibit poor reactivity under classical electrophilic alkylation conditions. Even in cases where electrophilic activation can be forced, the harsh Lewis or Brønsted acidic conditions required for Friedel–Crafts chemistry promote numerous competitive pathways, including overalkylation and carbocation rearrangements.^1,2^ The strongly electron-withdrawing nitro group represents one of the most challenging cases. A nitro substituent profoundly deactivates the aromatic ring toward electrophilic substitution, often eliminating reactivity altogether (Figure 1A). These intrinsic limitations have largely precluded Friedel–Crafts alkylation as a general or practical approach for modifying native nitroarenes. Alternative strategies such as vicarious nucleophilic substitution^3,4^ offer only limited opportunities for alkyl incorporation. These methods are largely confined to α-electron-withdrawing substituents, rely on prefunctionalized alkylating reagents, and often exhibit poor functional-group tolerance.^3,4^ Consequently, a streamlined, direct, and broadly applicable alkylation of unmodified nitroarenes remains an unsolved problem (Figure 1A).

**Figure 1:**
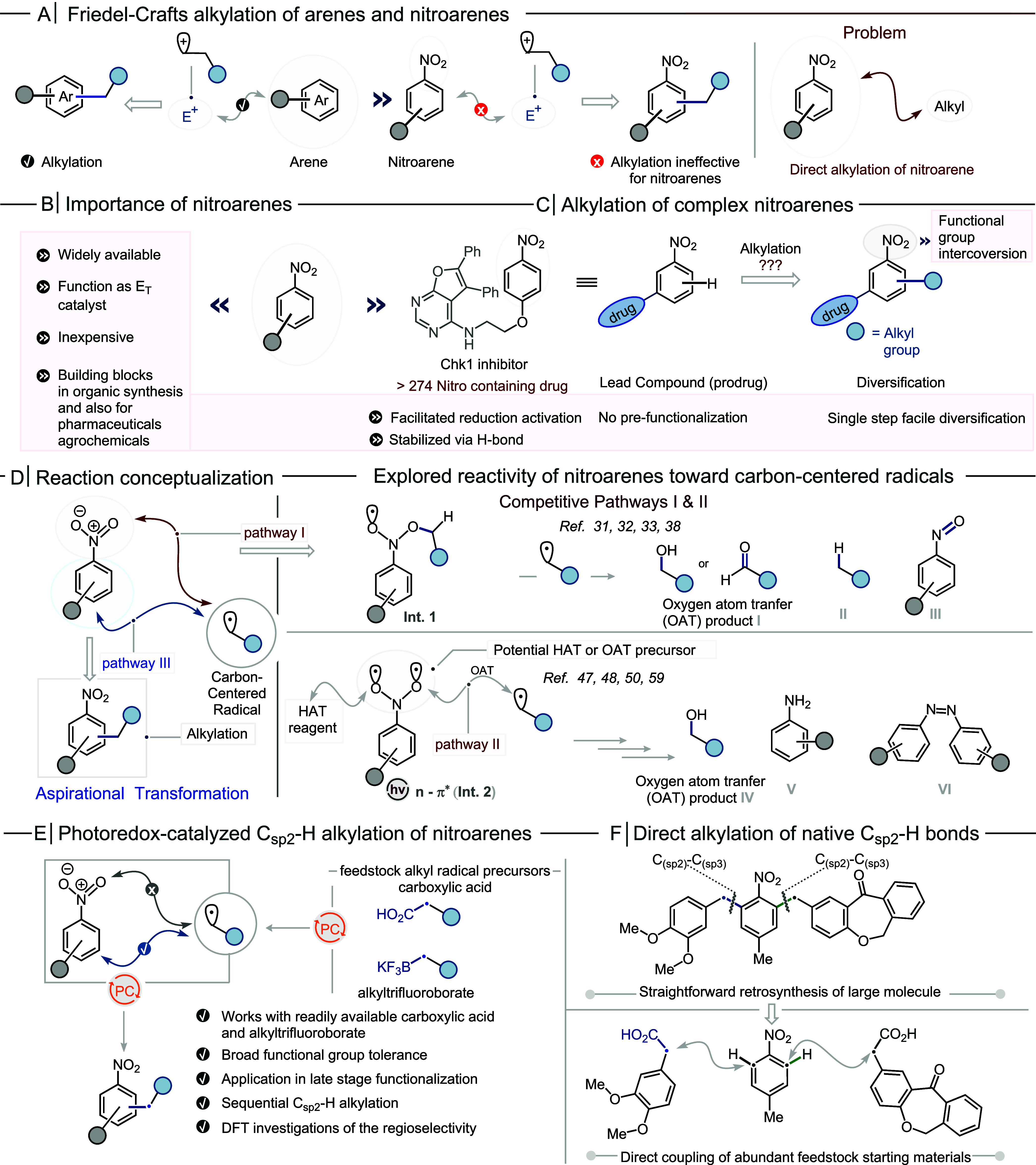
Friedel–Crafts alkylation of arenes and nitroarenes and bioactive molecules containing nitroarenes. A photoredox strategy for C–H alkylation of nitroarenes with readily available carboxylic acids and alkyltrifluoroborates.

This challenge is particularly significant given the central role of nitroarenes across multiple areas of chemistry (Figure 1B).^5–14^ Nitroarenes are inexpensive, widely available, and possess distinctive electronic properties that render them indispensable building blocks in pharmaceuticals^15–17^ and agrochemicals.^16,18^ Moreover, they can function as energy-transfer catalysts under photochemical conditions.^19^ In medicinal chemistry, nitroarenes can serve as versatile synthetic intermediates, enabling hydrogen-bonding interactions, imparting conformational effects, and acting as bioreductive triggers under specific conditions.^14,15,20^ However, nitro groups are often considered structural alerts due to associated toxicity concerns and are therefore typically transformed into other functional groups (e.g., anilines) in advanced compounds. Consequently, their value is primarily realized as synthetic intermediates rather than as functionalities in final drug candidates. Accordingly, methods that enable their efficient diversification are valuable for accelerating structure–activity relationship (SAR) studies and downstream functionalization (Figure 1C).^21,22^

Recent advances in visible-light photoredox catalysis and electro-organic synthesis have enabled the mild formation of C­(sp^2^)–C­(sp^3^) bonds from abundant and readily available precursors via radical pathways.^2,21,23^ Carboxylic acids are especially appealing, as they are inexpensive, widely available, and readily converted into alkyl radicals via visible-light-mediated decarboxylation.^24–26^ This reactivity underlies hallmark transformations such as the Minisci reaction,^27–30^ which effects direct decarboxylative alkylation of electron-deficient heteroarenes-most notably protonated pyridines and quinolines. By contrast, nitroarenes exhibit fundamentally distinct behavior in radical-mediated transformations, both under classical conditions^31–35^ and within photoredox manifolds, owing to the unique redox and photochemical properties of the nitro group.^9,10,36–51^ Upon photoexcitation, nitro groups can function as hydrogen-atom transfer (HAT) reagents,^47,48^ participate in oxygen-atom transfer processes,^47,48,50,52–54^ or serve as precursors to nitrene-type intermediates.^9,10,46,55,56^ In addition, nitroarene-derived radical anions function as alkyl radical traps^34,35^ and inhibitors in free-radical polymerization.^57,58^ Consistent with these features, carbon-centered radicals generated in situ—whether photochemically or by other means—often interact with the nitro moiety in either its ground state (pathway I, Figure 1D)^31–33,38^ or excited state (pathway II, Figure 1D).^47,48,50,59^ These processes commonly afford aromatic amines or oxygen-transfer products. This property underpins diverse photochemical and radical-based transformations,^31,34,35,39,40,42,43,45,50^ including Mukaiyama-type alkene hydration mediated by nitroarenes^33^ and access to 2-aminophenols via triplet-state interaction with carbon-centered radicals.^59^ Collectively, these studies establish significant advances in the engagement of nitro groups at the *O*-atom with alkyl radicals through oxygen-atom transfer and related processes, thereby broadening their synthetic utility. To the best of our knowledge, photochemical alkylation of native nitro­(hetero)­arenes via carbon-centered radical coupling to the arene ring remains unexplored.

We questioned whether appropriately designed photoredox conditions could bias alkyl radicals toward engagement with the aromatic π-system (Figure 1D, pathway III), thereby reducing participation of the canonical reactivity pathways of the nitro group. Achieving such selectivity would provide a powerful, robust strategy for nitroarene alkylation that lies beyond the scope of classical Friedel–Crafts alkylation chemistry. However, realization of this goal is impeded by several competing intrinsic pathways, including radical addition to the nitro group (pathway I, Figure 1D),^31–33,38^ oxygen-atom transfer from excited-state nitroarenes to alkyl radicals (pathway II)^47,48,50,59^ and photoreduction of the nitro moiety.^36,37,60,61^ Collectively, these factors present a major barrier to selective and efficient nitroarene alkylation.

A catalytic strategy capable of overcoming these challenges and enabling direct C­(sp^2^)–H alkylation of nitroarenes using widely available and inexpensive alkylating agents would therefore be highly desirable. This advance would markedly expand the chemical space of alkylated nitroarene derivatives, both in terms of arene substitution and alkyl group diversity. Such a transformation could streamline retrosynthetic strategies for complex nitroarene-containing drug targets. In addition, it would furnish a versatile handle for both early stage lead discovery and late-stage functionalization. Despite the vast potential and utility of such a method, progress in this area has remained limited – examples include our preliminary version of this work disclosed on *ChemRxiv* ^62^ and, in parallel with our work, a more recent report by Wang and co-workers describing iron-catalyzed electrochemical C­(sp^2^)–C­(sp^3^) coupling of nitro­(hetero)­arenes with alkyl bromides.^63^ However, the scope of that method is largely restricted to secondary benzylic and tertiary radicals bearing electron-withdrawing groups.

We herein report an efficient and practical method for the alkylation of nitroarenes using readily available, inexpensive, and commercially accessible alkylating agents such as carboxylic acids. The protocol accommodates a wide range of nitroarenes and carboxylic acids. The photocatalytic strategy can also employ alkyltrifluoroborates as alternative alkyl radical precursors. The transformation exhibits broad functional-group tolerance and is applicable to the late-stage functionalization of structurally complex molecules, including pharmaceutical derivatives. Furthermore, it enables sequential alkylation of C­(sp^2^)–H bonds in nitroarenes, providing a versatile platform for molecular diversification (Figure 1F). Importantly, this method enables incorporation of primary and secondary radicals (both benzylic and aliphatic) as well as tertiary aliphatic radicals from unactivated substrates, under operationally simple, metal-free conditions using native carboxylic acids as alkyl radical precursors.

## Results and Discussion

We initiated our investigation using 1-methyl-4-nitrobenzene (**1a**, 1.0 equiv) and 2-phenylacetic acid (**2a**, 1.5 equiv) as the alkyl radical precursor in DMA, employing 4CzIPN as the photocatalyst and TMG (1.5 equiv) as the base under purple light irradiation (390 nm). Under these conditions, the desired alkylated product (**3**) was detected by GC-MS in only trace amounts (2%). Instead, several byproducts predominated, including the reduction product *p*-toluidine (**3a**, 49%), the oxygen-transfer product benzaldehyde (**3b**), a condensation product (**3c**) arising from *p*-toluidine and benzaldehyde (**3b** + **3c**, 26%), and 1,2-di-*p*-tolyldiazene 1-oxide (**3d**, 6%) (Figure 2, column 1). The formation of benzaldehyde is consistent with the interception of the benzyl radical by the nitro group of the nitroarene, either in its ground state or via interaction with the excited nitroarene, as outlined in pathways I and II (Figure 1D).^31,32,38,47,48,59^ We therefore reasoned that excitation of the nitroarene may play a key role in promoting oxygen-atom transfer pathways and reduction of the nitro group to the corresponding aniline.^36,37,60^ On this basis, we hypothesized that employing lower-energy (longer-wavelength) light could suppress direct nitroarene excitation, thereby disfavoring oxygen-transfer processes and photoreduction of the nitro group. Under these conditions, alkyl radicals could instead be directed toward productive π-addition, enabling nitroarene C­(sp^2^)–H alkylation. Consistent with this hypothesis, switching to blue light irradiation (450 nm) increased the yield of the desired product (**3**) to 15%, accompanied by a substantial decrease in the formation of *p*-toluidine (**3a**, 10%) and the combined oxygen-transfer and condensation byproducts (**3b** + **3c**, 19%) (Figure 2, column 3). These observations further suggested that, beyond excited-state control, the stability of the alkyl radical may play a decisive role in determining reaction outcome.^32,57^ We therefore considered whether modulation of the solvent environment could exert additional control over radical reactivity. Accordingly, replacing DMA with DMSO led to a further enhancement in selective nitroarene C­(sp^2^)–H alkylation (column 4, 38% yield). This effect may reflect the high polarity and coordinating nature of DMSO, which can provide a stabilizing solvation environment for radical intermediates through interactions with the lone pairs of the sulfoxide oxygen,^64,65^ thereby favoring productive C­(sp^2^)–H functionalization. Replacing TMG with the stronger, non-nucleophilic base BTMG further improved the yield to 62% (Figure 2, column 8). Screening of photocatalysts identified 4CzIPN as the most efficient catalyst for this transformation (Figure 2, columns 9–10). Finally, employing a slight excess of 2-phenylacetic acid (1.3 equiv) increased the yield to 76% (column 11). Control experiments confirmed that visible light, the photocatalyst 4CzIPN and base are essential for this transformation (columns 12–13). Although competing nitro-group-centered pathways cannot be completely eliminated, the optimized photocatalytic conditions consistently favor arene π-addition across the examined substrates.

**Figure 2:**
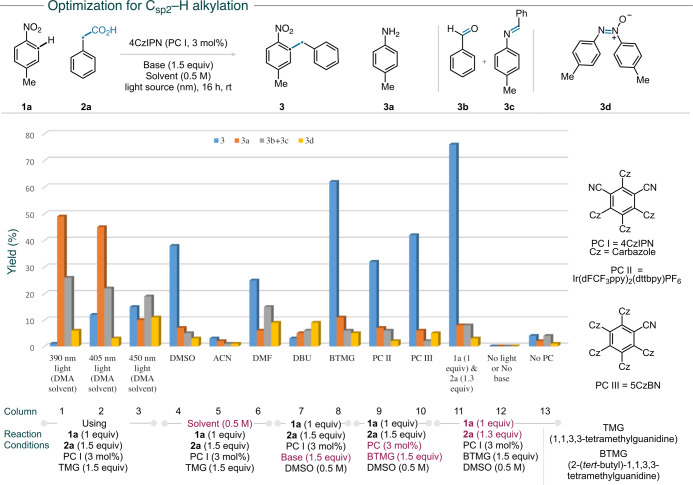
Optimization of reaction conditions for C–H alkylation reactions (Optimized Conditions: nitroarene (**1a**, 1 equiv), carboxylic acid (**2a**, 1.3 equiv), PC I (3 mol %), BTMG (1.5 equiv), DMSO (0.5 M), 450 nm, 16 h, see section 3 in the Supporting Information for experimental details). The yields of the desired products were monitored by GC and GC-MS analysis using 1,3-dimethoxybenzene as an internal standard.

### Synthetic Examples

With the optimized conditions established, we next evaluated the generality of the transformation by varying the carboxylic acid-derived radical precursor. A wide array of substituted phenylacetic acids proved compatible, furnishing the corresponding products (**3**–**18**, Figure 3) in moderate to good yields. Electron-donating substituents on the aromatic ring were particularly effective (products **5**–**17**), whereas strongly electron-withdrawing groups, exemplified by 2-(4-(trifluoromethyl)­phenyl)­acetic acid, resulted in diminished reactivity (product **19**). Acids bearing extended π-systems, such as 2-(naphthalen-1-yl)­acetic acid, also performed well, affording product **20** in good yield.

**Figure 3:**
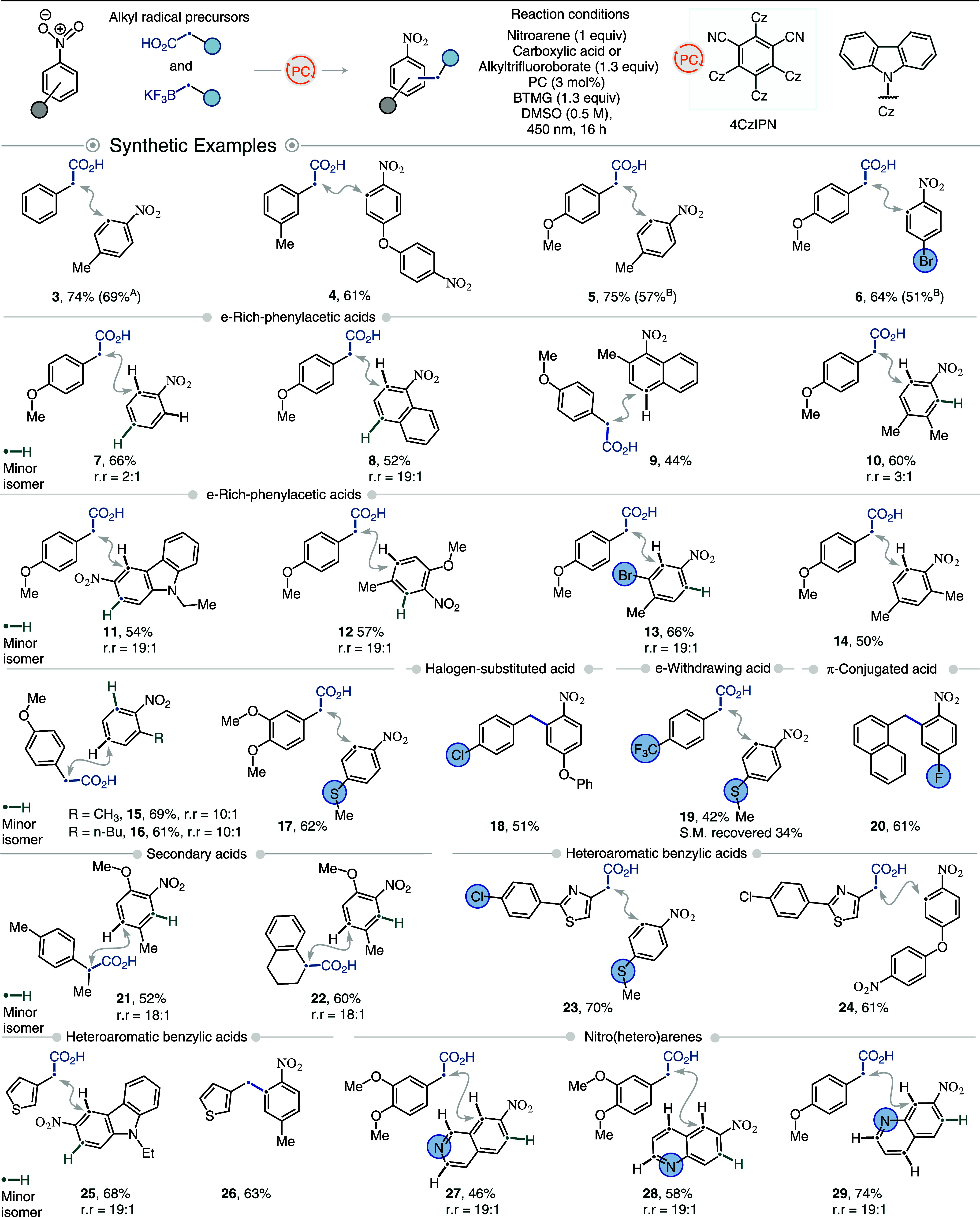
Synthetic examples of C­(sp^2^)–H alkylation reactions of nitroarenes under photoredox-catalyzed reaction conditions on a 0.2 mmol scale (see section 3B in the Supporting Information for experimental details). Isolated yields are reported unless noted otherwise. Regioisomeric ratios (r.r) are calculated by GC and GC-MS analysis. ^A^Using potassium benzyltrifluoroborate as alkyl radical precursor; ^B^using potassium trifluoro-(4-methoxybenzyl)­borate. S.M. = Starting Material (Nitroarene).

We then explored the scope of the nitroarene partner. A variety of nitroarenes, including 1-methyl-4-nitrobenzene, 4,4′-oxybis­(nitrobenzene), 1-bromo-4-nitrobenzene, methyl­(4-nitrophenyl)­sulfane, 1-nitro-4-phenoxybenzene, and 1-fluoro-4-nitrobenzene, underwent smooth ortho-alkylation (products **3**, **4**, **6**, **17**, **18**, and **20**, Figure 3). Nitrobenzene afforded a mixture of regioisomers (**7**, ortho:para = 2:1; see Figure 7 and section 4 in the Supporting Information for theoretical analysis of regioselectivity). In contrast, 1-nitronaphthalene displayed exclusive *ortho*-selectivity (product **8**). Notably, substrates bearing two distinct ortho sites, such as 9-ethyl-3-nitro-9*H*-carbazole and 2-bromo-1-methyl-4-nitrobenzene, exhibited high regioselectivity, with alkylation occurring preferentially at the more sterically hindered position (products **11** and **13**), likely governed by electronic effects (see Figure 7 and section 4 in the Supporting Information for theoretical analysis of regioselectivity). In contrast, 1-methoxy-4-methyl-2-nitrobenzene, which contains both ortho and para sites, afforded the para-alkylated product **12** exclusively. Various ortho-substituted nitroarenes, such as 1-methyl-2-nitrobenzene and 1-butyl-2-nitrobenzene, underwent efficient alkylation to afford the corresponding products (**15** and **16**) with moderate regioselectivity. The method is not limited to primary benzylic acids. Secondary benzylic acids-including 2-(*p*-tolyl)­propanoic acid and 1,2,3,4-tetrahydronaphthalene-1-carboxylic acid-provided the corresponding products (**21** and **22**) in good yields and regioselectivities. In contrast, tertiary benzylic acids did not participate effectively in the C–H alkylation reaction (see section 3B in the Supporting Information for unsuccessful substrates). Heteroaromatic benzylic acids such as 2-(2-(4-chlorophenyl)­thiazol-4-yl)­acetic acid and 2-(thiophen-3-yl)­acetic acid also coupled efficiently, affording products **23**–**26** in good yields. A broader set of nitroarenes, including methyl­(4-nitrophenyl)­sulfane, 4,4′-oxybis­(nitrobenzene), 9-ethyl-3-nitro-9*H*-carbazole, and 1-methyl-4-nitrobenzene, likewise underwent efficient functionalization (**23**–**26**). Alkyltrifluoroborates were also found to be effective alkylating agents under the same reaction conditions, affording the corresponding C–H alkylation products in good yields (**3**, **5**, and **6**). A range of nitro­(hetero)­arenes, in combination with aromatic benzylic carboxylic acids, underwent the desired alkylation in good yields with high regioselectivity (products **27**–**29**). Substrates bearing both nitro and heteroaromatic functionalities, such as 7-nitroquinoline, 7-nitroisoquinoline, and 6-nitroquinoline, exhibited selective alkylation at the nitro-substituted arene, with no reaction observed on the quinoline or isoquinoline ring (*i*.*e.*, no Minisci-type products **27**–**29**, Figure 3 and see section 4 in the Supporting Information for theoretical analysis of regioselectivity).

### Scope of Aliphatic Carboxylic Acid

Beyond benzylic substrates, the reactivity extends to aliphatic carboxylic acids under slightly modified conditions (see section 3B in the Supporting Information for experimental details). A variety of aliphatic carboxylic acids, including small-chain substrates, were compatible with the reaction, affording the desired products in moderate to good yields (**30**–**38**, Figure 4). For example, propionic acid enables the incorporation of an ethyl fragment into nitroarenes (product **30**), whereas but-3-enoic acid affords the vinylated product **32**, likely via isomerization of the initially formed alkylated intermediate to the thermodynamically more stable trans-conjugated alkene under the reaction conditions. Long-chain aliphatic acids, such as palmitic acid, stearic acid and oleic acid, are also well tolerated and serve as effective alkylating agents (product **39** to **41**). However, certain substrate combinations exhibited diminished reactivity; for example, the reaction of acetic acid with methyl (4-nitrophenyl)­sulfane to incorporate a methyl fragment afforded only trace amounts of the desired product (see section 3B in the Supporting Information). Different nitroarenes methyl (4-nitrophenyl)­sulfane, 1-methyl-4-nitrobenzene, 1-methoxy-4-methyl-2-nitrobenzene, 1-nitro-4-phenoxybenzene, 1-bromo-4-nitrobenzene and 2-bromo-1-methyl-4-nitrobenzene, in combination with various primary aliphatic carboxylic acids, undergo effective alkylation in good yields (product **30**, **42**, **43**, **50**, **51** and **52**, Figure 4). The reaction is not limited to primary carboxylic acids, as secondary and tertiary aliphatic acids also serve as effective alkylating agents, affording the desired products in moderate to good yields. A variety of secondary aliphatic carboxylic acids, including 2-methylpentanoic acid, 2-methylheptanoic acid, cyclopent-3-ene-1-carboxylic acid, and cyclohexanecarboxylic acid, act as efficient alkylating partners (products **44**–**47**). Importantly, cyclohexanecarboxylic acid with 4-nitrobenzonitrile affords the desired product in 41% yield (cyano-substituted product **47**; see Supporting Information, section 5.5.6). Furthermore, the use of 4-pentylbicyclo[2.2.2]­octane-1-carboxylic acid and adamantane-1-carboxylic acid as tertiary alkyl radical precursors with 1-methoxy-4-methyl-2-nitrobenzene and 4-nitrobenzonitrile, respectively, enables formation of quaternary carbon centers in moderate yields (**48** and **49**). Significantly, a range of nitro­(hetero)­arenes underwent selective ortho-alkylation with various aliphatic carboxylic acids at the nitro-substituted arene (product **53**–**56**), while no reaction was observed on the quinoline or isoquinoline ring, consistent with previous observations (i.e., no Minisci-type products, see Figure 7 and section 4 in the Supporting Information for theoretical analysis of regioselectivity).

**Figure 4:**
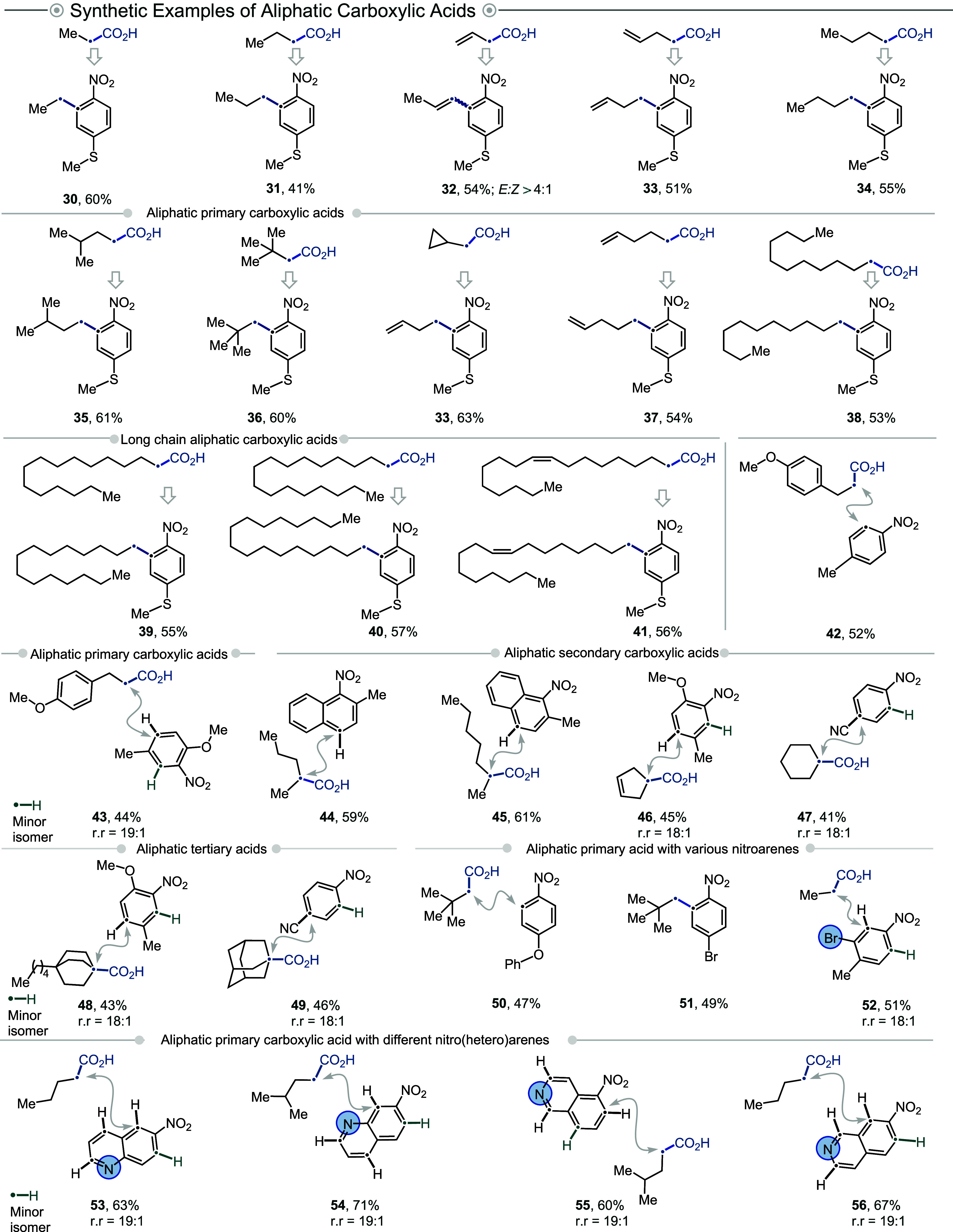
Synthetic examples of C­(sp^2^)–H alkylation reactions of nitroarenes with aliphatic carboxylic acid on a 0.2 mmol scale (Reaction Conditions: nitroarene (1 equiv), carboxylic acid (1.3 equiv), PC (6 mol %), BTMG (1.3 equiv), DMSO:ACN (3:2 ratio), 450 nm, 16 h, see section 3B in the Supporting Information for experimental details). Isolated yields are reported unless noted otherwise. Regioisomeric ratios (r.r) and diastereomeric ratio (*dr*) are calculated by GC and GC-MS analysis.

### Applications

The operational simplicity and robustness of the protocol render it particularly suitable for late-stage modification of pharmaceuticals and biomolecule derivatives. Several medicinally relevant carboxylic acids—including isoxepac, flurbiprofen, ibuprofen, zaltoprofen, ketoprofen, loxoprofen and naproxen (products **57**–**65**, Figure 5)—served as efficient alkylating agents. Nitroarenes such as 1-methoxy-4-methyl-2-nitrobenzene, 4,4′-oxybis­(nitrobenzene) and 7-nitroquinoline coupled effectively under the same conditions, with high regioselectivity governed by the substitution pattern of the aromatic core (products **57**, **58** and **59**). Notably, reactions of flurbiprofen with 4-nitrobenzonitrile furnish the cyano-substituted product **61** (see section 5.5.6 of the Supporting Information for mechanistic details). Certain substrate combinations, however, exhibited attenuated reactivity. For example, coupling of loxoprofen with 4-nitrobenzonitrile afforded the desired alkylation product in poor yield (cyano-substituted product **65**; see section 5.5.6 of the Supporting Information for mechanistic details), while a ketone byproduct (**66**) was formed in moderate yield. This divergence may arise from oxygen-atom transfer to the benzylic radical, generating an alcohol intermediate that subsequently undergoes photochemical oxidation to the corresponding ketone. The strategy is similarly effective for structurally intricate nitroarenes derived from complex scaffolds, including diacetone-*D*-glucose, gemfibrozil, oxaprozin, farnesol and diacetone-*D*-galactose, all of which furnished C­(sp^2^)–H alkylated products in moderate to good yields (**67**–**73**, Figure 5). A wide assortment of carboxylic acids—including aliphatic acids such as pentanoic acid and pent-4-enoic acid (**71** and **72**), aryl-substituted acids such as 2-(naphthalen-1-yl)­acetic acid (**73**), heteroaromatic acids such as 2-(2-(4-chlorophenyl)­thiazol-4-yl)­acetic acid (**68**), and pharmaceutically relevant acids such as isoxepac (**67**)—proved to be efficient alkylating partners. This approach is also effective for large-scale synthesis. For example, the coupling of isoxepac with 1-bromo-4-nitrobenzene provides the desired alkylated product **74** (Figure 5) in 57% isolated yield on a 2 mmol scale using a low photocatalyst loading.

**Figure 5:**
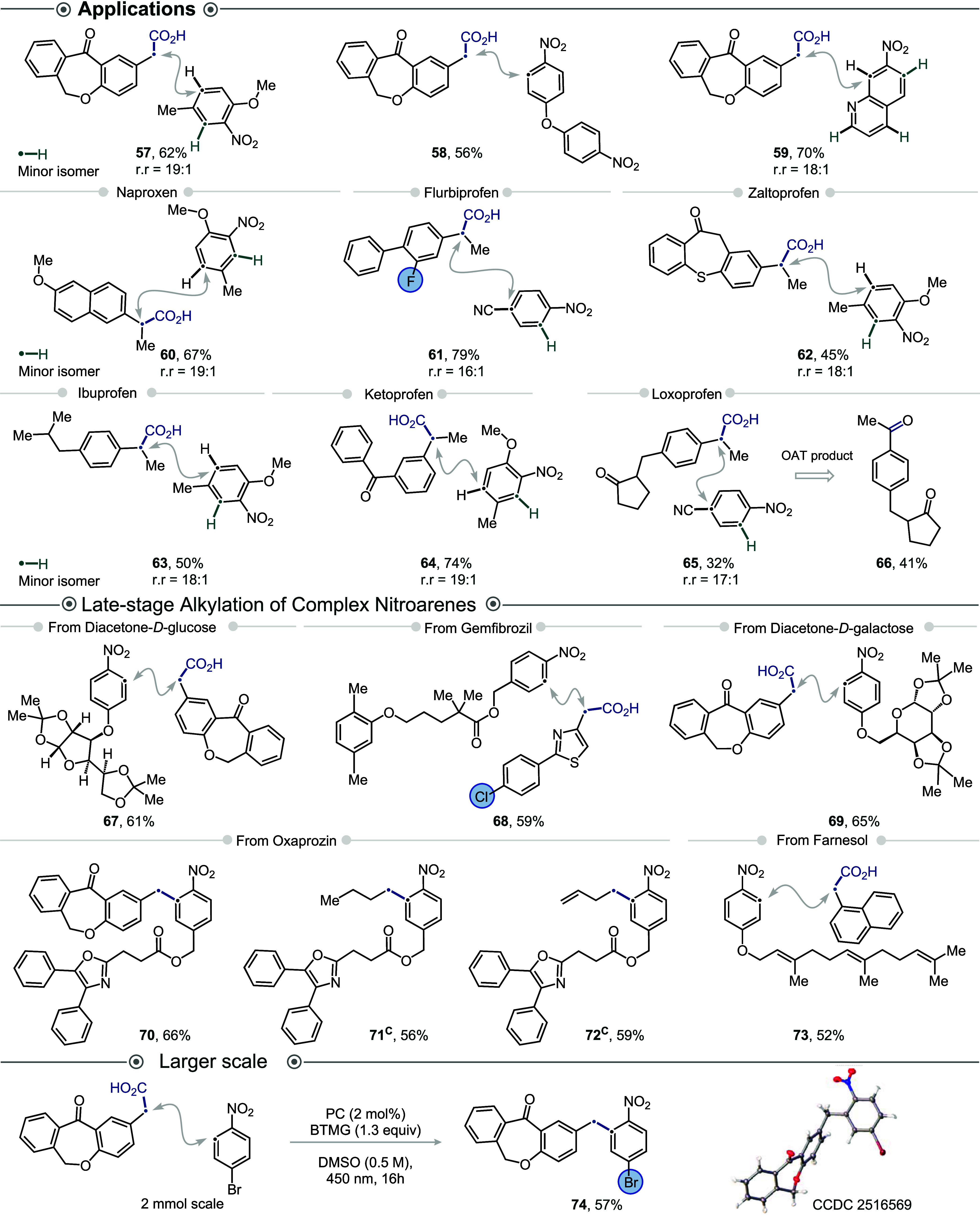
Synthetic examples of the functionalization of biomolecules as well as drug molecules on a 0.2 mmol scale (Reaction Conditions: nitroarene (1 equiv), carboxylic acid (1.3 equiv), PC (3 mol %), BTMG (1.3 equiv), DMSO (0.5 M), 450 nm, 16 h). ^C^Using modified reaction condition (nitroarene (1 equiv), carboxylic acid (1.3 equiv), PC (6 mol %), BTMG (1.3 equiv), DMSO:ACN (3:2), 450 nm, 16 h, see section 3B in the SI for experimental details). Larger scale synthesis (2 mmol scale). Isolated yields are reported unless noted otherwise. Regioisomeric ratios (r.r) are calculated by GC and GC-MS analysis.

To further showcase the synthetic versatility of this methodology, we carried out a series of representative downstream transformations. The coupling of 2-(naphthalen-1-yl)­acetic acid with 1-bromo-4-nitrobenzene furnished the corresponding ortho-alkylated product in good yield, even on a larger scale (product **75**, Figure 6, 2 mmol scale). This product was readily reduced under acidic iron conditions to give the corresponding aniline derivative (**76**). The resulting aniline intermediate offers two orthogonal synthetic handles: the aryl amine, which can participate as a nucleophile in photochemical nickel-catalyzed cross-coupling with aryl halides to furnish secondary arylamine (product **77**), and the C­(sp^2^)–Br bond, which undergoes efficient C­(sp^2^)–S bond formation with *N*-Boc-*L*-cysteine methyl ester under nickel catalysis to provide thioether product **78** in 74% isolated yield. We next explored whether this methodology could be extended to the sequential functionalization of nitroarenes using two distinct carboxylic acid-derived alkyl radicals. To our delight, the reaction of 4-nitrobenzonitrile with isoxepac as the alkylating agent successfully replaced the cyano group, affording the corresponding alkylated product **79** in 64% isolated yield. Subsequent addition of 2-(2-(4-chlorophenyl)­thiazol-4-yl)­acetic acid enabled C­(sp^2^)–H alkylation at the ortho position, furnishing the dialkylated product **80**. Encouraged by this result, we questioned whether sequential C­(sp^2^)–H alkylation could also be achieved. Gratifyingly, treatment of 1-methyl-4-nitrobenzene with 2-(3,4-dimethoxyphenyl)­acetic acid delivered the monoalkylated product **81** in good yield. A second alkylation at the remaining ortho position using isoxepac as the alkyl source provided the dialkylated product **82** in 40% isolated yield.

**Figure 6:**
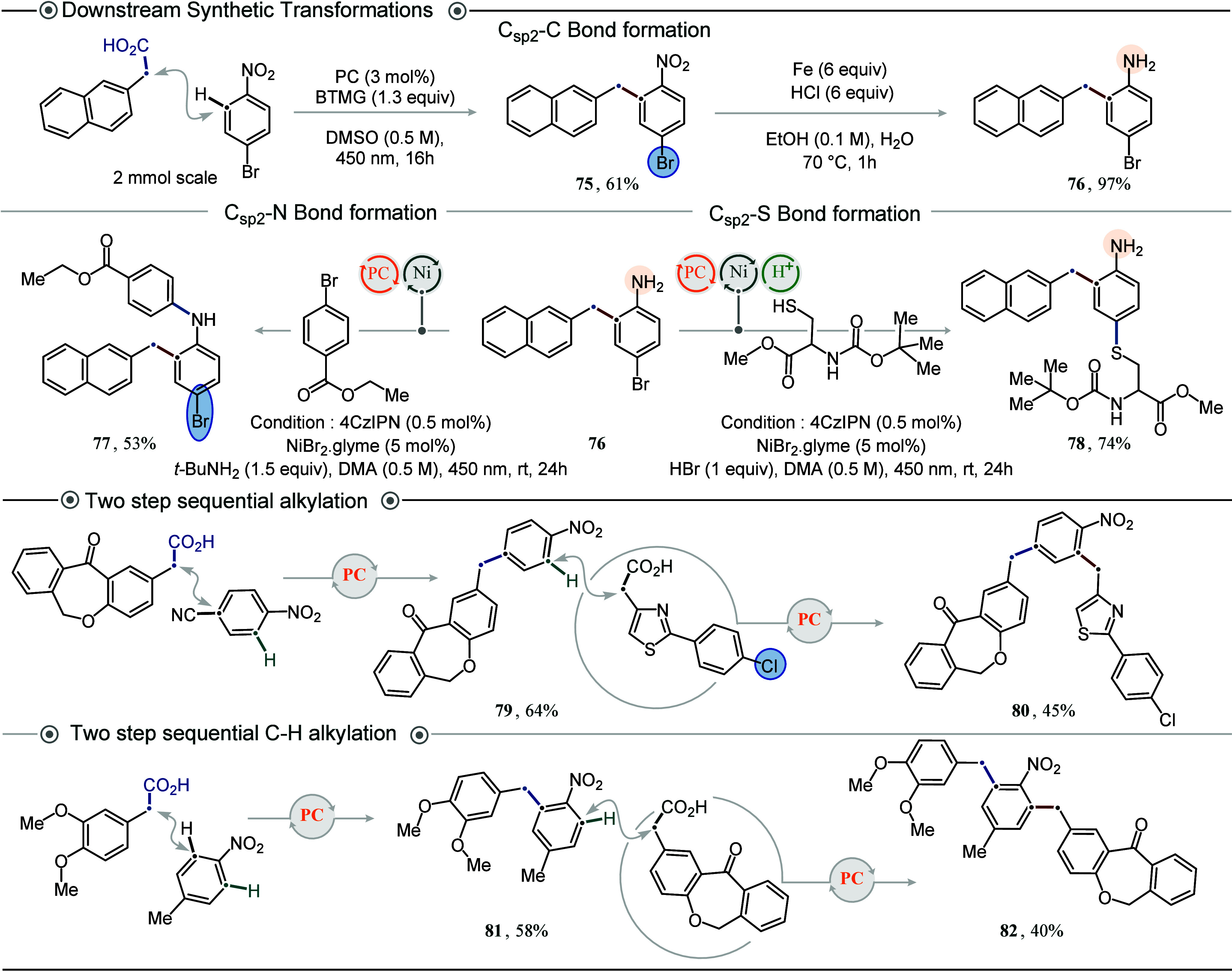
Representative downstream transformations and two-step synthetic transformations on a 0.2 mmol scale (see section 6 in the Supporting Information for experimental details). Isolated yields are reported unless noted otherwise.

### Regioselectivity

The regioselectivity of radical substitutions in nitroarenes has traditionally been explained by analyzing the stability of the radical intermediate.^66^ Both Lewis structures or simple Hückel theory-based arguments can be invoked to rationalize the relative intermediate stabilities, focusing on the stabilizing effects on the unpaired electron (Figure S3 in the Supporting Information). To better understand the regioselectivity patterns in the various substrates studied in this work, we conducted density functional theory (DFT) calculations of the ethyl radical addition to various nitroarenes. The main trends in intermediate stability were consistent for both ethyl and benzyl radicals (see section 4 in the Supporting Information), suggesting that the ethyl radical results should be representative of the general reactivity trends.

The most stable intermediate corresponded to the observed regioselectivity for several molecules, but not in all cases ( in the Supporting Information). For example, the nitrobenzene (nb) intermediates from the addition to the *ortho* and *para* positions show spin delocalization onto the nitro group and are lower in energy than the corresponding *meta* intermediate (Figure 7A), in agreement with the experimentally observed regioselectivity (**7**). We note that the 1 kcal/mol difference between *ortho* and *para* intermediates is within the uncertainty of our DFT calculations, making these two sites essentially indistinguishable in our calculations. The almost 4 kcal/mol energy difference between the *ortho* and *para* intermediates of 1-nitronaphthalene (1-nn) would suggest a majority para product formation (Figure 7A) – however, experimentally, only the *ortho* product was observed (**8**). A similar disagreement was observed for the structurally related 5-nitroisoquinoline (**55**, see Figure S4, in the Supporting Information), implying that the radical intermediate stability does not solely determine the selectivity, at least in some cases.

**Figure 7:**
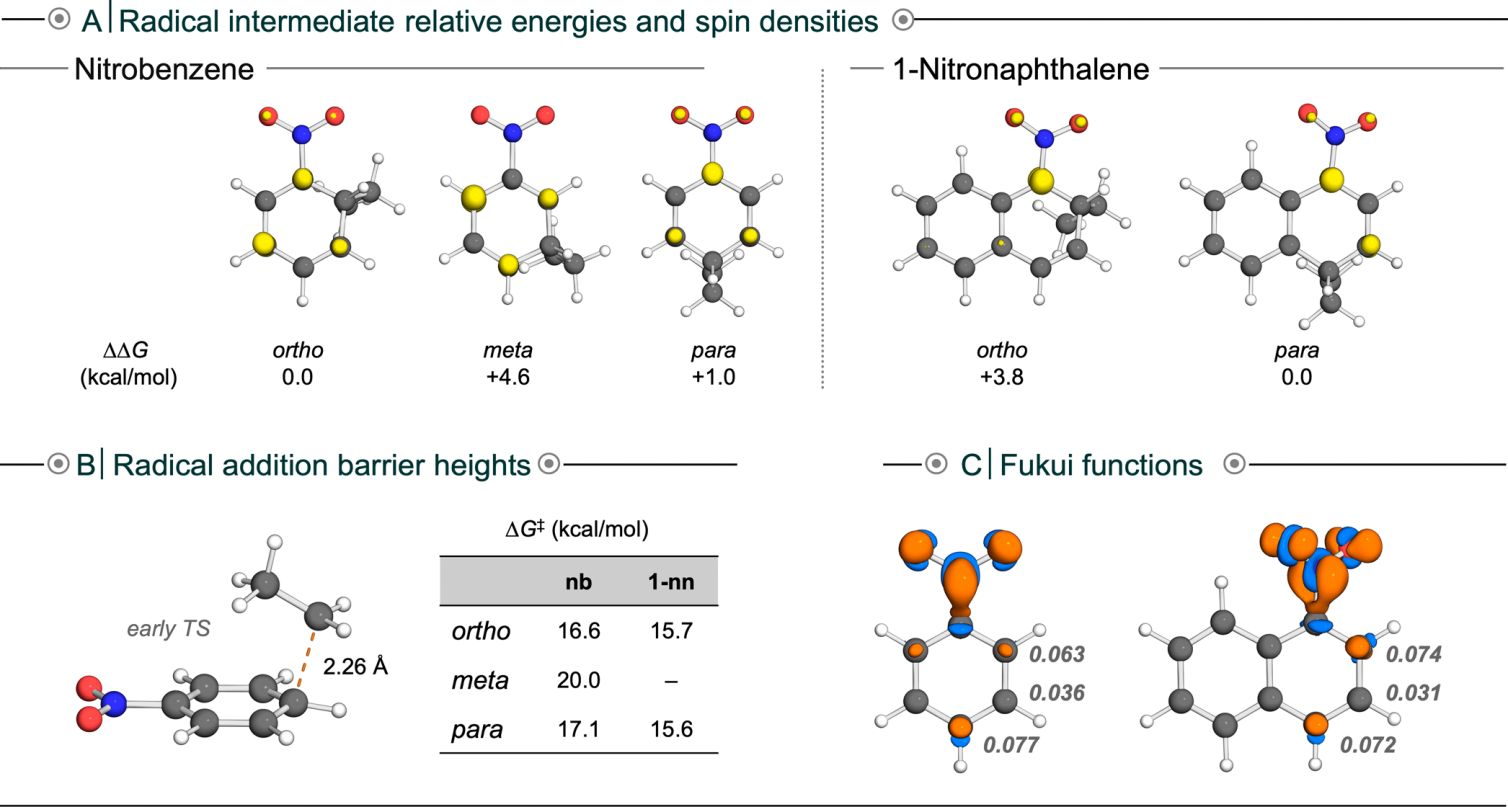
Calculated possible regioselectivity predictors. A) Radical intermediates for ethyl radical addition; relative free energies at 298.15 K with respect to the lowest energy intermediate and spin densities shown in yellow (surface isovalue 0.03 au); B) Ethyl radical addition transition state geometry for nitrobenzene and selected calculated free energy barriers at 298.15 K. C) Fukui function surfaces at isovalue 0.007 au, where orange shows positive and blue shows negative valued surface, together with condensed Fukui function values of select carbons in gray. Geometries were optimized and frequencies calculated with ORCA 6.1 ^70^ at the ωB97X-D3­(BJ)^71^/def2-TZVP^72^ level of theory, followed by single-point energy calculations at the ωB97X-D3­(BJ)/def2-QZVP level of theory, using the SMD solvent model for DMSO.^73^ Fukui functions were calculated with Multiwfn v2026.4.10.^74^ Visualizations were prepared with Open-Source PyMOL v3.^84^

We then studied the radical addition reaction barrier heights, assuming this step to be the selectivity-determining one. The barrier heights largely followed the same trends as the intermediate stabilities (see , in the Supporting Information). For nitrobenzene (nb), addition to the *ortho* and *para* positions is also kinetically favorable compared to the *meta* position (Figure 7B). However, for 1-nitronaphthalene (1-nn), the two positions that lead to thermodynamically inequivalent intermediates have almost equal barrier heights (Figure 7B). Although this shift in trend is notable, it still does not explain the experimentally observed selectivity.

As the explicit thermochemistry calculations did not fully agree with all the experimental results, we attempted to achieve similar semiquantitative regioselectivity predictions with more efficient approaches. We noted that the radical addition transition state geometries correspond to an early transition state, where the aromatic reactant is still mostly planar and the forming bond length is above 2 Å (Figure 7B). We therefore hypothesized that reactivity descriptors based on the reactant geometry could provide a practical predictor of regioselectivity. Considering that the radical in these reactions acts as a nucleophilic radical, we calculated the nucleophilic Fukui functions^67,68^ for the nitroarenes (see Figure S6, in the Supporting Information). As shown in Figure 7C, the Fukui function predicts the same regioselectivity trends that we arrived at with other, more costly calculations. Moreover, the atomic condensed Fukui function provides a rapid numerical descriptor for identifying the most likely sites for radical addition (Figure 7C).

We note that the regioselectivity of radical reactions can be influenced by multiple factors,^69^ which are not fully captured by our calculations (e.g., solvent effects). Thus, while we were able to achieve partial regioselectivity prediction for the radical addition, general and reliable prediction would likely require considerably more computational effort.

### Mechanistic Studies

To gain insight into the mechanism of the photoredox-catalyzed C–H alkylation, a series of control and radical trapping experiments was conducted. The reaction was first performed in the presence of 3 equiv of TEMPO (2,2,6,6-tetramethylpiperidine-1-oxyl), a well-known radical scavenger, under standard conditions. In this case, complete inhibition of the desired product (**3**) formation was observed and the benzylic radical trapped product **3g** (Figure 8) was detected in HRMS, indicating that the transformation proceeds through a radical-mediated pathway. To further support this hypothesis, a radical trapping experiment was carried out using 1,1-diphenylethylene. Analysis by GC-MS, ^1^H NMR and high-resolution mass spectrometry (HRMS) revealed the formation of both the expected C–H alkylated product and an additional adduct (compounds **83** Figure 8) consistent with the trapping of an alkyl radical by 1,1-diphenylethylene. These results strongly support the intermediacy of an alkyl radical species during the reaction. Further mechanistic insight was obtained from time-resolved photoluminescence quenching studies to probe the interaction between the excited-state photocatalyst and key reaction components. The organic photocatalyst 4CzIPN, which efficiently absorbs visible light, possesses excited-state redox potentials of *E*_1/2_(PC*/PC^•–^) = +1.4 V vs SCE and *E*_1/2_(PC^•+^/PC*) = –1.2 V vs SCE.^75^ Quenching experiments demonstrated that 2-phenylacetateBTMGH (compound **2c**, see section 5 in the Supporting Information for more details) quenches the excited state of 4CzIPN at a significantly faster rate than 1-methyl-4-nitrobenzene (compound **1a**), suggesting that single-electron oxidation of the carboxylate is the kinetically favored process. This observation is consistent with the oxidation potential of typical carboxylates, which fall within the range of +0.99 to +1.25 V vs SCE,^76^ making them well-suited for oxidation by the excited state of 4CzIPN. Taken together, these experimental findings, along with precedent in the literature, support a mechanistic pathway in which photoredox-mediated single-electron oxidation of the carboxylate by the photocatalyst’s excited state triggers decarboxylation, generating an alkyl radical (**I**). This radical subsequently adds to the *ortho* and *para* positions of the arene ring of the nitroarene substrate (see Figure 7 for further details), forming a radical intermediate (**II**). This intermediate then undergoes oxidation by the photocatalyst to yield a cationic species (**III**), which upon deprotonation, delivers the final alkylated product (**3**). Alternatively, intermediate **II** may undergo a concerted proton-coupled electron transfer (PCET, see section 5.5.5 in the Supporting Information for a more details) process, thereby avoiding formation of a discrete nitroarene cation intermediate (III) and directly affording the alkylated product (**3**). Concurrently, the reduced state of the photocatalyst can transfer an electron to the nitroarene, promoting its stepwise reduction to the corresponding nitrosoarene and, ultimately, to the aniline (a net six-electron/six-proton process).^43,44,49,77^ This sequence regenerates the ground-state photocatalyst, thereby completing the catalytic cycle. Consistent with this proposal, significant amounts of the nitroarene reduction products (nitrosoarene and aniline) were observed when electron donors were employed under identical reaction conditions (see control experiments, Figure 8). This observation indicates that a sacrificial quantity of nitroarene is required for photocatalyst turnover. In addition, a reduced form of the alkylated product (**3f**, Figure 8) was detected in GC-MS, likely arising from a sequential alkylation-reduction pathway (see section 5.5 in the Supporting Information for a more detailed mechanistic discussion).

**Figure 8:**
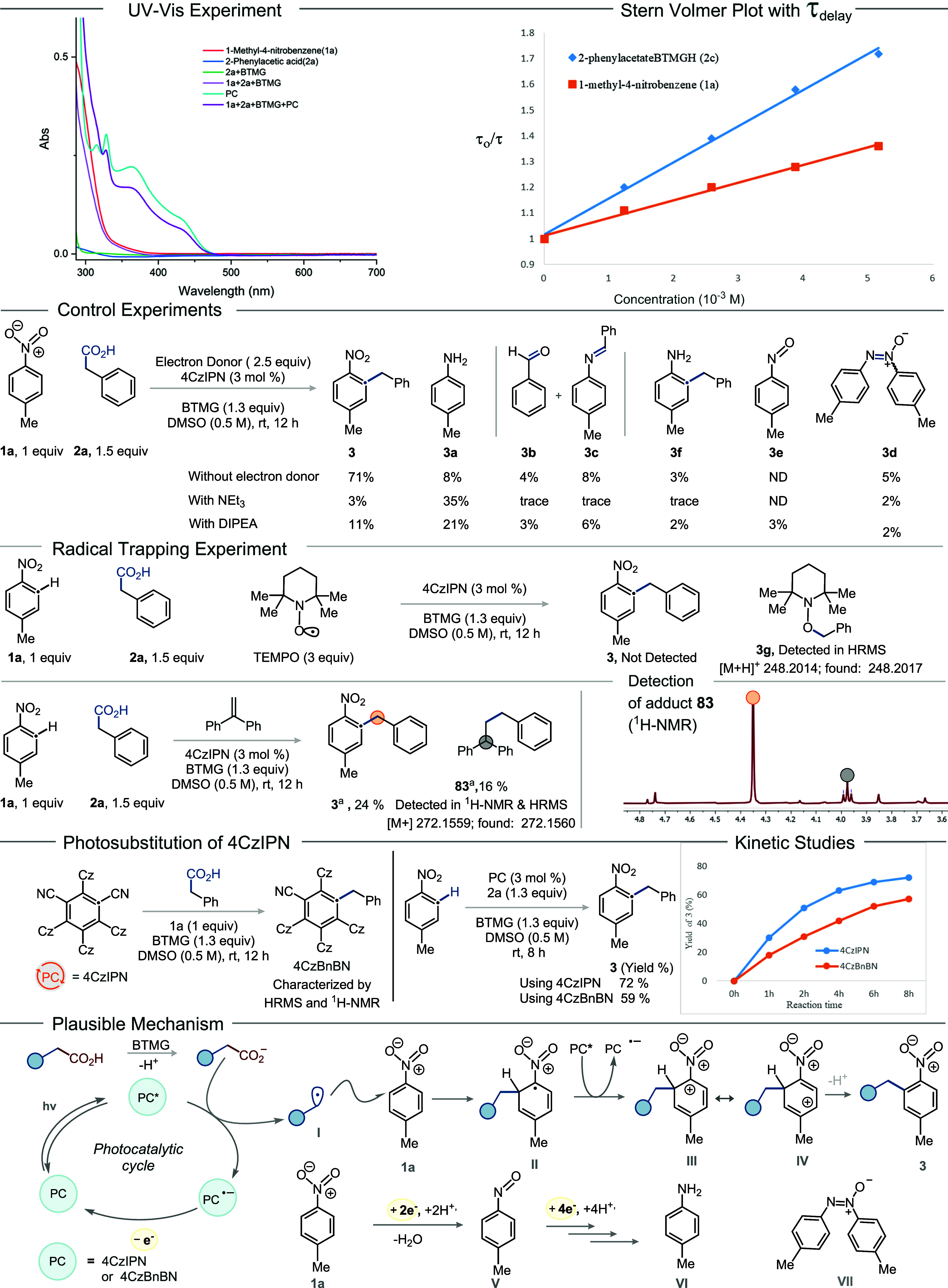
Spectroscopic investigations, control experiments, and a plausible mechanism for C­(sp^2^)–H alkylation reactions of nitroarenes. UV–vis experiment along with time-resolved quenching studies of 4CzIPN with 2-phenylacetateBTMGH (**2c**, BTMGH = 2-(*tert*-butyl)-1,1,3,3-tetramethylguanidinium), and 1-methyl-4-nitrobenzene (**1a**). Yields are calculated by GC and GC-MS analysis using 1,3-dimethoxybenzene as an internal standard. ^a^Yields were determined by ^1^H NMR spectroscopy using dibromomethane as an internal standard.

However, we also considered the possibility that DMSO may serve as a co-oxidant, as it has been reported to act as an oxidant under photochemical conditions.^78,79^ To probe this hypothesis, control experiments were conducted using sulfinyldibenzene and tetrahydrothiophene 1-oxide as a DMSO variant, under the standard reaction conditions. The corresponding reduction product of sulfinyldibenzene (product **86**, see section 5.5.5 in the Supporting Information) was detected by GC-MS, providing evidence that a sulfoxide can act as an oxidant under the present conditions.

Recent studies from our group have demonstrated that photosubstitution of 4CzIPN can occur under related conditions, wherein benzylic radicals substitute one of the nitrile groups of 4CzIPN to afford a blue-shifted derivative (4CzBnBN).^80–83^ Because benzylic radicals are also generated in the present system, formation of this photosubstituted species (4CzBnBN) was similarly detected in HRMS and confirmed by 1H-NMR spectroscopy (Figure 8, photosubstitution of 4CzIPN, see section 5 in the Supporting Information for further details). These observations raised the possibility that 4CzBnBN might participate in catalysis. To evaluate its catalytic competency, we evaluated the activity of the photosubstituted photocatalyst by employing 3 mol % of 4CzBnBN under the standard conditions for the nitroarene C­(sp^2^)–H benzylation (Figure 8, photosubstitution of 4CzIPN). When 4CzBnBN was used as the photocatalyst, the desired C–H alkylation product was obtained in lower yield (59%) compared with 4CzIPN (72%), indicating reduced efficiency. Reaction-kinetic analysis of the nitroarene C­(sp^2^)–H benzylation further corroborated the lower catalytic activity of 4CzBnBN relative to 4CzIPN.

## Conclusion

We have developed a modular and robust strategy for efficient C­(sp^2^)–H functionalization of nitroarenes using simple carboxylic acid derivatives as alkylating agents under mild conditions. The method is also compatible with alkyltrifluoroborates as alternative alkyl radical precursors. By controlling photoredox conditions, alkyl radicals are selectively directed to add to the aromatic π-system while preserving the nitro group, thereby suppressing oxygen-atom transfer, reduction, and nitro-group interception pathways that typically dominate nitroarene photochemistry. This approach significantly expands the synthetic utility of nitroarenes, enabling sequential C–H alkylation. The wide substrate compatibility, high functional-group tolerance, and operational simplicity render this method particularly well suited for late-stage functionalization and drug diversification. We anticipate that these findings will inspire further exploration of nitroarene reactivity in radical-based transformations and expand their utility in synthetic and medicinal chemistry.

## Experimental Section

Detailed experimental procedures and complete characterization data for all compounds are provided in the .

## Supplementary Material





## Notes

Additional computational data (ZIP)
Experimental details, optimization of the reaction, mechanistic experiments, theoretical analysis of regioselectivity, X-ray structure of **74**, characterization and crystal data, NMR spectra of new compounds (PDF)
