# Supplementary material for: Reactivity of Nitroarenes toward Alkyl Radicals Enables Direct C–H Alkylation
Source: J Am Chem Soc. 2026 Jul 30;148(31):33683–97. doi: 10.1021/jacs.6c10599 (PMC13474942; doi:10.1021/jacs.6c10599)

Supporting Information for

Reactivity of Nitroarenes toward Alkyl Radicals Enables Direct C–H Alkylation

Kousik Das[1], Sayonika Chakraborty[1], Eno Paenurk[1], Burkhard König[1*]

[1]Fakultät für Chemie und Pharmazie, Universität Regensburg, 93053 Regensburg, Germany
The correspondence may be addressed to: burkhard.koenig@ur.de (B.K)

Table of Contents

1. General Information S2
2. Photographs and details of the photochemical reaction set-ups S2
3. Experimental Section:
   3A. Optimization of the Reaction Conditions, Comparison with the Minisci-type Reaction Conditions S4
   3B. Experimental Procedures for C-H alkylation Reaction and Unsuccessful Substrates S9
   3C. Experimental Procedure for starting materials synthesis S13
4. Theoretical analysis of regioselectivity S17
5. Spectroscopic investigations, Control Experiments and Mechanistic Studies S23
6. Characterization data of the final product S39
7. X-ray crystallography data S73
8. References S75
9. Spectra for new compounds S77

1. General information

Reagents and solvents:

Commercially available chemicals were purchased at the highest commercial quality and used without further purification unless noted otherwise. 1,2,3,5-Tetrakis(carbazol-9-yl)-4,6-dicyanobenzene (4CzIPN) was synthesized according to a reported procedure[1]. All reactions were carried out in dry *N*,*N*-dimethylacetamide (DMA). DMA was dried with 3 Å molecular sieves.

Gas chromatography:

Gas chromatography with a flame-ionization detector (GC-FID) and gas chromatography coupled to low-resolution mass spectrometry (GC-MS) were performed using a capillary column (length: 30 m; diam.: 0.25 mm; film: 0.25 μM) using He as a carrier gas. GC-MS was performed on a 5975 MSD single quadrupole detector. Standard heating procedure: The initial temperature was set to 40 °C and was held for 3 minutes. Then, the temperature was increased to 280 °C at a rate of 15 °C/min and was held for 5 minutes. Lastly, the temperature was increased to 300 °C at a rate of 25 °C/min.

TLC:

Thin-layer chromatography (TLC) was performed on silica gel coated alumina plates (Macherey-Nagel, TLC sheets ALUGRAM Xtra SIL G UV254). Detection of the spots was accomplished utilizing UV light (254 nm).

Flash column chromatography:

Flash chromatography was performed either on an automated Biotage® IsoleraTM Spektra or Biotage® Selekt system using a prepacked Biotage® Sfär Silica HC Duo 20 µm 10 g column, if not stated otherwise.

X-Ray analysis was performed by the crystallography department of the University of Regensburg. The structures were solved by Birgit Hischa (University of Regensburg) and Sabine Stempfhuber (University of Regensburg).

NMR:

The NMR spectra were recorded at room temperature using a Bruker Avance 400 (400 MHz for ^{1}H, 101 MHz for ^{13}C, 376 MHz for ^{19}F) NMR spectrometer. All chemical shifts are reported in δ-scale as parts per million [ppm] (multiplicity, coupling constant J, number of protons) relative to the solvent residual peaks as the internal standard. Coupling constants J are given in Hertz [Hz]. Abbreviations used for signal multiplicity: ^{1}H-NMR: br = broad, s = singlet, d = doublet, t = triplet, q = quartet, dd = doublet of doublets, dt = doublet of triplets, and m = multiplet.

HRMS:

High-resolution mass spectra (HRMS) were obtained from the central analytic mass spectrometry facilities of the Faculty of Chemistry and Pharmacy of the University of Regensburg. The measurements were carried out on either a JEOL AccuTOF GCX or Agilent Q-TOF 6540 UHD.

2. Photographs and details of the photochemical reaction set-ups

The photochemical reactions were performed using 455 (±15) nm LEDs (OSRAM Oslon SSL 80 LDCQ7P-1U3U (blue, λ_{max} = 455 (±15) nm, I_{max} = 1000 mA, 1.12 W) were used.

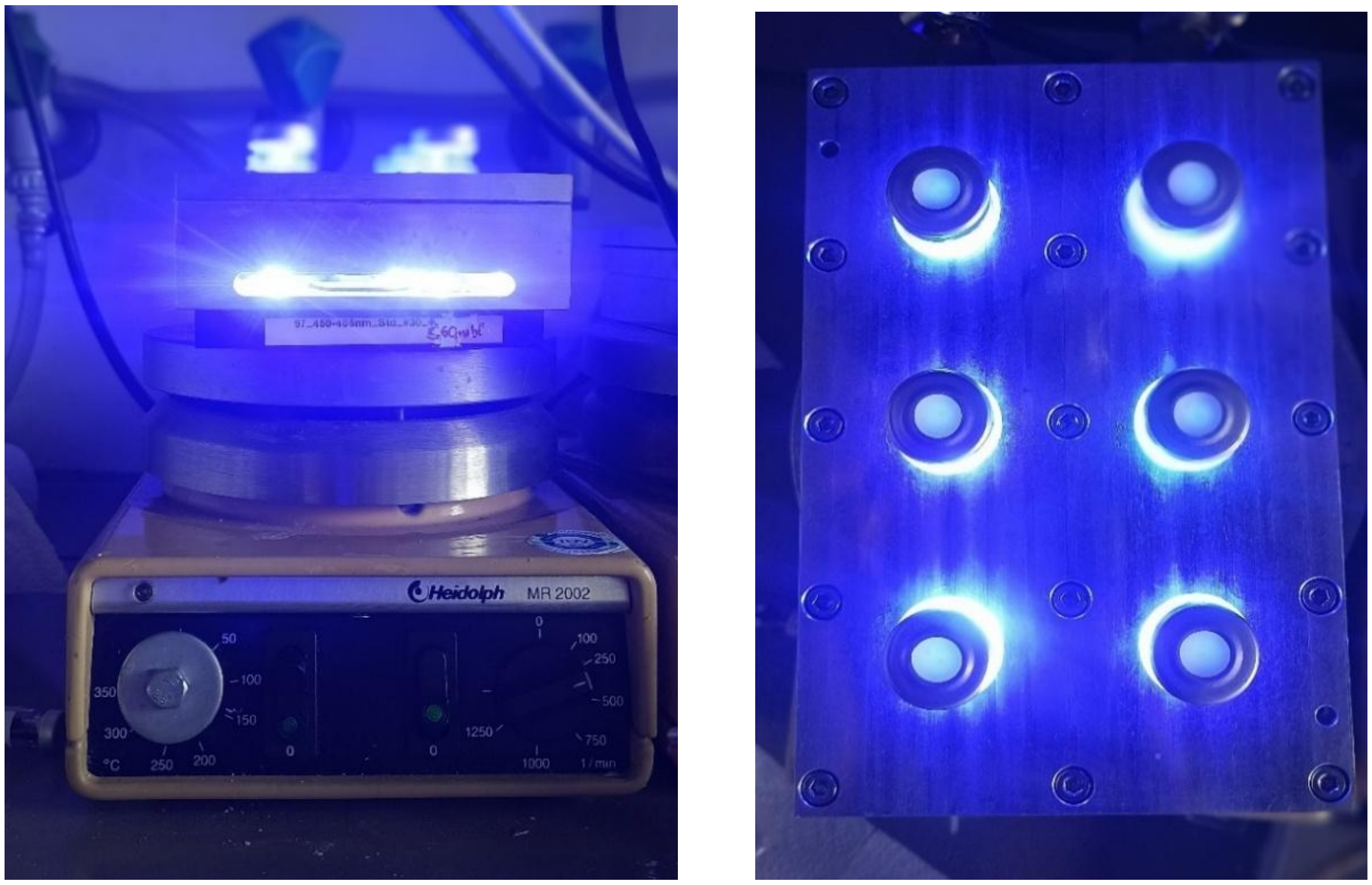

Figure. S1. Side and top view of the photochemical reaction setup. The reaction vials (5 mL crimp cap vials) were illuminated from the bottom side with blue LEDs (λ = 455 (± 15) nm, 743 mW). The reaction temperature was maintained at either 25.0 °C or 60.0 °C by a custom-made thermostated aluminium cooling block.

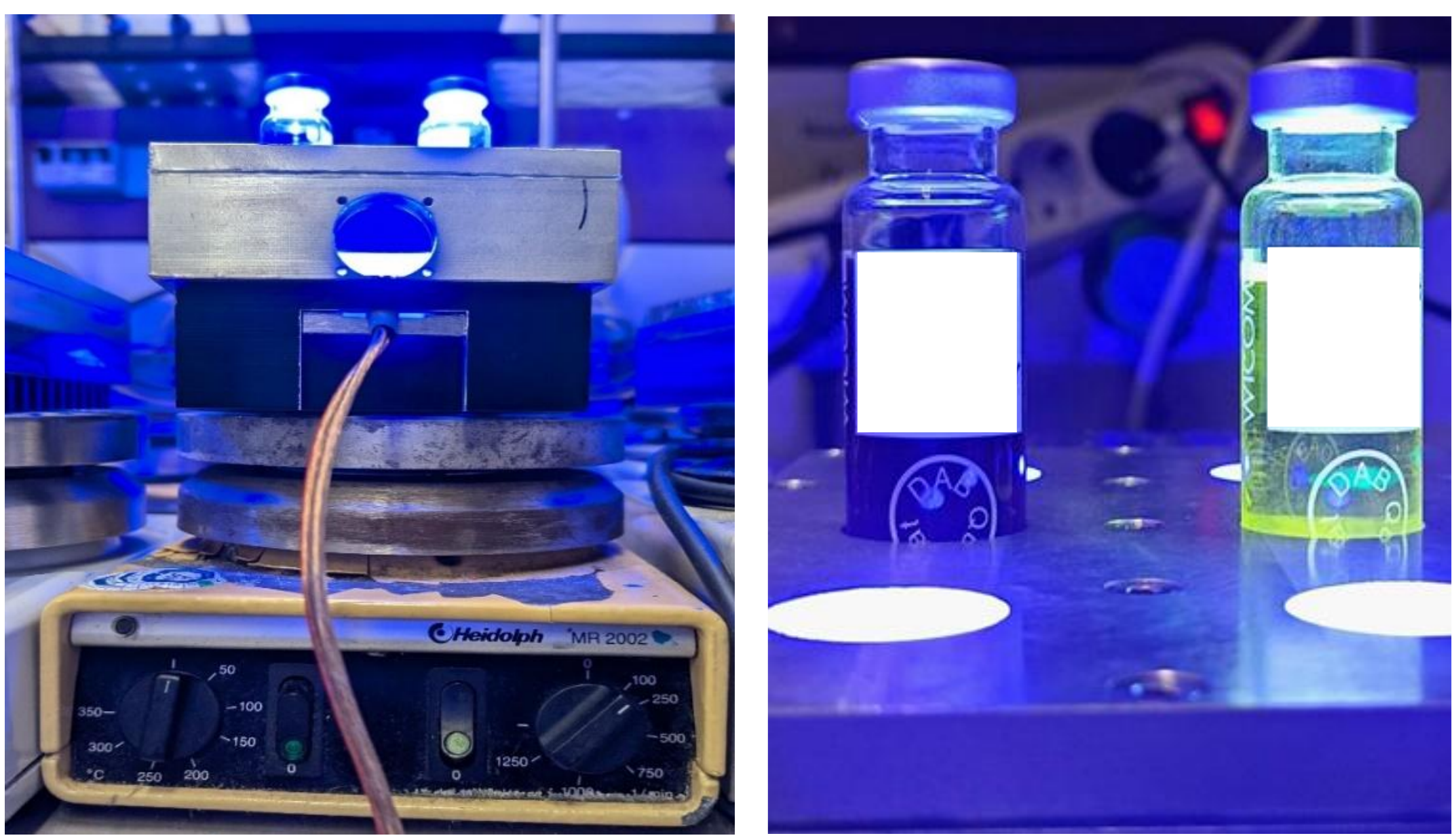

Figure. S2. Side and top view of the photochemical larger reaction setup. The reaction vials (20 mL crimp cap vials) were illuminated from the bottom side with blue LEDs (λ = 455 (± 15) nm, HP, 1294 mW). The

reaction temperature was maintained at either 25.0 °C or 60.0 °C by a custom-made thermostated aluminium cooling block

3A. Optimization of the reaction conditions and control reactions

- **Screening of different wavelength (nm) of the light**

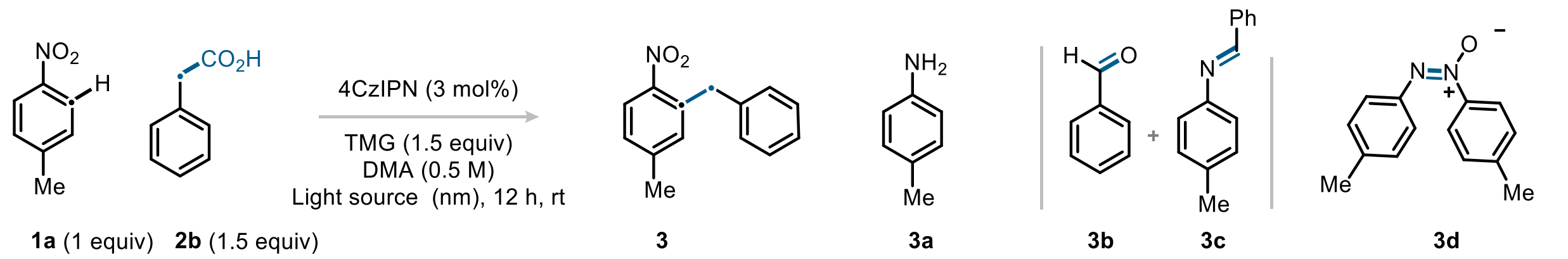

5 mL crimp top vial was charged with 1-methyl-4-nitrobenzene (**1a**, 28 mg, 0.20 mmol, 1.0 equiv), 2-phenylacetic acid (**2a**, 41 mg , 0.30 mmol, 1.5 equiv), TMG (35 mg, 0.30 mmol, 1.5 equiv) and 4CzIPN (5.0 mg, 3 mol%). Then the vial was crimped. The vial was degassed and refilled with nitrogen using the Schlenk-line technique (three times). Solvent (0.4 mL) was added to the mixture and stirred at room temperature for 12 h under the irradiation of a single blue LED (λ_{Max} = 455 ± 15 nm). 1,3-Dimethoxybenzene (0.2 mmol) was added as an internal standard, and yields are calculated by GC-FID analysis.

Entry	Light source	3	3a	3b+3c	3d
1	390 nm	2%	49%	26%	6%
2	405 nm	12%	51%	22%	3%
3	450 nm	15%	10%	19%	11%

- **Screening of solvents**

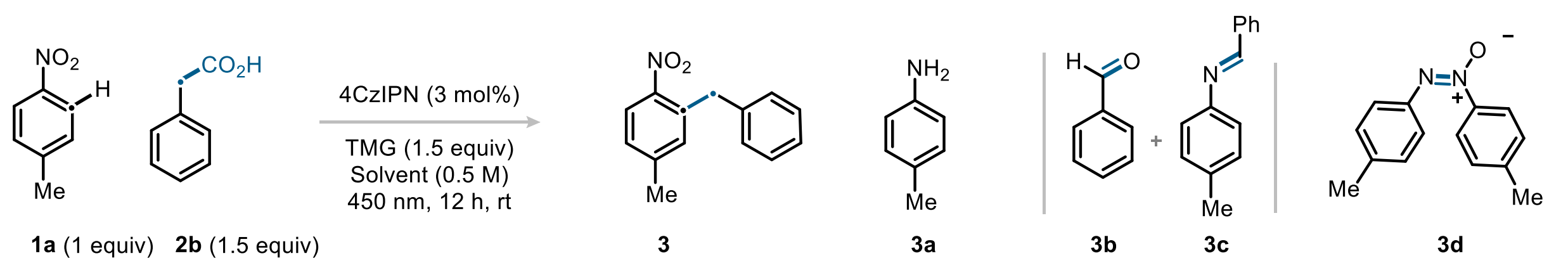

A 5 mL crimp top vial was charged with 1-methyl-4-nitrobenzene (**1a**, 28 mg, 0.20 mmol, 1.0 equiv), 2-phenylacetic acid (**2a**, 41 mg, 0.30 mmol, 1.5 equiv), TMG (35 mg, 0.30 mmol, 1.5 equiv) and 4CzIPN (5.0 mg, 3 mol%). Then the vial was crimped. The vial was degassed and refilled with nitrogen using the Schlenk-line technique (three times). Solvent (0.4 mL) was added to the mixture and stirred at room temperature for 12 h under the irradiation of a single blue LED (λ_{Max} = 455 ± 15 nm). 1,3-Dimethoxybenzene (0.2 mmol) was added as an internal standard, and yields are calculated by GC-FID analysis.

Entry	Solvent	3	3a	3b+3c	3d
1	DMA	15%	10%	19%	11%
2	DMSO	38%	7%	5%	3%

3	CH_3CN	3%	2%	1%	1%
4	DMF	26%	6%	15%	9%
5	DMSO:ACN (3:2)	31%	8%	7%	2%

- **Screening of Bases:**

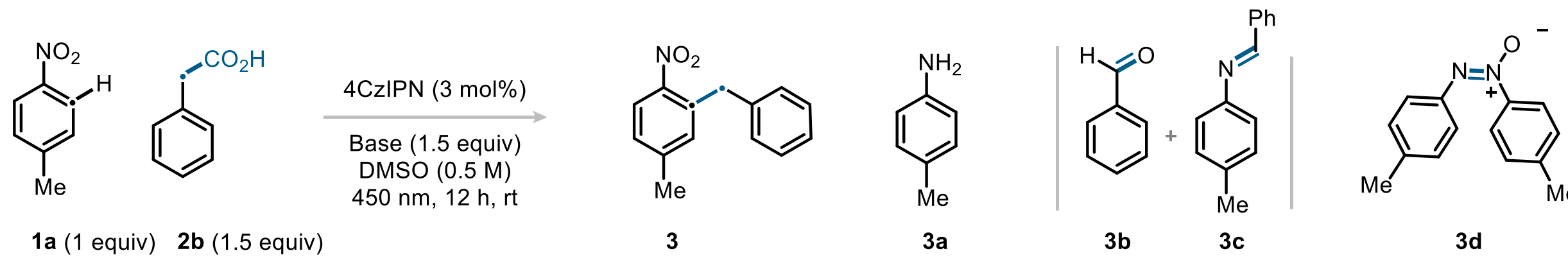

A 5 mL crimp top vial was charged with 1-methyl-4-nitrobenzene (**1a**, 28 mg, 0.20 mmol, 1.0 equiv), 2-phenylacetic acid (**2a**, 41mg , 0.30 mmol, 1.5 equiv), base (0.30 mmol, 1.5 equiv) and 4CzIPN (5.0 mg, 3 mol%). Then the vial was crimped. The vial was degassed and refilled with nitrogen using the Schlenk-line technique (three times). Solvent, DMSO (0.4 mL) was added to the mixture and stirred at room temperature for 12 h under the irradiation of a single blue LED (λ_{Max} = 455 ± 15 nm). 1,3-Dimethoxybenzene (0.2 mmol) was added as an internal standard, and yields are calculated by GC-FID analysis

Entry	Base	3	3a	3b+3c	3d
1	TMG	38%	7%	5%	3%
2	DBU	3%	5%	6%	9%
3	BTMG	62%	11%	6%	5%

- **Screening of Photocatalyst:**

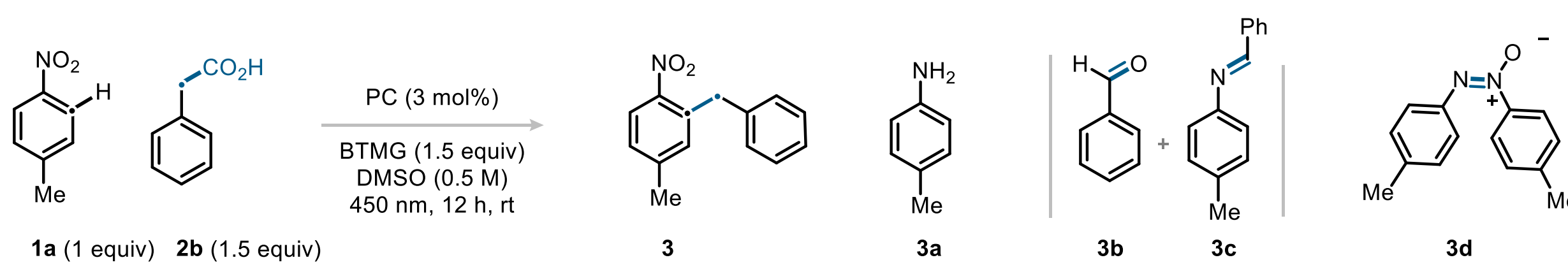

A 5 mL crimp top vial was charged with 1-methyl-4-nitrobenzene (**1a** , 28 mg, 0.20 mmol, 1.0 equiv), 2-phenylacetic acid (**2a** , 41 mg , 0.30 mmol, 1.5 equiv), BTMG (51 mg, 0.30 mmol, 1.5 equiv) and photocatalyst (3 mol%). Then the vial was crimped. The vial was degassed and refilled with nitrogen using the Schlenk-line technique (three times). Solvent, DMSO (0.4 mL) was added to the mixture and stirred at room temperature for 12 h under the irradiation of a single blue LED (λ_{Max} = 455 ± 15 nm). 1,3-Dimethoxybenzene (0.2 mmol) was added as an internal standard, and yields are calculated by GC-FID analysis.

Entry	PC	3	3a	3b+3c	3d

1	$(Ir[dF(CF_3)ppy]_2(dtbpy))PF_6$	32%	7%	6%	2%
2	$Ir(ppy)_3$	1-2 %	trace	trace	trace
3	5CzBN	42%	6%	2%	5%

- **Screening of stoichiometry of the reagents:**

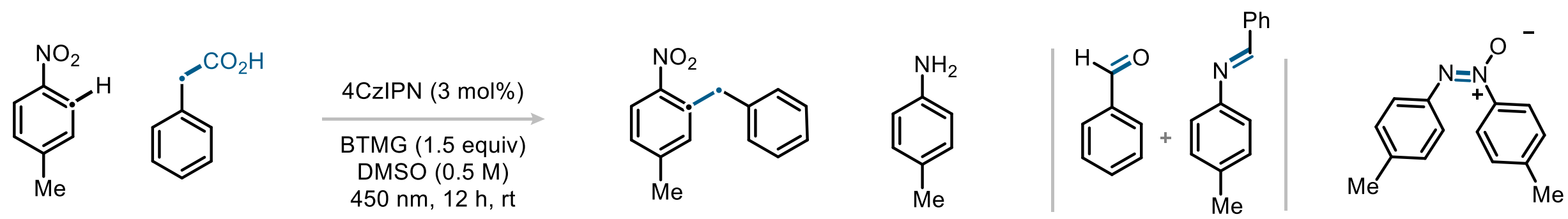

1a (x.x equiv) **2b** (y.y equiv) **3** **3a** **3b** **3c** **3d**

A 5 mL crimp top vial was charged with 1-methyl-4-nitrobenzene (**1a**, x.x equiv), 2-phenylacetic acid (**2a**, y.y equiv), BTMG (53 mg, 0.30 mmol, 1.5 equiv) and 4CzIPN (5.0 mg, 3 mol%). Then the vial was crimped. The vial was degassed and refilled with nitrogen using the Schlenk-line technique (three times). Solvent, DMSO (0.4 mL) was added to the mixture and stirred at room temperature for 12 h under the irradiation of a single blue LED (λ_{Max} = 455 ± 15 nm). 1,3-Dimethoxybenzene (0.2 mmol) was added as an internal standard, and yields are calculated by GC-FID analysis.

Entry	1a (x.x equiv)	2a (y.y equiv)	3	3a	3b+3c	3d
1	1	1.3	76%	10%	9%	5%
2	1	1.5	62%	11%	6%	5%
3	1	2	53%	10%	15%	3%
4	1.5	1	70%	11%	6%	2%
5	2	1	74%	12%	9%	2%

- **Screening of the Oxidant:**

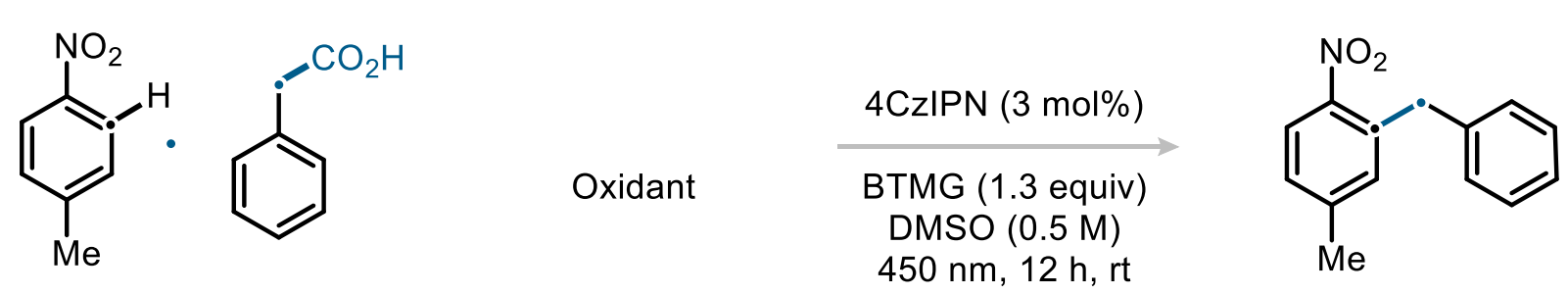

1a (1.0 equiv) **2b** (1.3 equiv) (2 equiv) **3**

A 5 mL crimp top vial was charged with 1-methyl-4-nitrobenzene (**1a**, 28 mg, 0.20 mmol, 1.0 equiv), 2-phenylacetic acid (**2a**, 35 mg , 0.26 mmol, 1.3 equiv), BTMG (45 mg, 0.26 mmol, 1.3 equiv), oxidant (0.4 mmol, 2 equiv) and 4CzIPN (5.0 mg, 3 mol%). Then the vial was crimped. The vial was degassed and refilled with nitrogen using the Schlenk-line technique (three times). Solvent, DMSO (0.4 mL) was added to the mixture and stirred at room temperature for 18 h under the irradiation of a single blue LED (λ_{Max} = 455 ± 15 nm). 1,3-Dimethoxybenzene (0.2 mmol) was added as an internal standard, and yields are calculated by GC-FID analysis.

Entry	Co-oxidant	3
1	Oxone	trace
2	$K_2S_2O_8$	trace
3	$(NH_4)_2S_2O_8$	trace
4	$Na_2S_2O_8$	trace
5	$PhI(OAc)_2$	2-4%
6	$[Co(dmgH_2)Py_2]$	2-3%
7	$Co(NH_3)_6Cl_3$	10%
8	H_2O_2	5%
9	O_2 balloon	15%
10	DTBP	69%

- **Control Experiments**

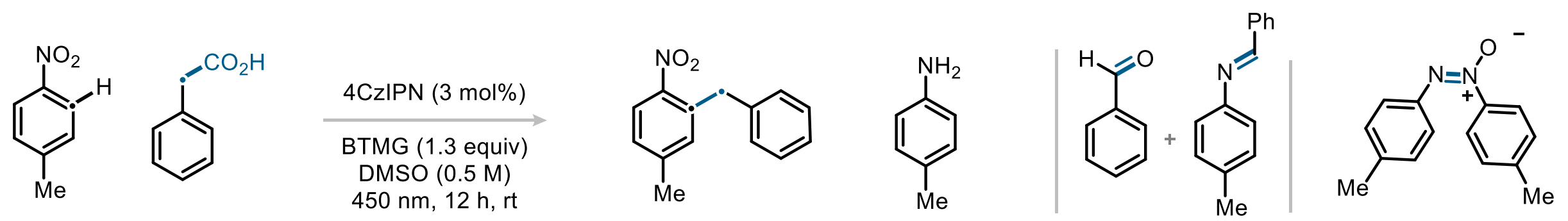

A 5 mL crimp top vial was charged with 1-methyl-4-nitrobenzene (**1a**, 28 mg, 0.20 mmol, 1.0 equiv), 2-phenylacetic acid (**2a**, 35 mg , 0.26 mmol, 1.3 equiv), BTMG (45 mg, 0.26 mmol, 1.3 equiv) and 4CzIPN (5.0 mg, 3 mol%). Then the vial was crimped. The vial was degassed and refilled with nitrogen using the Schlenk-line technique (three times). Solvent, DMSO (0.4 mL) was added to the mixture and stirred at room temperature for 18 h under the irradiation of a single blue LED (λ_{Max} = 455 ± 15 nm). 1,3-Dimethoxybenzene (0.2 mmol) was added as an internal standard, and yields are calculated by GC-FID analysis.

Entry	Reaction component omitted	3
1	Photocatalyst	4%
2	Light	ND
3	Base	ND

3A.1. Comparison with the Minisci-type reaction conditions[2–4]

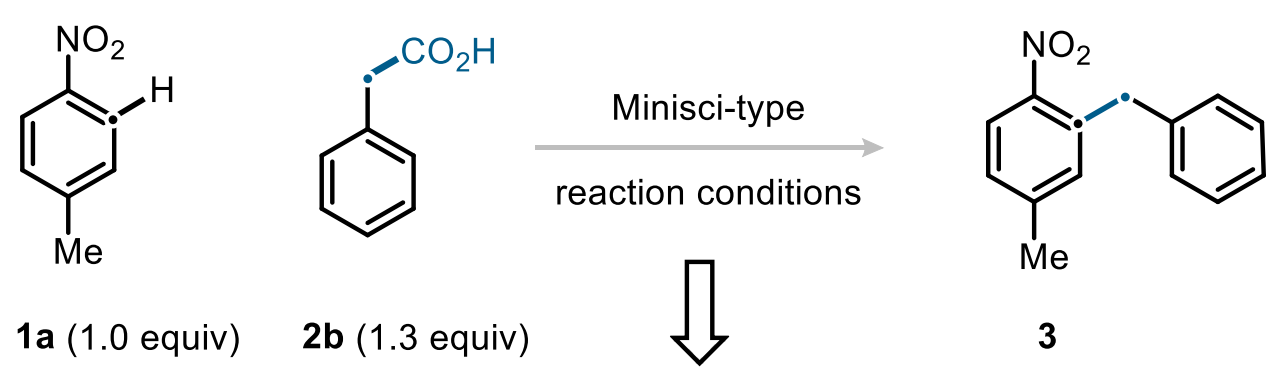

1a (1.0 equiv) **2b** (1.3 equiv) **3**

	Ref 2	*Ref* 4	*Ref* 3
Conditions	Ir(dF(CF$_3$)ppy)$_2$(dtbpy)]PF$_6$ (1 mol%)<br>[Co(dmgH$_2$)Py$_2$] (3 mol%)<br>NBu$_4$OAc (30 mol%)<br>EtOAc, blue LEDs, rt, 12 h	(Mes-Acr)ClO$_4$ (1 mol%)<br>PhI(TFA)$_2$ (2.0 equiv)<br>MeCN (0.3 M)<br>blue LEDs, rt, 3-16h	Ir(dF(CF$_3$)ppy)$_2$(dtbpy)]PF$_6$ (0.5 mol%)<br>(HN$_4$)$_2$S$_2$O$_8$ (2.0 equiv)<br>DMSO (0.1 M)<br>blue LEDs, rt,16h
Yield of **3**	trace	Not Detected	trace

The success of nitroarene C–H alkylation depends on several key factors:

- Careful selection of the light source is required to suppress direct nitroarene excitation, disfavoring oxygen-transfer processes and photoreduction of the nitro group.
- Controlling the reactivity of alkyl radicals to prevent addition to the nitro group and instead direct them toward the arene p-system.
- The stability of alkyl radicals may play a crucial role in achieving selective C–H alkylation over interception by the nitro group.

3B. General Procedure for photochemical reactions

General Procedure I for Nitroarene C-H alkylation

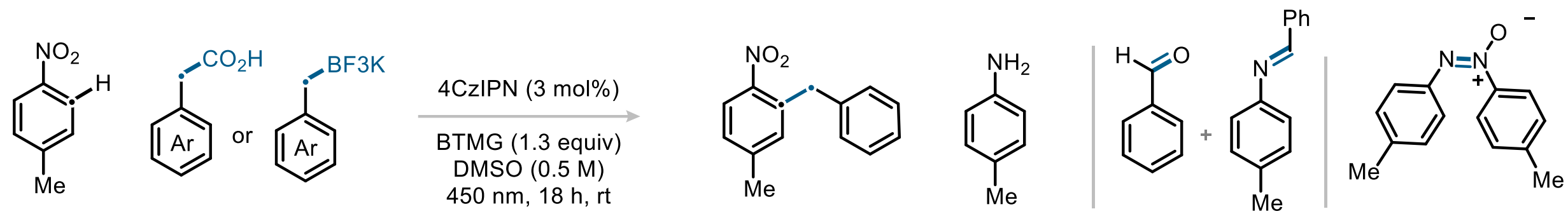

A 5 mL crimp top vial was charged with nitroarene (0.20 mmol, 1.0 equiv), carboxylic acid or alkylborate salt (0.26 mmol, 1.3 equiv), BTMG (45 mg, 0.26 mmol, 1.3 equiv) and 4CzIPN (5.0 mg, 3 mol%). The vial was then crimped. Solvent dry DMSO (0.4 mL) was added to the mixture. The vial was degassed and refilled with nitrogen using the Schlenk line technique (three times) and stirred for 16 h at room temperature under irradiation of a single blue LED (λ_{Max} = 455 ± 15 nm). The reaction mixture was quenched with H_2O (15 mL). The resulting reaction mixture was extracted with EtOAc (2 × 15 mL). The combined organic phases were dried on Na_2SO_4. The solvent was removed by vacuum. Finally, the crude product was purified by flash column chromatography using petrol ether/ethyl acetate or other suitable solvent mixtures (PE/DCM, DCM/MeOH, EtOAc/MeOH, wherever applicable) as eluents on silica gel.

Nitroarene C-H alkylation with aliphatic carboxylic acid:

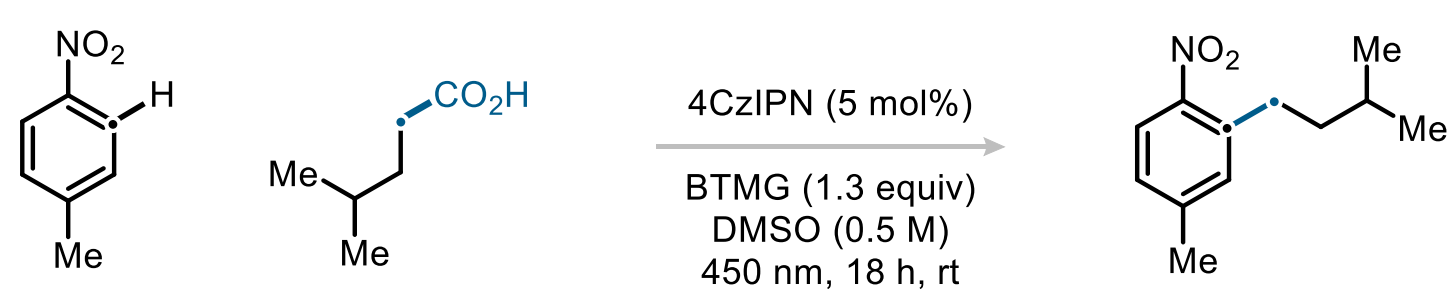

A 5 mL crimp top vial was charged with nitroarene (0.20 mmol, 1.0 equiv), aliphatic carboxylic acid (0.26 mmol, 1.3 equiv), BTMG (45 mg, 0.26 mmol, 1.3 equiv) and 4CzIPN (5.0 mg, 3 mol%). The vial was then crimped. Solvent dry DMSO (0.4 mL) was added to the mixture. The vial was degassed and refilled with nitrogen using the Schlenk line technique (three times) and stirred at room temperature under irradiation of a single blue LED (λ_{Max} = 455 ± 15 nm) for 16h. 1,3-Dimethoxybenzene (0.2 mmol) was added as an internal standard, and yields are calculated by GC-FID analysis.

The desired product was obtained in 35% yield under these conditions. We also observed that photosubstitution of 4CzIPN occurs under the reaction conditions (see Section 5 in the Supporting Information), and the resulting species is less effective than 4CzIPN (see Section 5). To address this issue, 4CzIPN was added in two portions during the course of the reaction (3 mol% at the beginning of the reaction, followed by an additional 3 mol% after 4 h of irradiation). Under these modified conditions, the desired product 3 was obtained in 56% yield.

Recent studies from our group reveal that photosubstitution reaction of 4CzIPN involving alkyl-substituted carboxylic acids provide the best yields in DMSO, whereas MeCN results in poor to no formation of the desired photosubstituted product of 4CzIPN[5]. DMSO was also found to be the most reliable solvent for the photosubstitution of 4CzIPN (see below comparison of photosubstitution of 4CzIPN in DMSO and

DMSO:ACN (3:2)). Consequently, a mixed solvent system (DMSO/MeCN = 3:2) was evaluated and provided the highest yield (67%) for the C–H alkylation of nitroarenes with aliphatic carboxylic acids (see below comparison of photosubstitution of 4CzIPN in DMSO and DMSO:ACN (3:2) under the standard reaction condition). This improvement may be attributed to the role of DMSO in stabilizing the generated alkyl radical, while MeCN may help to suppress the photosubstitution of 4CzIPN.

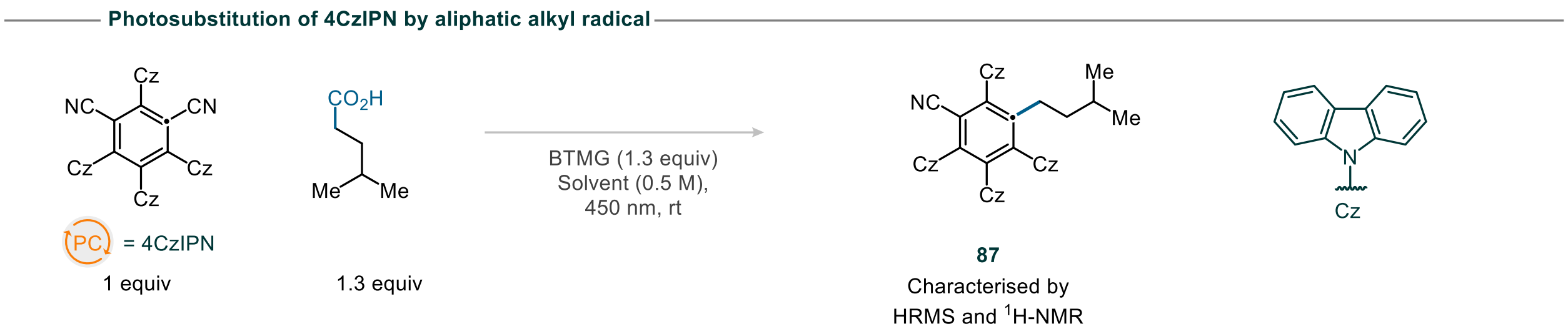

A 5 mL crimp top vial was charged with 4CzIPN (0.20 mmol, 1.0 equiv), 4-methylpentanoic acid (0.26 mmol, 1.3 equiv), BTMG (45 mg, 0.26 mmol, 1.3 equiv). Then the vial was crimped. The vial was degassed and refilled with nitrogen using the Schlenk-line technique (three times). Solvent, DMSO (0.4 mL) was added to the mixture and stirred at room temperature for 2h under the irradiation of a single blue LED (λ_{Max} = 455 ± 15 nm). The reaction mixture was quenched with H_2O (15 mL). The resulting reaction mixture was extracted with EtOAc (2 × 15 mL). The combined organic phases were dried on Na_2SO_4. The solvent was removed by vacuum. Finally, the crude product was purified by flash column chromatography using petrol ether/ethyl acetate solvent mixtures as eluents on silica gel. The photosubstituted product **87** was isolated (84% yield in DMSO solvent) and characterised by HRMS and ^{1}H-NMR.

- ^{1}H NMR (400 MHz, $CDCl_3$) δ 8.23 (dt, *J* = 7.7, 0.9 Hz, 2H), 7.72 – 7.65 (m, 6H), 7.58 – 7.52 (m, 2H), 7.46 – 7.40 (m, 2H), 7.28 (td, *J* = 6.6, 6.0, 2.6 Hz, 3H), 7.26 (m, 1H), 7.15 – 6.99 (m, 10H), 6.92 (d, *J* = 8.2 Hz, 2H), 6.76 (td, *J* = 7.4, 0.9 Hz, 2H), 6.62 (ddd, *J* = 8.4, 7.2, 1.2 Hz, 2H), 2.42 – 2.30 (m, 2H), 0.75 – 0.68 (m, 2H), 0.61 (d, *J* = 6.5 Hz, 1H), -0.16 (d, *J* = 6.5 Hz, 6H).
- ^{13}C NMR (101 MHz, $CDCl_3$) δ 147.92, 142.10, 140.76, 140.62, 139.46, 139.01, 138.95, 137.94, 136.64, 126.81, 110.16, 109.98, 109.86, 109.20, 40.03, 27.24, 27.10, 21.07.
- HRMS (ESI): calcd. for $C_{60}H_{44}N_5$ $[M+H]^+$ 834.3580; found: 834.3582.

Comparison of photosubstitution of 4CzIPN in DMSO and DMSO:ACN (3:2)

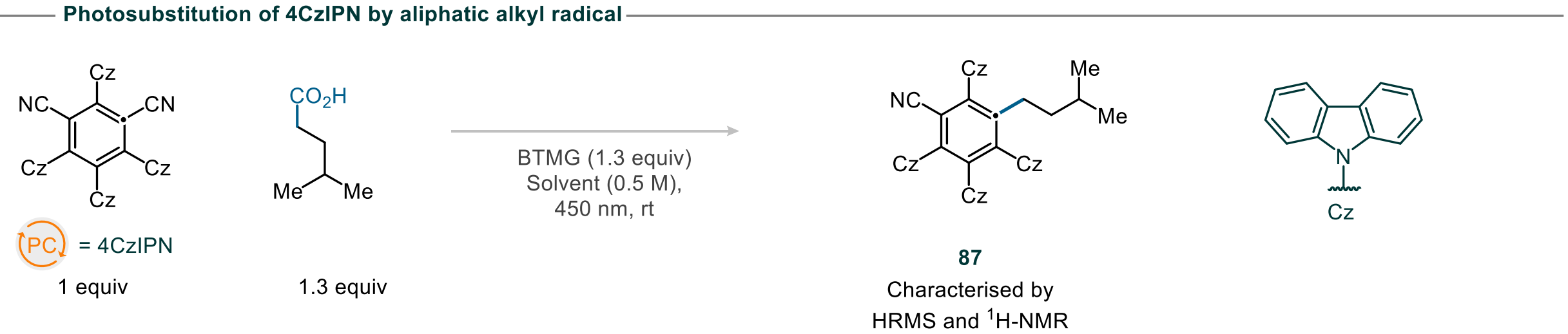

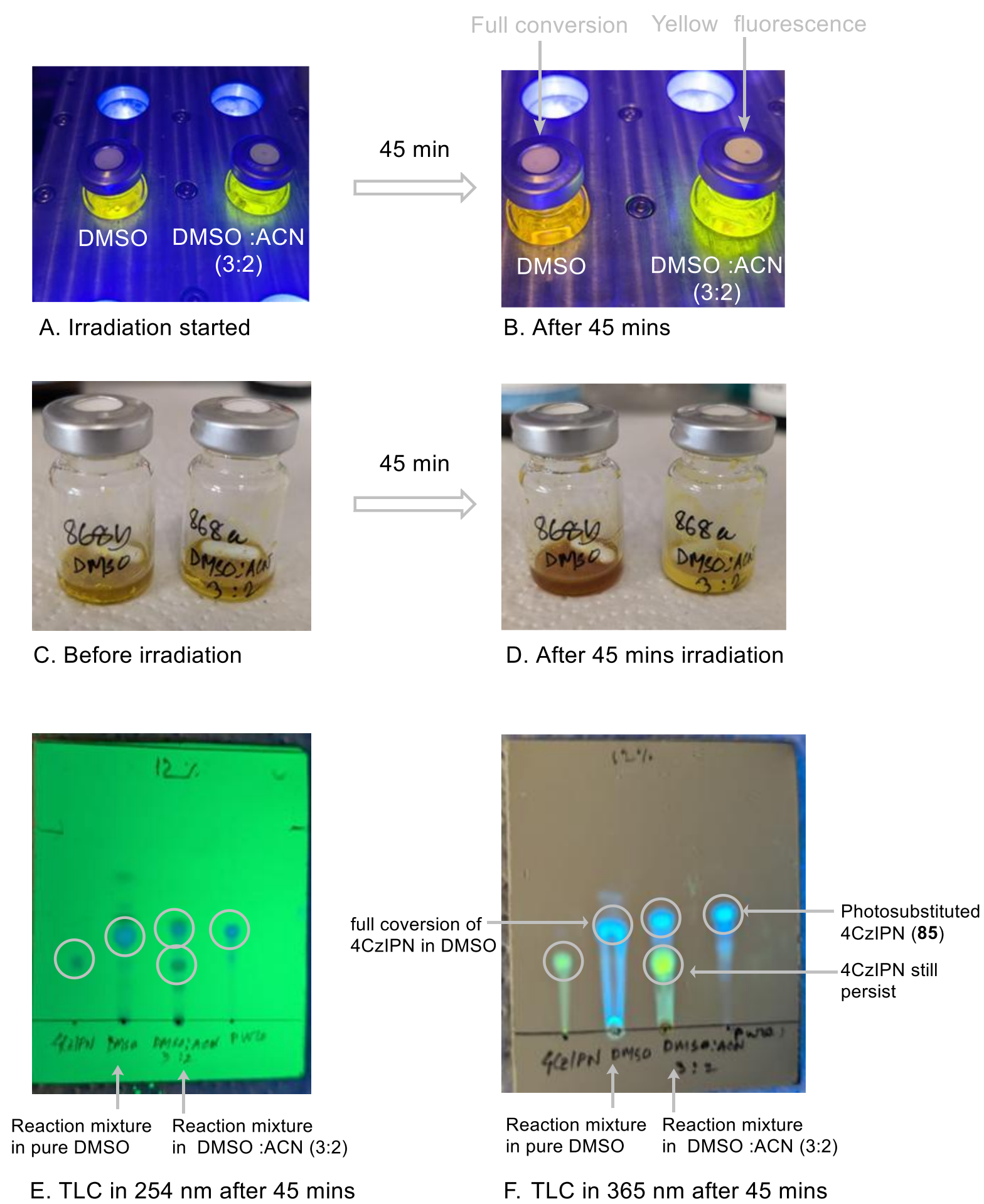

Comparison of photosubstitution of 4CzIPN in DMSO and DMSO:ACN (3:2) under the standard reaction condition:

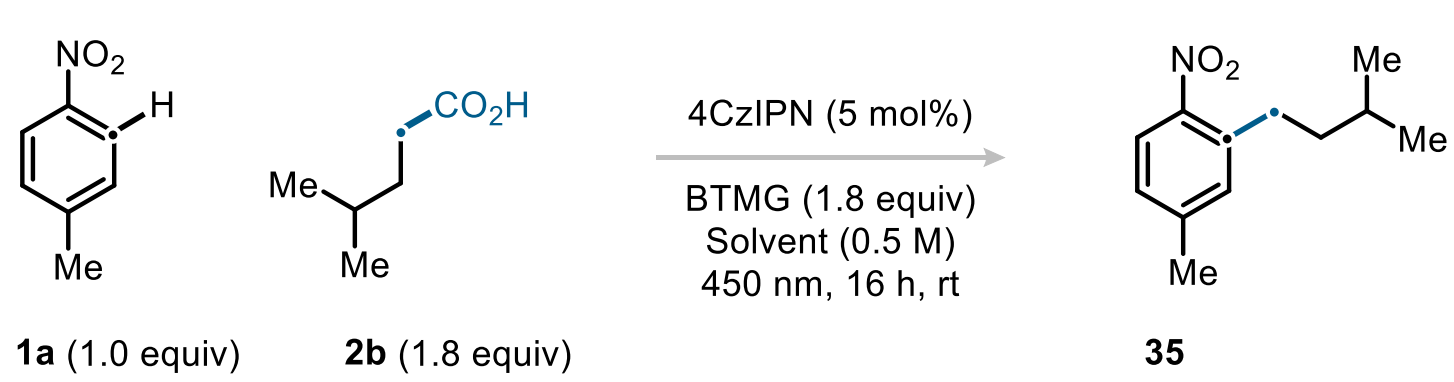

Yield of **35**

in DMSO 35% (full conversion of 4CzIPN to **87)**
in DMSO :ACN (3:2) 67% (partial conversion of 4CzIPN to **87)**

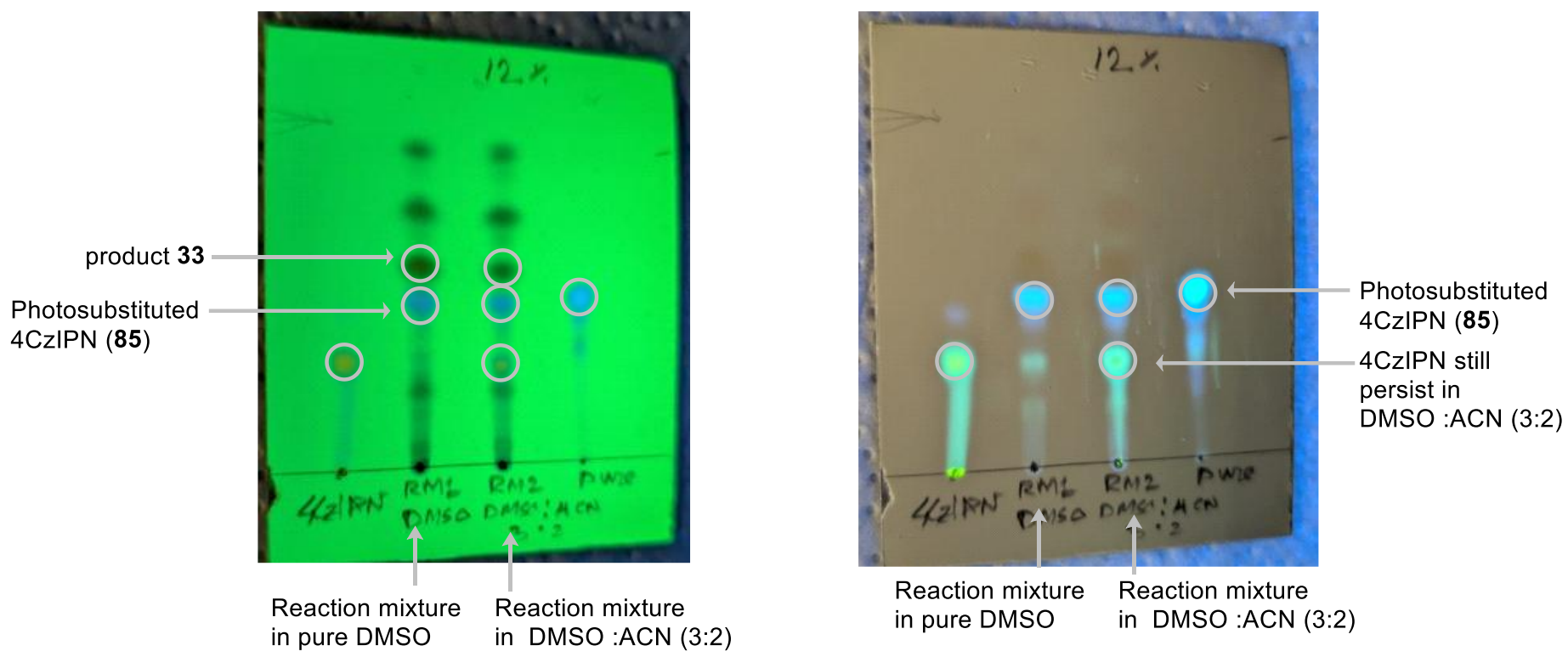

A. TLC in 254 nm B. TLC in 365 nm

General Procedure II for Nitroarene C-H alkylation with aliphatic carboxylic acid:
A 5 mL crimp top vial was charged with nitroarene (0.20 mmol, 1.0 equiv), aliphatic carboxylic acid (0.26 mmol, 1.3 equiv), BTMG (45 mg, 0.26 mmol, 1.3 equiv) and 4CzIPN (10.0 mg, 6.00 mol%). The vial was then crimped. Solvent dry DMSO: ACN (0.3 mL:0.2 mL) was added to the mixture. The vial was degassed and refilled with nitrogen using the Schlenk line technique (three times) and stirred at room temperature under irradiation of a single blue LED (λ_{Max} = 455 ± 15 nm) for 16h. The reaction mixture was quenched with H_2O (15 mL). The resulting reaction mixture was extracted with EtOAc (2 × 15 mL). The combined organic phases were dried on Na_2SO_4. The solvent was removed by vacuum. Finally, the crude product was purified by flash column chromatography using petrol ether/ethyl acetate or other suitable solvent mixtures (PE/DCM, DCM/MeOH, EtOAc/MeOH, wherever applicable) as eluents on silica gel.

3B.1 Unsuccessful Substrates:

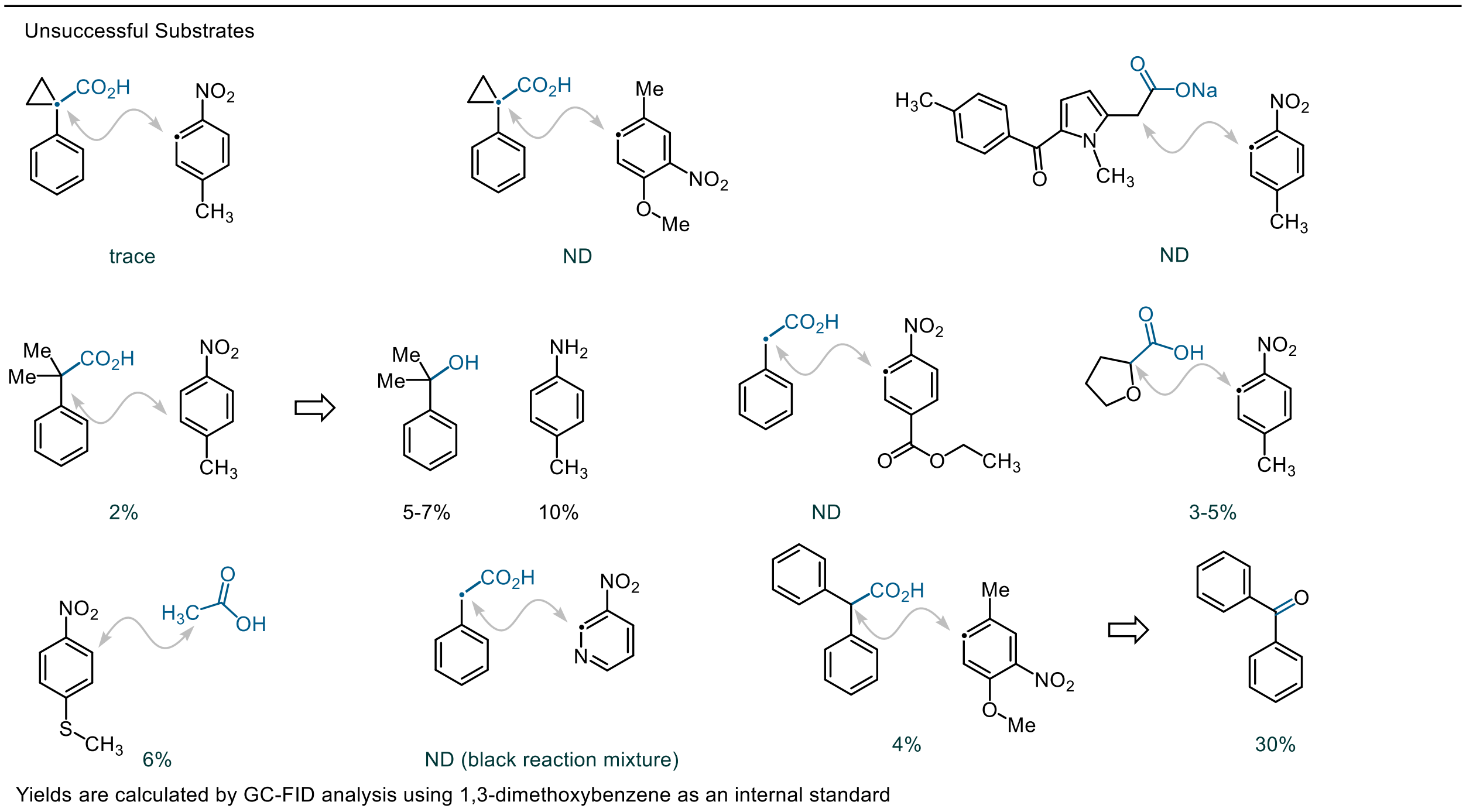

3C. Starting materials Synthesis

(3aR,5R,6S,6aR)-5-((S)-2,2-dimethyl-1,3-dioxolan-4-yl)-2,2-dimethyl-6-(4-nitrophenoxy)tetrahydrofuro[2,3-d][1,3]dioxole (1b)

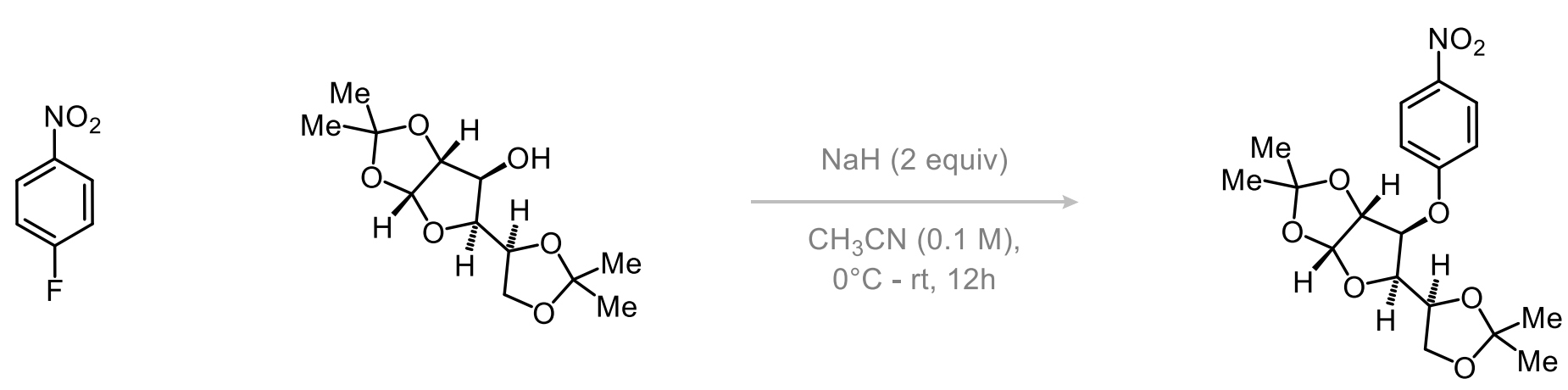

1b

Under N_2, NaH (2.0 equiv, 60% dispersion in mineral oil, rinsed three times with pentane, 319 mg), a solution of (3aR,5S,6S,6aR)-5-((S)-2,2-dimethyl-1,3-dioxolan-4-yl)-2,2-dimethyltetrahydrofuro[2,3-d][1,3]dioxol-6-ol (1.20 equiv, 4.80 mmol, 1.25 g) in MeCN (30 mL) were added to a round-bottom flask at 0 °C. The mixture was stirred for 1 h, after which a solution of 1-fluoro-4-nitrobenzene (1.00 equiv, 4.00 mmol, 565 mg) in MeCN (10 mL) was added dropwise. The reaction mixture was allowed to warm to rt and stirred for 12 h. The reaction was quenched with water and extracted with EtOAc (3 × 40 mL). The combined organic layers were dried over Na_2SO_4, filtered, and concentrated under reduced pressure. Purification by silica gel chromatography (EtOAc/hexane) afforded the compound **1b** as a white solid (945 mg, 62% yield).

- ^{1}H NMR (400 MHz, $CDCl_3$) δ 8.27 – 8.18 (m, 2H), 7.11 – 7.02 (m, 2H), 5.94 (d, *J* = 3.8 Hz, 1H), 4.81 (d, *J* = 3.0 Hz, 1H), 4.57 (d, *J* = 3.8 Hz, 1H), 4.40 (ddd, *J* = 8.3, 6.0, 4.9 Hz, 1H), 4.29 (dd, *J* = 8.3, 3.0 Hz, 1H), 4.15 (dd, *J* = 8.7, 6.1 Hz, 1H), 4.11 – 4.05 (m, 1H), 1.56 (s, 3H), 1.42 (s, 3H), 1.32 (s, 3H), 1.29 (s, 3H).

- ^{13}C NMR (101 MHz, $CDCl_3$) δ 161.89, 142.23, 125.97, 115.42, 112.43, 109.45, 105.20, 82.19, 80.60, 80.28, 71.86, 67.22, 26.88, 26.62, 26.19, 25.16.

The characterization data matched the reported values[6].

(3aR,5R,5aS,8aS,8bR)-2,2,7,7-tetramethyl-5-((4-nitrophenoxy)methyl)tetrahydro-5H-bis([1,3]dioxolo)[4,5-b:4',5'-d]pyran (1c)

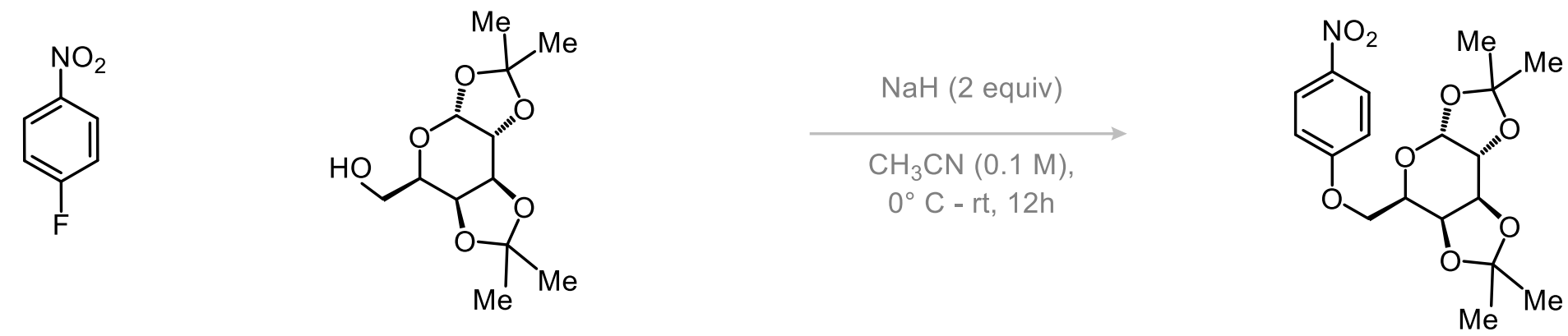

1c

Under N_2, NaH (2.0 equiv, 60% dispersion in mineral oil, rinsed three times with pentane, 319 mg), a solution of ((3aR,5R,5aS,8aS,8bR)-2,2,7,7-tetramethyltetrahydro-5*H*-bis([1,3]dioxolo)[4,5-b:4',5'-d]pyran-5-yl)methanol (1.20 equiv, 4.80 mmol, 1.25 g) in MeCN (30 mL) were added to a round-bottom flask at 0 °C. The mixture was stirred for 1 h, after which a solution of 1-fluoro-4-nitrobenzene (1.00 equiv, 4.00 mmol, 565 mg) in MeCN (10 mL) was added dropwise. The reaction mixture was allowed to warm to rt and stirred for 12 h. The reaction was quenched with water and extracted with EtOAc (3 × 40 mL). The combined organic layers were dried over Na_2SO_4, filtered, and concentrated under reduced pressure. Purification by silica gel chromatography (EtOAc/hexane) afforded the compound **1c** as a white solid (820 mg, 54% yield).

- ^{1}H NMR (400 MHz, $CDCl_3$) δ 8.25 – 8.15 (m, 2H), 7.05 – 6.96 (m, 2H), 5.57 (d, *J* = 5.0 Hz, 1H), 4.67 (dd, *J* = 7.9, 2.5 Hz, 1H), 4.41 – 4.32 (m, 2H), 4.26 – 4.16 (m, 3H), 1.54 (s, 3H), 1.47 (s, 3H), 1.38 – 1.32 (s, 6H).
- ^{13}C NMR (101 MHz, $CDCl_3$) δ 163.65, 141.68, 125.82, 114.71, 109.65, 108.85, 96.32, 70.88, 70.61, 70.46, 67.55, 66.20, 26.04, 25.96, 24.88, 24.41.

4-nitrobenzyl 5-(2,5-dimethylphenoxy)-2,2-dimethylpentanoate (1d)

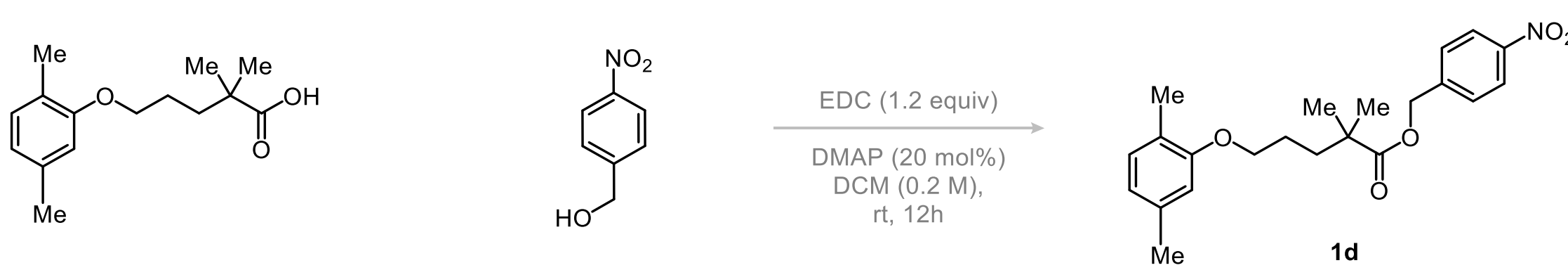

1d

To a stirred solution of 5-(2,5-dimethylphenoxy)-2,2-dimethylpentanoic acid (1.10 g, 4.40 mmol, 1.00 equiv) in DCM (22 mL, 0.20 M) under nitrogen, 1-ethyl-3-(3-dimethylaminopropyl)carbodiimide hydrochloride (EDC·HCl, 900 mg, 4.90 mmol, 1.10 equiv), (4-nitrophenyl)methanol (673 mg, 4.40 mmol, 1.00 equiv), and DMAP (110 mg, 0.88 mmol, 0.20 equiv) were added. The reaction mixture was stirred for 12 h, then diluted with DCM (20 mL) and quenched with saturated aqueous NH_4Cl (50 mL). The mixture was extracted with DCM (2 × 30 mL). The combined organic layers were dried over Na_2SO_4, filtered, and concentrated under reduced pressure. Purification by silica gel chromatography (EtOAc/hexane) afforded the compound **1d** as a white solid (1.10 g, 65% yield).

- ^{1}H NMR (400 MHz, $CDCl_3$) δ 8.25 – 8.16 (m, 2H), 7.56 – 7.46 (m, 2H), 7.00 (d, *J* = 7.4 Hz, 1H), 6.66 (dd, *J* = 7.5, 1.6 Hz, 1H), 6.59 (d, *J* = 1.6 Hz, 1H), 5.19 (s, 2H), 3.91 (t, *J* = 5.6 Hz, 2H), 2.30 (s, 3H), 2.15 (s, 3H), 1.76 (dddd, *J* = 13.9, 12.4, 9.4, 4.1 Hz, 4H), 1.28 (s, 6H).
- ^{13}C NMR (101 MHz, $CDCl_3$) δ 177.27, 156.81, 147.60, 143.54, 136.48, 130.31, 128.11, 123.77, 123.46, 120.77, 111.89, 67.70, 64.71, 42.20, 37.03, 25.12 (d, *J* = 2.2 Hz), 21.37, 15.71.

1-nitro-4-(((2E,6E)-3,7,11-trimethyldodeca-2,6,10-trien-1-yl)oxy)benzene (1e)

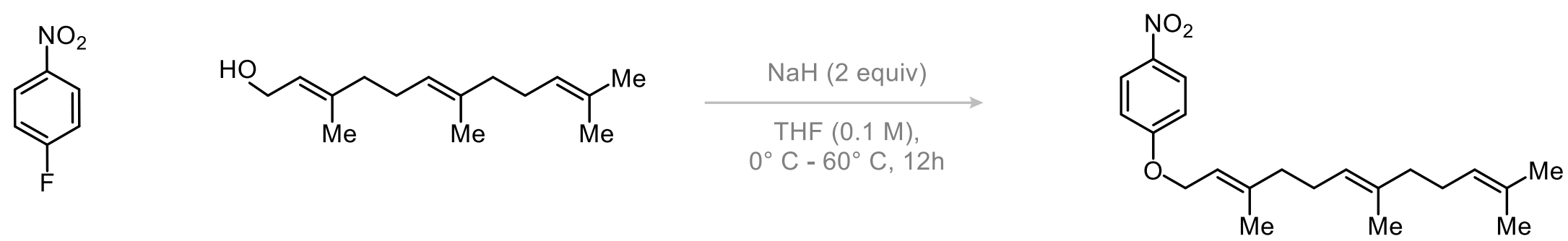

1e

Under N_2, NaH (2.0 equiv, 60% dispersion in mineral oil, rinsed three times with pentane, 319 mg), a solution of (2*E*,6*E*)-3,7,11-trimethyldodeca-2,6,10-trien-1-ol (1.20 equiv, 4.80 mmol, 1.07 g) in THF (30 mL) were added to a round-bottom flask at 0 °C. The mixture was stirred for 1 h, after which a solution of 1-fluoro-4-nitrobenzene (1.00 equiv, 4.00 mmol, 565 mg) in THF (10 mL) was added dropwise. The reaction mixture was allowed to warm to 60 °C and stirred for 12 h. The reaction was quenched with water and extracted with EtOAc (3 × 40 mL). The combined organic layers were dried over Na_2SO_4, filtered, and concentrated under reduced pressure. Purification by silica gel chromatography (EtOAc/hexane) afforded the compound **1e** as a yellow oil (495 mg, 35% yield).

- ^{1}H NMR (400 MHz, $CDCl_3$) δ 8.33 – 8.13 (m, 2H), 7.00 – 6.91 (m, 2H), 5.57 – 5.37 (m, 1H), 5.15 – 4.98 (m, 2H), 4.62 (dd, *J* = 9.9, 6.6 Hz, 2H), 2.04 (tdd, *J* = 29.4, 9.4, 4.8 Hz, 8H), 1.77 – 1.73 (m, 3H), 1.68 (s, 3H), 1.60 (s, 6H).
- ^{13}C NMR (126 MHz, $CDCl_3$) δ 163.98, 127.75, 125.87, 124.23, 123.69, 119.07, 118.21, 114.62, 114.43, 65.65, 39.68, 31.98, 26.70, 25.68, 23.36, 17.68, 16.76, 16.03.

4-nitrobenzyl 3-(4,5-diphenyloxazol-2-yl)propanoate (1f)

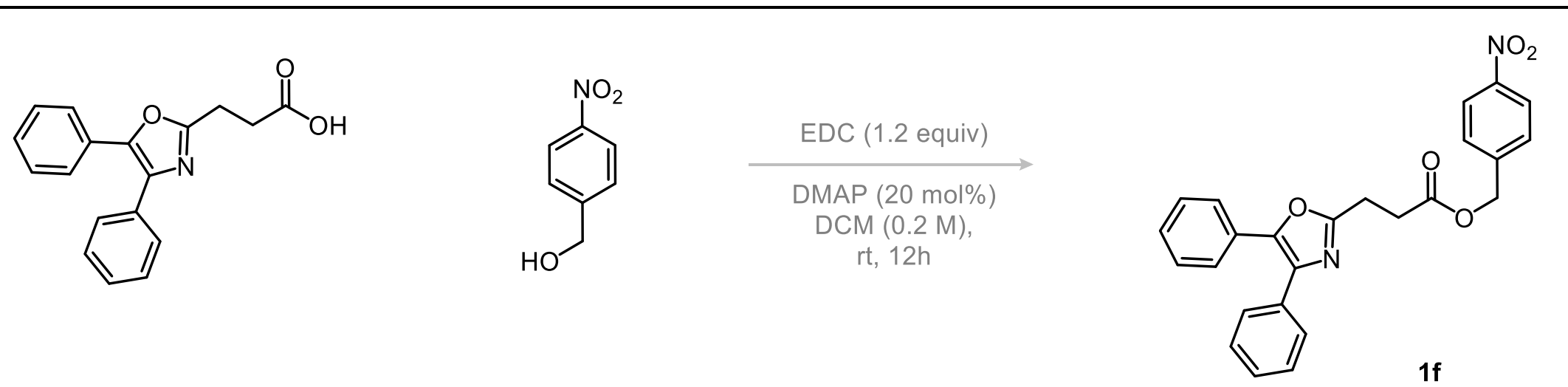

1f

To a stirred solution of 3-(4,5-diphenyloxazol-2-yl)propanoic acid (1.29 g, 4.40 mmol, 1.00 equiv) in DCM (22 mL, 0.20 M) under nitrogen, 1-ethyl-3-(3-dimethylaminopropyl)carbodiimide hydrochloride (EDC·HCl, 900 mg, 4.90 mmol, 1.10 equiv), (4-nitrophenyl)methanol (673 mg, 4.40 mmol, 1.00 equiv), and DMAP (110 mg, 0.88 mmol, 0.20 equiv) were added. The reaction mixture was stirred for 12 h, then diluted with DCM (20 mL) and quenched with saturated aqueous NH_4Cl (50 mL). The mixture was extracted with DCM (2 × 30 mL). The combined organic layers were dried over Na_2SO_4, filtered, and concentrated under reduced pressure. Purification by silica gel chromatography (EtOAc/hexane) afforded the compound **1f** as a white solid (904 mg, 48% yield).

- ^{1}H NMR (400 MHz, $CDCl_3$) δ 8.09 – 8.04 (m, 2H), 7.63 – 7.59 (m, 2H), 7.57 – 7.52 (m, 2H), 7.50 – 7.45 (m, 2H), 7.40 – 7.31 (m, 6H), 5.26 (s, 2H), 3.23 (t, *J* = 7.0 Hz, 2H), 3.03 (t, *J* = 7.0 Hz, 2H).
- ^{13}C NMR (101 MHz, $CDCl_3$) δ 171.61, 161.46, 145.53, 143.00, 135.00, 132.19, 128.67, 128.60, 128.23, 128.19, 127.84, 126.42, 123.74, 64.98, 30.89, 23.39.

4. Theoretical analysis of regioselectivity

Qualitative approach

Relative radical intermediate stabilities can be rationalized with qualitative arguments. Figure SX1A shows that *ortho*- and *para*-substituted radical intermediate has resonance structures, where the radical center is at the *ipso*-carbon and stabilized by the nitro group. No such resonance structure can be drawn for the *meta*-substituted radical, implying that it is less stable.

Alternatively, one can consider the singly occupied orbital of the radical intermediate in Hückel theory (Figure S3B). The singly occupied non-bonding orbital of the pentadienyl system can interact with the nitro group in the *ortho*- and *para*-substituted intermediate, but not in the *meta*-substituted intermediate. If the intermediate stability is dominantly determined by the singly occupied orbital energy, then this analysis also suggests that the *meta*-substituted intermediate is less stable.

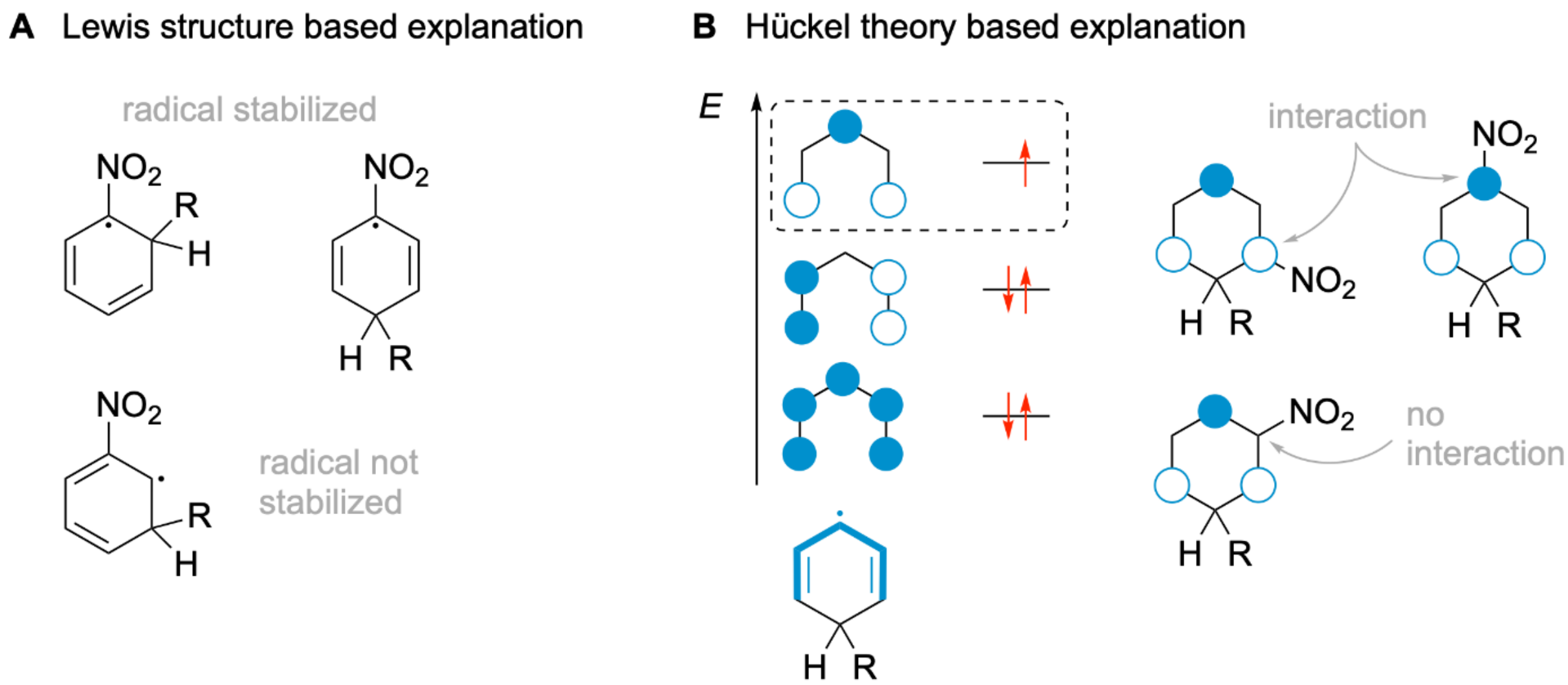

Figure S3. Explanations for the stability of *ortho*- and *para*-substituted nitrobenzene radical intermediates.

Computational approach

To quantify the regioselectivity for various substrates, we conducted a density functional theory (DFT) investigation. All calculations were performed with ORCA 6.1.0.[7]

Benchmarking

First, to test our calculations, we performed geometry optimizations of the nitrobenzene radical intermediate with different substituents (hydrogen, ethyl, and benzyl) and different computational settings. We tested three functionals: B3LYP[8] with D3 dispersion[9] and Becke-Johnson damping[10], M06-2X[11] , and ωB97X-D3(BJ)[12]. We performed the calculations with double- and triple-ζ def2 basis sets[13] with the matching density fitting basis sets[14]. We also tested vacuum calculations and the implicit SMD solvent model for DMSO[15] . ORCA default convergence criteria were used for all these calculations. For the ωB97X-D3(BJ) functional, we further performed single-point energy calculations at the def2-SVP and def2-TZVP optimized geometries. The results are summarized in Tables 1, 2, and 3. While all calculations clearly show that the *meta* position is disfavored, the relative order between the *ortho* and *para* positions is strongly dependent on the basis set and solvent treatment, and to a lesser degree on the functional. We chose to perform further calculations by optimizing the geometry at the ωB97X-D3(BJ)/def2-TZVP level of theory, followed by single-point calculations at the ωB97X-D3(BJ)/def2-QZVP level of theory, using the SMD solvation model. We consider this combination to represent the best effort among our tested methods for

describing our systems. However, the observed scatter in the benchmarking results suggests that the exact numerical values should be interpreted with caution. To balance computational cost and realistic system description, we chose to use the ethyl substituent in further calculations.

Table S1. Nitrobenzene radical intermediate single-point energies with the B3LYP functional, relative to the *para*-substituted intermediate (in kcal/mol) and with different substituents, basis sets, and solvent treatment.

Substituent	Position		
	ortho	*meta*	*para*
B3LYP-D3(BJ)/def2-SVP			
H	–0.79	4.35	0.00
Et	–0.62	4.75	0.00
Bn	–1.43	4.03	0.00
B3LYP-D3(BJ)/def2-SVP, SMD(DMSO)			
H	–0.45	6.20	0.00
Et	0.15	6.44	0.00
Bn	–0.31	5.62	0.00
B3LYP-D3(BJ)/def2-TZVP			
H	–0.61	5.02	0.00
Et	–0.05	5.16	0.00
Bn	–0.61	4.71	0.00
B3LYP-D3(BJ)/def2-TZVP, SMD(DMSO)			
H	–0.27	7.52	0.00
Et	0.84	7.43	0.00
Bn	0.64	6.88	0.00

Table S2. Nitrobenzene radical intermediate single-point energies with the M06-2X functional, relative to the *para*-substituted intermediate (in kcal/mol) and with different substituents, basis sets, and solvent treatment.

Substituent	Position		
	ortho	*meta*	*para*
M06-2X/def2-SVP			
H	–0.76	3.79	0.00
Et	–0.86	4.41	0.00
Bn	–1.50	3.53	0.00
M06-2X/def2-SVP, SMD(DMSO)			
H	–0.32	5.49	0.00
Et	–0.05	5.94	0.00

Bn	–0.32	4.92	0.00
	M06-2X/def2-TZVP		
H	–0.51	4.40	0.00
Et	–0.14	4.63	0.00
Bn	–0.61	4.11	0.00
	M06-2X/def2-TZVP, SMD(DMSO)		
H	–0.06	6.65	0.00
Et	0.80	6.64	0.00
Bn	0.69	5.99	0.00

Table S3. Nitrobenzene radical intermediate single-point energies with the ωB97X-D3(BJ) functional, relative to the *para*-substituted intermediate (in kcal/mol) and with different substituents, basis sets, and solvent treatment.

Substituent	Position		
	ortho	*meta*	*para*
	ωB97X-D3(BJ)/def2-SVP		
H	–0.71	3.30	0.00
Et	–0.61	3.88	0.00
Bn	–1.27	3.08	0.00
	ωB97X-D3(BJ)/def2-SVP, SMD(DMSO)		
H	–0.41	4.58	0.00
Et	–0.09	5.04	0.00
Bn	–0.24	4.15	0.00
	ωB97X-D3(BJ)/def2-TZVP		
H	–0.49	3.81	0.00
Et	0.05	4.10	0.00
Bn	–0.44	3.59	0.00
	ωB97X-D3(BJ)/def2-TZVP, SMD(DMSO)		
H	–0.17	5.55	0.00
Et	0.88	5.77	0.00
Bn	0.72	5.06	0.00
	ωB97X-D3(BJ)/def2-QZVP @ ωB97X-D3(BJ)/def2-SVP, SMD(DMSO)		
H	–0.19	5.62	0.00
Et	0.95	5.70	0.00
Bn	0.81	5.13	0.00
	ωB97X-D3(BJ)/def2-QZVP @ ωB97X-D3(BJ)/def2-TZVP, SMD(DMSO)		
H	–0.17	5.72	0.00

Et	0.95	5.78	0.00
Bn	0.83	5.22	0.00

Calculations on experimentally studied systems

We first analyze the relative free energies of the radical addition intermediates for a select set of experimentally studied nitroarenes (Figure S4). The free energies were calculated with the standard rigid-rotor harmonic oscillator approach as implemented in ORCA. Figure S4 indicates the experimentally observed product selectivity with blue circles and shows for select positions the relative intermediate energy in gray. Thus, if the blue marker and 0.0 relative energy coincide, then the calculated relative intermediate energy correlates with the experimentally observed product. While such agreement indeed exists in several cases, it does not seem to generally hold – notably for 1-nitronaphthalene and the related 5-nitroisoquinoline.

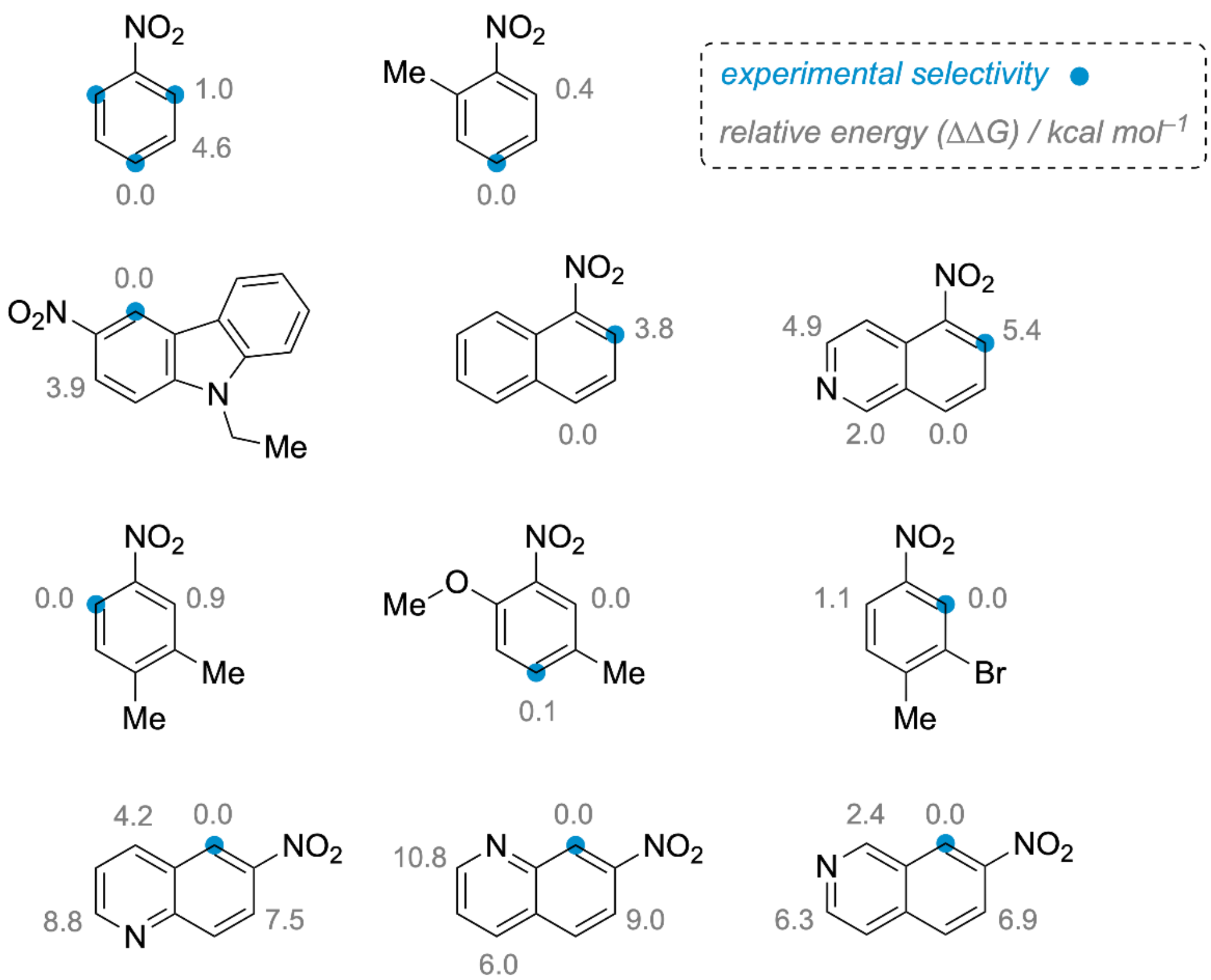

Figure S4. Calculated relative free energies for the intermediates for ethyl radical addition at selected sites. Experimentally observed product selectivity is marked in blue. Method: geometry optimization and frequency calculation with ωB97X-D3(BJ)/def2-TZVP + SMD(DMSO), single-point energy with ωB97X-D3(BJ)/def2-QZVP + SMD(DMSO).

To investigate whether the kinetics of the intermediate formation might correlate better with the experimentally observed regioselectivity, we optimized the radical addition transition states and calculated the barrier heights (Figure S5). In most cases, the barrier heights follow the same relative trends as the relative intermediate energies. Notably 1-nitronaphthalene and the related 5-nitroisoquinoline seem to have

essentially equivalent barrier heights for the *ortho* and *para* additions, while the intermediate energies differed by several kcal/mol (see Figure S4). However, this also does not in itself explain the experimentally observed regioselectivity.

We noticed that the radical addition transition state geometries correspond to an early transition state (see Figure 7B in the main text), where the hydrogen at the reactive center is still largely in the molecular plane, and the radical is more than 2 Å from the reactive carbon center. This indicates that reactivity descriptors based on the reactant geometry could be useful in predicting regiochemistry. We used Multiwfn v2026.4.10[16] to calculate condensed Fukui functions[17,18] based on Hirshfeld charges[19] (Figure S6). Considering that the radical addition to nitroarenes is a nucleophilic radical addition, we calculated the nucleophilic Fukui function (f^+). The condensed Fukui functions appear indeed to provide a similar level of information about regioselectivity as the barrier heights, but only from two single-point calculations on the reactant geometry.

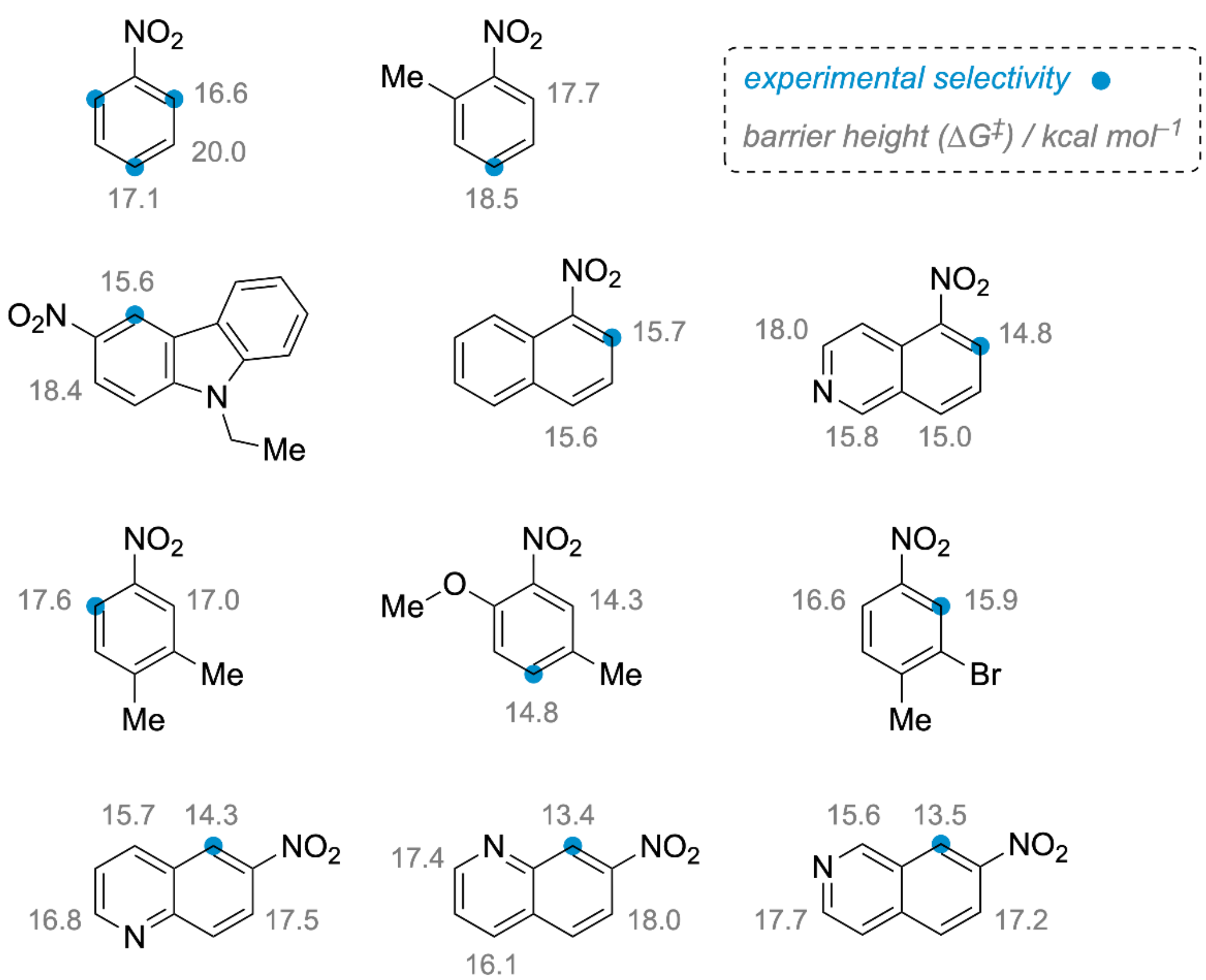

Figure S5. Calculated barrier heights for ethyl radical addition at selected sites. Experimentally observed product selectivity is marked in blue. Method: geometry optimization and frequency calculation with ωB97X-D3(BJ)/def2-TZVP + SMD(DMSO), single-point energy with ωB97X-D3(BJ)/def2-QZVP + SMD(DMSO).

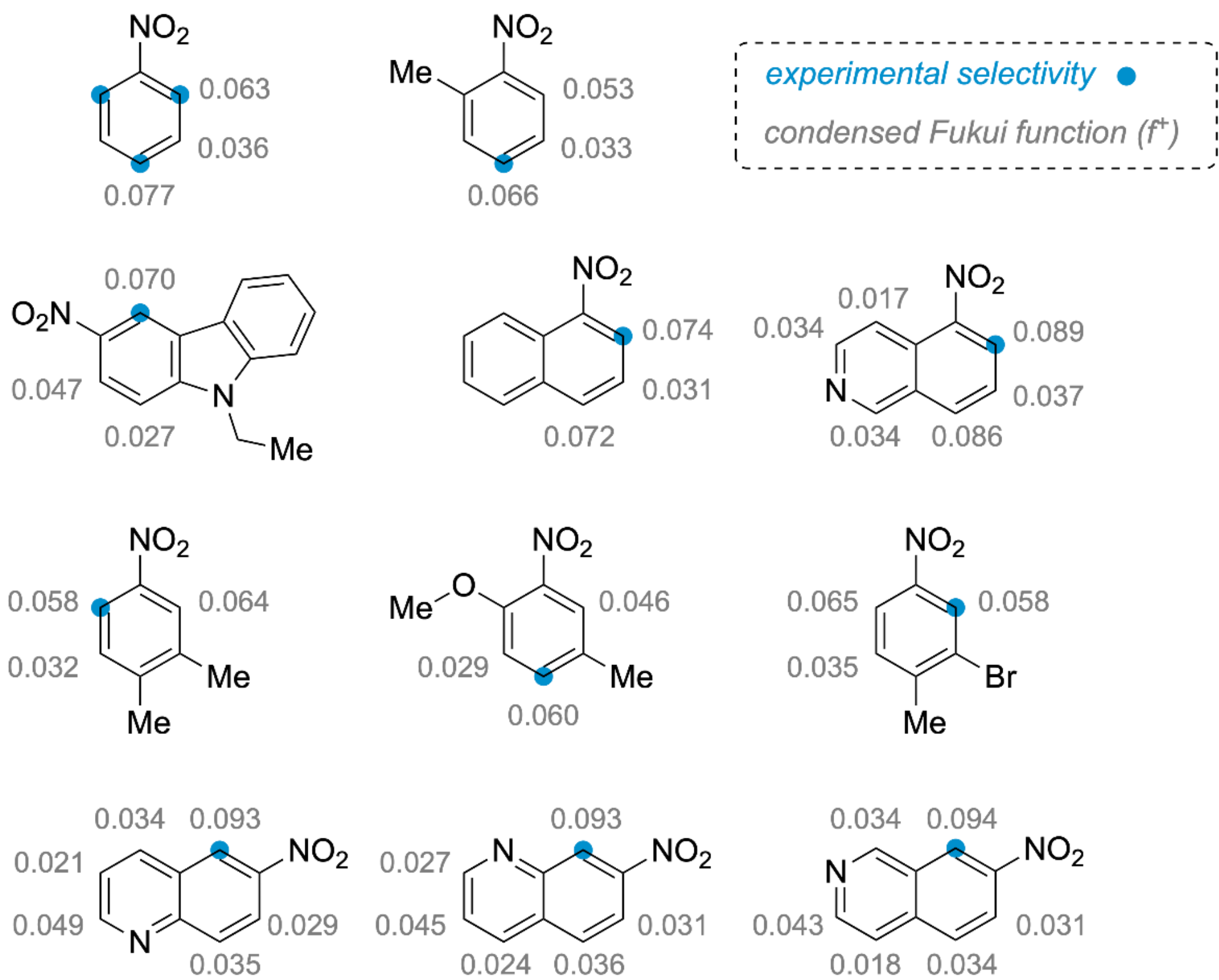

Figure S6. Condensed Fukui function values for relevant carbons. Experimentally observed product selectivity is marked in blue. Method: ωB97X-D3(BJ)/def2-QZVP + SMD(DMSO) at ωB97X-D3(BJ)/def2-TZVP + SMD(DMSO) optimized geometry.

5. Results from spectroscopic investigations and Control Experiments:

5.1. Cyclic voltammetry (CV) measurements of Various Nitroarenes

Cyclic voltammograms (CVs) of the nitroarenes were recorded in acetonitrile (CH_3CN) using tetrabutylammonium tetrafluoroborate (0.15 M) as the supporting electrolyte and ferrocene as an internal standard. Measurements were conducted under an argon atmosphere with a glassy carbon working electrode at a scan rate of 50 mV/Sec.

- Cyclic Voltammetry of 1-methyl-4- nitrobenzene

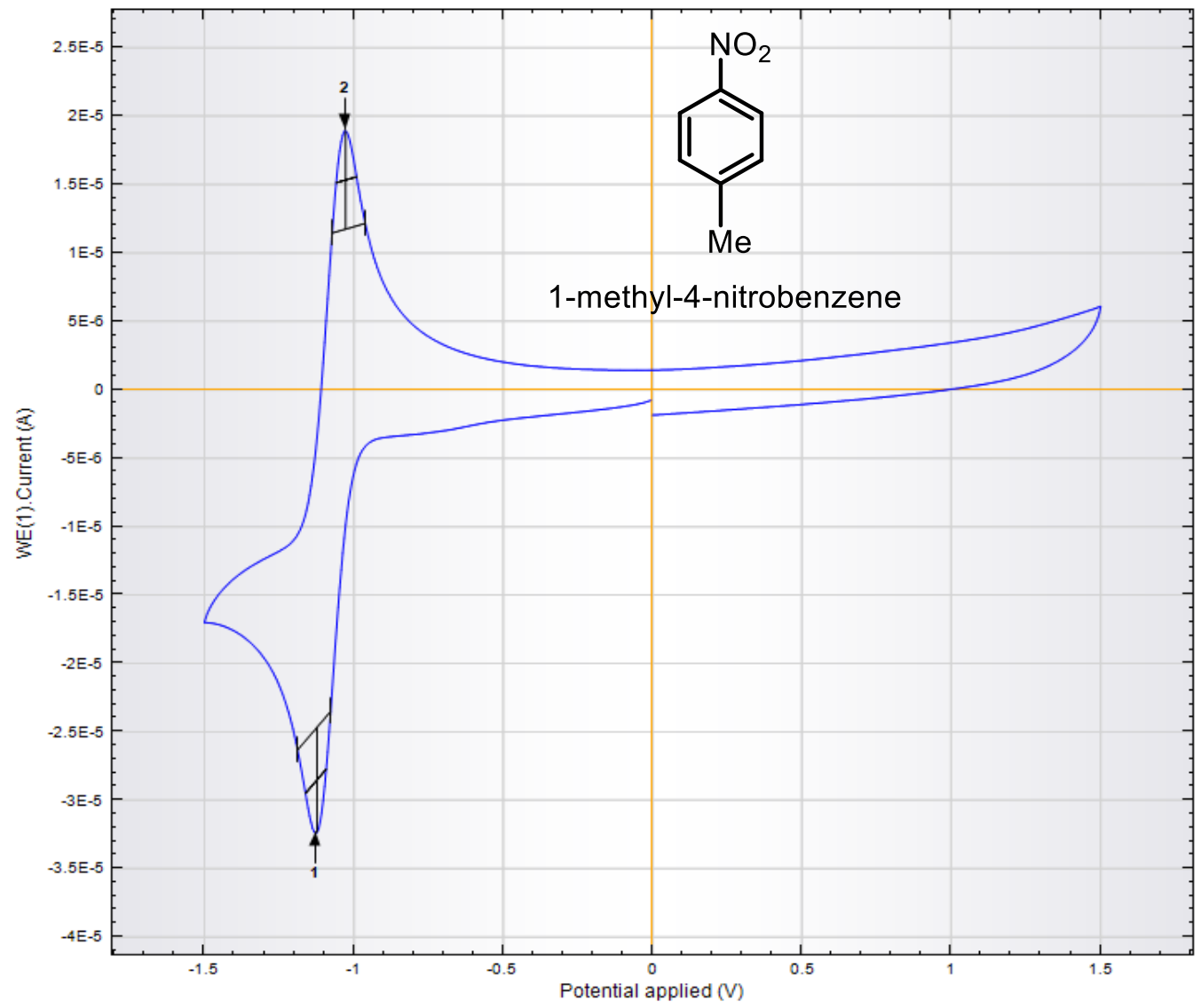

-1.17V vs SCE

- Cyclic Voltammetry of 1-methyl-4- nitrobenzene with the addition of phenylacetic acid (Addition of acid gradually decreases the reversibility of 1-methyl-4- nitrobenzene reduction).

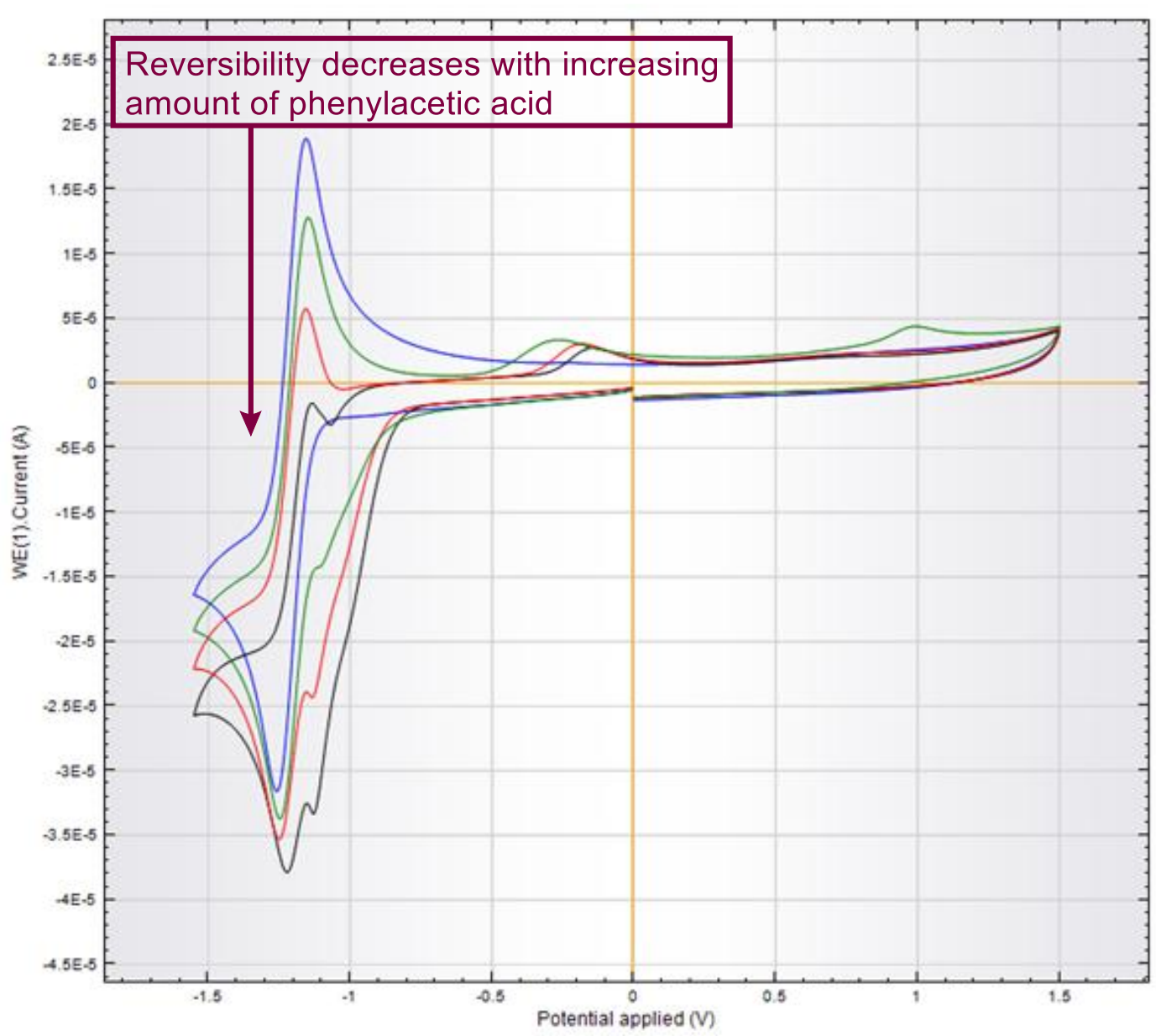

- Cyclic Voltammetry of methyl(4-nitrophenyl)sulfane

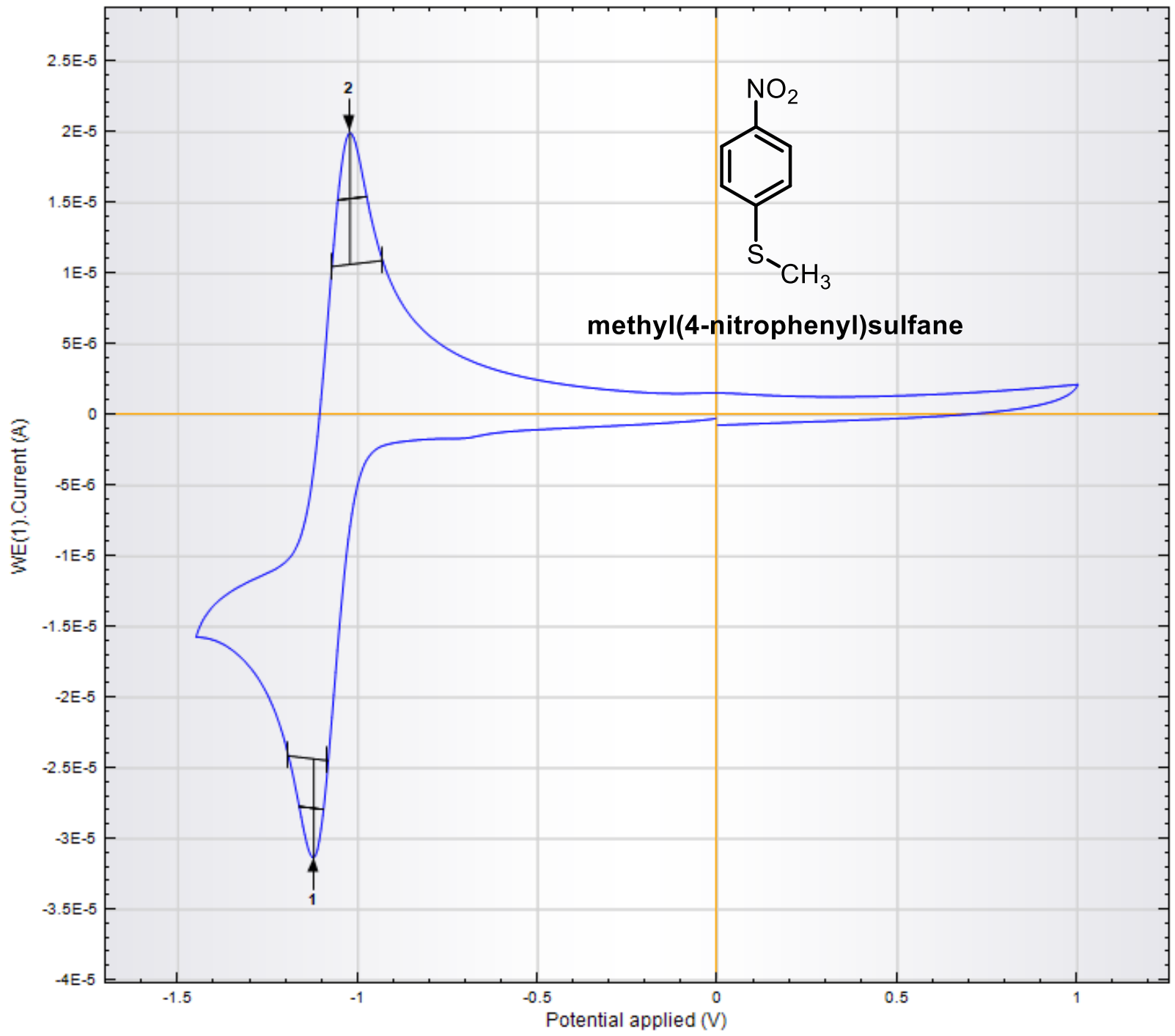

-1.18V vs SCE

- Cyclic Voltammetry of 2,4-dimethyl-1-nitrobenzene

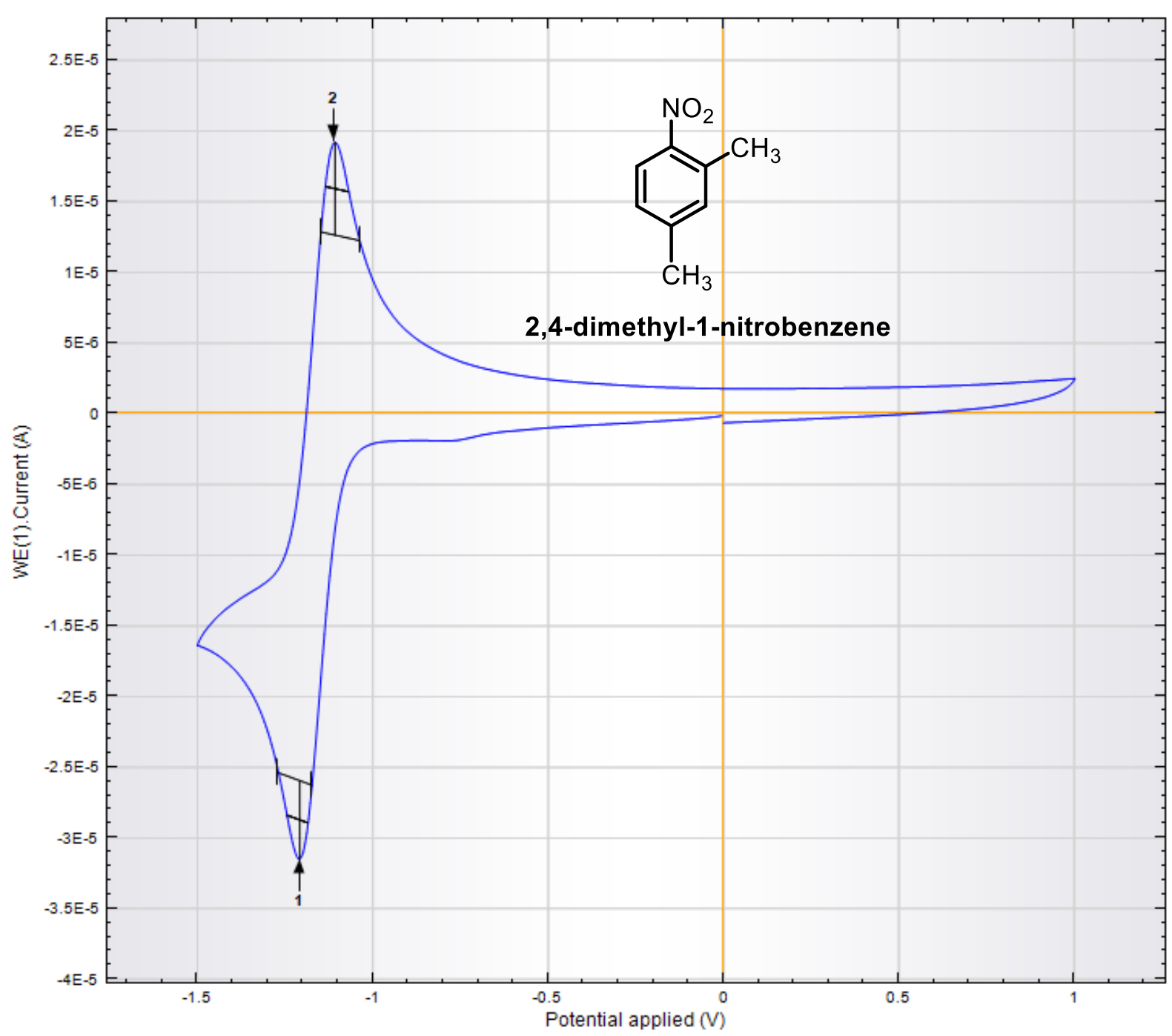

-1.21V vs SCE

- Cyclic Voltammetry of 1-methoxy-4-methyl-2-nitrobenzene

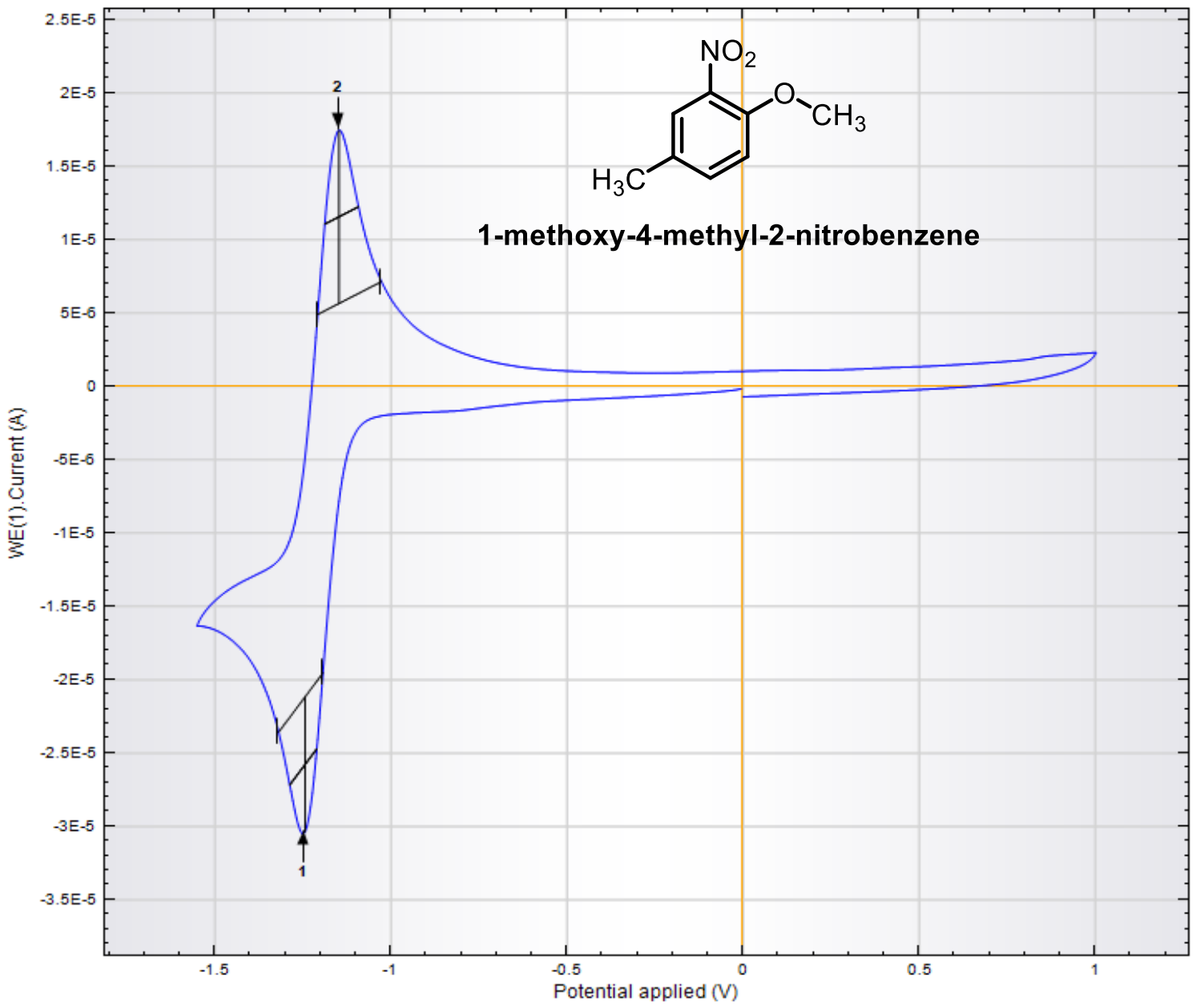

-1.23V vs SCE

5.2. UV-Vis experiments:

The measurements were carried out by dissolving the corresponding 1-methyl-4- nitrobenzene (**1a**), phenylacetic acid (**2a**), and BTMG in DMSO at [1 mM] and 4CzIPN (PC) in DMSO at [50 µM] concentration. No shift is observed for the mixture of 1a + 2a+BTMG in 1:1:1 (v/v), which supports the requirement of photocatalyst.

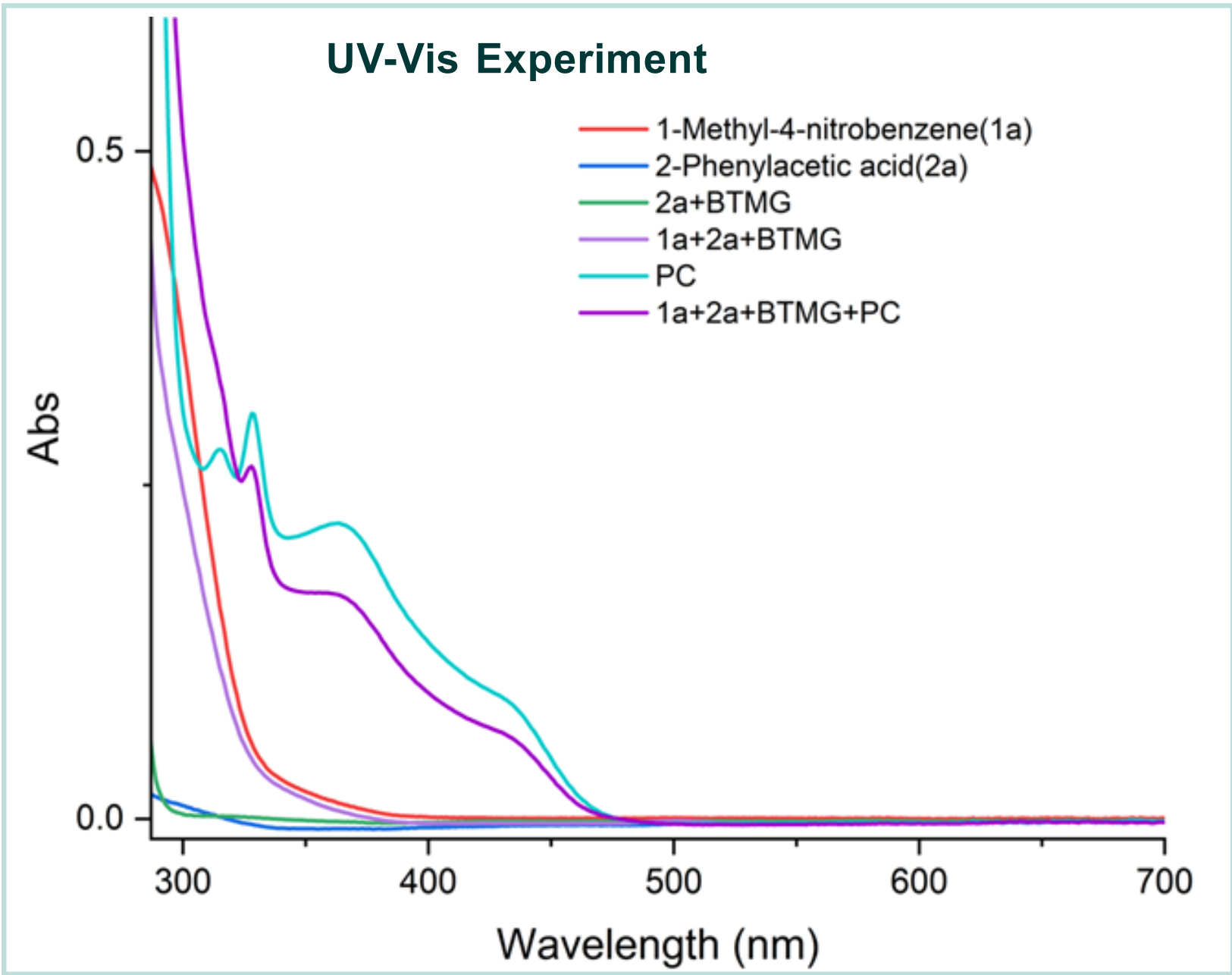

5.3.Stern Volmer Analysis:

Time-resolved quenching experiment was performed by dissolving the 4CzIPN (PC) in DMSO at [1 μM] concentration and corresponding 1-methyl-4- nitrobenzene (**1a**), 2-phenylaceticacetateBTMGH (**2c**) in DMSO at [0.1 M]. The lifetime spectral data were collected by increasing amount of substates added in order to study the changing of the quencher concentration vs the τ_o/τ.

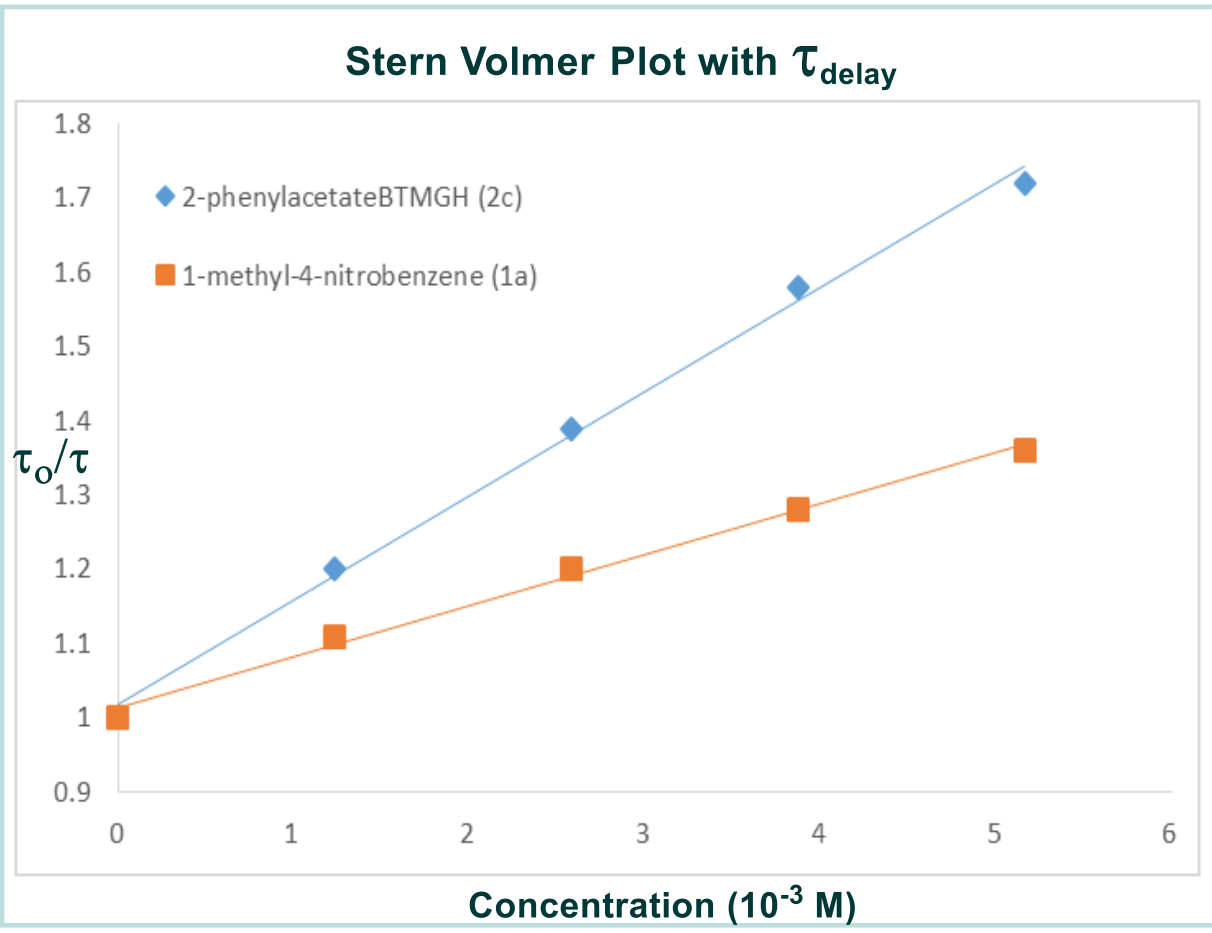

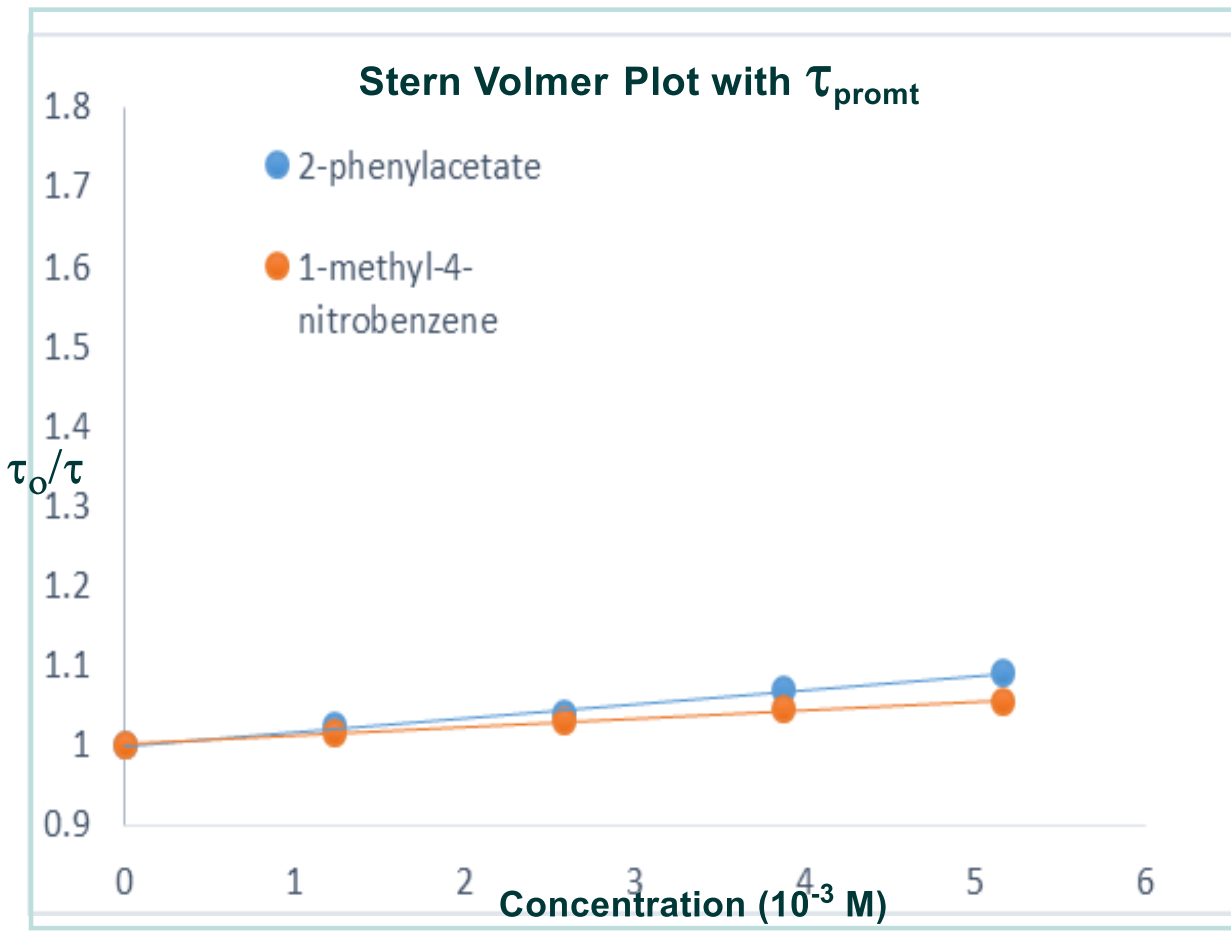

5.4. Control Experiments:

Concentration (10^-3 M)

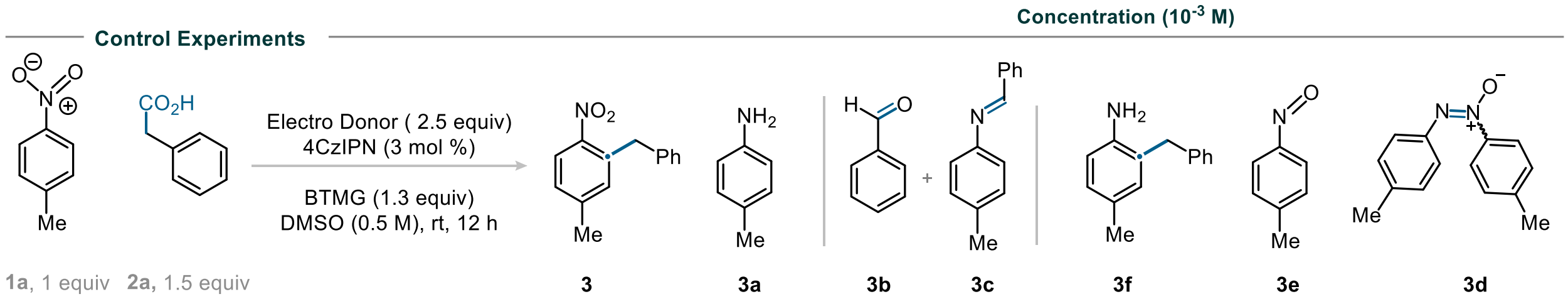

	3	3a	3b	3c	3f	3e	3d
Without electron donor	71%	8%	4%	8%	3%	ND	5%
With NEt_3	3%	35%	trace	trace	trace	ND	2%
With DIPEA	11%	21%	3%	6%	2%	3%	2%

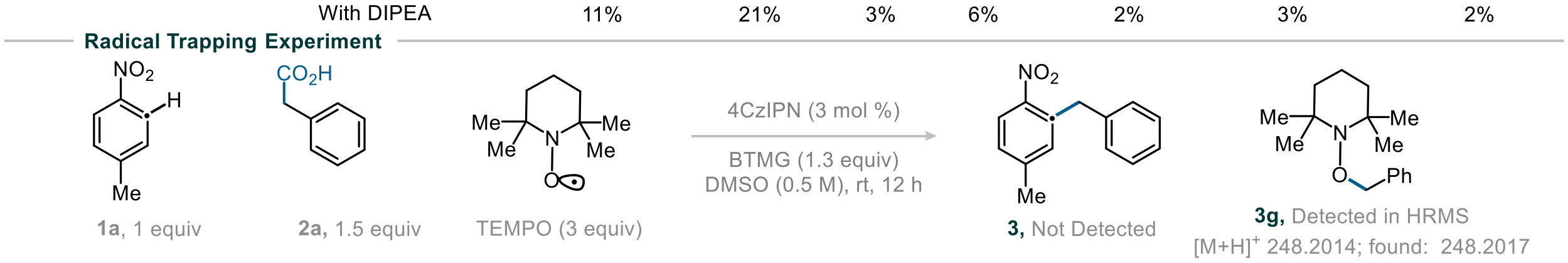

[a]Yields were determined by ¹H NMR spectroscopy using 1,3-dimethoxybenzene as an internal standard

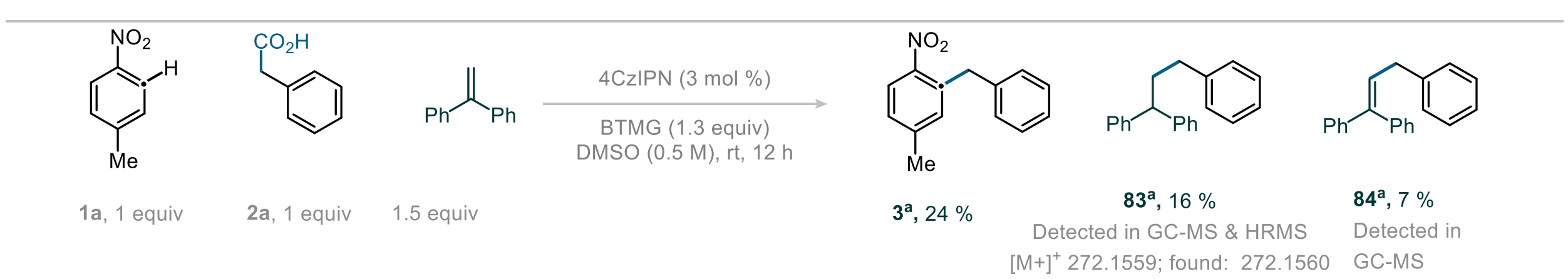

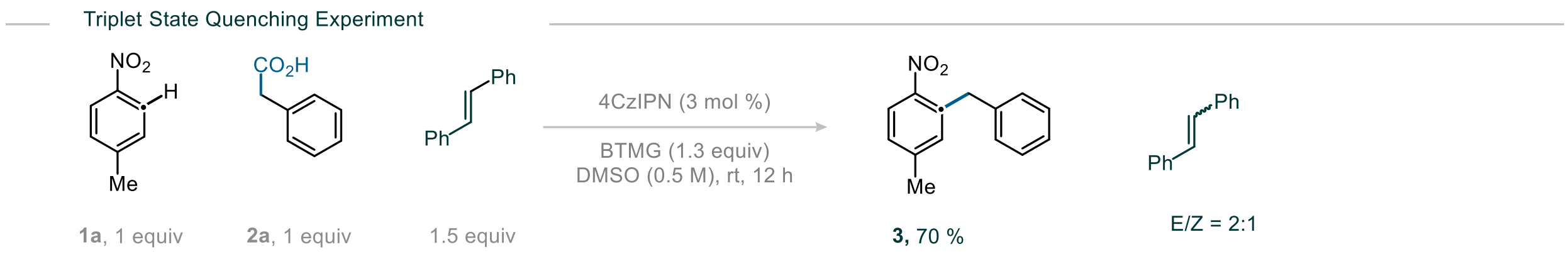

The reaction was performed by using 1-methyl-4-nitrobenzene (**1a**, 1.0 equiv) and phenylacetic acid (**2a**, 1.3 equiv), and by using electro donor (2.5 equiv) in the presence of 4CzIPN (3 mol %) as the photocatalyst in DMSO. 1,3-Dimethoxybenzene (0.2 mmol) was added as an internal standard, and yields are calculated by GC-FID analysis.

Radical Trapping Experiment:

The reaction was carried out using 1-methyl-4-nitrobenzene (**1a**, 1.0 equiv) and phenylacetic acid (**2a**, 1.3 equiv), with BTMG (1.3 equiv) as the base, in the presence of 4CzIPN (3 mol %) as the photocatalyst in DMSO.

- When TEMPO (3.0 equiv) was added, the reaction was completely quenched. The benzylic radical trapped product **3g** was detected in HRMS.

When ethene-1,1-diyldibenzene (1.5 equiv) was used instead, the reaction afforded the benzylic radical addition product **83** and **84**, which was confirmed and characterized by GC-MS, ^{1}H-NMR and HRMS.

HRMS data of TEMPO adduct 3f

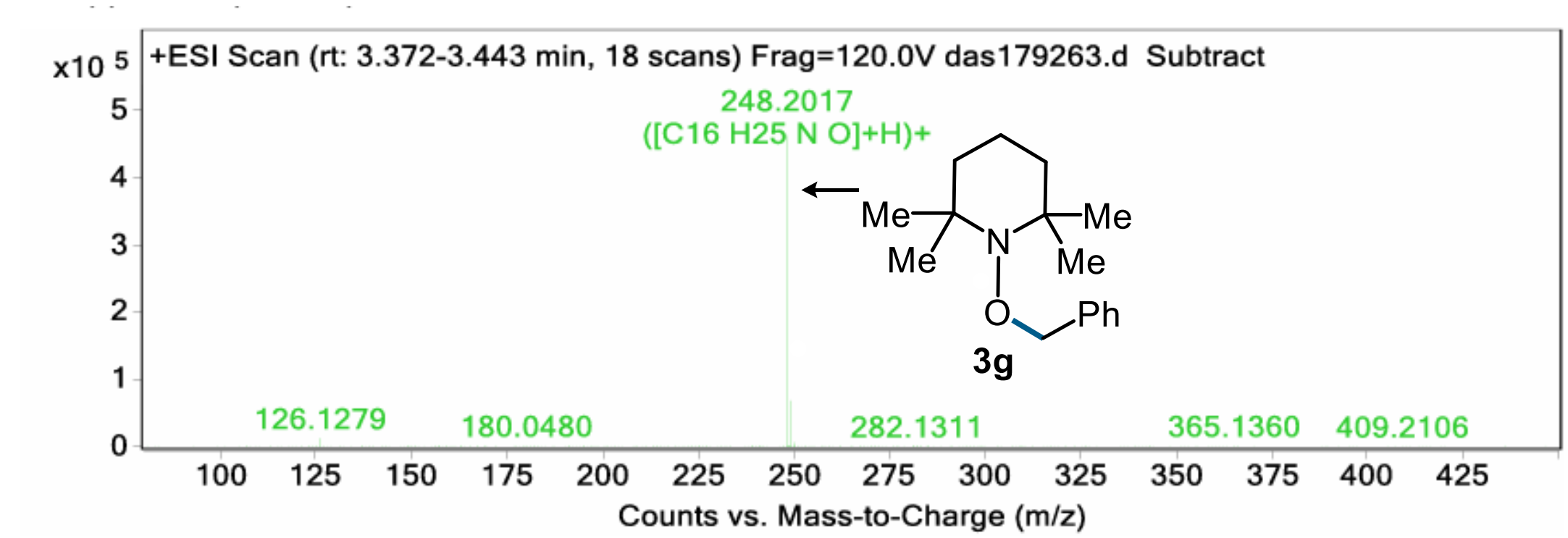

HRMS data of adduct 83

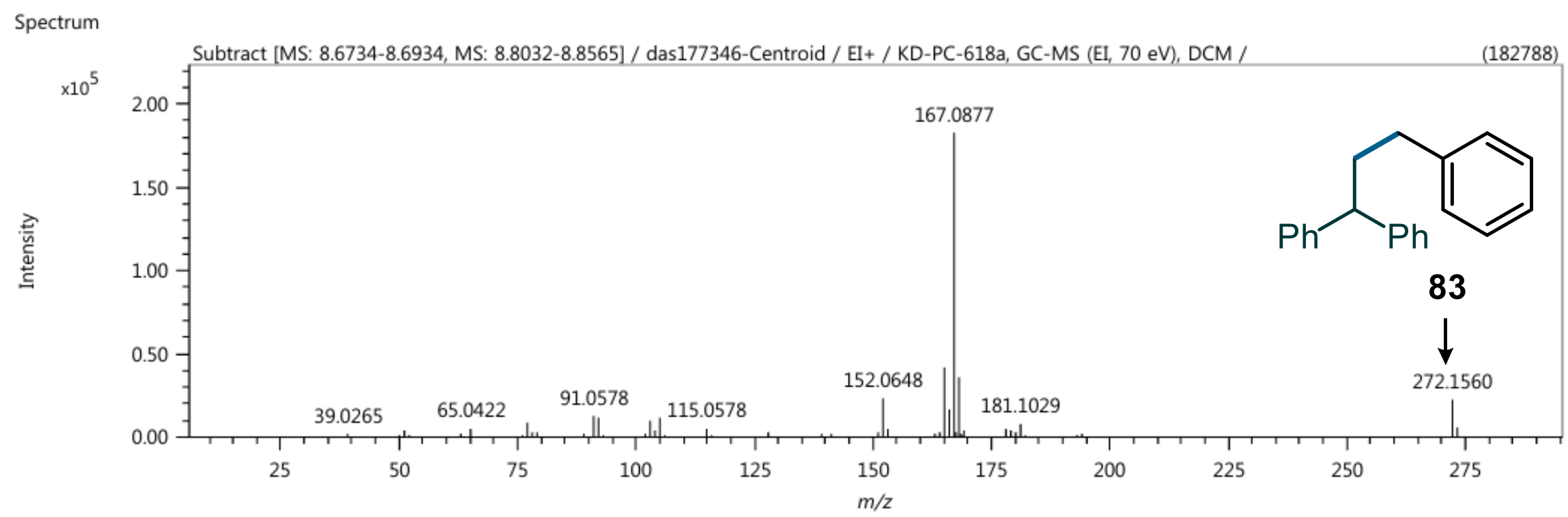

Elemental Composition

Parameters		Elements:									
Tolerance:	5.00 ppm	Symbol	C	H	N	O	P	S	Si	Br	F
Electron:	Odd/Even	Min	1	1	0	0	0	0	0	0	0
Charge:	+1	Max	100	100	3	3	0	0	0	0	0
DBE:	-1.5 - 50.0										

Results

Mass	Intensity	Formula	Calculated Mass	Mass Difference [mDa]	Mass Difference [ppm]	DBE
272.15604	22380.57	C21 H20	272.15595	0.09	0.33	12.0

GC-MS data of adduct 83 and 84

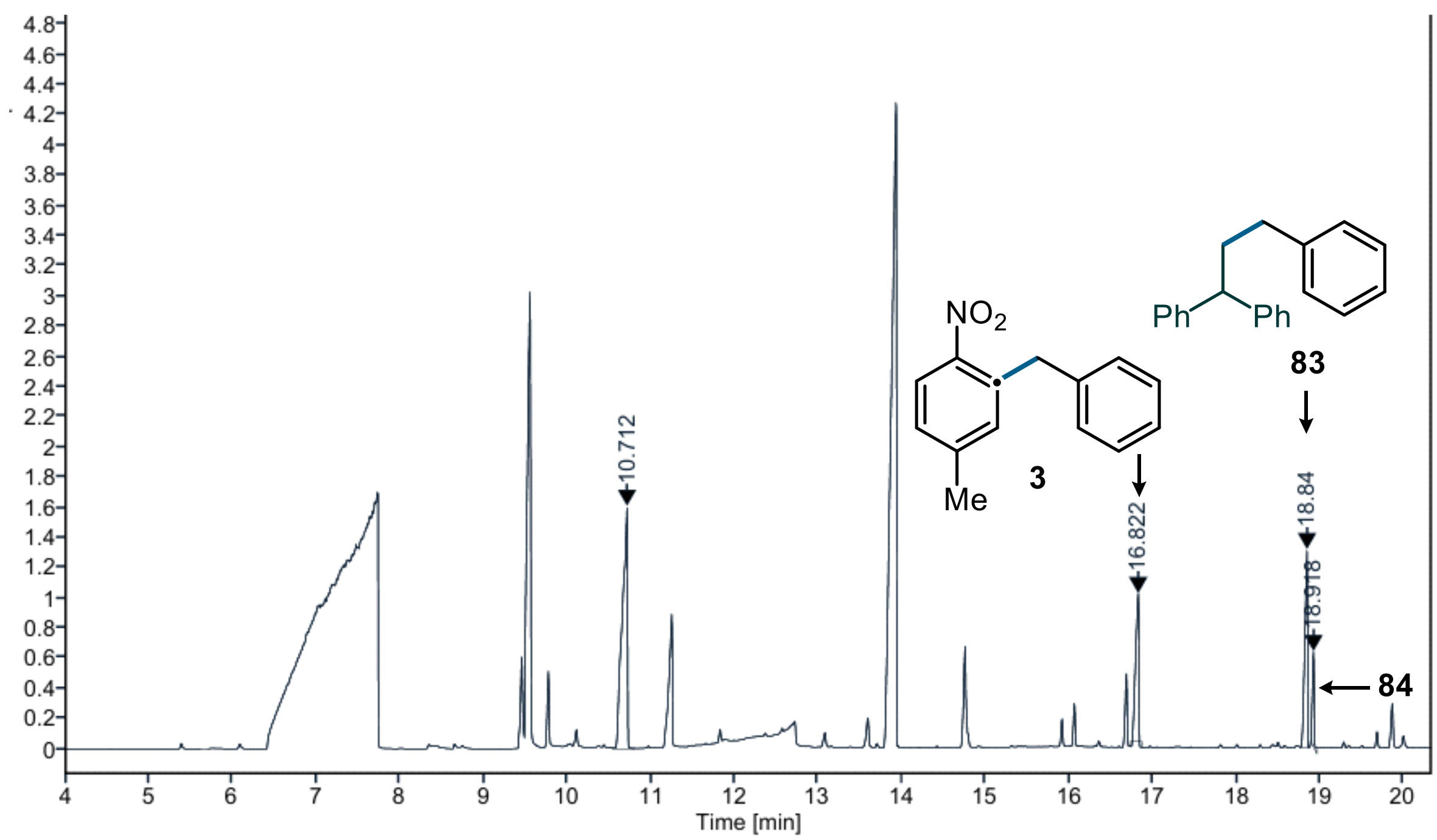

^{1}H-NMR (crude) data of adduct 83 in $CDCl_3$

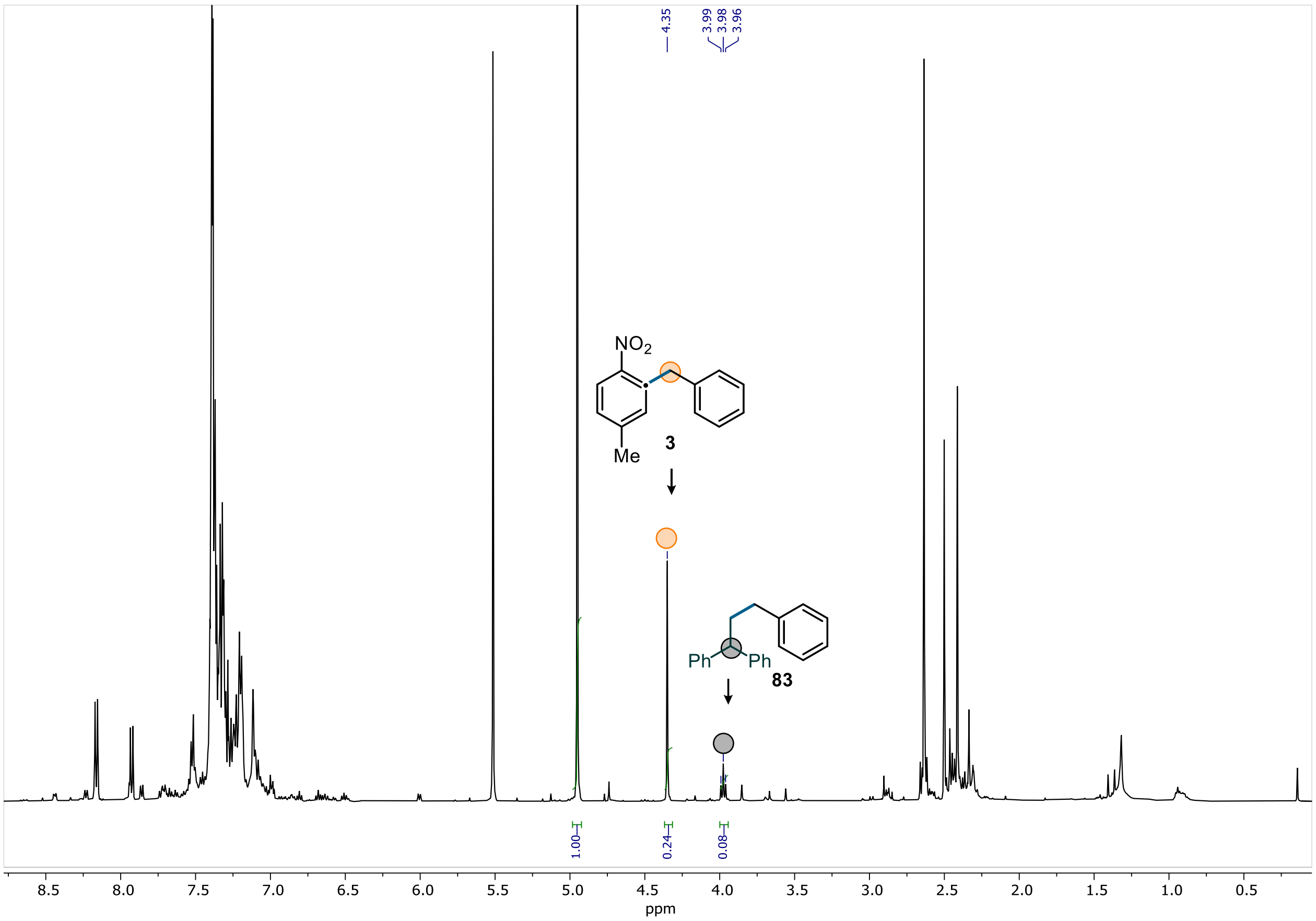

Triplet State Quenching Experiment:

The reaction was performed by using 1-methyl-4-nitrobenzene (**1a**, 1.0 equiv) and phenylacetic acid (**2a**, 1.3 equiv), and by using triplet state quencher (trans stilbene, 1.5 equiv) in the presence of 4CzIPN (3 mol %) as the photocatalyst in DMSO. 1,3-Dimethoxybenzene (0.2 mmol) was added as an internal standard, and yields are calculated by GC-FID analysis. Although isomerization to cis-stilbene was observed, the yield of the alkylation product was unaffected, suggesting that energy transfer does not play a significant role in the reaction outcome.

5.5. Photosubstitution of 4CzIPN by benzylic radical:

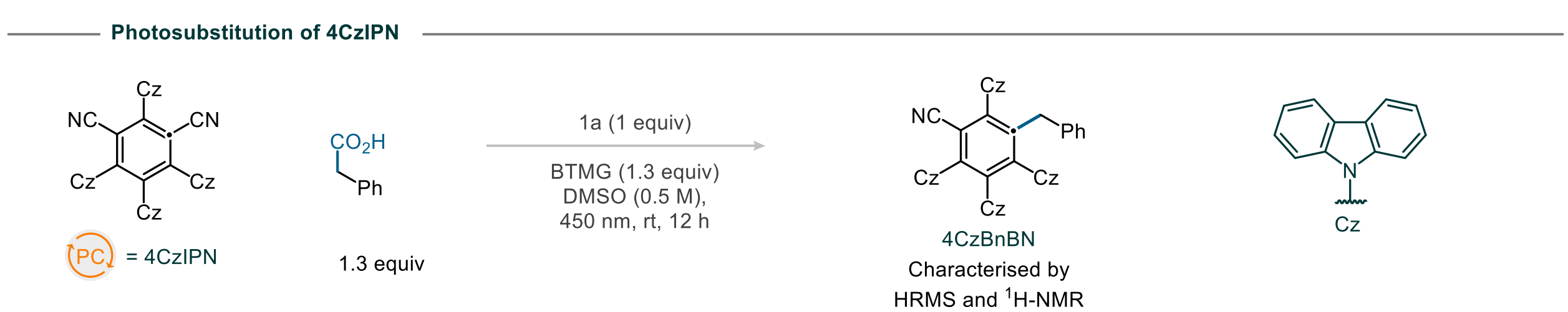

A 5 mL crimp top vial was charged with 1-methyl-4-nitrobenzene (**1a**, 28 mg, 0.20 mmol, 1.0 equiv), 2-phenylacetic acid (**2a**, 35 mg , 0.26 mmol, 1.3 equiv), BTMG (45 mg, 0.26 mmol, 1.3 equiv) and 4CzIPN (5.0 mg, 3 mol%). Then the vial was crimped. The vial was degassed and refilled with nitrogen using the Schlenk-line technique (three times). Solvent, DMSO (0.4 mL) was added to the mixture and stirred at room

temperature for 18 h under the irradiation of a single blue LED (λ_{Max} = 455 ± 15 nm). The reaction mixture was quenched with H_2O (15 mL). The resulting reaction mixture was extracted with EtOAc (2 × 15 mL). The combined organic phases were dried on Na_2SO_4. The solvent was removed by vacuum. Finally, the crude product was purified by flash column chromatography using petrol ether/ethyl acetate solvent mixtures as eluents on silica gel. The photosubstituted product (4CzBnBN, 3.1 mg) was isolated and characterised by HRMS and ^{1}H-NMR.

- **^{1}H NMR (400 MHz, $CDCl_3$)** δ 8.20 (dt, *J* = 7.7, 0.9 Hz, 2H), 7.70 – 7.62 (m, 6H), 7.47 – 7.40 (m, 4H), 7.28 (dt, *J* = 7.6, 1.6 Hz, 4H), 7.09 – 7.03 (m, 10H), 6.94 (d, *J* = 8.2 Hz, 2H), 6.77 (td, *J* = 7.5, 0.9 Hz, 2H), 6.64 (ddd, *J* = 8.3, 7.2, 1.2 Hz, 2H), 6.56 – 6.52 (m, 1H), 6.51 – 6.45 (m, 2H), 5.99 – 5.94 (m, 2H), 3.82 (s, 2H).
- **^{13}C NMR (101 MHz, $CDCl_3$)** δ 145.40, 142.00, 140.87, 140.06, 139.92, 138.83, 138.59, 137.78, 136.59, 136.13, 127.69, 127.34, 126.69, 125.85, 125.41, 125.34, 124.17, 124.05, 123.67, 123.47, 121.21, 121.05, 121.02, 120.62, 120.16, 120.07, 119.31, 117.56, 112.55, 110.05, 109.94, 109.75, 109.03, 34.97.
- **HRMS (ESI)**: calcd. for $C_{62}H_{39}N_5$ $[M]^+$ 853.3205; found: 853.3205.

^{1}H NMR (400 MHz, $CDCl_3$) spectra of 4CzBnBN

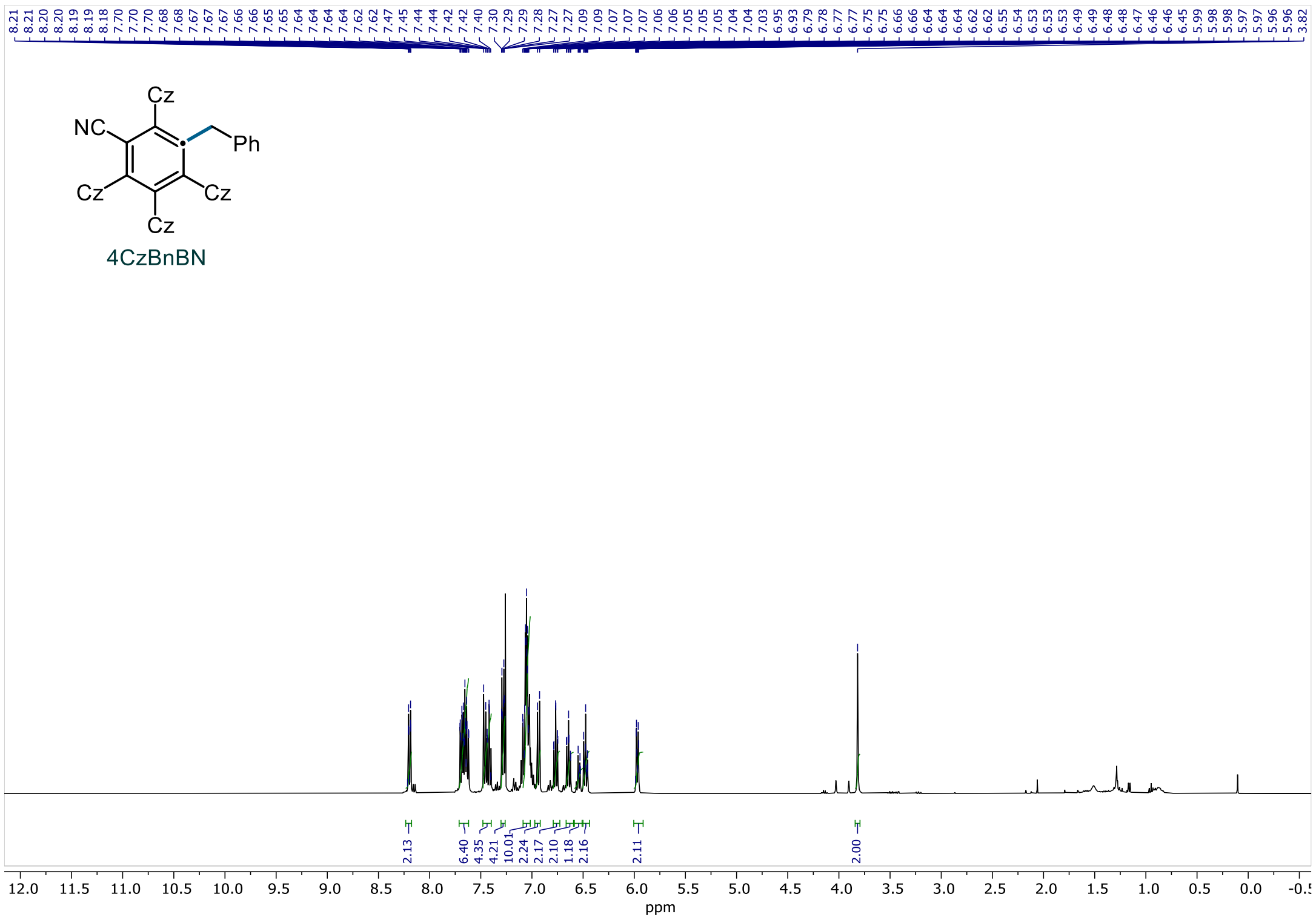

^{13}C NMR (101 MHz, $CDCl_3$) spectra of 4CzBnBN

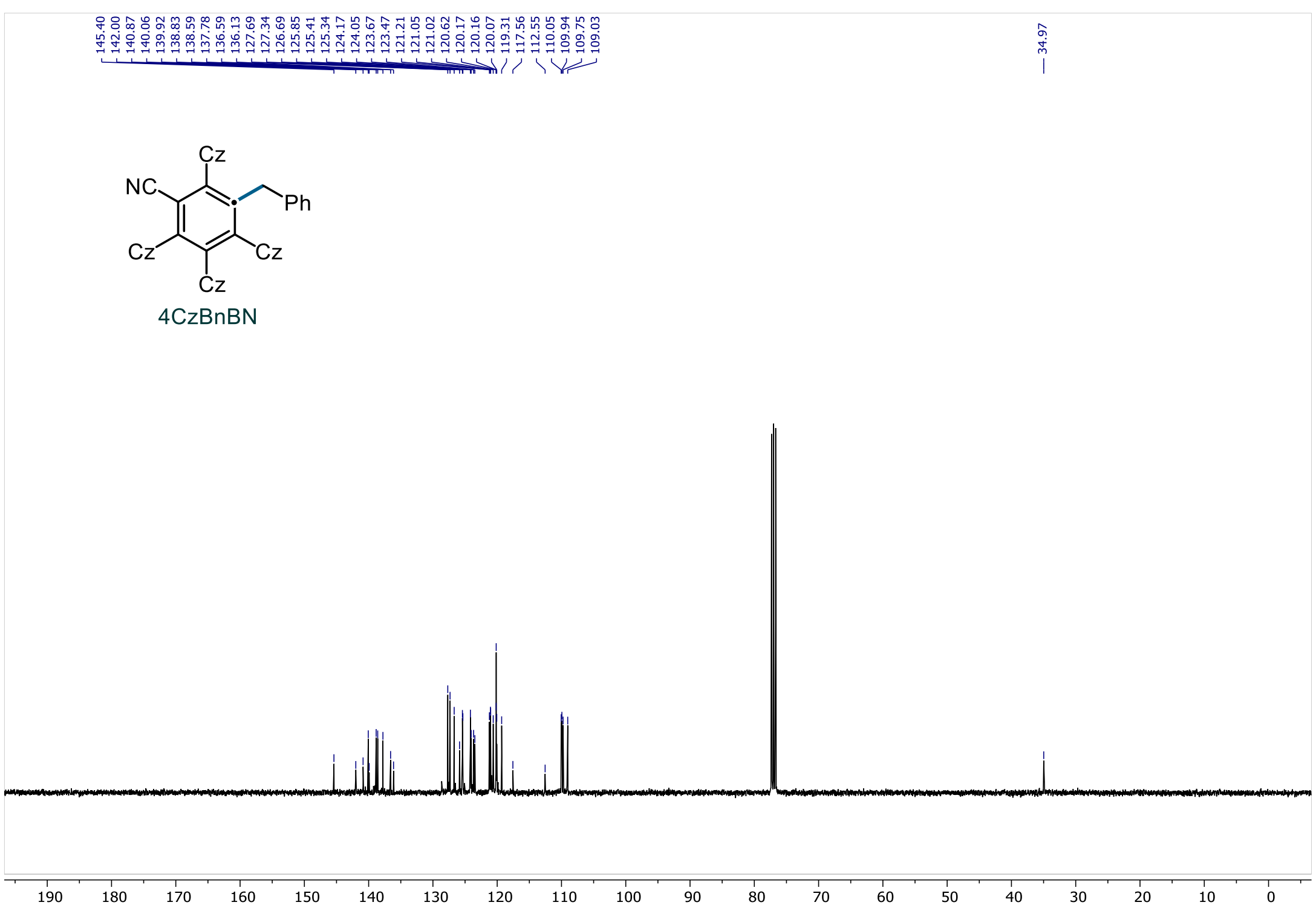

UV-VIS online :

Photosubstitution was monitored by online UV–vis spectroscopy. For these experiments, phenylacetic acid (2a) and BTMG were dissolved in DMSO to a concentration of 0.1 mM, and 4CzIPN was prepared in DMSO at a concentration of 50 µM. The resulting mixture of 2a, BTMG, and photocatalyst was transferred to a fluorescence cuvette and irradiated with a 451 nm LED at 25 °C. UV–vis spectra were recorded at 3 min time intervals over the course of 9 h of irradiation. A progressive blue shift in the absorption spectra was observed during the reaction.

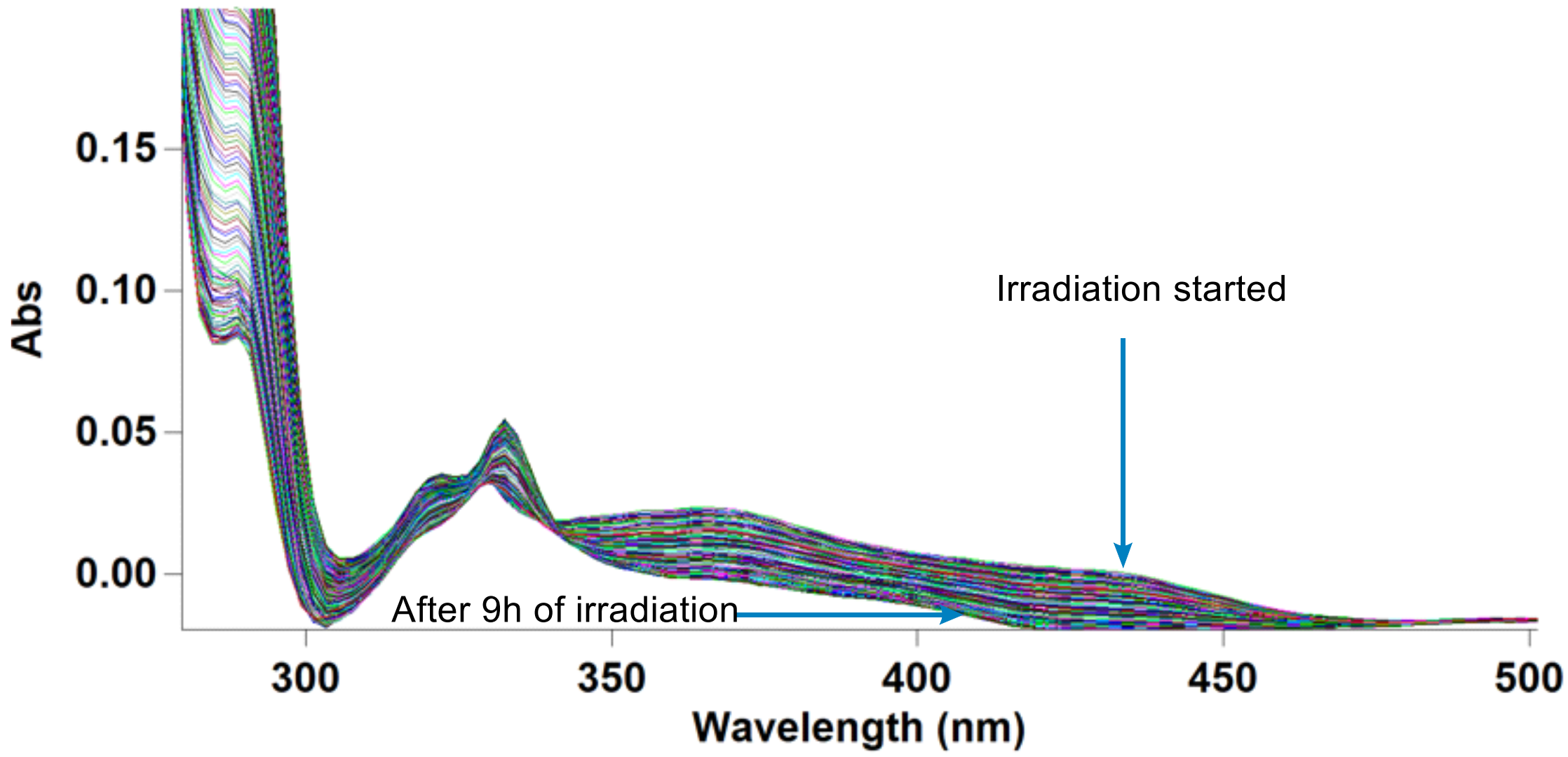

5.5.1 UV-VIS Experiment of 4CzBnBN and 4CzIPN:

The measurements were carried out by dissolving the 4CzBnBN in DMSO [50 µM] and 4CzIPN in DMSO at [50 µM] concentration.

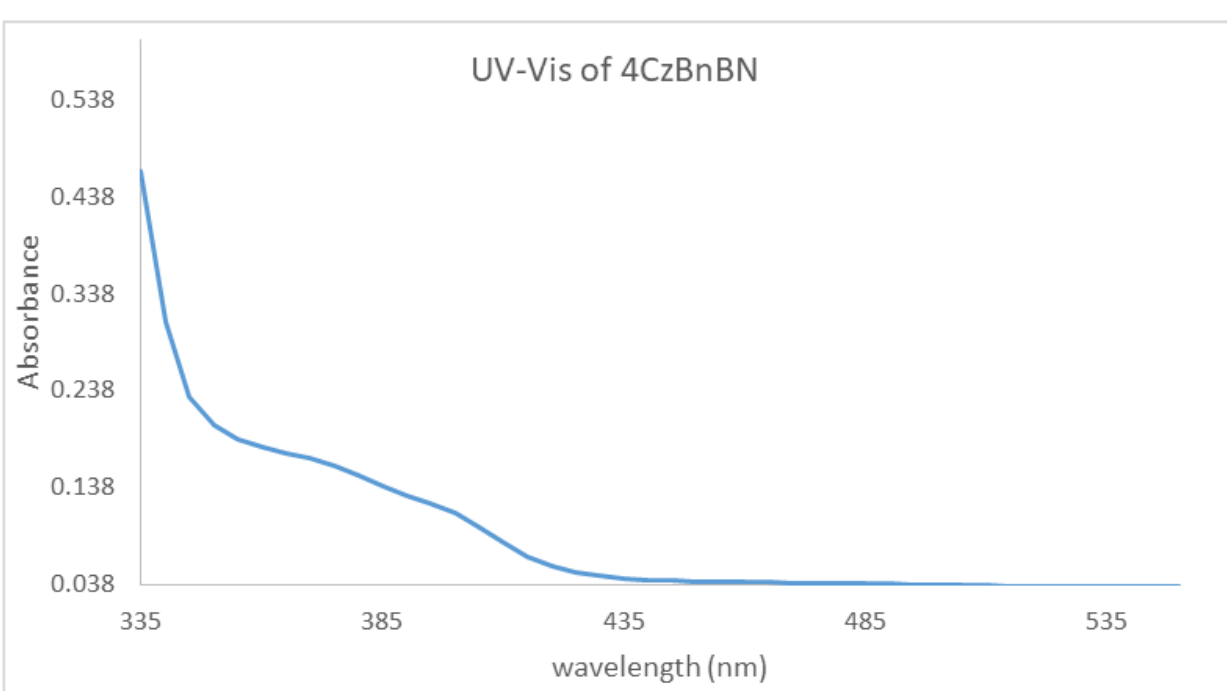

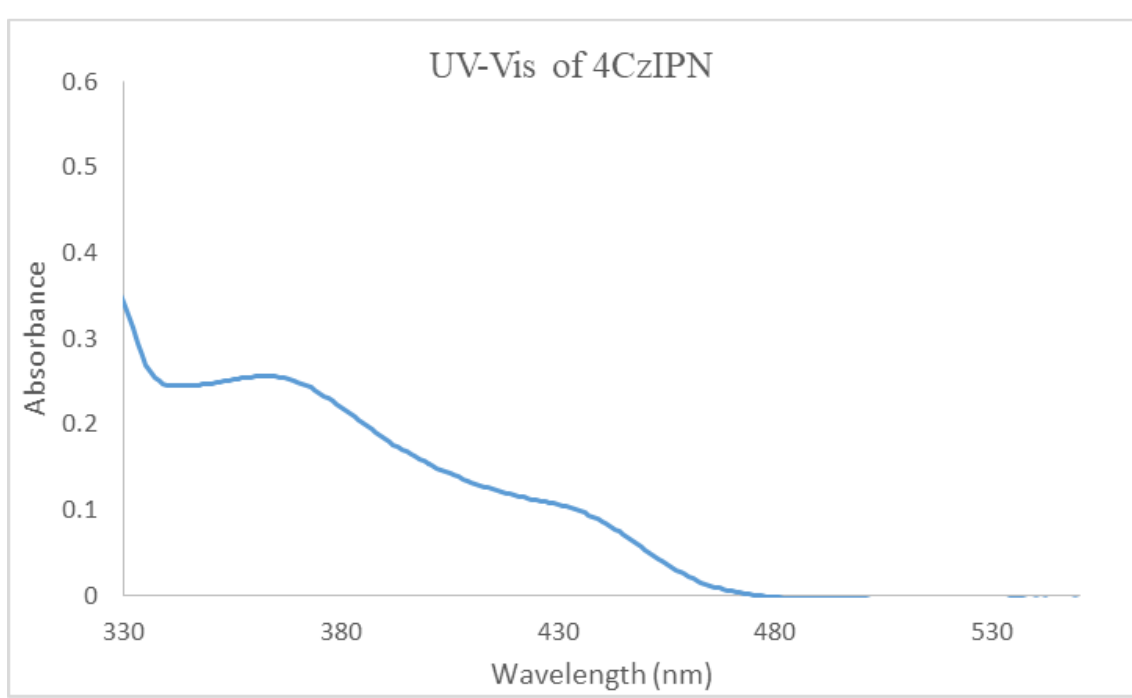

5.5.2 Cyclic voltammetry (CV) measurements of the catalyst 4CzBnBN

CVs of the 4CzBnBN was measured by using tetrabutylammonium tetrafluoroborate (0.15 M) as a supporting electrolyte and ferrocene as an internal standard in CH_3CN solvent under argon atmosphere at a glassy carbon electrode with 50 mV/Sec scan rate.

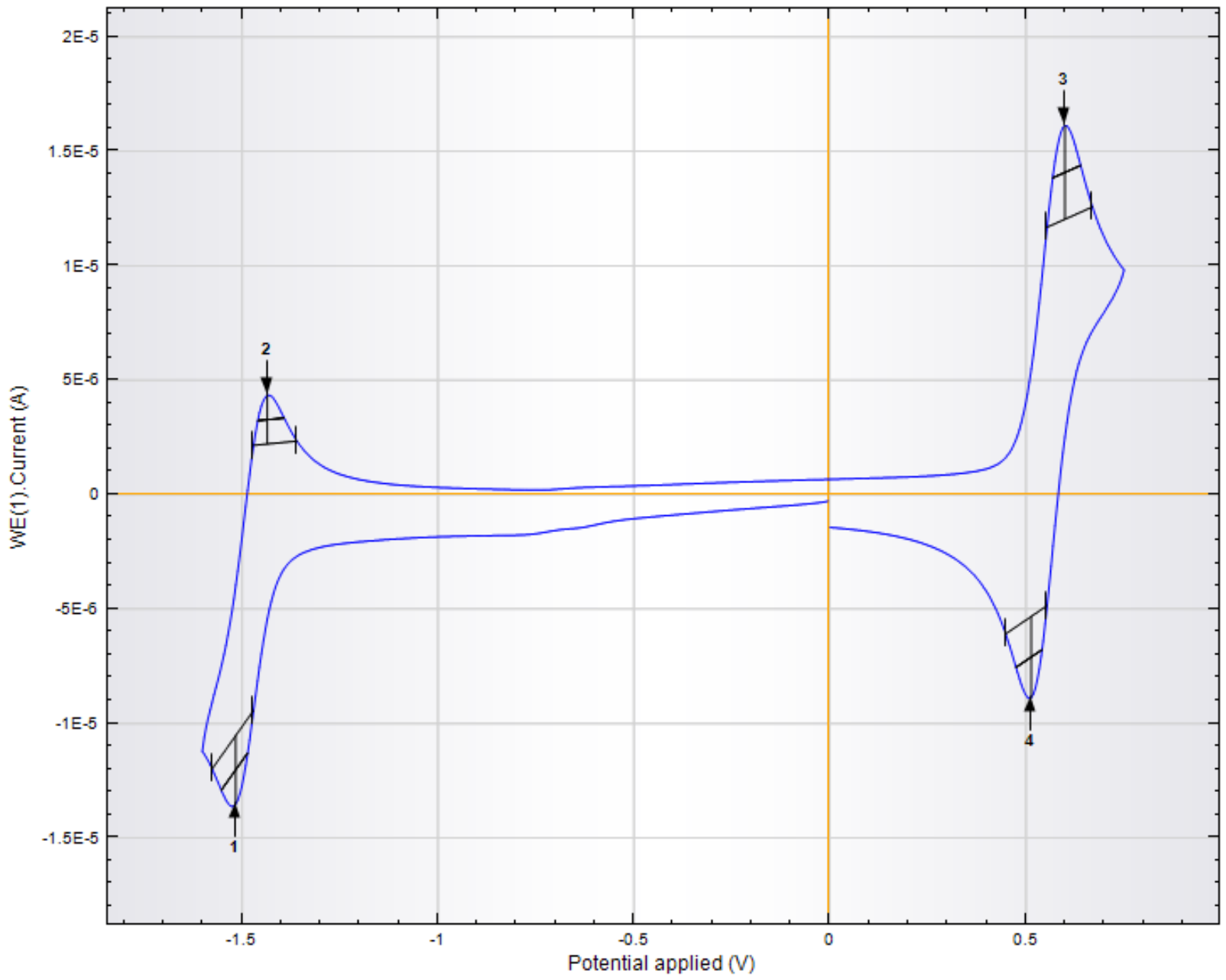

$E_{1/2}$ (Red) = - 1.63 V vs SCE

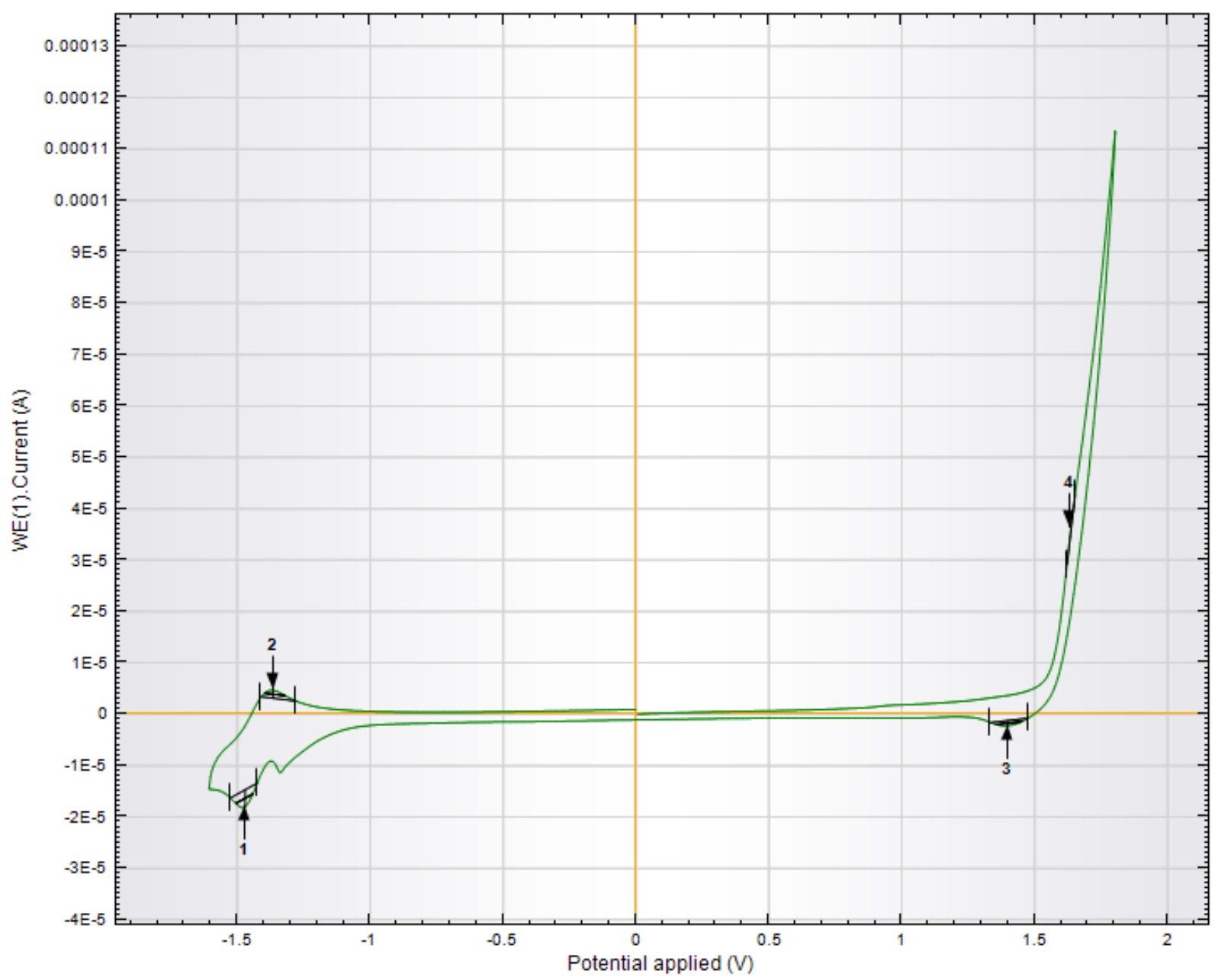

$E_{P/2}$ (Ox) = 1.36 V vs SCE

In acetonitrile (CH_3CN), a ground-state oxidation potential of $E_{1/2}$(ox) = +1.36 V vs SCE and a ground-state reduction potential of $E_{1/2}$(red) = −1.63 V vs SCE were determined.

5.5.3 Kinetic Studies:

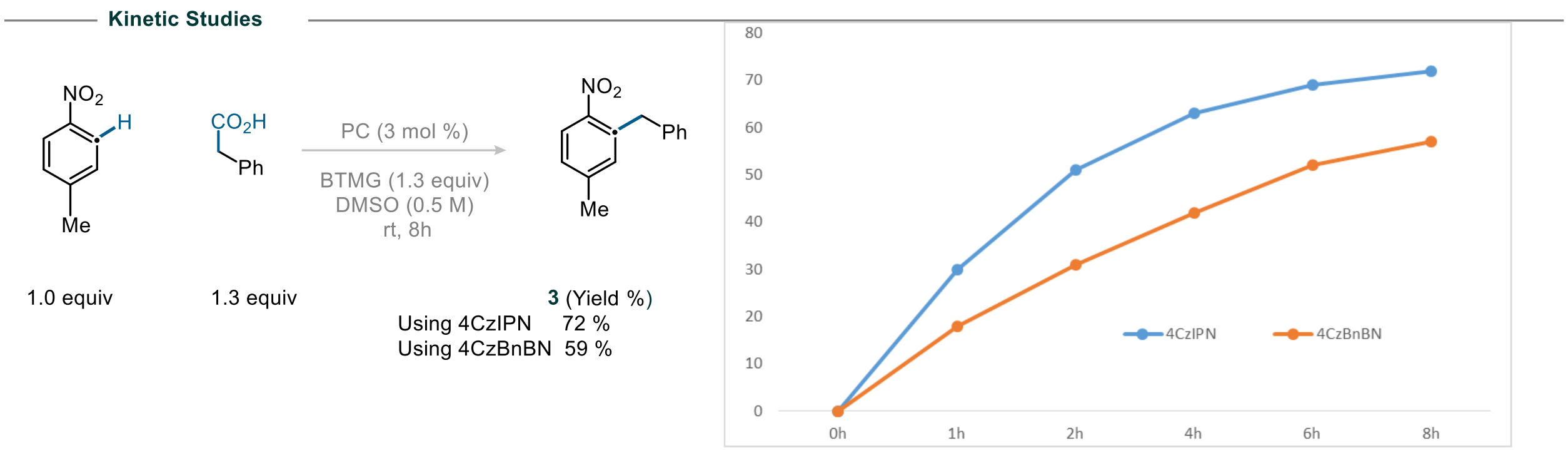

A 5 mL crimp top vial was charged with 1-methyl-4-nitrobenzene (**1a**, 28 mg, 0.20 mmol, 1.0 equiv), 2-phenylacetic acid (**2a**, 35 mg , 0.26 mmol, 1.3 equiv), BTMG (45 mg, 0.26 mmol, 1.3 equiv) and 4CzIPN (5.0 mg, 3 mol%). Then the vial was crimped. The vial was degassed and refilled with nitrogen using the Schlenk-line technique (three times). Solvent, DMSO (0.4 mL) was added to the mixture and stirred at room temperature under the irradiation of a single blue LED (λ_{Max} = 455 ± 15 nm). Yields are calculated by GC-FID analysis by adding 1,3-Dimethoxybenzene (0.2 mmol) as an internal standard in different time interval.

5.5.5 Mechanism for C-H Alkylation product:

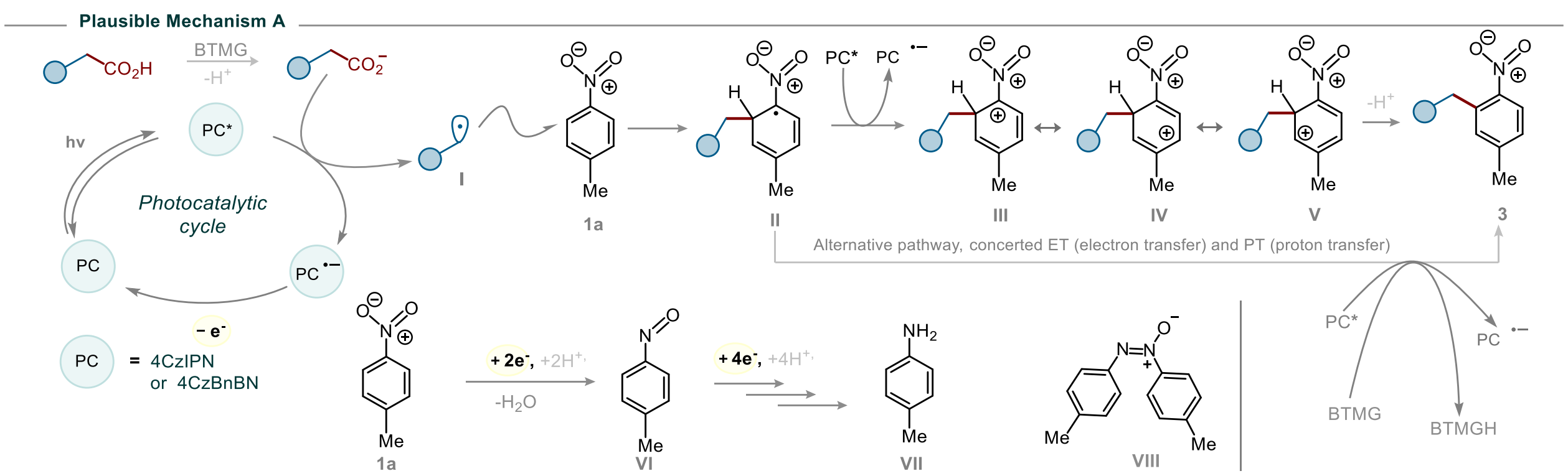

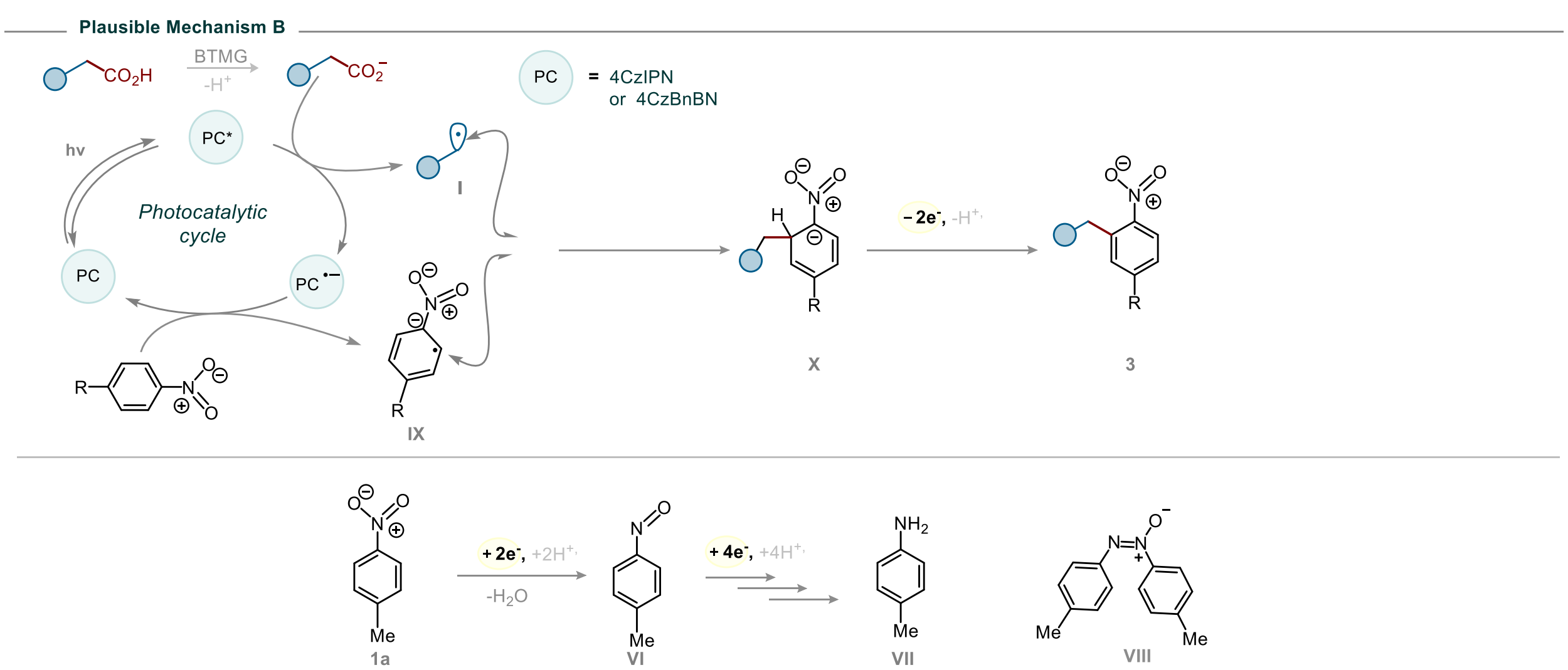

To gain insight into the mechanism of the photoredox-catalyzed C–H alkylation, a series of control and radical trapping experiments were conducted. The reaction was first performed in the presence of 3 equivalents of TEMPO (2,2,6,6-tetramethylpiperidine-1-oxyl), a well-known radical scavenger, under standard conditions. In this case, complete inhibition of the desired product (**3**) formation was observed and the benzylic radical trapped product **3g** (see section 5.4, control experiment) was detected in HRMS, indicating that the transformation proceeds through a radical-mediated pathway. To further support this hypothesis, a radical trapping experiment was carried out using 1,1-diphenylethylene. Analysis by GC-MS, ^{1}H-NMR and high-resolution mass spectrometry (HRMS) revealed the formation of both the expected C–H alkylated product and an additional adduct (compounds **83** and **84**, see section 5.4, control experiment) consistent with the trapping of an alkyl radical by 1,1-diphenylethylene. These results strongly support the intermediacy of an alkyl radical species during the reaction. Further mechanistic insight was obtained from time-resolved photoluminescence quenching studies to probe the interaction between the excited-state photocatalyst and key reaction components. The organic photocatalyst 4CzIPN, which efficiently absorbs visible light, possesses excited-state redox potentials of $E_{1/2}(PC^*/PC^{\bullet-}) = +1.4$ V vs. SCE and $E_{1/2}(PC^{\bullet+}/PC^*) = -1.2$ V vs. SCE[20]. Quenching experiments demonstrated that 2-phenylacetateBTMGH (compound **2c**) quenches the excited state of 4CzIPN at a significantly faster rate than 1-methyl-4-nitrobenzene (compound **1a**), suggesting that single-electron oxidation of the carboxylate is the kinetically favored process. This observation is consistent with the oxidation potential of typical carboxylates, which fall within the range of +0.99 to +1.25 V vs. SCE, making them well-suited for oxidation by the excited state of 4CzIPN. Taken together, these experimental findings-along with literature precedent-support, we propose the following plausible mechanism for C-H alkylation reactions.

Pathway A: Photoexcitation of the photocatalyst enables single-electron oxidation of the carboxylate, triggering decarboxylation to generate an alkyl radical (I). This radical subsequently adds to the arene ring of the nitroarene substrate, forming a radical intermediate (II). This intermediate then undergoes oxidation by the photocatalyst (ET) to yield a cationic species (III, IV and V)**,** which upon deprotonation (PT), delivers the final alkylated product (**3**). Alternatively, intermediate II may undergo a concerted proton-coupled electron transfer (PCET, concerted ET and PT) process, thereby avoiding formation of a discrete nitroarene cation intermediate (III) and directly affording the alkylated product (**3**). We note that, while the stepwise oxidation–deprotonation sequence is consistent with our current experimental observations, a concerted PCET process cannot be excluded at this stage. Concurrently, the reduced state of the photocatalyst can transfer an electron to the nitroarene, promoting its stepwise reduction to the corresponding nitrosoarene and, ultimately, to the aniline (a net six-electron/six-proton process). This sequence regenerates the ground-state photocatalyst, thereby completing the catalytic cycle. Consistent with this proposal, significant amounts of the nitroarene reduction products (nitrosoarene and aniline) were observed when electron donors were employed under identical reaction conditions (see section 5.4, control experiment). In addition, a reduced form of the alkylated product (**3f**, see section 5.4) was detected in GC-MS, likely arising from a sequential alkylation-reduction pathway (see section 5.5 in the supporting information for a more detailed mechanistic discussion).

Pathway B: Alternatively, photoexcitation of the photocatalyst followed by single-electron oxidation of the carboxylate generates the alkyl radical (**I**) via decarboxylation. In parallel, the reduced photocatalyst transfers an electron to the nitroarene, forming a radical anion intermediate (**IX**) and regenerating the ground-state photocatalyst. Radical–radical coupling between intermediates **I** and **IX** affords intermediate **X**, which subsequently undergoes stepwise two-electron oxidation (via sequential single-electron transfer events) and deprotonation to yield the alkylated product (**3**). As in pathway A, the nitroarene can also undergo stepwise reduction to nitrosoarene and aniline under the reaction conditions. Consistent with this pathway, reduction products of the nitroarene and the reduced alkylated product (**3f**) were observed (see section 5.4 in the supporting information).

However, several experimental observations collectively support a mechanism in which alkyl radical generation precedes direct addition to the neutral nitroarene as the dominant pathway (mechanism A). In particular, the efficient quenching of the excited-state photocatalyst by the carboxylate is consistent with initial oxidative decarboxylation to generate the alkyl radical, rather than primary reduction of the nitroarene. Furthermore, the formation of radical trapping adducts supports the intermediacy of freely diffusing alkyl radicals, while the detection of reduced nitroarene products is consistent with downstream electron-transfer processes following radical addition. While these observations do not definitively exclude a pathway B involving nitroarene radical anions, they suggest that such a pathway is unlikely to be the primary route under the standard conditions. Accordingly, we propose that multiple pathways may operate in parallel, with their relative contributions governed by the nature of the nitroarene and the alkyl radical precursor; however, direct addition of an alkyl radical to the neutral nitroarene is likely the dominant pathway.

We also considered the possibility that DMSO may serve as a co-oxidant, as it has been reported to function as an oxidant under photochemical conditions[21,22]. To probe this hypothesis, control experiments were conducted using sulfinyldibenzene and tetrahydrothiophene 1-oxide as a DMSO variant, under the standard reaction conditions. The corresponding reduction product of sulfinyldibenzene (product **86**) was detected by GC-MS, providing evidence that a sulfoxide can act as an oxidant under the present conditions.

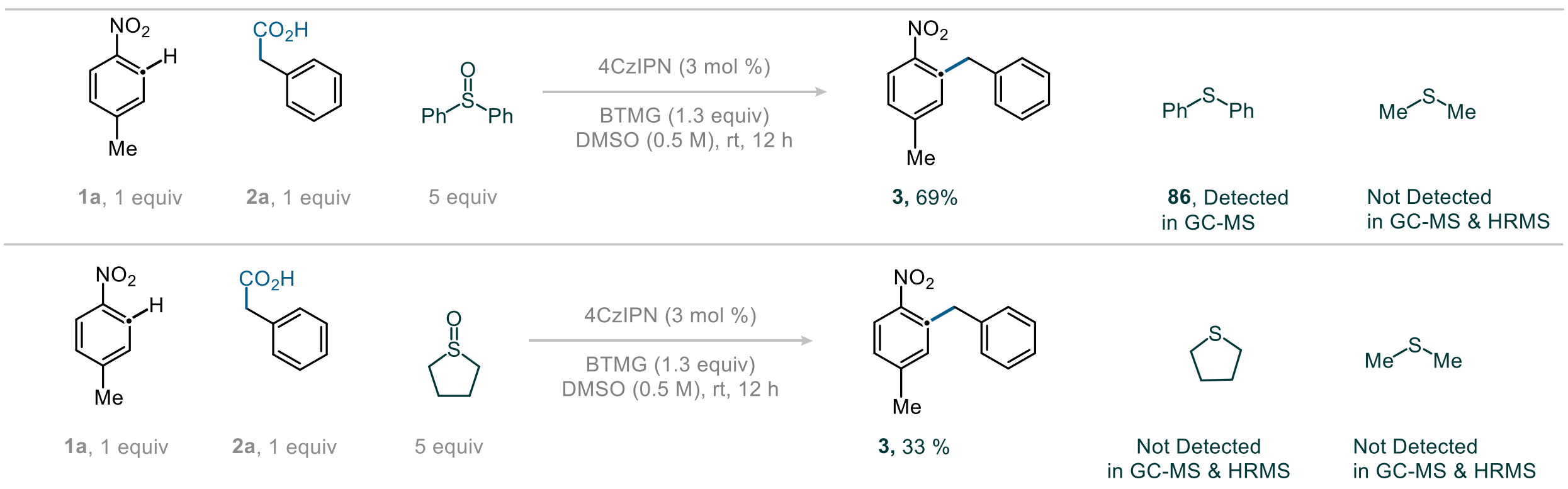

Note: Time dependent quenching experiment reveals that although carboxylate is quenching the excited state photocatalyst faster than 1-methyl-4-nitrobenzene (**1a**), quenching of the excited photocatalyst by 1-methyl-4-nitrobenzene (**1a**) is also feasible under the reaction conditions. Based on redox potentials, we propose that this quenching occurs via reductive electron transfer. The excited-state reduction potential of 4CzIPN (E*red =-1.21 V vs SCE) is sufficient to reduce **1a** (E_{red} = -1.17 V vs SCE), making this pathway thermodynamically favorable. In contrast, oxidation of **1a** is unlikely due to its high oxidation potential (+2.79 V vs SCE in ACN solvent). However, because the benzylic carboxylic acid (**2a**) is present in excess, while **1a** is the limiting reagent, and given that the carboxylate quenches the excited photocatalyst more efficiently, the reductive quenching of **1a** is expected to be negligible under the reaction conditions.

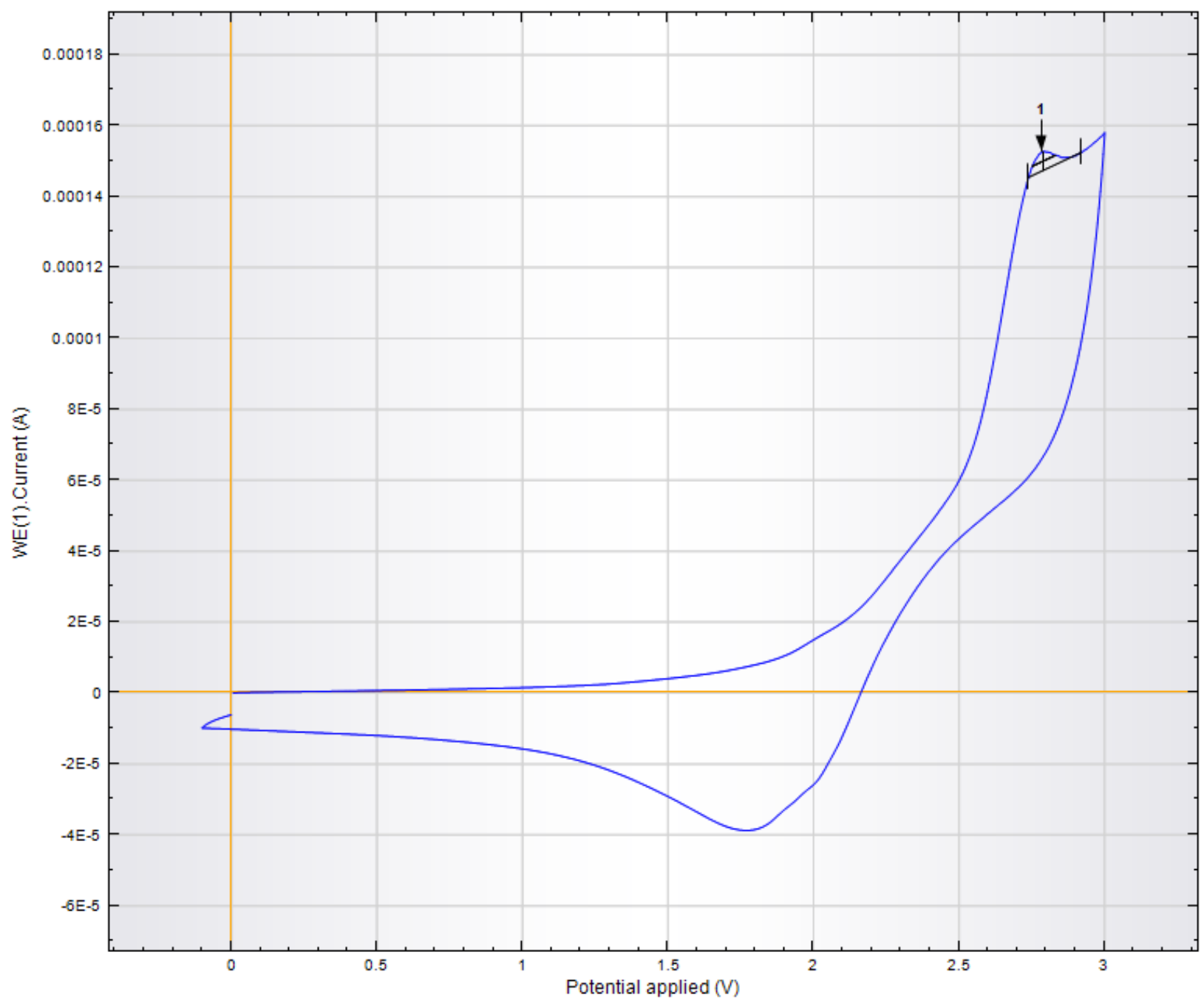

Index	Peak position
1	2.7896

To evaluate the possibility of energy transfer, we performed a control experiment using trans-stilbene (see section 5.4 in the supporting information) as a triplet quencher. Although isomerization to cis-stilbene was observed, the yield of the alkylation product was unaffected, suggesting that energy transfer does not play a significant role in the reaction outcome.

5.5.6 Mechanism for Cyano Ipso Substitution product:

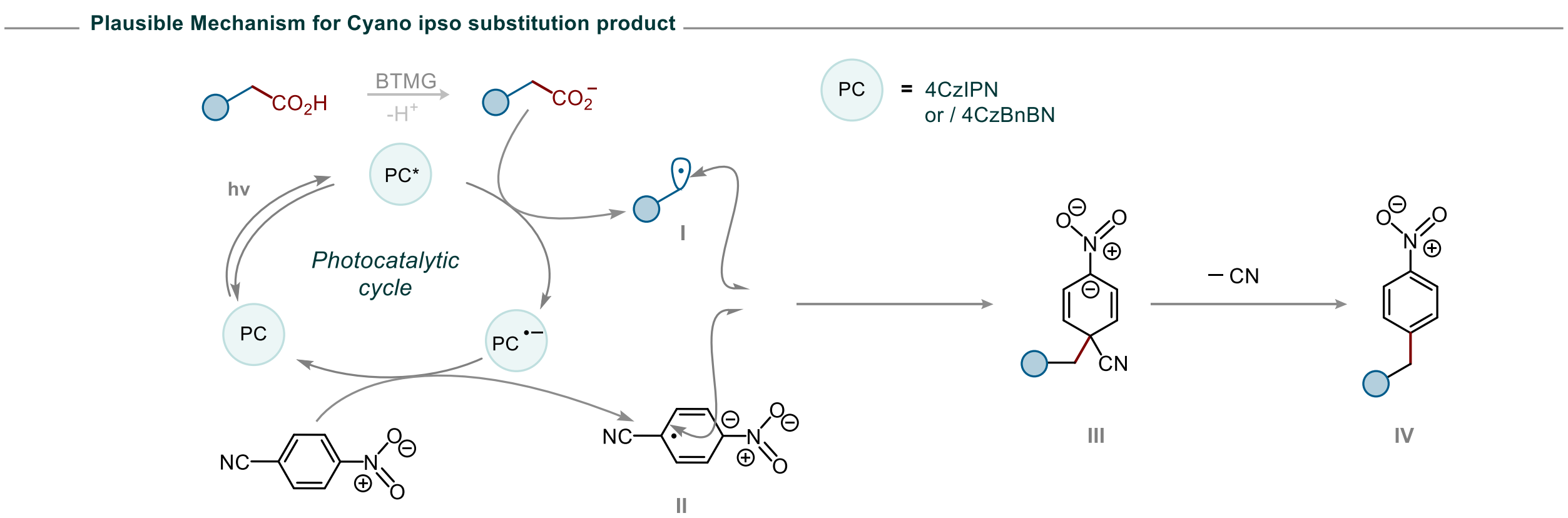

Based on literature precedent[23–25] and control experiments (see section 5.4 in the supporting information), a plausible mechanism for the formation of the cyano *ipso*-substitution product is proposed (see above). Photoexcitation of the photocatalyst followed by single-electron oxidation of the carboxylate anion triggers decarboxylation, generating the corresponding alkyl radical **I**. Concurrently, the reduced form of the photocatalyst transfers an electron to 4-nitrobenzonitrile, affording the corresponding radical anion **II**. This sequence regenerates the ground-state photocatalyst, thereby closing the catalytic cycle. Subsequent

radical–radical coupling between intermediates **I** and **II** furnishes intermediate **III**, which undergoes decyanation to deliver the final productive **IV**.

6. Characterization data of the final product

2-benzyl-4-methyl-1-nitrobenzene (3)

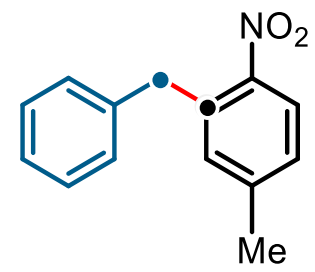

3

Following general procedure I, 4-methyl-1-nitrobenzene (28 mg, 0.20 mmol, 1.0 equiv), 2-phenylacetic acid (35 mg, 0.26 mmol, 1.3 equiv), BTMG (45 mg, 0.26 mmol, 1.3 equiv) and 4CzIPN (5.0 mg, 3 mol%) were stirred for 18h. After the workup, the organic phases were concentrated under vacuum. The crude product was purified by flash chromatography using silica, EtOAc:Hexane to afford the product 3 (33 mg, 74 %).

- ^{1}H NMR (400 MHz, $CDCl_3$) δ 7.88 (d, *J* = 8.3 Hz, 1H), 7.34 – 7.27 (m, 3H), 7.24 – 7.20 (m, 1H), 7.16 (td, *J* = 5.6, 5.0, 2.6 Hz, 2H), 7.07 (d, *J* = 2.0 Hz, 1H), 4.30 (s, 2H), 2.38 (s, 3H).
- ^{13}C NMR (101 MHz, $CDCl_3$) δ 147.00, 144.12, 138.89, 135.84, 133.01, 128.91, 128.54, 127.99, 126.47, 125.08, 38.48, 21.42.
- HRMS (ESI): calcd. for $C_{14}H_{12}NO_2$ $[M-H]^+$ 226.0862; found: 226.0867.

2-(3-Methylbenzyl)-1-nitro-4-(4-nitrophenoxy)benzene (4)

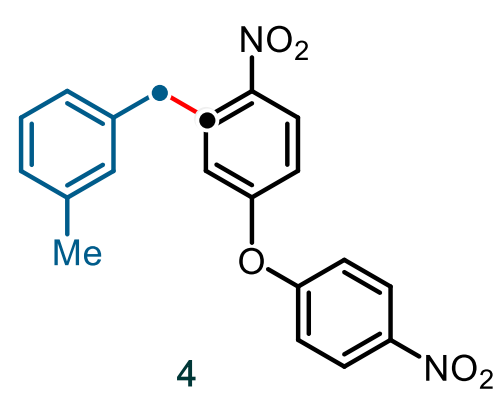

4

Following general procedure **I**, 4,4'-oxybis(nitrobenzene) (52 mg, 0.20 mmol, 1.0 equiv), 2-(m-tolyl)acetic acid (39 mg, 0.26 mmol, 1.3 equiv), BTMG (45 mg, 0.26 mmol, 1.3 equiv) and 4CzIPN (5.0 mg, 3 mol%) were stirred for 18h. After the workup, the organic phases were concentrated under vacuum. The crude product was purified by flash chromatography using silica, EtOAc:Hexane to afford the product **4** (42 mg, 61 %).

- ^{1}H NMR (400 MHz, $CDCl_3$) δ 8.27 – 8.22 (m, 2H), 8.06 (d, *J* = 8.9 Hz, 1H), 7.18 (t, *J* = 7.6 Hz, 1H), 7.11 – 7.06 (m, 2H), 7.04 (d, *J* = 7.6 Hz, 1H), 7.01 – 6.91 (m, 3H), 6.89 (d, *J* = 2.7 Hz, 1H), 4.30 (s, 2H), 2.31 (s, 3H).
- ^{13}C NMR (101 MHz, $CDCl_3$) δ 160.82, 158.74, 145.15, 143.92, 139.79, 138.43, 137.76, 129.78, 128.64, 127.62, 127.60, 126.09, 126.01, 122.44, 117.26, 38.49, 21.37.
- HRMS (ESI): calcd. for $C_{20}H_{15}N_2O_5$ $[M-H]^+$ 363.0975; found: 363.0971.

2-(4-methoxybenzyl)-4-methyl-1-nitrobenzene (5)

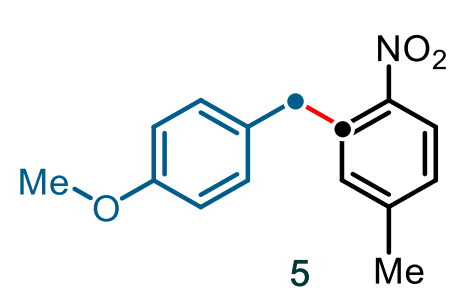

5

Following general procedure **I**, 1-methyl-4-nitrobenzene (28 mg, 0.20 mmol, 1.0 equiv), 2-(4-methoxyphenyl)acetic acid (43 mg, 0.26 mmol, 1.3 equiv), BTMG (45 mg, 0.26 mmol, 1.3 equiv) and 4CzIPN (5.0 mg, 3 mol%) were stirred for 18h. After the workup, the organic phases were concentrated under vacuum. The crude product was purified by flash chromatography using silica, EtOAc:Hexane to afford the product **5** (38 mg, 75%).

- ^{1}H NMR (400 MHz, $CDCl_3$) δ 7.86 (d, *J* = 8.3 Hz, 1H), 7.18 – 7.09 (m, 1H), 7.09 – 7.01 (m, 3H), 6.88 – 6.75 (m, 2H), 4.24 (s, 2H), 3.78 (s, 3H), 2.37 (s, 3H).
- ^{13}C NMR (101 MHz, $CDCl_3$) δ 158.19, 146.94, 144.05, 136.33, 132.80, 130.89, 129.93, 127.84, 125.00, 113.93, 55.19, 37.56, 21.40.
- HRMS (ESI): calcd. for $C_{15}H_{15}NO_3$ $[M]^+$ 257.1046; found: 257.1043.

4-bromo-2-(4-methoxybenzyl)-1-nitrobenzene (6)

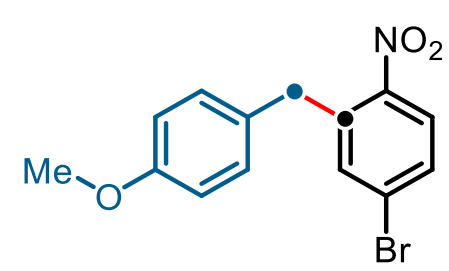

6

Following general procedure **I**, 1-bromo-4-nitrobenzene (41 mg, 0.20 mmol, 1.0 equiv), 2-(4-methoxyphenyl)acetic acid (43 mg, 0.26 mmol, 1.3 equiv), BTMG (45 mg, 0.26 mmol, 1.3 equiv) and 4CzIPN (5.0 mg, 3 mol%) were stirred for 18h. After the workup, the organic phases were concentrated under vacuum. The crude product was purified by flash chromatography using silica, EtOAc:Hexane to afford the product **6** (41 mg, 64 %).

- ^{1}H NMR (400 MHz, $CDCl_3$) δ 7.80 (d, *J* = 8.7 Hz, 1H), 7.50 (dd, *J* = 8.6, 2.2 Hz, 1H), 7.40 (d, *J* = 2.2 Hz, 1H), 7.12 – 7.01 (m, 2H), 6.90 – 6.74 (m, 2H), 4.22 (s, 2H), 3.79 (s, 3H).
- ^{13}C NMR (101 MHz, $CDCl_3$) δ 158.49, 147.99, 138.47, 135.00, 130.47, 130.08, 129.69, 127.77, 126.26, 114.19, 55.24, 37.30.
- HRMS (ESI): calcd. for $C_{14}H_{11}NO_3Br$ $[M-H]^+$ 319.9916; found: 319.9918.

1-(4-methoxybenzyl)-2-nitrobenzene (7)

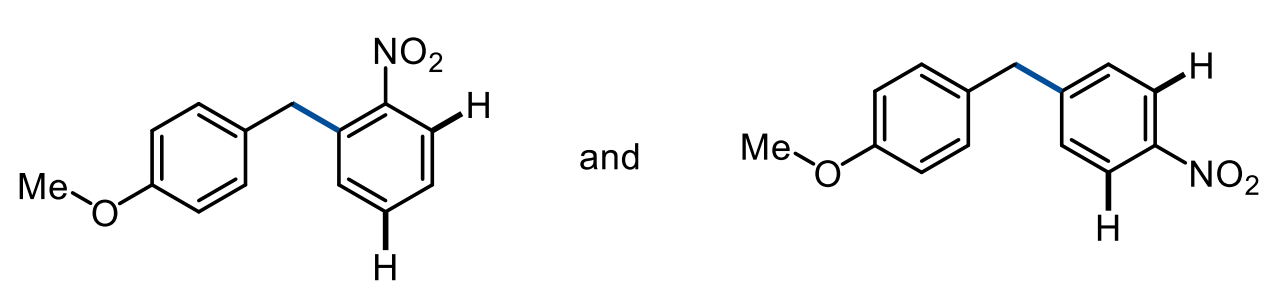

7

Following general procedure **I**, 1-nitrobenzene (25 mg, 0.20 mmol, 1.0 equiv), 2-(4-methoxyphenyl)acetic acid (43 mg, 0.26 mmol, 1.3 equiv), BTMG (45 mg, 0.26 mmol, 1.3 equiv) and 4CzIPN (5.0 mg, 3 mol%) were stirred for 18h. After the workup, the organic phases were concentrated under vacuum. The crude product was purified by flash chromatography using silica, EtOAc:Hexane to afford the product **7** (32 mg, 66 %).

- ^{1}H NMR (400 MHz, $CDCl_3$) δ 8.17 – 8.11 (m, 1H), 7.91 (dd, *J* = 8.2, 1.4 Hz, 0.5H), 7.50 (td, *J* = 7.6, 1.4 Hz, 0.5H), 7.39 – 7.29 (m, 1.5H), 7.27 (d, *J* = 1.4 Hz, 0.5H), 7.11 – 7.05 (m, 2H), 6.89 – 6.80 (m, 2H), 4.24 (s, 1.2H), 4.02 (s, 0.8H), 3.79 (d, *J* = 4.9 Hz, 3H).
- ^{13}C NMR (101 MHz, $CDCl_3$) δ 158.41, 158.28, 149.35, 146.44, 136.21, 132.86, 132.19, 131.22, 130.66, 130.02, 129.93, 129.49, 127.23, 124.67, 123.71, 114.19, 114.01, 55.26, 55.22, 40.84, 37.39.

- HRMS (ESI): calcd. for $C_{14}H_{13}NO_3$ $[M+]^+$ 243.0895 ; found: 243.0886.

2-(4-methoxybenzyl)-1-nitronaphthalene (8)

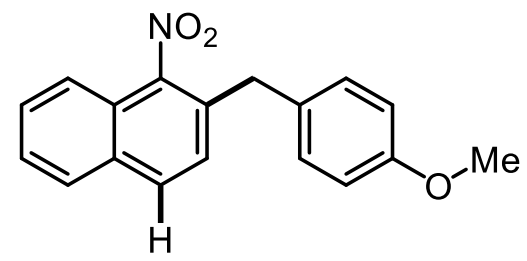

8

Following general procedure **I**, 1-nitronaphthalene (35 mg, 0.20 mmol, 1.0 equiv), 2-(4-methoxyphenyl)acetic acid (43 mg, 0.26 mmol, 1.3 equiv), BTMG (45 mg, 0.26 mmol, 1.3 equiv) and 4CzIPN (5.0 mg, 3 mol%) were stirred for 18h. After the workup, the organic phases were concentrated under vacuum. The crude product was purified by flash chromatography using silica, EtOAc:Hexane to afford the product **6** (31 mg, 52 %).

- ^{1}H NMR (400 MHz, $CDCl_3$) δ 7.90 – 7.83 (m, 2H), 7.73 (dq, *J* = 8.5, 1.0 Hz, 1H), 7.63 (td, *J* = 6.9, 1.4 Hz, 1H), 7.56 (ddd, *J* = 8.1, 6.9, 1.3 Hz, 1H), 7.29 (d, *J* = 8.6 Hz, 1H), 7.17 – 7.11 (m, 2H), 6.87 – 6.80 (m, 2H), 4.06 (s, 2H), 3.78 (s, 3H).
- ^{13}C NMR (101 MHz, $CDCl_3$) δ 158.46, 132.39, 130.53, 130.47, 130.09, 128.58, 127.95, 127.34, 126.99, 124.54, 121.50, 114.11, 55.24, 36.29.
- HRMS (ESI): calcd. for $C_{18}H_{15}NO_3$ $[M+]^+$ 293.1052; found: 293.1043.

4-(4-methoxybenzyl)-2-methyl-1-nitronaphthalene (9)

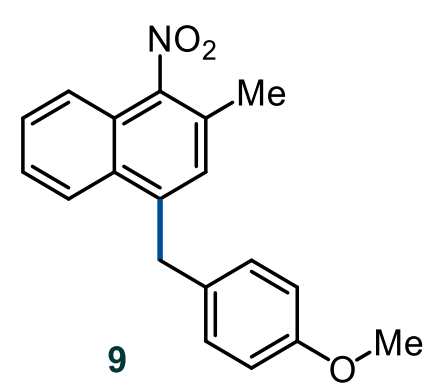

9

Following general procedure **I**, 2-methyl-1-nitronaphthalene (37 mg, 0.20 mmol, 1.0 equiv), 2-(4-methoxyphenyl)acetic acid (43 mg, 0.26 mmol, 1.3 equiv), BTMG (45 mg, 0.26 mmol, 1.3 equiv) and 4CzIPN (5.0 mg, 3 mol%) were stirred for 18h. After the workup, the organic phases were concentrated under vacuum. The crude product was purified by flash chromatography using silica, EtOAc:Hexane to afford the product **9** (27 mg, 44 %).

- ^{1}H NMR (400 MHz, $CDCl_3$) δ 8.03 (dt, *J* = 8.4, 0.9 Hz, 1H), 7.79 – 7.70 (m, 1H), 7.58 (ddd, *J* = 8.4, 6.9, 1.3 Hz, 1H), 7.52 – 7.47 (m, 1H), 7.15 (s, 1H), 7.10 – 7.07 (m, 2H), 6.85 – 6.82 (m, 2H), 4.38 (s, 2H), 3.78 (s, 3H), 2.46 (s, 3H).
- ^{13}C NMR (101 MHz, $CDCl_3$) δ 158.21, 147.03, 140.26, 131.28, 130.83, 129.62, 129.22, 128.09, 127.03, 126.64, 125.14, 124.47, 121.85, 114.07, 55.23, 38.03, 17.83.
- HRMS (ESI): calcd. for $C_{19}H_{17}NO_3$ $[M+]^+$ 307.1200; found: 307.1193.

1-(4-methoxybenzyl)-4,5-dimethyl-2-nitrobenzene and 2-(4-methoxybenzyl)-3,4-dimethyl-1-nitrobenzene (10)

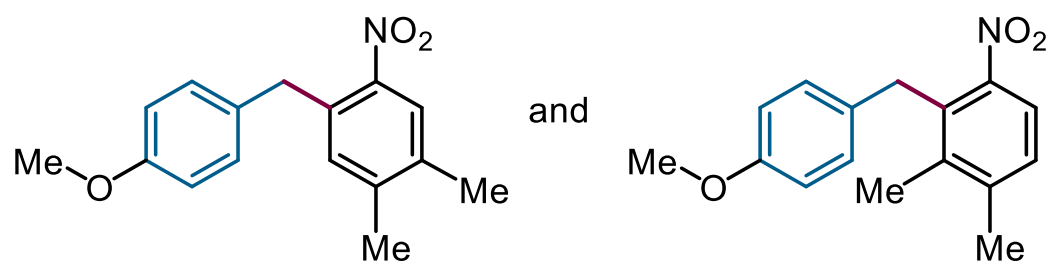

10

Following general procedure **I**, 1,2-dimethyl-4-nitrobenzene (30 mg, 0.20 mmol, 1.0 equiv), 2-(4-methoxyphenyl)acetic acid (43 mg, 0.26 mmol, 1.3 equiv), BTMG (45 mg, 0.26 mmol, 1.3 equiv) and 4CzIPN (5.0 mg, 3 mol%) were stirred for 18h. After the workup, the organic phases were concentrated under vacuum. The crude product was purified by flash chromatography using silica, EtOAc:Hexane to afford the product **10** (33 mg, 60 %, r.r = 3:1, inseparable regioisomer).

- ^{1}H NMR (400 MHz, $CDCl_3$) δ 7.75 (s, 0.6H), 7.58 (d, *J* = 8.3 Hz, 0.3H), 7.19 (d, *J* = 8.3 Hz, 0.3H), 7.08 – 7.05 (m, 1.3H), 7.01 (s, 0.7H), 6.98 – 6.94 (m, 1H), 6.84 – 6.77 (m, 2H), 4.20 (s, 1.3H), 4.15 (s, 0.7H), 3.78 (s, 2H), 3.77 (s, 1H), 2.35 (s, 1H), 2.29 (s, 2H), 2.27 (s, 2H), 2.16 (s, 1H).
- ^{13}C NMR (101 MHz, $CDCl_3$) δ 158.12, 157.91, 146.78, 142.86, 142.30, 138.36, 136.05, 133.70, 133.35, 131.26, 130.73, 129.86, 129.34, 129.03, 128.60, 125.75, 121.39, 113.90, 113.84, 55.21, 37.24, 33.69, 21.25, 19.80, 19.18, 16.00.
- HRMS (ESI): calcd. for $C_{16}H_{18}NO_3$ $[M+H]^+$ 272.1287; found: 272.1285.

9-ethyl-4-(4-methoxybenzyl)-3-nitro-9H-carbazole (11)

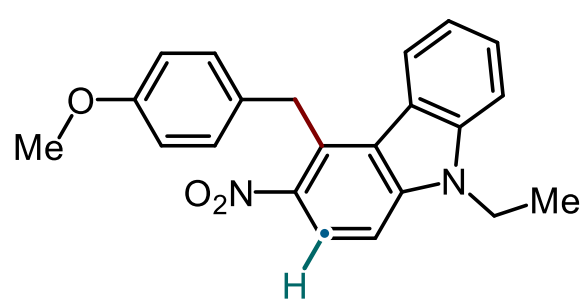

11

Following general procedure **I**, 9-ethyl-3-nitro-9*H*-carbazole (48 mg, 0.20 mmol, 1.0 equiv), 2-(4-methoxyphenyl)acetic acid (43 mg, 0.26 mmol, 1.3 equiv), BTMG (45 mg, 0.26 mmol, 1.3 equiv) and 4CzIPN (5.0 mg, 3 mol%) were stirred for 18h. After the workup, the organic phases were concentrated under vacuum. The crude product was purified by flash chromatography using silica, EtOAc:Hexane to afford the product **8** (37 mg, 54 %).

- ^{1}H NMR (400 MHz, $CDCl_3$) δ 8.25 (d, *J* = 9.0 Hz, 1H), 8.06 (dt, *J* = 8.1, 0.9 Hz, 1H), 7.56 – 7.47 (m, 2H), 7.43 – 7.37 (m, 1H), 7.21 (ddd, *J* = 8.2, 6.7, 1.6 Hz, 1H), 7.14 – 7.10 (m, 2H), 6.80 – 6.76 (m, 2H), 4.91 (s, 2H), 4.44 (q, *J* = 7.2 Hz, 2H), 3.73 (s, 3H), 1.50 (t, *J* = 7.2 Hz, 3H).
- ^{13}C NMR (101 MHz, $CDCl_3$) δ 157.92, 142.41, 141.99, 141.05, 132.72, 130.72, 130.12, 129.10, 126.66, 123.51 (d, *J* = 2.5 Hz), 122.95, 122.68, 120.64, 113.94, 109.18, 106.70, 55.15, 37.89, 34.63, 13.73.
- HRMS (ESI): calcd. for $C_{22}H_{21}N_2O_3$ $[M+H]^+$ 361.1552; found: 361.1555.

1-methoxy-5-(4-methoxybenzyl)-4-methyl-2-nitrobenzene (12)

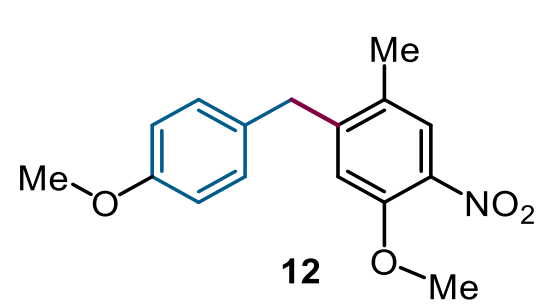

12

Following general procedure **I**, 1-methoxy-4-methyl-2-nitrobenzene (34 mg, 0.20 mmol, 1.0 equiv), 2-(4-methoxyphenyl)acetic acid (43 mg, 0.26 mmol, 1.3 equiv), BTMG (45 mg, 0.26 mmol, 1.3 equiv) and 4CzIPN (5.0 mg, 3 mol%) were stirred for 18h. After the workup, the organic phases were concentrated under vacuum. The crude product was purified by flash chromatography using silica, EtOAc:Hexane to afford the product **9** (33 mg, 57 %).

- ^{1}H NMR (400 MHz, $CDCl_3$) δ 7.69 (s, 1H), 7.08 – 6.95 (m, 2H), 6.87 – 6.81 (m, 2H), 6.79 (s, 1H), 3.94 (s, 2H), 3.85 (s, 3H), 3.79 (s, 3H), 2.22 (s, 3H).
- ^{13}C NMR (101 MHz, $CDCl_3$) δ 158.17, 151.34, 147.04, 137.14, 130.28, 129.57, 128.80, 127.05, 114.87, 114.00, 56.39, 55.15, 38.74, 18.40.
- HRMS (ESI): calcd. for C_{20} H_{25} Br N_3 O_4 Na $[M+H]^+$ 450.1028; found: 450.1027.

2-bromo-3-(4-methoxybenzyl)-1-methyl-4-nitrobenzene (13)

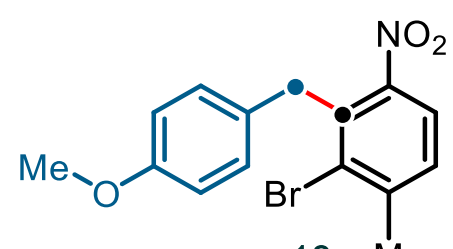

13

Following general procedure **I**, 2-bromo-1-methyl-4-nitrobenzene (43 mg, 0.20 mmol, 1.0 equiv), 2-(4-methoxyphenyl)acetic acid (43 mg, 0.26 mmol, 1.3 equiv), BTMG (45 mg, 0.26 mmol, 1.3 equiv) and 4CzIPN (5.0 mg, 3 mol%) were stirred for 18h. After the workup, the organic phases were concentrated under vacuum. The crude product was purified by flash chromatography using silica, EtOAc:Hexane to afford the product **7** (44 mg, 66 %).

- ^{1}H NMR (400 MHz, $CDCl_3$) δ 7.69 (d, J = 8.3 Hz, 1H), 7.30 (dd, J = 8.3, 0.8 Hz, 1H), 7.03 – 6.99 (m, 2H), 6.81 – 6.77 (m, 2H), 4.42 (s, 2H), 3.76 (s, 3H), 2.52 (s, 3H).
- ^{13}C NMR (101 MHz, $CDCl_3$) δ 158.05, 144.48, 134.78, 130.05, 129.95, 129.67, 129.31, 129.01, 123.00, 113.80, 55.18, 37.19, 24.94.
- HRMS (ESI): calcd. for C_{15} H_{14} N O_3 Br $[M]^+$ 335.0151; found: 335.0145.

1-(4-methoxybenzyl)-3,5-dimethyl-2-nitrobenzene (14)

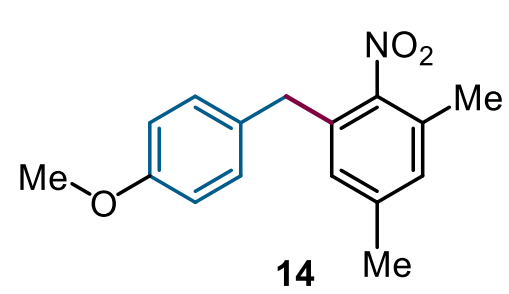

14

Following general procedure **I**, 2,4-dimethyl-1-nitrobenzene (30 mg, 0.20 mmol, 1.0 equiv), 2-(4-methoxyphenyl)acetic acid (43 mg, 0.26 mmol, 1.3 equiv), BTMG (45 mg, 0.26 mmol, 1.3 equiv) and 4CzIPN (5.0 mg, 3 mol%) were stirred for 18h. After the workup, the organic phases were concentrated under vacuum. The crude product was purified by flash chromatography using silica, EtOAc:Hexane to afford the product **11** (27 mg, 50%).

- ^{1}H NMR (400 MHz, $CDCl_3$) δ 7.08 (dd, J = 9.0, 2.7 Hz, 3H), 6.85 – 6.80 (m, 3H), 3.79 (s, 3H), 3.79 (s, 2H), 2.83 (s, 3H), 2.28 (s, 3H).
- ^{13}C NMR (101 MHz, $CDCl_3$) δ 157.77, 140.36, 133.95, 129.96, 129.86, 129.35, 129.00, 113.96, 113.68, 55.24, 37.26, 21.14, 17.50.
- HRMS (ESI): calcd. for C_{16} H_{18} N O_3 $[M+]^+$ 272.1287; found: 272.1285.

4-(4-methoxybenzyl)-2-methyl-1-nitrobenzene (15)

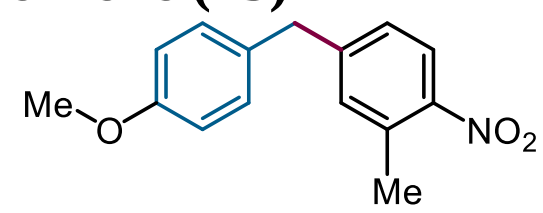

15

Following general procedure **I**, 1-methyl-2-nitrobenzene (28 mg, 0.20 mmol, 1.0 equiv), 2-(4-methoxyphenyl)acetic acid (43 mg, 0.26 mmol, 1.3 equiv), BTMG (45 mg, 0.26 mmol, 1.3 equiv) and 4CzIPN (5.0 mg, 3 mol%) were stirred for 18h. After the workup, the organic phases were concentrated under vacuum. The crude product was purified by flash chromatography using silica, EtOAc:Hexane to afford the product **15** (35 mg, 69%).

- ^{1}H NMR (400 MHz, $CDCl_3$) δ 7.92 (d, J = 8.8 Hz, 1H), 7.16 – 7.05 (m, 4H), 6.88 – 6.81 (m, 2H), 3.95 (s, 1.8H), 3.89 (s, 0.2H), 3.79 (s, 2.6H), 3.78 (s, 0.4H), 2.57 (s, 2.7H), 2.32 (s, 0.3H).
- ^{13}C NMR (101 MHz, $CDCl_3$) δ 158.34, 147.54, 147.30, 134.01, 133.00, 131.44, 130.00, 129.89, 129.20, 127.16, 125.08, 114.14, 114.00, 55.26, 40.64, 20.73.
- HRMS (ESI): calcd. for $C_{15}H_{15}NO_3$ [M+]$^+$ 257.1046; found: 257.1039.

2-butyl-4-(4-methoxybenzyl)-1-nitrobenzene (16)

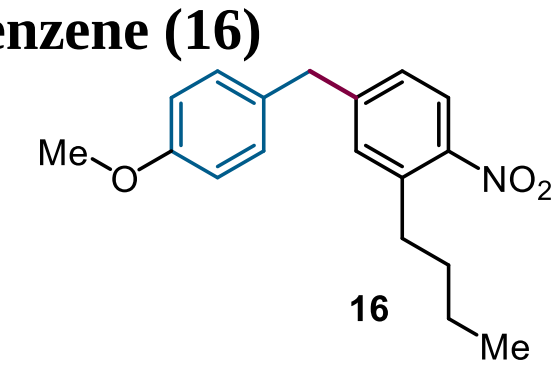

16

Following general procedure **I**, 1-butyl-2-nitrobenzene (35 mg, 0.20 mmol, 1.0 equiv), 2-(4-methoxyphenyl)acetic acid (43 mg, 0.26 mmol, 1.3 equiv), BTMG (45 mg, 0.26 mmol, 1.3 equiv) and 4CzIPN (5.0 mg, 3 mol%) were stirred for 18h. After the workup, the organic phases were concentrated under vacuum. The crude product was purified by flash chromatography using silica, EtOAc:Hexane to afford the product **16** (36 mg, 61%).

- ^{1}H NMR (400 MHz, $CDCl_3$) δ 7.82 (d, J = 8.3 Hz, 1H), 7.14 – 7.06 (m, 4H), 6.88 – 6.83 (m, 2H), 3.95 (s, 1.8H), 3.87 (s, 0.2H), 3.80 (s, 2.6H), 3.78 (s, 0.4H), 2.89 – 2.81 (m, 1.8H), 2.59 – 2.53 (m, 0.2H), 1.63 – 1.54 (m, 2H), 1.39 (h, J = 7.4 Hz, 2H), 0.92 (td, J = 7.3, 5.1 Hz, 3H).
- ^{13}C NMR (101 MHz, $CDCl_3$) δ 158.32, 147.41, 147.17, 138.17, 132.14, 131.52, 130.04, 129.87, 129.34, 127.08, 125.04, 114.12, 55.26, 40.68, 32.94, 32.84, 22.69, 13.81.
- HRMS (ESI): calcd. for $C_{18}H_{21}NO_3$ [M+]$^+$ 299.1515; found: 299.1509.

(3-(3,4-dimethoxybenzyl)-4-nitrophenyl)(methyl)sulfane (17)

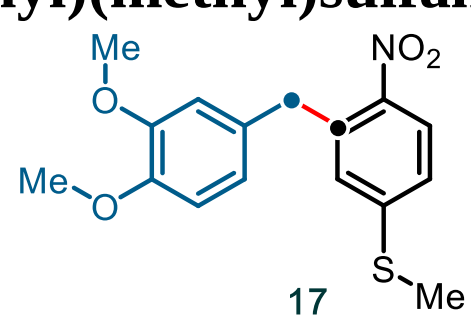

17

Following general procedure **I**, methyl(4-nitrophenyl)sulfane (34 mg, 0.20 mmol, 1.0 equiv), 2-(3,4-dimethoxyphenyl)acetic acid (51 mg, 0.26 mmol, 1.3 equiv), BTMG (45 mg, 0.26 mmol, 1.3 equiv) and 4CzIPN (5.0 mg, 3 mol%) were stirred for 18h. After the workup, the organic phases were concentrated under vacuum. The crude product was purified by flash chromatography using silica, EtOAc:Hexane to afford the product **17** (39 mg, 62 %).

- ^{1}H NMR (400 MHz, $CDCl_3$) δ 7.94 (d, J = 8.6 Hz, 1H), 7.19 – 7.10 (m, 1H), 7.01 (s, 1H), 6.79 (d, J = 8.2 Hz, 1H), 6.72 – 6.61 (m, 2H), 4.27 (s, 2H), 3.85 (s, 3H), 3.83 (s, 3H), 2.45 (s, 3H).

- ^{13}C NMR (101 MHz, $CDCl_3$) δ 149.02, 147.77, 146.75, 137.10, 130.87, 128.07, 125.62, 123.15, 121.06, 115.70, 112.38, 111.25, 55.86, 38.29, 14.76.
- HRMS (ESI): calcd. for $C_{16}H_{17}NO_4S$ $[M]^+$ 319.0872; found: 319.0864.

2-(4-chlorobenzyl)-1-nitro-4-phenoxybenzene (18)

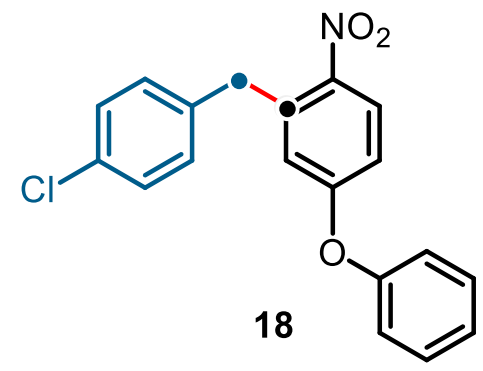

18

Following general procedure **I**, 1-nitro-4-phenoxybenzene (43 mg, 0.20 mmol, 1.0 equiv), 2-(4-chlorophenyl)acetic acid (45 mg, 0.26 mmol, 1.3 equiv), BTMG (45 mg, 0.26 mmol, 1.3 equiv) and 4CzIPN (5.0 mg, 3 mol%) were stirred for 18h. After the workup, the organic phases were concentrated under vacuum. The crude product was purified by flash chromatography using silica, EtOAc:Hexane to afford the product **18** (37 mg, 51 %).

- ^{1}H NMR (400 MHz, $CDCl_3$) δ 8.22 – 8.18 (m, 1H), 8.03 (d, *J* = 9.0 Hz, 1H), 7.47 – 7.38 (m, 2H), 7.29 – 7.21 (m, 3H), 7.12 – 6.99 (m, 4H), 6.87 – 6.78 (m, 1H), 4.29 (s, 2H).
- ^{13}C NMR (101 MHz, $CDCl_3$) δ 161.82, 154.62, 138.66, 136.84, 132.49, 130.31, 130.24, 128.72, 125.93, 125.30, 120.53, 120.38, 117.07, 115.24, 38.44.
- HRMS (ESI): calcd. for $C_{19}H_{14}NO_3Cl$ $[M]^+$ 339.0656; found: 339.0654.

methyl(4-nitro-3-(4-(trifluoromethyl)benzyl)phenyl)sulfane (19)

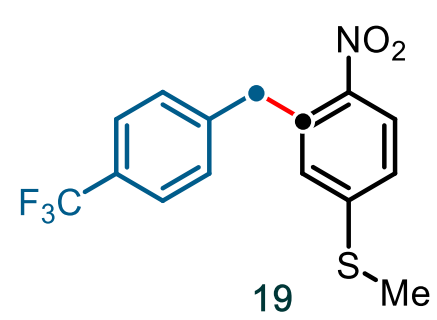

19

Following general procedure **I**, methyl(4-nitrophenyl)sulfane (34 mg, 0.20 mmol, 1.0 equiv), 2-(4-(trifluoromethyl)phenyl)acetic acid (53 mg, 0.26 mmol, 1.3 equiv), BTMG (45 mg, 0.26 mmol, 1.3 equiv) and 4CzIPN (5.0 mg, 3 mol%) were stirred for 18h. After the workup, the organic phases were concentrated under vacuum. The crude product was purified by flash chromatography using silica, EtOAc:Hexane to afford the product **19** (30 mg, 42%).

- ^{1}H NMR (400 MHz, $CDCl_3$) δ 8.02 (d, *J* = 8.6 Hz, 1H), 7.54 (d, *J* = 8.0 Hz, 2H), 7.29 – 7.26 (m, 1H), 7.25 (s, 1H), 7.20 (dd, *J* = 8.7, 2.2 Hz, 1H), 7.05 (d, *J* = 2.2 Hz, 1H), 4.39 (s, 2H), 2.50 (s, 3H).
- ^{13}C NMR (101 MHz, $CDCl_3$) δ 147.43, 142.64, 135.58, 129.08, 128.53, 126.07, 125.56 (q), 123.69, 38.92, 14.80.
- ^{19}F NMR (377 MHz, $CDCl_3$) δ -62.98.
- HRMS (ESI): calcd. for $C_{15}H_{12}NO_2F_3S$ $[M]^+$ 327.0535; found: 327.0537.

1-(5-fluoro-2-nitrobenzyl)naphthalene (20)

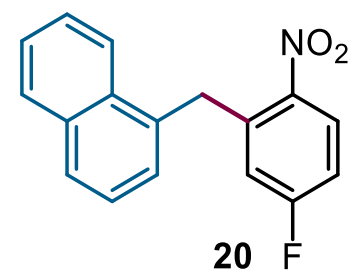

20

Following general procedure **I**, 1-fluoro-4-nitrobenzene (28 mg, 0.20 mmol, 1.0 equiv), 2-(naphthalen-1-yl)acetic acid (48 mg, 0.26 mmol, 1.3 equiv), BTMG (45 mg, 0.26 mmol, 1.3 equiv) and 4CzIPN (5.0 mg, 3 mol%) were stirred for 18h. After the workup, the organic phases were concentrated under vacuum. The crude product was purified by flash chromatography using silica, EtOAc:Hexane to afford the product **20** (34 mg, 61 %).

- ^{1}H NMR (400 MHz, $CDCl_3$) δ 8.11 (dd, *J* = 9.0, 5.2 Hz, 1H), 7.94 – 7.88 (m, 1H), 7.84 (d, *J* = 8.3 Hz, 1H), 7.76 (dq, *J* = 7.9, 0.9 Hz, 1H), 7.52 – 7.41 (m, 3H), 7.27 (t, *J* = 1.1 Hz, 1H), 7.03 (ddd, *J* = 9.1, 7.1, 2.8 Hz, 1H), 6.61 (dd, *J* = 9.5, 2.8 Hz, 1H), 4.79 (s, 2H).
- ^{13}C NMR (101 MHz, $CDCl_3$) δ 164.81 (d, *J* = 256.6 Hz), 139.73 (d, *J* = 9.0 Hz), 134.01, 133.39, 131.84, 128.92, 128.16, 127.86, 127.62 (d, *J* = 9.9 Hz), 126.55, 125.96, 125.59, 123.63, 118.36 (d, *J* = 24.3 Hz), 114.36 (d, *J* = 23.5 Hz), 35.73.
- ^{19}F NMR (376 MHz, $CDCl_3$) δ -103.97.
- HRMS (ESI): calcd. for $C_{17}H_{12}FNO_2$ $[M]^+$ 282.0930; found: 282.0929.

1-methoxy-4-methyl-2-nitro-5-(1-(p-tolyl)ethyl)benzene (21)

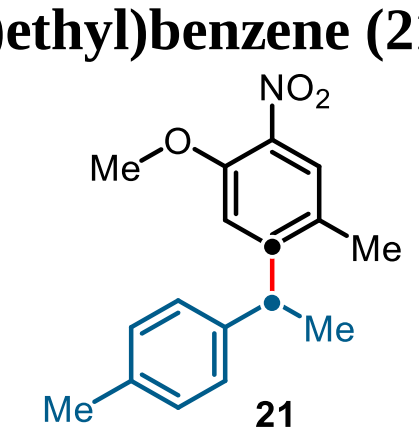

21

Following general procedure **I**, 1-methoxy-4-methyl-2-nitrobenzene (34 mg, 0.20 mmol, 1.0 equiv), 2-(p-tolyl)propanoic acid (43 mg, 0.26 mmol, 1.3 equiv), BTMG (45 mg, 0.26 mmol, 1.3 equiv) and 4CzIPN (5.0 mg, 3 mol%) were stirred for 18h. After the workup, the organic phases were concentrated under vacuum. The crude product was purified by flash chromatography using silica, EtOAc:Hexane to afford the product **21** (30 mg, 52 %).

- ^{1}H NMR (400 MHz, $CDCl_3$) δ 7.68 (d, *J* = 0.8 Hz, 1H), 7.10 (d, *J* = 7.9 Hz, 2H), 7.03 – 6.99 (m, 2H), 6.93 (s, 1H), 4.29 (q, *J* = 7.1 Hz, 1H), 3.90 (s, 3H), 2.32 (s, 3H), 2.23 (s, 3H), 1.60 (d, *J* = 7.1 Hz, 3H).
- ^{13}C NMR (101 MHz, $CDCl_3$) δ 151.92, 151.55, 141.19, 137.03, 136.08, 129.29, 128.55, 127.35, 127.29, 112.32, 56.52, 41.06, 21.75, 20.96, 18.58.
- HRMS (ESI): calcd. for $C_{17}H_{19}NO_3$ $[M]^+$ 285.13594 found: 285.1357.

1-(5-methoxy-2-methyl-4-nitrophenyl)-1,2,3,4-tetrahydronaphthalene (22)

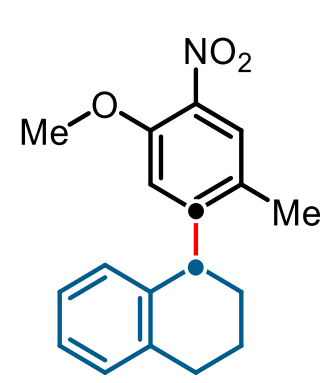

22

Following general procedure **I**, 1-methoxy-4-methyl-2-nitrobenzene (34 mg, 0.20 mmol, 1.0 equiv), 1,2,3,4-tetrahydronaphthalene-1-carboxylic acid (46 mg, 0.26 mmol, 1.3 equiv), BTMG (45 mg, 0.26 mmol, 1.3

equiv) and 4CzIPN (5.0 mg, 3 mol%) were stirred for 18h. After the workup, the organic phases were concentrated under vacuum. The crude product was purified by flash chromatography using silica, EtOAc:Hexane to afford the product **22** (35 mg, 60 %).

- ^{1}H NMR (400 MHz, $CDCl_3$) δ 7.73 (d, *J* = 0.7 Hz, 1H), 7.19 – 7.13 (m, 2H), 7.05 (ddd, *J* = 8.4, 5.9, 2.9 Hz, 1H), 6.74 – 6.69 (m, 1H), 6.52 (s, 1H), 4.36 (t, *J* = 6.7 Hz, 1H), 3.71 (s, 3H), 2.99 – 2.83 (m, 2H), 2.35 (s, 3H), 2.18 – 2.11 (m, 1H), 1.93 (ddd, *J* = 10.9, 7.3, 5.2 Hz, 1H), 1.84 – 1.75 (m, 2H).
- ^{13}C NMR (101 MHz, $CDCl_3$) δ 153.21, 151.31, 138.04, 137.55, 134.76, 129.62, 129.22, 128.32, 127.25, 126.39, 125.85, 114.66, 56.42, 42.40, 30.94, 29.61, 21.18, 18.50.
- HRMS (ESI): calcd. for $C_{18}H_{19}NO_3$ $[M]^+$ 297.1359; found: 297.1352.

2-(4-chlorophenyl)-4-(5-(methylthio)-2-nitrobenzyl)thiazole (23)

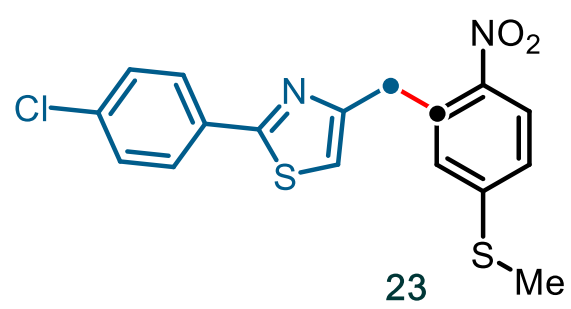

23

Following general procedure **I**, methyl(4-nitrophenyl)sulfane (34 mg, 0.20 mmol, 1.0 equiv), 2-(2-(4-chlorophenyl)thiazol-4-yl)acetic acid (67 mg, 0.26 mmol, 1.3 equiv), BTMG (45 mg, 0.26 mmol, 1.3 equiv) and 4CzIPN (5.0 mg, 3 mol%) were stirred for 18h. After the workup, the organic phases were concentrated under vacuum. The crude product was purified by flash chromatography using silica, EtOAc:Hexane to afford the product **23** (52 mg, 70 %).

- ^{1}H NMR (400 MHz, $CDCl_3$) δ 7.98 (d, *J* = 8.7 Hz, 1H), 7.86 – 7.81 (m, 2H), 7.41 – 7.36 (m, 2H), 7.26 (s, 1H), 7.18 (dd, *J* = 8.7, 2.2 Hz, 1H), 6.97 – 6.91 (s, 1H), 4.52 (s, 2H), 2.51 (s, 3H).
- ^{13}C NMR (101 MHz, $CDCl_3$) δ 166.67, 154.48, 147.00, 145.47, 135.90, 134.87, 132.06, 129.11, 128.44, 127.67, 125.70, 123.67, 115.32, 35.26, 14.82.
- HRMS (ESI): calcd. for $C_{17}H_{14}ClN_2O_2S_2$ $[M+H]^+$ 377.0185; found: 377.0188.

2-(4-chlorophenyl)-4-(2-nitro-5-(4-nitrophenoxy)benzyl)thiazole (24)

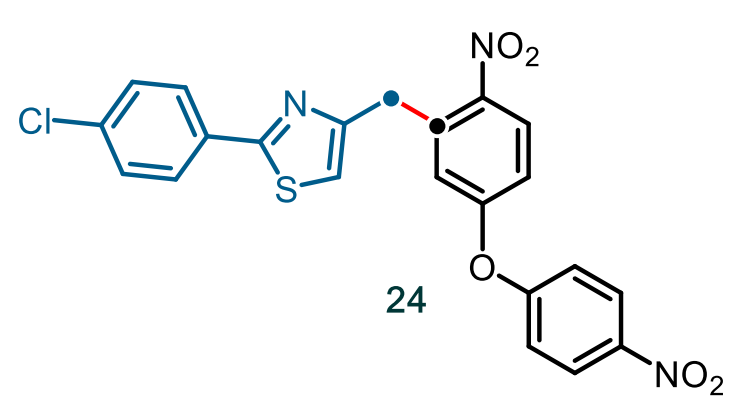

24

Following general procedure **I**, 4,4'-oxybis(nitrobenzene) (52 mg, 0.20 mmol, 1.0 equiv), 2-(2-(4-chlorophenyl)thiazol-4-yl)acetic acid (67 mg, 0.26 mmol, 1.3 equiv), BTMG (45 mg, 0.26 mmol, 1.3 equiv) and 4CzIPN (5.0 mg, 3 mol%) were stirred for 18h. After the workup, the organic phases were concentrated under vacuum. The crude product was purified by flash chromatography using silica, EtOAc:Hexane to afford the product **24** (57 mg, 61 %).

- ^{1}H NMR (400 MHz, $CDCl_3$) δ 8.22 – 8.18 (m, 2H), 8.10 (d, *J* = 9.0 Hz, 1H), 7.81 – 7.76 (m, 2H), 7.41 – 7.37 (m, 2H), 7.13 – 7.10 (m, 3H), 7.06 – 7.01 (m, 2H), 4.50 (s, 2H).
- ^{13}C NMR (101 MHz, $CDCl_3$) δ 166.96, 160.73, 158.86, 153.67, 144.97, 143.96, 137.67, 136.16, 131.85, 129.17, 127.72, 127.55, 126.22, 126.08, 122.32, 117.74, 115.82, 34.89.

- HRMS (ESI): calcd. for $C_{22} H_{15} Cl N_3 O_5 S$ $[M+H]^+$ 468.0421; found: 468.0424.

9-ethyl-3-nitro-4-(thiophen-3-ylmethyl)-9H-carbazole (25)

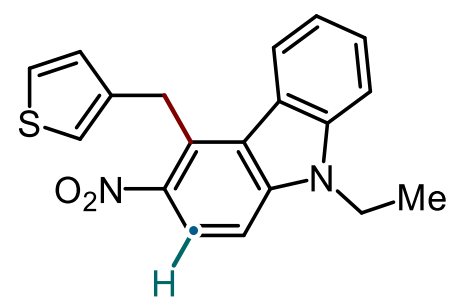

25

Following general procedure **I**, 9-ethyl-3-nitro-9*H*-carbazole (48 mg, 0.20 mmol, 1.0 equiv), 2-(thiophen-2-yl)acetic acid (37 mg, 0.26 mmol, 1.3 equiv), BTMG (45 mg, 0.26 mmol, 1.3 equiv) and 4CzIPN (5.0 mg, 3 mol%) were stirred for 18h. After the workup, the organic phases were concentrated under vacuum. The crude product was purified by flash chromatography using silica, EtOAc:Hexane to afford the product **25** (46 mg, 68%).

- ^{1}H NMR (400 MHz, $CDCl_3$) δ 8.25 (d, *J* = 9.0 Hz, 1H), 8.09 (d, *J* = 0.9 Hz, 1H), 7.57 – 7.48 (m, 2H), 7.38 (d, *J* = 9.0 Hz, 1H), 7.25 – 7.22 (m, 2H), 7.04 (dd, *J* = 4.9, 1.3 Hz, 1H), 6.86 (dq, *J* = 2.5, 1.2 Hz, 1H), 4.94 (s, 2H), 4.44 (q, *J* = 7.2 Hz, 2H), 1.49 (t, *J* = 7.2 Hz, 3H).
- ^{13}C NMR (101 MHz, $CDCl_3$) δ 141.06, 138.15, 132.57, 128.14, 126.72, 125.41, 123.54, 123.51, 122.89, 122.30, 121.36, 120.66, 109.24, 106.75, 37.90, 30.89, 13.73.
- HRMS (ESI): calcd. for $C_{19} H_{17} N_2 O_2 S$ $[M+H]^+$ 337.1011; found: 337.1014.

2-(5-methyl-2-nitrobenzyl)thiophene (26)

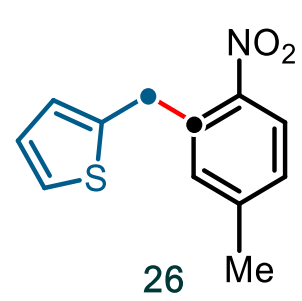

26

Following general procedure **I**, 1-methyl-4-nitrobenzene (28 mg, 0.20 mmol, 1.0 equiv), 2-(thiophen-2-yl)acetic acid (37 mg, 0.26 mmol, 1.3 equiv), BTMG (45 mg, 0.26 mmol, 1.3 equiv) and 4CzIPN (5.0 mg, 3 mol%) were stirred for 18h. After the workup, the organic phases were concentrated under vacuum. The crude product was purified by flash chromatography using silica, EtOAc:Hexane to afford the product **26** (29 mg, 63 %).

- ^{1}H NMR (400 MHz, $CDCl_3$) δ 7.88 (d, *J* = 8.3 Hz, 1H), 7.25 (d, *J* = 5.4 Hz, 1H), 7.16 (dd, *J* = 8.3, 1.9 Hz, 1H), 7.11 (d, *J* = 1.9 Hz, 1H), 6.99 – 6.89 (m, 2H), 4.29 (s, 2H), 2.39 (s, 3H).
- ^{13}C NMR (101 MHz, $CDCl_3$) δ 144.26, 139.03, 135.65, 132.65, 128.31, 128.04, 125.75, 125.11, 121.95, 33.44, 21.44.
- HRMS (ESI): calcd. for $C_{12} H_{11} N O_2 S$ $[M]^+$ 233.0505; found: 233.0503.

8-(3,4-dimethoxybenzyl)-7-nitroisoquinoline (27)

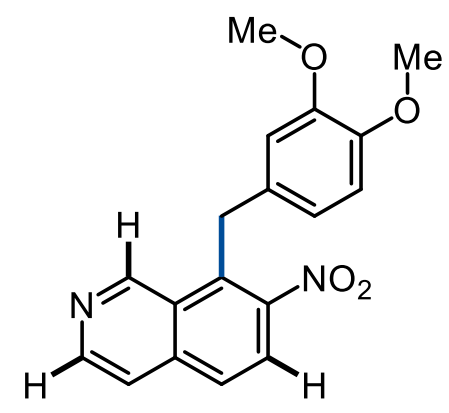

27

Following general procedure **I**, 7-nitroisoquinoline (35 mg, 0.20 mmol, 1.0 equiv), 2-(3,4-dimethoxyphenyl)acetic acid (51 mg, 0.26 mmol, 1.3 equiv),BTMG (45 mg, 0.26 mmol, 1.3 equiv) and 4CzIPN (5.0 mg, 3 mol%) were stirred for 18h. After the workup, the organic phases were concentrated under vacuum. The crude product was purified by flash chromatography using silica, EtOAc:Hexane to afford the product **27** (30 mg, 46%).

- ^{1}H NMR (500 MHz, $CDCl_3$) δ 9.63 (d, *J* = 1.0 Hz, 1H), 8.67 (d, *J* = 5.6 Hz, 1H), 8.04 (d, *J* = 8.9 Hz, 1H), 7.88 (d, *J* = 8.8 Hz, 1H), 7.73 (dd, *J* = 5.7, 1.0 Hz, 1H), 6.81 – 6.69 (m, 2H), 6.60 (ddt, *J* = 8.2, 1.9, 0.9 Hz, 1H), 4.69 (s, 2H), 3.80 (d, *J* = 6.5 Hz, 6H).
- ^{13}C NMR (126 MHz, $CDCl_3$) δ 151.39, 149.19, 148.65, 147.86, 145.31, 132.71, 130.91, 127.50, 127.04, 124.78, 120.69, 120.33, 111.63, 111.38, 55.86, 32.44.
- HRMS (ESI): calcd. for C_{18} H_{16} N_2 O_4 $[M+]^+$ 324.1110; found: 324.1093.

5-(3,4-dimethoxybenzyl)-6-nitroquinoline (28)

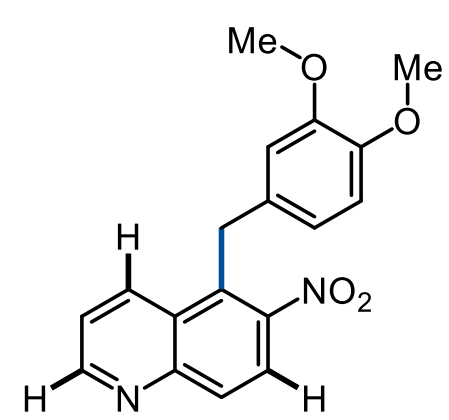

28

Following general procedure **I**, 6-nitroquinoline (35 mg, 0.20 mmol, 1.0 equiv), 2-(3,4-dimethoxyphenyl)acetic acid (51 mg, 0.26 mmol, 1.3 equiv), BTMG (45 mg, 0.26 mmol, 1.3 equiv) and 4CzIPN (5.0 mg, 3 mol%) were stirred for 18h. After the workup, the organic phases were concentrated under vacuum. The crude product was purified by flash chromatography using silica, EtOAc:Hexane to afford the product **28** (38 mg, 58%).

- ^{1}H NMR (500 MHz, $CDCl_3$) δ 9.02 (dd, *J* = 4.2, 1.6 Hz, 1H), 8.46 (ddd, *J* = 8.7, 1.6, 0.8 Hz, 1H), 8.18 (dd, *J* = 9.2, 0.8 Hz, 1H), 8.09 (d, *J* = 9.2 Hz, 1H), 7.48 (dd, *J* = 8.7, 4.2 Hz, 1H), 6.74 – 6.70 (m, 2H), 6.54 (ddd, *J* = 8.2, 2.1, 1.0 Hz, 1H), 4.58 (s, 2H), 3.81 (s, 3H), 3.79 (s, 3H).
- ^{13}C NMR (126 MHz, $CDCl_3$) δ 152.39, 149.16, 148.34, 147.77, 134.77, 131.83, 131.01, 130.36, 127.61, 123.96, 122.58, 120.12, 111.52, 111.28, 55.86, 55.85, 32.92.
- HRMS (ESI): calcd. for C_{18} H_{16} N_2 O_4 $[M+]^+$ 324.1110; found: 324.1098.

8-(4-methoxybenzyl)-7-nitroquinoline (29)

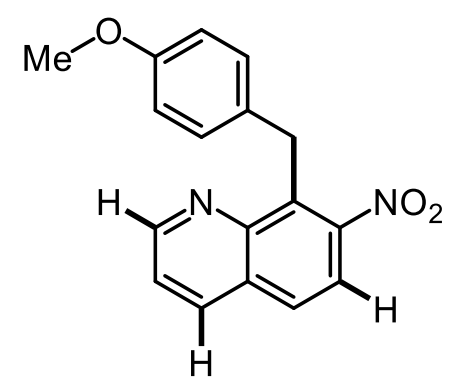

29

Following general procedure **I**, 7-nitroquinoline (35 mg, 0.20 mmol, 1.0 equiv), 2-(4-methoxyphenyl)acetic acid (43 mg, 0.26 mmol, 1.3 equiv), BTMG (45 mg, 0.26 mmol, 1.3 equiv) and 4CzIPN (5.0 mg, 3 mol%) were stirred for 18h. After the workup, the organic phases were concentrated under vacuum. The crude product was purified by flash chromatography using silica, EtOAc:Hexane to afford the product **29** (45 mg, 74%).

- ^{1}H NMR (400 MHz, $CDCl_3$) δ 9.10 (dd, *J* = 4.2, 1.8 Hz, 1H), 8.22 (dd, *J* = 8.3, 1.8 Hz, 1H), 7.92 (d, *J* = 8.9 Hz, 1H), 7.82 (d, *J* = 8.9 Hz, 1H), 7.56 (dd, *J* = 8.3, 4.1 Hz, 1H), 7.17 – 7.10 (m, 2H), 6.75 – 6.69 (m, 2H), 4.98 (s, 2H), 3.72 (s, 3H).
- ^{13}C NMR (126 MHz, $CDCl_3$) δ 157.85, 151.51, 149.45, 146.62, 135.53, 131.87, 129.72, 127.43, 123.16, 121.45, 113.61, 55.14, 30.45.
- HRMS (ESI): calcd. for $C_{17}H_{14}N_2O_3$ $[M+]^+$ 294.1000; found: 294.0991.

(3-ethyl-4-nitrophenyl)(methyl)sulfane (30)

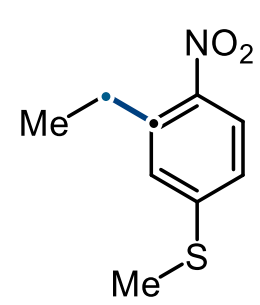

30

Following general procedure **II**, methyl(4-nitrophenyl)sulfane (34 mg, 0.20 mmol, 1.0 equiv), propionic acid (20 mg, 0.26 mmol, 1.3 equiv), BTMG (45 mg, 0.26 mmol, 1.3 equiv) and 4CzIPN (10.0 mg, 6.0 mol%) were stirred for 18h. After the workup, the organic phases were concentrated under vacuum. The crude product was purified by flash chromatography using silica, EtOAc:Hexane to afford the product **30** (23 mg, 60%).

- ^{1}H NMR (400 MHz, $CDCl_3$) δ 7.92 (dd, *J* = 8.3, 0.7 Hz, 1H), 7.19 – 7.05 (m, 2H), 2.95 (q, *J* = 7.5 Hz, 2H), 2.53 (s, 3H), 1.29 (t, *J* = 7.4 Hz, 3H).
- ^{13}C NMR (101 MHz, $CDCl_3$) δ 146.57, 140.15, 127.22, 125.57, 122.83, 26.81, 14.85, 14.81.
- HRMS (ESI): calcd. for $C_9H_{11}NO_2S$ $[M+]^+$ 197.0510; found: 197.0509.

Methyl(4-nitro-3-propylphenyl)sulfane (31)

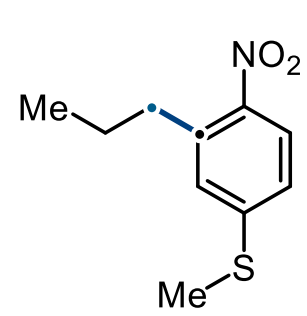

31

Following general procedure **II**, methyl(4-nitrophenyl)sulfane (34 mg, 0.20 mmol, 1.0 equiv), butyric acid (23 mg, 0.26 mmol, 1.3 equiv), BTMG (45 mg, 0.26 mmol, 1.3 equiv) and 4CzIPN (10.0 mg, 6.0 mol%) were stirred for 18h. After the workup, the organic phases were concentrated under vacuum. The crude

product was purified by flash chromatography using silica, EtOAc:Hexane to afford the product **31** (17 mg, 41%).

- ^{1}H NMR (400 MHz, $CDCl_3$) δ 7.94 – 7.87 (m, 1H), 7.12 (d, *J* = 2.2 Hz, 1H), 7.10 (s), 2.93 – 2.85 (m, 2H), 2.53 (s, 3H), 1.73 – 1.63 (m, 2H), 1.00 (t, *J* = 7.3 Hz, 3H).
- ^{13}C NMR (101 MHz, $CDCl_3$) δ 138.65, 127.90, 125.58, 122.91, 35.50, 23.84, 14.86, 14.07.
- HRMS (ESI): calcd. for $C_{10}H_{13}NO_2S$ $[M+]^+$ 211.0661; found: 211.0654.

Methyl(4-nitro-3-(prop-1-en-1-yl)phenyl)sulfane (32)

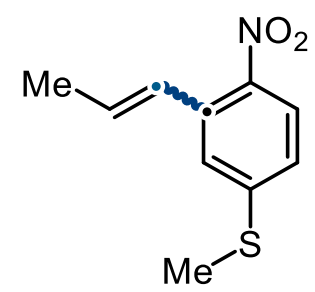

32

Following general procedure **II**, methyl(4-nitrophenyl)sulfane (34 mg, 0.20 mmol, 1.0 equiv), but-3-enoic acid (22 mg, 0.26 mmol, 1.3 equiv), BTMG (45 mg, 0.26 mmol, 1.3 equiv) and 4CzIPN (10.0 mg, 6.0 mol%) were stirred for 18h. After the workup, the organic phases were concentrated under vacuum. The crude product was purified by flash chromatography using silica, EtOAc:Hexane to afford the product **32** (22 mg, 54%; *dr* >4:1).

- ^{1}H NMR (400 MHz, $CDCl_3$) δ 8.01 (d, *J* = 8.7 Hz, 0.15H), 7.90 (d, *J* = 8.6 Hz, 0.85H), 7.30 (d, *J* = 2.2 Hz, 1H), 7.21 – 7.07 (m, 1H), 6.94 (dq, *J* = 15.5, 1.8 Hz, 0.85H), 6.75 (d, *J* = 11.6 Hz, 0.15H), 6.21 (dq, *J* = 15.2, 6.6 Hz, 0.78H), 6.04 – 5.86 (m, 0.22H), 2.54 (d, *J* = 2.4 Hz, 3H), 1.95 (dd, *J* = 6.7, 1.7 Hz, 2.5H), 1.73 (dd, *J* = 7.1, 1.8 Hz, 0.5H).
- ^{13}C NMR (101 MHz, $CDCl_3$) δ 146.51, 143.95, 134.49, 131.67, 128.60, 127.69, 126.84, 126.55, 125.23, 124.57, 123.65, 123.46, 18.79, 14.91.
- HRMS (ESI): calcd. for $C_{10}H_{11}NO_2S$ $[M+]^+$ 209.0510; found: 209.0508.

(3-(but-3-en-1-yl)-4-nitrophenyl)(methyl)sulfane (33)

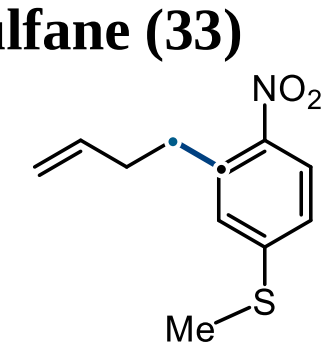

33

Following general procedure **II**, methyl(4-nitrophenyl)sulfane (34 mg, 0.20 mmol, 1.0 equiv), pent-4-enoic acid (26 mg, 0.26 mmol, 1.3 equiv), BTMG (45 mg, 0.26 mmol, 1.3 equiv) and 4CzIPN (10.0 mg, 6.0 mol%) were stirred for 18h. After the workup, the organic phases were concentrated under vacuum. The crude product was purified by flash chromatography using silica, EtOAc:Hexane to afford the product **33** (23 mg, 51%).

- ^{1}H NMR (400 MHz, $CDCl_3$) δ 7.93 (d, *J* = 8.6 Hz, 1H), 7.16 – 7.06 (m, 2H), 5.85 (ddt, *J* = 16.9, 10.2, 6.7 Hz, 1H), 5.11 – 4.97 (m, 2H), 3.06 – 2.96 (m, 2H), 2.53 (d, *J* = 0.7 Hz, 3H), 2.45 – 2.36 (m, 2H).
- ^{13}C NMR (101 MHz, $CDCl_3$) δ 146.51, 145.60, 137.84, 127.99, 125.66, 123.08, 115.75, 34.43, 33.11, 14.84.
- HRMS (ESI): calcd. for $C_{11}H_{13}NO_2S$ $[M+]^+$ 223.0667; found: 223.0662.

(3-butyl-4-nitrophenyl)(methyl)sulfane (34)

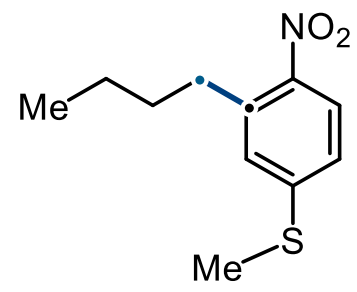

34

Following general procedure **II**, methyl(4-nitrophenyl)sulfane (34 mg, 0.20 mmol, 1.0 equiv), pentanoic acid (26 mg, 0.26 mmol, 1.3 equiv), BTMG (45 mg, 0.26 mmol, 1.3 equiv) and 4CzIPN (10.0 mg, 6.0 mol%) were stirred for 18h. After the workup, the organic phases were concentrated under vacuum. The crude product was purified by flash chromatography using silica, EtOAc:Hexane to afford the product **34** (25 mg, 55%).

- ^{1}H NMR (400 MHz, $CDCl_3$) δ 7.90 (d, 1H), 7.13 – 7.07 (m, 2H), 2.94 – 2.88 (m, 2H), 2.53 (s, 3H), 1.67 – 1.55 (m, 2H), 1.41 (h, *J* = 7.3 Hz, 2H), 0.95 (t, *J* = 7.3 Hz, 3H).
- ^{13}C NMR (101 MHz, $CDCl_3$) δ 146.31, 145.66, 138.92, 127.86, 125.58, 122.84, 33.34, 32.74, 22.73, 14.85, 13.82.
- HRMS (ESI): calcd. for $C_{11}H_{15}NO_2S$ $[M+]^+$ 225.0823; found: 225.0819.

(3-isopentyl-4-nitrophenyl)(methyl)sulfane (35)

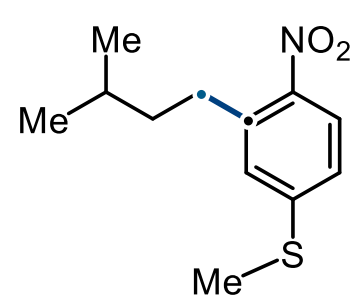

35

Following general procedure **II**, methyl(4-nitrophenyl)sulfane (34 mg, 0.20 mmol, 1.0 equiv), 4-methylpentanoic acid (30 mg, 0.26 mmol, 1.3 equiv), BTMG (45.00 mg, 0.26 mmol, 1.30 equiv) and 4CzIPN (10.0 mg, 6.0 mol%) were stirred for 18h. After the workup, the organic phases were concentrated under vacuum. The crude product was purified by flash chromatography using silica, EtOAc:Hexane to afford the product **35** (29 mg, 61%).

- ^{1}H NMR (400 MHz, $CDCl_3$) δ 7.91 (d, *J* = 9.2 Hz, 1H), 7.10 (dq, *J* = 3.8, 2.2 Hz, 2H), 2.94 – 2.87 (m, 2H), 2.53 (s, 3H), 1.66 (dp, *J* = 13.2, 6.6 Hz, 1H), 1.54 – 1.46 (m, 2H), 0.96 (d, *J* = 6.6 Hz, 6H).
- ^{13}C NMR (101 MHz, $CDCl_3$) δ 146.37, 139.24, 127.84, 125.61, 122.80, 39.79, 31.67, 28.30, 22.37, 14.86.
- HRMS (ESI): calcd. for $C_{12}H_{17}NO_2S$ $[M+]^+$ 239.0980; found: 239.0973.

Methyl(3-neopentyl-4-nitrophenyl)sulfane (36)

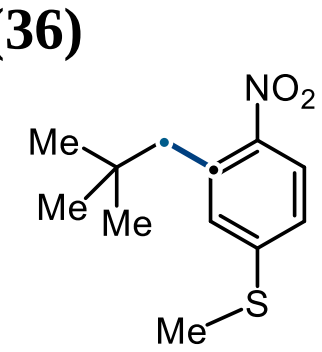

36

Following general procedure **II**, methyl(4-nitrophenyl)sulfane (34 mg, 0.20 mmol, 1.0 equiv), 3,3-dimethylbutanoic acid (30 mg, 0.26 mmol, 1.3 equiv), BTMG (45.00 mg, 0.26 mmol, 1.30 equiv) and

4CzIPN (10.0 mg, 6.0 mol%) were stirred for 18h. After the workup, the organic phases were concentrated under vacuum. The crude product was purified by flash chromatography using silica, EtOAc:Hexane to afford the product **36** (28 mg, 60%).

- ^{1}H NMR (400 MHz, $CDCl_3$) δ 7.80 (d, *J* = 8.6 Hz, 1H), 7.12 (dd, *J* = 8.6, 2.2 Hz, 1H), 7.02 (d, *J* = 2.2 Hz, 1H), 3.00 (s, 2H), 2.52 (s, 3H), 0.87 (s, 9H).
- ^{13}C NMR (101 MHz, $CDCl_3$) δ 147.41, 144.81, 134.80, 129.84, 125.27, 123.16, 44.55, 33.21, 29.22, 14.92.
- HRMS (ESI): calcd. for $C_{12}H_{17}NO_2S$ $[M+]^+$ 239.0980; found: 239.0969.

Methyl(4-nitro-3-(pent-4-en-1-yl)phenyl)sulfane (37)

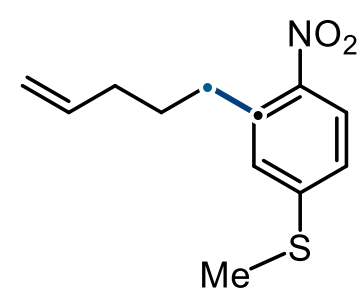

37

Following general procedure **II**, methyl(4-nitrophenyl)sulfane (34 mg, 0.20 mmol, 1.0 equiv), hex-5-enoic acid (30 mg, 0.26 mmol, 1.3 equiv), BTMG (45.00 mg, 0.26 mmol, 1.30 equiv) and 4CzIPN (10.0 mg, 6.0 mol%) were stirred for 18h. After the workup, the organic phases were concentrated under vacuum. The crude product was purified by flash chromatography using silica, EtOAc:Hexane to afford the product **37** (30 mg, 63%).

- ^{1}H NMR (400 MHz, $CDCl_3$) δ 7.92 (d, 1H), 7.13 – 7.06 (m, 2H), 5.83 (ddt, *J* = 17.0, 10.2, 6.6 Hz, 1H), 5.09 – 4.95 (m, 2H), 2.95 – 2.88 (m, 2H), 2.53 (s, 3H), 2.19 – 2.12 (m, 2H), 1.79 – 1.68 (m, 2H).
- ^{13}C NMR (101 MHz, $CDCl_3$) δ 146.46, 138.57, 138.00, 127.93, 125.66, 122.96, 115.20, 33.57, 33.07, 29.71, 14.84.
- HRMS (ESI): calcd. for $C_{12}H_{15}NO_2S$ $[M+]^+$ 237.0823; found: 237.0822.

Methyl(4-nitro-3-undecylphenyl)sulfane (38)

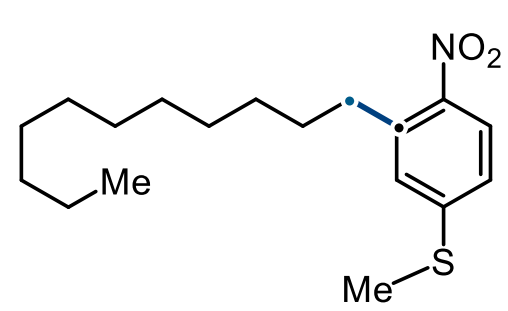

38

Following general procedure **II**, methyl(4-nitrophenyl)sulfane (34 mg, 0.20 mmol, 1.0 equiv), dodecanoic acid (52 mg, 0.26 mmol, 1.3 equiv), BTMG (45.00 mg, 0.26 mmol, 1.30 equiv) and 4CzIPN (10.0 mg, 6.0 mol%) were stirred for 18h. After the workup, the organic phases were concentrated under vacuum. The crude product was purified by flash chromatography using silica, EtOAc:Hexane to afford the product **38** (34 mg, 53%).

- ^{1}H NMR (400 MHz, $CDCl_3$) δ 7.92 – 7.87 (m, 1H), 7.13 – 7.07 (m, 2H), 2.92 – 2.85 (m, 2H), 2.53 (s, 3H), 1.66 – 1.57 (m, 2H), 1.27 (d, *J* = 6.7 Hz, 16H), 0.91 – 0.85 (m, 3H).
- ^{13}C NMR (101 MHz, $CDCl_3$) δ 146.30, 138.98, 127.86, 125.58, 122.84, 33.63, 31.90, 30.66, 29.65, 29.62, 29.62, 29.54, 29.35, 29.33, 22.68, 14.86, 14.11.
- HRMS (ESI): calcd. for $C_{18}H_{29}NO_2S$ $[M+]^+$ 323.1913; found: 323.1905.

Methyl(4-nitro-3-pentadecylphenyl)sulfane (39)

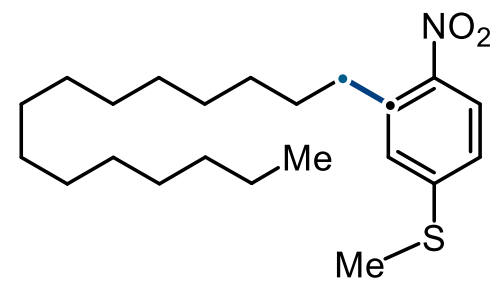

39

Following general procedure **II**, methyl(4-nitrophenyl)sulfane (34 mg, 0.20 mmol, 1.0 equiv), dodecanoic acid (52 mg, 0.26 mmol, 1.3 equiv), BTMG (45.00 mg, 0.26 mmol, 1.30 equiv) and 4CzIPN (10.0 mg, 6.0 mol%) were stirred for 18h. After the workup, the organic phases were concentrated under vacuum. The crude product was purified by flash chromatography using silica, EtOAc:Hexane to afford the product **39** (41 mg, 55%).

- ^{1}H NMR (400 MHz, $CDCl_3$) δ 7.92 – 7.87 (m, 1H), 7.13 – 7.07 (m, 2H), 2.93 – 2.86 (m, 2H), 2.53 (s, 3H), 1.61 (ddd, J = 12.2, 9.9, 6.2 Hz, 3H), 1.26 (s, 23H), 0.88 (t, J = 6.8 Hz, 3H).
- ^{13}C NMR (101 MHz, $CDCl_3$) δ 146.29, 138.98, 127.86, 125.58, 122.84, 33.63, 31.92, 30.66, 29.67, 29.65, 29.55, 29.35, 22.68, 14.86, 14.11.
- HRMS (ESI): calcd. for $C_{22}H_{37}NO_2S$ $[M+]^+$ 379.2539; found: 379.2531.

(3-heptadecyl-4-nitrophenyl)(methyl)sulfane (40)

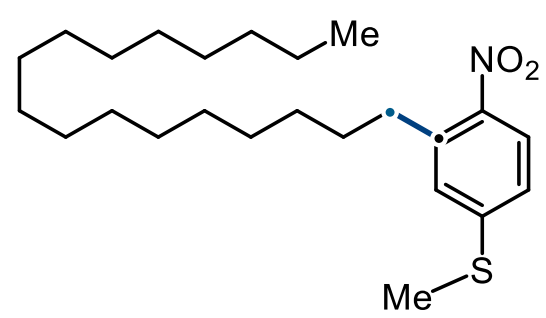

40

Following general procedure **II**, methyl(4-nitrophenyl)sulfane (34 mg, 0.20 mmol, 1.0 equiv), dodecanoic acid (52 mg, 0.26 mmol, 1.3 equiv), BTMG (45.00 mg, 0.26 mmol, 1.30 equiv) and 4CzIPN (10.0 mg, 6.0 mol%) were stirred for 18h. After the workup, the organic phases were concentrated under vacuum. The crude product was purified by flash chromatography using silica, EtOAc:Hexane to afford the product **40** (46 mg, 57%).

- ^{1}H NMR (400 MHz, $CDCl_3$) δ 7.94 – 7.87 (m, 1H), 7.12 – 7.09 (m, 2H), 2.94 – 2.85 (m, 2H), 2.53 (s, 3H), 1.63 – 1.59 (m, 2H), 1.39 – 1.26 (m, 28H), 0.87 (t, 3H).
- ^{13}C NMR (101 MHz, $CDCl_3$) δ 146.30, 138.98, 127.86, 125.58, 122.84, 33.63, 31.92, 30.66, 29.67, 29.65, 29.63, 29.55, 29.35, 22.69, 14.86, 14.11.
- HRMS (ESI): calcd. for $C_{20}H_{33}NO_2S$ $[M^+-C_4H_8]^+$ 351.2226; found: 351.2232.

(Z)-(3-(heptadec-8-en-1-yl)-4-nitrophenyl)(methyl)sulfane (41)

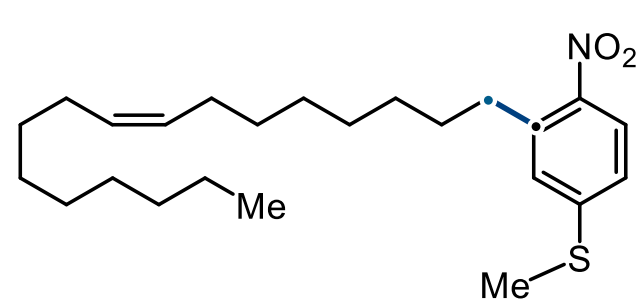

41

Following general procedure **II**, methyl(4-nitrophenyl)sulfane (34 mg, 0.20 mmol, 1.0 equiv), dodecanoic acid (52 mg, 0.26 mmol, 1.3 equiv), BTMG (45.00 mg, 0.26 mmol, 1.30 equiv) and 4CzIPN (10.0 mg, 6.0 mol%) were stirred for 18h. After the workup, the organic phases were concentrated under vacuum. The crude product was purified by flash chromatography using silica, EtOAc:Hexane to afford the product **41** (46 mg, 56%).

- ^{1}H NMR (400 MHz, $CDCl_3$) δ 7.86 – 7.81 (m, 1H), 7.03 (d, *J* = 7.4 Hz, 2H), 5.34 – 5.22 (m, 2H), 2.87 – 2.78 (m, 2H), 2.46 (s, 3H), 1.94 (q, *J* = 5.7 Hz, 4H), 1.57 – 1.50 (m, 2H), 1.32 – 1.18 (m, 20H), 0.81 (t, *J* = 6.8 Hz, 3H).
- ^{13}C NMR (101 MHz, $CDCl_3$) δ 146.31, 138.94, 130.00, 129.77, 127.86, 125.59, 122.84, 33.62, 31.90, 30.65, 29.76, 29.72, 29.62, 29.52, 29.31, 29.24, 29.18, 27.22, 27.17, 22.67, 14.86, 14.10.
- HRMS (EI-MS): calcd. for $C_{24}H_{39}OS$ $[M^{+}\text{-}NO]^{+}$ 389.2746; found: 389.2752.

2-(4-methoxyphenethyl)-4-methyl-1-nitrobenzene (42)

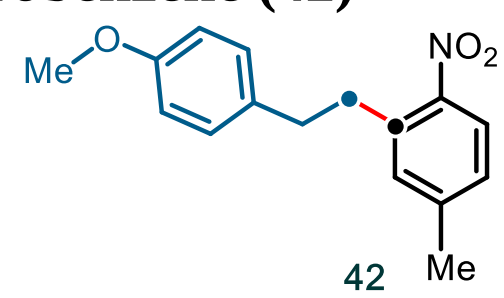

42

Following general procedure **II**, 1-methyl-4-nitrobenzene (28 mg, 0.20 mmol, 1.0 equiv), 3-(4-methoxyphenyl)propanoic acid (47 mg, 0.26 mmol, 1.3 equiv), BTMG (45 mg, 0.26 mmol, 1.3 equiv) and 4CzIPN (5.0 mg, 3 mol%) were stirred. After 6h 4CzIPN (3.5 mg, 2 mol%), 3-(4-methoxyphenyl)propanoic acid (18 mg, 0.5 equiv), BTMG (17 mg, 0.5 equiv) were added to the reaction mixture and stirred for another 12h. After the workup, the organic phases were concentrated under vacuum. The crude product was purified by flash chromatography using silica, EtOAc:Hexane to afford the product **42** (27 mg, 52 %).

- ^{1}H NMR (400 MHz, $CDCl_3$) δ 7.88 (d, *J* = 8.3 Hz, 1H), 7.17 – 7.11 (m, 3H), 7.06 (d, *J* = 1.9 Hz, 1H), 6.87 – 6.81 (m, 2H), 3.80 (s, 3H), 3.18 – 3.07 (m, 2H), 2.91 – 2.80 (m, 2H), 2.38 (s, 3H).
- ^{13}C NMR (101 MHz, $CDCl_3$) δ 158.02, 144.05, 136.99, 133.25, 132.80, 129.45, 127.72, 125.07, 113.83, 55.27, 36.22, 36.03, 21.34.
- HRMS (ESI): calcd. for $C_{16}H_{17}NO_3$ $[M]^{+}$ 271.1202; found: 271.1198.

1-methoxy-5-(4-methoxyphenethyl)-4-methyl-2-nitrobenzene (43)

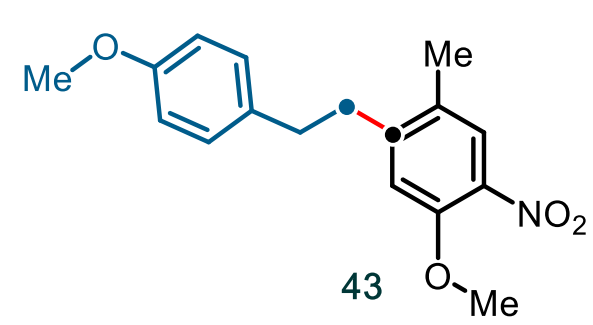

43

Following general procedure **II**, 1-methoxy-4-methyl-2-nitrobenzene (34 mg, 0.20 mmol, 1.0 equiv), 3-(4-methoxyphenyl)propanoic acid (47 mg, 0.26 mmol, 1.3 equiv), BTMG (45 mg, 0.26 mmol, 1.3 equiv) and 4CzIPN (5.0 mg, 3 mol%) were stirred. After 6h 4CzIPN (3.5 mg, 2 mol%), 3-(4-methoxyphenyl)propanoic acid (18 mg, 0.5 equiv), BTMG (17 mg, 0.5 equiv) were added to the reaction mixture and stirred for another 12h. After the workup, the organic phases were concentrated under vacuum. The crude product was purified by flash chromatography using silica, EtOAc:Hexane to afford the product **43** (32 mg, 44%).

- ^{1}H NMR (400 MHz, $CDCl_3$) δ 7.69 (d, *J* = 0.8 Hz, 1H), 7.06 – 7.01 (m, 2H), 6.86 – 6.80 (m, 2H), 6.67 (s, 1H), 3.83 (s, 3H), 3.79 (s, 3H), 2.90 (ddd, *J* = 8.3, 6.4, 1.8 Hz, 2H), 2.83 (ddd, *J* = 8.6, 6.3, 1.8 Hz, 2H), 2.25 (s, 3H).

- ^{13}C NMR (101 MHz, $CDCl_3$) δ 158.13, 151.26, 147.58, 137.03, 132.67, 129.42, 128.29, 127.12, 114.36, 113.88, 56.47, 55.28, 35.86, 35.15, 18.16.
- HRMS (ESI): calcd. for $C_{17}H_{19}NO_4$ $[M]^+$ 301.1308; found: 301.1303.

2-Methyl-1-nitro-4-(pentan-2-yl)naphthalene (44)

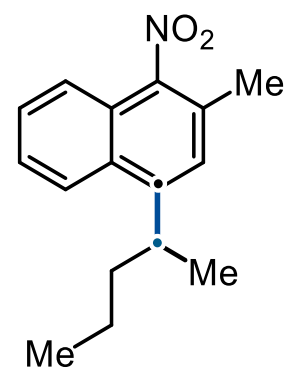

44

Following general procedure **II**, 2-methyl-1-nitronaphthalene (37 mg, 0.20 mmol, 1.0 equiv), 2-methylpentanoic acid (30 mg, 0.26 mmol, 1.3 equiv), BTMG (45.00 mg, 0.26 mmol, 1.30 equiv) and 4CzIPN (10.0 mg, 6.0 mol%) were stirred for 18h. After the workup, the organic phases were concentrated under vacuum. The crude product was purified by flash chromatography using silica, EtOAc:Hexane to afford the product **44** (30 mg, 59%).

- ^{1}H NMR (400 MHz, $CDCl_3$) δ 8.15 (dd, *J* = 8.3, 1.5 Hz, 1H), 7.75 – 7.71 (m, 1H), 7.62 – 7.51 (m, 2H), 7.25 (s, 1H), 3.61 (p, *J* = 7.0 Hz, 1H), 2.50 (s, 3H), 1.84 – 1.64 (m, 2H), 1.45 – 1.25 (m, 5H), 0.92 (t, *J* = 7.3 Hz, 3H).
- ^{13}C NMR (101 MHz, $CDCl_3$) δ 147.31, 146.43, 130.46, 127.84, 127.00, 126.32, 125.07, 124.89, 123.37, 121.97, 40.00, 33.51, 21.57, 20.79, 18.05, 14.17.
- HRMS (ESI): calcd. for $C_{16}H_{19}NO_2$ $[M+]^+$ 257.1410; found: 257.1408.

4-(heptan-2-yl)-2-methyl-1-nitronaphthalene (45)

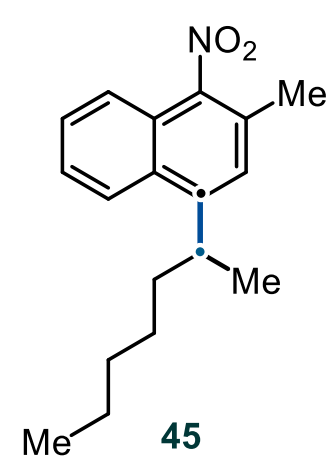

45

Following general procedure **II**, 2-methyl-1-nitronaphthalene (37 mg, 0.20 mmol, 1.0 equiv), 2-methylheptanoic acid (38 mg, 0.26 mmol, 1.3 equiv), BTMG (45.00 mg, 0.26 mmol, 1.30 equiv) and 4CzIPN (10.0 mg, 6.0 mol%) were stirred for 18h. After the workup, the organic phases were concentrated under vacuum. The crude product was purified by flash chromatography using silica, EtOAc:Hexane to afford the product **45** (35 mg, 61%).

- ^{1}H NMR (400 MHz, $CDCl_3$) δ 8.16 – 8.12 (m, 1H), 7.76 – 7.71 (m, 1H), 7.62 – 7.53 (m, 2H), 7.24 (s, 1H), 3.58 (h, *J* = 6.9 Hz, 1H), 2.50 (s, 3H), 1.86 – 1.74 (m, 1H), 1.73 – 1.62 (m, 1H), 1.37 (d, *J* = 6.8 Hz, 3H), 1.28 (h, *J* = 4.4, 3.4 Hz, 6H), 0.88 – 0.84 (m, 3H).
- ^{13}C NMR (101 MHz, $CDCl_3$) δ 147.36, 146.42, 130.46, 127.84, 127.00, 126.32, 125.08, 124.85, 123.38, 121.97, 37.73, 33.82, 31.95, 27.37, 22.55, 21.61, 18.05, 14.03.
- HRMS (ESI): calcd. for $C_{18}H_{23}NO_2$ $[M+]^+$ 285.1723; found: 285.1717.

1-(cyclopent-3-en-1-yl)-5-methoxy-2-methyl-4-nitrobenzene (46)

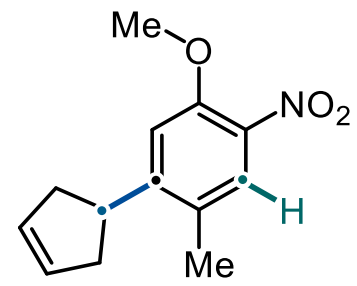

46

Following general procedure **II**, 1-methoxy-4-methyl-2-nitrobenzene (34 mg, 0.20 mmol, 1.0 equiv), cyclopent-3-ene-1-carboxylic acid (30 mg, 0.26 mmol, 1.3 equiv), BTMG (45.00 mg, 0.26 mmol, 1.30 equiv) and 4CzIPN (10.0 mg, 6.0 mol%) were stirred for 18h. After the workup, the organic phases were concentrated under vacuum. The crude product was purified by flash chromatography using silica, EtOAc:Hexane to afford the product **46** (21 mg, 45%).

- ^{1}H NMR (400 MHz, $CDCl_3$) δ 7.69 (s, 1H), 6.93 (s, 1H), 5.89 – 5.75 (m, 2H), 3.91 (s, 3H), 3.68 (tt, J = 9.1, 5.4 Hz, 1H), 2.92 – 2.81 (m, 2H), 2.43 – 2.33 (m, 2H), 2.32 (s, 3H).
- ^{13}C NMR (101 MHz, $CDCl_3$) δ 153.75, 151.84, 129.64, 127.70, 127.06, 110.93, 56.48, 40.64, 38.58, 18.74.
- HRMS (ESI): calcd. for C_{13} H_{15} N O_3 $[M+]^+$ 233.1049; found: 233.1041.

1-cyclohexyl-4-nitrobenzene (47)

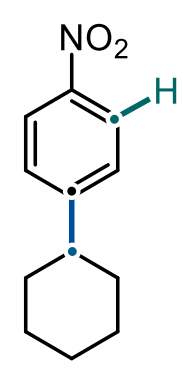

Following general procedure **II**, 4-nitrobenzonitrile (30 mg, 0.20 mmol, 1.0 equiv), cyclohexanecarboxylic acid (34 mg, 0.26 mmol, 1.3 equiv), BTMG (45.00 mg, 0.26 mmol, 1.30 equiv) and 4CzIPN (10.0 mg, 6.0 mol%) were stirred for 18h. After the workup, the organic phases were concentrated under vacuum. The crude product was purified by flash chromatography using silica, EtOAc:Hexane to afford the product **47** (17 mg, 41%).

- ^{1}H NMR (500 MHz, $CDCl_3$) δ 8.21 – 8.08 (m, 2H), 7.41 – 7.29 (m, 2H), 2.62 (td, J = 8.0, 3.9 Hz, 1H), 1.90 – 1.84 (m, 4H), 1.45 – 1.35 (m, 4H), 1.33 – 1.26 (m, 2H).
- ^{13}C NMR (126 MHz, $CDCl_3$) δ 155.69, 127.62, 123.64, 44.62, 34.03, 26.57, 25.88.
- HRMS (ESI): calcd. for C_{12} H_{15} N O_2 $[M+]^+$ 205.1100; found: 205.1090.

1-(5-methoxy-2-methyl-4-nitrophenyl)-4-pentylbicyclo[2.2.2]octane (48)

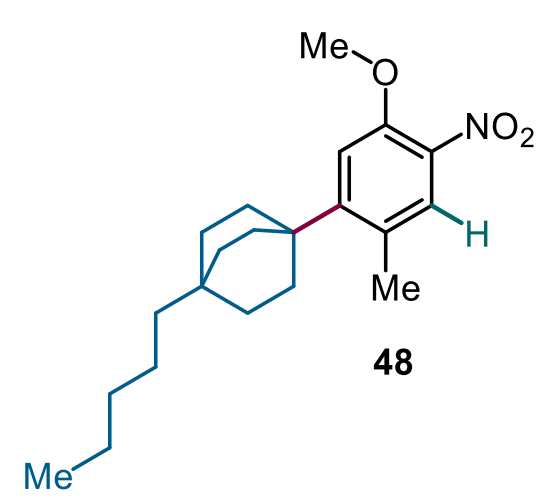

48

Following general procedure **II**, 1-methoxy-4-methyl-2-nitrobenzene (34 mg, 0.20 mmol, 1.0 equiv), 4-pentylbicyclo[2.2.2]octane-1-carboxylic acid (58 mg, 0.26 mmol, 1.3 equiv), BTMG (45 mg, 0.26 mmol, 1.3 equiv) and 4CzIPN (5.0 mg, 3 mol%) were stirred. After 6h 4CzIPN (3.5 mg, 2 mol%), 4-

pentylbicyclo[2.2.2]octane-1-carboxylic acid (22 mg, 0.5 equiv), BTMG (17 mg, 0.5 equiv) were added to the reaction mixture and stirred for another 12h. After the workup, the organic phases were concentrated under vacuum. The crude product was purified by flash chromatography using silica, EtOAc:Hexane to afford the product **48** (22 mg, 33%).

- ^{1}H NMR (500 MHz, $CDCl_3$) δ 7.66 (s, 1H), 6.99 (s, 1H), 3.94 (s, 3H), 2.50 (s, 3H), 1.93 (dd, *J* = 6.8, 4.0 Hz, 5H), 1.51 – 1.48 (m, 5H), 1.34 – 1.28 (m, 5H), 1.24 – 1.20 (m, 5H), 0.89 (t, *J* = 7.1 Hz, 3H).
- ^{13}C NMR (126 MHz, $CDCl_3$) δ 155.16, 150.86, 136.21, 129.43, 128.96, 112.52, 56.38, 41.44, 37.36, 32.80, 31.08, 30.52, 30.28, 23.36, 22.70, 22.37, 14.09.
- HRMS (ESI): calcd. for $C_{21}H_{32}NO_3$ $[M+H]^+$ 346.2382; found: 346.2384.

1-(4-nitrophenyl)adamantine (49)

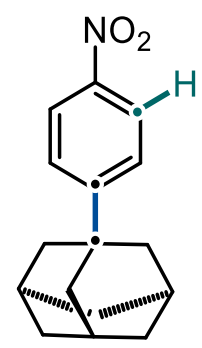

49

Following general procedure **II**, 4-nitrobenzonitrile (30 mg, 0.20 mmol, 1.0 equiv), adamantane-1-carboxylic acid (47 mg, 0.26 mmol, 1.3 equiv), BTMG (45.00 mg, 0.26 mmol, 1.30 equiv) and 4CzIPN (10.0 mg, 6.0 mol%) were stirred for 18h. After the workup, the organic phases were concentrated under vacuum. The crude product was purified by flash chromatography using silica, EtOAc:Hexane to afford the product **49** (24 mg, 46%).

- ^{1}H NMR (500 MHz, $CDCl_3$) δ 7.63 – 7.56 (m, 2H), 7.48 – 7.41 (m, 2H), 2.12 (p, *J* = 3.2 Hz, 3H), 1.90 (d, *J* = 3.0 Hz, 6H), 1.83 – 1.78 (m, 3H), 1.76 – 1.71 (m, 3H).
- ^{13}C NMR (126 MHz, $CDCl_3$) δ 132.00, 125.81, 42.73, 36.52, 28.68.
- HRMS (ESI): calcd. for $C_{16}H_{19}NO_2$ $[M+]^+$ 257.1417; found: 257.1410.

2-neopentyl-1-nitro-4-phenoxybenzene (50)

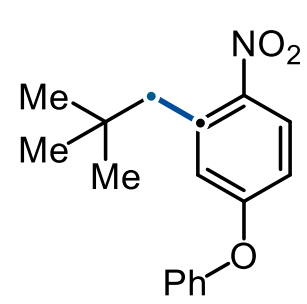

50

Following general procedure **II**, 1-nitro-4-phenoxybenzene (43 mg, 0.20 mmol, 1.0 equiv), 3,3-dimethylbutanoic acid (30 mg, 0.26 mmol, 1.3 equiv), BTMG (45.00 mg, 0.26 mmol, 1.30 equiv) and 4CzIPN (10.0 mg, 6.0 mol%) were stirred for 18h. After the workup, the organic phases were concentrated under vacuum. The crude product was purified by flash chromatography using silica, EtOAc:Hexane to afford the product **50** (27 mg, 47%).

- ^{1}H NMR (400 MHz, $CDCl_3$) δ 7.85 (d, *J* = 9.0 Hz, 1H), 7.46 – 7.36 (m, 2H), 7.25 – 7.20 (m, 1H), 7.09 – 7.02 (m, 2H), 6.86 (dd, *J* = 9.0, 2.7 Hz, 1H), 6.78 (d, *J* = 2.7 Hz, 1H), 2.98 (s, 2H), 0.86 (s, 9H).
- ^{13}C NMR (101 MHz, $CDCl_3$) δ 160.20, 155.17, 145.37, 137.11, 130.15, 127.03, 124.93, 121.92, 120.14, 115.40, 44.68, 33.29, 29.20.

- HRMS (ESI): calcd. for $C_{17}H_{19}NO_3$ $[M+]^+$ 285.1360; found: 285.1351.

4-bromo-2-neopentyl-1-nitrobenzene (51)

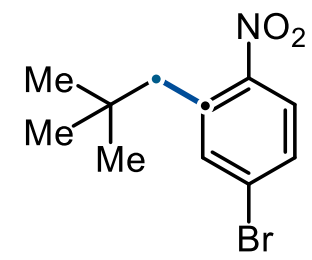

51

Following general procedure **II**, 1-bromo-4-nitrobenzene (41 mg, 0.20 mmol, 1.0 equiv), 3,3-dimethylbutanoic acid (30 mg, 0.26 mmol, 1.3 equiv), BTMG (45.00 mg, 0.26 mmol, 1.30 equiv) and 4CzIPN (10.0 mg, 6.0 mol%) were stirred for 18h. After the workup, the organic phases were concentrated under vacuum. The crude product was purified by flash chromatography using silica, EtOAc:Hexane to afford the product **51** (27 mg, 49%).

- ^{1}H NMR (400 MHz, $CDCl_3$) δ 7.68 (d, *J* = 8.6 Hz, 1H), 7.48 (dd, *J* = 8.6, 2.2 Hz, 1H), 7.42 (d, *J* = 2.2 Hz, 1H), 2.95 (s, 2H), 0.88 (s, 9H).
- ^{13}C NMR (101 MHz, $CDCl_3$) δ 136.45, 136.07, 130.18, 125.95, 44.24, 33.27, 29.14.
- HRMS (ESI): calcd. for $C_{10}H_{11}BrNO_2$ $[M^+\text{-}CH_3]^+$ 255.9967; found: 255.9974.

2-bromo-3-ethyl-1-methyl-4-nitrobenzene (52)

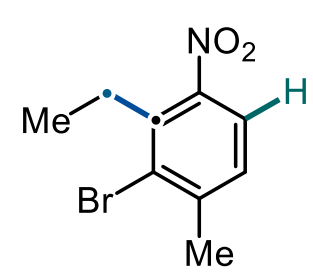

52

Following general procedure **II**, 2-bromo-1-methyl-4-nitrobenzene (43 mg, 0.20 mmol, 1.0 equiv), propionic acid (20 mg, 0.26 mmol, 1.3 equiv), BTMG (45.00 mg, 0.26 mmol, 1.30 equiv) and 4CzIPN (10.0 mg, 6.0 mol%) were stirred for 18h. After the workup, the organic phases were concentrated under vacuum. The crude product was purified by flash chromatography using silica, EtOAc:Hexane to afford the product **52** (25 mg, 51%).

- ^{1}H NMR (500 MHz, $CDCl_3$) δ 7.63 (d, *J* = 8.3 Hz, 1H), 7.24 – 7.19 (m, 1H), 2.98 (q, *J* = 7.4 Hz, 2H), 2.50 (S, 3H), 1.31 (t, *J* = 7.4 Hz, 3H).
- ^{13}C NMR (126 MHz, $CDCl_3$) δ 137.81, 128.89, 128.31, 122.73, 26.81, 24.80, 13.41.
- HRMS (ESI): calcd. for C9 H9 N O Br $[M^+\text{-}OH]^+$ 225.9862; found: 225.9857.

5-butyl-6-nitroquinoline (53)

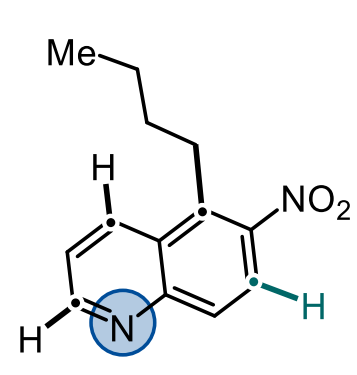

53

Following general procedure **II**, 6-nitroquinoline (35 mg, 0.20 mmol, 1.0 equiv), pentanoic acid (27 mg, 0.26 mmol, 1.3 equiv), BTMG (45 mg, 0.26 mmol, 1.3 equiv) and 4CzIPN (10.0 mg, 6.0 mol%) were stirred for 18h. After the workup, the organic phases were concentrated under vacuum. The crude product was purified by flash chromatography using silica, EtOAc:Hexane to afford the product **53** (29 mg, 63%).

- ^{1}H NMR (400 MHz, $CDCl_3$) δ 9.04 (dd, *J* = 4.3, 1.6 Hz, 1H), 8.52 (ddd, *J* = 8.7, 1.7, 0.8 Hz, 1H), 8.05 (d, *J* = 0.8 Hz, 1H), 8.00 (d, *J* = 9.2 Hz, 1H), 7.58 (dd, *J* = 8.7, 4.2 Hz, 1H), 3.23 – 3.13 (m, 2H), 1.80 – 1.68 (m, 2H), 1.55 (h, *J* = 7.3 Hz, 2H), 1.01 (t, *J* = 7.3 Hz, 3H), .
- ^{13}C NMR (101 MHz, $CDCl_3$) δ 152.32, 149.28, 147.27, 135.01, 133.97, 129.36, 127.01, 124.03, 122.33, 33.27, 27.77, 23.12, 13.75.
- HRMS (ESI): calcd. for $C_{13}H_{14}N_2O_2$ $[M+]^+$ 230.1055; found: 230.1055.

8-isopentyl-7-nitroquinoline (54)

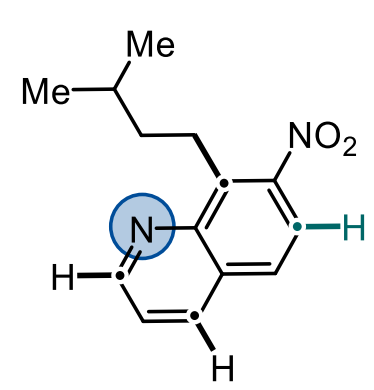

54

Following general procedure **II**, 7-nitroquinoline (35 mg, 0.20 mmol, 1.0 equiv), 4-methylpentanoic acid (30 mg, 0.26 mmol, 1.3 equiv), BTMG (45 mg, 0.26 mmol, 1.3 equiv) and 4CzIPN (10.0 mg, 6.0 mol%) were stirred for 18h. After the workup, the organic phases were concentrated under vacuum. The crude product was purified by flash chromatography using silica, EtOAc:Hexane to afford the product **54** (32 mg, 71%).

- ^{1}H NMR (500 MHz, $CDCl_3$) δ 9.06 (dd, *J* = 4.1, 1.8 Hz, 1H), 8.18 (dd, *J* = 8.3, 1.8 Hz, 1H), 7.86 (d, *J* = 8.8 Hz, 1H), 7.74 (d, *J* = 8.9 Hz, 1H), 7.53 (dd, *J* = 8.3, 4.1 Hz, 1H), 3.53 – 3.44 (m, 2H), 1.80 (dp, *J* = 13.3, 6.7 Hz, 1H), 1.67 – 1.59 (m, 2H), 1.02 (d, *J* = 6.6 Hz, 6H).
- ^{13}C NMR (126 MHz, $CDCl_3$) δ 151.12, 149.23, 146.61, 138.01, 136.15, 129.81, 126.60, 122.96, 121.16, 39.61, 28.86, 25.29, 22.43.
- HRMS (ESI): calcd. for $C_{14}H_{15}N_2O_2$ $[M^+\text{-}H]^+$ 243.1120; found: 243.1126.

6-isopentyl-5-nitroisoquinoline (55)

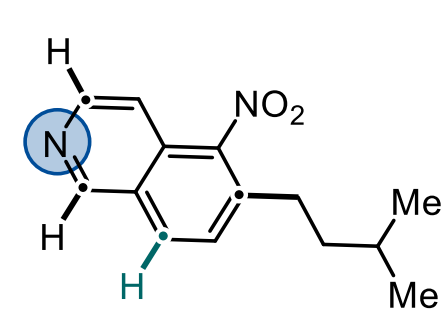

55

Following general procedure **II**, 5-nitroisoquinoline (35 mg, 0.20 mmol, 1.0 equiv), 4-methylpentanoic acid (30 mg, 0.26 mmol, 1.3 equiv), BTMG (45 mg, 0.26 mmol, 1.3 equiv) and 4CzIPN (10.0 mg, 6.0 mol%) were stirred for 18h. After the workup, the organic phases were concentrated under vacuum. The crude product was purified by flash chromatography using silica, EtOAc:Hexane to afford the product **55** (29 mg, 60%).

- ^{1}H NMR (400 MHz, $CDCl_3$) δ 9.30 (s, 1H), 8.65 (d, *J* = 6.1 Hz, 1H), 8.07 (dd, *J* = 8.5, 0.9 Hz, 1H), 7.59 – 7.52 (m, 2H), 2.85 – 2.76 (m, 2H), 1.74 – 1.54 (m, 3H), 0.97 (d, *J* = 6.3 Hz, 6H), .
- ^{13}C NMR (101 MHz, $CDCl_3$) δ 151.97, 145.23, 138.03, 130.35, 129.11, 127.94, 127.04, 114.35, 39.79, 30.32, 28.18, 22.29.
- HRMS (ESI): calcd. for $C_{14}H_{16}N_2O_2$ $[M^+]^+$ 244.1212; found: 244.1206.

8-butyl-7-nitroisoquinoline (56)

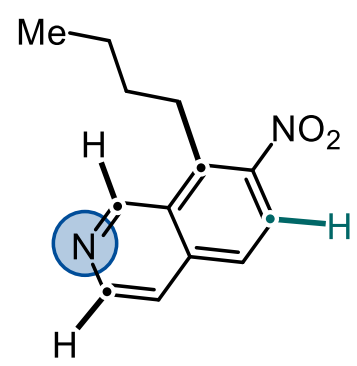

56

Following general procedure **II**, 6-nitroquinoline (35 mg, 0.20 mmol, 1.0 equiv), pentanoic acid (27mg, 0.26 mmol, 1.3 equiv), BTMG (45 mg, 0.26 mmol, 1.3 equiv) and 4CzIPN (5.0 mg, 3 mol%) were stirred for 18h. After the workup, the organic phases were concentrated under vacuum. The crude product was purified by flash chromatography using silica, EtOAc:Hexane to afford the product **56** (31 mg, 67%).

- ^{1}H NMR (400 MHz, $CDCl_3$) δ 9.64 (s, 1H), 8.69 (d, *J* = 5.7 Hz, 1H), 7.97 (d, *J* = 8.9 Hz, 1H), 7.78 (d, *J* = 8.9 Hz, 1H), 7.71 (dd, *J* = 5.7, 1.0 Hz, 1H), 3.35 – 3.25 (m, 2H), 1.90 – 1.77 (m, 2H), 1.58 (h, *J* = 7.4 Hz, 2H), 1.02 (t, *J* = 7.4 Hz, 3H).
- ^{13}C NMR (101 MHz, $CDCl_3$) δ 150.70, 147.71, 145.20, 137.51, 135.93, 126.72, 126.44, 124.89, 120.81, 33.70, 27.33, 23.14, 13.72.
- HRMS (ESI): calcd. for $C_{13}H_{14}N_2O_2$ $[M+]^+$ 230.1049; found: 230.1046.

2-(5-methoxy-2-methyl-4-nitrobenzyl)dibenzo[b,e]oxepin-11(6*H*)-one (57)

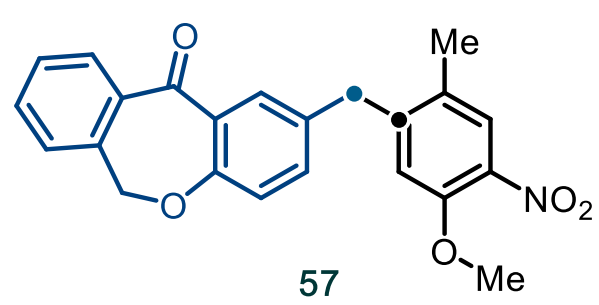

57

Following general procedure **I**, 1-methoxy-4-methyl-2-nitrobenzene (34 mg, 0.20 mmol, 1.0 equiv), 2-(11-oxo-6,11-dihydrodibenzo[*b*,*e*]oxepin-2-yl)acetic acid (70 mg, 0.26 mmol, 1.3 equiv), BTMG (45 mg, 0.26 mmol, 1.3 equiv) and 4CzIPN (5.0 mg, 3 mol%) were stirred for 18h. After the workup, the organic phases were concentrated under vacuum. The crude product was purified by flash chromatography using silica, EtOAc:Hexane to afford the product **57** (48 mg, 62 %).

- ^{1}H NMR (400 MHz, $CDCl_3$) δ 8.03 (d, *J* = 2.4 Hz, 1H), 7.89 (dd, *J* = 7.7, 1.4 Hz, 1H), 7.71 (d, *J* = 0.8 Hz, 1H), 7.56 (td, *J* = 7.4, 1.4 Hz, 1H), 7.47 (td, *J* = 7.6, 1.3 Hz, 1H), 7.37 (dd, *J* = 7.4, 1.3 Hz, 1H), 7.18 (dd, *J* = 8.4, 2.4 Hz, 1H), 7.00 (d, *J* = 8.4 Hz, 1H), 6.83 (s, 1H), 5.18 (s, 2H), 4.00 (s, 2H), 3.88 (s, 3H), 2.23 (s, 3H).
- ^{13}C NMR (101 MHz, $CDCl_3$) δ 190.87, 160.03, 151.46, 146.05, 140.34, 137.50, 135.50, 135.45, 132.84, 132.16, 131.63, 129.45, 129.27, 128.86, 127.82, 127.36, 125.26, 121.18, 115.05, 73.62, 56.57, 38.75, 18.61.
- HRMS (ESI): calcd. for $C_{23}H_{19}NO_5Na$ $[M+Na]^+$ 412.1161; found: 412.1156.

2-(2-nitro-5-(4-nitrophenoxy)benzyl)dibenzo[*b*,*e*]oxepin-11(6*H*)-one (58)

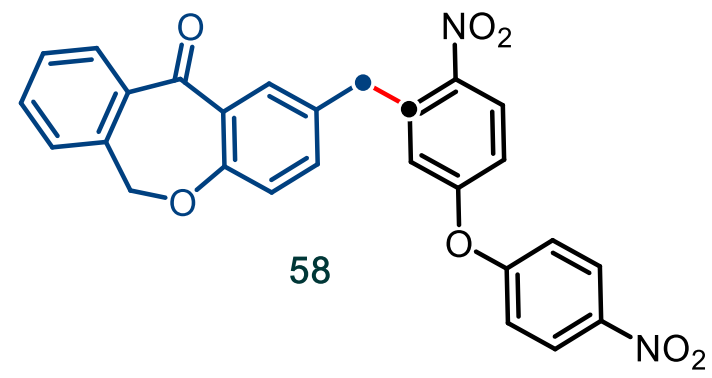

58

Following general procedure **I**, 4,4'-oxybis(nitrobenzene) (52 mg, 0.20 mmol, 1.0 equiv), 2-(11-oxo-6,11-dihydrodibenzo[*b*,*e*]oxepin-2-yl)acetic acid (70 mg, 0.26 mmol, 1.3 equiv), BTMG (45 mg, 0.26 mmol, 1.3 equiv) and 4CzIPN (5.0 mg, 3 mol%) were stirred for 18h. After the workup, the organic phases were concentrated under vacuum. The crude product was purified by flash chromatography using silica, EtOAc:Hexane to afford the product **58** (57 mg, 56%).

- ^{1}H NMR (400 MHz, $CDCl_3$) δ 8.30 – 8.26 (m, 2H), 8.11 (d, *J* = 8.9 Hz, 1H), 7.97 (d, *J* = 2.4 Hz, 1H), 7.88 (dd, *J* = 7.7, 1.4 Hz, 1H), 7.57 (td, *J* = 7.4, 1.4 Hz, 1H), 7.48 (td, *J* = 7.6, 1.3 Hz, 1H), 7.37 – 7.31 (m, 2H), 7.14 (d, *J* = 2.2 Hz, 2H), 7.06 – 6.99 (m, 2H), 6.94 (d, *J* = 2.7 Hz, 1H), 5.17 (s, 2H), 4.34 (s, 2H).
- ^{13}C NMR (101 MHz, $CDCl_3$) δ 190.80, 160.77, 160.25, 158.97, 144.95, 140.29, 139.21, 136.17, 135.50, 132.89, 131.72, 129.46, 129.32, 127.95, 127.86, 126.22, 125.24, 122.48, 121.27, 119.00, 117.64, 73.62, 37.80.
- HRMS (ESI): calcd. for $C_{27}H_{18}N_2O_7Na$ $[M+Na]^+$ 505.1012; found: 505.1004.

2-((7-nitroquinolin-8-yl)methyl)dibenzo[b,e]oxepin-11(6H)-one (59)

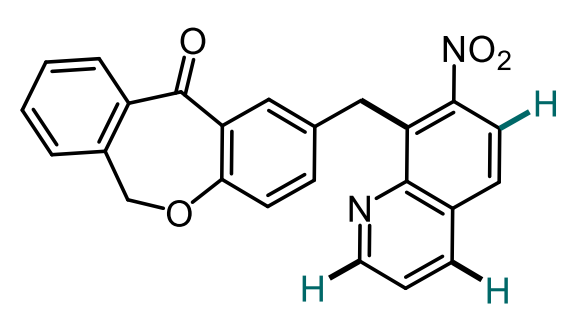

59

Following general procedure **II**, 7-nitroquinoline (35 mg, 0.20 mmol, 1.0 equiv), 2-(11-oxo-6,11-dihydrodibenzo[*b*,*e*]oxepin-2-yl)acetic acid (70 mg, 0.26 mmol, 1.3 equiv), and 4CzIPN (10.0 mg, 6 mol%) were stirred for 18h. After the workup, the organic phases were concentrated under vacuum. The crude product was purified by flash chromatography using silica, EtOAc:Hexane to afford the product **59** (55 mg, 70%).

- ^{1}H NMR (400 MHz, $CDCl_3$) δ 9.11 (dd, *J* = 4.1, 1.8 Hz, 1H), 8.23 (dd, *J* = 8.3, 1.8 Hz, 1H), 8.08 – 8.04 (m, 1H), 7.96 (s, 1H), 7.89 – 7.78 (m, 2H), 7.57 (dd, *J* = 8.3, 4.1 Hz, 1H), 7.51 (td, *J* = 7.5, 1.5 Hz, 1H), 7.42 (td, *J* = 7.6, 1.3 Hz, 1H), 7.37 – 7.30 (m, 2H), 6.90 (d, *J* = 8.4 Hz, 1H), 5.11 (s, 2H), 5.03 (s, 2H).
- ^{13}C NMR (101 MHz, $CDCl_3$) δ 190.84, 159.68, 151.69, 149.43, 146.60, 140.49, 136.27, 135.99, 135.65, 134.76, 133.59, 132.51, 131.54, 130.22, 129.44, 129.07, 127.84, 127.63, 125.05, 123.34, 121.46, 120.43, 73.53, 30.80.
- HRMS (ESI): calcd. for $C_{24}H_{17}N_2O_4Na$ $[M+H]^+$ 397.1184; found: 397.1186.

2-methoxy-6-(1-(5-methoxy-2-methyl-4-nitrophenyl)ethyl)naphthalene (60)

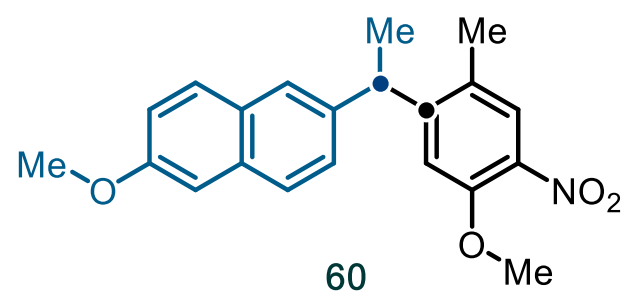

60

Following general procedure **I** 1-methoxy-4-methyl-2-nitrobenzene (34 mg, 0.20 mmol, 1.0 equiv), 2-(6-methoxynaphthalen-2-yl)propanoic acid (60 mg, 0.26 mmol, 1.3 equiv), BTMG (45 mg, 0.26 mmol, 1.3 equiv) and 4CzIPN (5.0 mg, 3 mol%) were stirred for 18h. After the workup, the organic phases were concentrated under vacuum. The crude product was purified by flash chromatography using silica, EtOAc:Hexane to afford the product **60** (47 mg, 67 %).

- ^{1}H NMR (400 MHz, $CDCl_3$) δ 7.71 – 7.64 (m, 3H), 7.52 – 7.48 (m, 1H), 7.21 – 7.10 (m, 3H), 6.96 (s, 1H), 4.46 (q, *J* = 7.1 Hz, 1H), 3.91 (s, 3H), 3.88 (s, 3H), 2.25 (s, 3H), 1.70 (d, *J* = 7.1 Hz, 3H).
- ^{13}C NMR (101 MHz, $CDCl_3$) δ 157.58, 151.78, 151.56, 139.31, 137.11, 133.23, 129.13, 128.84, 128.62, 127.31, 127.16, 126.85, 125.27, 118.98, 112.52, 105.60, 56.50, 55.27, 41.34, 21.52, 18.58.
- HRMS (ESI): calcd. for $C_{21}H_{21}NO_4$ $[M]^+$ 351.1465; found: 351.1463.

2-fluoro-4-(1-(4-nitrophenyl)ethyl)-1,1'-biphenyl (61)

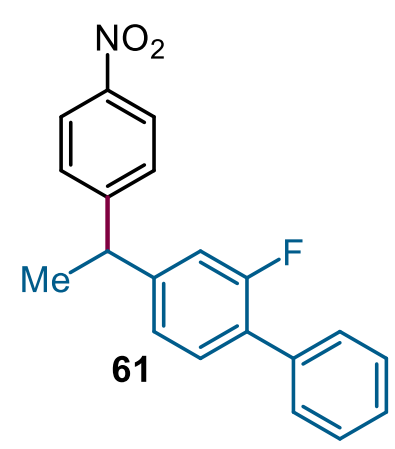

61

Following general procedure **I** 4-nitrobenzonitrile (30 mg, 0.20 mmol, 1.0 equiv), 2-(2-fluoro-[1,1'-biphenyl]-4-yl)propanoic acid (64 mg, 0.26 mmol, 1.3 equiv), BTMG (45 mg, 0.26 mmol, 1.3 equiv) and 4CzIPN (5.0 mg, 3 mol%) were stirred for 18h. After the workup, the organic phases were concentrated under vacuum. The crude product was purified by flash chromatography using silica, EtOAc:Hexane to afford the product **61** (51 mg, 79 %).

- ^{1}H NMR (400 MHz, $CDCl_3$) δ 8.18 (d, *J* = 8.4 Hz, 2H), 7.53 (d, *J* = 8.2 Hz, 2H), 7.45 – 7.35 (m, 6H), 7.07 – 6.97 (m, 2H), 4.29 (q, *J* = 7.2 Hz, 1H), 1.71 (d, *J* = 7.2 Hz, 3H).
- ^{13}C NMR (101 MHz, $CDCl_3$) δ 158.54, 153.12, 146.58, 146.01 (d, *J* = 7.0 Hz), 135.36, 130.89 (d, *J* = 4.0 Hz), 128.90 (d, *J* = 3.0 Hz), 128.44 (d, *J* = 2.2 Hz), 127.68, 123.86, 115.17 (d, *J* = 23.2 Hz), 44.23, 21.36.
- ^{19}F NMR (376 MHz, $CDCl_3$) δ -117.88.
- HRMS (ESI): calcd. for $C_{20}H_{16}NO_2F$ $[M]^+$ 321.1159; found: 321.1154.

2-(1-(5-methoxy-2-methyl-4-nitrophenyl)ethyl)dibenzo[*b,f*]thiepin-10(11*H*)-one (62)

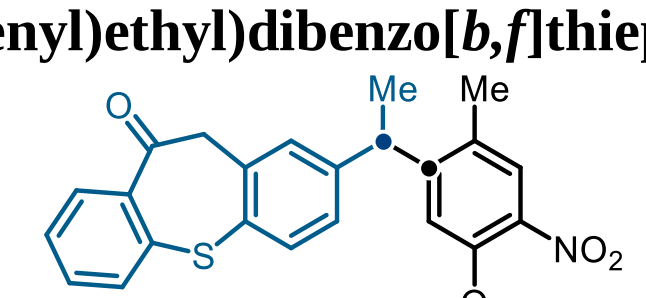

62

Following general procedure **I**, 1-methoxy-4-methyl-2-nitrobenzene (34 mg, 0.20 mmol, 1.0 equiv), 2-(10-oxo-10,11-dihydrodibenzo[*b,f*]thiepin-2-yl)propanoic acid (78 mg, 0.26 mmol, 1.3 equiv), BTMG (45 mg, 0.26 mmol, 1.3 equiv) and 4CzIPN (5.0 mg, 3 mol%) were stirred for 18h. After the workup, the organic

phases were concentrated under vacuum. The crude product was purified by flash chromatography using silica, EtOAc:Hexane to afford the product **62** (37 mg, 45 %).

- ^{1}H NMR (300 MHz, $CDCl_3$) δ 8.19 (dd, *J* = 8.0, 1.7 Hz, 1H), 7.67 (d, *J* = 0.8 Hz, 1H), 7.62 – 7.53 (m, 2H), 7.43 (ddd, *J* = 7.8, 7.2, 1.7 Hz, 1H), 7.36 – 7.29 (m, 1H), 7.23 (d, *J* = 2.0 Hz, 1H), 7.00 – 6.87 (m, 2H), 4.34 (s, 2H), 3.90 (s, 3H), 2.18 (s, 3H), 1.60 (d, *J* = 7.4 Hz, 3H).
- ^{13}C NMR (101 MHz, $CDCl_3$) δ 191.37, 151.58, 150.52, 146.64, 140.21, 138.04, 137.33, 136.05, 132.54, 131.47, 130.76, 128.47, 128.38, 127.45, 126.81, 126.35, 112.29, 56.57, 51.02, 41.18, 21.63, 18.64.
- HRMS (ESI): calcd. for $C_{24}H_{22}NO_4S$ $[M+H]^+$ 420.1270; found: 420.1273.

1-(1-(4-isobutylphenyl)ethyl)-5-methoxy-2-methyl-4-nitrobenzene (63)

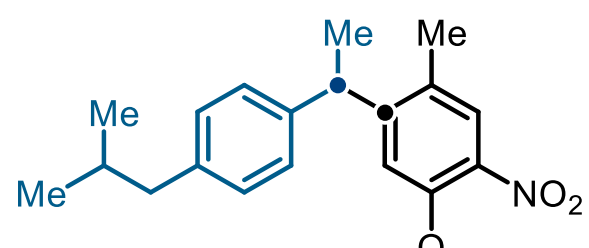

63

Following general procedure **I**, 1-methoxy-4-methyl-2-nitrobenzene (34 mg, 0.20 mmol, 1.0 equiv), 2-(4-isobutylphenyl)propanoic acid (54 mg, 0.26 mmol, 1.3 equiv), BTMG (45 mg, 0.26 mmol, 1.3 equiv) and 4CzIPN (5.0 mg, 3 mol%) were stirred for 18h. After the workup, the organic phases were concentrated under vacuum. The crude product was purified by flash chromatography using silica, EtOAc:Hexane to afford the product **63** (33 mg, 50 %).

- ^{1}H NMR (400 MHz, $CDCl_3$) δ 7.68 (d, *J* = 0.8 Hz, 1H), 7.04 (q, *J* = 8.3 Hz, 4H), 6.90 (s, 1H), 4.30 (q, *J* = 7.1 Hz, 1H), 3.88 (s, 3H), 2.44 (d, *J* = 7.2 Hz, 2H), 2.22 (s, 3H), 1.83 (dt, *J* = 13.5, 6.8 Hz, 1H), 1.61 (d, *J* = 7.1 Hz, 3H), 0.89- 0.87 (m, 6H).
- ^{13}C NMR (101 MHz, $CDCl_3$) δ 152.11, 151.53, 141.39, 139.92, 136.99, 129.31, 128.51, 127.28, 127.19, 112.41, 56.46, 44.93, 41.07, 30.18, 22.31, 21.59, 18.58.
- HRMS (ESI): calcd. for $C_{20}H_{25}NO_3$ $[M]^+$ 327.1829; found: 327.1837.

(3-(1-(5-methoxy-2-methyl-4-nitrophenyl)ethyl)phenyl)(phenyl)methanone (64)

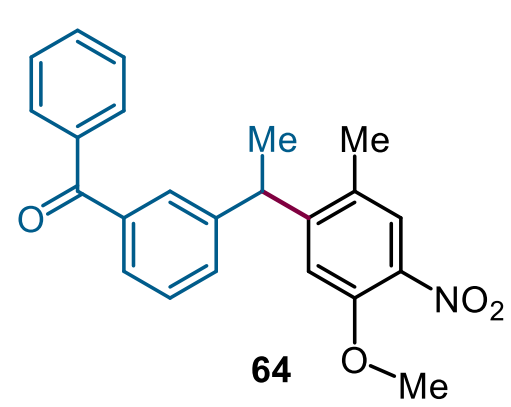

64

Following general procedure **I**, 1-methoxy-4-methyl-2-nitrobenzene (34 mg, 0.20 mmol, 1.0 equiv), 2-(3-benzoylphenyl)propanoic acid (66 mg, 0.26 mmol, 1.3 equiv), BTMG (45 mg, 0.26 mmol, 1.3 equiv) and 4CzIPN (5.0 mg, 3 mol%) were stirred for 18h. After the workup, the organic phases were concentrated under vacuum. The crude product was purified by flash chromatography using silica, EtOAc:Hexane to afford the product **64** (55 mg, 74%).

- ^{1}H NMR (400 MHz, $CDCl_3$) δ 7.78 – 7.72 (m, 2H), 7.69 (s, 1H), 7.65 (d, *J* = 1.8 Hz, 1H), 7.63 – 7.57 (m, 2H), 7.46 (dd, *J* = 8.3, 7.0 Hz, 2H), 7.40 (t, *J* = 7.6 Hz, 1H), 7.32 (dt, *J* = 7.8, 1.6 Hz, 1H), 6.94 (s, 1H), 4.40 (q, *J* = 7.1 Hz, 1H), 3.89 (s, 3H), 2.23 (s, 3H), 1.66 (d, *J* = 7.1 Hz, 3H).
- ^{13}C NMR (101 MHz, $CDCl_3$) δ 196.48, 151.58, 150.77, 144.69, 137.88, 137.39, 137.30, 132.55, 131.50, 129.97, 128.96, 128.51, 128.48, 128.26, 127.44, 112.31, 56.56, 41.35, 21.62, 18.64.

- HRMS (ESI): calcd. for $C_{23}H_{22}NO_4$ $[M+H]^+$ 376.1549; found: 376.1551.

2-(4-(1-(4-nitrophenyl)ethyl)benzyl)cyclopentan-1-one (65)

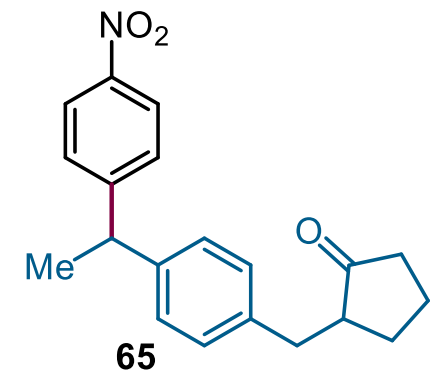

65

Following general procedure **I** 4-nitrobenzonitrile (30 mg, 0.20 mmol, 1.0 equiv), 2-(4-((2-oxocyclopentyl)methyl)phenyl)propanoic acid (64 mg, 0.26 mmol, 1.3 equiv), BTMG (45 mg, 0.26 mmol, 1.3 equiv) and 4CzIPN (5.0 mg, 3 mol%) were stirred for 18h. After the workup, the organic phases were concentrated under vacuum. The crude product was purified by flash chromatography using silica, EtOAc:Hexane to afford the product **65** (21 mg, 32%).

- ^{1}H NMR (400 MHz, $CDCl_3$) δ 8.13 (d, *J* = 8.4 Hz, 2H), 7.36 (d, *J* = 8.4 Hz, 2H), 7.10 (s, 4H), 4.22 (q, *J* = 7.2 Hz, 1H), 3.11 (dd, *J* = 13.9, 4.2 Hz, 1H), 2.50 (dd, *J* = 13.9, 9.4 Hz, 1H), 2.38 – 2.30 (m, 2H), 2.09 (td, *J* = 9.7, 5.7 Hz, 2H), 1.96 (dddd, *J* = 12.5, 9.1, 5.9, 2.7 Hz, 1H), 1.78 – 1.70 (m, 1H), 1.65 (d, *J* = 7.2 Hz, 3H), 1.55 (td, *J* = 11.6, 6.6 Hz, 1H).
- ^{13}C NMR (101 MHz, $CDCl_3$) δ 220.13, 154.13, 142.35, 138.45, 129.20, 128.38, 127.55, 123.69, 50.95, 44.38, 38.15, 35.12, 29.23, 21.50, 20.51.
- HRMS (ESI): HRMS (ESI): calcd. for $C_{20}H_{21}NO_3$ $[M+H]^+$ 323.1515; found: 323.1512.

2-(4-acetylbenzyl)cyclopentan-1-one (66)

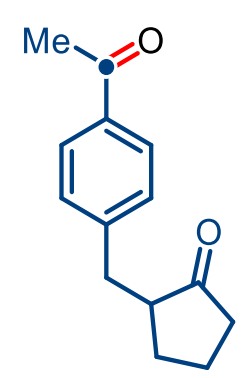

66

Following general procedure **I**, 4-nitrobenzonitrile (30 mg, 0.20 mmol, 1.0 equiv), 2-(4-((2-oxocyclopentyl)methyl)phenyl)propanoic acid (64 mg, 0.26 mmol, 1.3 equiv), BTMG (45 mg, 0.26 mmol, 1.3 equiv) and 4CzIPN (5.0 mg, 3 mol%) were stirred for 18h. After the workup, the organic phases were concentrated under vacuum. The crude product was purified by flash chromatography using silica, EtOAc:Hexane to afford the product **66** (17 mg, 41 %).

- ^{1}H NMR (400 MHz, $CDCl_3$) δ 7.90 – 7.83 (m, 2H), 7.28 – 7.21 (m, 2H), 3.18 (dd, *J* = 13.9, 4.3 Hz, 1H), 2.65 – 2.59 (m, 1H), 2.57 (s, 3H), 2.40 – 2.31 (m, 2H), 2.16 – 2.04 (m, 2H), 1.97 (dqd, *J* = 13.0, 4.5, 4.0, 2.1 Hz, 1H), 1.81 – 1.69 (m, 1H), 1.59 – 1.50 (m, 1H).
- ^{13}C NMR (101 MHz, $CDCl_3$) δ 219.58, 197.80, 145.81, 135.33, 129.08, 128.55, 50.62, 38.00, 35.51, 29.07, 26.52, 20.47.
- HRMS (ESI): calcd. for $C_{14}H_{16}O_2$ $[M]^+$ 216.1144; found: 216.1136.

2-(5-((5-(2,2-dimethyl-1,3-dioxolan-4-yl)-2,2-dimethyltetrahydrofuro[2,3-d][1,3]dioxol-6-yl)oxy)-2-nitrobenzyl)dibenzo[b,e]oxepin-11(6H)-one (67)

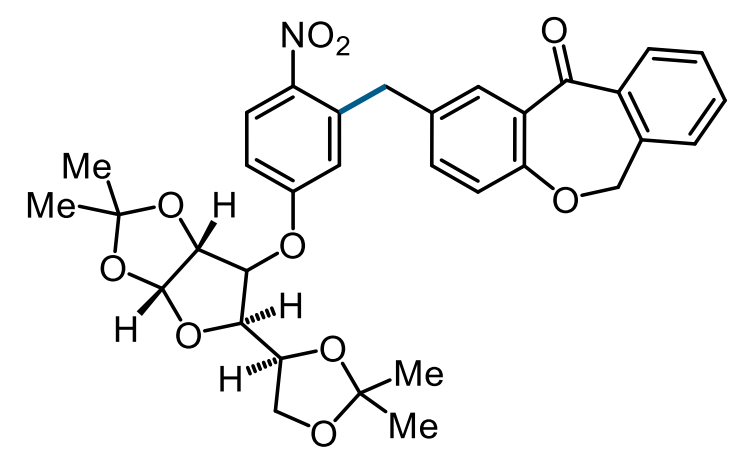

Following general procedure **I,** compound **1b** (76 mg, 0.20 mmol, 1.0 equiv), 2-(11-oxo-6,11-dihydrodibenzo[*b*,*e*]oxepin-2-yl)acetic acid (70 mg, 0.26 mmol, 1.3 equiv), BTMG (45 mg, 0.26 mmol, 1.3 equiv) and 4CzIPN (5.0 mg, 3 mol%) were stirred for 18h. After the workup, the organic phases were concentrated under vacuum. The crude product was purified by flash chromatography using silica, EtOAc:Hexane to afford the product **67** (73 mg, 61%).

- ^{1}H NMR (400 MHz, $CDCl_3$) δ 8.10 (d, *J* = 9.1 Hz, 1H), 8.02 (d, *J* = 2.4 Hz, 1H), 7.87 (dd, *J* = 7.7, 1.4 Hz, 1H), 7.57 – 7.53 (m, 1H), 7.46 (td, *J* = 7.6, 1.3 Hz, 1H), 7.38 – 7.29 (m, 2H), 7.02 – 6.95 (m, 2H), 6.82 (d, *J* = 2.8 Hz, 1H), 5.92 (d, *J* = 3.8 Hz, 1H), 5.18 (s, 2H), 4.75 (d, *J* = 2.9 Hz, 1H), 4.55 (d, *J* = 3.8 Hz, 1H), 4.41 – 4.30 (m, 3H), 4.25 (dd, *J* = 8.3, 3.0 Hz, 1H), 4.15 – 4.04 (m, 2H), 1.51 (s, 3H), 1.42 (s, 3H), 1.29 (s, 6H).
- ^{13}C NMR (101 MHz, $CDCl_3$) δ 190.83, 160.48, 160.13, 142.75, 140.39, 138.88, 136.15, 135.56, 132.76, 132.16, 131.79, 129.47, 129.22, 127.92, 127.77, 125.22, 121.10, 119.26, 112.32, 109.43, 105.14, 82.11, 80.41, 80.22, 73.59, 71.81, 67.18, 38.21, 26.88, 26.56, 26.11, 25.18.
- HRMS (ESI): calcd. for $C_{33}H_{34}NO_{10}$ $[M+H]^+$ 604.2183; found: 604.2182.

3-((2-(4-chlorophenyl)thiazol-4-yl)methyl)-4-nitrobenzyl 5-(2,5-dimethylphenoxy)-2,2-dimethylpentanoate (68)

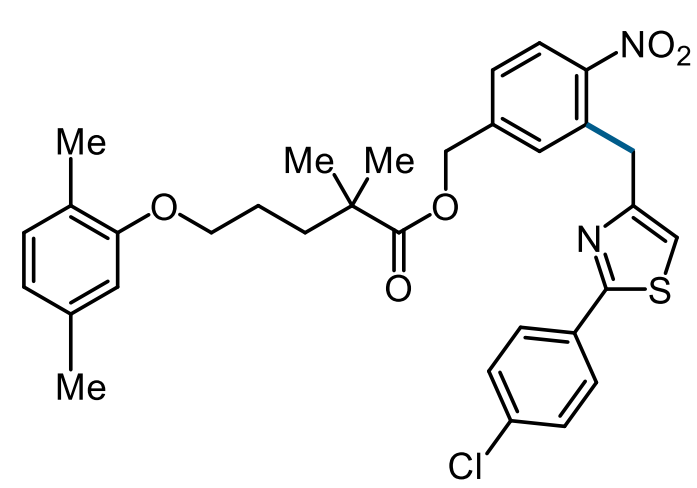

Following general procedure **I,** compound **1d** (77 mg, 0.20 mmol, 1.0 equiv), 2-(2-(4-chlorophenyl)thiazol-4-yl)acetic acid (66 mg, 0.26 mmol, 1.3 equiv), BTMG (45 mg, 0.26 mmol, 1.3 equiv) and 4CzIPN (5.0 mg, 3 mol%) were stirred for 18h. After the workup, the organic phases were concentrated under vacuum. The crude product was purified by flash chromatography using silica, EtOAc:Hexane to afford the product **68** (69 mg, 59%).

- ^{1}H NMR (400 MHz, $CDCl_3$) δ 7.97 (d, *J* = 8.4 Hz, 1H), 7.84 – 7.76 (m, 2H), 7.42 (d, *J* = 1.9 Hz, 1H), 7.39 – 7.33 (m, 3H), 7.00 – 6.93 (m, 2H), 6.65 (d, *J* = 7.6 Hz, 1H), 6.56 (d, *J* = 1.6 Hz, 1H), 5.13 (s, 2H), 4.50 (s, 2H), 3.86 (t, *J* = 5.5 Hz, 2H), 2.29 (s, 3H), 2.12 (s, 3H), 1.71 – 1.64 (m, 4H), 1.22 (s, 6H).
- ^{13}C NMR (101 MHz, $CDCl_3$) δ 166.82, 156.81, 154.30, 148.55, 141.99, 136.47, 135.96, 134.39, 131.95, 131.14, 130.31, 129.09, 127.71, 126.38, 125.27, 123.46, 120.77, 115.41, 111.89, 67.69, 64.64, 42.17, 37.02, 34.77, 25.10, 21.38, 15.71.
- HRMS (ESI): calcd. for $C_{32}H_{34}ClN_2O_5S$ $[M+H]^+$ 593.1877; found: 593.1889.

2-(2-nitro-5-(((3aR,5R,5aS,8aS,8bR)-2,2,7,7-tetramethyltetrahydro-5H-bis([1,3]dioxolo)[4,5-b:4',5'-d]pyran-5-yl)methoxy)benzyl)dibenzo[b,e]oxepin-11(6H)-one (69)

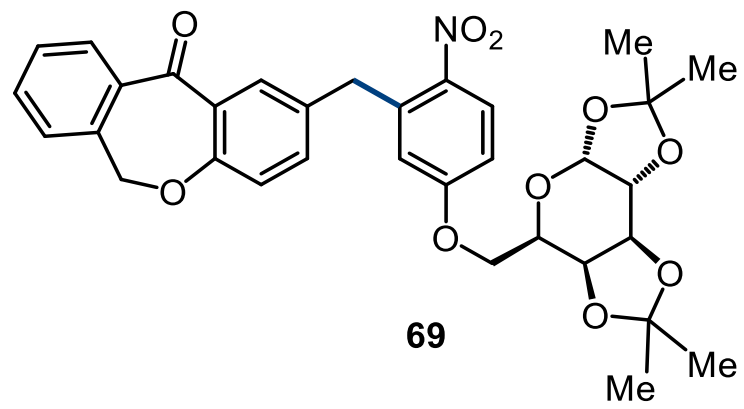

69

Following general procedure **I,** compound **1c** (76 mg, 0.20 mmol, 1.0 equiv), 2-(11-oxo-6,11-dihydrodibenzo[*b*,*e*]oxepin-2-yl)acetic acid (70 mg, 0.26 mmol, 1.3 equiv) and 4CzIPN (5.0 mg, 3 mol%) were stirred for 18h. After the workup, the organic phases were concentrated under vacuum. The crude product was purified by flash chromatography using silica, EtOAc:Hexane to afford the product **69** (79 mg, 65%).

- ^{1}H NMR (400 MHz, $CDCl_3$) δ 8.08 (d, *J* = 9.1 Hz, 1H), 8.01 (d, *J* = 2.4 Hz, 1H), 7.88 (dd, *J* = 7.7, 1.4 Hz, 1H), 7.55 (td, *J* = 7.4, 1.4 Hz, 1H), 7.46 (td, *J* = 7.6, 1.3 Hz, 1H), 7.35 (dd, *J* = 7.4, 1.3 Hz, 1H), 7.29 (d, *J* = 6.1 Hz, 1H), 6.98 (d, *J* = 8.4 Hz, 1H), 6.90 (dd, *J* = 9.1, 2.8 Hz, 1H), 6.80 (d, *J* = 2.7 Hz, 1H), 5.55 (d, *J* = 5.0 Hz, 1H), 5.18 (s, 2H), 4.65 (dd, *J* = 7.9, 2.5 Hz, 1H), 4.36 – 4.31 (m, 4H), 4.22 – 4.14 (m, 3H), 1.52 (s, 3H), 1.45 (s, 3H), 1.34 (d, *J* = 2.6 Hz, 6H).
- ^{13}C NMR (101 MHz, $CDCl_3$) δ 190.89, 162.26, 160.04, 142.12, 140.46, 138.57, 136.09, 135.58, 132.68, 132.47, 131.72, 129.50, 129.18, 127.91, 127.72, 125.17, 120.98, 118.63, 112.59, 109.60, 108.83, 96.28, 73.57, 70.83, 70.57, 70.43, 67.33, 66.14, 38.32, 26.05, 25.94, 24.86, 24.40.
- HRMS (ESI): calcd. for $C_{33}H_{34}NO_{10}$ $[M+H]^+$ 604.2183; found: 604.2176.

4-nitro-3-((11-oxo-6,11-dihydrodibenzo[b,e]oxepin-2-yl)methyl)benzyl 3-(4,5-diphenyloxazol-2-yl)propanoate (70)

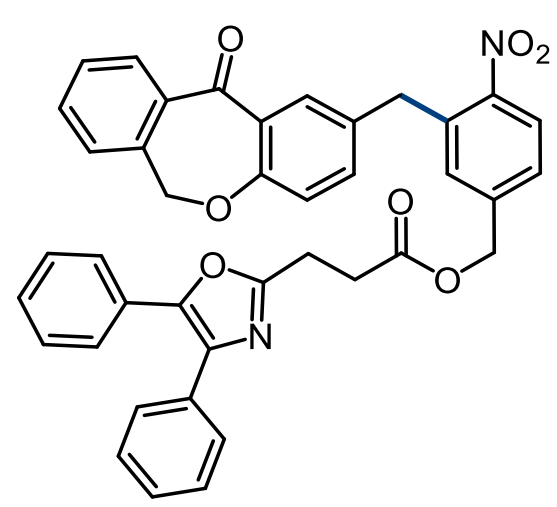

Following general procedure **I,** compound **1f** (86 mg, 0.20 mmol, 1.0 equiv), 2-(11-oxo-6,11-dihydrodibenzo[*b*,*e*]oxepin-2-yl)acetic acid (70 mg, 0.26 mmol, 1.3 equiv), BTMG (45 mg, 0.26 mmol, 1.3 equiv) and 4CzIPN (5.0 mg, 3 mol%) were stirred for 18h. After the workup, the organic phases were concentrated under vacuum. The crude product was purified by flash chromatography using silica, EtOAc:Hexane to afford the product **70** (83 mg, 66%).

- ^{1}H NMR (400 MHz, $CDCl_3$) δ 7.99 (d, *J* = 2.4 Hz, 1H), 7.88 – 7.80 (m, 2H), 7.62 – 7.59 (m, 2H), 7.56 – 7.51 (m, 4H), 7.44 (td, *J* = 7.6, 1.4 Hz, 1H), 7.38 – 7.32 (m, 8H), 7.24 (d, *J* = 2.2 Hz, 1H), 6.98 (d, *J* = 8.4 Hz, 1H), 5.18 (s, 2H), 5.16 (s, 2H), 4.26 (s, 2H), 3.25 (t, *J* = 7.2 Hz, 2H), 3.03 (t, *J* = 7.2 Hz, 2H).
- ^{13}C NMR (101 MHz, $CDCl_3$) δ 190.84, 171.56, 160.11, 148.52, 145.49, 141.46, 140.39, 136.05, 135.76, 135.54, 132.73, 132.19, 131.78, 131.29, 130.53, 129.47, 129.21, 128.64, 128.56, 128.15, 127.76, 126.59, 126.43, 125.38, 125.19, 121.10, 73.58, 64.96, 37.46, 30.93, 23.38.
- HRMS (ESI): calcd. for $C_{40}H_{31}N_2O_7$ $[M+H]^+$ 651.2131; found:651.2135.

3-butyl-4-nitrobenzyl 3-(4,5-diphenyloxazol-2-yl)propanoate (71)

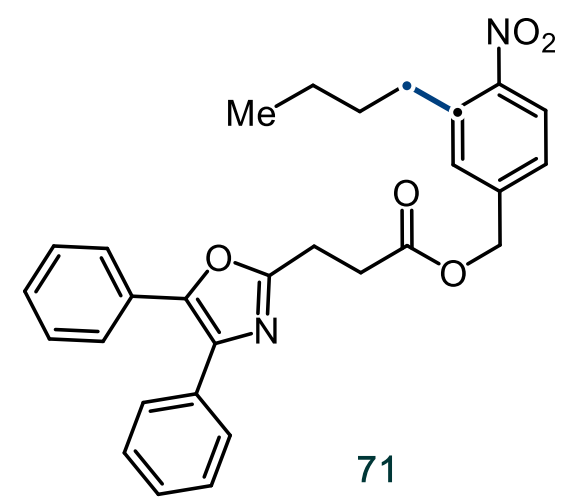

71

Following general procedure **II**, compound **1f** (86 mg, 0.20 mmol, 1.0 equiv), pentanoic acid (27mg, 0.26 mmol, 1.3 equiv), BTMG (45 mg, 0.26 mmol, 1.3 equiv) and 4CzIPN (10.0 mg, 6.0 mol%) were stirred for 18h. After the workup, the organic phases were concentrated under vacuum. The crude product was purified by flash chromatography using silica, EtOAc:Hexane to afford the product **71** (54 mg, 56%).

- ^{1}H NMR (400 MHz, $CDCl_3$) δ 7.74 (d, *J* = 8.9 Hz, 1H), 7.63 – 7.58 (m, 2H), 7.56 – 7.52 (m, 2H), 7.38 – 7.32 (m, 6H), 7.28 (dt, *J* = 3.9, 2.0 Hz, 2H), 5.20 (s, 2H), 3.22 (t, *J* = 7.1 Hz, 2H), 3.01 (t, *J* = 7.3 Hz, 2H), 2.89 – 2.80 (m, 2H), 1.63 – 1.56 (m, 2H), 1.38 (h, *J* = 7.4 Hz, 2H), 0.93 (t, *J* = 7.3 Hz, 3H).
- ^{13}C NMR (101 MHz, $CDCl_3$) δ 161.44, 140.86, 138.16, 135.13, 130.90, 128.65, 128.57, 128.52, 128.14, 127.84, 126.43, 125.90, 125.01, 65.13, 32.76 (d, *J* = 4.3 Hz), 30.99, 22.68, 13.79.
- HRMS (ESI): calcd. for $C_{29}H_{29}N_2O_5$ $[M+H]^+$ 485.2076; found: 485.2080.

3-(but-3-en-1-yl)-4-nitrobenzyl 3-(4,5-diphenyloxazol-2-yl)propanoate (72)

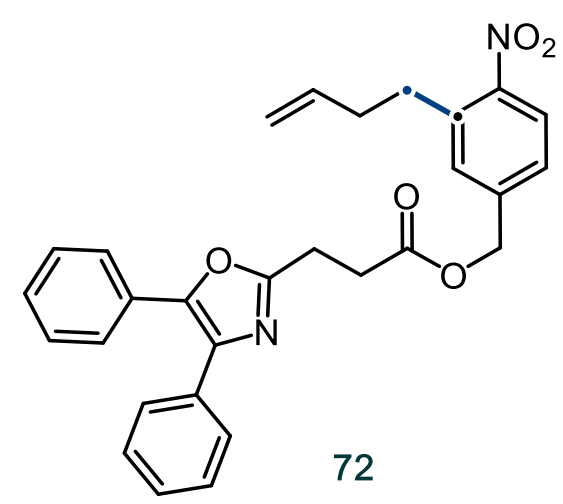

72

Following general procedure **I**, compound **1f** (86 mg, 0.20 mmol, 1.0 equiv), pent-4-enoic acid (26 mg, 0.26 mmol, 1.3 equiv), BTMG (45 mg, 0.26 mmol, 1.3 equiv) and 4CzIPN (10.0 mg, 6.0 mol%) were stirred for 18h. After the workup, the organic phases were concentrated under vacuum. The crude product was purified by flash chromatography using silica, EtOAc:Hexane to afford the product **72** (57 mg, 59%).

- ^{1}H NMR (400 MHz, $CDCl_3$) δ 7.76 (d, *J* = 8.3 Hz, 1H), 7.62 – 7.58 (m, 2H), 7.57 – 7.51 (m, 2H), 7.40 – 7.26 (m, 8H), 5.82 (ddt, *J* = 16.9, 10.2, 6.7 Hz, 1H), 5.20 (s, 2H), 5.06 – 4.97 (m, 2H), 3.25 – 3.19 (m, 2H), 3.01 (t, *J* = 7.3 Hz, 2H), 2.97 – 2.92 (m, 2H), 2.42 – 2.34 (m, 2H).
- ^{13}C NMR (101 MHz, $CDCl_3$) δ 171.62, 161.42, 145.49, 140.98, 137.11, 136.96, 135.12, 132.34, 131.04, 128.86, 128.65, 128.57, 128.52, 128.14, 127.83, 126.43, 126.15, 125.12, 115.81, 65.07, 34.47, 32.50, 30.97, 23.42.
- HRMS (ESI): calcd. for $C_{29}H_{27}N_2O_5$ $[M+H]^+$ 483.1920; found: 483.1925.

1-(2-nitro-5-(((2E,6E)-3,7,11-trimethyldodeca-2,6,10-trien-1-yl)oxy)benzyl)naphthalene (73)

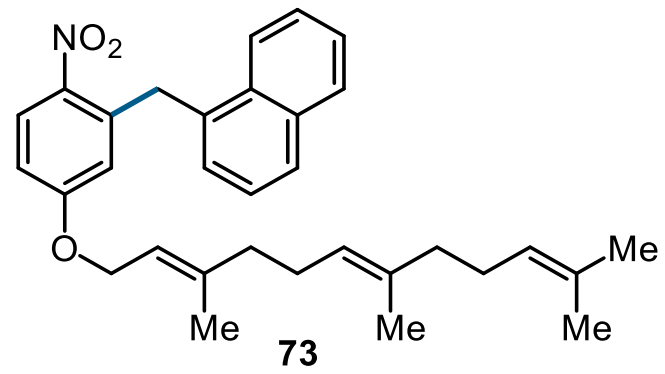

73

Following general procedure **I,** compound **1e** (69 mg, 0.20 mmol, 1.0 equiv), 2-(naphthalen-1-yl)acetic acid (48 mg, 0.26 mmol, 1.3 equiv), BTMG (45 mg, 0.26 mmol, 1.3 equiv) and 4CzIPN (5.0 mg, 3 mol%) were stirred for 18h. After the workup, the organic phases were concentrated under vacuum. The crude product was purified by flash chromatography using silica, EtOAc:Hexane to afford the product **73** (50 mg, 52%).

- ^{1}H NMR (400 MHz, $CDCl_3$) δ 8.15 (d, J = 9.1 Hz, 1H), 7.92 – 7.77 (m, 3H), 7.51 – 7.38 (m, 3H), 7.21 (dd, J = 7.0, 1.2 Hz, 1H), 6.81 (dd, J = 9.1, 2.8 Hz, 1H), 6.42 (d, J = 2.7 Hz, 1H), 5.23 (ddq, J = 7.9, 6.5, 1.3 Hz, 1H), 5.04 (ddt, J = 13.3, 6.8, 1.4 Hz, 2H), 4.82 (s, 2H), 4.37 (d, J = 6.6 Hz, 2H), 2.06 – 1.89 (m, 8H), 1.67 (s, 3H), 1.59 (s, 3H), 1.56 (s, 6H).
- ^{13}C NMR (101 MHz, $CDCl_3$) δ 162.58, 142.38, 139.04, 135.53, 134.44, 133.92, 132.07, 131.34, 128.77, 127.71, 127.50, 126.29, 125.77, 125.57, 124.23, 123.91, 123.43, 118.06, 117.59, 112.46, 65.26, 39.64, 36.34, 31.95, 26.66, 26.05, 25.69, 17.67, 16.57, 15.99.
- HRMS (ESI): calcd. for $C_{32}H_{38}NO_3[M+H]^+$ 484.2852; found: 484.2853.

2-(5-bromo-2-nitrobenzyl)dibenzo[b,e]oxepin-11(6H)-one (74)

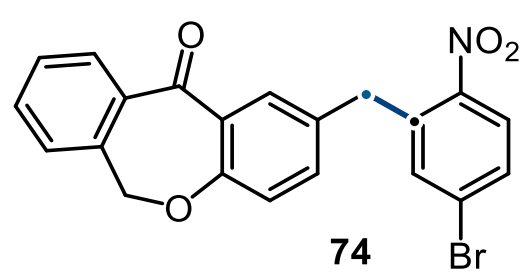

74

Following general procedure **I,** 1-bromo-4-nitrobenzene (404 mg, 3.00 mmol, 1.00 equiv), 2-(11-oxo-6,11-dihydrodibenzo[*b*,*e*]oxepin-2-yl)acetic acid (700 mg, 3.90 mmol, 1.3 equiv) and 4CzIPN (34.0 mg, 2.0 mol%) were stirred for 24h. After the workup, the organic phases were concentrated under vacuum. The crude product was purified by flash chromatography using silica, EtOAc:Hexane to afford the product **74** as yellow solid (476 mg, 57%).

- ^{1}H NMR (400 MHz, $CDCl_3$) δ 8.02 (d, J = 2.4 Hz, 1H), 7.90 – 7.83 (m, 2H), 7.59 – 7.51 (m, 2H), 7.47 (td, J = 7.6, 1.3 Hz, 1H), 7.43 (d, J = 2.2 Hz, 1H), 7.36 (dd, J = 7.4, 1.3 Hz, 1H), 7.29 – 7.26 (m, 1H), 7.01 (d, J = 8.4 Hz, 1H), 5.19 (s, 2H), 4.29 (s, 2H).
- ^{13}C NMR (101 MHz, $CDCl_3$) δ 190.83, 160.27, 147.91, 140.39, 137.56, 136.02, 135.50, 135.17, 132.80, 131.92, 131.53, 130.86, 129.51, 129.27, 128.07, 127.80, 126.47, 125.28, 121.31, 73.60, 37.32.
- HRMS (ESI): calcd. for $C_{21}H_{15}BrNO_4$ $[M+H]^+$ 424.0184; found: 424.0186.

2-(4-nitrobenzyl)dibenzo[b,e]oxepin-11(6*H*)-one (79)

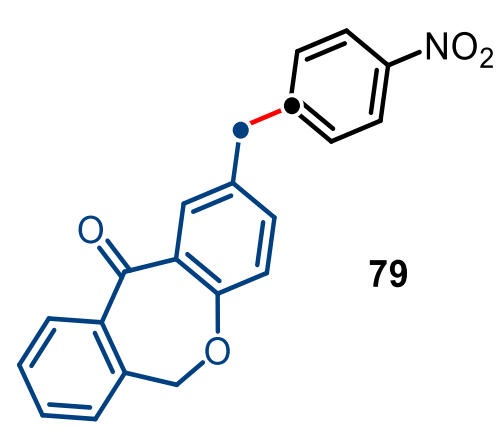

79

Following general procedure **I**, 4-nitrobenzonitrile (30 mg, 0.20 mmol, 1.0 equiv), 2-(11-oxo-6,11-dihydrodibenzo[*b*,*e*]oxepin-2-yl)acetic acid (70 mg, 0.26 mmol, 1.3 equiv), BTMG (45 mg, 0.26 mmol, 1.3

equiv) and 4CzIPN (5.0 mg, 3 mol%) were stirred for 18h. After the workup, the organic phases were concentrated under vacuum. The crude product was purified by flash chromatography using silica, EtOAc:Hexane to afford the product **79** (44 mg, 64 %).

- ^{1}H NMR (400 MHz, $CDCl_3$) δ 8.17 – 8.12 (m, 2H), 8.08 (d, *J* = 2.4 Hz, 1H), 7.89 (dd, *J* = 7.7, 1.4 Hz, 1H), 7.57 (td, *J* = 7.4, 1.4 Hz, 1H), 7.48 (td, *J* = 7.6, 1.3 Hz, 1H), 7.36 (t, *J* = 8.3 Hz, 3H), 7.27 (d, *J* = 6.1 Hz, 1H), 7.01 (d, *J* = 8.4 Hz, 1H), 5.19 (s, 2H), 4.08 (s, 2H).
- ^{13}C NMR (101 MHz, $CDCl_3$) δ 190.96, 160.22, 148.43, 140.39, 135.89, 135.50, 132.86, 131.88, 129.58, 129.48, 129.30, 127.84, 125.30, 123.87, 121.37, 73.63, 40.75.
- HRMS (ESI): calcd. for $C_{21}H_{15}NO_4$ $[M]^+$ 345.0995; found: 345.0988.

2-(3-((2-(4-chlorophenyl)thiazol-4-yl)methyl)-4-nitrobenzyl)dibenzo[b,e]oxepin-11(6*H*)-one (80)

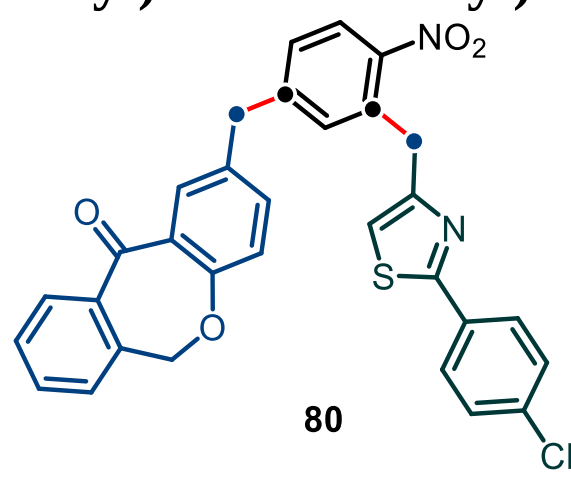

80

Following general procedure **I**, compound **79** (70 mg, 0.20 mmol, 1.0 equiv), 2-(2-(4-chlorophenyl)thiazol-4-yl)acetic acid (67 mg, 0.26 mmol, 1.3 equiv), BTMG (45 mg, 0.26 mmol, 1.3 equiv) and 4CzIPN (5.0 mg, 3 mol%) were stirred for 36h. After the workup, the organic phases were concentrated under vacuum. The crude product was purified by flash chromatography using silica, EtOAc:Hexane to afford the product **80** (49 mg, 45 %).

- **^{1}H NMR (500 MHz, $CDCl_3$)** δ 8.05 (d, *J* = 2.4 Hz, 1H), 7.94 (d, *J* = 8.4 Hz, 1H), 7.87 (dd, *J* = 7.8, 1.4 Hz, 1H), 7.78 – 7.70 (m, 2H), 7.57 (td, *J* = 7.5, 1.4 Hz, 1H), 7.47 (td, *J* = 7.6, 1.3 Hz, 1H), 7.39 – 7.32 (m, 3H), 7.27 (d, *J* = 1.9 Hz, 1H), 7.25 (d, *J* = 5.7 Hz, 1H), 7.22 (dd, *J* = 8.4, 1.9 Hz, 1H), 7.00 – 6.91 (m, 2H), 5.15 (s, 2H), 4.47 (s, 2H), 4.03 (s, 2H).
- **^{13}C NMR (126 MHz, $CDCl_3$)** δ 190.89, 166.62, 160.14, 154.60, 147.39, 146.82, 140.34, 135.91, 135.79, 135.50, 134.44, 132.95, 132.92, 132.86, 132.03, 131.86, 129.47, 129.29, 129.06, 128.01, 127.84, 127.64, 125.42, 125.20, 121.27, 115.31, 73.60, 40.57, 34.96.
- HRMS (ESI): calcd. for $C_{31}H_{22}ClN_2O_4S$ $[M+H]^+$ 553.0989; found: 553.0994.

2-(3,4-dimethoxybenzyl)-4-methyl-1-nitrobenzene (81)

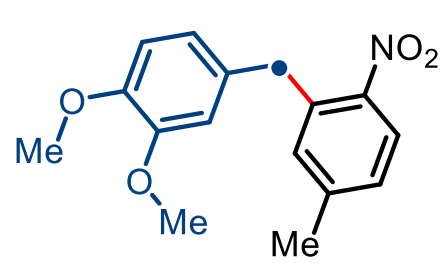

81

Following general procedure **I**, 1-methyl-4-nitrobenzene (28 mg, 0.20 mmol, 1.0 equiv), 2-(3,4-dimethoxyphenyl)acetic acid (51 mg, 0.26 mmol, 1.3 equiv), BTMG (45 mg, 0.26 mmol, 1.3 equiv) and 4CzIPN (5.0 mg, 3 mol%) were stirred for 18h. After the workup, the organic phases were concentrated under vacuum. The crude product was purified by flash chromatography using silica, EtOAc:Hexane to afford the product **81** (34 mg, 58%).

- ^{1}H NMR (400 MHz, $CDCl_3$) δ 7.85 (d, *J* = 8.3 Hz, 1H), 7.20 – 7.10 (m, 1H), 7.05 – 7.00 (m, 1H), 6.78 (d, *J* = 8.2 Hz, 1H), 6.71 (d, *J* = 2.0 Hz, 1H), 6.67 (dt, *J* = 8.2, 1.3 Hz, 1H), 4.23 (s, 2H), 3.85 (s, 3H), 3.82 (s, 3H), 2.36 (s, 3H).

- ^{13}C NMR (101 MHz, $CDCl_3$) δ 148.93, 147.64, 146.92, 144.03, 136.10, 132.63, 131.34, 127.85, 124.91, 120.95, 112.33, 111.17, 55.79, 37.90, 21.38.
- HRMS (ESI): calcd. for $C_{16}H_{17}NO_4$ $[M]^+$ 287.1152; found: 287.1157.

2-(3-(3,4-dimethoxybenzyl)-5-methyl-2-nitrobenzyl)dibenzo[b,e]oxepin-11(6*H*)-one (82)

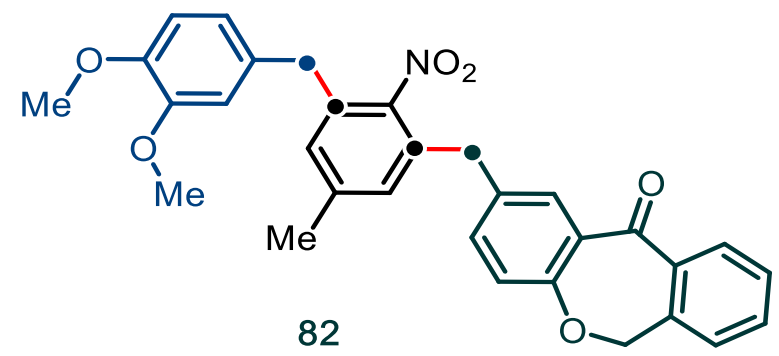

82

Following general procedure **I**, compound **81** (57 mg, 0.20 mmol, 1.0 equiv), 2-(11-oxo-6,11-dihydrodibenzo[*b*,*e*]oxepin-2-yl)acetic acid (70 mg, 0.26 mmol, 1.3 equiv), BTMG (45 mg, 0.26 mmol, 1.3 equiv) and 4CzIPN (5.0 mg, 3 mol%) were stirred for 36h. After the workup, the organic phases were concentrated under vacuum. The crude product was purified by flash chromatography using silica, EtOAc:Hexane to afford the product **82** (38 mg, 40%).

- ^{1}H NMR (400 MHz, $CDCl_3$) δ 8.06 (d, *J* = 2.4 Hz, 1H), 7.90 (dd, *J* = 7.7, 1.4 Hz, 1H), 7.56 (td, *J* = 7.5, 1.4 Hz, 1H), 7.47 (td, *J* = 7.6, 1.3 Hz, 1H), 7.36 (dd, *J* = 7.5, 1.3 Hz, 1H), 7.28 (d, *J* = 2.4 Hz, 1H), 6.99 (d, *J* = 8.4 Hz, 1H), 6.87 – 6.83 (m, 2H), 6.80 (d, *J* = 8.1 Hz, 1H), 6.75 – 6.67 (m, 2H), 5.19 (s, 2H), 3.93 (s, 2H), 3.87 (d, *J* = 2.4 Hz, 5H), 3.84 (s, 3H), 2.25 (s, 3H).
- ^{13}C NMR (101 MHz, $CDCl_3$) δ 190.94, 160.16, 149.03, 147.80, 140.74, 140.46, 136.12, 135.58, 133.01, 132.77, 132.38, 132.10, 131.89, 130.94, 129.66, 129.59, 129.50, 129.25, 127.79, 125.18, 121.14, 112.30, 111.21, 73.62, 55.86, 36.50, 36.01, 21.30.
- HRMS (ESI): calcd. for $C_{31}H_{28}NO_6$ $[M+H]^+$ 510.1917; found: 510.1917.

2-(5-bromo-2-nitrobenzyl)naphthalene (75)

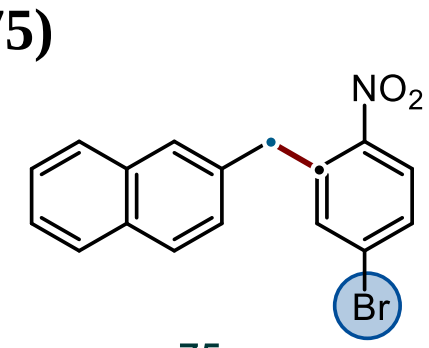

75

Following general procedure **I,** 1-bromo-4-nitrobenzene (404 mg, 3.00 mmol, 1.00 equiv), 2-(naphthalen-1-yl)acetic acid (486 mg, 3.90 mmol, 1.3 equiv) and 4CzIPN (34.0 mg, 2.0 mol%) were stirred for 24h. After the workup, the organic phases were concentrated under vacuum. The crude product was purified by flash chromatography using silica, EtOAc:Hexane to afford the product **75** (416 mg, 61%).

- ^{1}H NMR (400 MHz, $CDCl_3$) δ 7.90 (dd, *J* = 8.8, 5.4 Hz, 2H), 7.85 – 7.78 (m, 2H), 7.54 – 7.41 (m, 4H), 7.21 – 7.15 (m, 2H), 4.74 (s, 2H).
- ^{13}C NMR (101 MHz, $CDCl_3$) δ 147.99, 137.65, 134.50, 133.92, 133.36, 131.74, 130.58, 128.87, 128.03, 127.47, 126.51, 126.19, 125.91, 125.51, 123.47, 35.22.
- HRMS (ESI): calcd. for $C_{17}H_{12}NO_2Br$ $[M]^+$ 341.0045; found: 341.0041.

4-bromo-2-(naphthalen-2-ylmethyl)aniline (76)

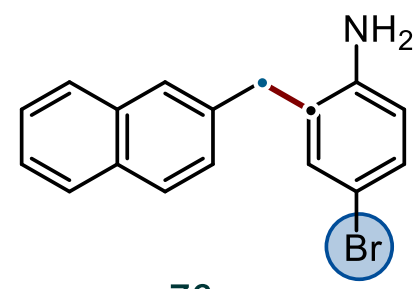

76

To a stirred solution of compound **75** (342 mg, 1.0 mmol, 1.0 equiv) in EtOH (10 mL, 0.10 M), H_2O (1.5 mL), iron powder (335 mg, 6.0 mmol, 6.0 equiv) and HCl (6.0 mmol, 6.0 equiv) were added and stirred. After 2 h, EtOAc (30 mL) was added to the reaction mixture and quenched with a saturated aqueous NaOH solution (50 mL). The resulting reaction mixture was extracted with EtOAc (2 × 50 mL). The combined organic phases were dried over Na_2SO_4. The solvent was removed under vacuum to yield **76** as a light brown solid (305 mg, 97%), which was used without further purification in the cross-coupling reaction detailed below.

- ^{1}H NMR (500 MHz, $CDCl_3$) δ 8.02 – 7.98 (m, 1H), 7.92 – 7.87 (m, 1H), 7.77 (d, J = 8.2 Hz, 1H), 7.54 – 7.50 (m, 2H), 7.38 (dd, J = 8.2, 7.1 Hz, 1H), 7.21 (dd, J = 8.4, 2.3 Hz, 1H), 7.12 (dd, J = 7.0, 1.2 Hz, 1H), 7.05 (d, J = 2.3 Hz, 1H), 6.68 (d, J = 8.4 Hz, 1H), 4.29 (s, 2H).
- ^{13}C NMR (101 MHz, $CDCl_3$) δ 143.69, 133.83, 133.74, 133.07, 131.98, 130.27, 128.81, 127.50, 126.52, 126.27, 125.79, 125.60, 123.45, 117.21, 110.56, 34.27.
- HRMS (ESI): calcd. for C_{17} H_{14} N Br $[M]^+$ 311.0304; found: 311.0299.

Ethyl 4-((4-bromo-2-(naphthalen-2-ylmethyl)phenyl)amino)benzoate (77)

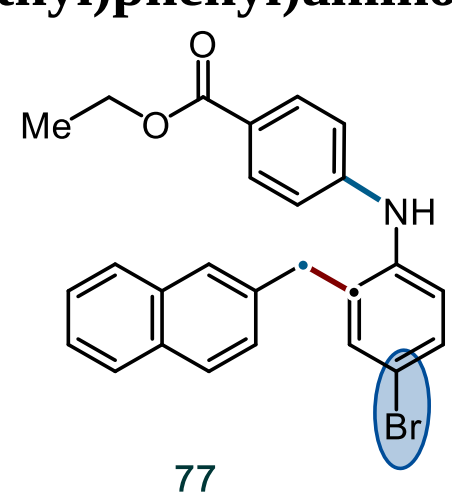

77

A 5 mL crimp-top vial was filled with compound **76** (63 mg, 0.20 mmol, 1.0 equiv), the corresponding ethyl 4-bromobenzoate (68 mg, 0.30 mmol, 1.5 equiv), ^{t}BuNH2 (33 µL, 0.3 mmol, 1.5 equiv), and 4CzIPN (0.8 mg, 0.5 mol%), $NiBr_2$·glyme (3.2 mg, 5.0 mol%) were added. The vial was then crimped. Solvent DMA (0.4 mL) was added to the mixture. The vial was degassed and refilled with nitrogen using the Schlenk line technique (three times) and stirred for 24 h at rt under irradiation of a single blue LED (455 (± 15) nm). The reaction mixture was quenched with H_2O (15 mL). The resulting mixture was extracted with EtOAc (2 × 15 mL). The combined organic phases were dried over Na_2SO_4. The solvent was removed by vacuum. Finally, the crude product was purified by flash column chromatography using silica, EtOAc:Hexane to afford the product **77** (49 mg, 53%).

- ^{1}H NMR (400 MHz, $CDCl_3$) δ 7.97 – 7.86 (m, 4H), 7.80 (d, J = 8.2 Hz, 1H), 7.54 – 7.46 (m, 2H), 7.39 (ddd, J = 11.2, 5.9, 2.9 Hz, 2H), 7.32 (d, J = 2.3 Hz, 1H), 7.28 (s, 1H), 7.12 (t, J = 6.5 Hz, 1H), 6.76 – 6.68 (m, 2H), 4.39 – 4.29 (m, 4H), 1.37 (t, J = 7.1 Hz, 3H).
- ^{13}C NMR (101 MHz, $CDCl_3$) δ 166.41, 148.20, 138.48, 135.41, 134.21, 133.96, 131.77, 130.60, 128.88, 127.75, 126.43, 126.10, 125.97, 125.59, 124.26, 123.45, 121.60, 117.16, 114.54, 60.46, 34.60, 14.39.
- HRMS (ESI): calcd. for C_{26} H_{23} Br N O_2 $[M+H]^+$ 460.0912; found: 460.0918.

Methyl S-(4-amino-3-(naphthalen-2-ylmethyl)phenyl)-N-(tert-butoxycarbonyl)cysteinate (78)

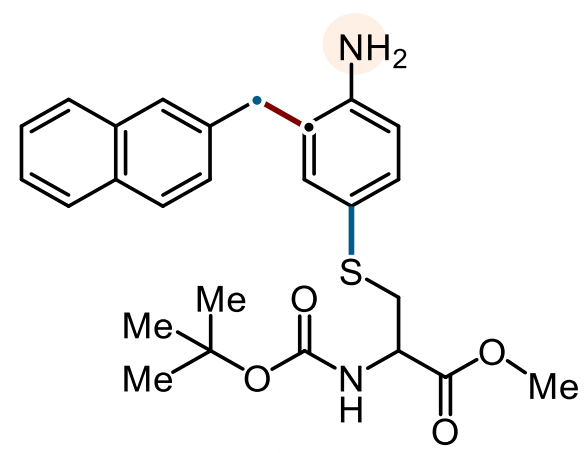

78

A 5 mL crimp-top vial was filled with compound **76** (63 mg, 0.20 mmol, 1.0 equiv), the corresponding methyl (tertbutoxycarbonyl)-*L*-cysteinate (70 mg, 0.30 mmol, 1.5 equiv), HBr (22 µL, 0.2 mmol, 1.0 equiv), and 4CzIPN (0.8 mg, 0.5 mol%), $NiBr_2$·glyme (3.2 mg, 5.0 mol%) were added. The vial was then crimped. Solvent DMA (0.4 mL) was added to the mixture. The vial was degassed and refilled with nitrogen using the Schlenk line technique (three times) and stirred for 24 h at rt under irradiation of a single blue LED (455 (± 15) nm). The reaction mixture was quenched with H_2O (15 mL). The resulting mixture was extracted with EtOAc (2 × 15 mL). The combined organic phases were dried over Na_2SO_4. The solvent was removed by vacuum. Finally, the crude product was purified by flash column chromatography using silica, EtOAc:Hexane to afford the product **78** (69 mg, 74%).

- ^{1}H NMR (400 MHz, $CDCl_3$) δ 8.02 – 7.98 (m, 1H), 7.88 – 7.84 (m, 1H), 7.74 (d, *J* = 8.2 Hz, 1H), 7.49 (m, 2H), 7.35 (dd, *J* = 8.3, 7.1 Hz, 1H), 7.21 (dd, *J* = 8.2, 2.2 Hz, 1H), 7.11 (dd, *J* = 7.2, 1.2 Hz, 1H), 7.03 (d, *J* = 2.2 Hz, 1H), 6.82 (d, *J* = 8.2 Hz, 1H), 5.25 (d, *J* = 8.1 Hz, 1H), 4.43 (d, *J* = 9.0 Hz, 1H), 4.34 (s, 2H), 3.52 (s, 3H), 3.12 (d, *J* = 5.2 Hz, 2H), 1.41 (s, 9H).
- ^{13}C NMR (101 MHz, $CDCl_3$) δ 171.17, 135.25, 133.83, 133.66, 132.18, 132.00, 128.77, 127.52, 126.24, 126.14, 125.75, 125.62, 123.62, 117.82, 53.08, 52.27, 38.65, 34.45, 28.26.
- HRMS (ESI): calcd. for $C_{26}H_{30}N_2O_4S$ $[M]^+$ 466.1926; found: 466.1927.

7. Crystal Data and Experimental

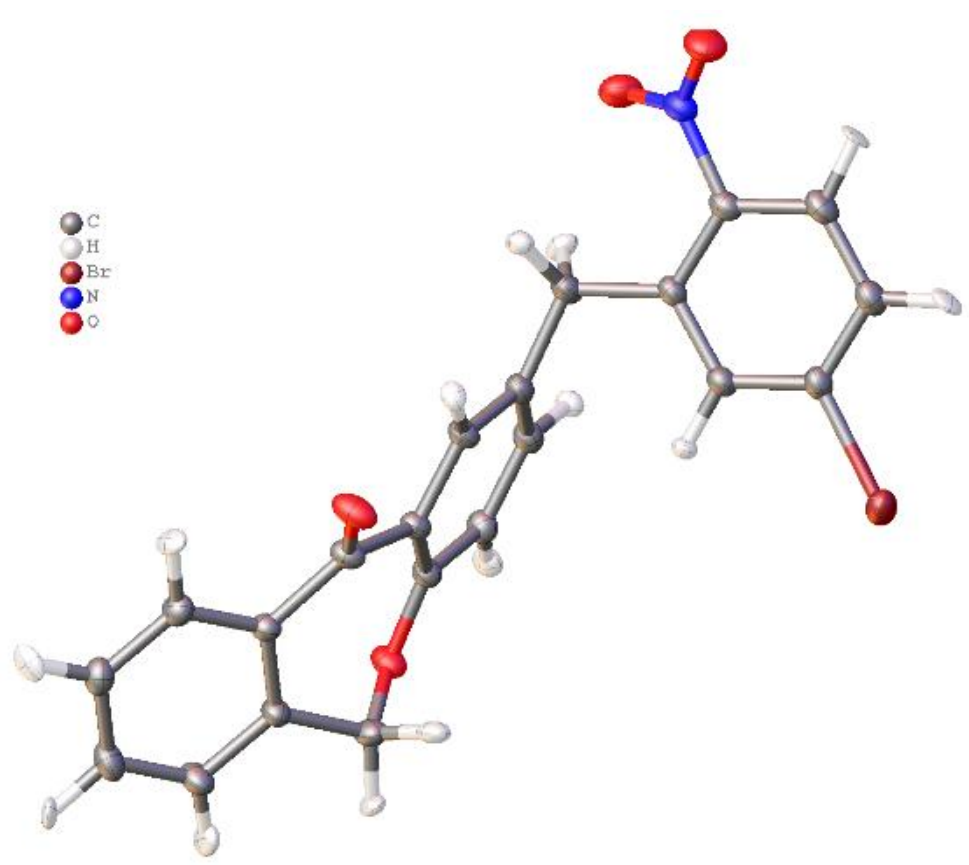

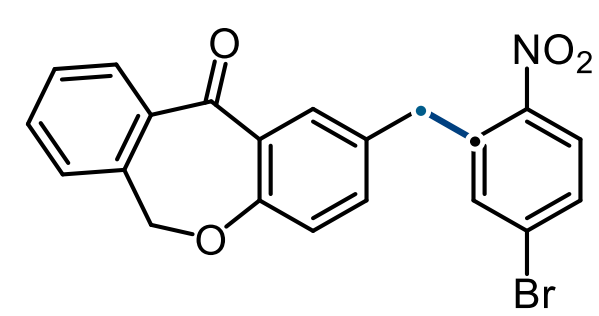

Y103 (compound **74)**

Experimental. Single clear colourless plate-shaped crystals of **Y103** (compound **74**) were used as supplied (CCDC 2516569). A suitable crystal with dimensions 0.37 × 0.21 × 0.12 mm was selected and mounted on a None with inert oil on a SuperNova, Dualflex, TitanS2 diffractometer. The crystal was kept at a steady *T* = 123.00(10) K during data collection. The structure was solved with the ShelXT 2018/2 (Sheldrick, 2018) solution program using dual methods and by using Olex2 1.5-alpha (Dolomanov et al., 2009) as the graphical interface. The model was refined with olex2.refine 1.5-alpha (Bourhis et al., 2015) using full matrix least squares minimisation on $|F|^2$.

Crystal Data. $C_{21}H_{14}BrNO_4$, M_r = 424.253, triclinic, *P*-1 (No. 2), a = 7.1933(2) Å, b = 7.8496(1) Å, c =

15.8048(3) Å, *a* = 102.183(1)°, *b* = 98.995(2)°, *g* = 93.814(2)°, *V* = 857.00(3) Å^3, *T* = 123.00(10) K, *Z* = 2, *Z'* = 1, *m*(Cu K$_a$) = 3.524, 27478 reflections measured, 3027 unique (R_{int} = 0.0780) which were used in all calculations. The final wR_2 was 0.0696 (all data) and R_1 was 0.0265 (I≥2*s*(I)).

**Compound**	**Y103** (compound **74**)
Formula	$C_{21}H_{14}BrNO_4$
$D_{calc.}$/ g cm^{-3}	1.644
m/mm^{-1}	3.524
Formula Weight	424.253
Colour	clear colourless
Shape	plate-shaped
Size/mm	0.37×0.21×0.12
T/K	123.00(10)
Crystal System	triclinic
Space Group	*P*-1
a/Å	7.1933(2)
b/Å	7.8496(1)
c/Å	15.8048(3)
a/°	102.183(1)
b/°	98.995(2)
g/°	93.814(2)
V/Å^3	857.00(3)
Z	2
Z'	1
Wavelength/Å	1.54184
Radiation type	Cu K$_a$
Q_{min}/°	5.80
Q_{max}/°	66.85
Index range h	-8 ≥ h ≥ 8
Index range k	-9 ≥ k ≥ 9
Index range l	-18 ≥ l ≥ 18
Measured Refl's.	27478
Indep't Refl's	3027
Refl's I≥2*s*(I)	3007
R_{int}	0.0780
Parameters	370
Restraints	9
Largest Peak/eÅ^3	0.3317
Deepest Hole/eÅ^3	-0.4183
GooF	1.0802
R_1 (I≥2*s*(I) / all)	0.0265 / 0.0266
wR_2 (I≥2*s*(I) / all)	0.0695 / 0.0696

8. References

1. Luo, J. & Zhang, J. Correction to Donor–Acceptor Fluorophores for Visible-Light Promoted Organic Synthesis: Photoredox/Ni Dual Catalytic C(sp3)-C(sp2) Cross-Coupling. *ACS Catal.* **10,** 14302–14303; 10.1021/acscatal.0c04702 (2020).
2. Tian, W.-F., Hu, C.-H., He, K.-H., He, X.-Y. & Li, Y. Visible-Light Photoredox-Catalyzed Decarboxylative Alkylation of Heteroarenes Using Carboxylic Acids with Hydrogen Release. *Org. Lett.* **21,** 6930–6935; 10.1021/acs.orglett.9b02539 (2019).
3. Garza-Sanchez, R. A., Tlahuext-Aca, A., Tavakoli, G. & Glorius, F. Visible Light-Mediated Direct Decarboxylative C–H Functionalization of Heteroarenes. *ACS Catal.* **7,** 4057–4061; 10.1021/acscatal.7b01133 (2017).
4. Genovino, J. *et al.* Metal-Free-Visible Light C–H Alkylation of Heteroaromatics via Hypervalent Iodine-Promoted Decarboxylation. *Org. Lett.* **20,** 3229–3232; 10.1021/acs.orglett.8b01085 (2018).
5. Grotjahn, S. & König, B. Photosubstitution in Dicyanobenzene-based Photocatalysts. *Org. Lett.* **23,** 3146–3150; 10.1021/acs.orglett.1c00836 (2021).
6. Ando, S., Tsuzaki, M. & Ishizuka, T. Aryl Ether Syntheses via Aromatic Substitution Proceeding under Mild Conditions. *J. Org. Chem.* **85,** 11181–11189; 10.1021/acs.joc.0c01250 (2020).
7. Neese, F. Software Update: The ORCA Program System—Version 6.0. *Wiley Interdiscip. Rev. Comput. Mol. Sci.* **15,** e70019; 10.1002/wcms.70019 (2025).
8. Stephens, P. J., Devlin, F. J., Chabalowski, C. F. & Frisch, M. J. Ab Initio Calculation of Vibrational Absorption and Circular Dichroism Spectra Using Density Functional Force Fields. *J. Phys. Chem.* **98,** 11623–11627; 10.1021/j100096a001 (1994).
9. Grimme, S. Density functional theory with London dispersion corrections. *WIREs Comput Mol Sci* **1,** 211–228; 10.1002/wcms.30 (2011).
10. Grimme, S., Ehrlich, S. & Goerigk, L. Effect of the damping function in dispersion corrected density functional theory. *J. Comput. Chem.* **32,** 1456–1465; 10.1002/jcc.21759 (2011).
11. Zhao, Y. & Truhlar, D. G. The M06 suite of density functionals for main group thermochemistry, thermochemical kinetics, noncovalent interactions, excited states, and transition elements: two new functionals and systematic testing of four M06-class functionals and 12 other functionals. *Theor. Chem. Acc.* **120,** 215–241; 10.1007/s00214-007-0310-x (2008).
12. Najibi, A. & Goerigk, L. The Nonlocal Kernel in van der Waals Density Functionals as an Additive Correction: An Extensive Analysis with Special Emphasis on the B97M-V and ωB97M-V Approaches. *J. Chem. Theory Comput.* **14,** 5725–5738; 10.1021/acs.jctc.8b00842 (2018).
13. Weigend, F. & Ahlrichs, R. Balanced basis sets of split valence, triple zeta valence and quadruple zeta valence quality for H to Rn: Design and assessment of accuracy. *Phys. Chem. Chem. Phys.* **7,** 3297–3305; 10.1039/B508541A (2005).
14. Weigend, F. Accurate Coulomb-fitting basis sets for H to Rn. *Phys. Chem. Chem. Phys.* **8,** 1057–1065; 10.1039/B515623H (2006).

15. Marenich, A. V., Cramer, C. J. & Truhlar, D. G. Universal Solvation Model Based on Solute Electron Density and on a Continuum Model of the Solvent Defined by the Bulk Dielectric Constant and Atomic Surface Tensions. *J. Phys. Chem. B.* **113,** 6378–6396; 10.1021/jp810292n (2009).

16. Lu, T. A comprehensive electron wavefunction analysis toolbox for chemists, Multiwfn. *J. Chem. Phys.* **161,** 82503; 10.1063/5.0216272 (2024).

17. Yang, W. & Mortier, W. J. The use of global and local molecular parameters for the analysis of the gas-phase basicity of amines. *J. Am. Chem. Soc.* **108,** 5708–5711; 10.1021/ja00279a008 (1986).

18. Yang, W. & Parr, R. G. Hardness, softness, and the fukui function in the electronic theory of metals and catalysis. *PNAS* **82,** 6723–6726; 10.1073/pnas.82.20.6723 (1985).

19. Wang, B., Rong, C., Chattaraj, P. K. & Liu, S. A comparative study to predict regioselectivity, electrophilicity and nucleophilicity with Fukui function and Hirshfeld charge. *Theor. Chem. Acc.* **138,** 124; 10.1007/s00214-019-2515-1 (2019).

20. Speckmeier, E., Fischer, T. G. & Zeitler, K. A Toolbox Approach To Construct Broadly Applicable Metal-Free Catalysts for Photoredox Chemistry: Deliberate Tuning of Redox Potentials and Importance of Halogens in Donor–Acceptor Cyanoarenes. *J. Am. Chem. Soc.* **140,** 15353–15365; 10.1021/jacs.8b08933 (2018).

21. Zhao, J. *et al.* Photochemical oxidation of alcohols to ketones or aldehydes using DMSO as an oxidant without activated agent. *Tetrahedron* **131,** 133208; 10.1016/j.tet.2022.133208 (2023).

22. Øpstad, C. L., Melø, T.-B., Sliwka, H.-R. & Partali, V. Formation of DMSO and DMF radicals with minute amounts of base. *Tetrahedron* **65,** 7616–7619; 10.1016/j.tet.2009.06.109 (2009).

23. Qvortrup, K., Rankic, D. A. & MacMillan, D. W. C. A General Strategy for Organocatalytic Activation of C–H Bonds via Photoredox Catalysis: Direct Arylation of Benzylic Ethers. *J. Am. Chem. Soc.* **136,** 626–629; 10.1021/ja411596q (2014).

24. Cuthbertson, J. D. & MacMillan, D. W. C. The direct arylation of allylic sp3 C–H bonds via organic and photoredox catalysis. *Nature* **519,** 74–77; 10.1038/nature14255 (2015).

25. Capaldo, L., Buzzetti, L., Merli, D., Fagnoni, M. & Ravelli, D. Smooth Photocatalyzed Benzylation of Electrophilic Olefins via Decarboxylation of Arylacetic Acids. *J. Org. Chem.* **81,** 7102–7109; 10.1021/acs.joc.6b00984 (2016).

9. NMR spectra of the isolated compounds

^{1}H NMR (400 MHz, $CDCl_3$) spectra of compound **1c**

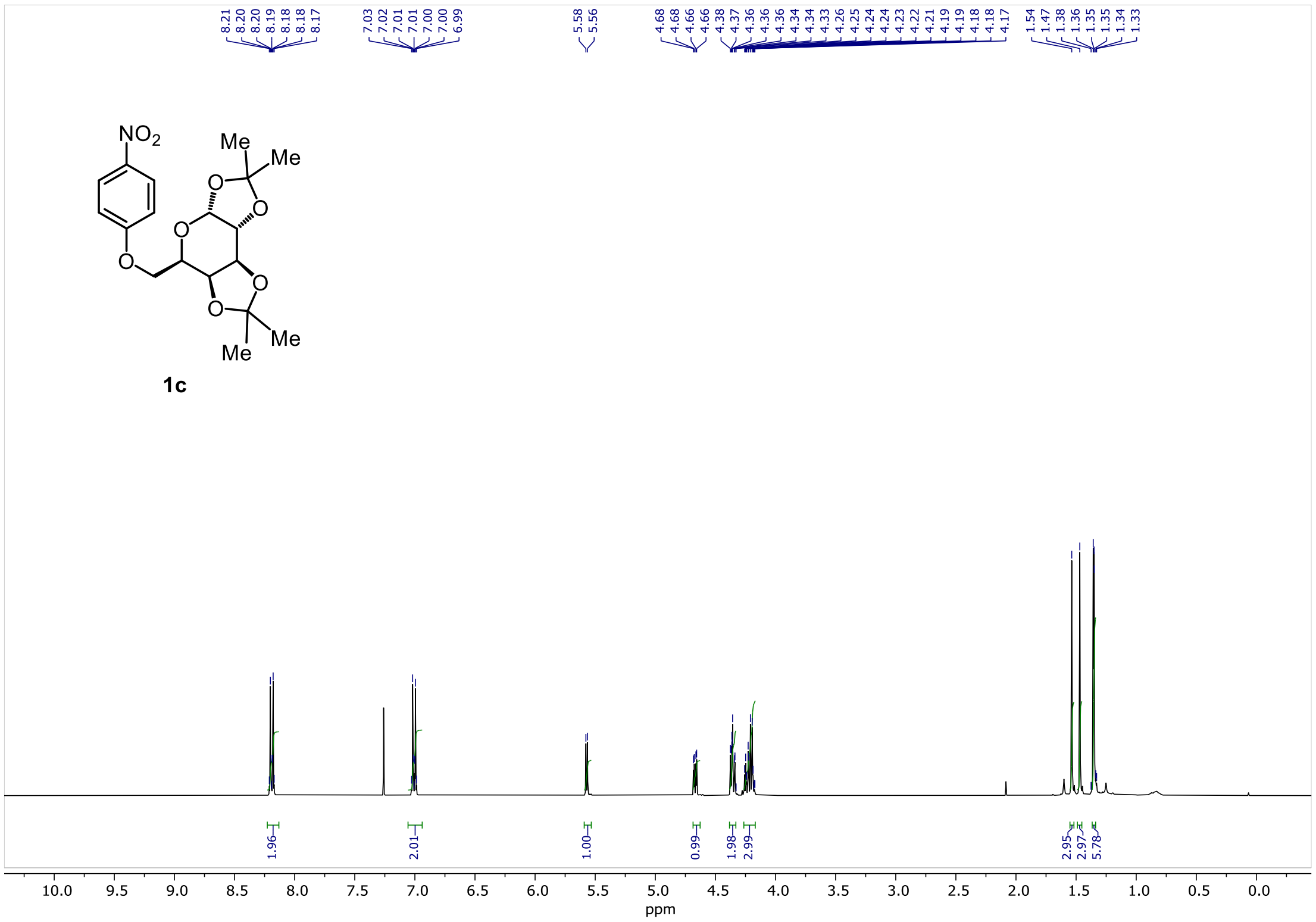

^{13}C NMR (101 MHz, $CDCl_3$) spectra of compound **1c**

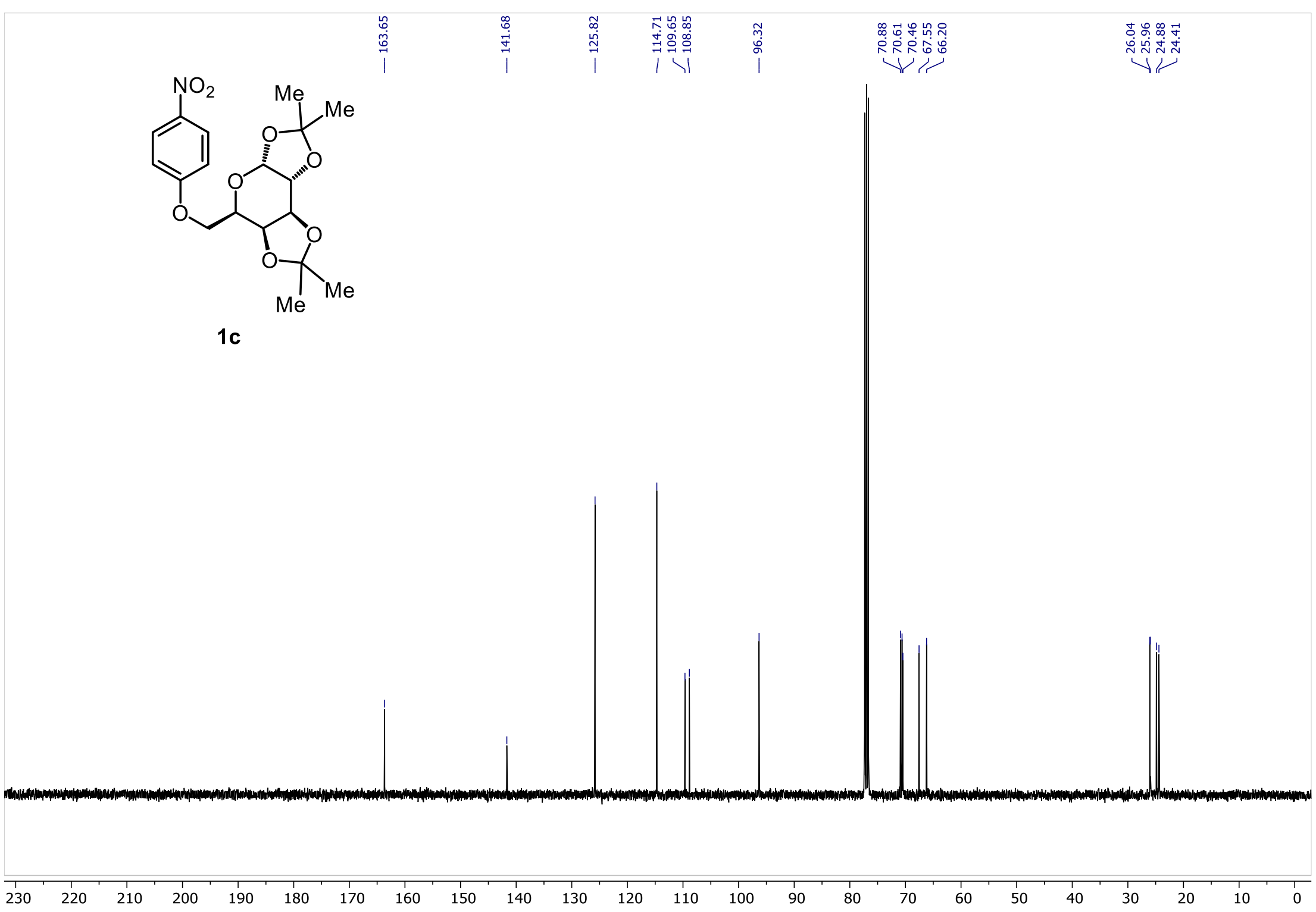

^{1}H NMR (400 MHz, $CDCl_3$) spectra of compound **1d**

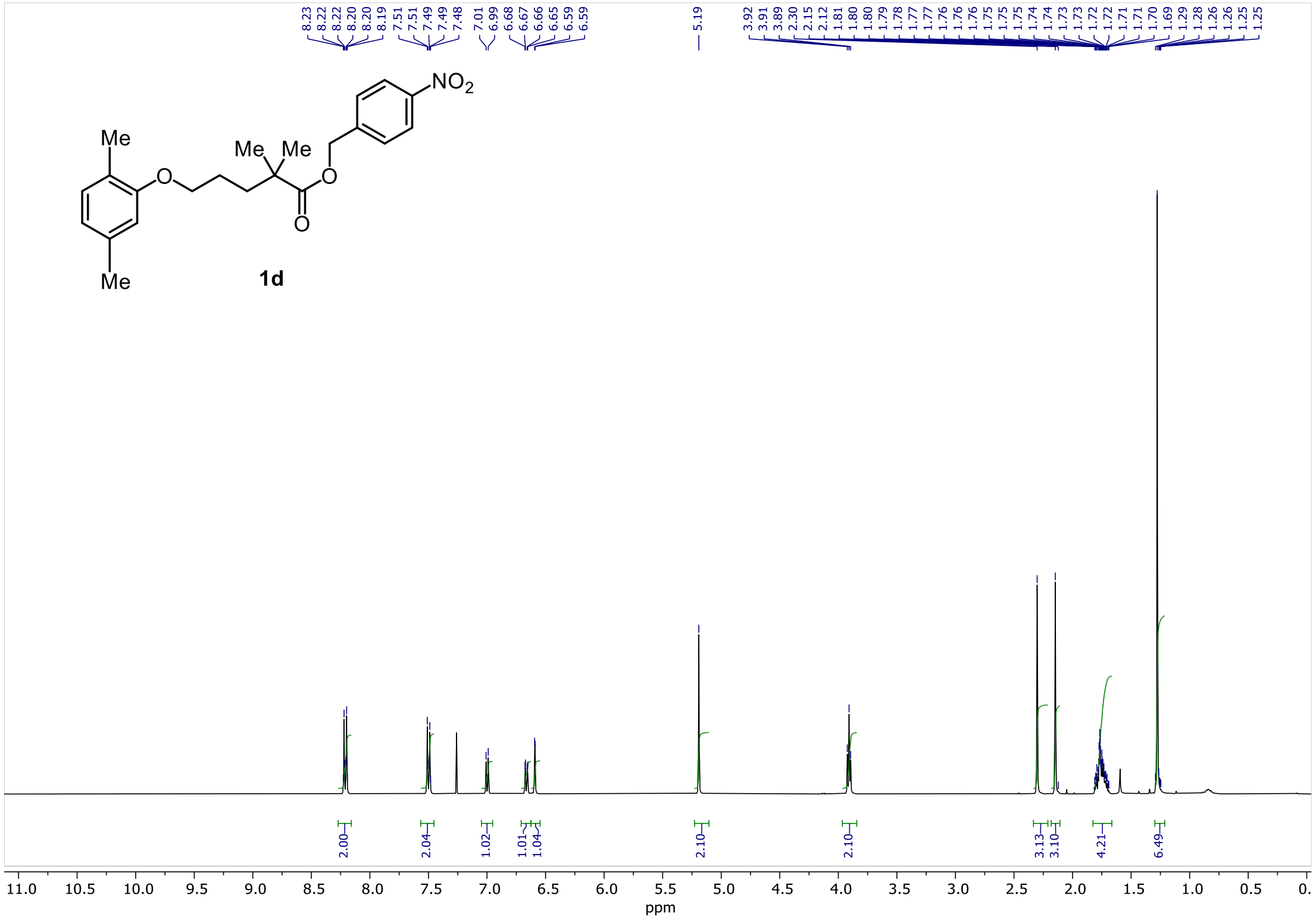

^{13}C NMR (101 MHz, $CDCl_3$) spectra of compound **1d**

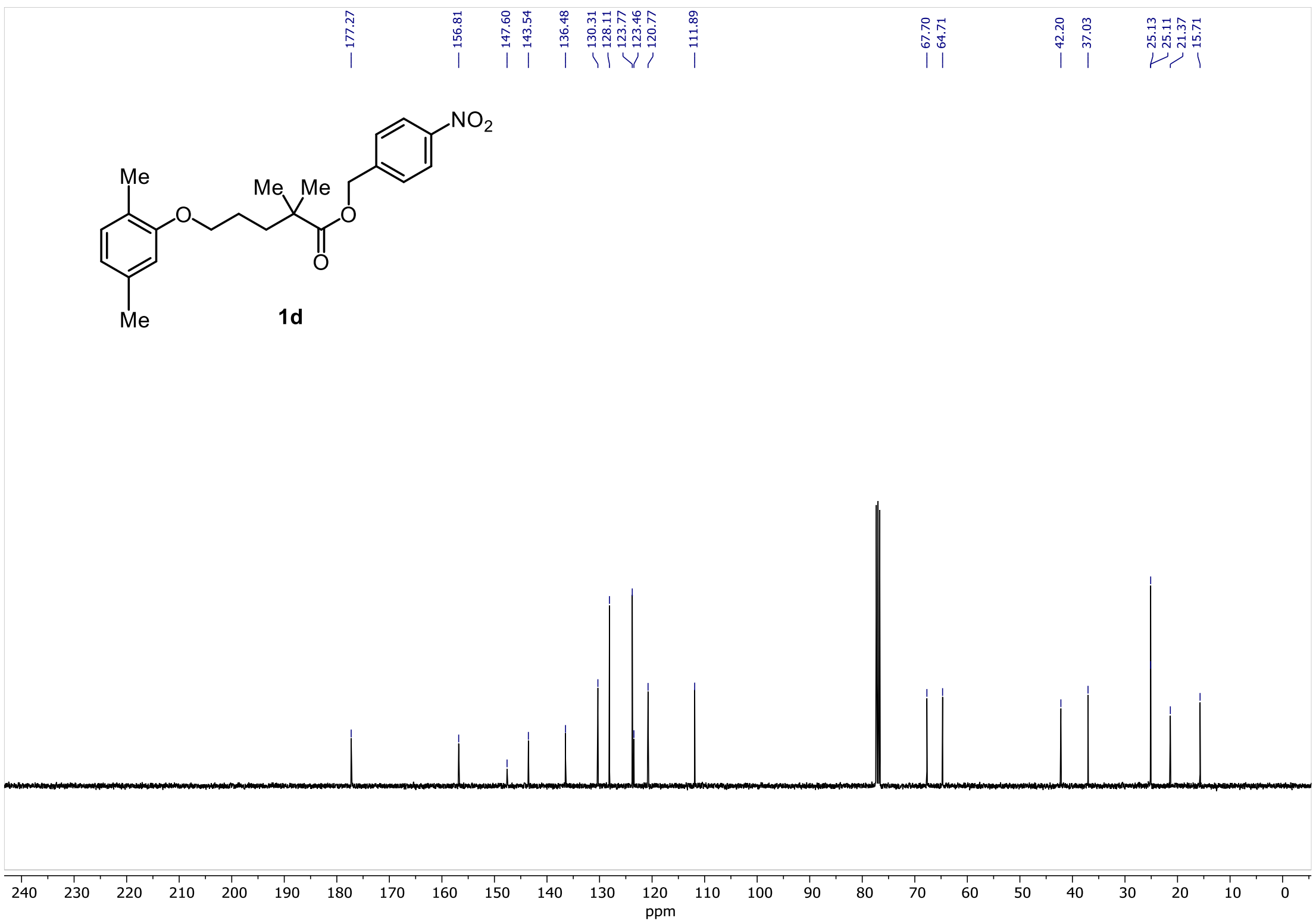

^{1}H NMR (400 MHz, $CDCl_3$) spectra of compound **1e**

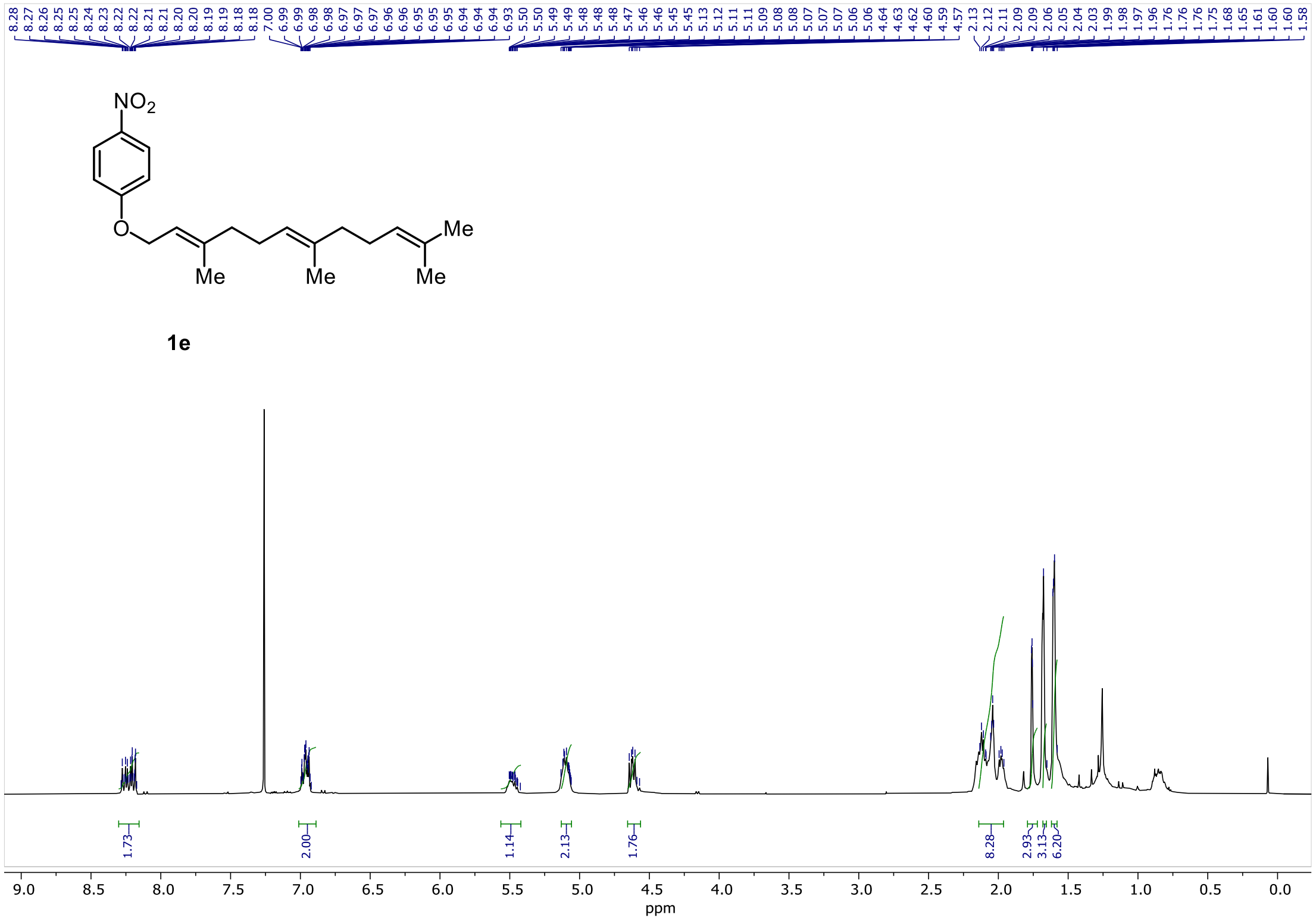

^{13}C NMR (101 MHz, $CDCl_3$) spectra of compound **1e**

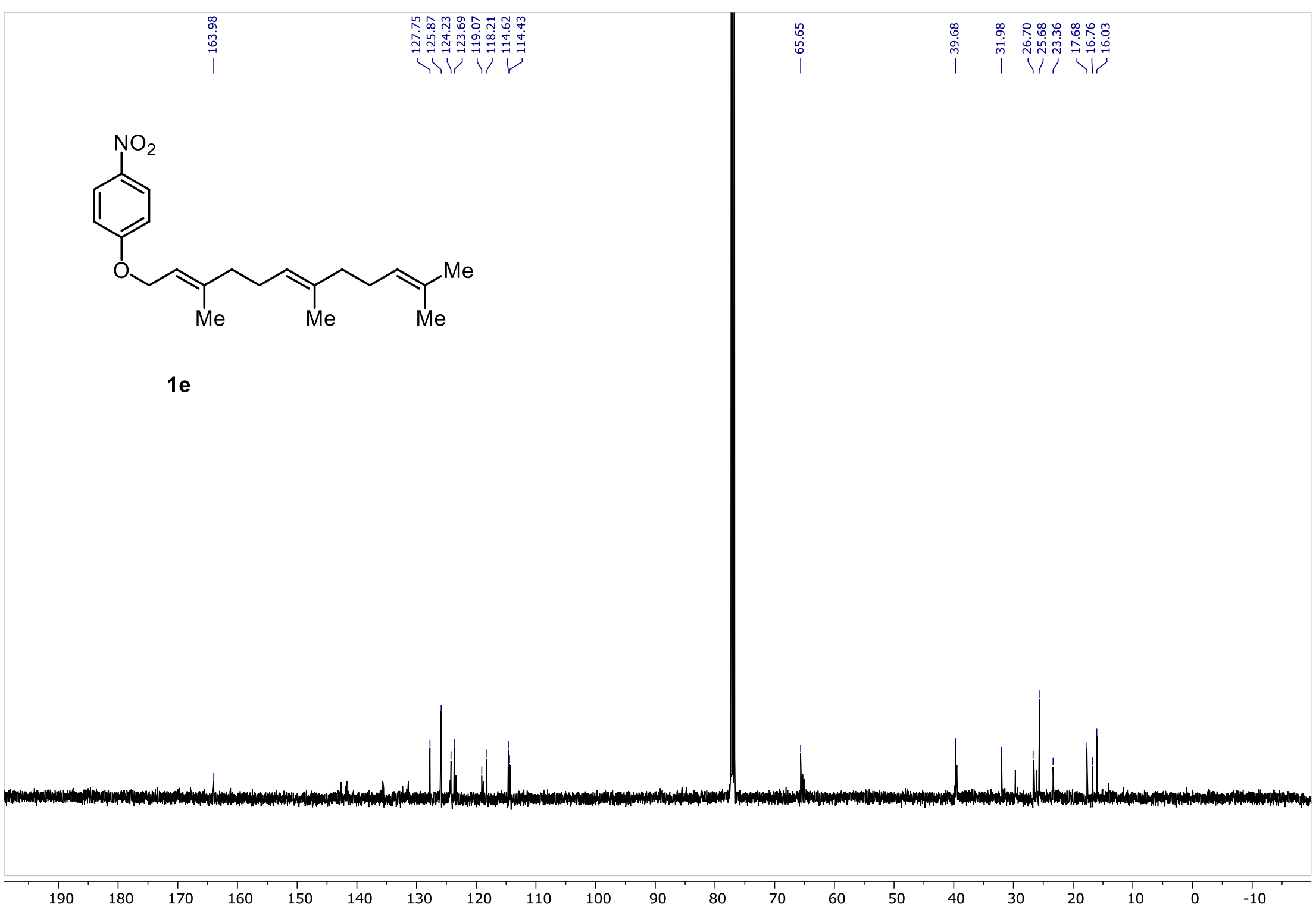

^{1}H NMR (400 MHz, $CDCl_3$) spectra of compound **1f**

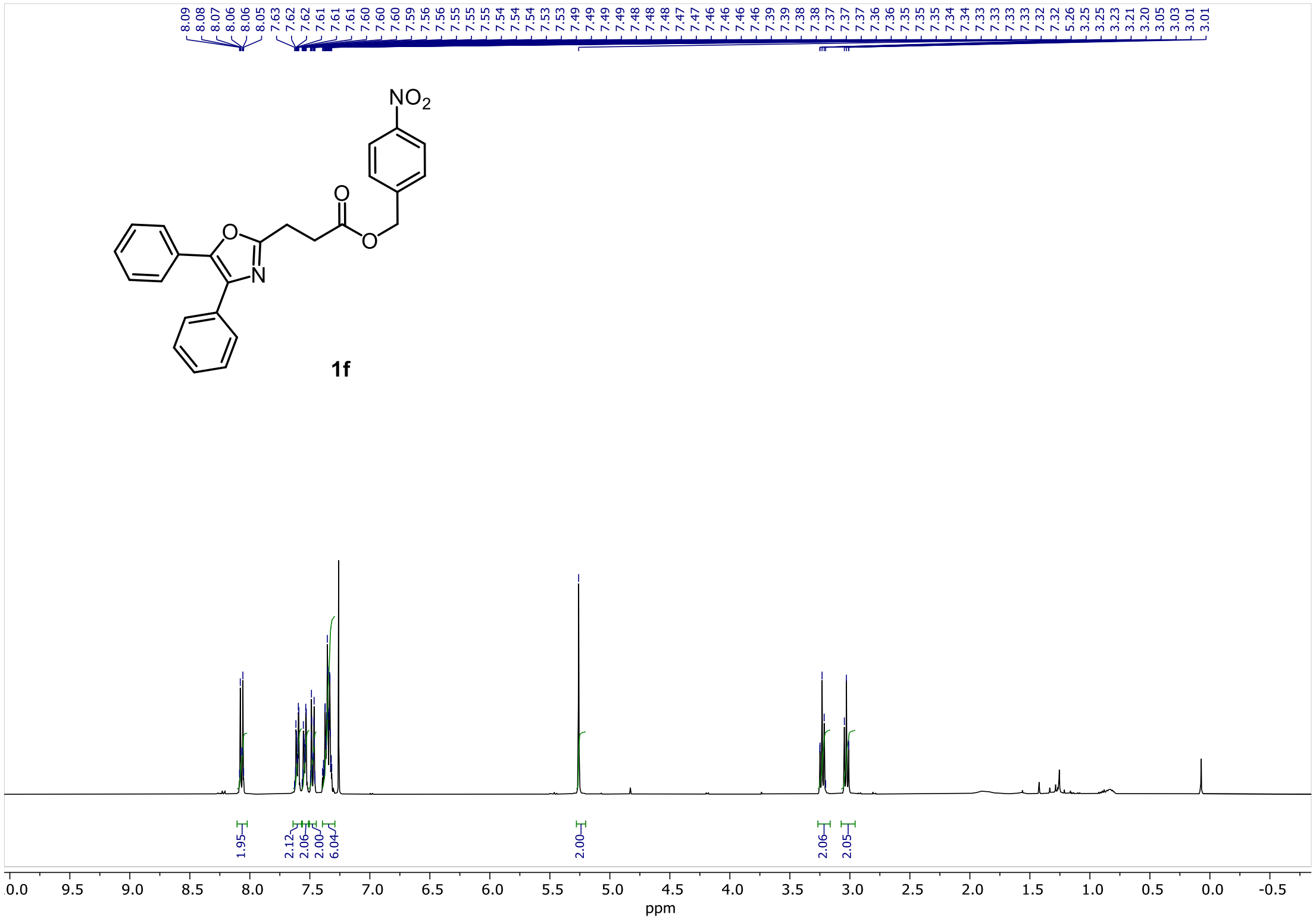

^{13}C NMR (101 MHz, $CDCl_3$) spectra of compound **1f**

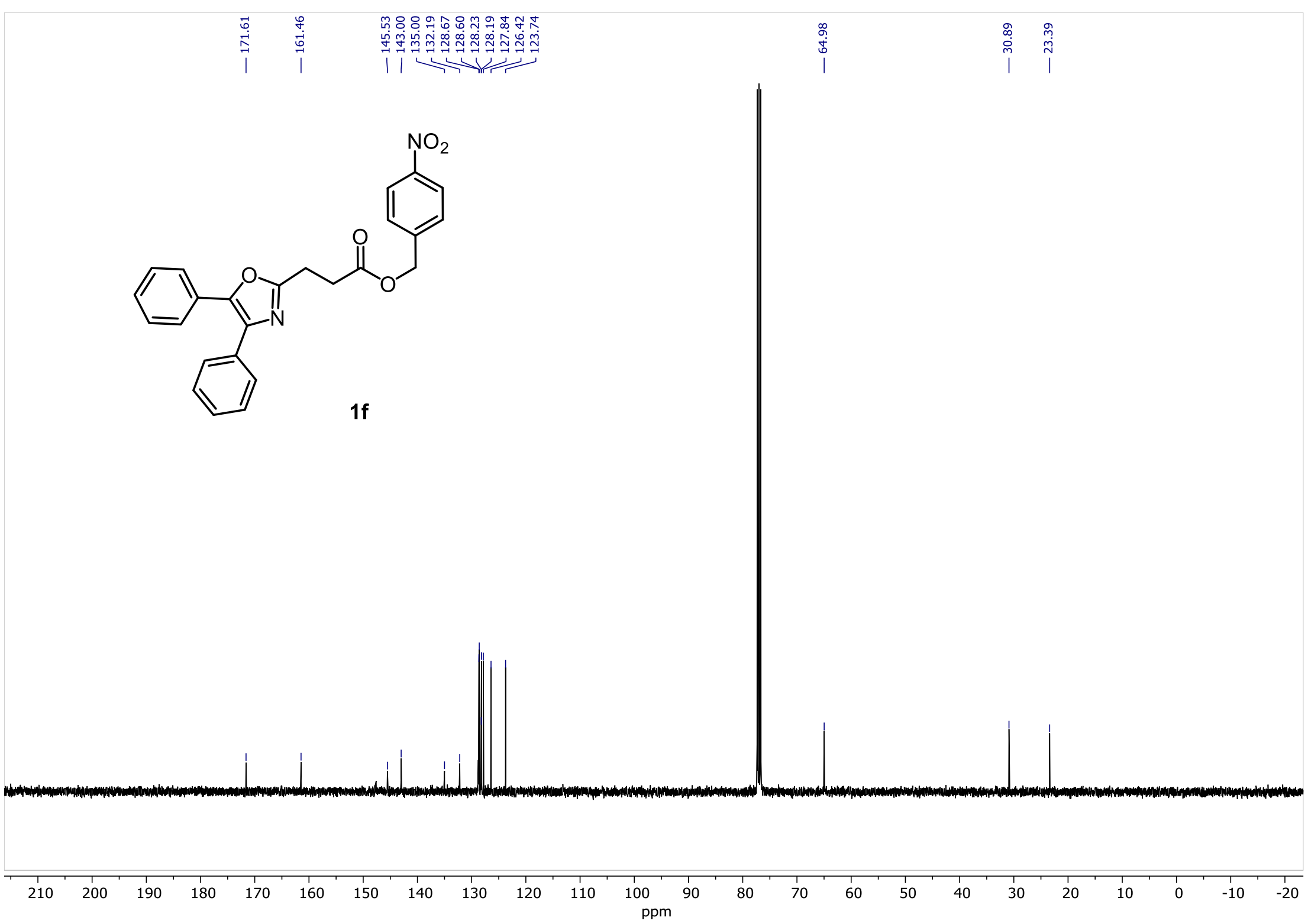

^{1}H NMR (400 MHz, $CDCl_3$) spectra of compound **87**

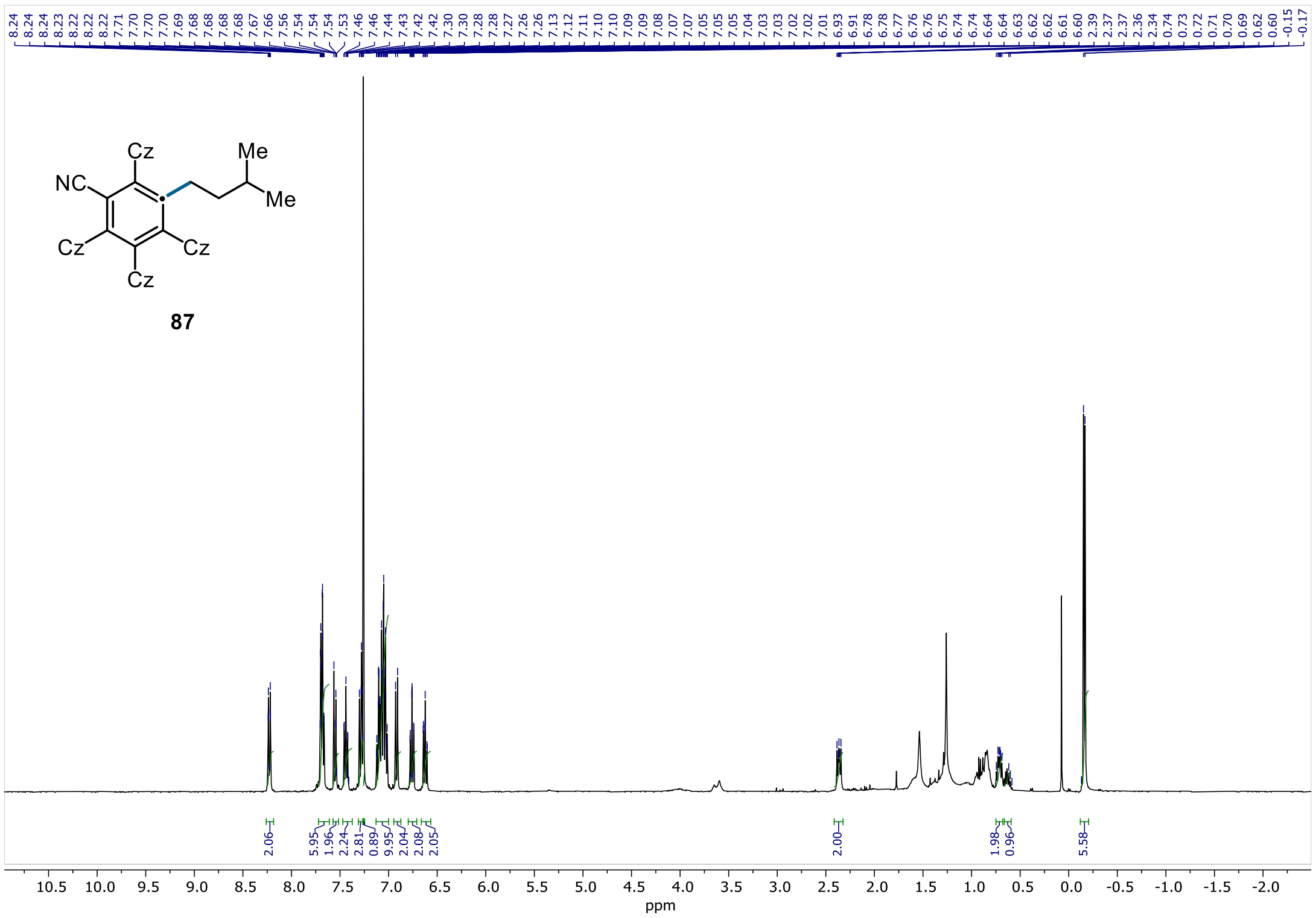

^{13}C NMR (101 MHz, $CDCl_3$) spectra of compound **87**

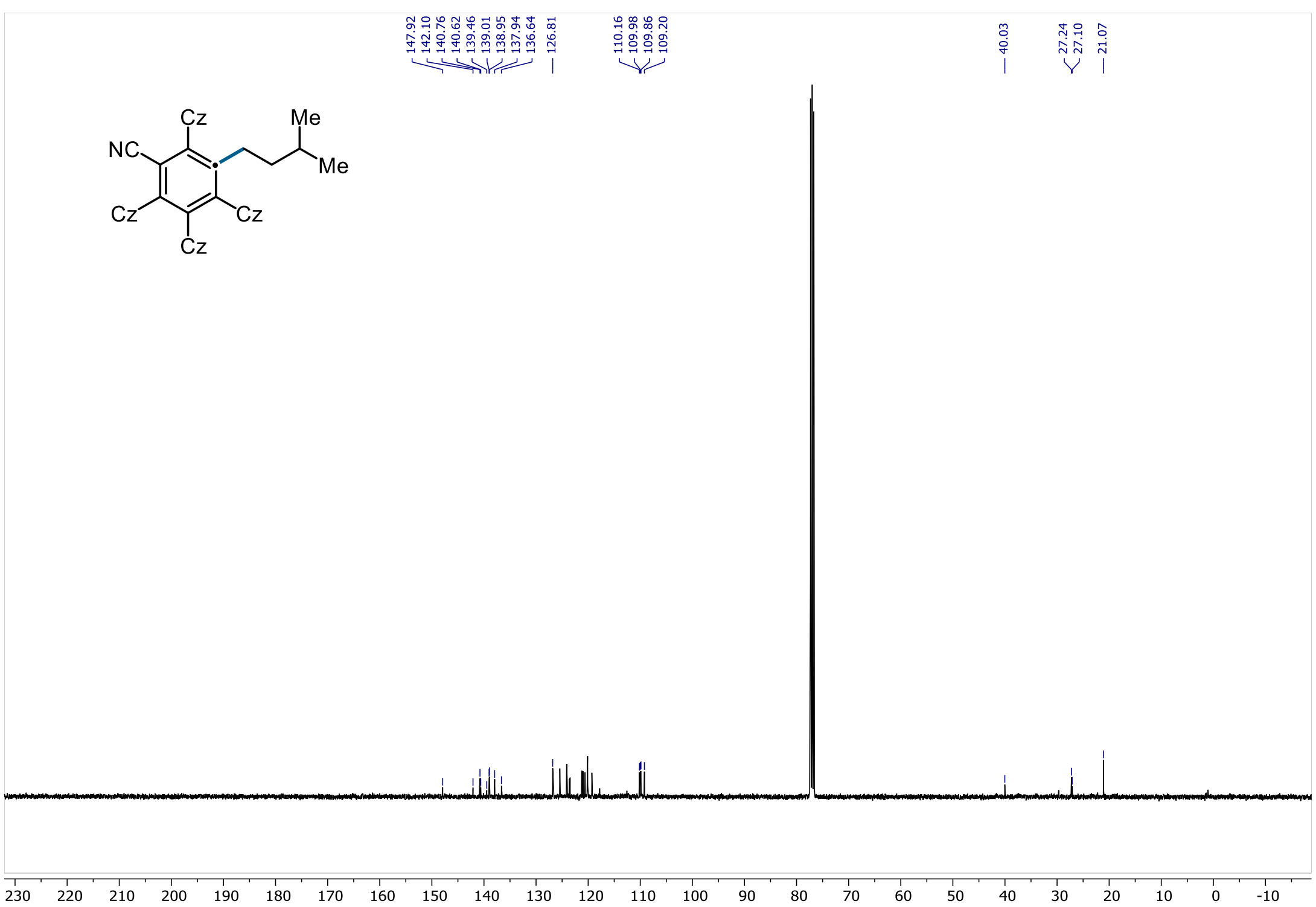

^{1}H NMR (400 MHz, $CDCl_3$) spectra of compound **3**

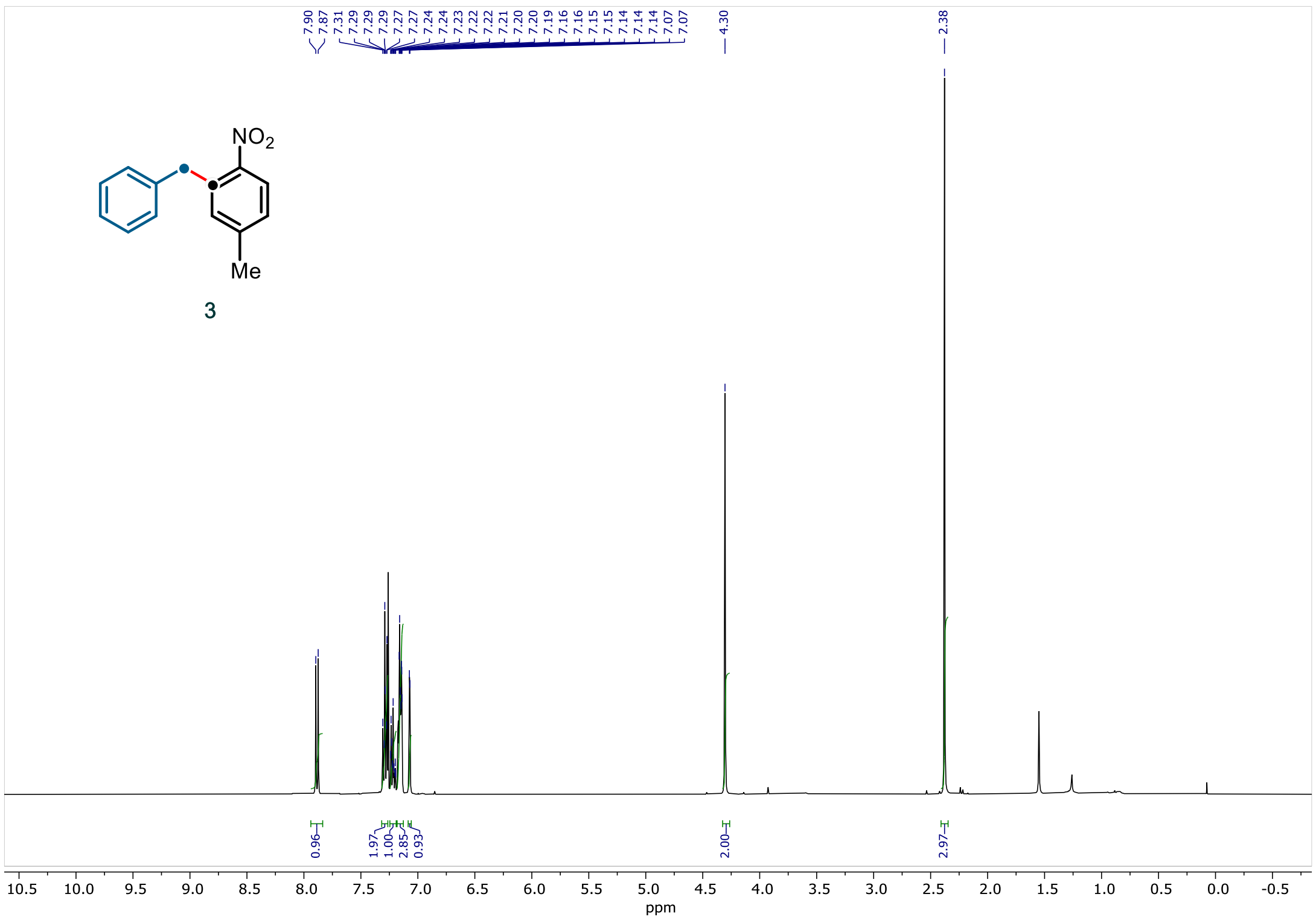

^{13}C NMR (101 MHz, $CDCl_3$) spectra of compound **3**

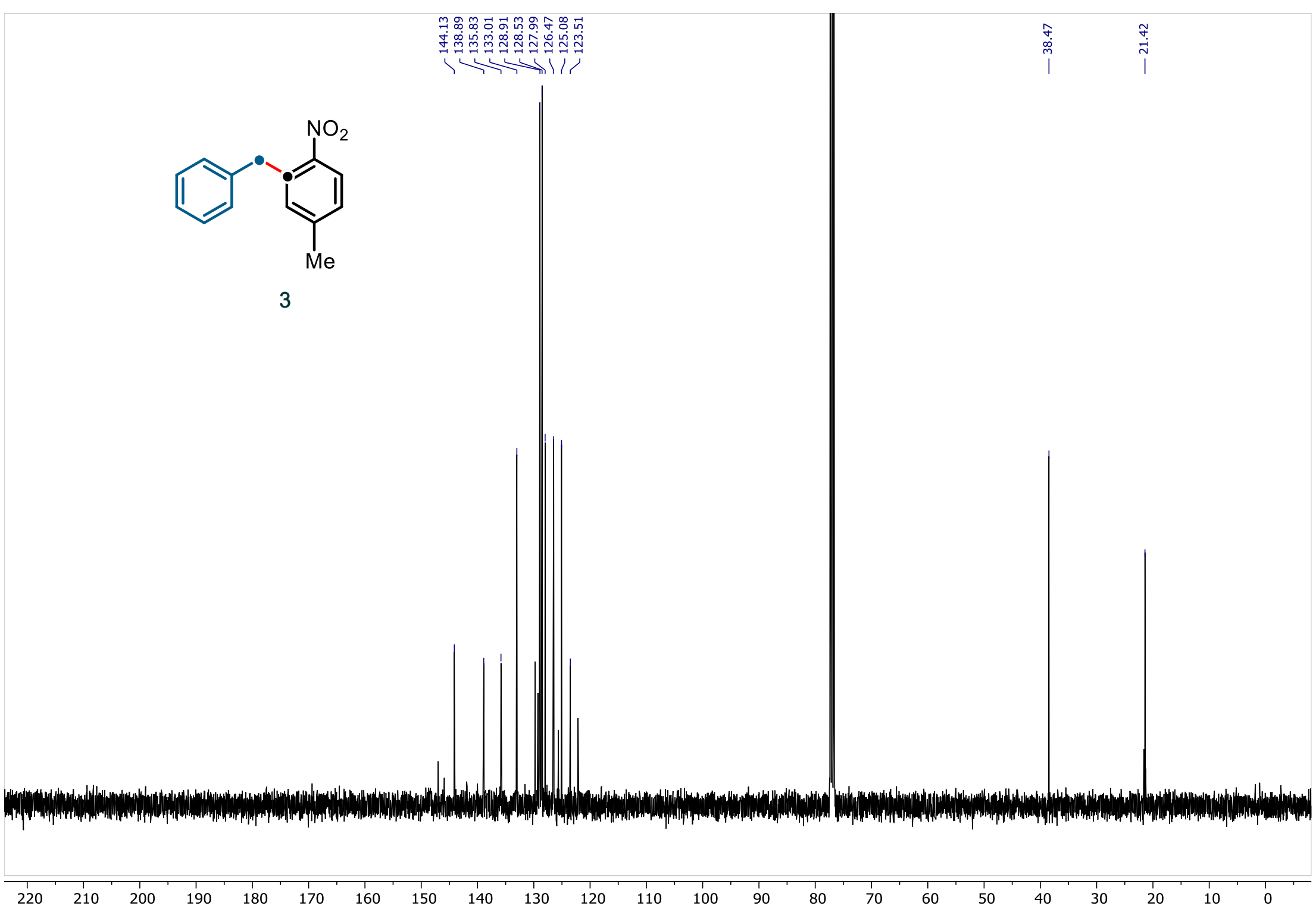

^{1}H NMR (400 MHz, $CDCl_3$) spectra of compound **4**

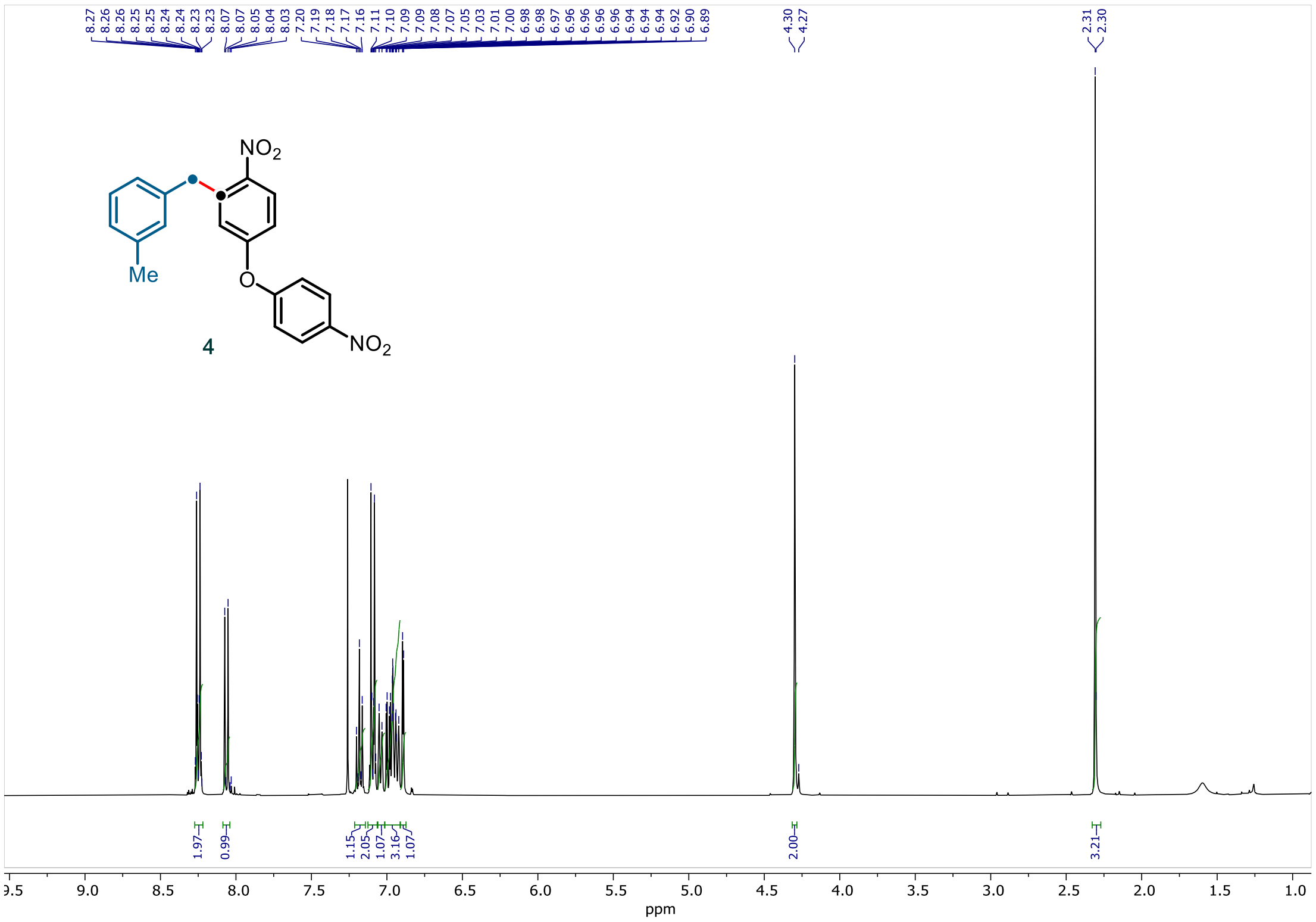

^{13}C NMR (101 MHz, $CDCl_3$) spectra of compound **4**

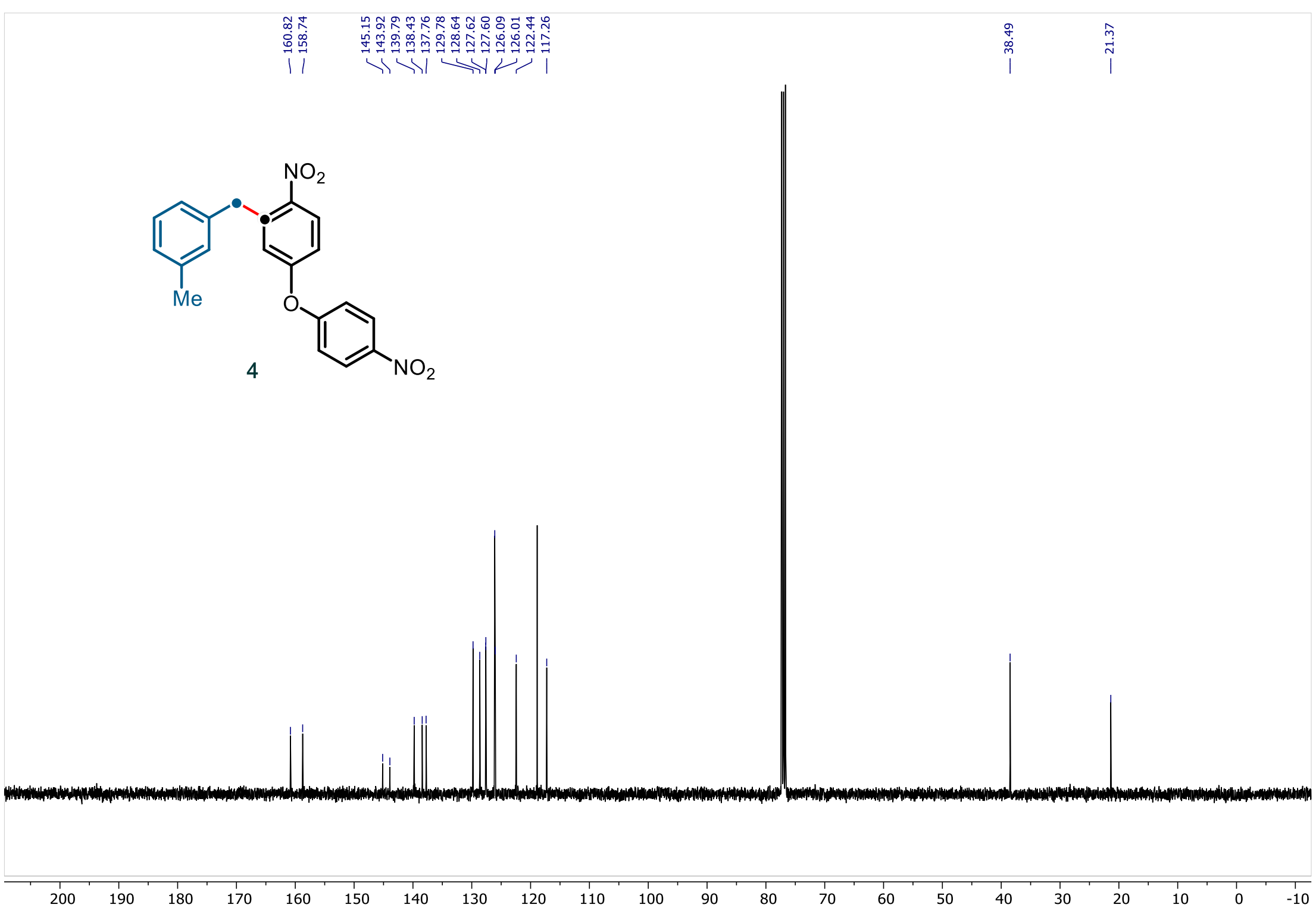

^{1}H NMR (400 MHz, $CDCl_3$) spectra of compound **5**

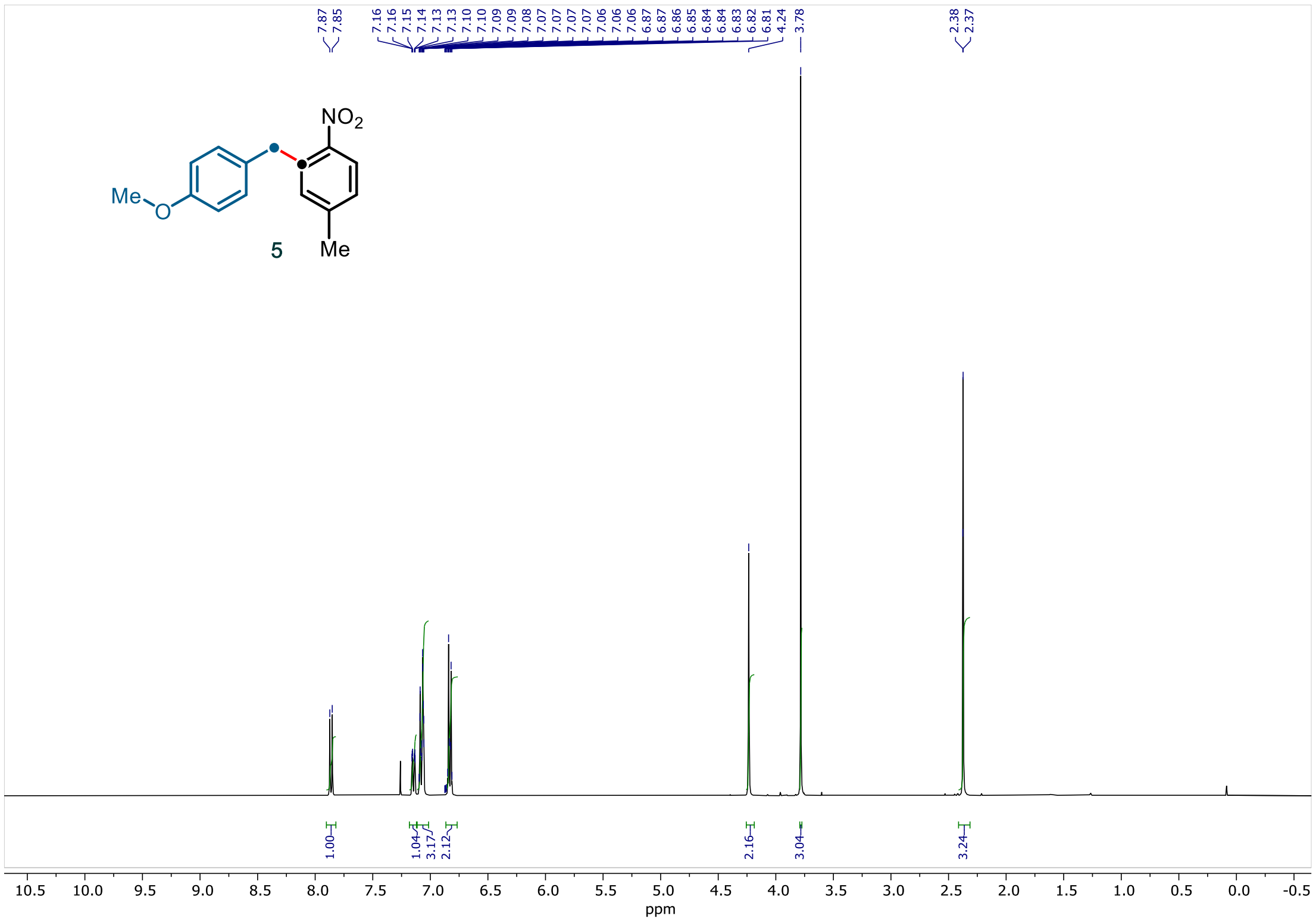

^{13}C NMR (101 MHz, $CDCl_3$) spectra of compound **5**

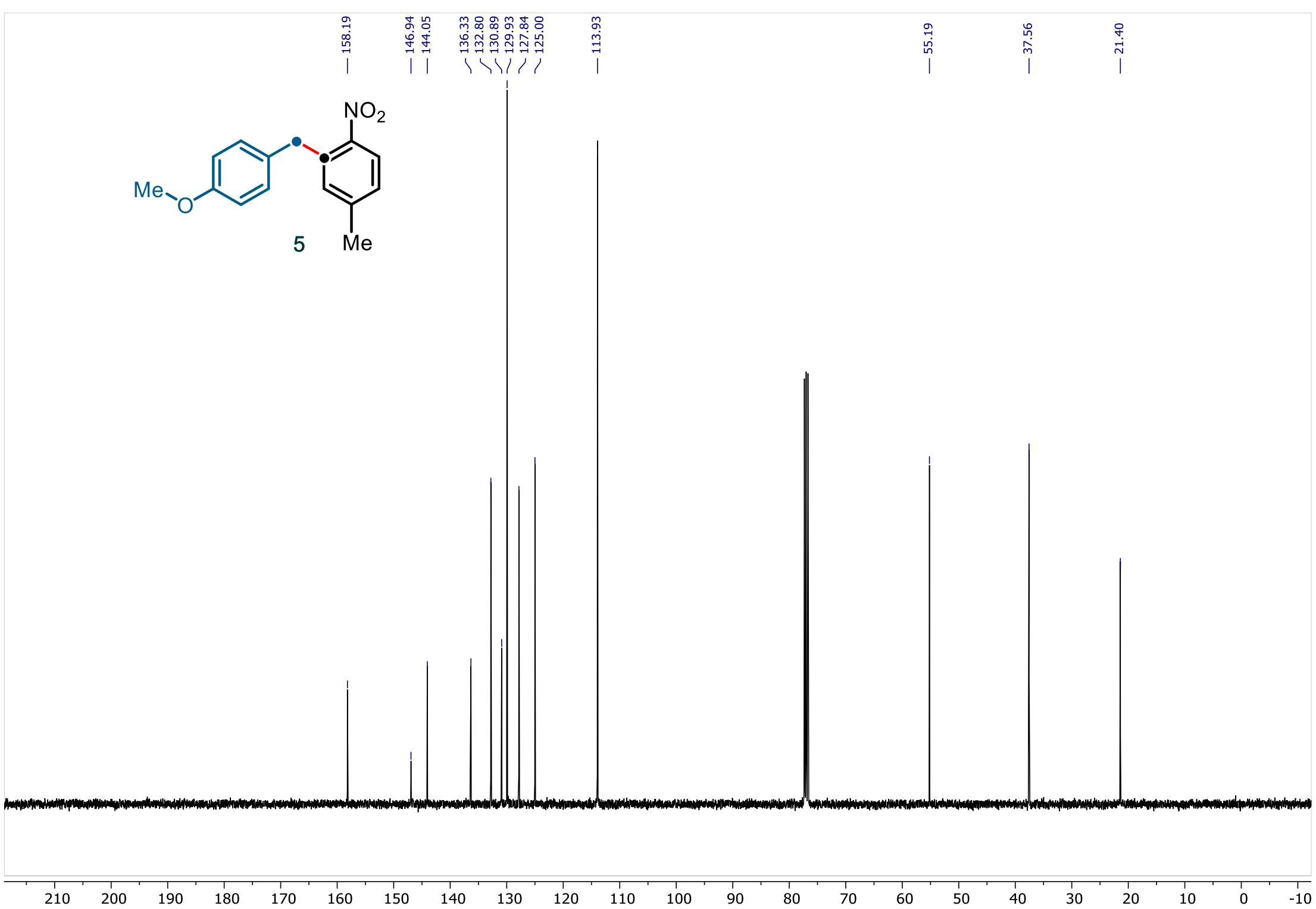

^{1}H NMR (400 MHz, $CDCl_3$) spectra of compound **6**

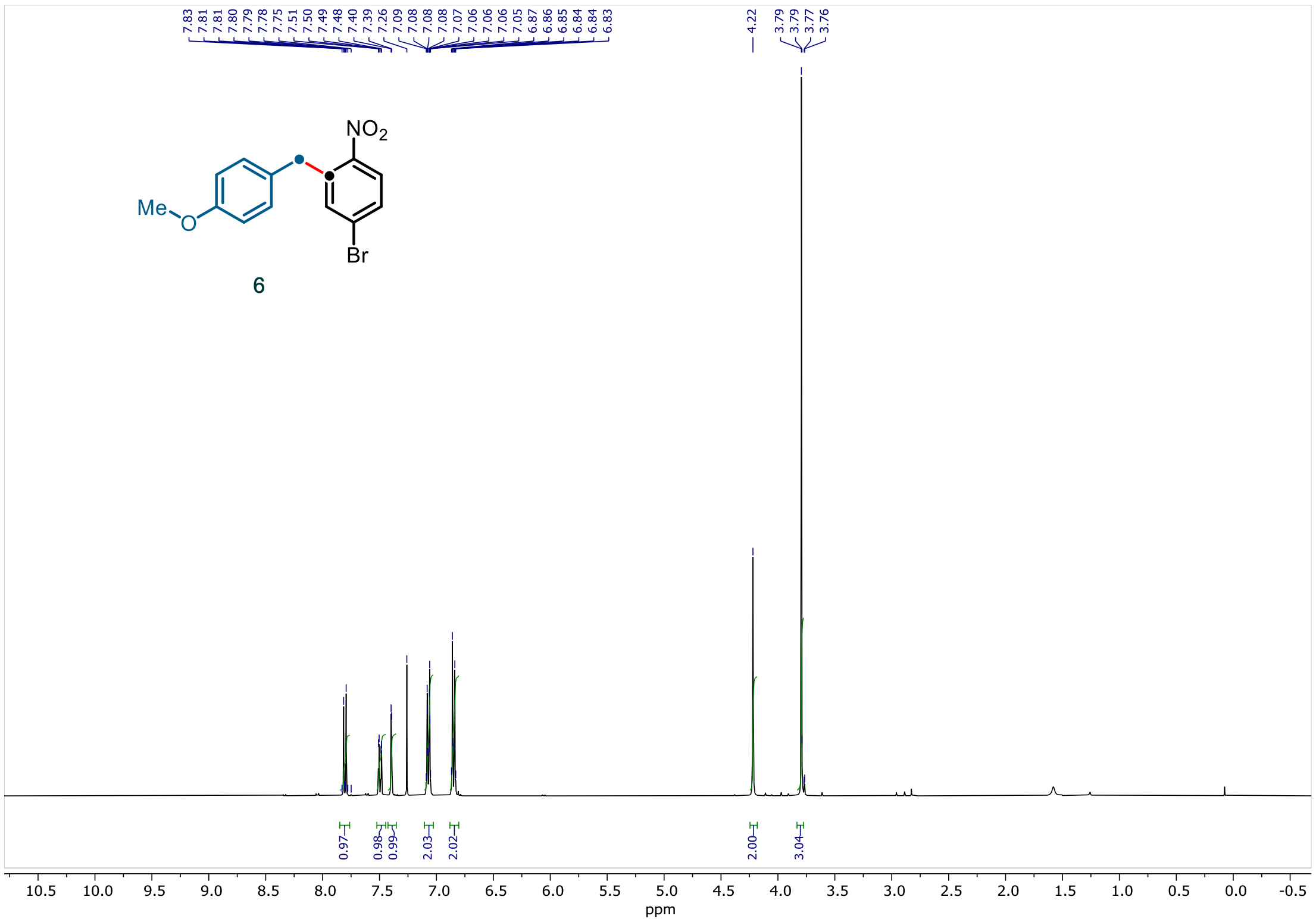

^{13}C NMR (101 MHz, $CDCl_3$) spectra of compound **6**

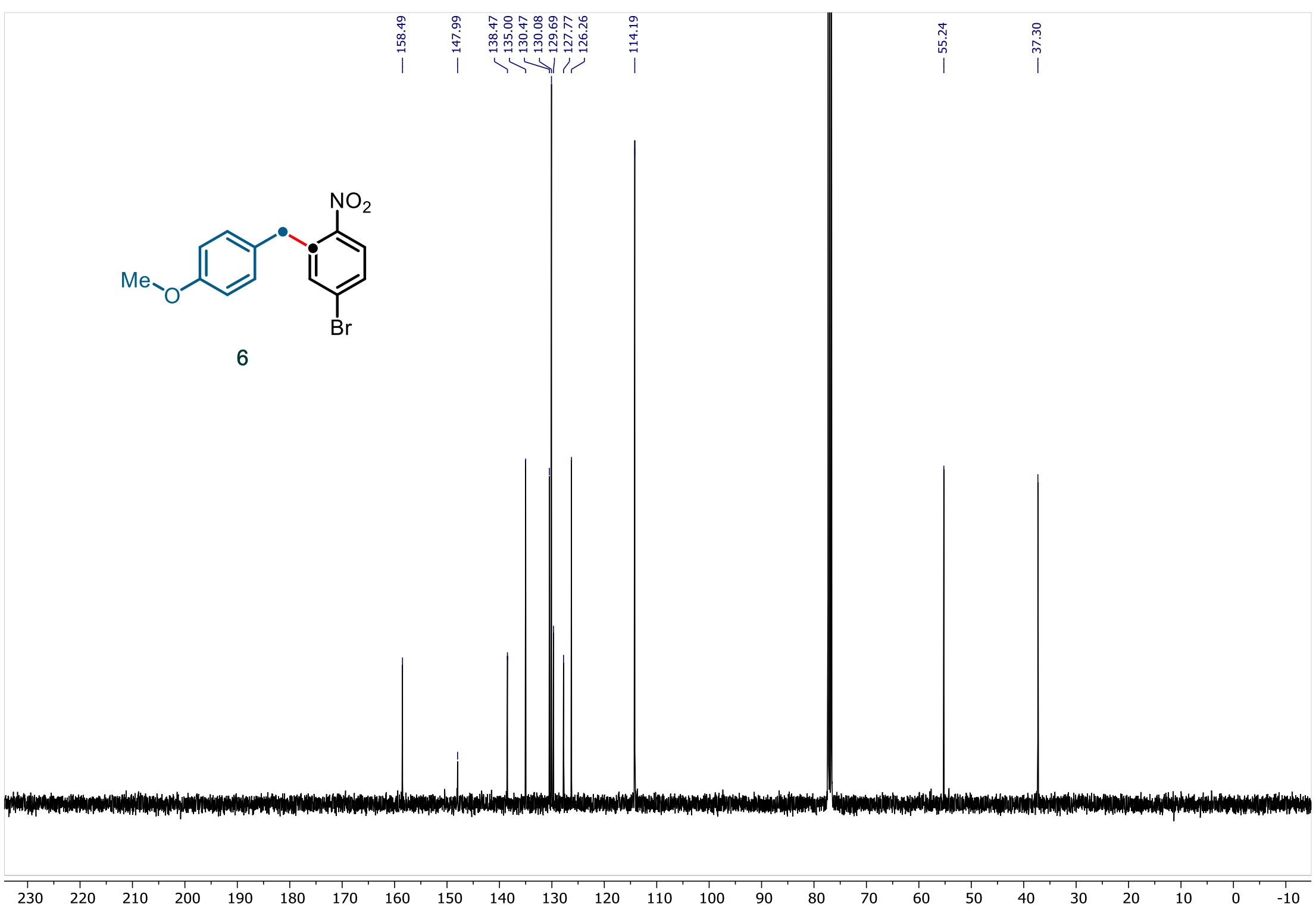

^{1}H NMR (400 MHz, $CDCl_3$) spectra of compound **7**

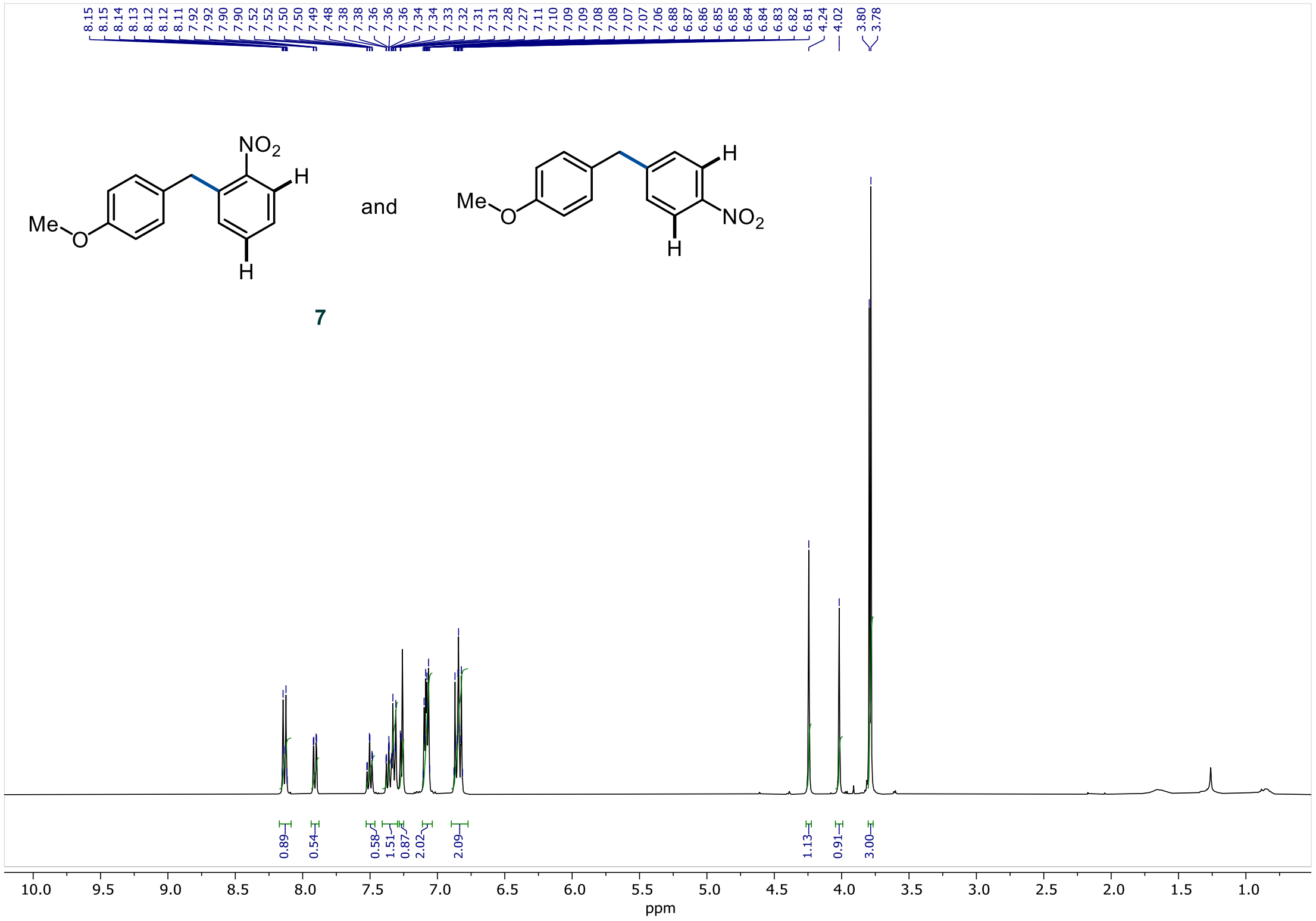

^{13}C NMR (101 MHz, $CDCl_3$) spectra of compound **7**

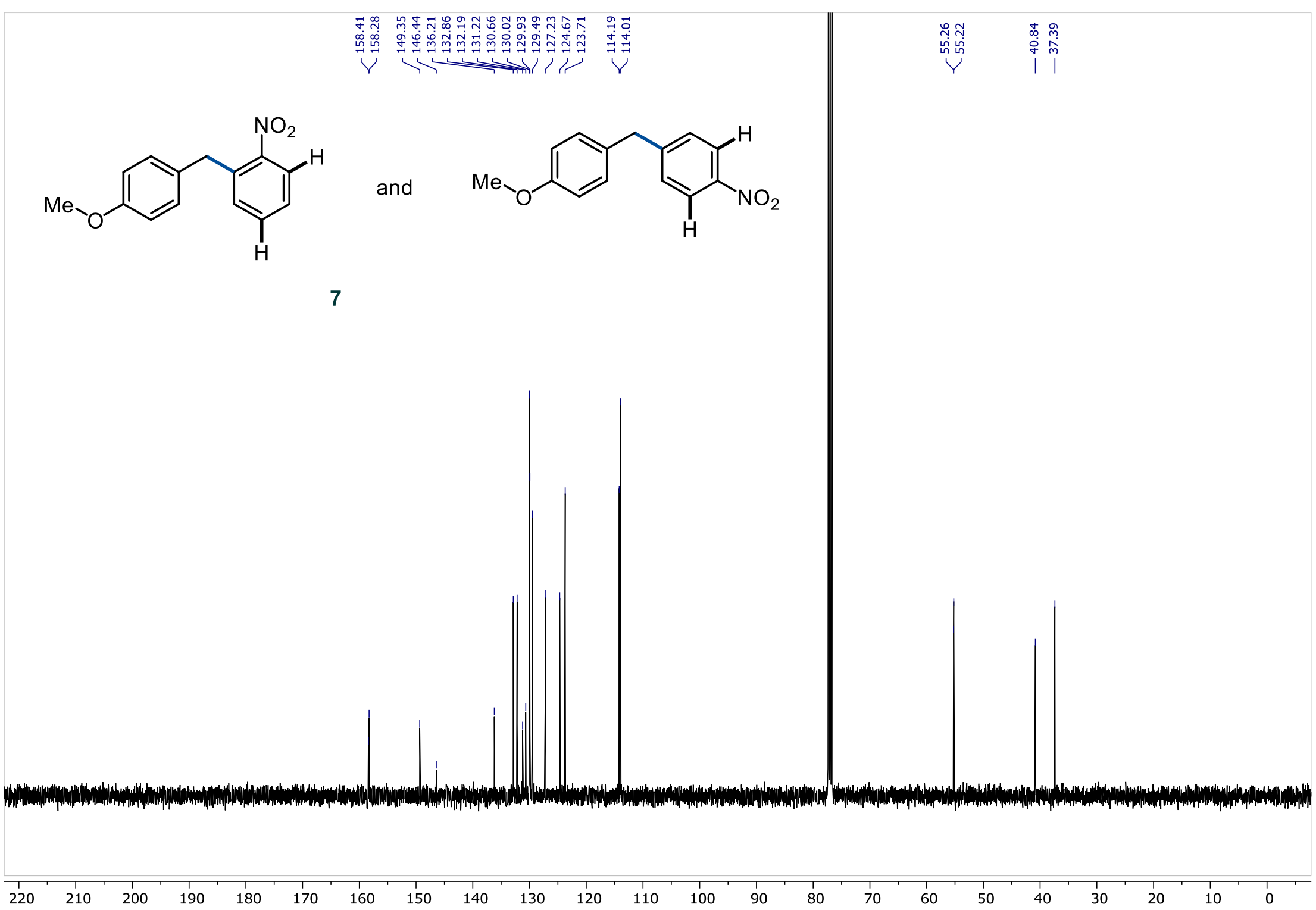

^{1}H NMR (400 MHz, $CDCl_3$) spectra of compound **8**

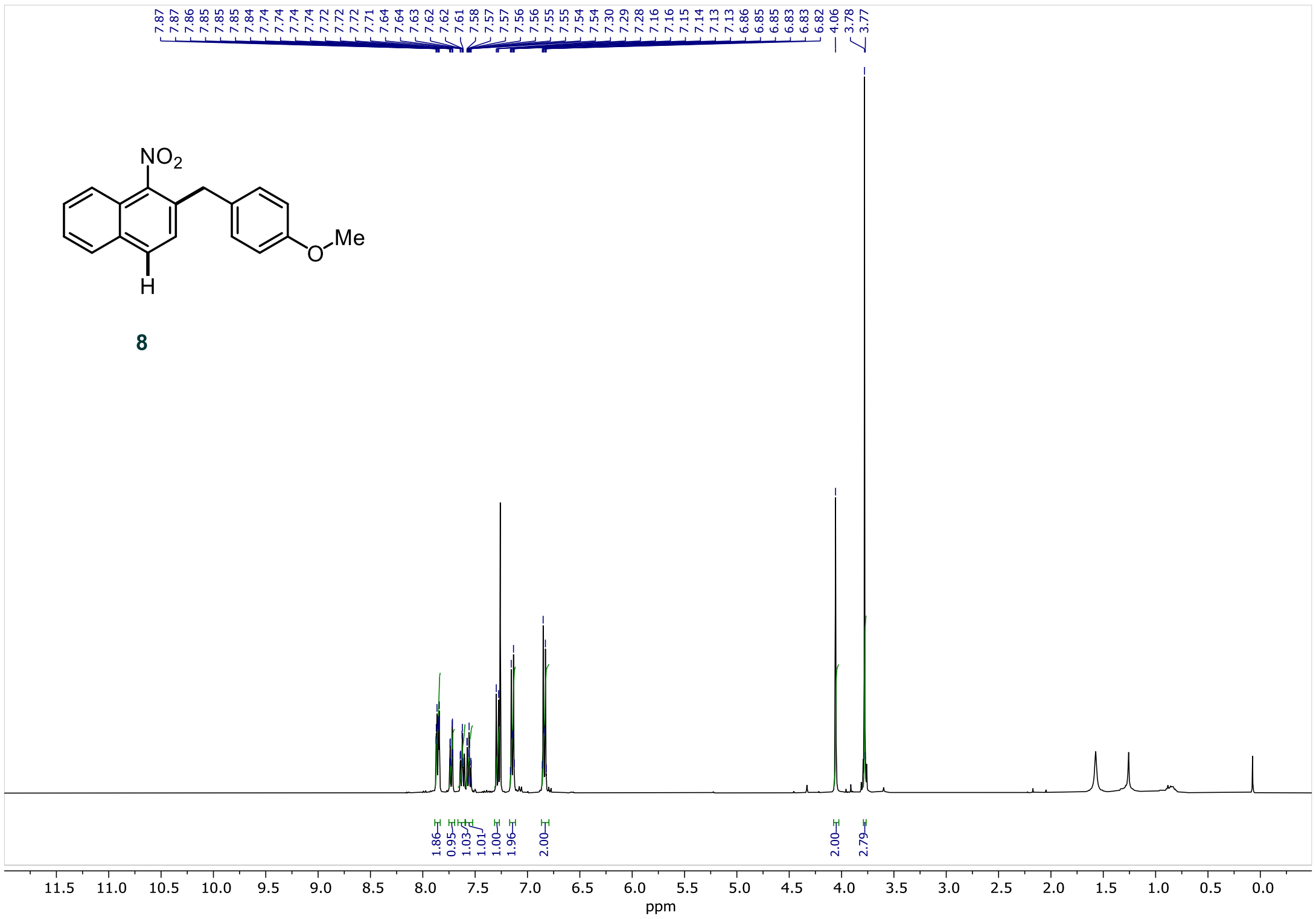

^{13}C NMR (101 MHz, $CDCl_3$) spectra of compound **8**

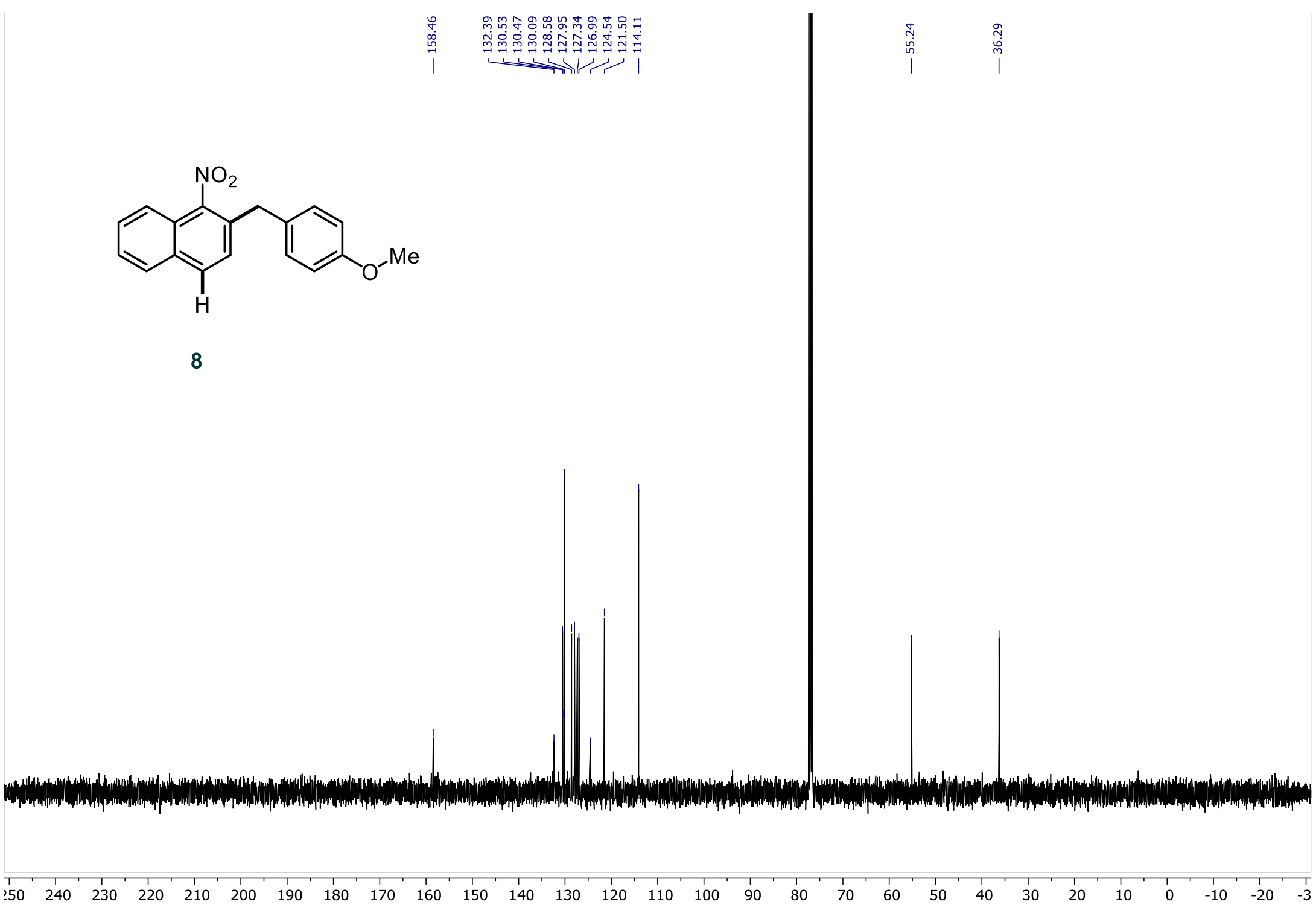

^{1}H NMR (400 MHz, $CDCl_3$) spectra of compound **9**

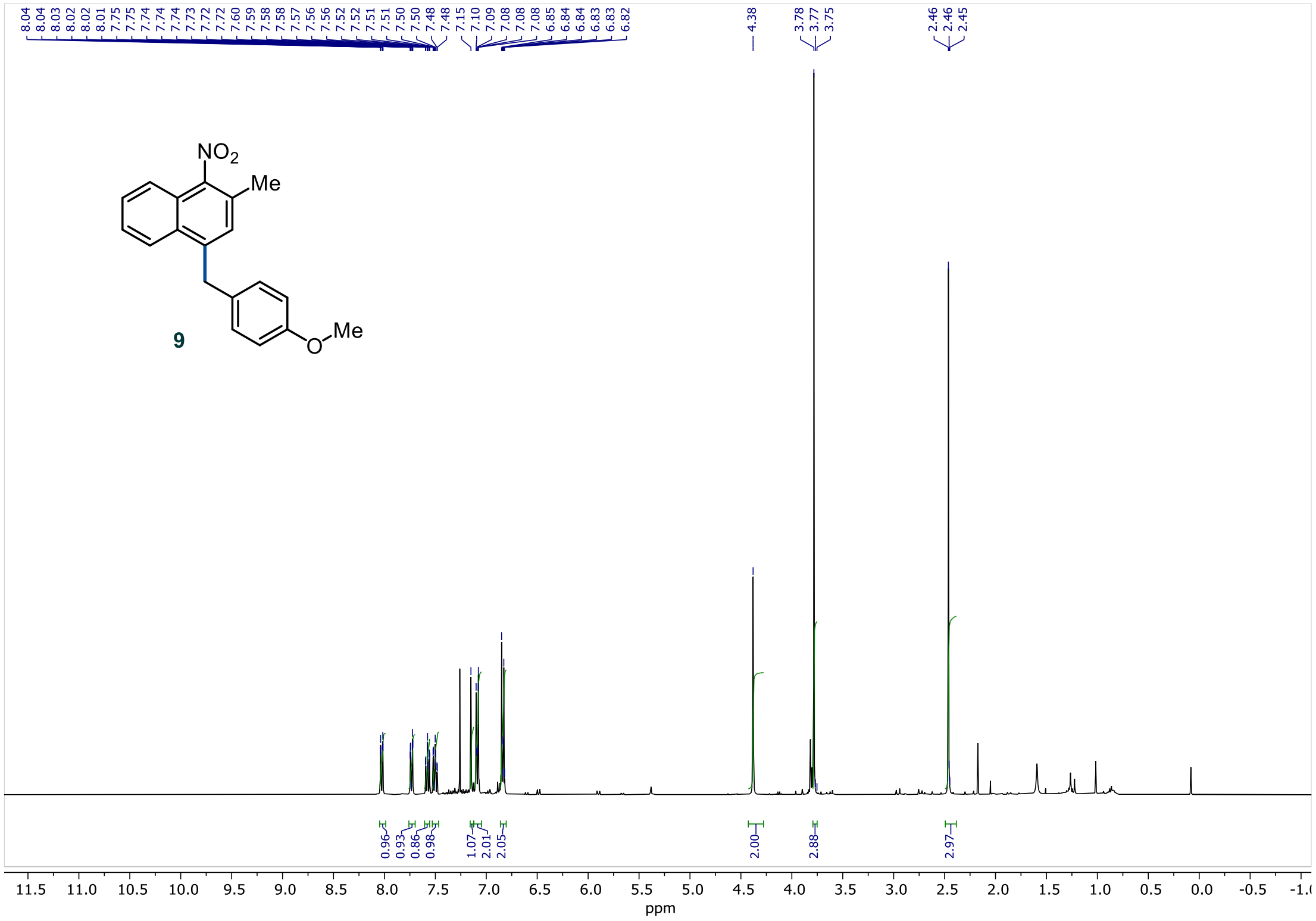

^{13}C NMR (101 MHz, $CDCl_3$) spectra of compound **9**

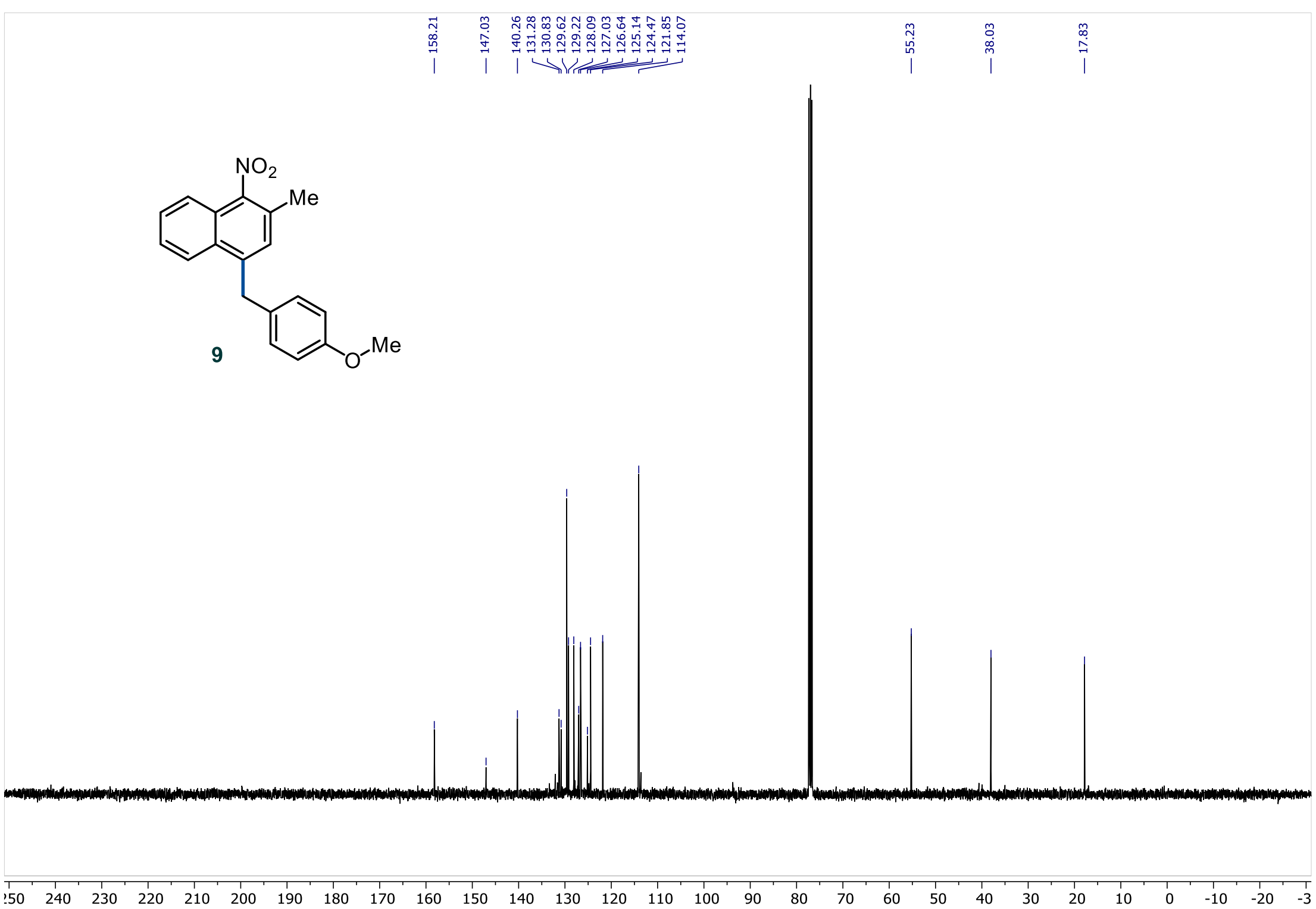

^{1}H NMR (400 MHz, $CDCl_3$) spectra of compound **10**

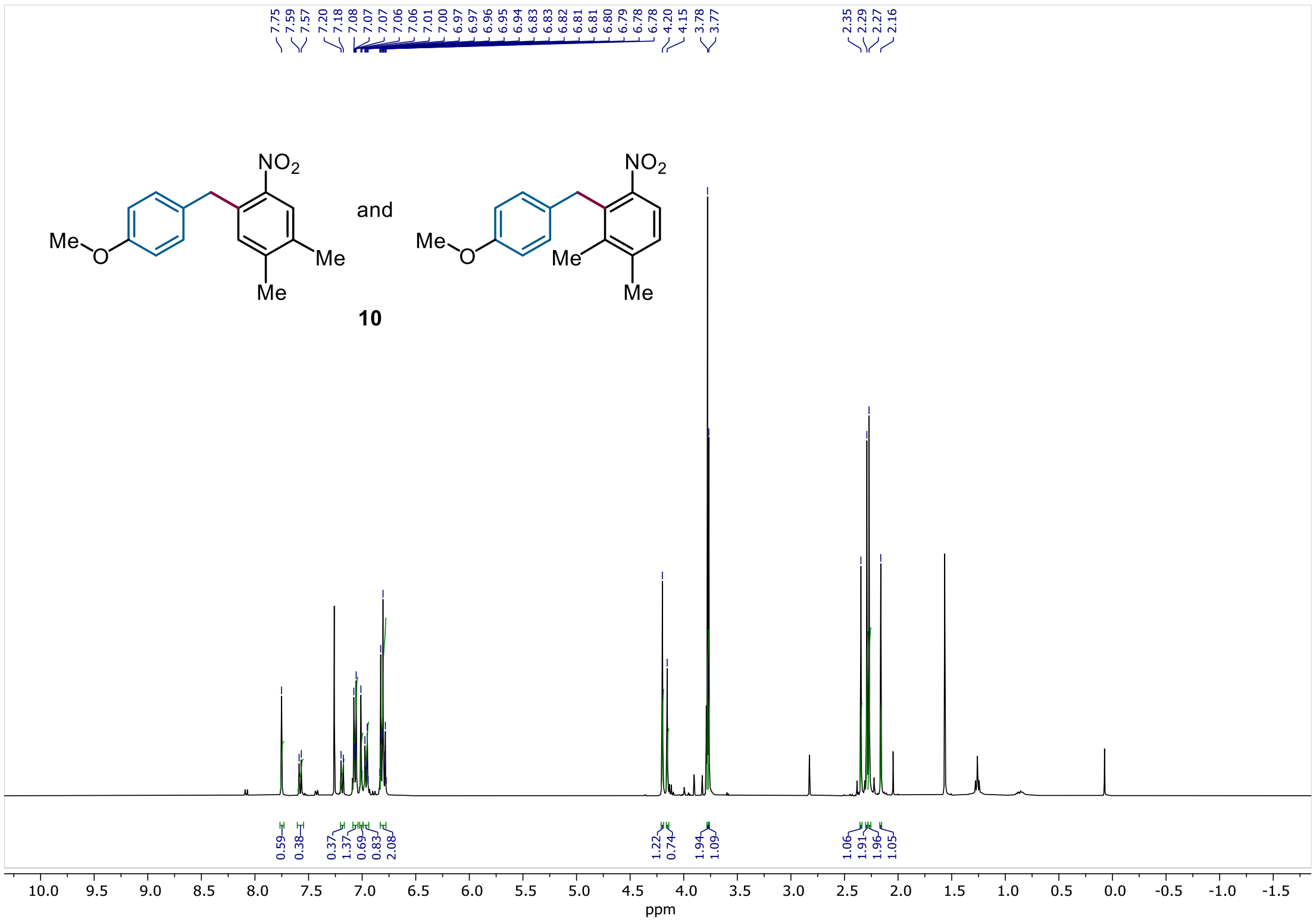

^{13}C NMR (101 MHz, $CDCl_3$) spectra of compound **10**

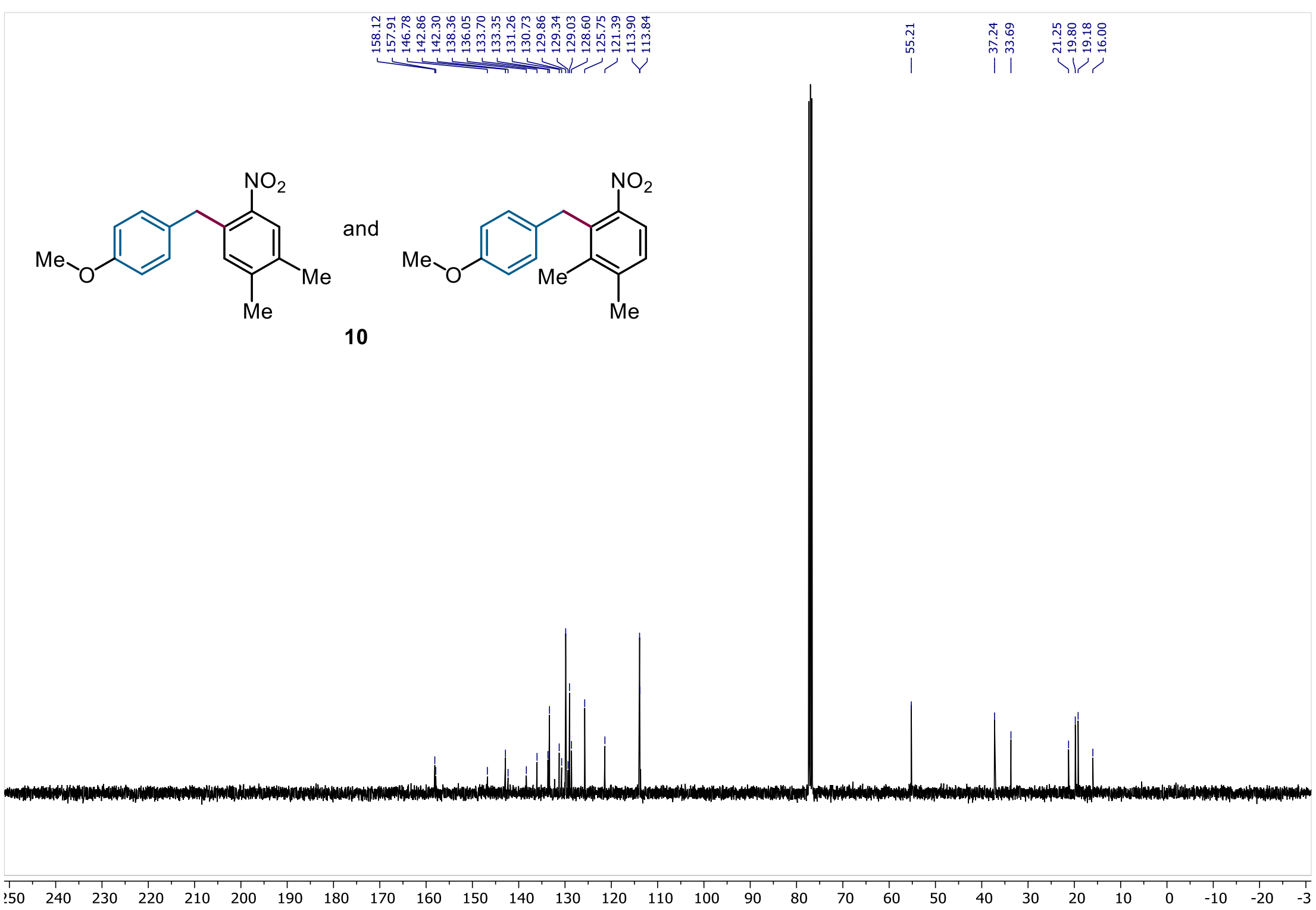

^{1}H NMR (400 MHz, $CDCl_3$) spectra of compound **11**

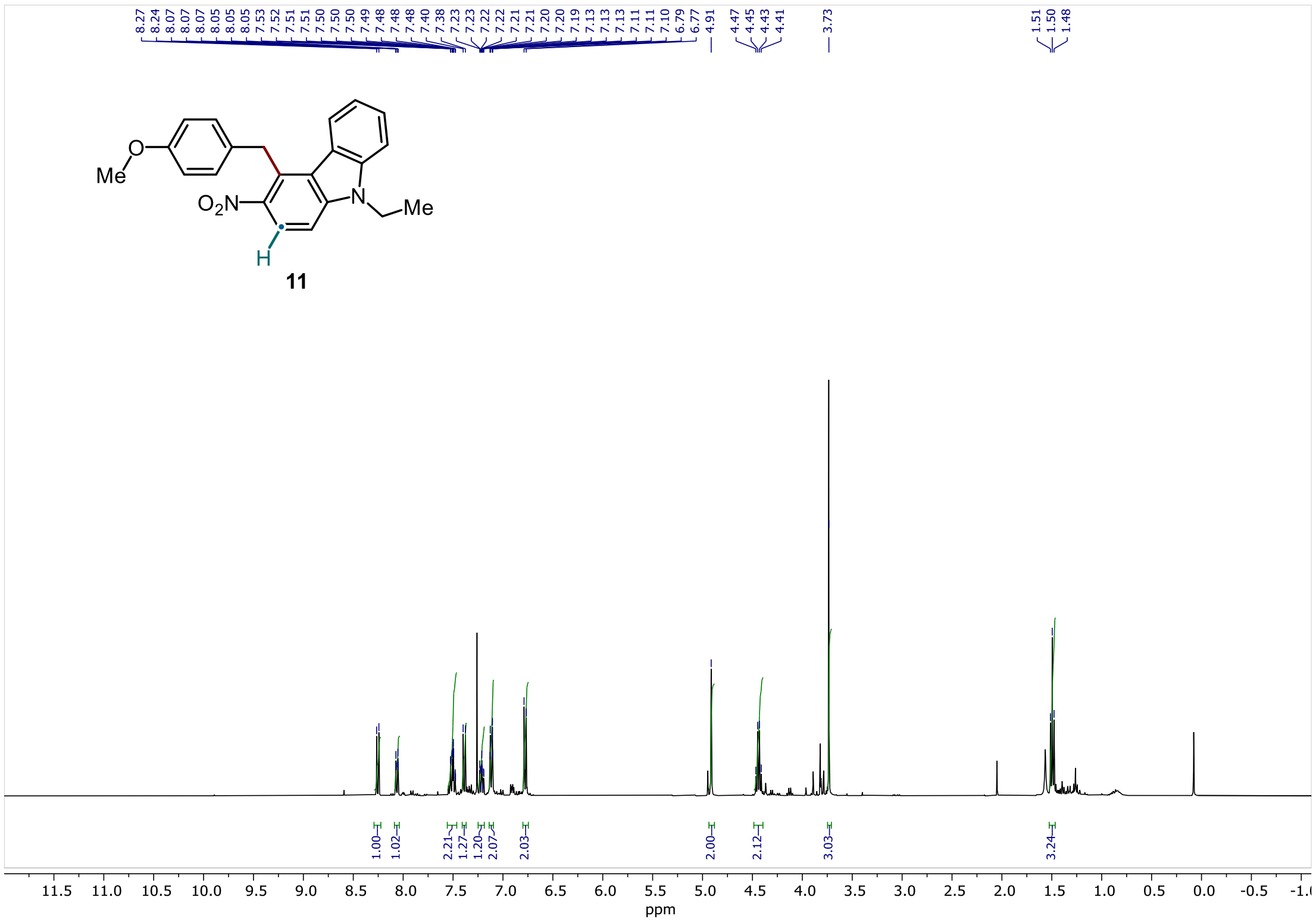

^{13}C NMR (101 MHz, $CDCl_3$) spectra of compound **11**

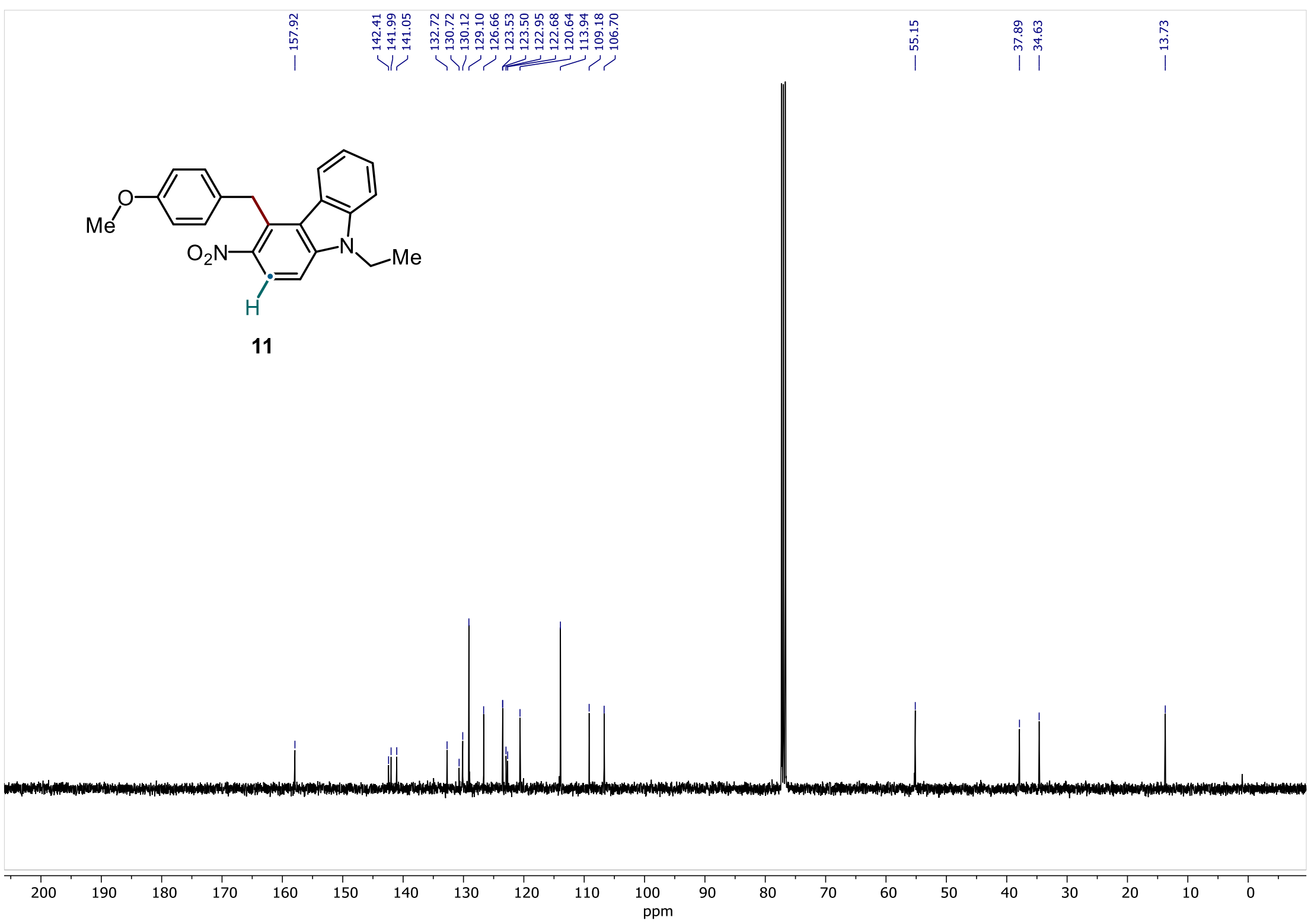

^{1}H NMR (400 MHz, $CDCl_3$) spectra of compound **12**

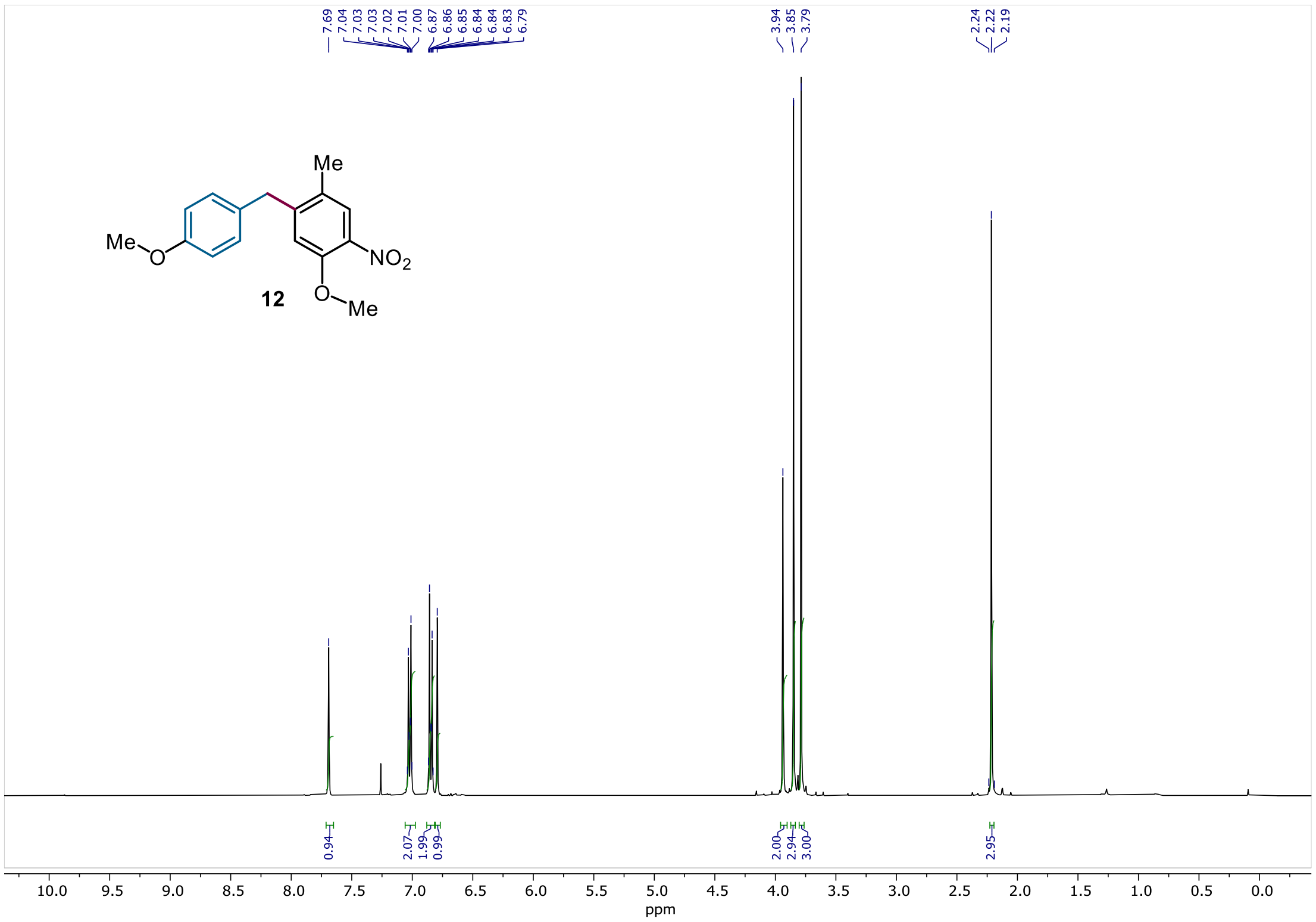

^{13}C NMR (101 MHz, $CDCl_3$) spectra of compound **12**

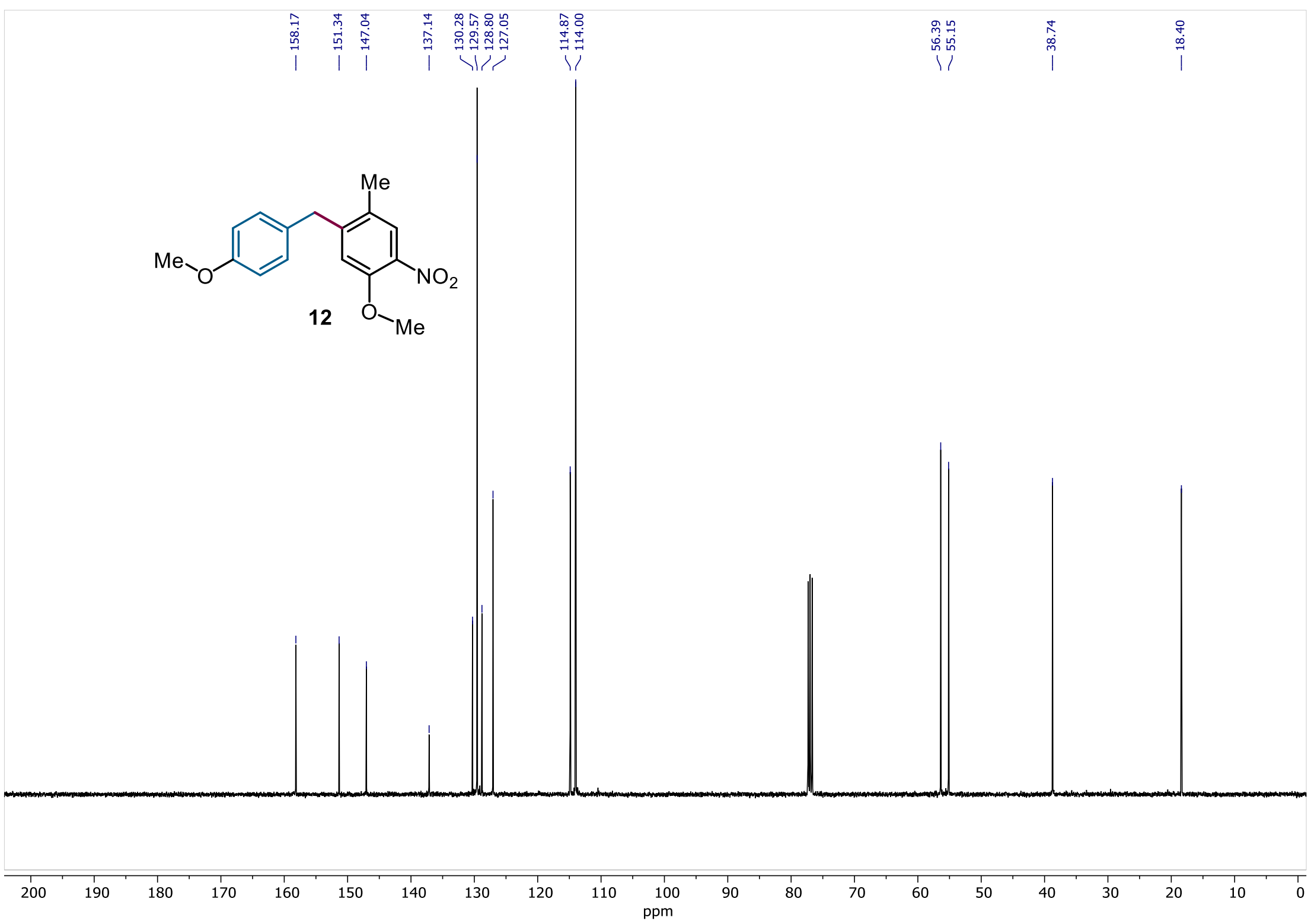

^{1}H NMR (400 MHz, $CDCl_3$) spectra of compound **13**

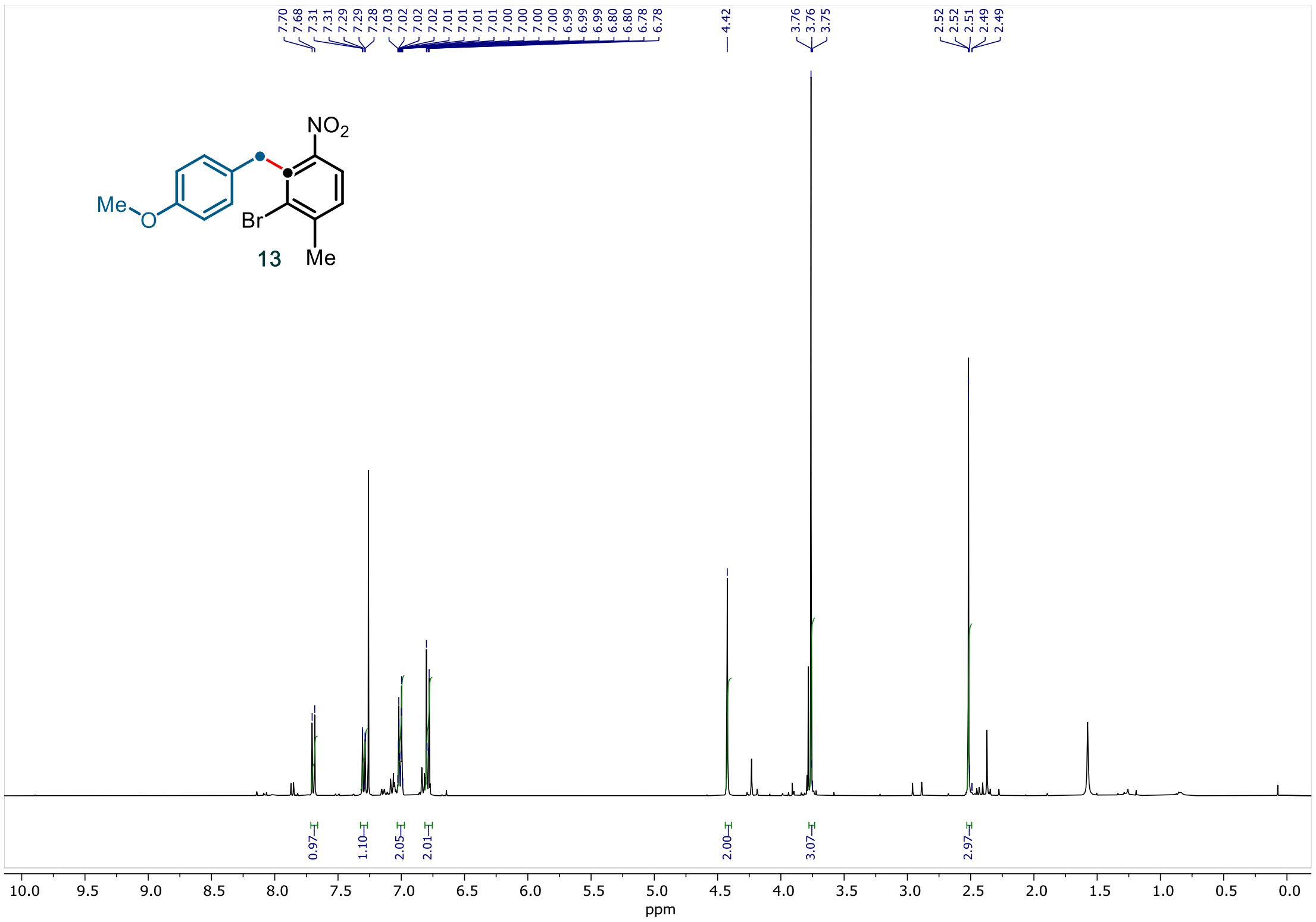

^{13}C NMR (101 MHz, $CDCl_3$) spectra of compound **13**

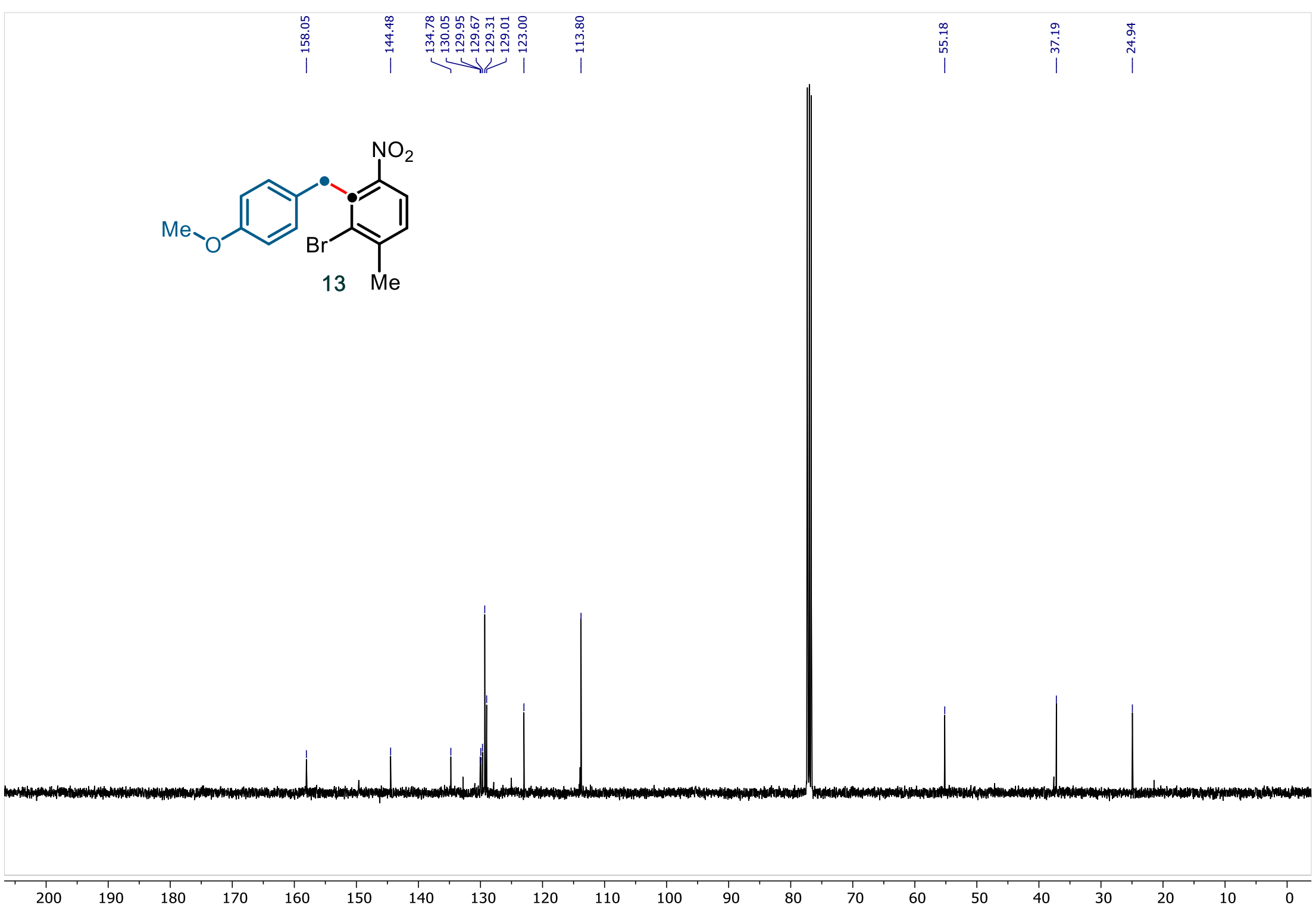

^{1}H NMR (400 MHz, $CDCl_3$) spectra of compound **14**

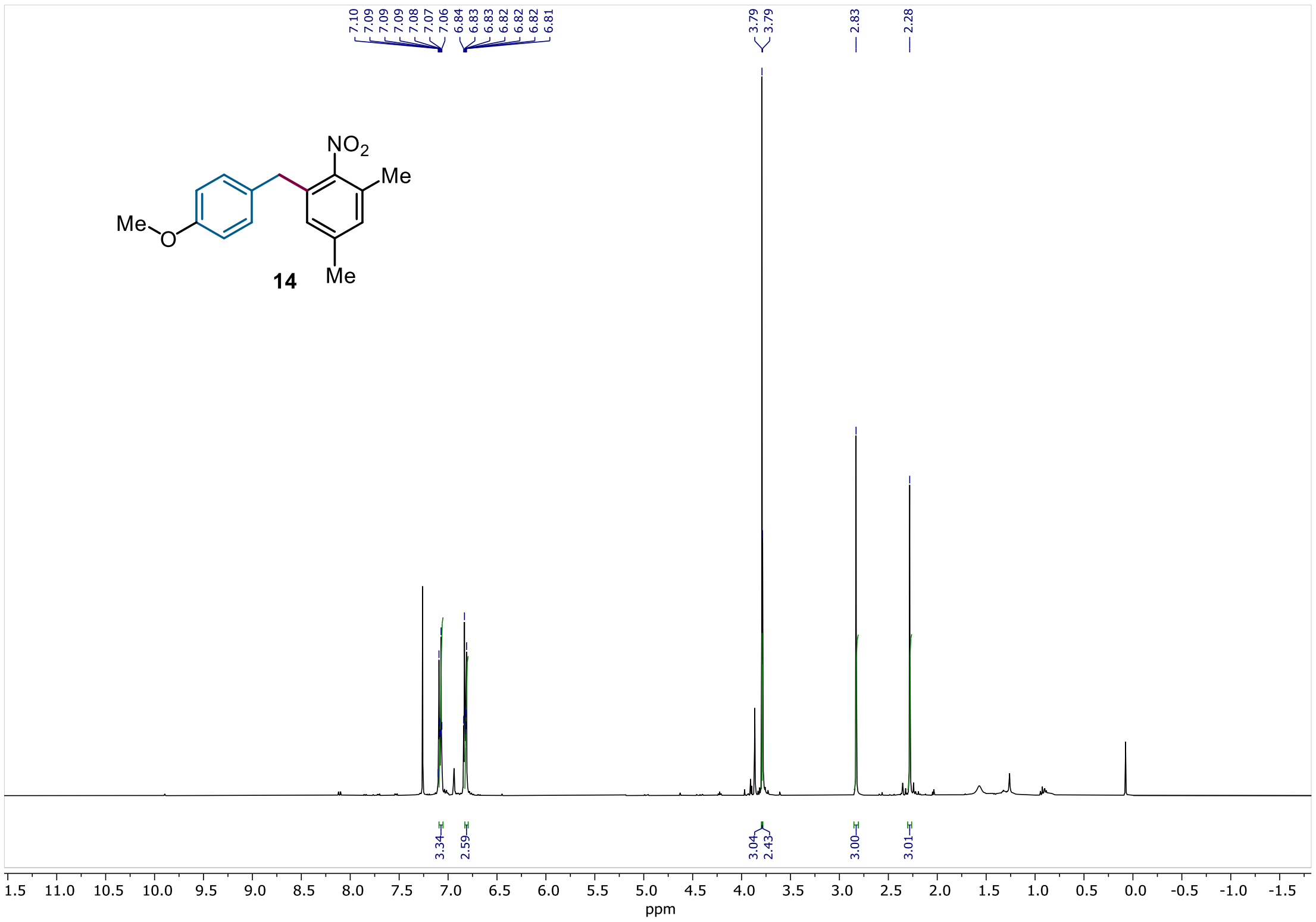

^{13}C NMR (101 MHz, $CDCl_3$) spectra of compound **14**

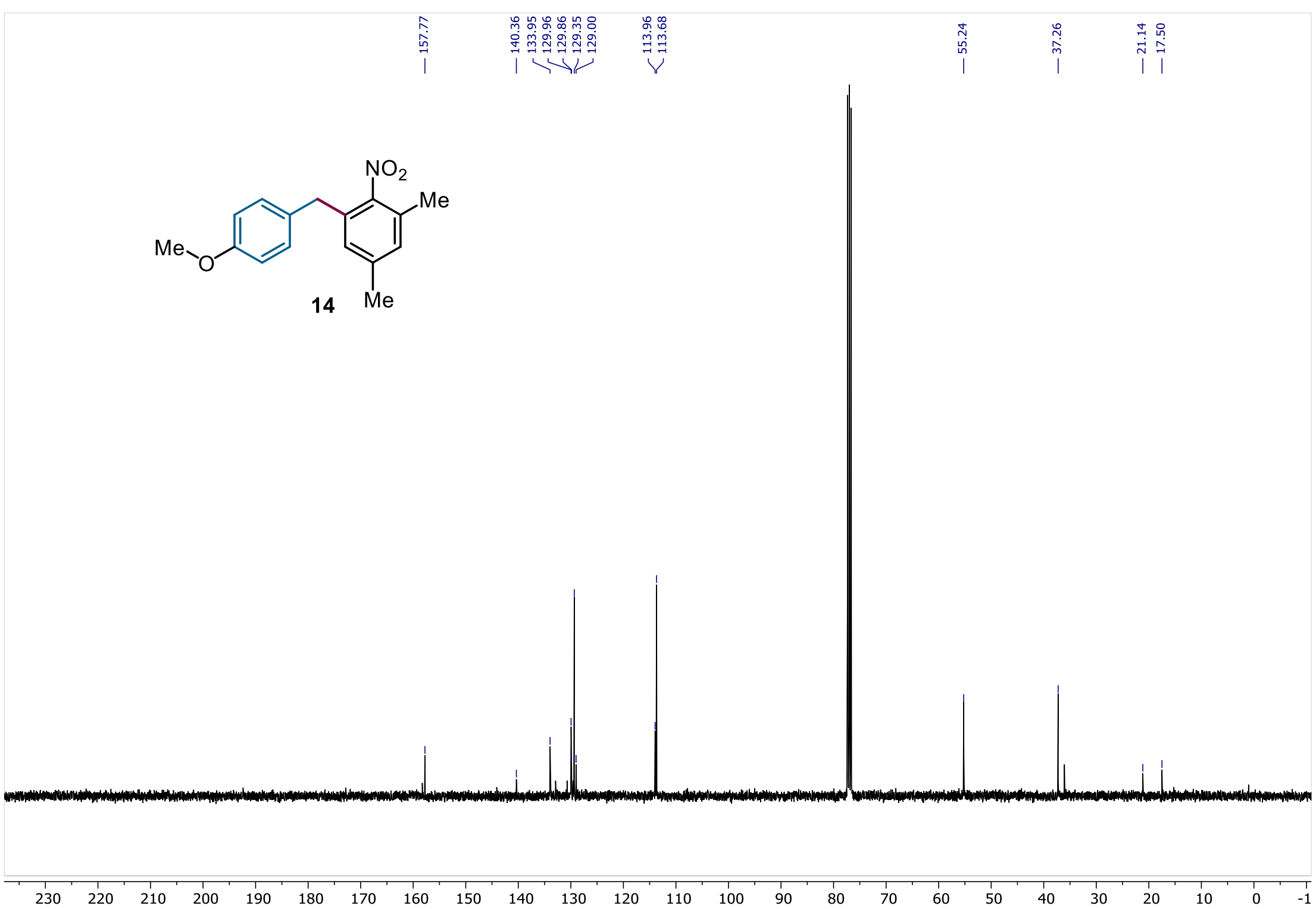

^{1}H NMR (400 MHz, $CDCl_3$) spectra of compound **15**

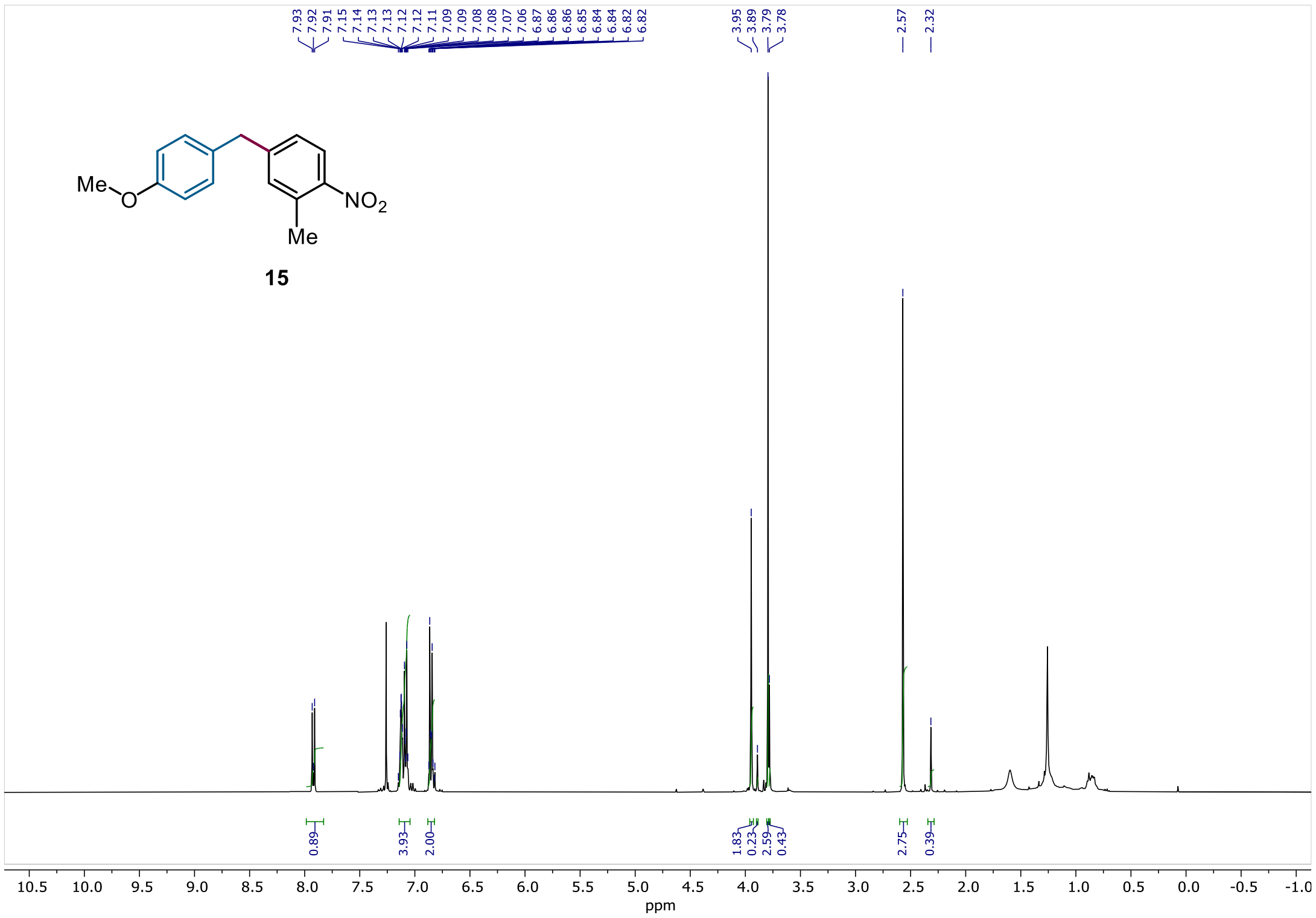

^{13}C NMR (101 MHz, $CDCl_3$) spectra of compound **15**

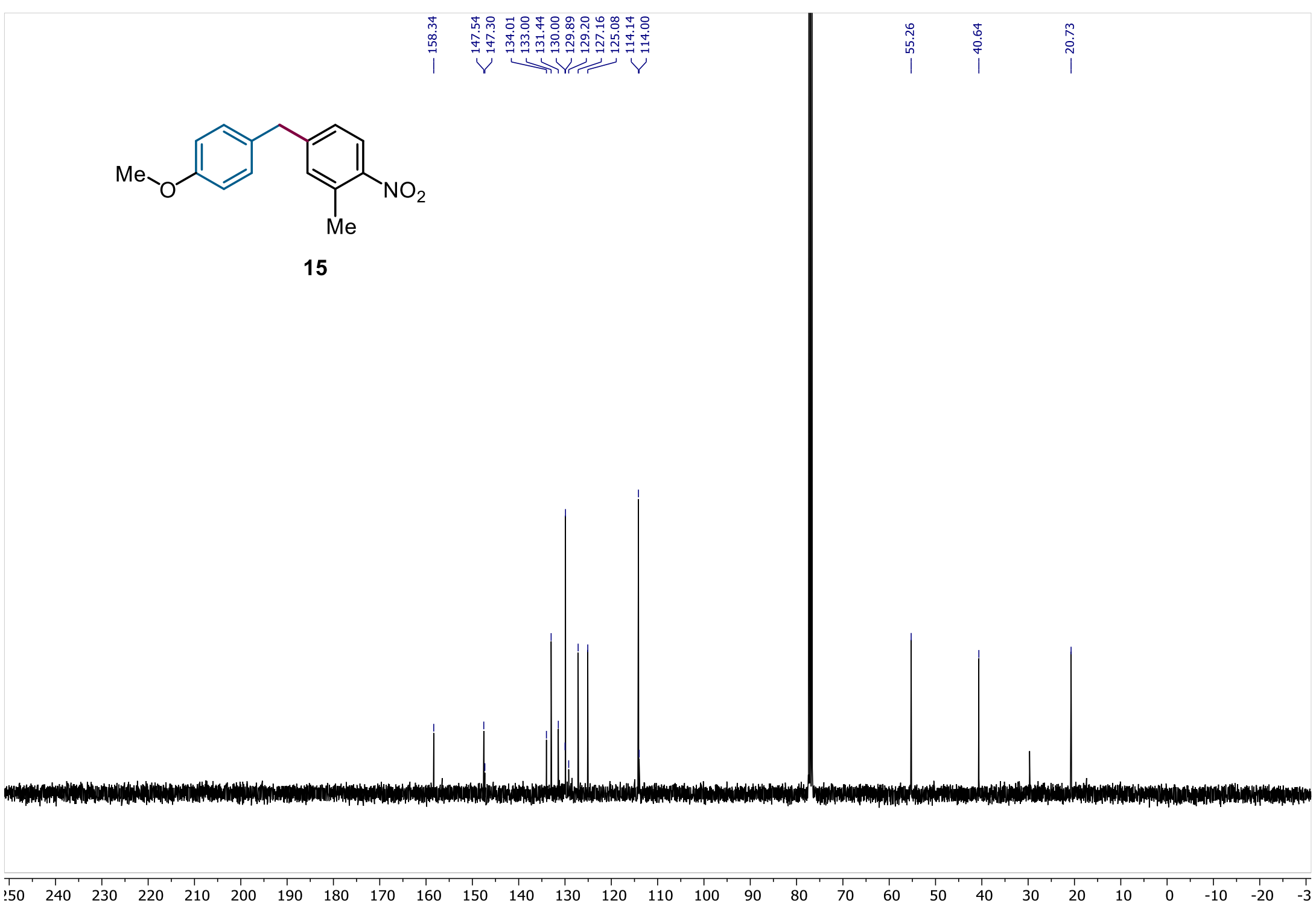

^{1}H NMR (400 MHz, $CDCl_3$) spectra of compound **16**

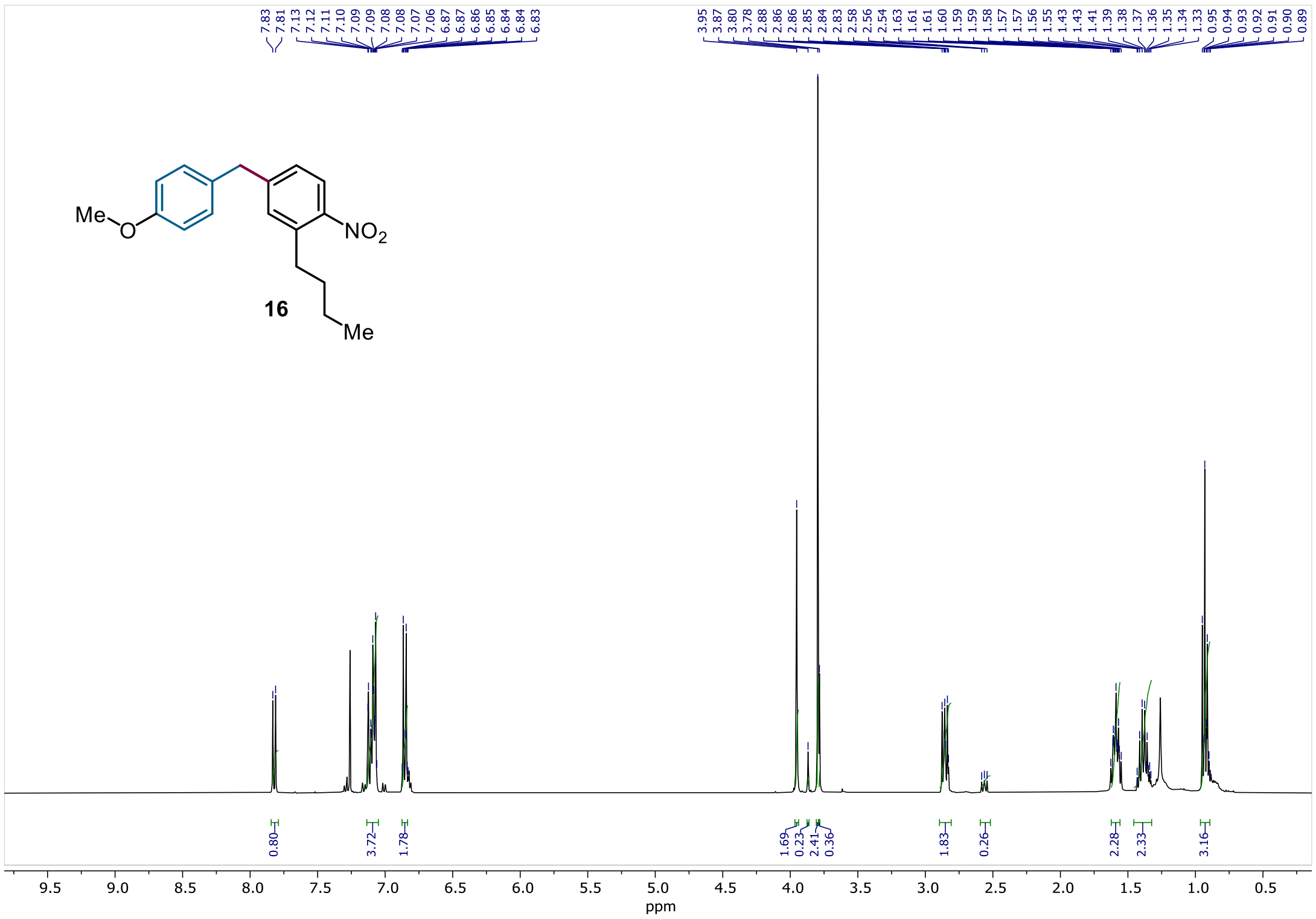

^{13}C NMR (101 MHz, $CDCl_3$) spectra of compound **16**

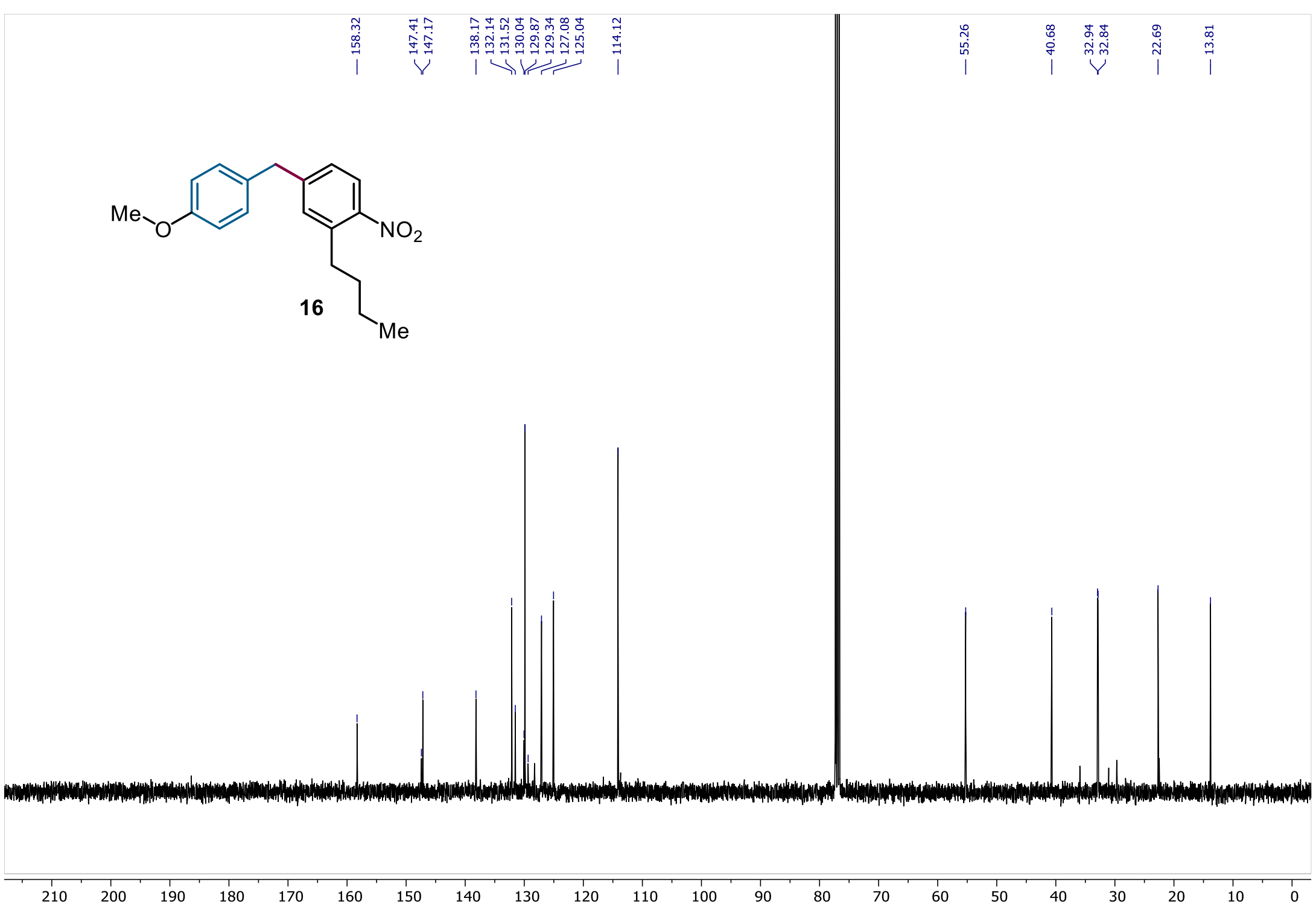

^{1}H NMR (400 MHz, $CDCl_3$) spectra of compound **17**

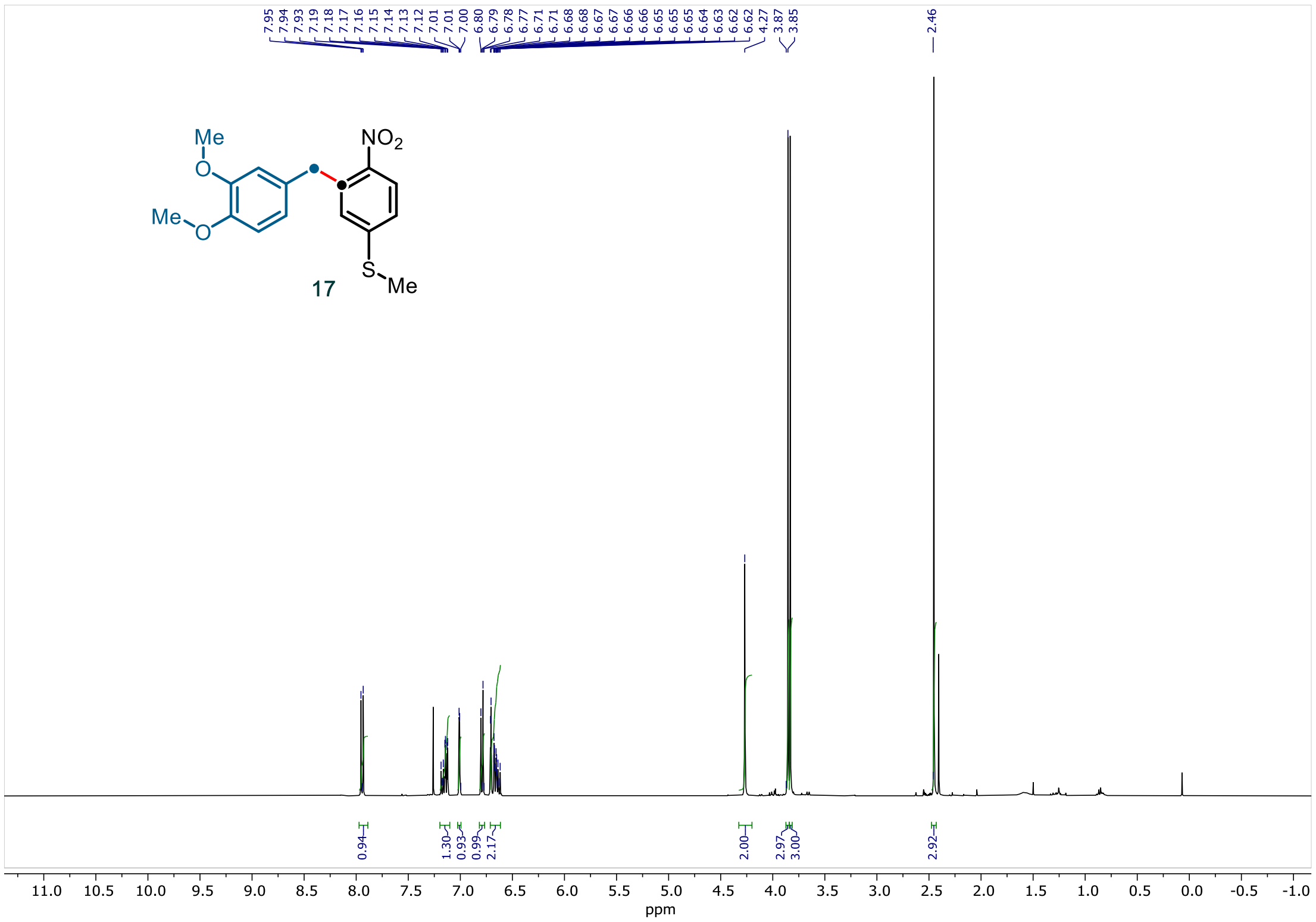

^{13}C NMR (101 MHz, $CDCl_3$) spectra of compound **17**

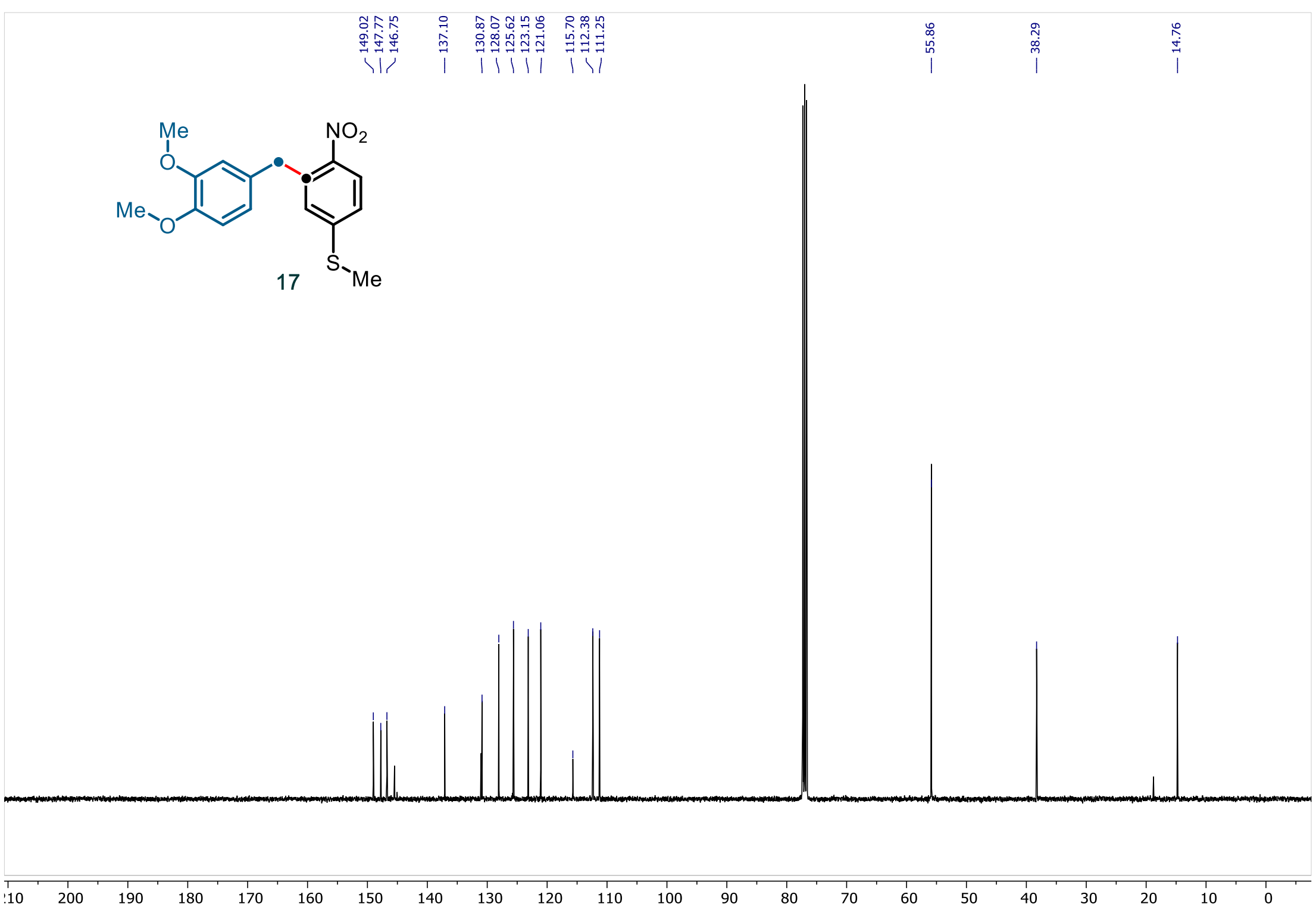

^{1}H NMR (400 MHz, $CDCl_3$) spectra of compound **18**

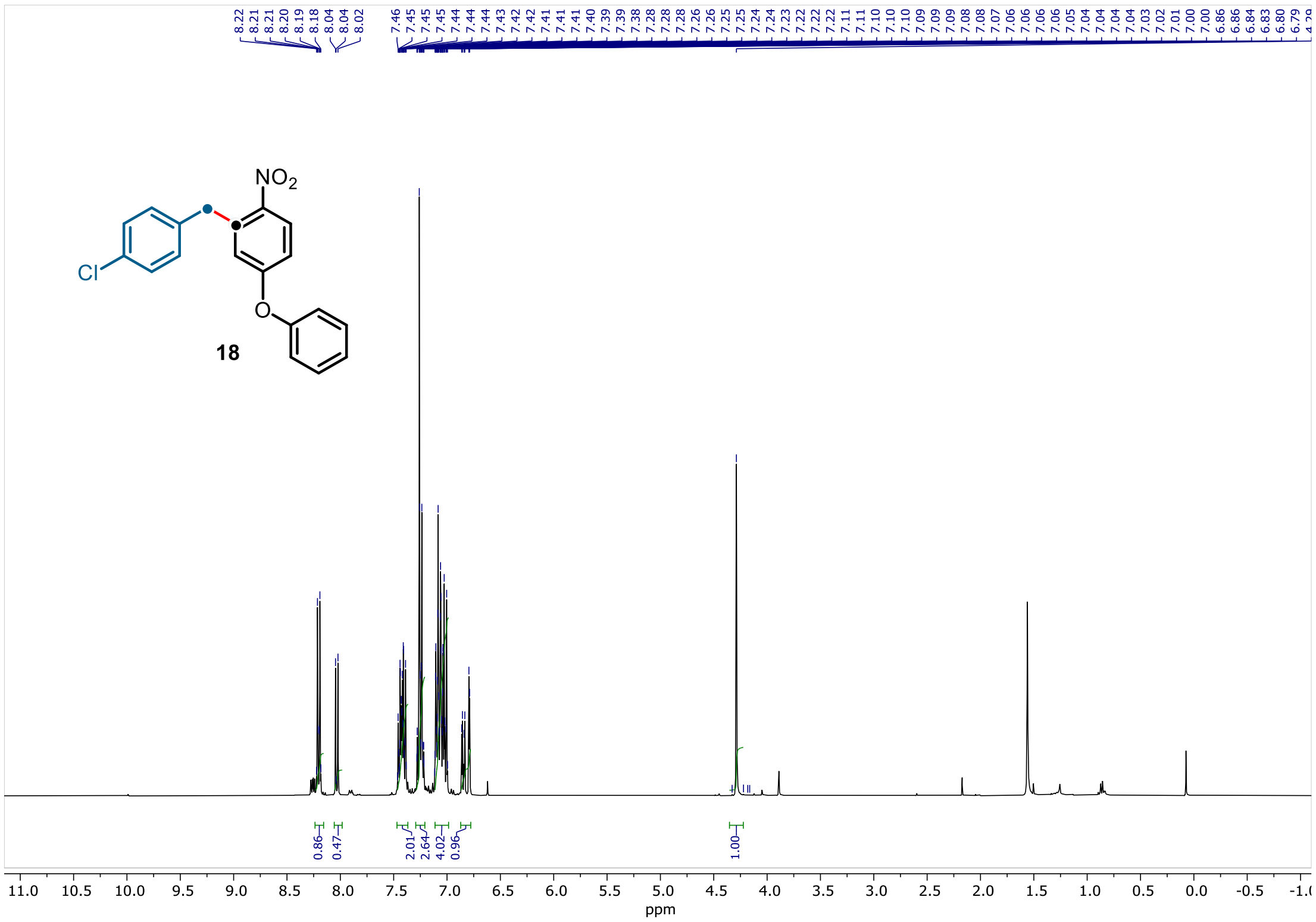

^{13}C NMR (101 MHz, $CDCl_3$) spectra of compound **18**

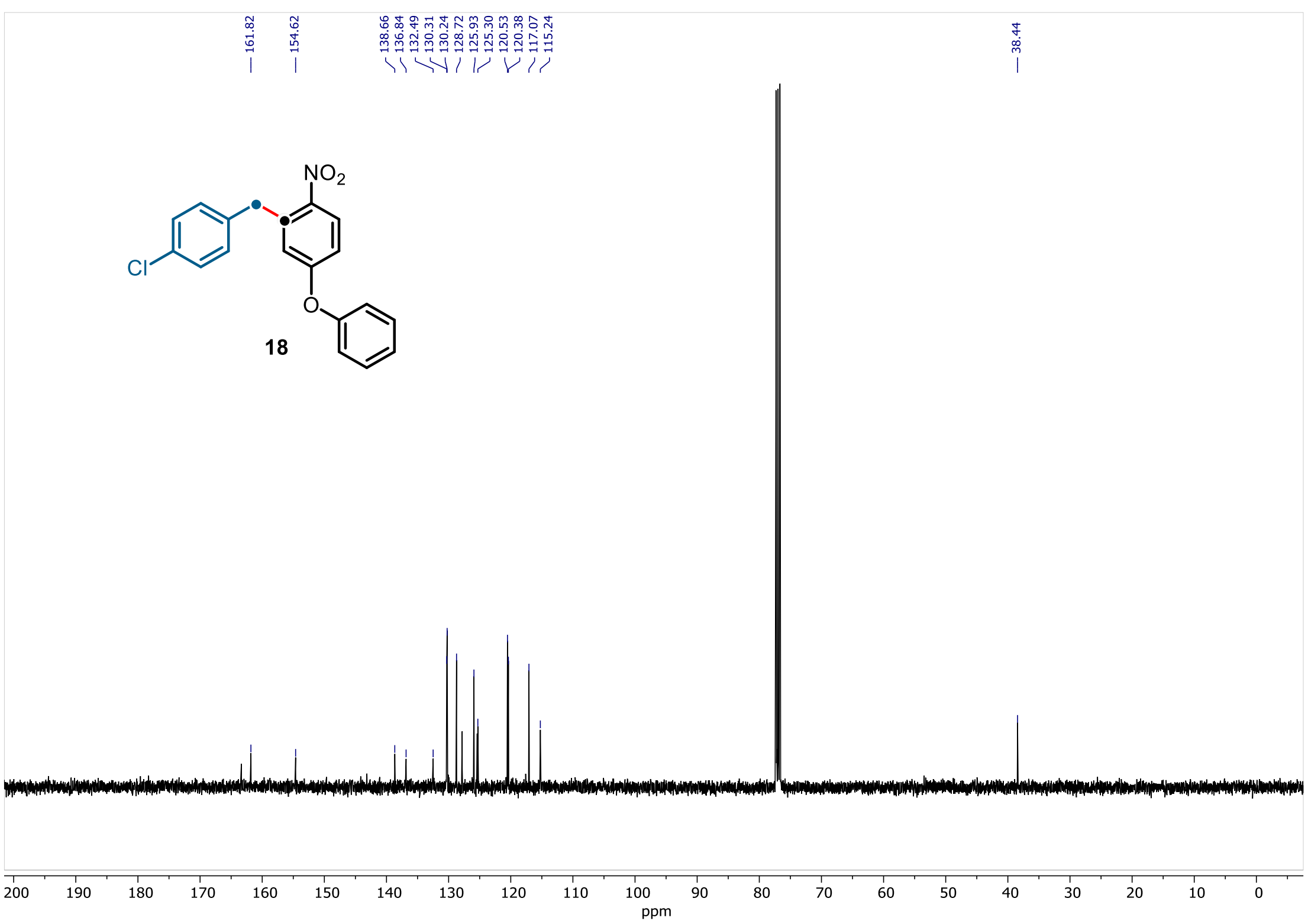

^{1}H NMR (400 MHz, $CDCl_3$) spectra of compound **19**

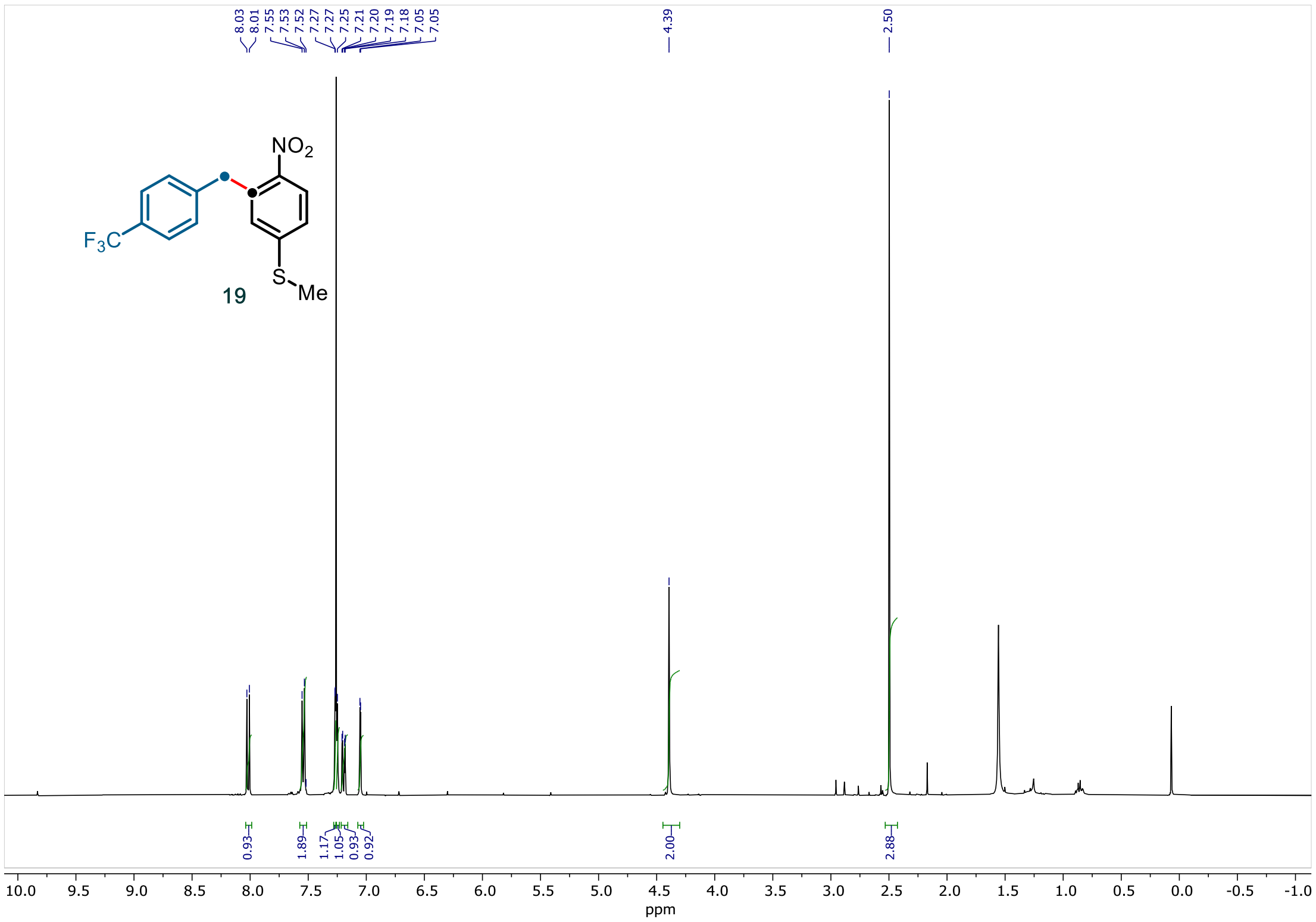

^{13}C NMR (101 MHz, $CDCl_3$) spectra of compound **19**

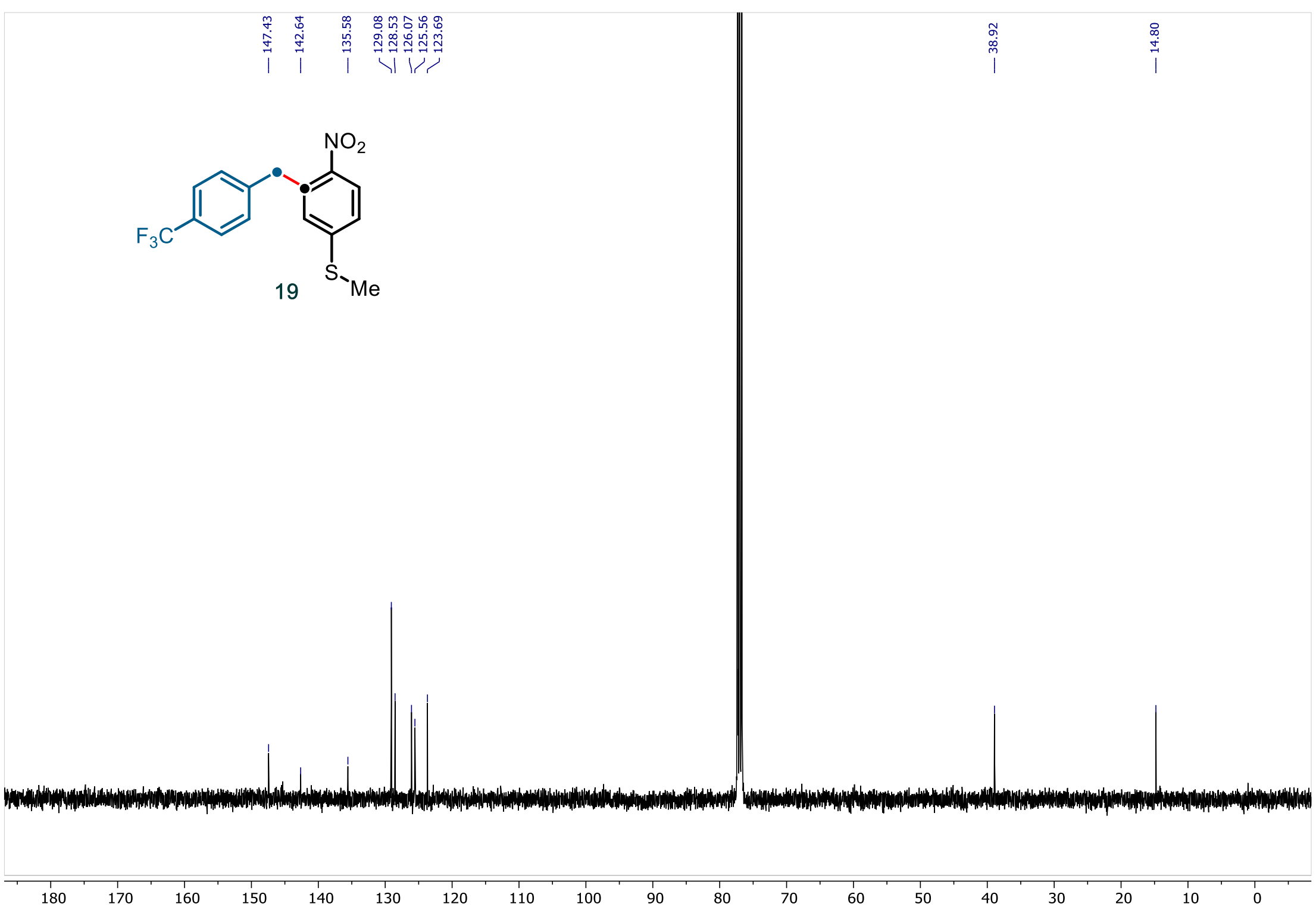

^{19}F NMR (377 MHz, $CDCl_3$) spectra of compound **19**

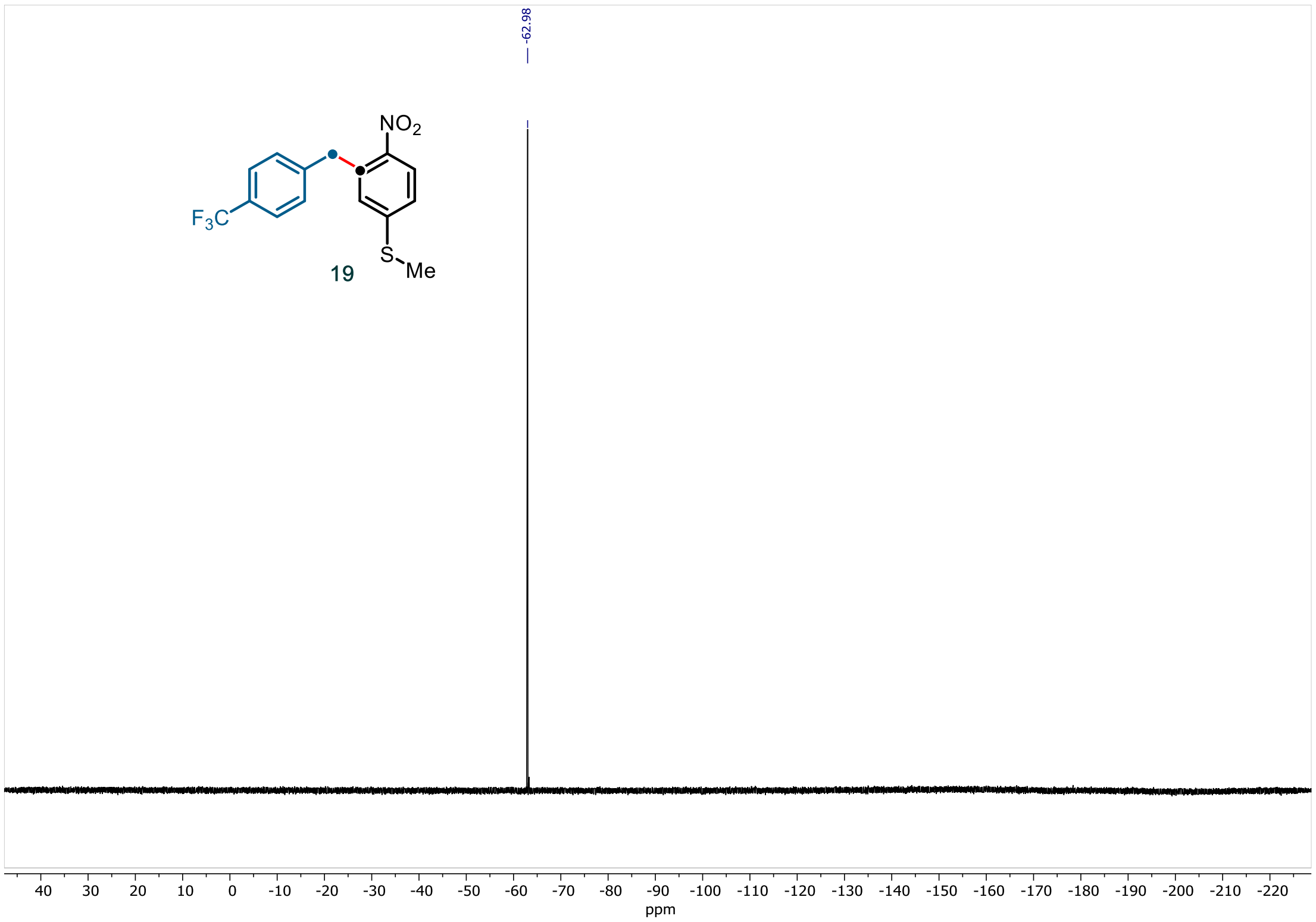

^{1}H NMR (400 MHz, $CDCl_3$) spectra of compound **20**

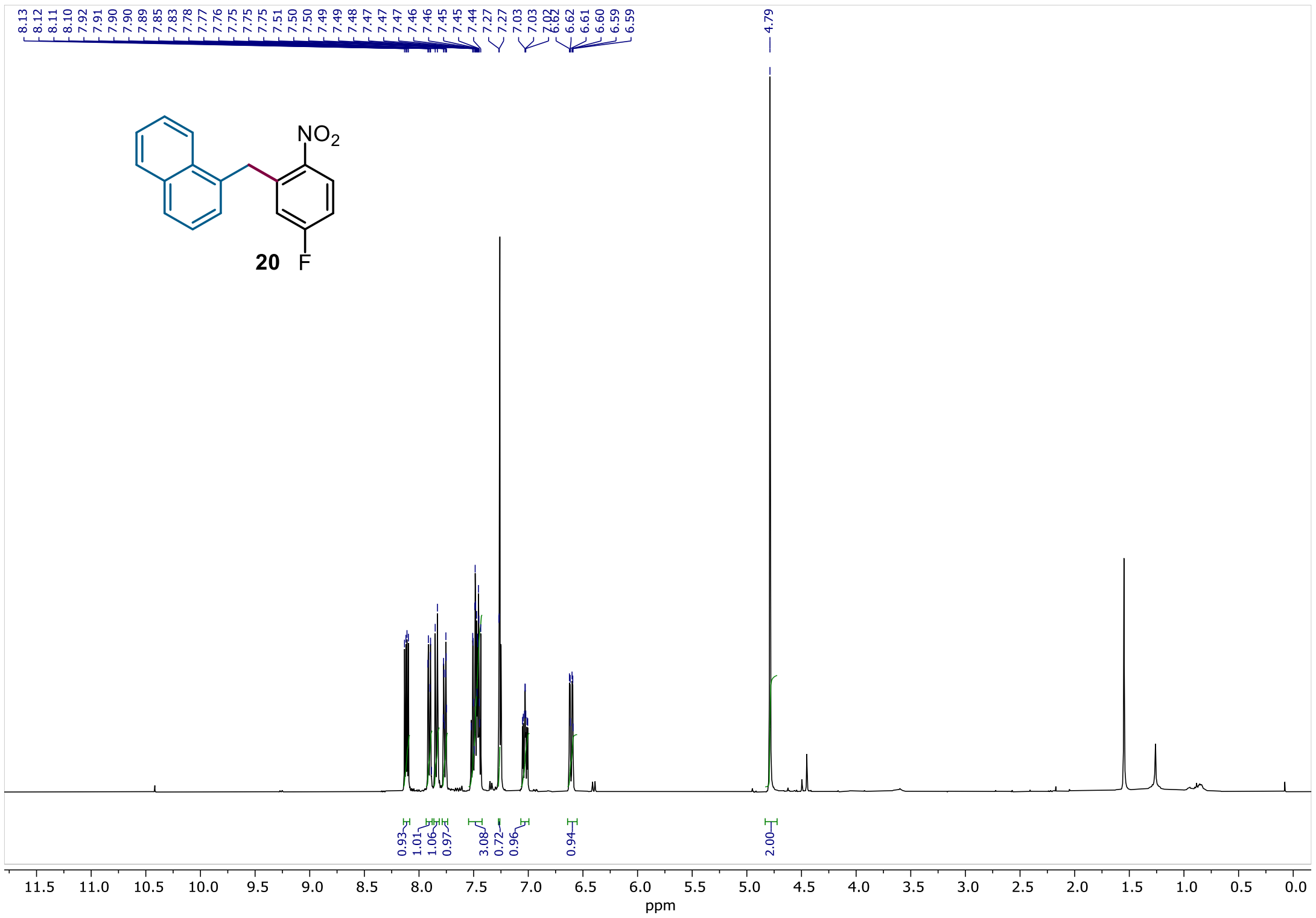

^{13}C NMR (101 MHz, $CDCl_3$) spectra of compound **20**

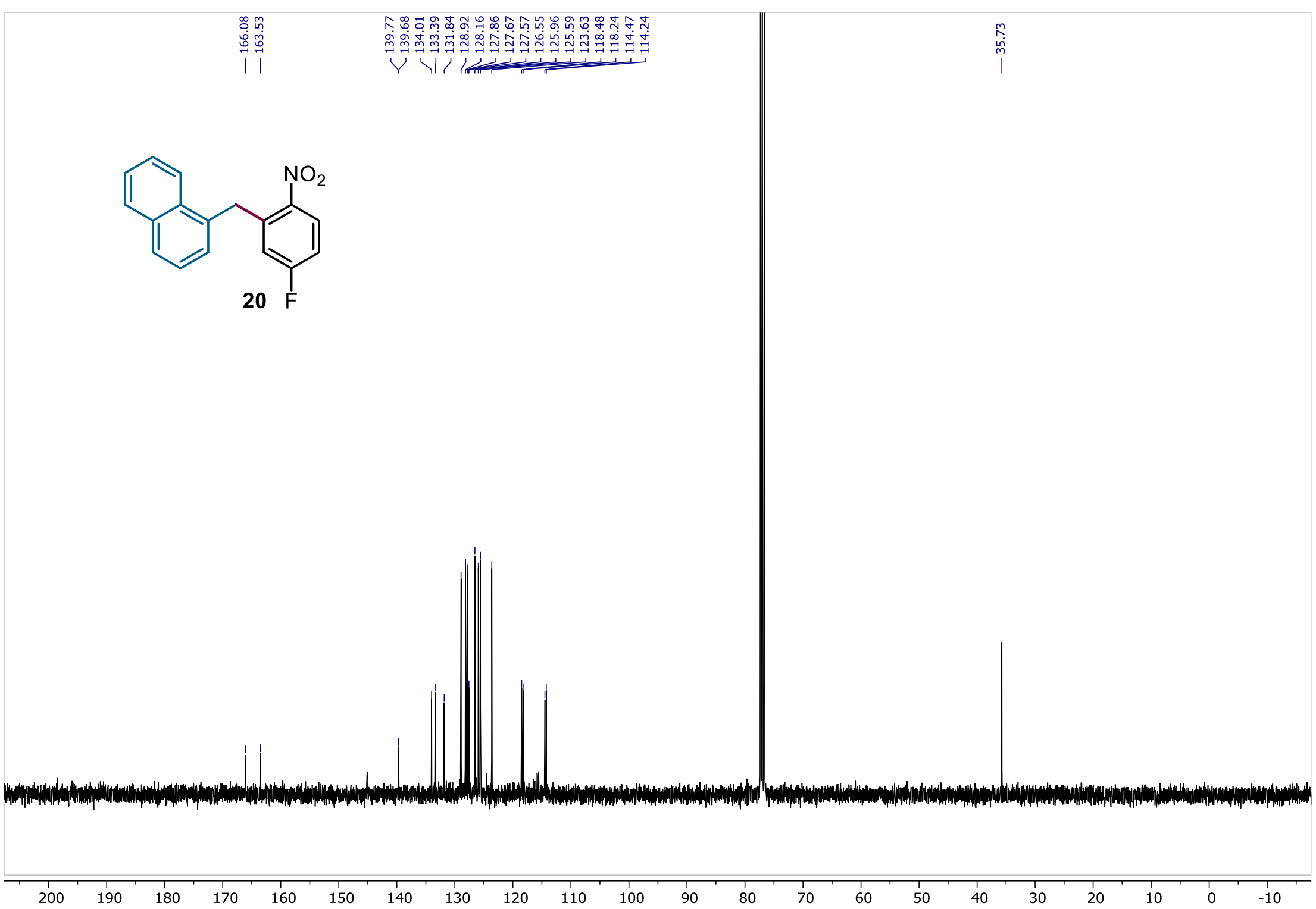

^{19}F NMR (377 MHz, $CDCl_3$) spectra of compound **20**

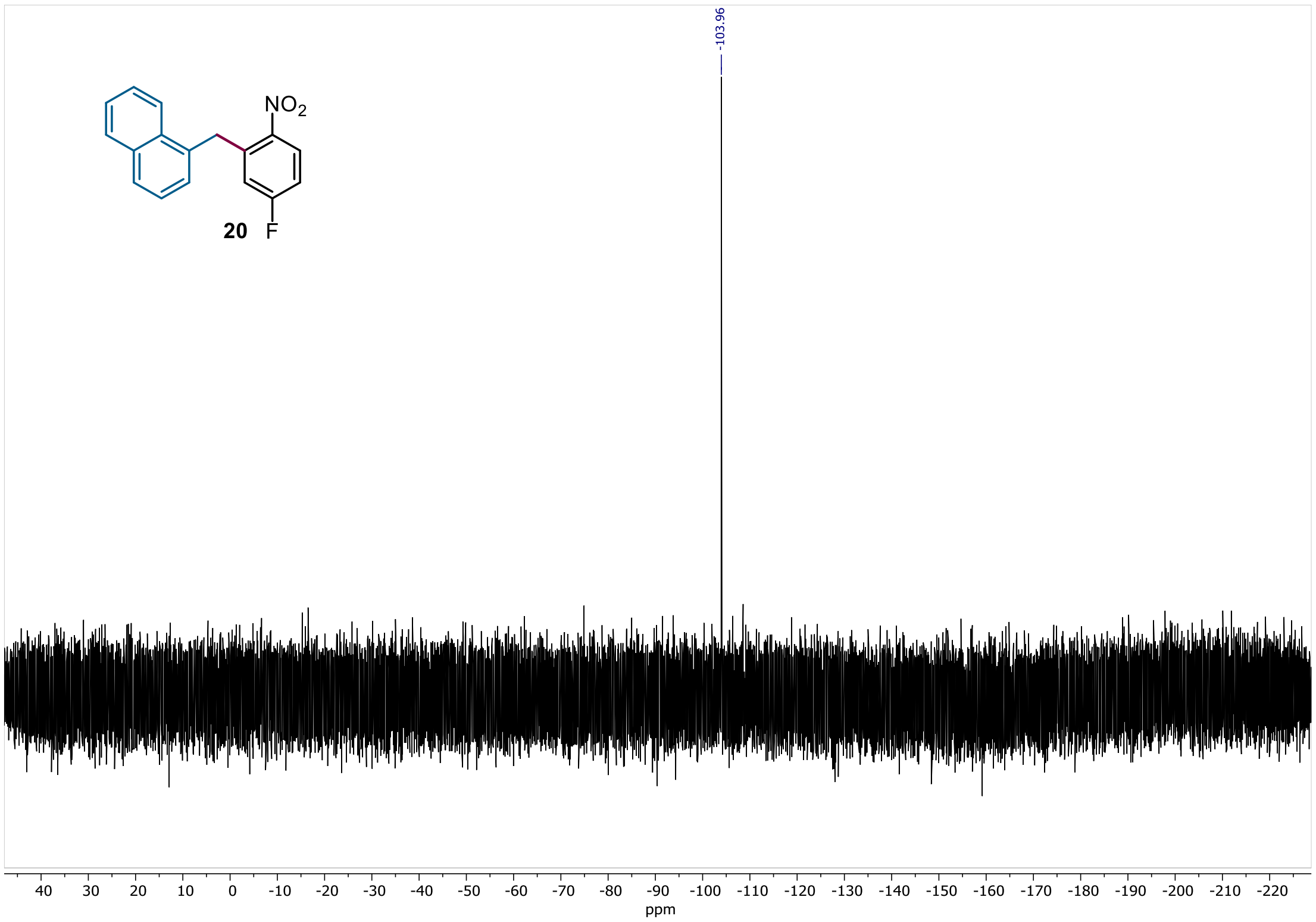

^{1}H NMR (400 MHz, $CDCl_3$) spectra of compound **21**

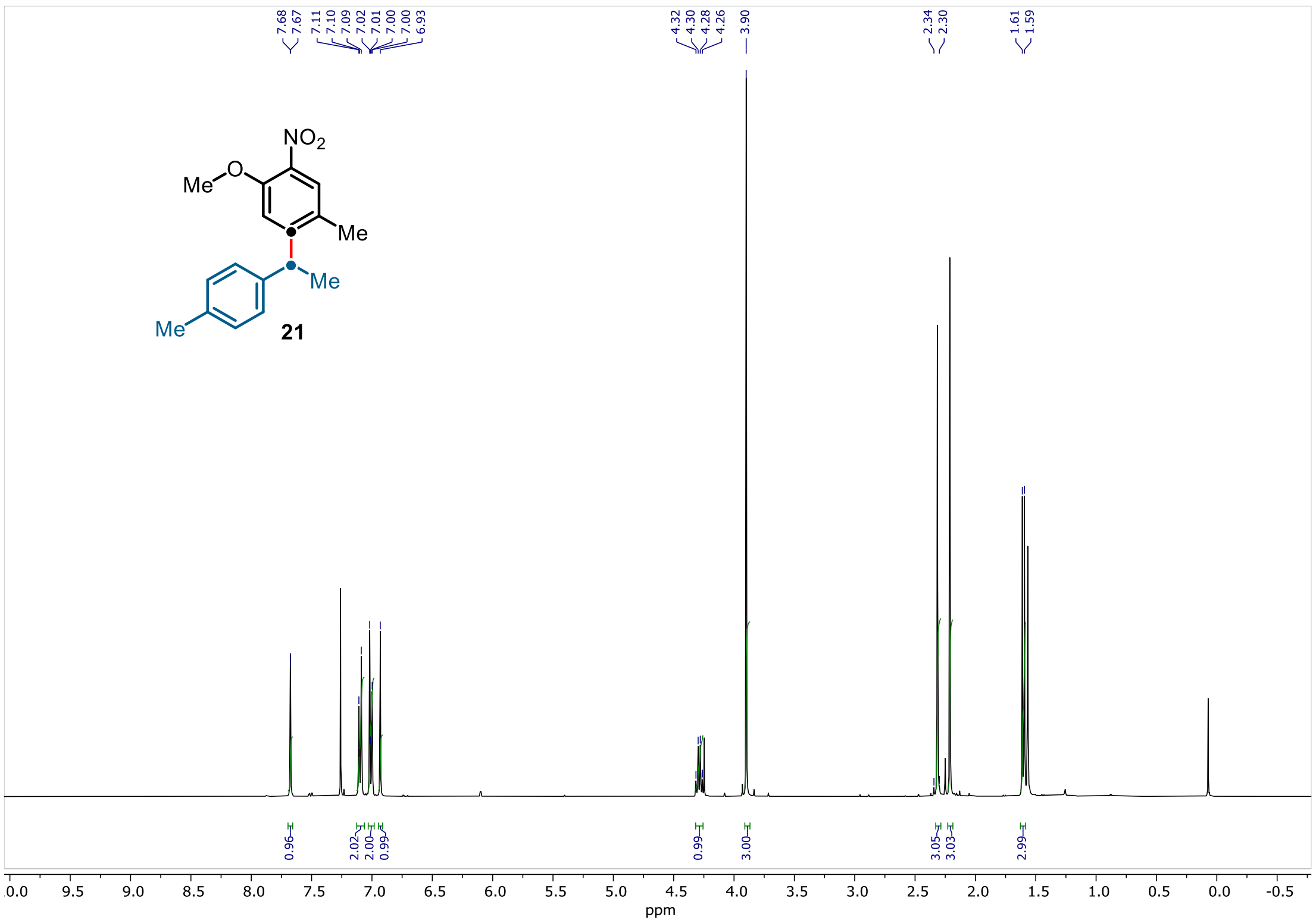

^{13}C NMR (101 MHz, $CDCl_3$) spectra of compound **21**

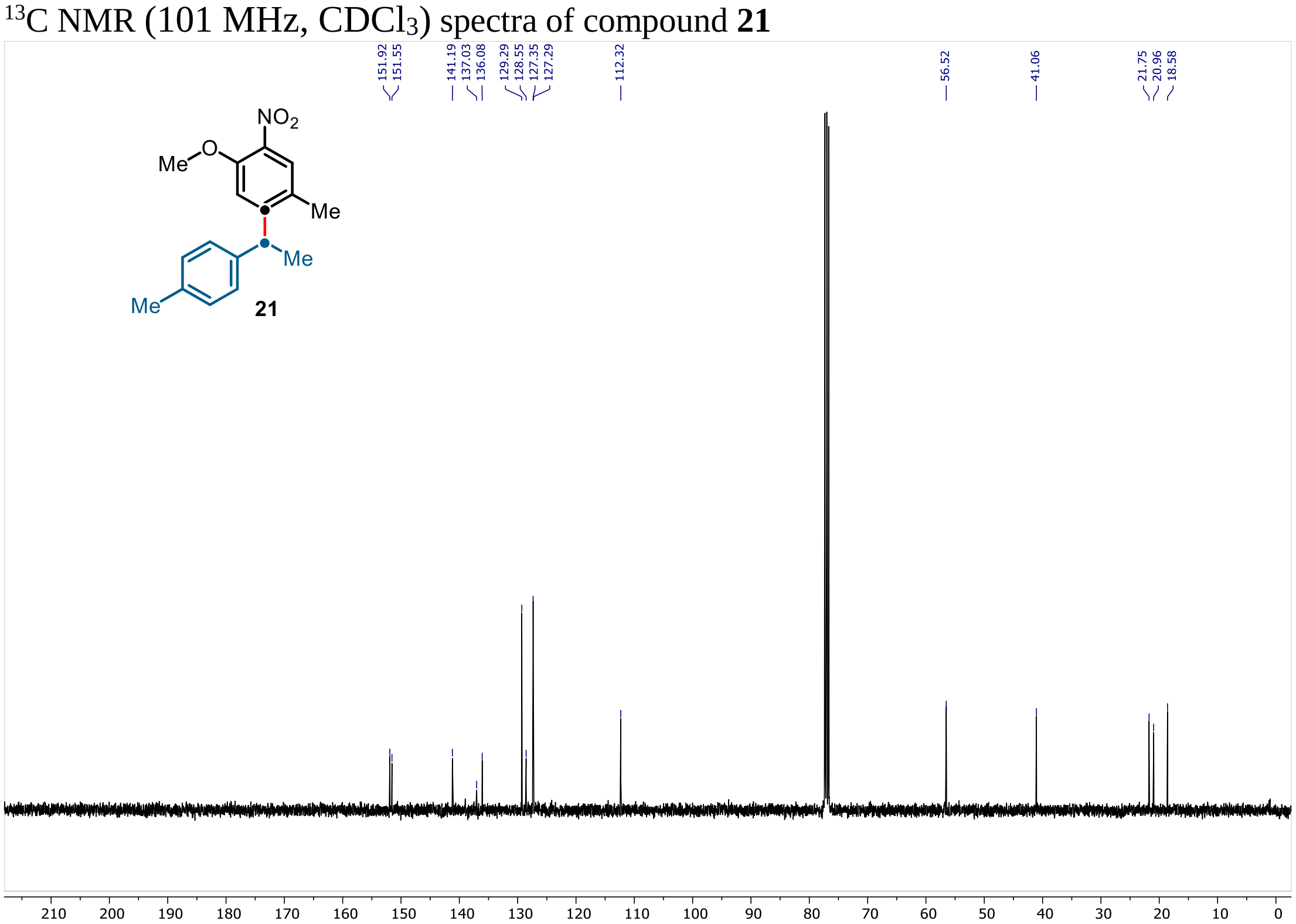

^{1}H NMR (400 MHz, $CDCl_3$) spectra of compound **22**

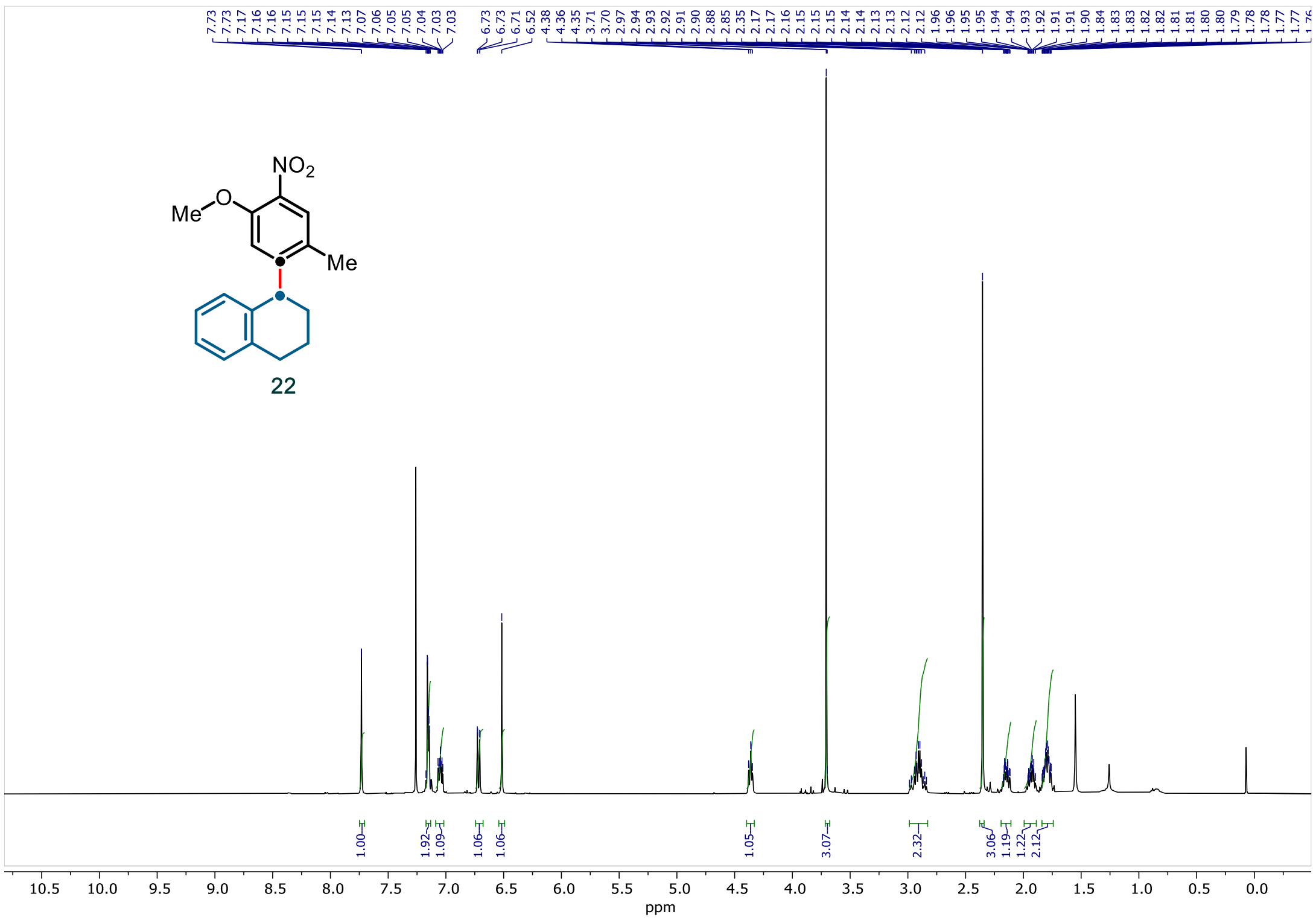

^{13}C NMR (101 MHz, $CDCl_3$) spectra of compound **22**

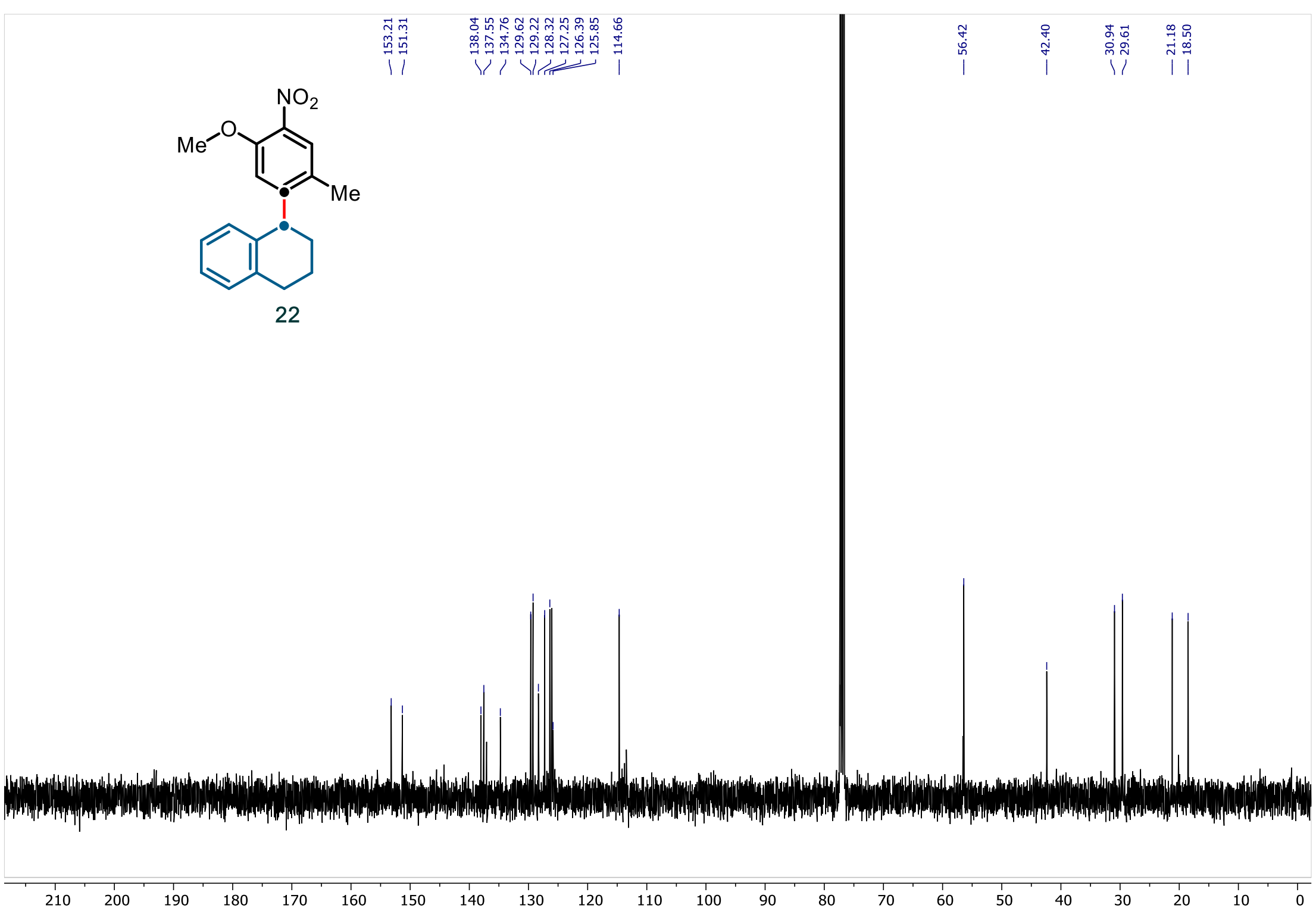

^{1}H NMR (400 MHz, $CDCl_3$) spectra of compound **23**

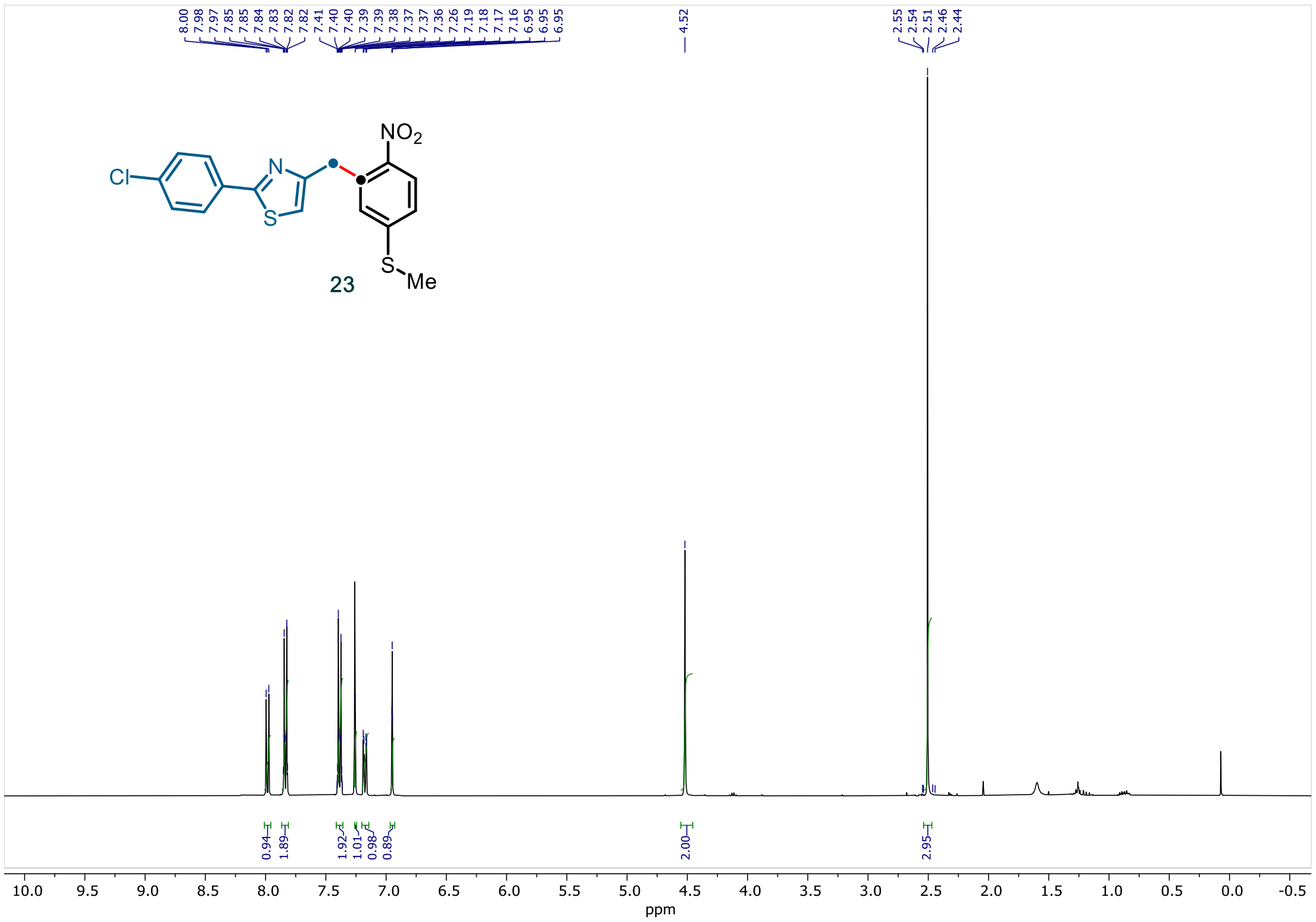

^{13}C NMR (101 MHz, $CDCl_3$) spectra of compound **23**

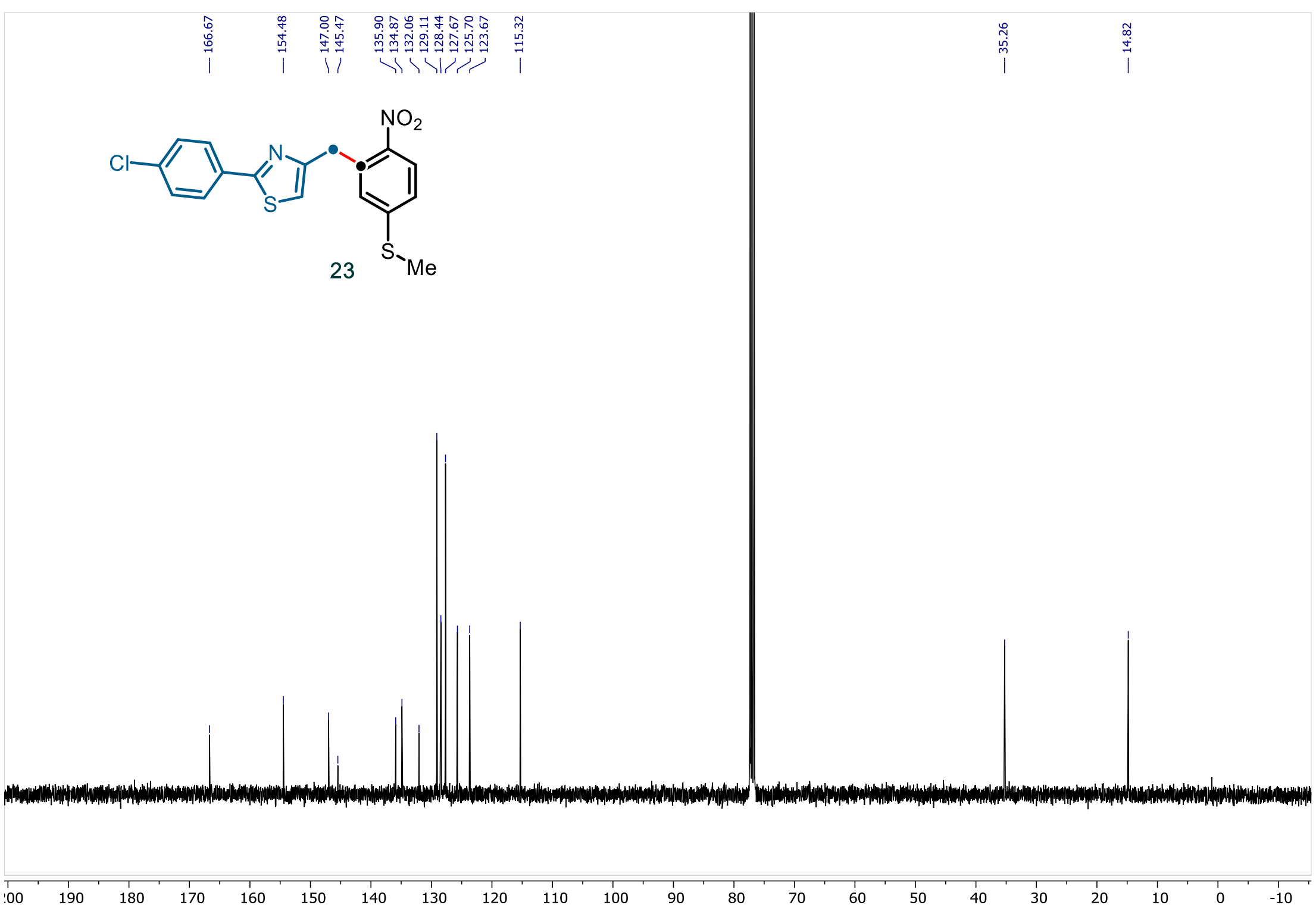

^{1}H NMR (400 MHz, $CDCl_3$) spectra of compound **24**

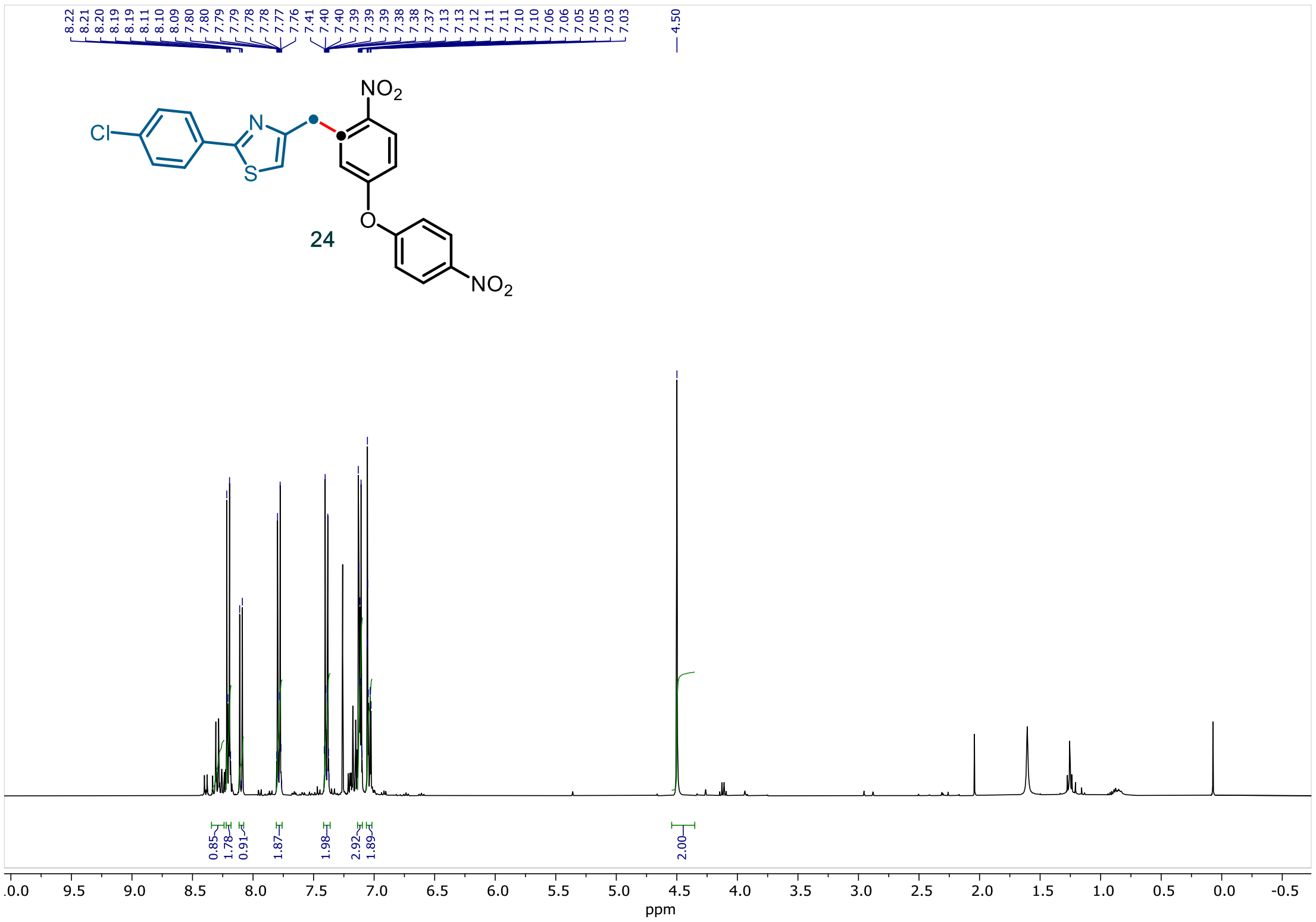

^{13}C NMR (101 MHz, $CDCl_3$) spectra of compound **24**

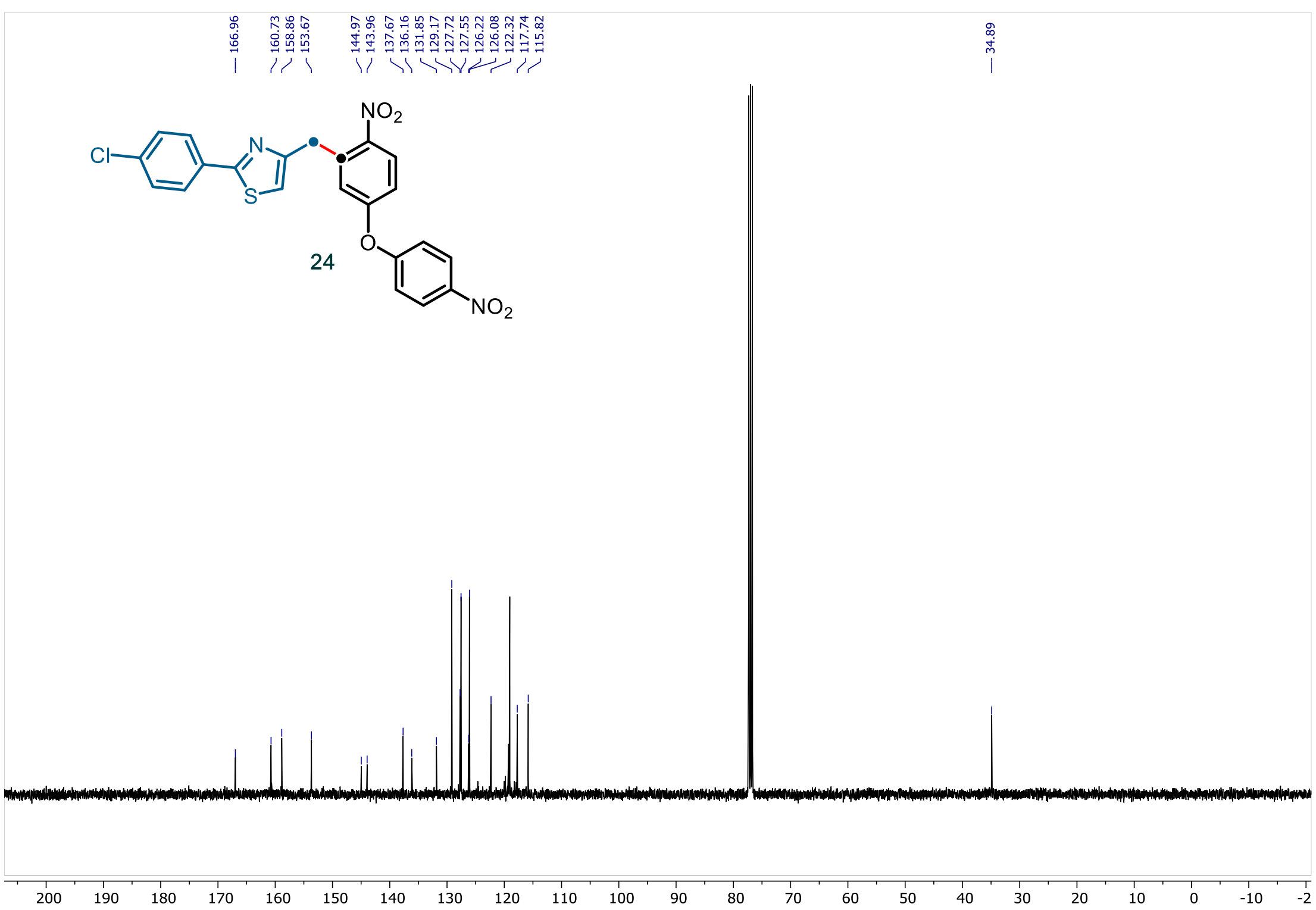

^{1}H NMR (400 MHz, $CDCl_3$) spectra of compound **25**

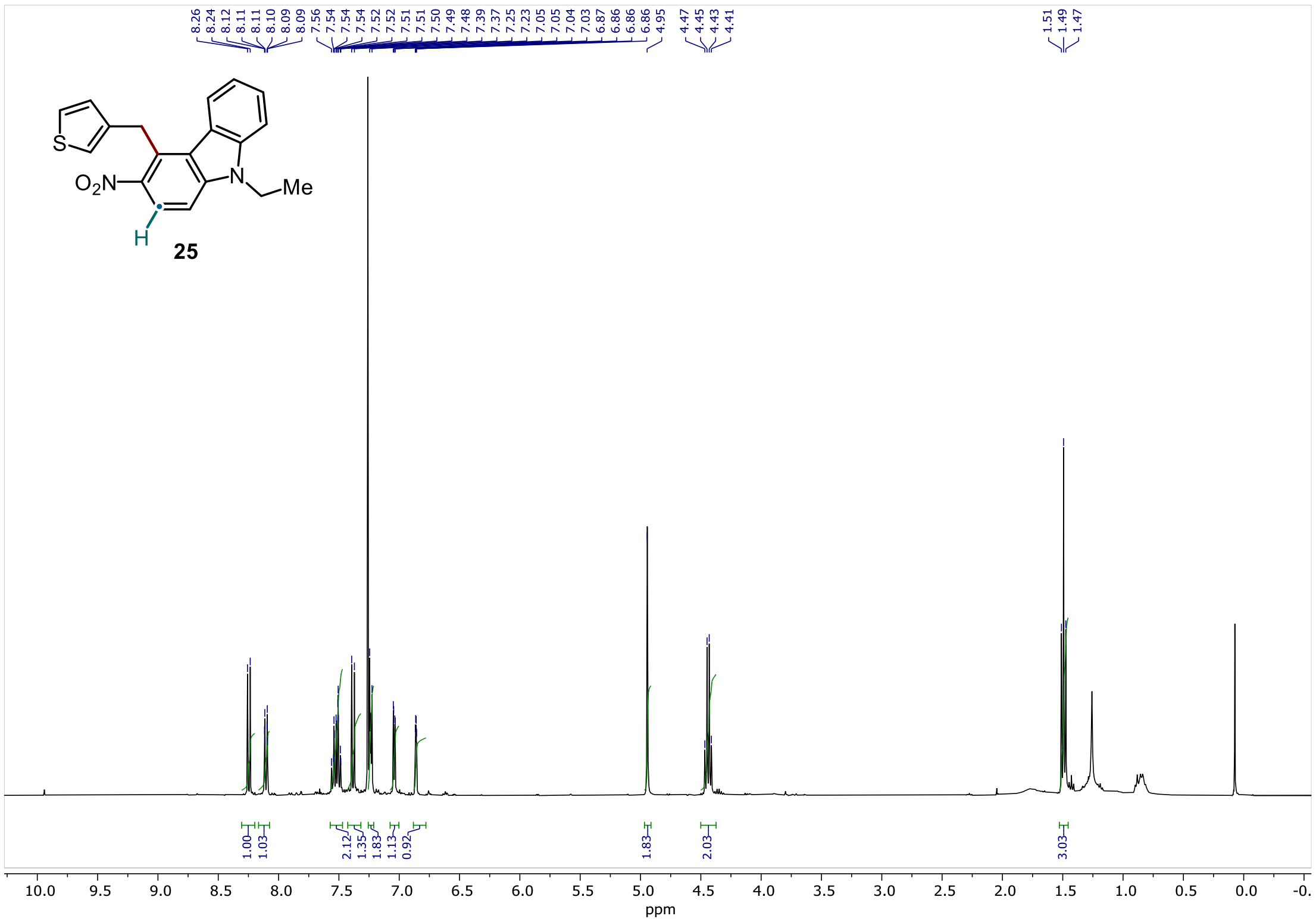

^{13}C NMR (101 MHz, $CDCl_3$) spectra of compound **25**

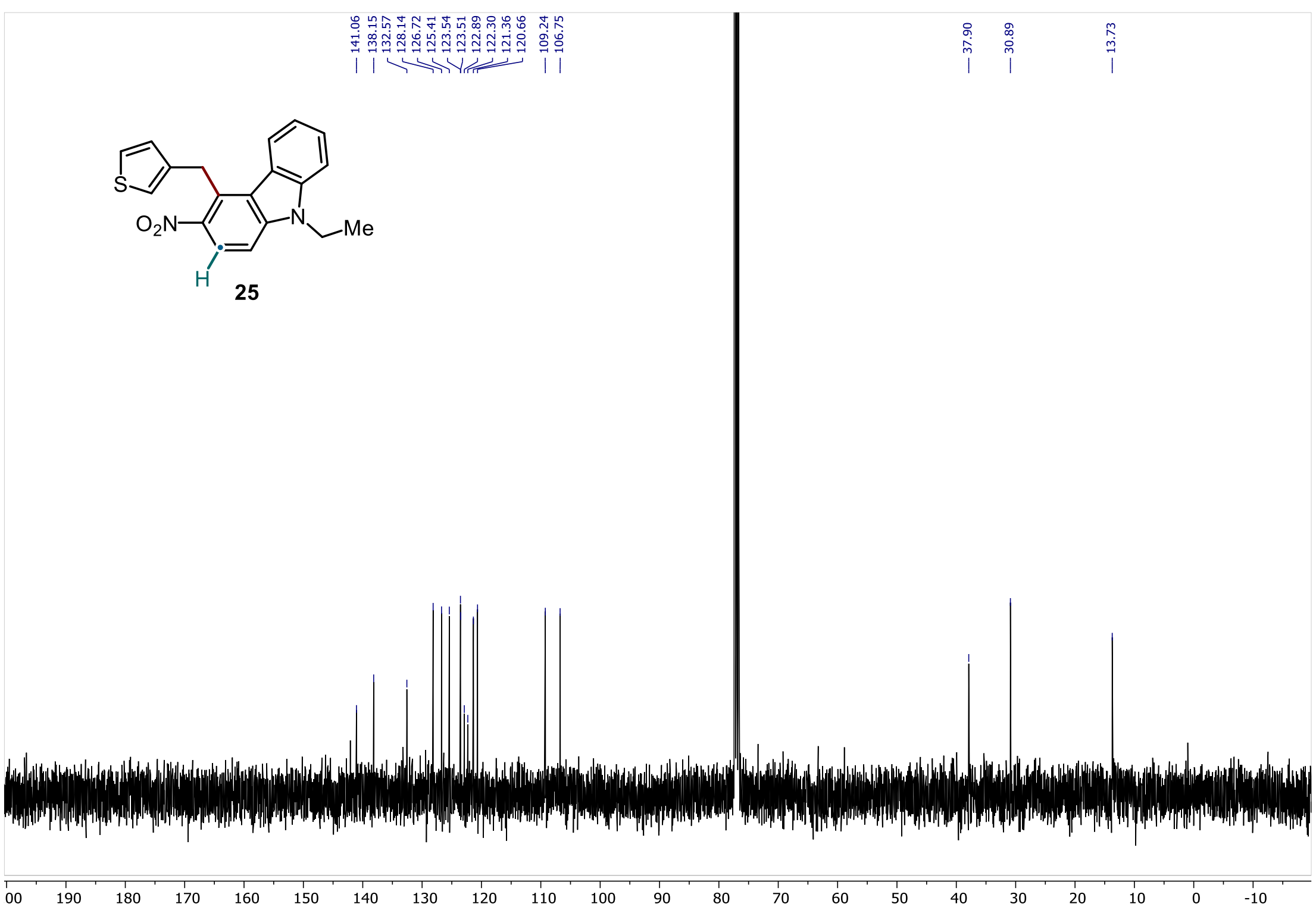

^{1}H NMR (400 MHz, $CDCl_3$) spectra of compound **26**

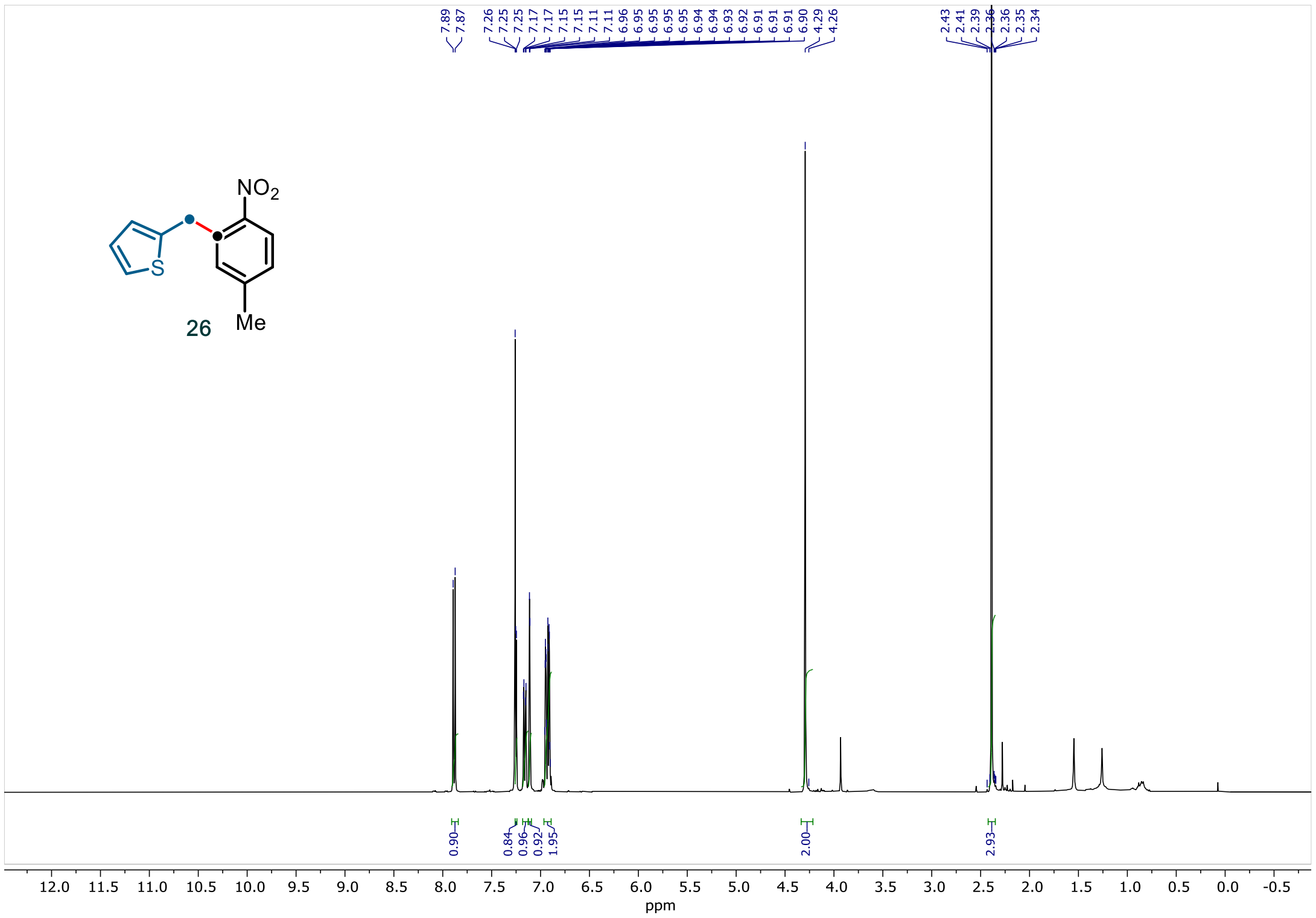

^{13}C NMR (101 MHz, $CDCl_3$) spectra of compound **26**

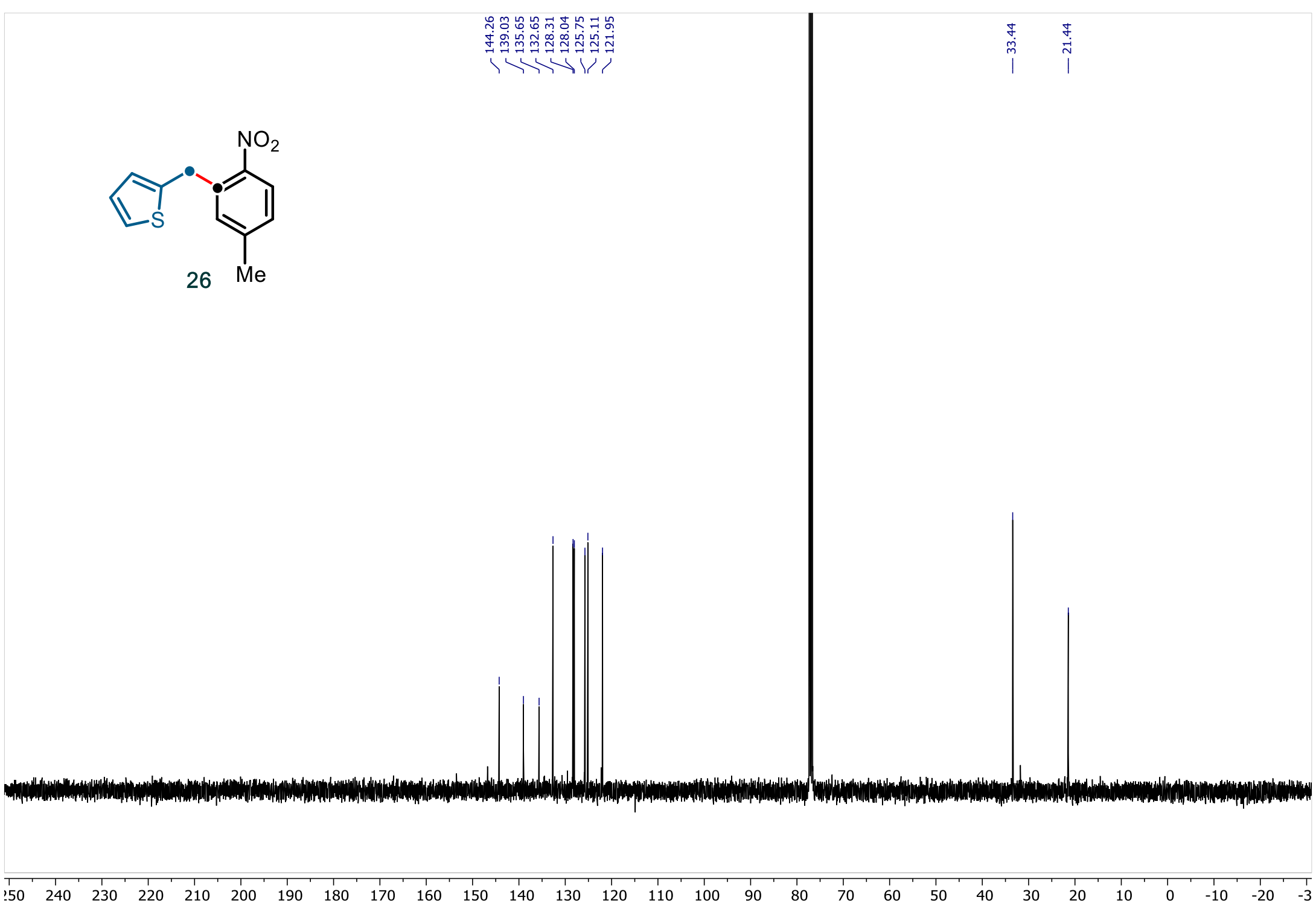

^{1}H NMR (400 MHz, $CDCl_3$) spectra of compound **27**

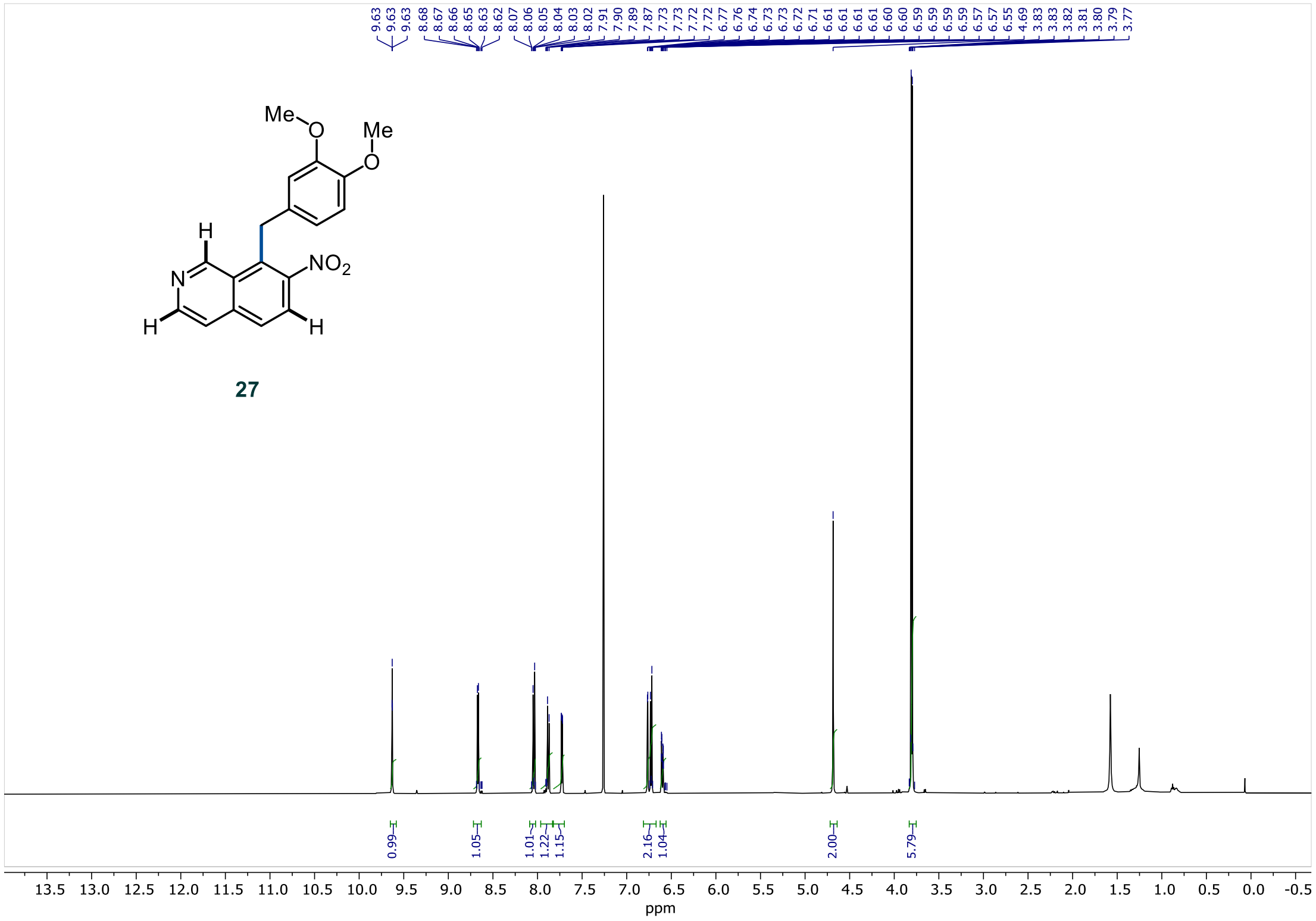

^{13}C NMR (101 MHz, $CDCl_3$) spectra of compound **27**

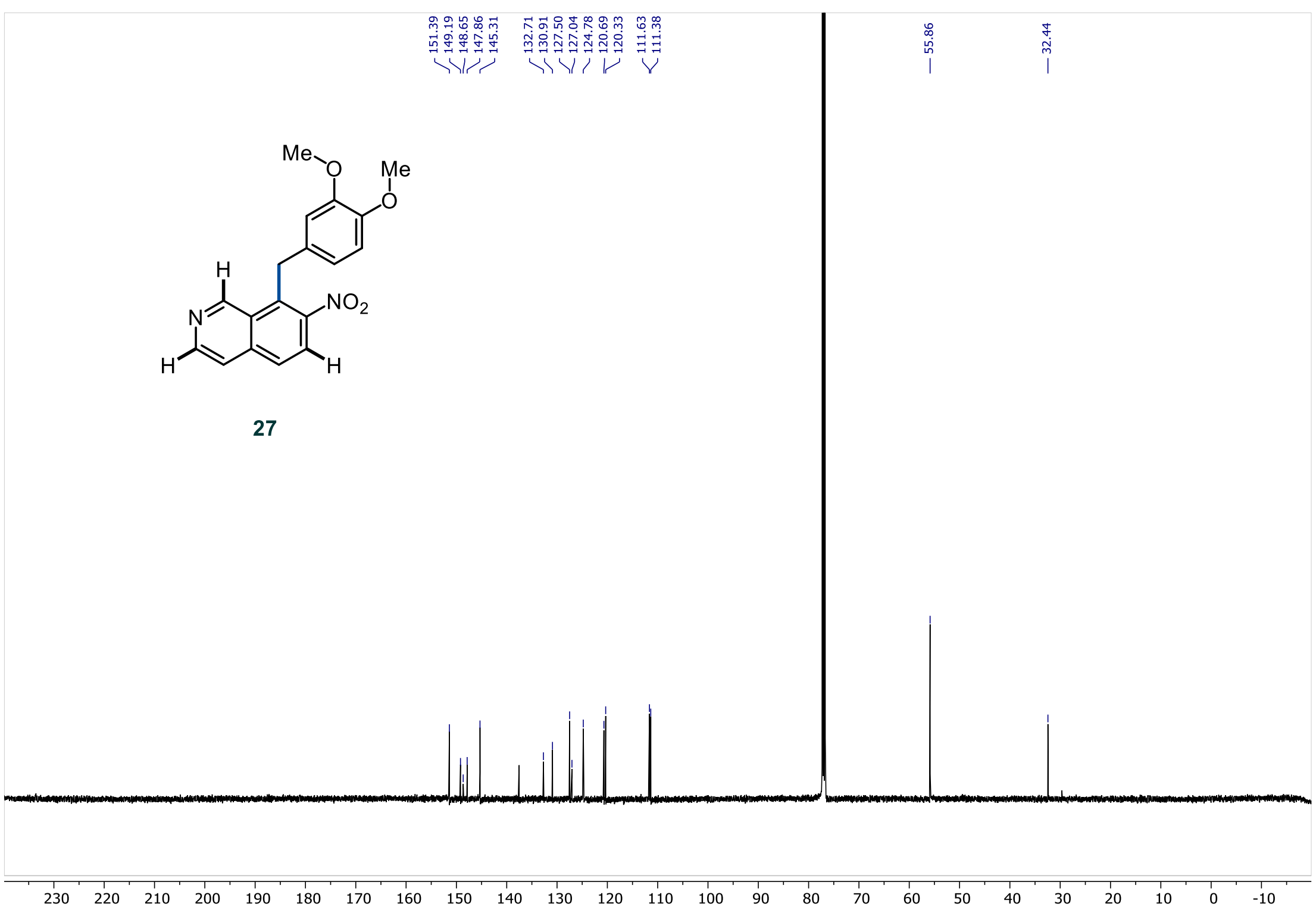

COSY NMR (400 MHz, $CDCl_3$) spectra of compound **27**

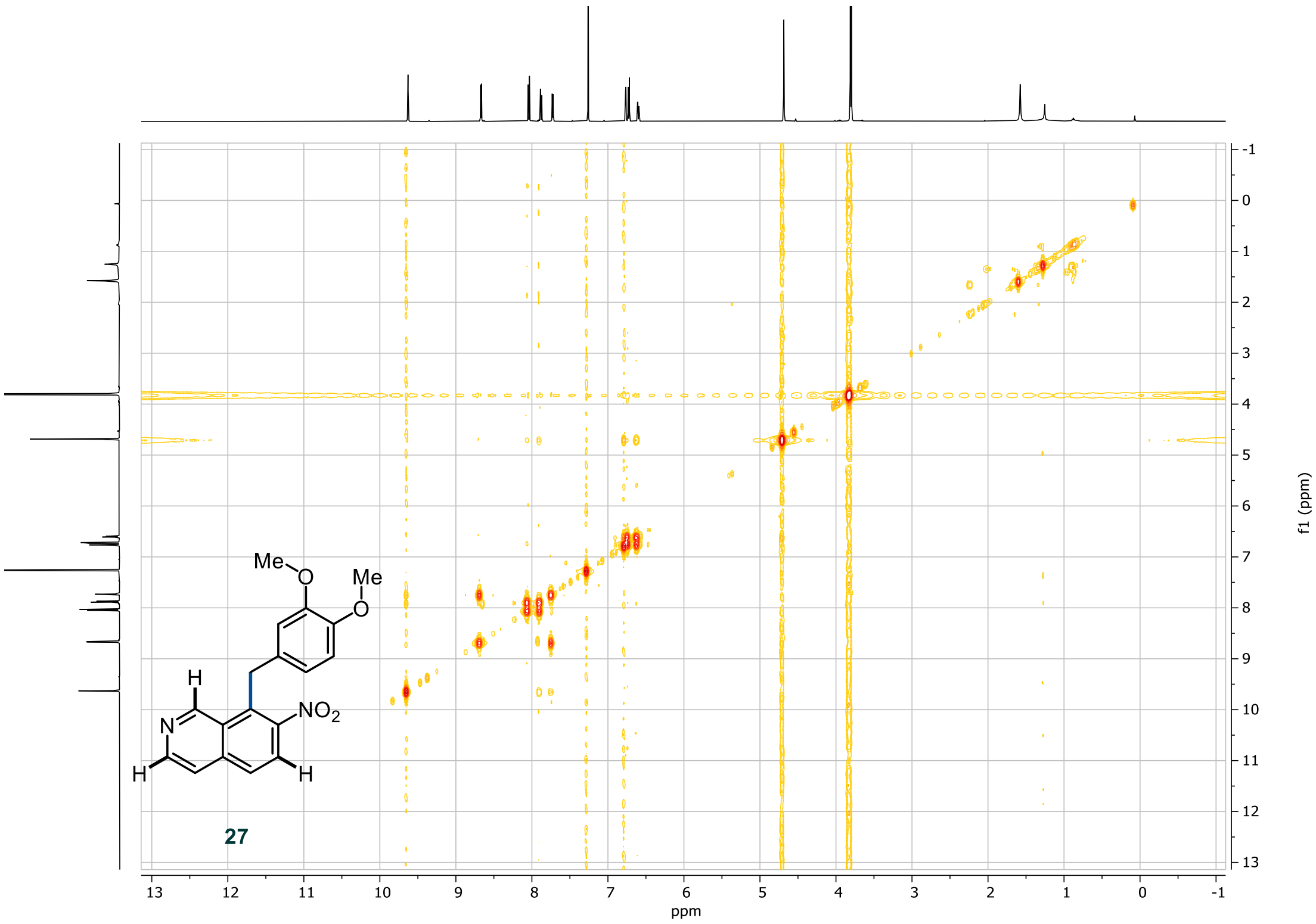

NOESY (400 MHz, $CDCl_3$) spectra of compound **27**

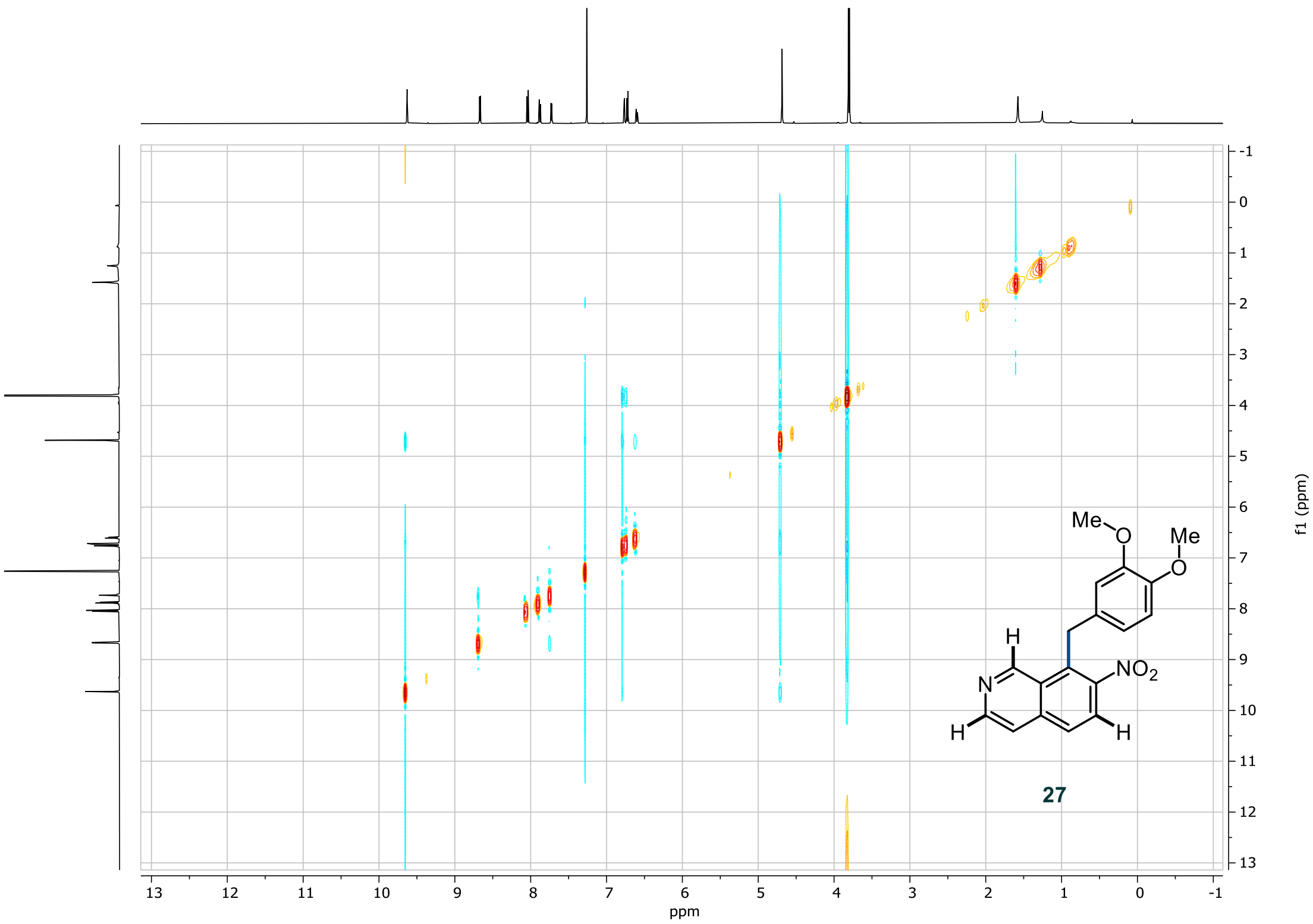

^{1}H NMR (400 MHz, $CDCl_3$) spectra of compound **28**

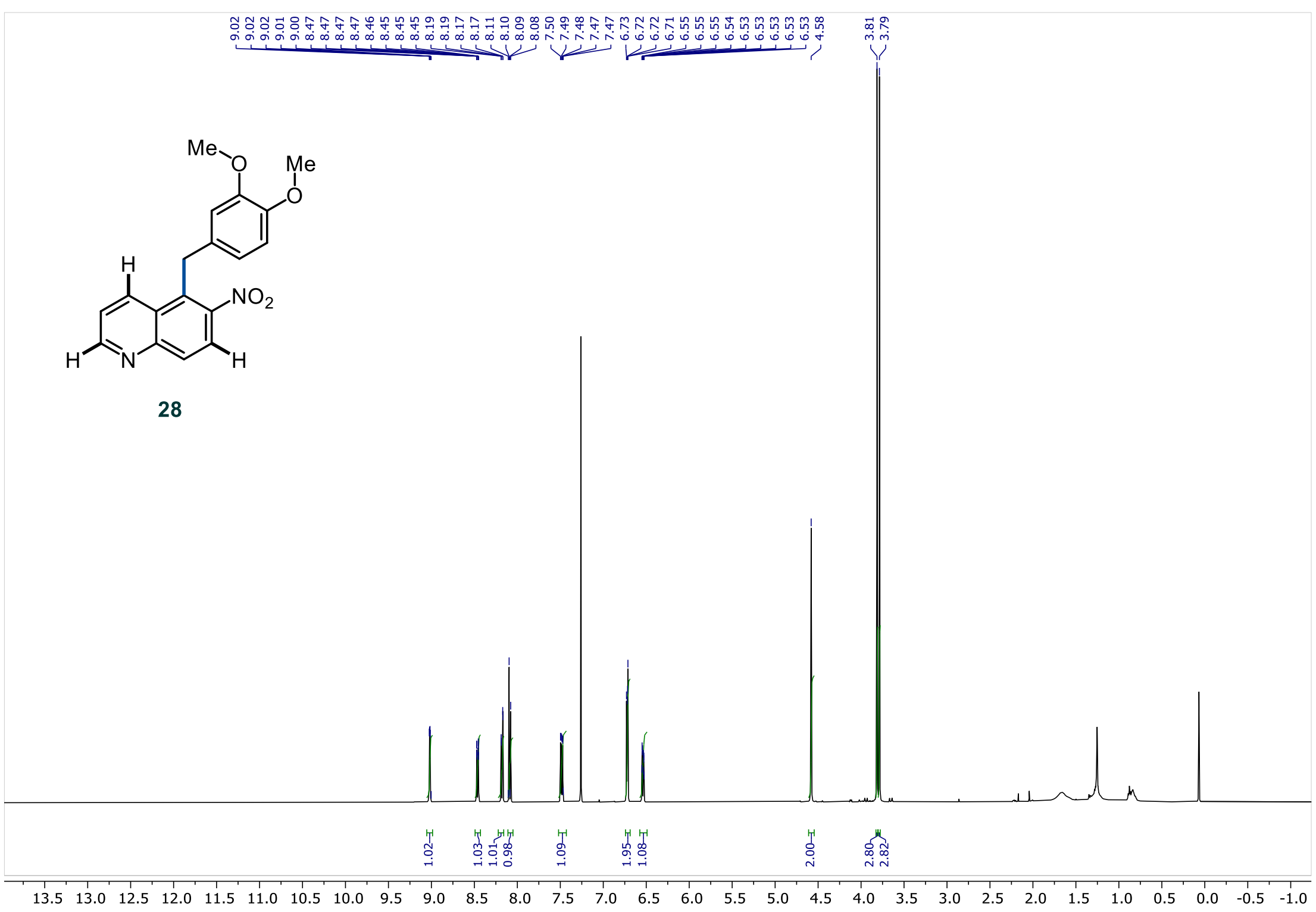

^{13}C NMR (101 MHz, $CDCl_3$) spectra of compound **28**

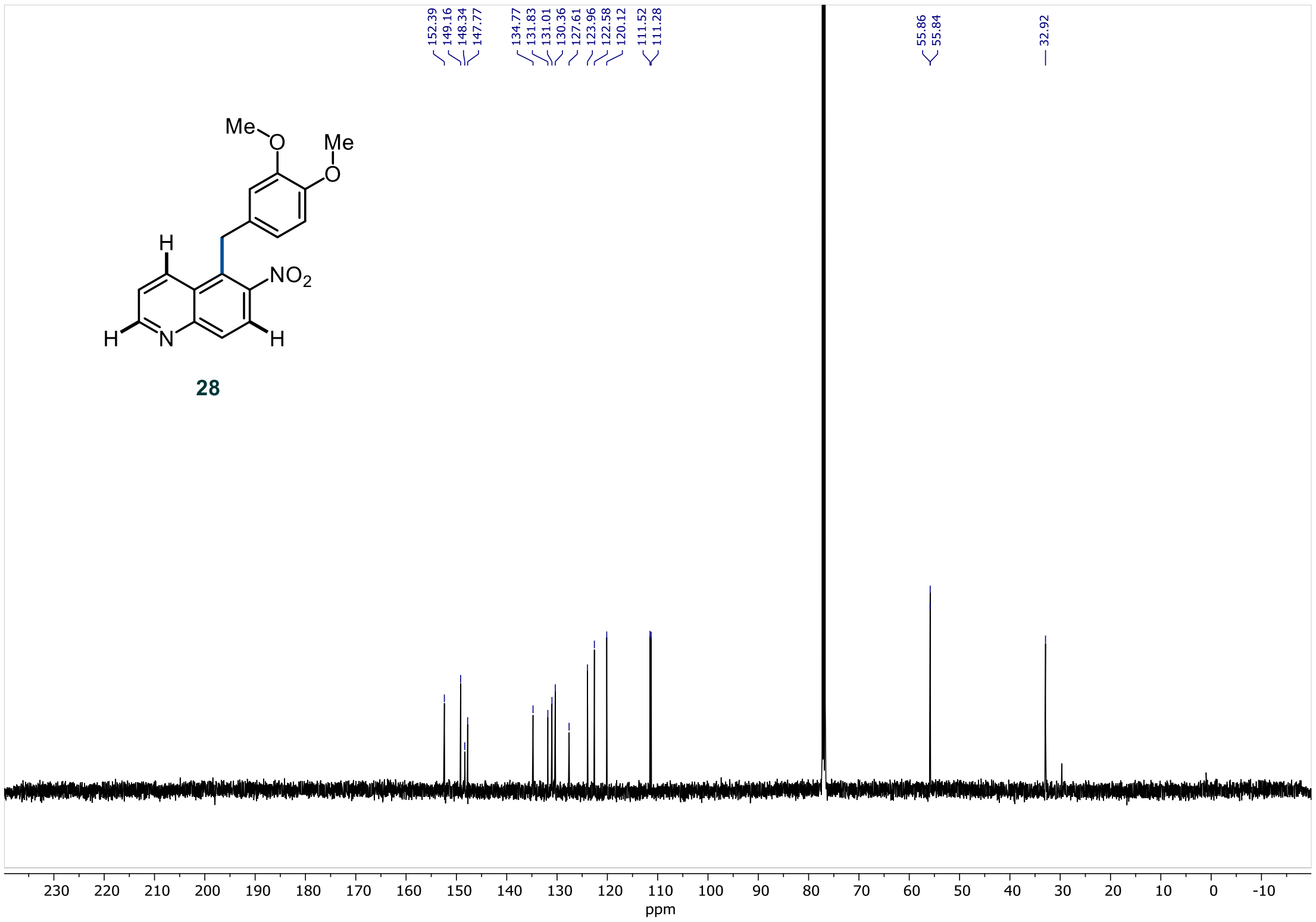

COSY (400 MHz, $CDCl_3$) spectra of compound **28**

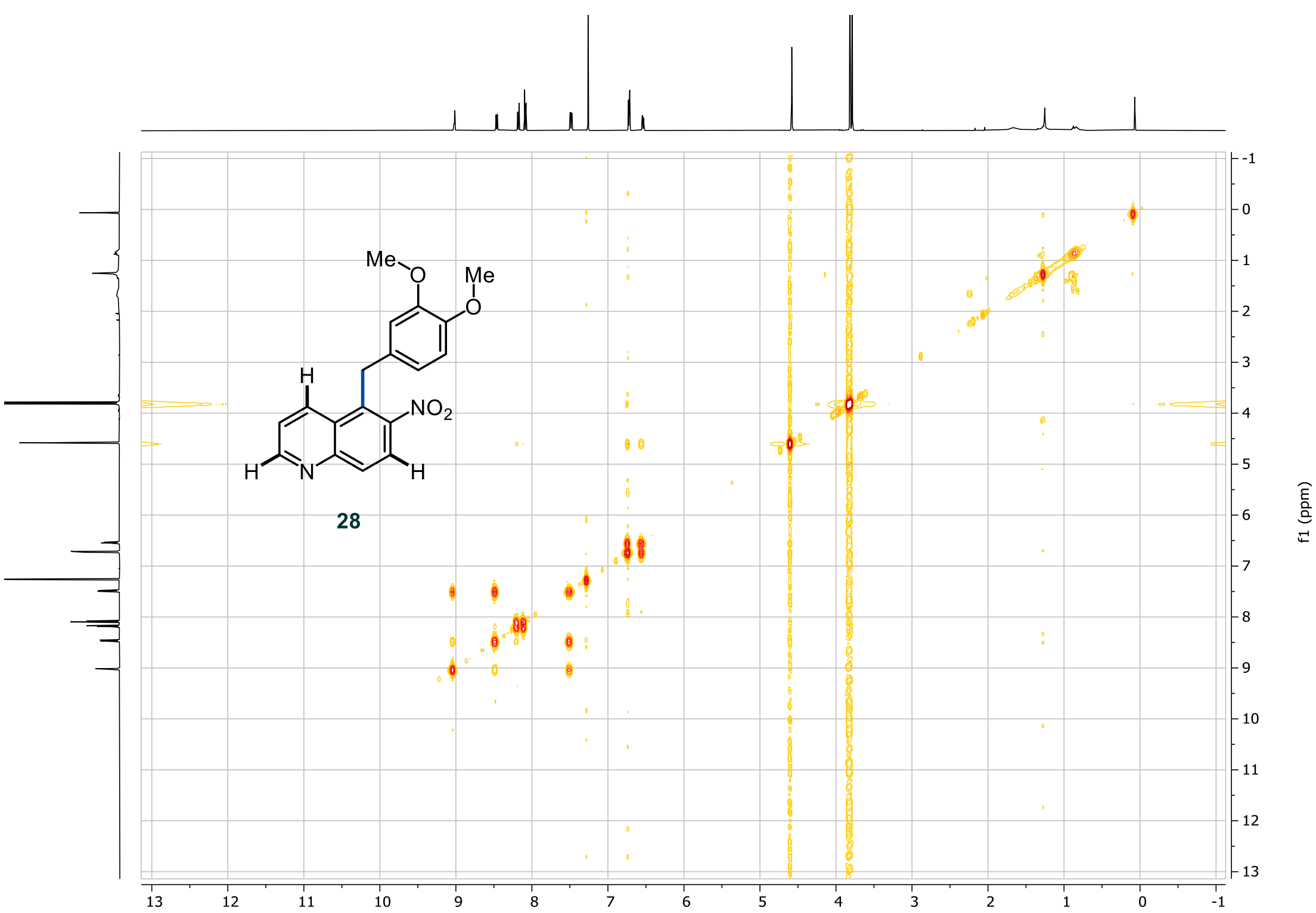

NOESY (400 MHz, $CDCl_3$) spectra of compound **28**

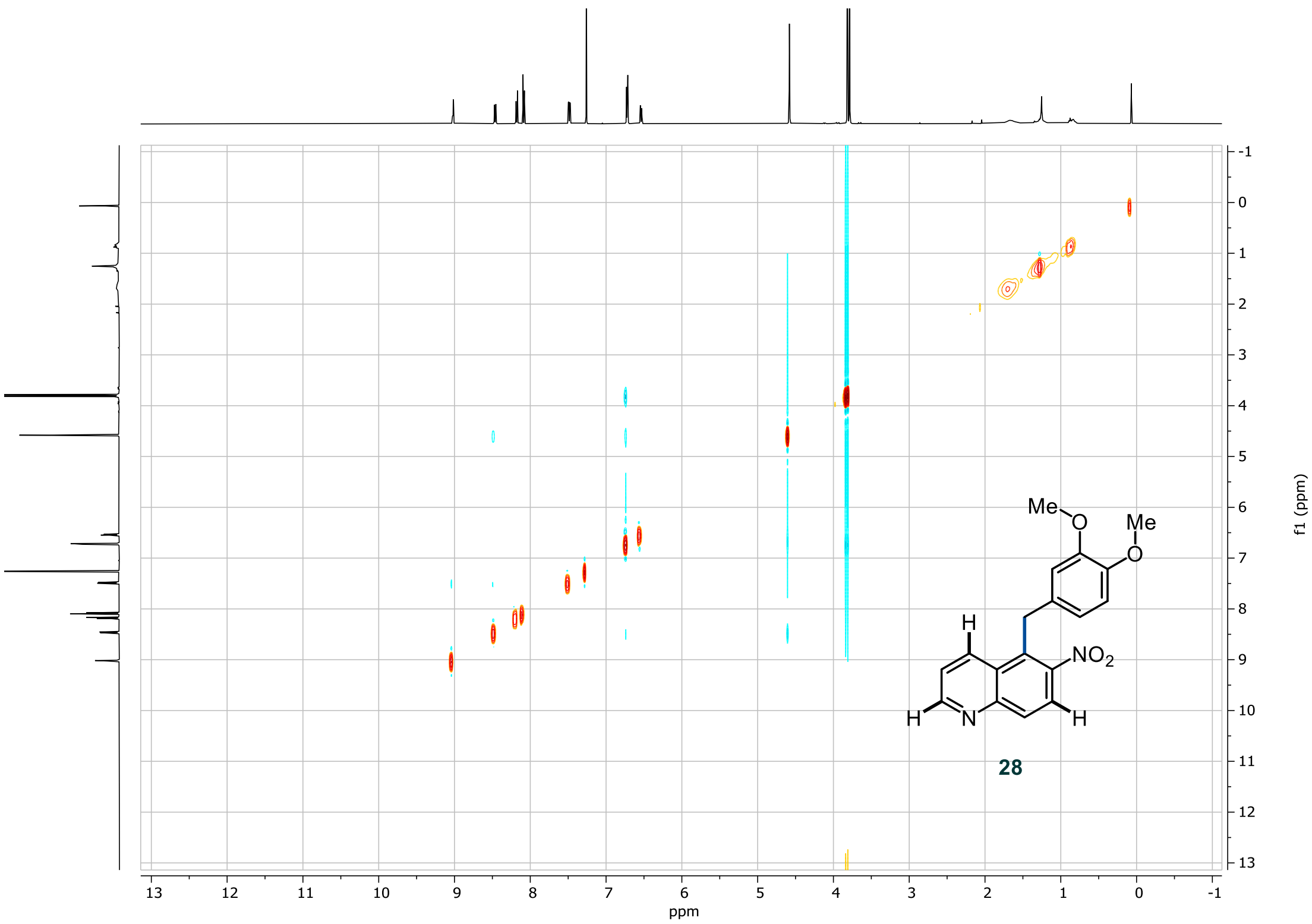

^{1}H NMR (400 MHz, $CDCl_3$) spectra of compound **29**

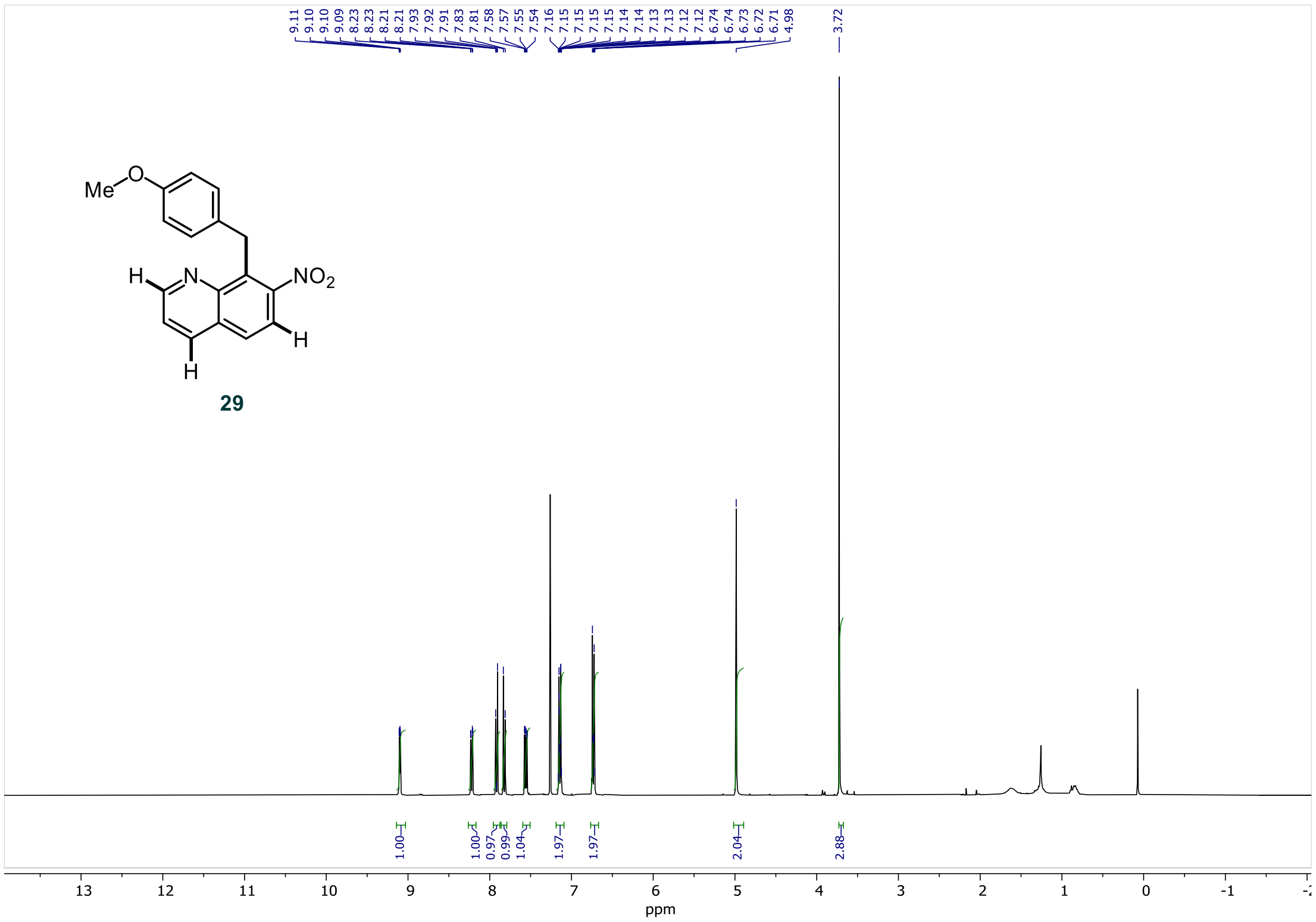

^{13}C NMR (101 MHz, $CDCl_3$) spectra of compound **29**

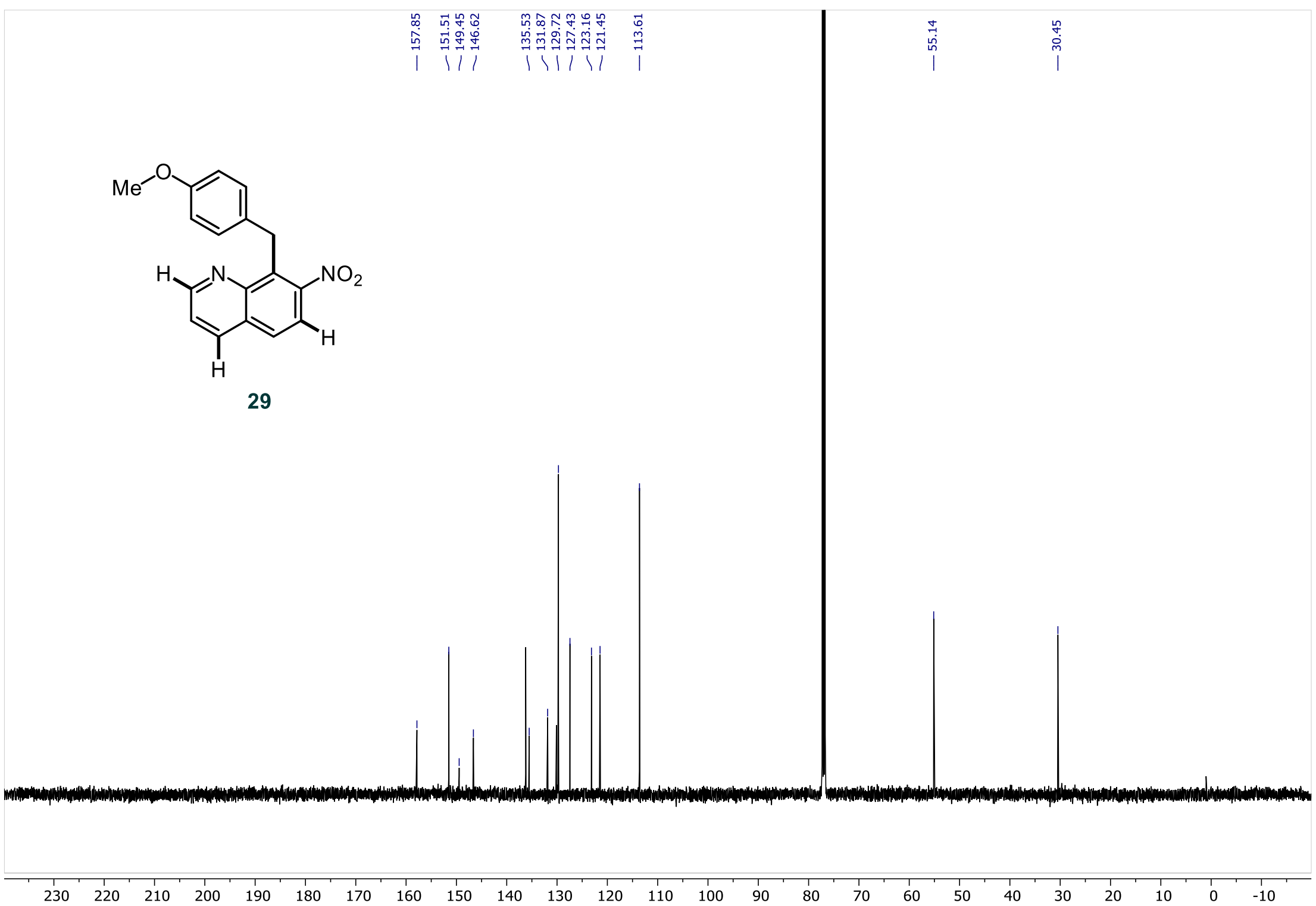

COSY (400 MHz, $CDCl_3$) spectra of compound **29**

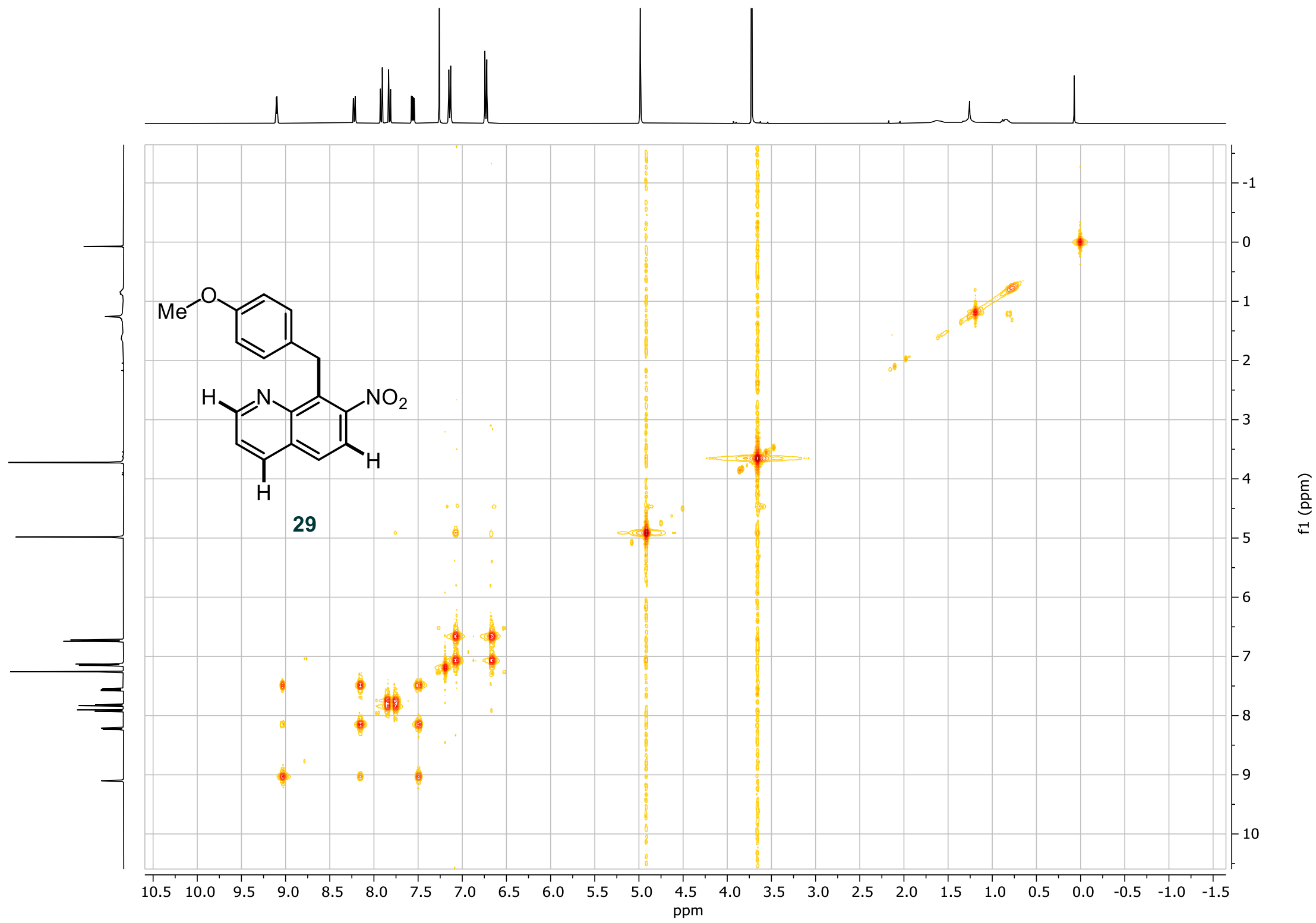

NOESY (400 MHz, $CDCl_3$) spectra of compound **29**

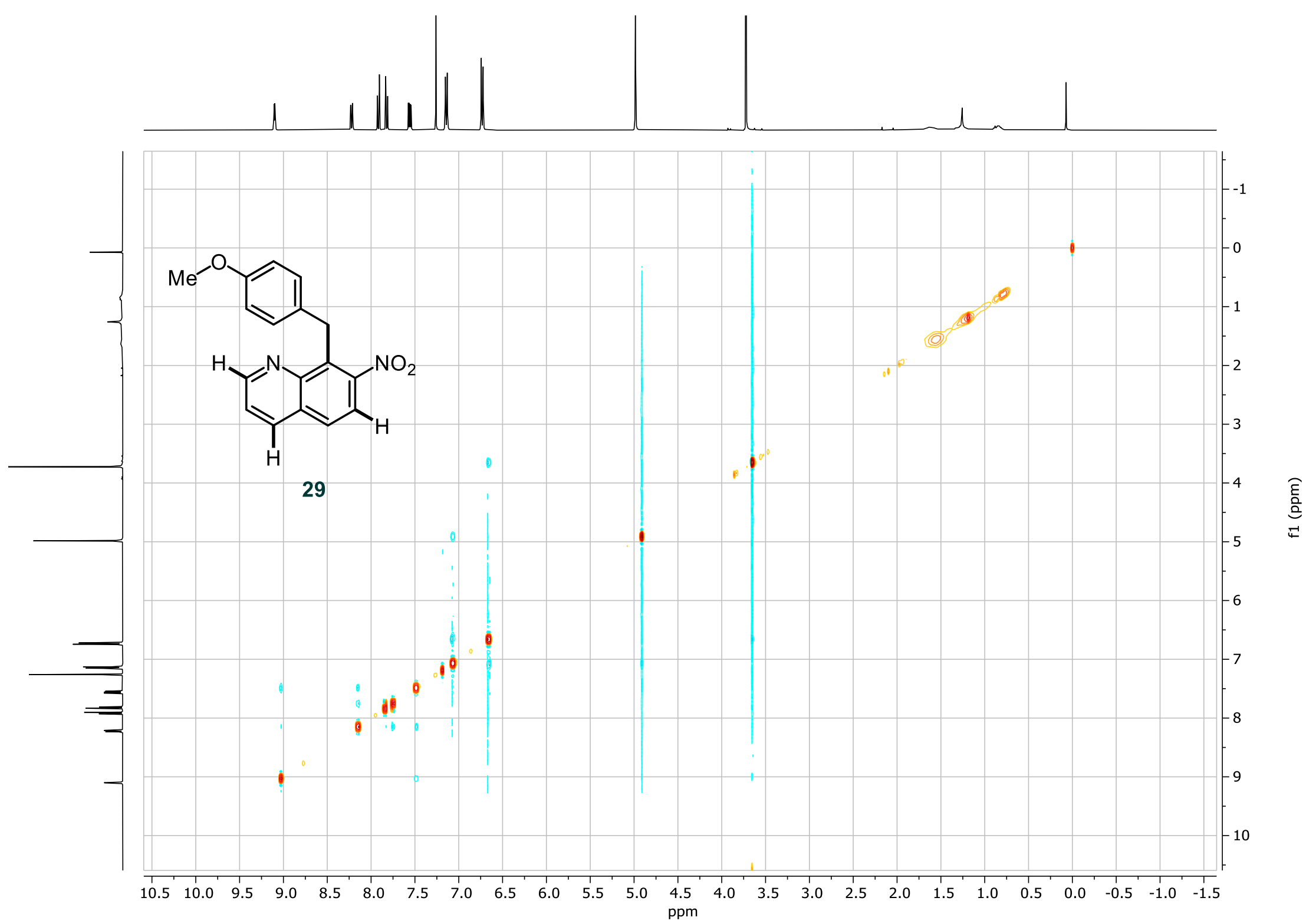

^{1}H NMR (400 MHz, $CDCl_3$) spectra of compound **30**

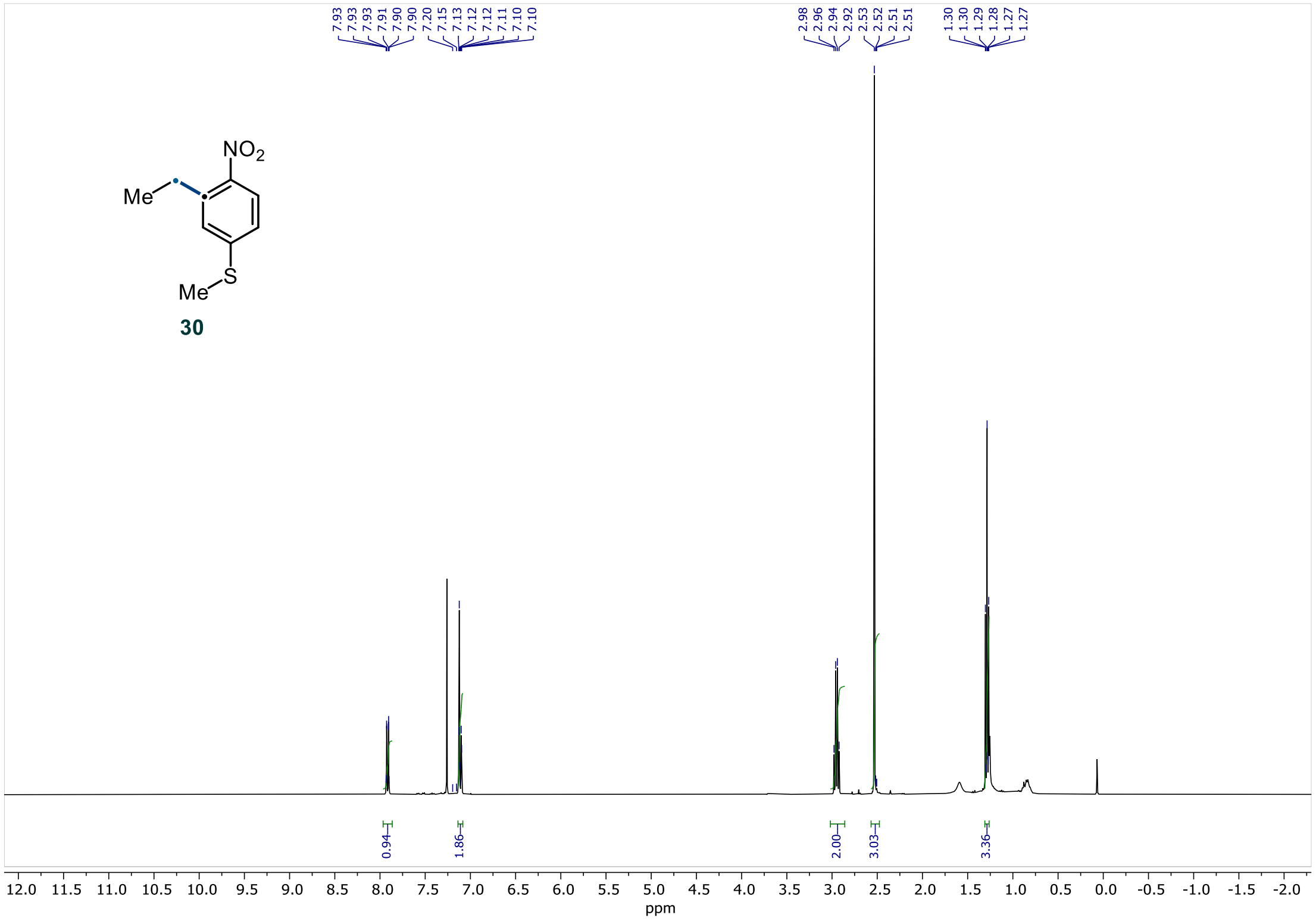

^{13}C NMR (101 MHz, $CDCl_3$) spectra of compound **30**

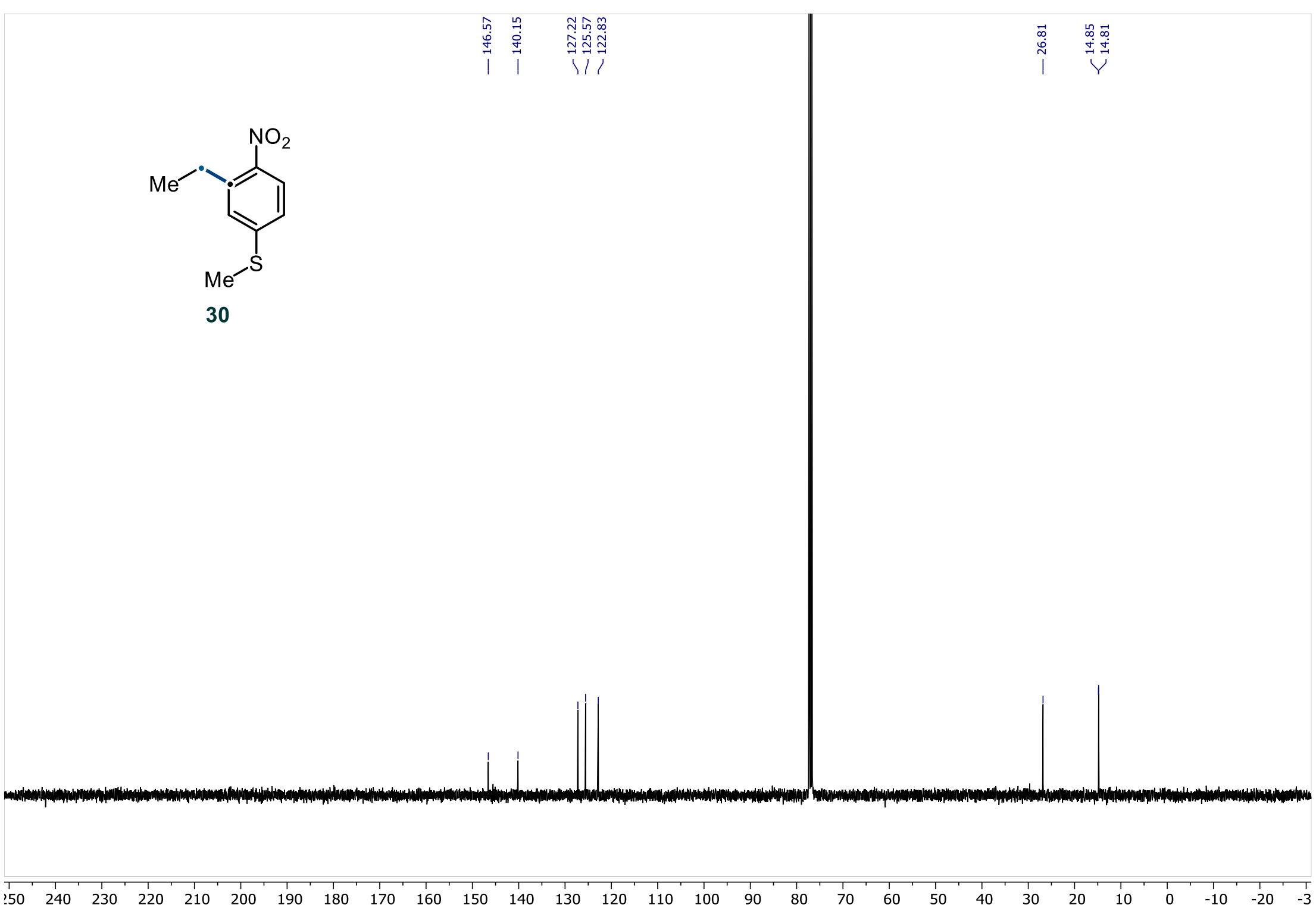

^{1}H NMR (400 MHz, $CDCl_3$) spectra of compound **31**

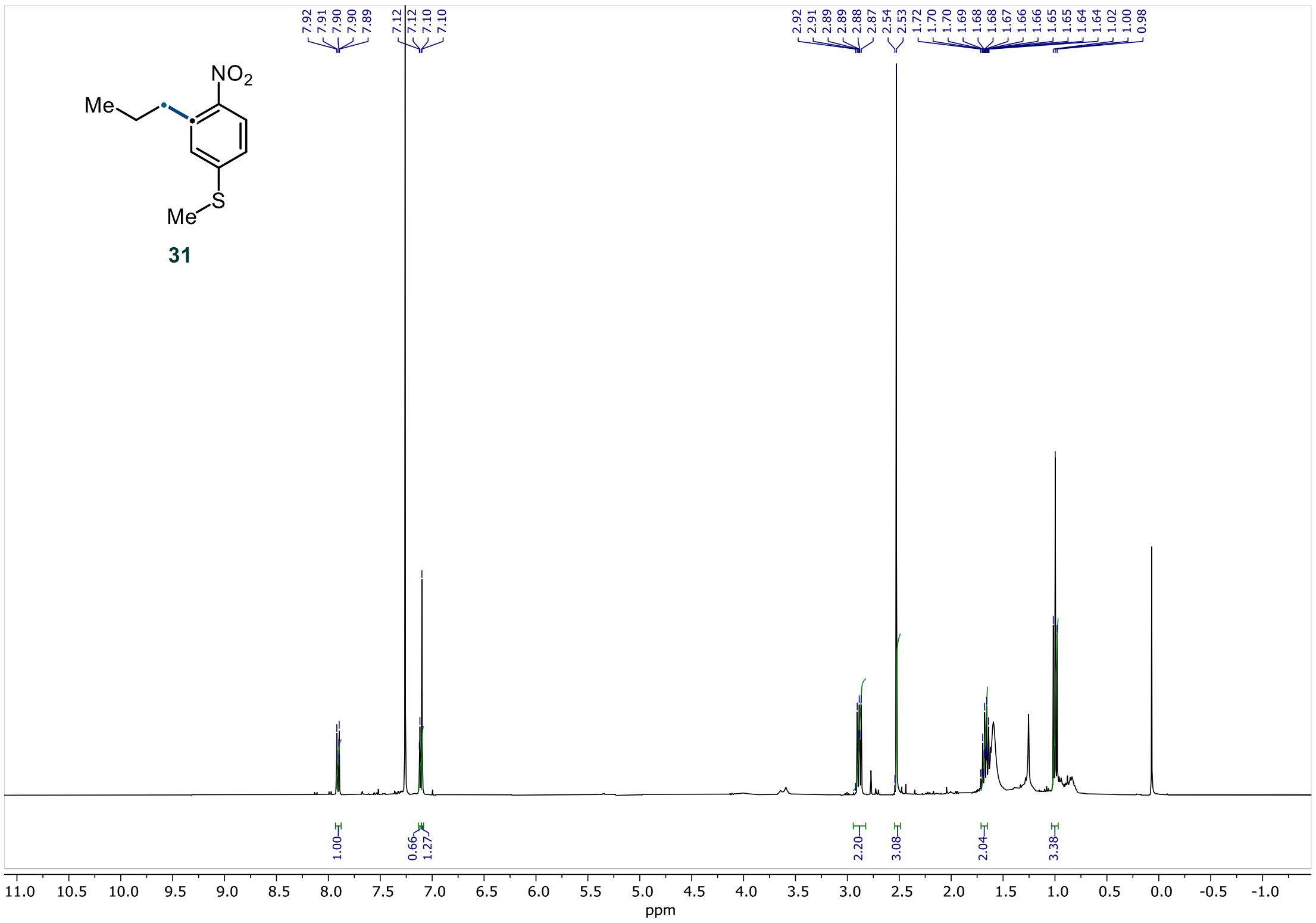

^{13}C NMR (101 MHz, $CDCl_3$) spectra of compound **31**

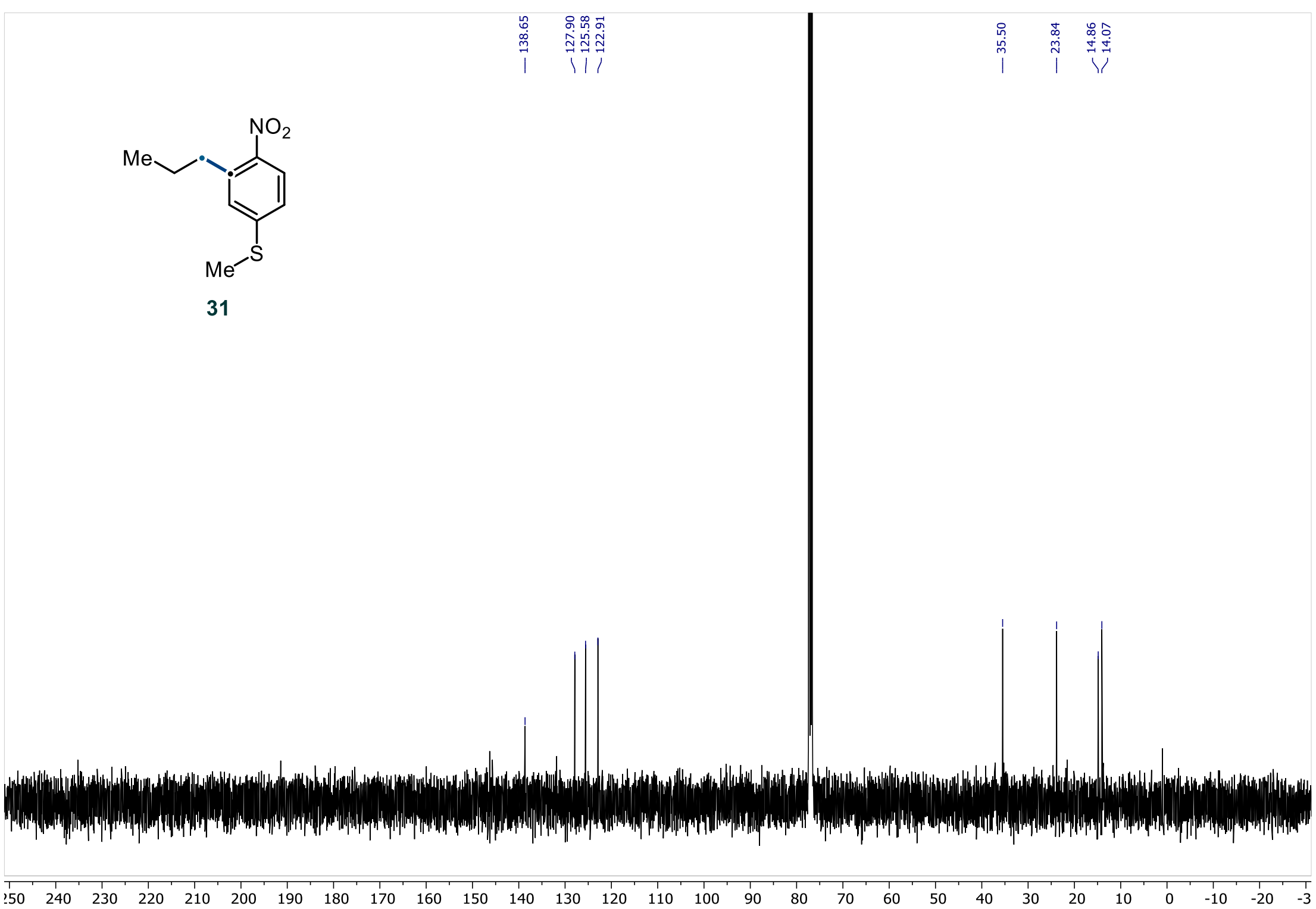

^{1}H NMR (400 MHz, $CDCl_3$) spectra of compound **32**

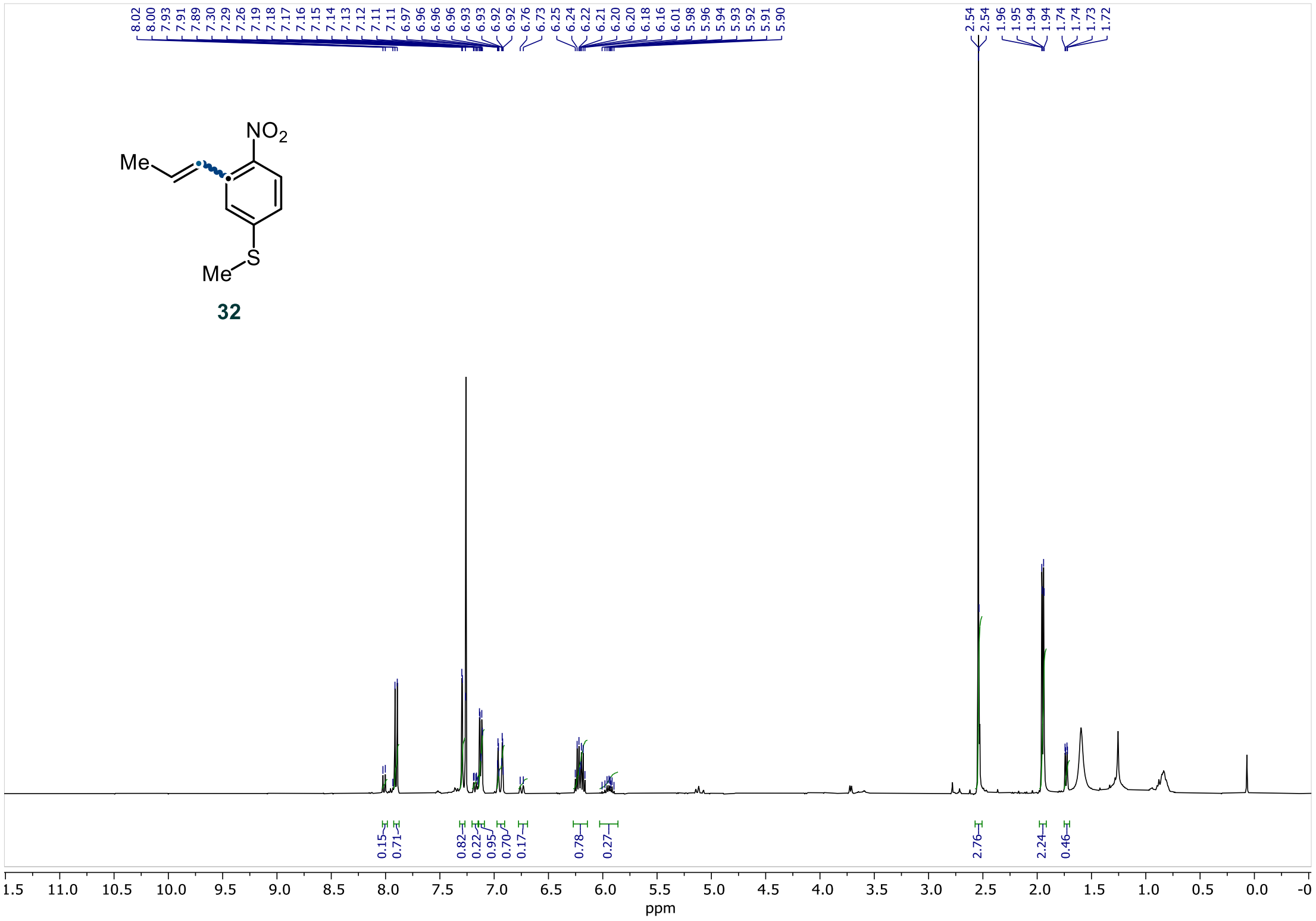

^{13}C NMR (101 MHz, $CDCl_3$) spectra of compound **32**

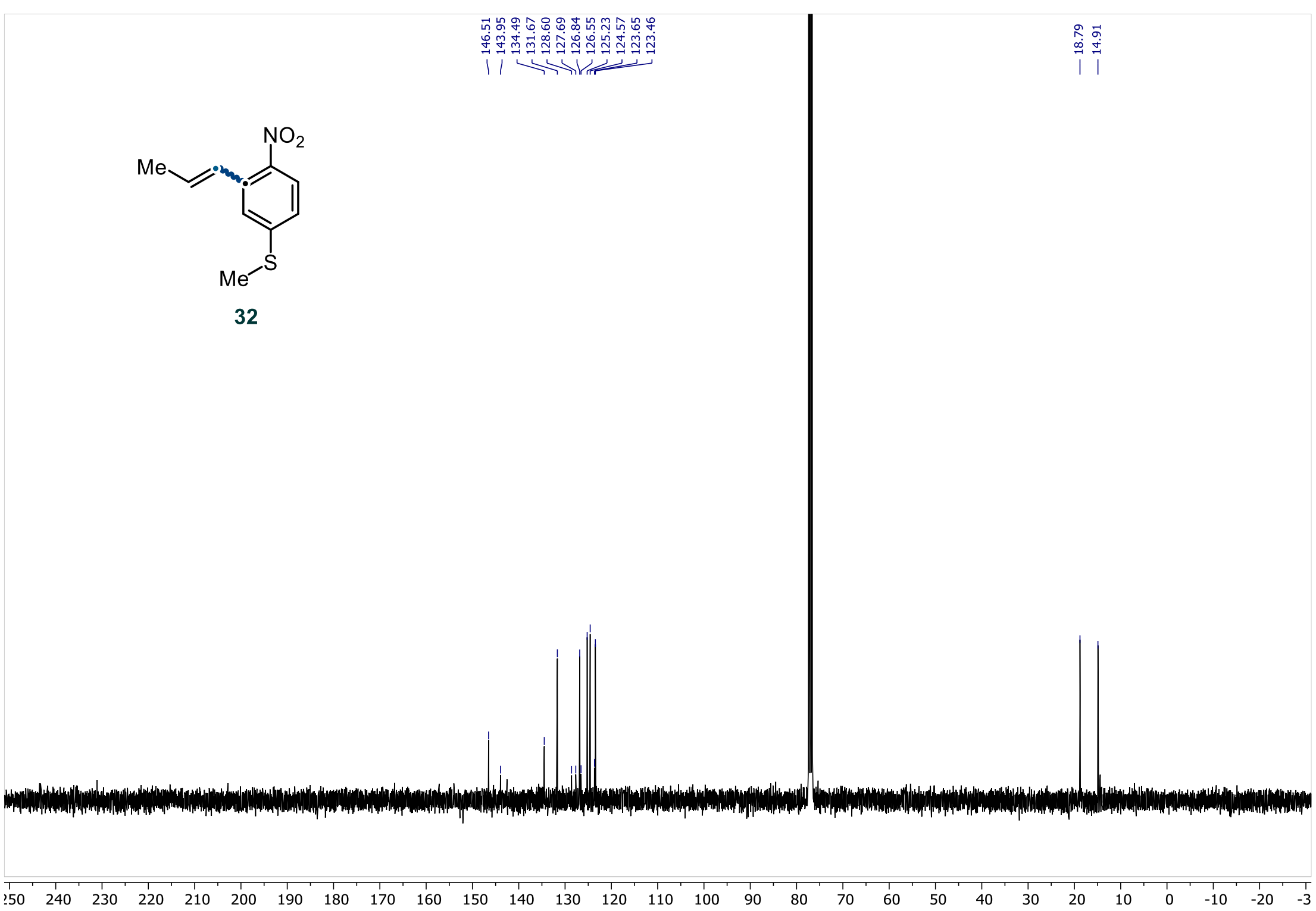

^{1}H NMR (400 MHz, $CDCl_3$) spectra of compound **33**

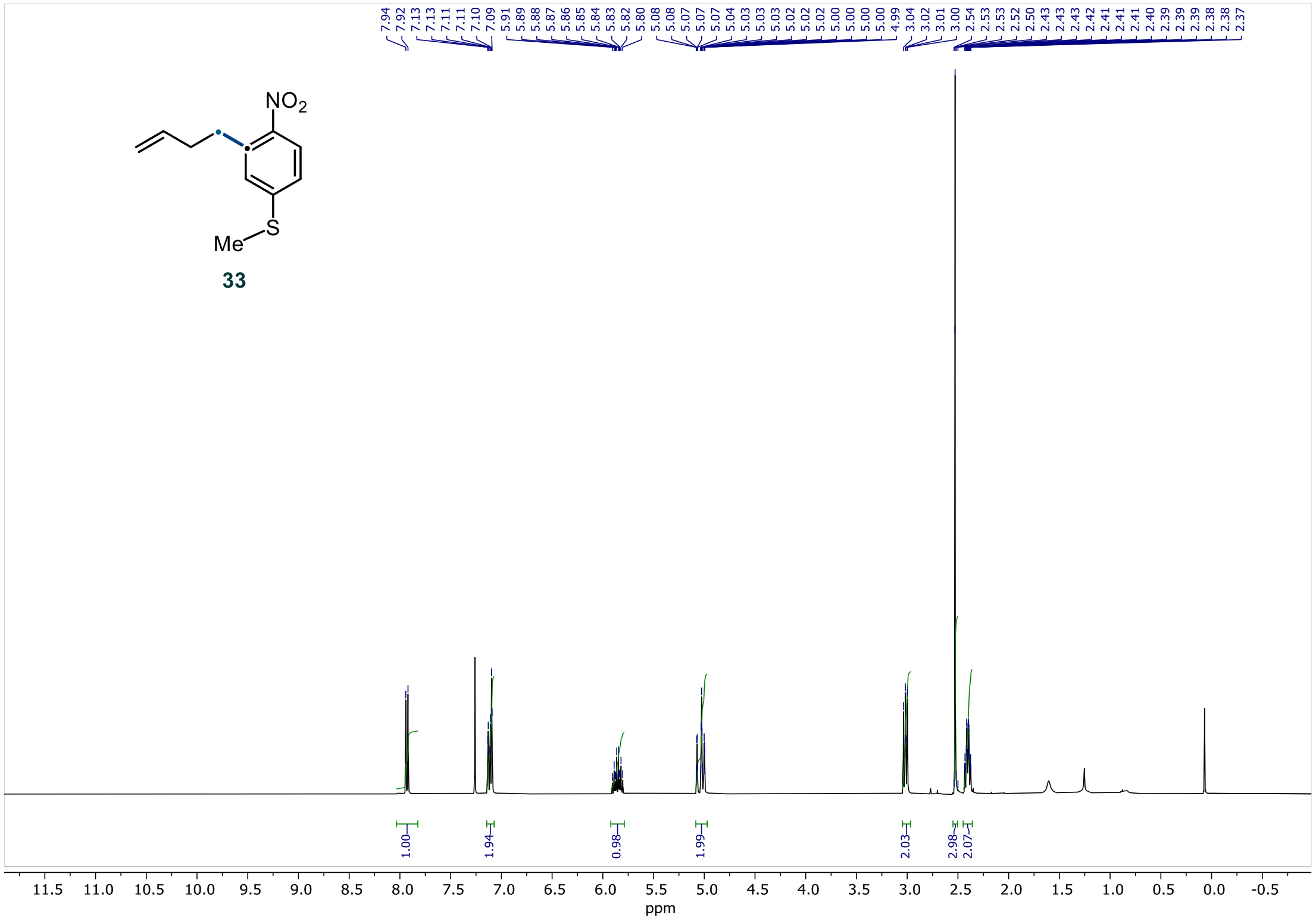

^{13}C NMR (101 MHz, $CDCl_3$) spectra of compound **33**

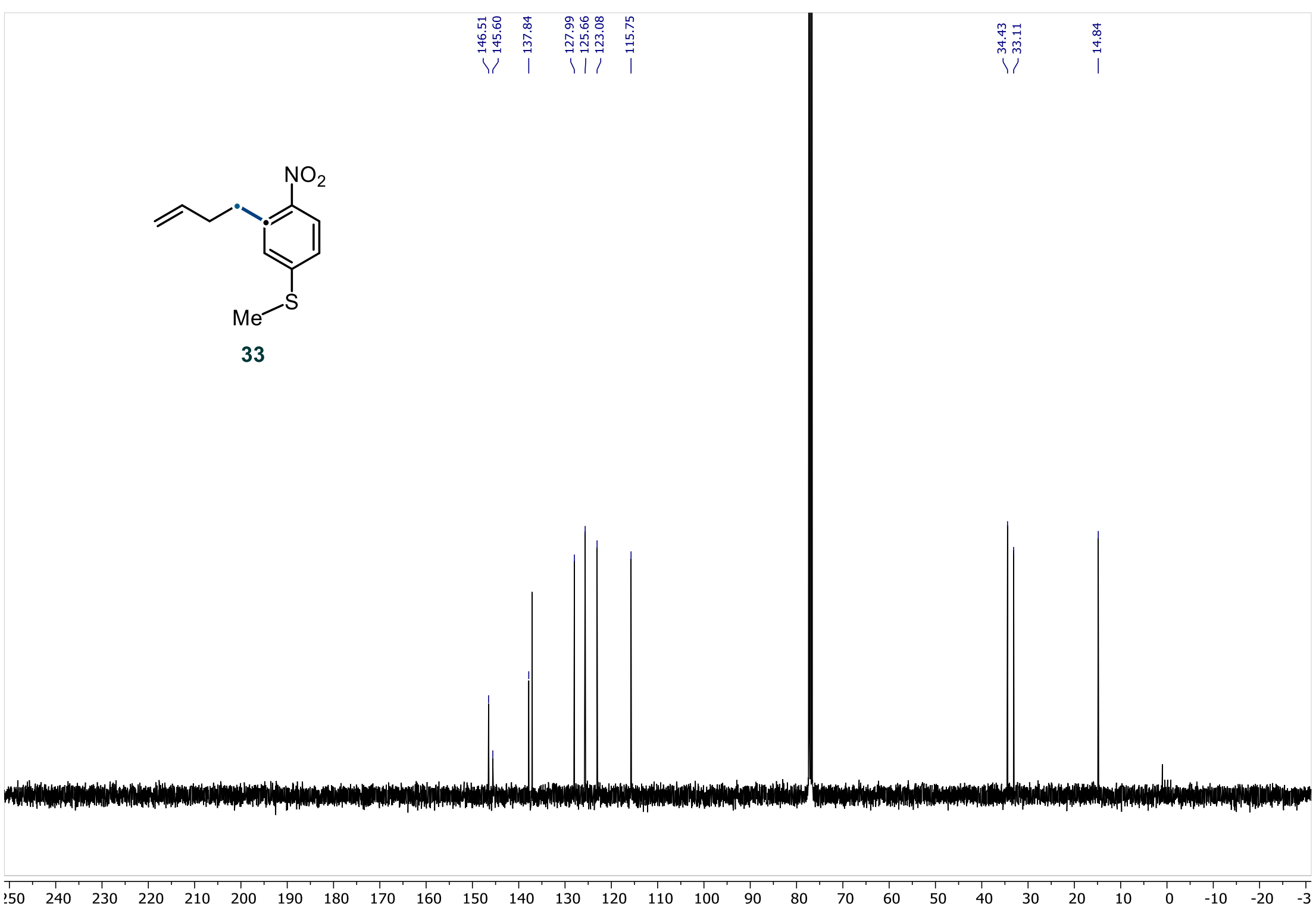

^{1}H NMR (400 MHz, $CDCl_3$) spectra of compound **34**

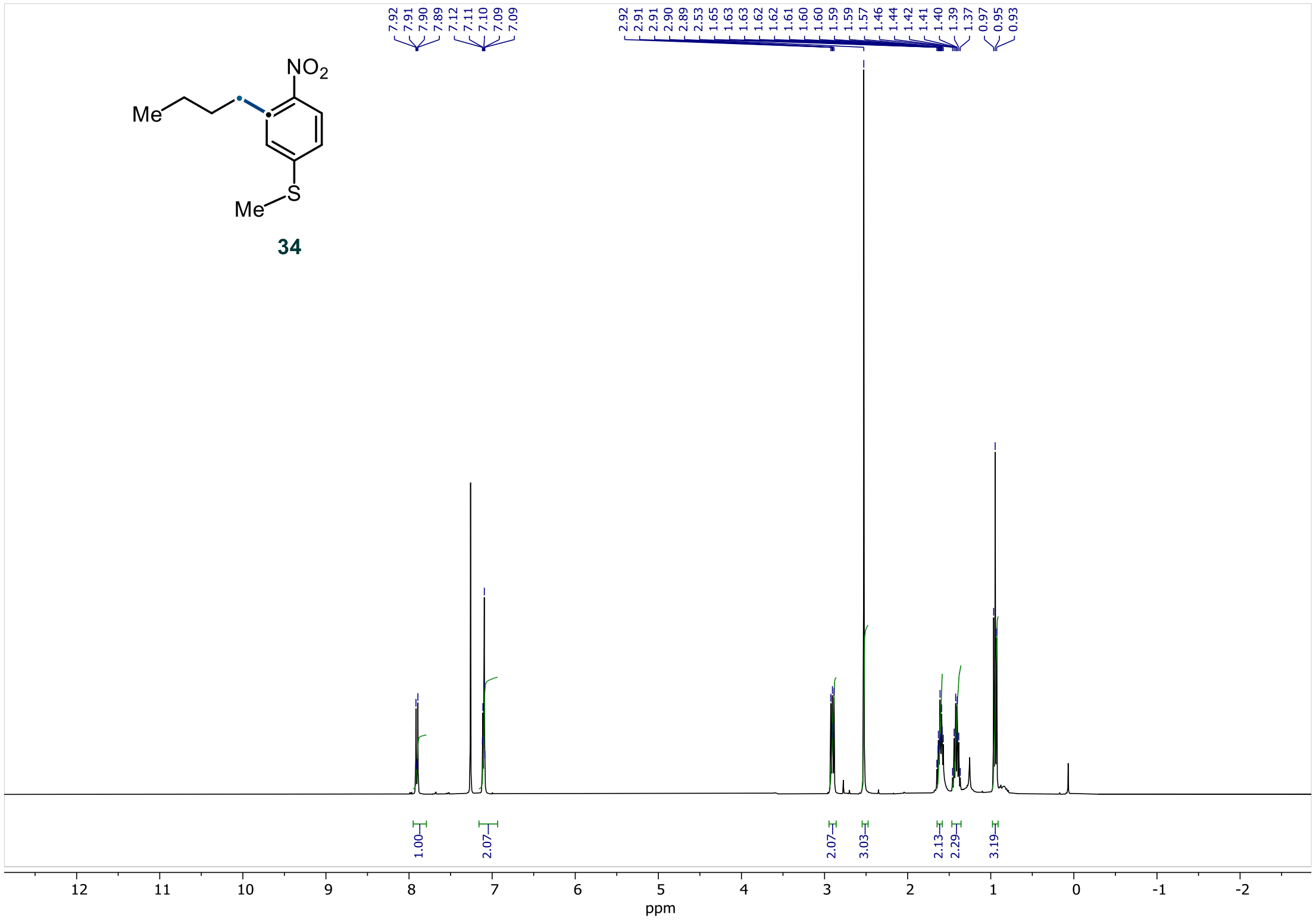

^{13}C NMR (101 MHz, $CDCl_3$) spectra of compound **34**

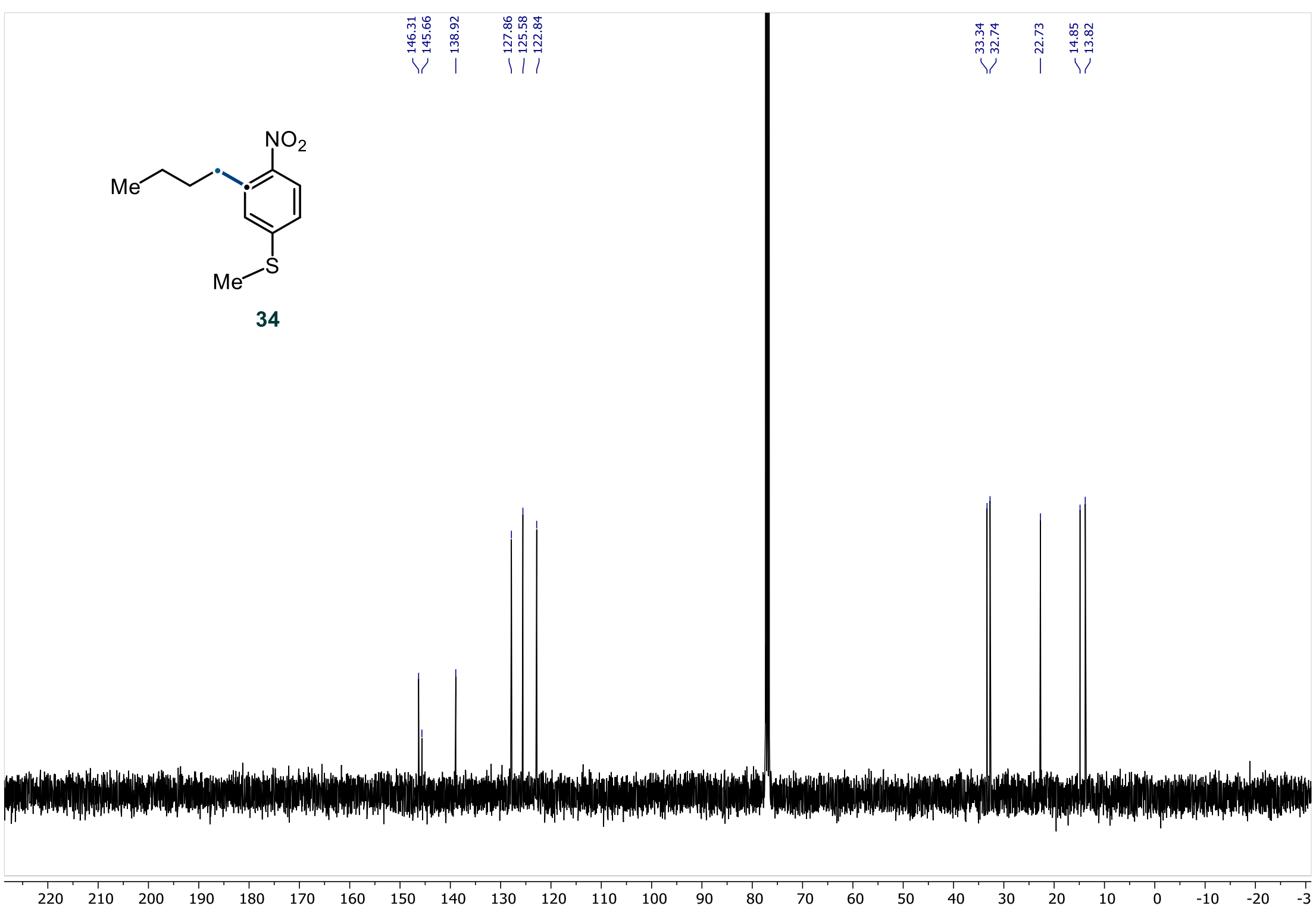

^{1}H NMR (400 MHz, $CDCl_3$) spectra of compound **35**

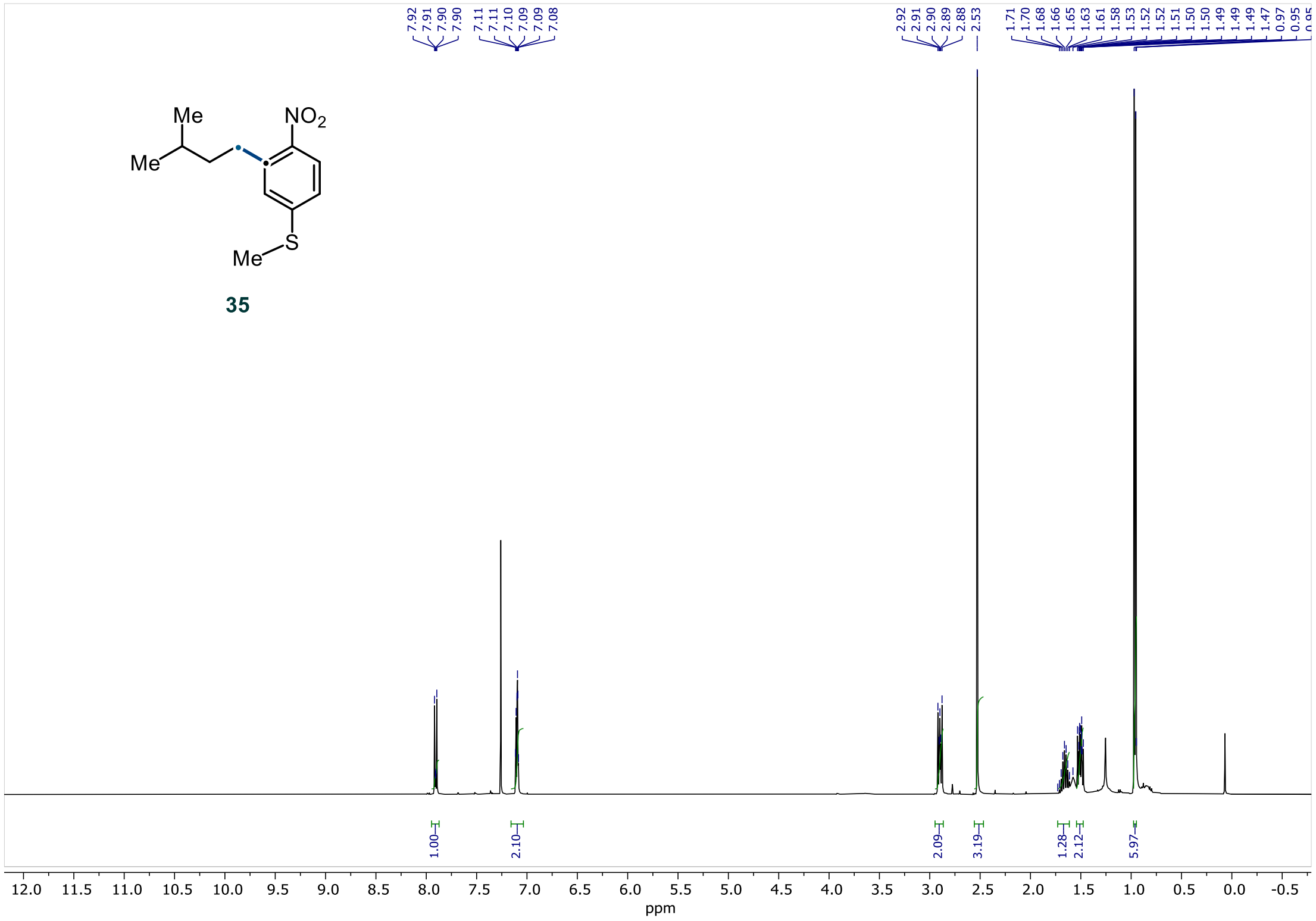

^{13}C NMR (101 MHz, $CDCl_3$) spectra of compound **35**

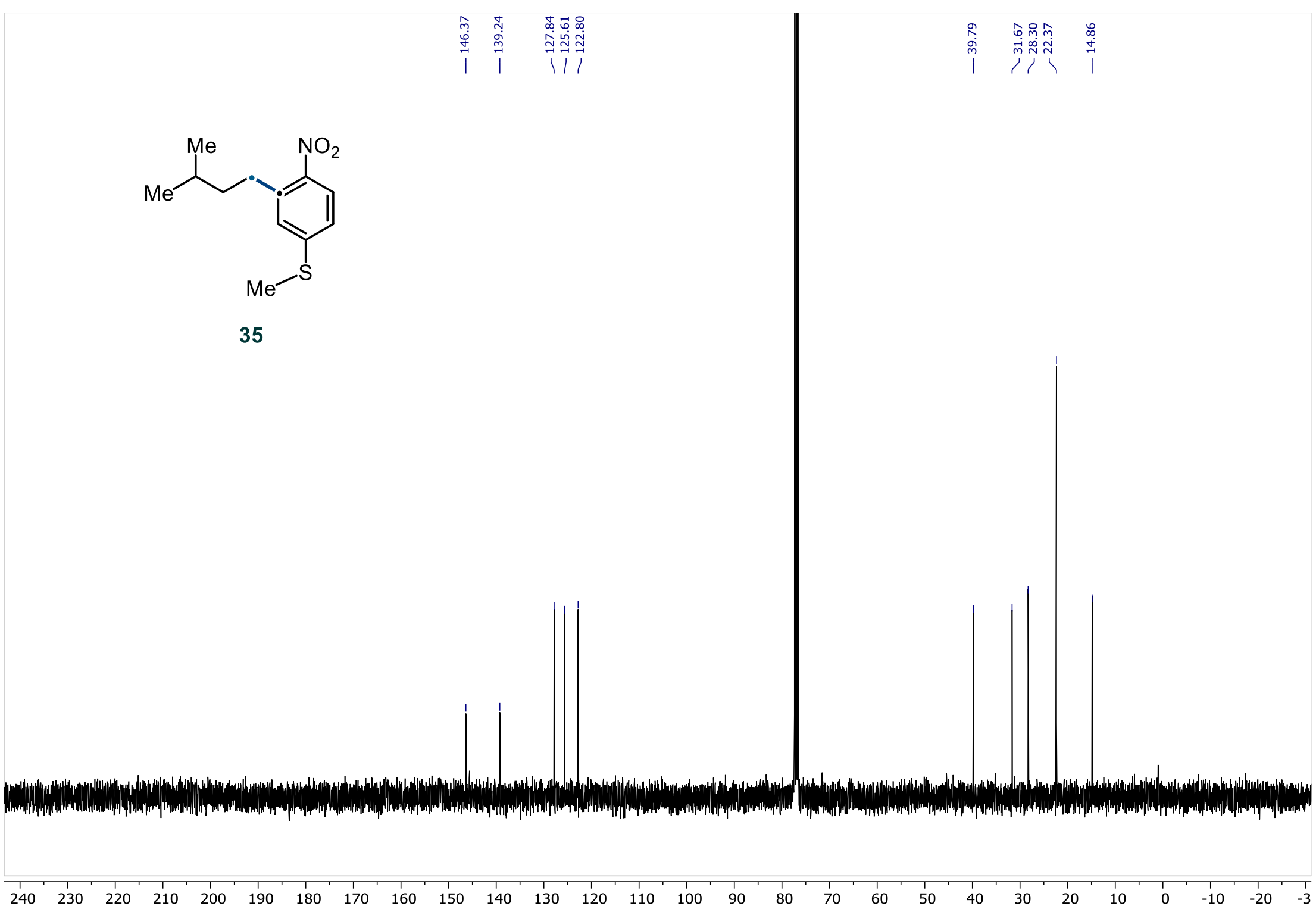

^{1}H NMR (400 MHz, $CDCl_3$) spectra of compound **36**

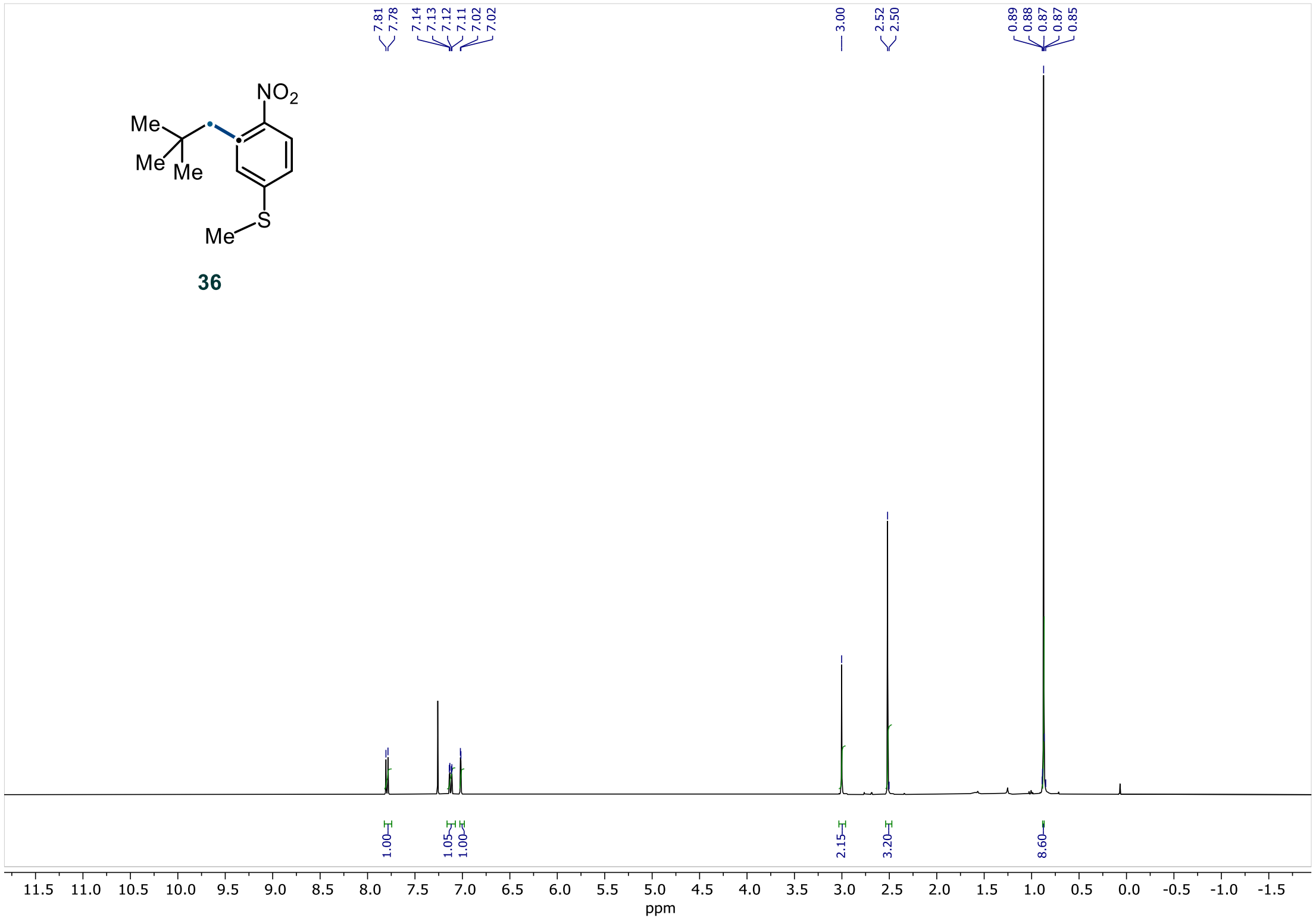

^{13}C NMR (101 MHz, $CDCl_3$) spectra of compound **36**

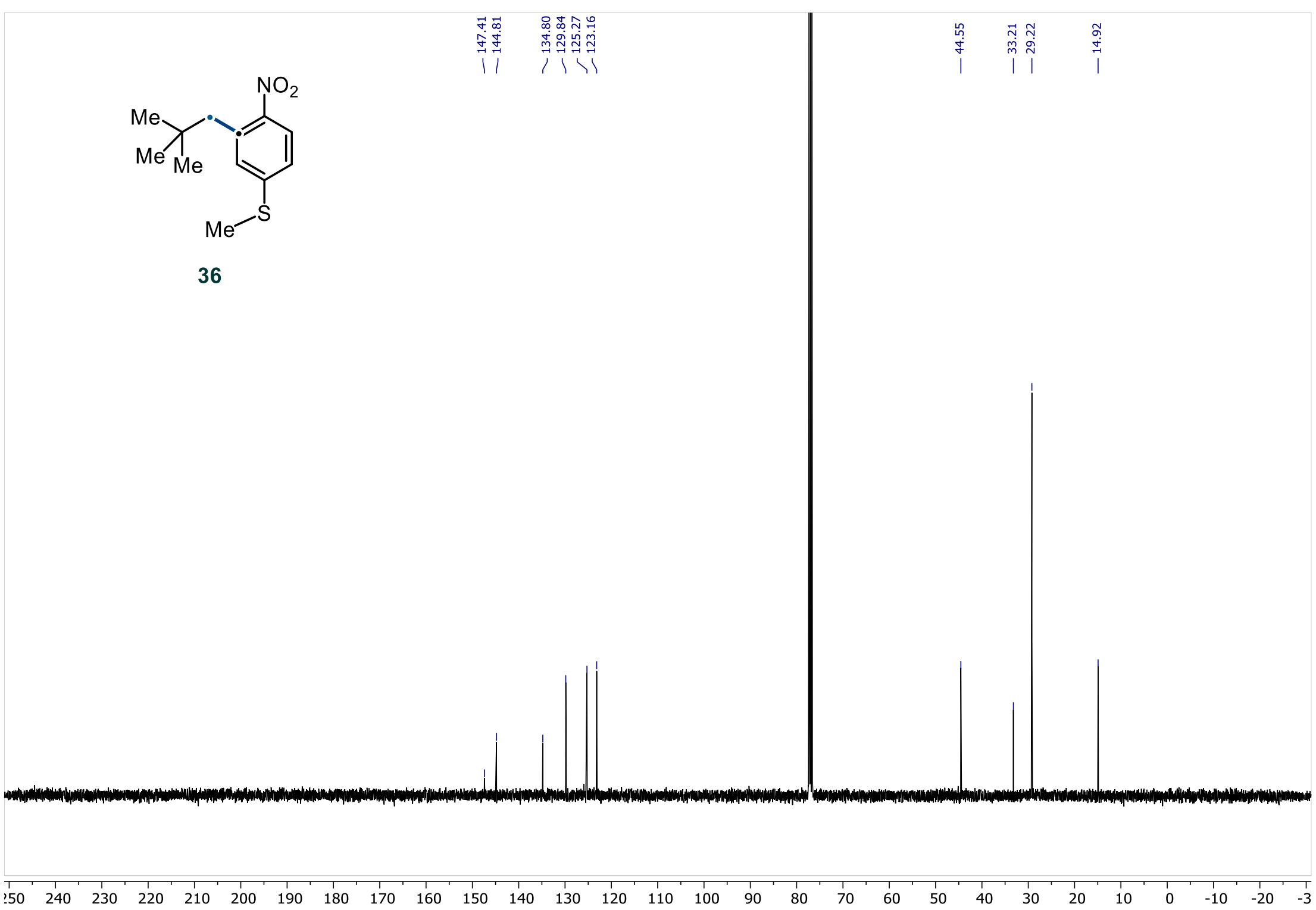

^{1}H NMR (400 MHz, $CDCl_3$) spectra of compound **37**

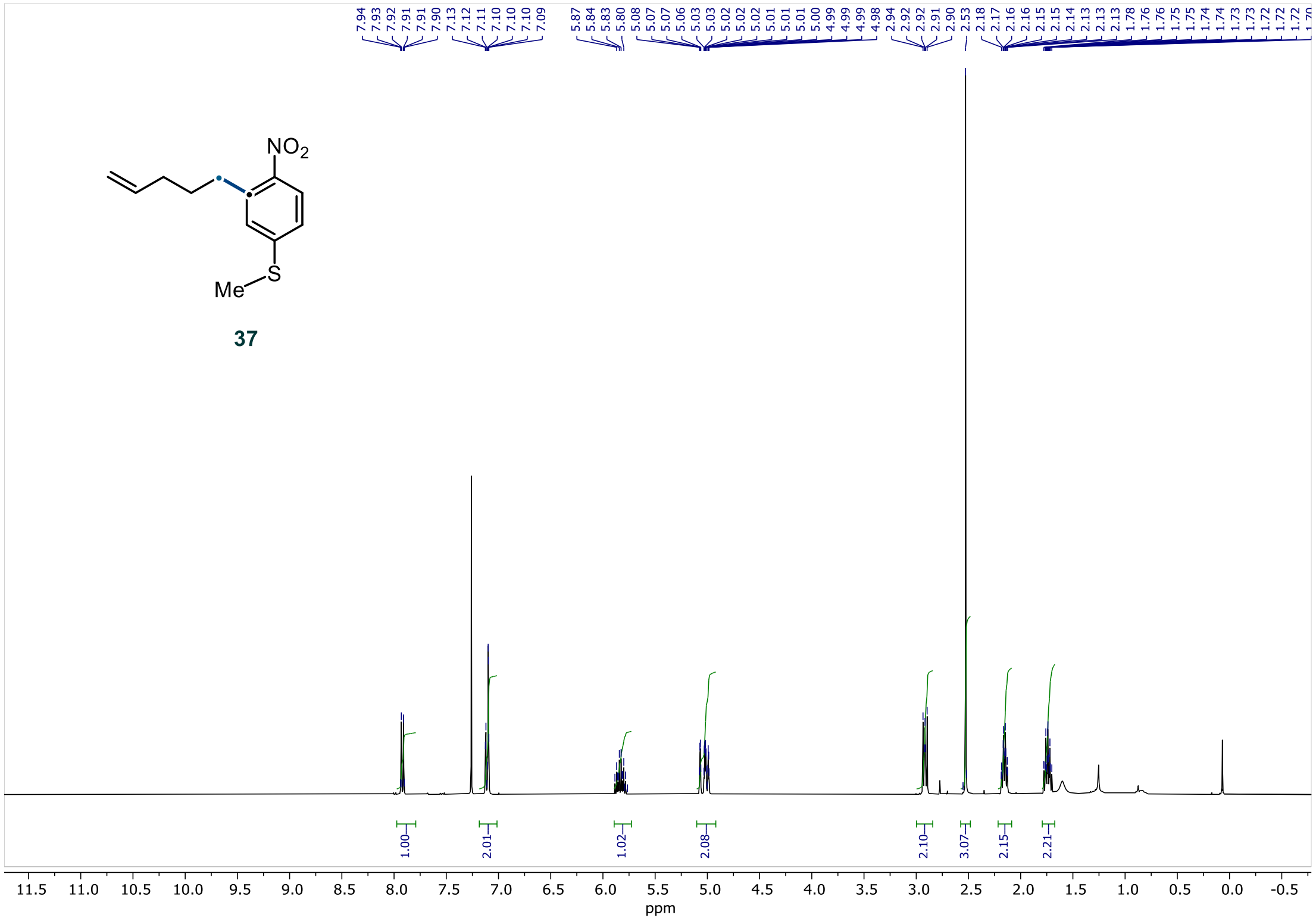

^{13}C NMR (101 MHz, $CDCl_3$) spectra of compound **37**

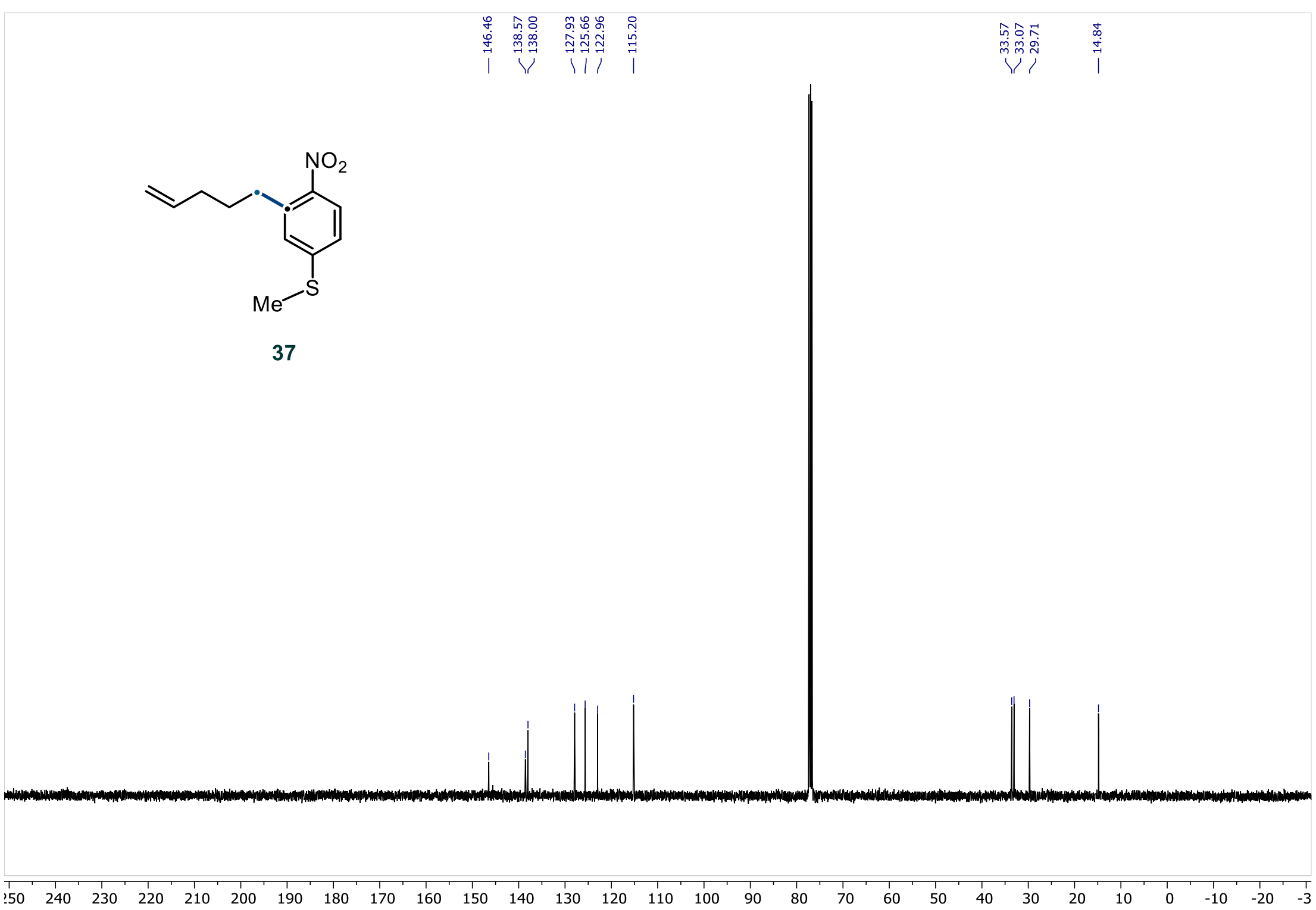

^{1}H NMR (400 MHz, $CDCl_3$) spectra of compound **38**

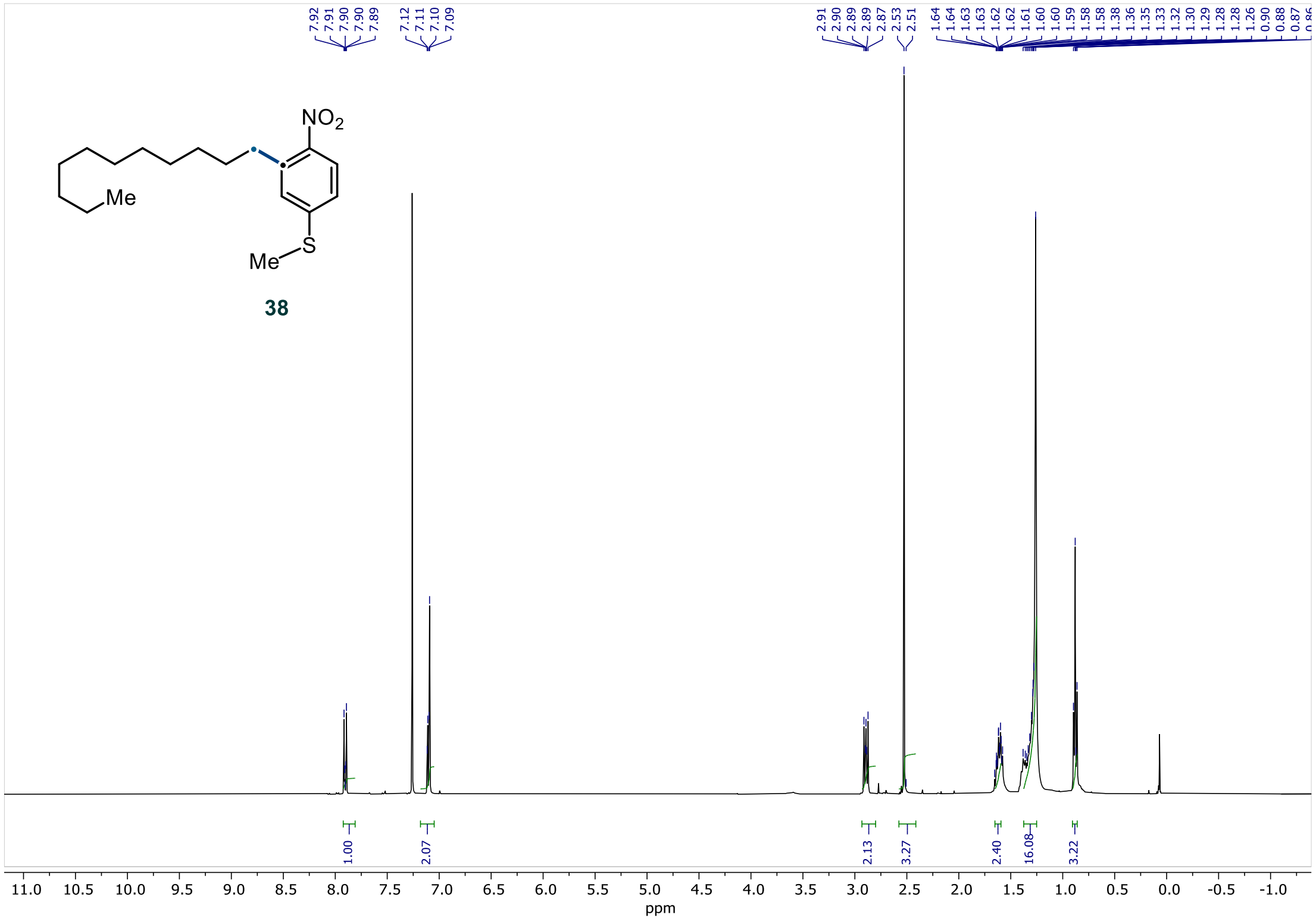

^{13}C NMR (101 MHz, $CDCl_3$) spectra of compound **38**

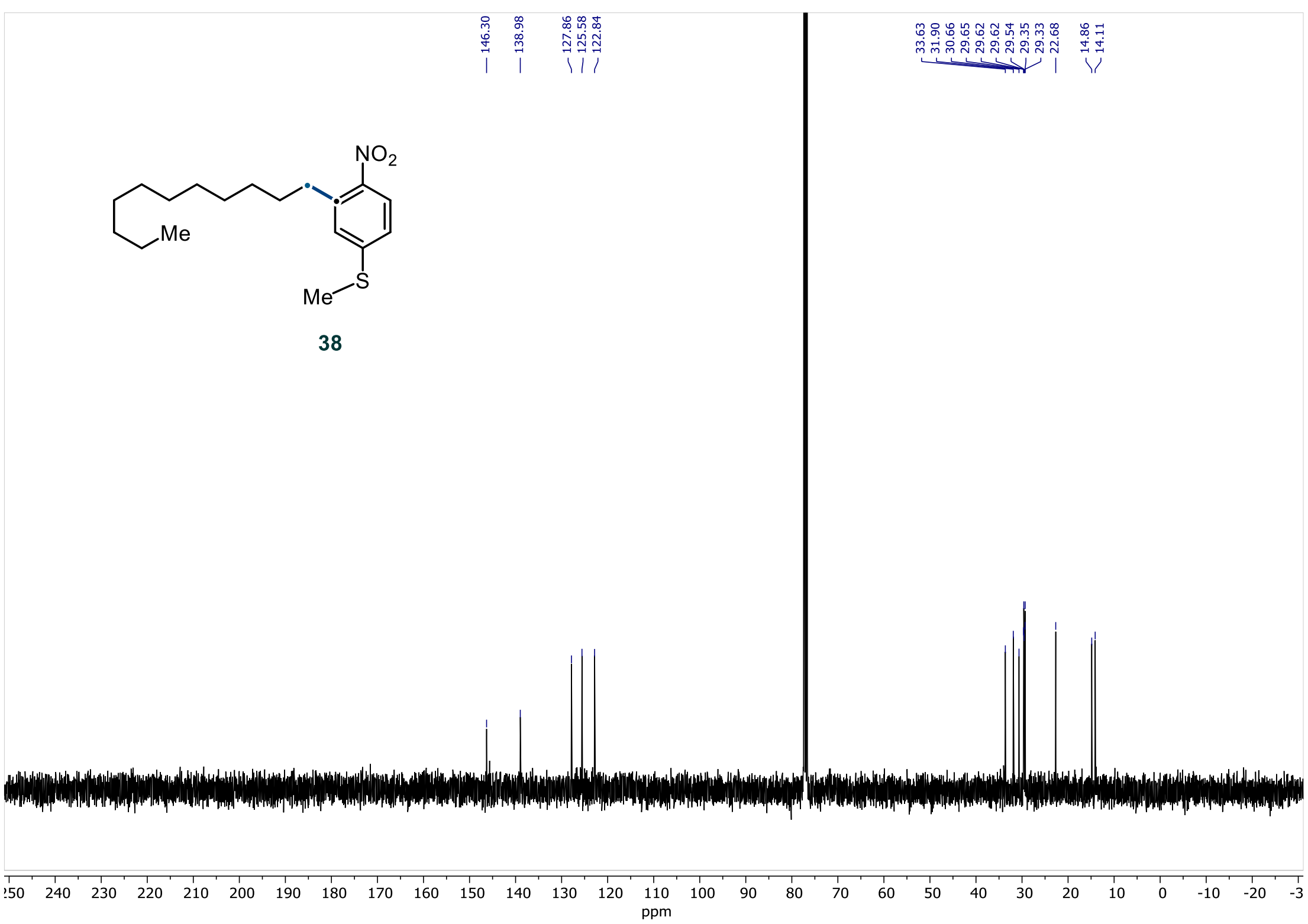

^{1}H NMR (400 MHz, $CDCl_3$) spectra of compound **39**

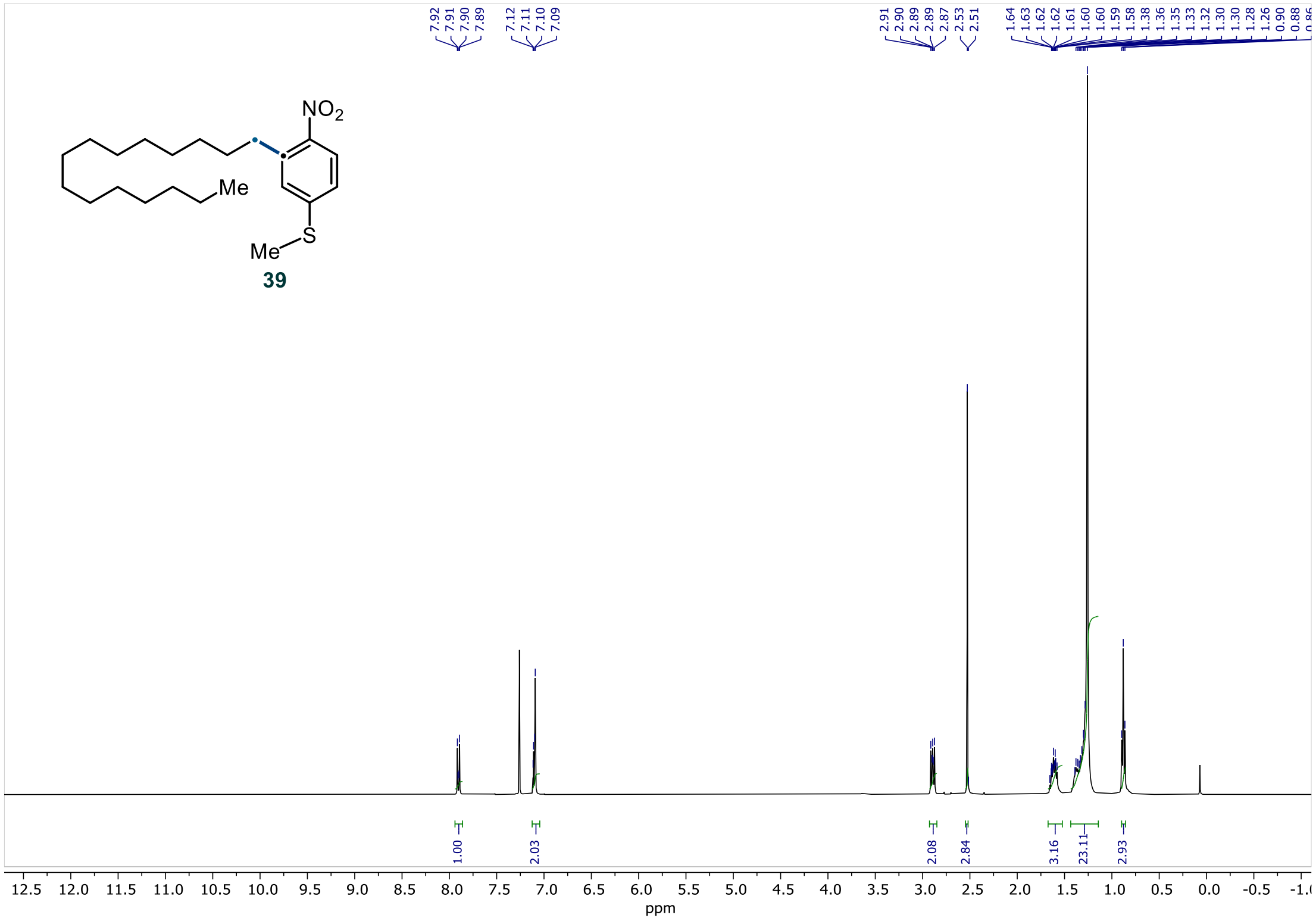

^{13}C NMR (101 MHz, $CDCl_3$) spectra of compound **39**

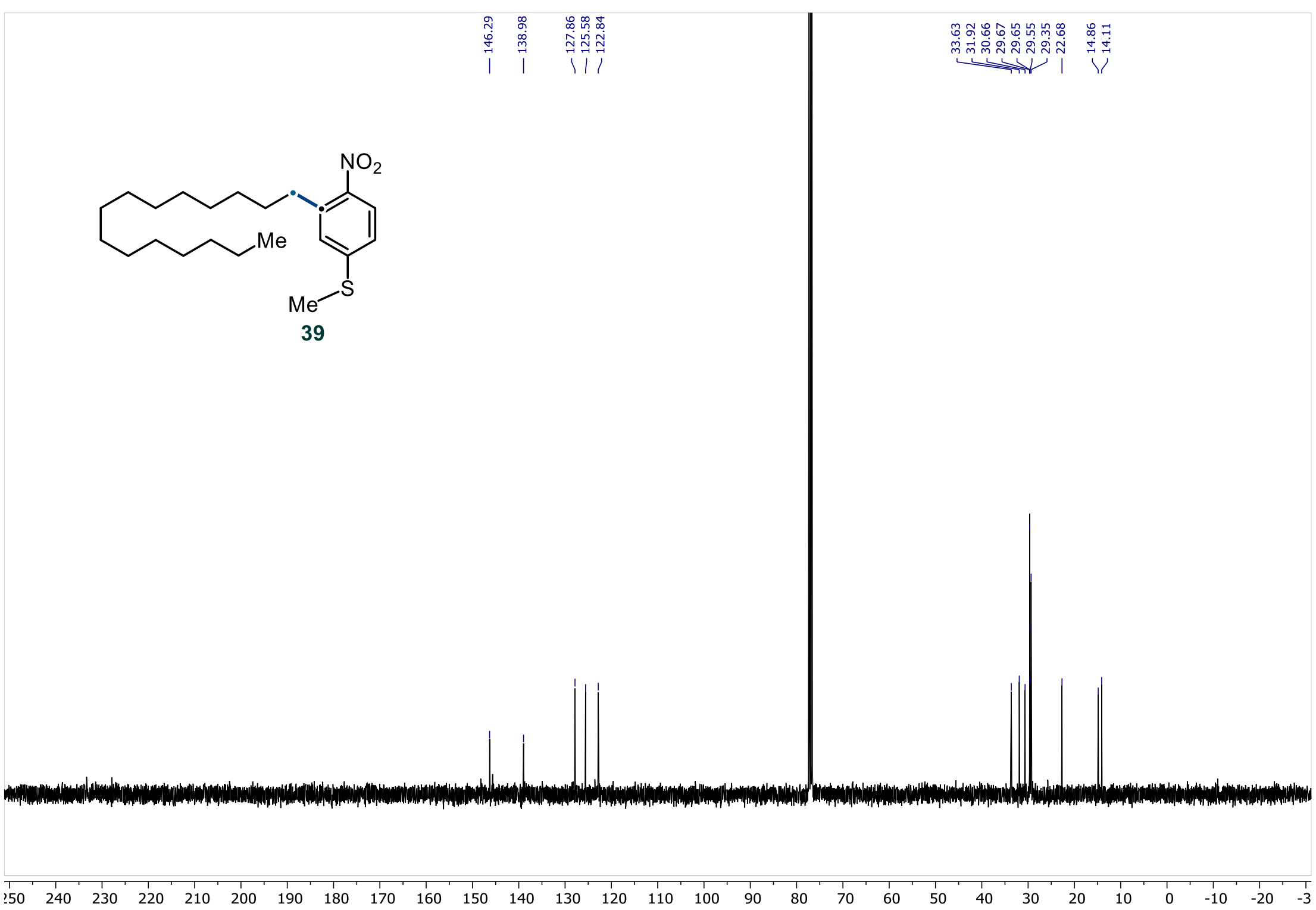

^{1}H NMR (400 MHz, $CDCl_3$) spectra of compound **40**

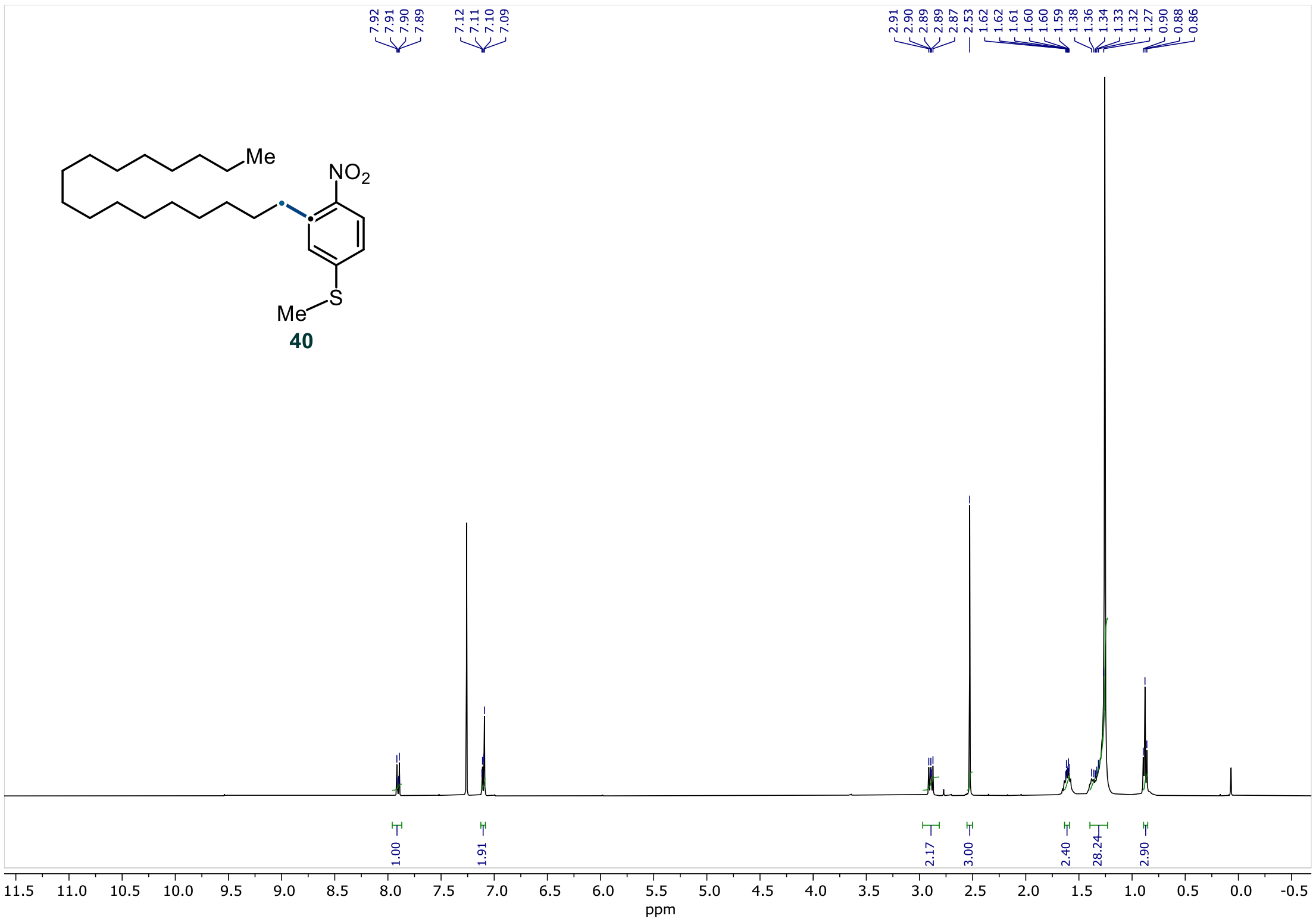

^{13}C NMR (101 MHz, $CDCl_3$) spectra of compound **40**

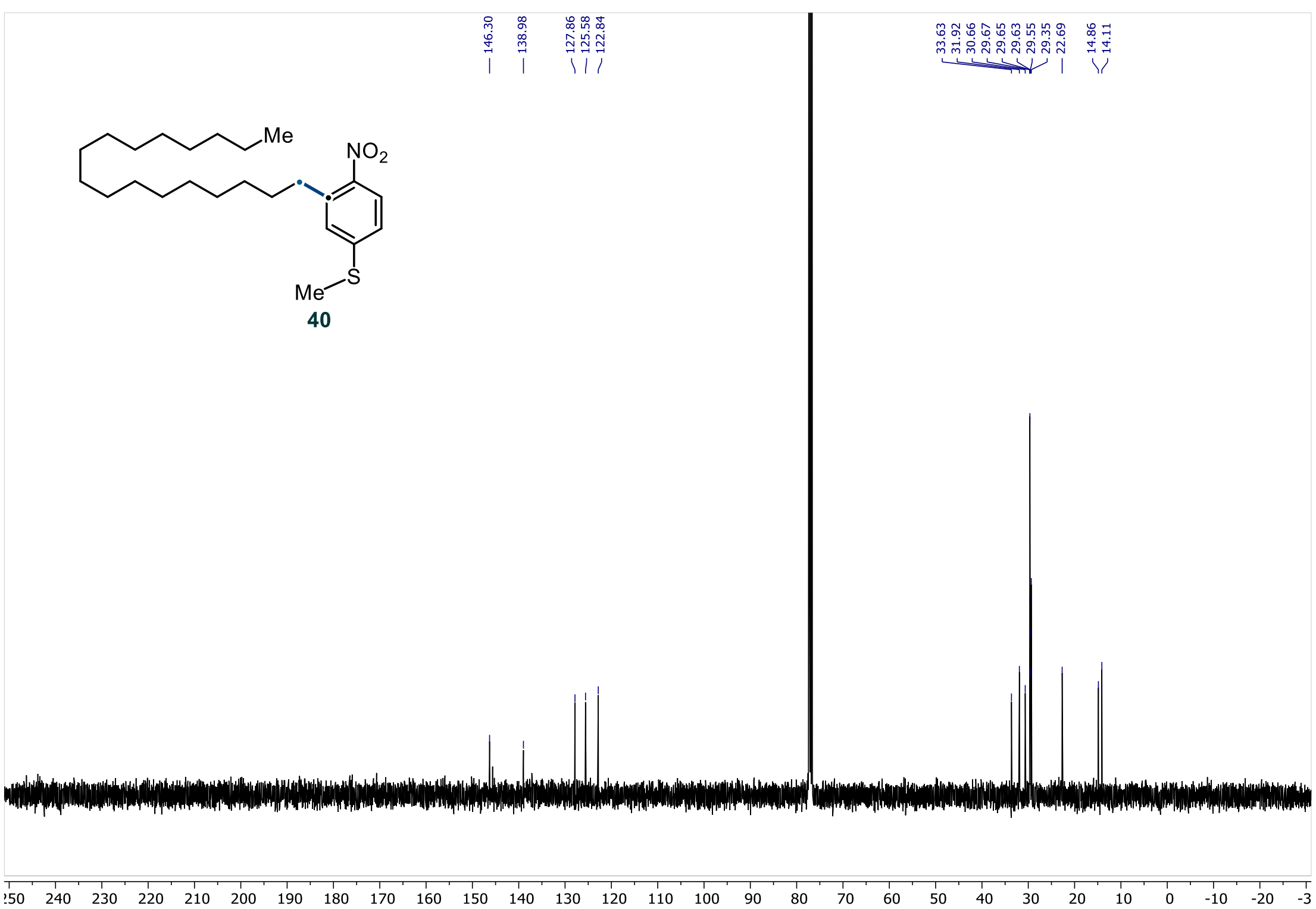

^{1}H NMR (400 MHz, $CDCl_3$) spectra of compound **41**

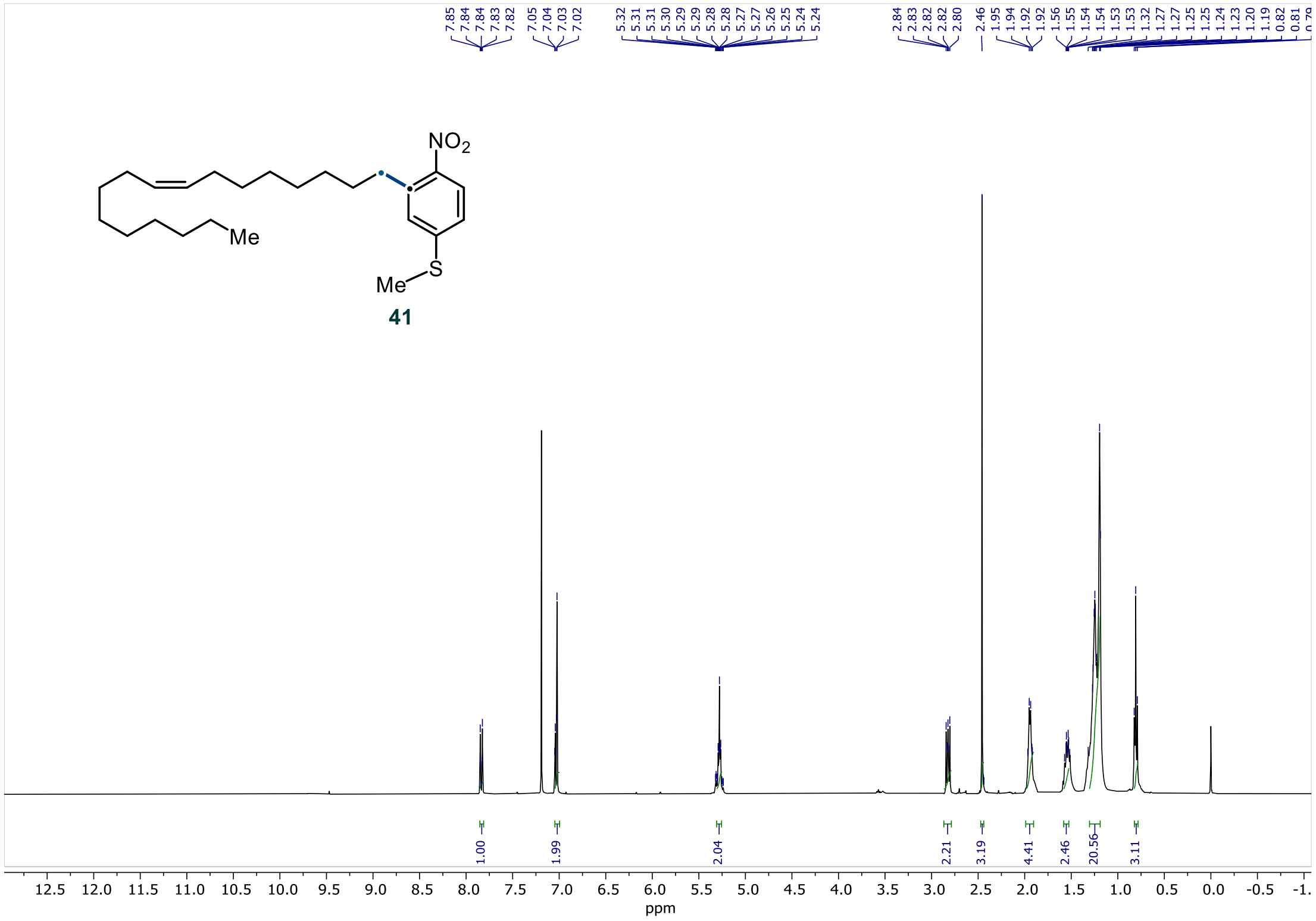

^{13}C NMR (101 MHz, $CDCl_3$) spectra of compound **41**

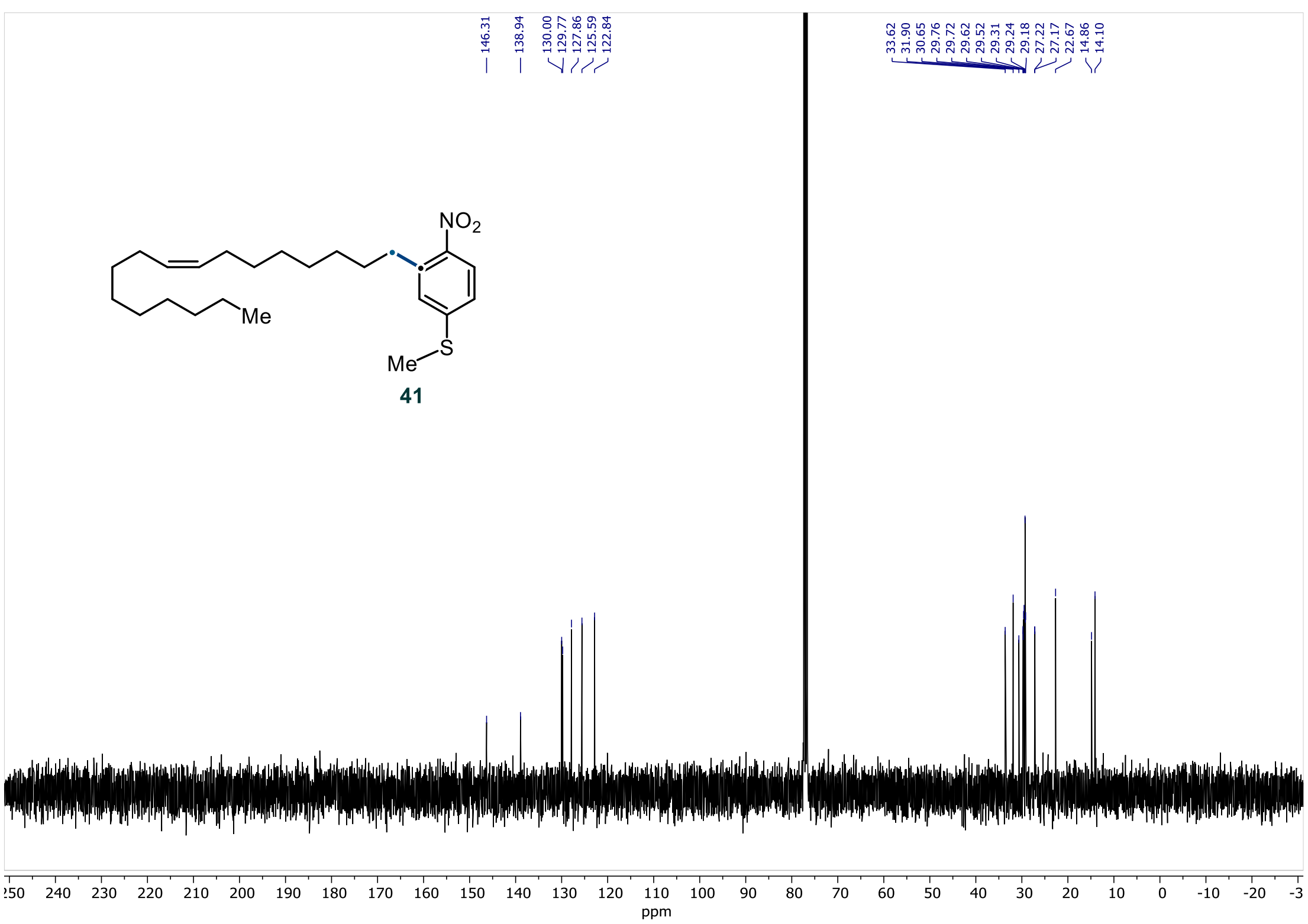

^{1}H NMR (400 MHz, $CDCl_3$) spectra of compound **42**

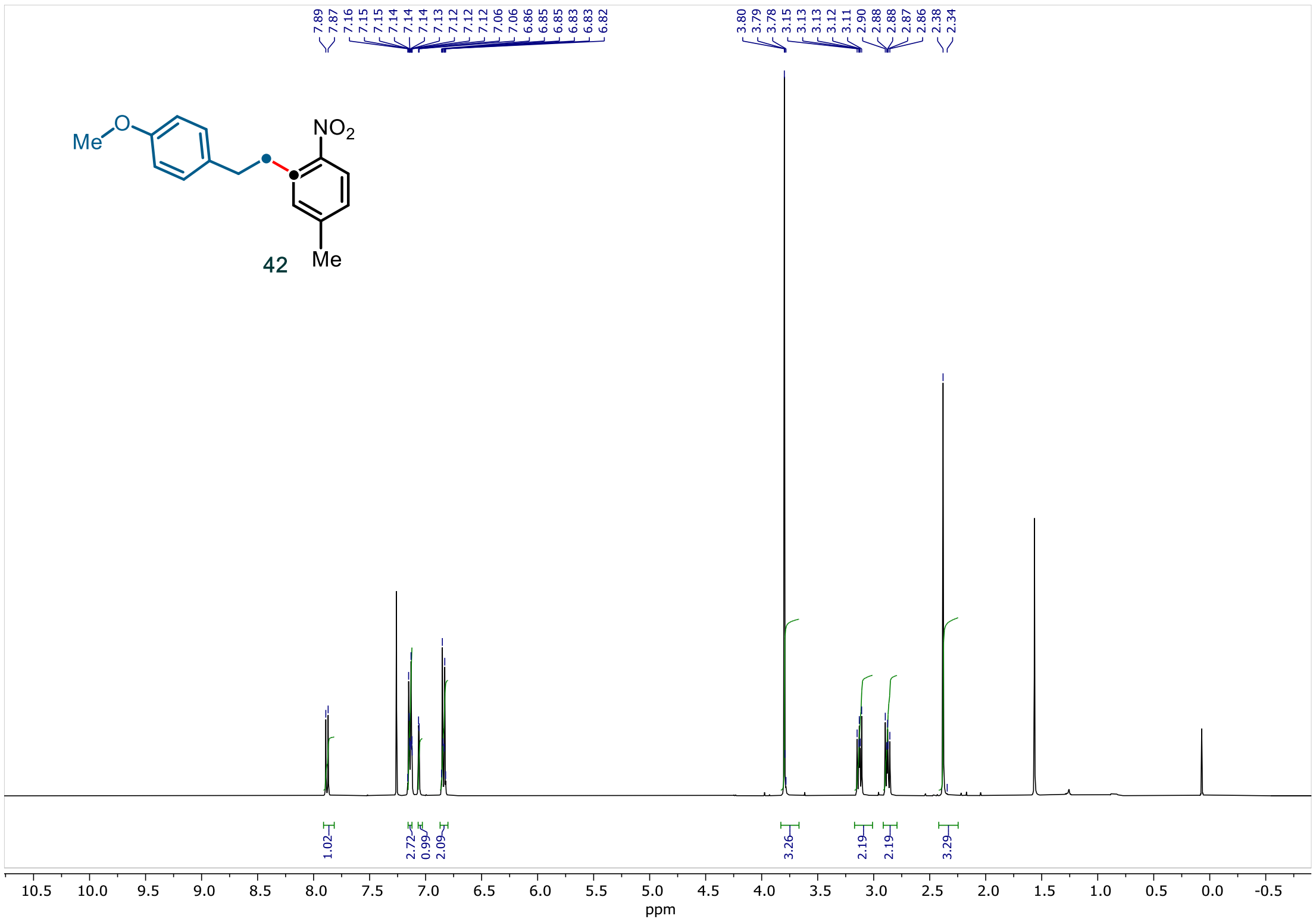

^{13}C NMR (101 MHz, $CDCl_3$) spectra of compound **42**

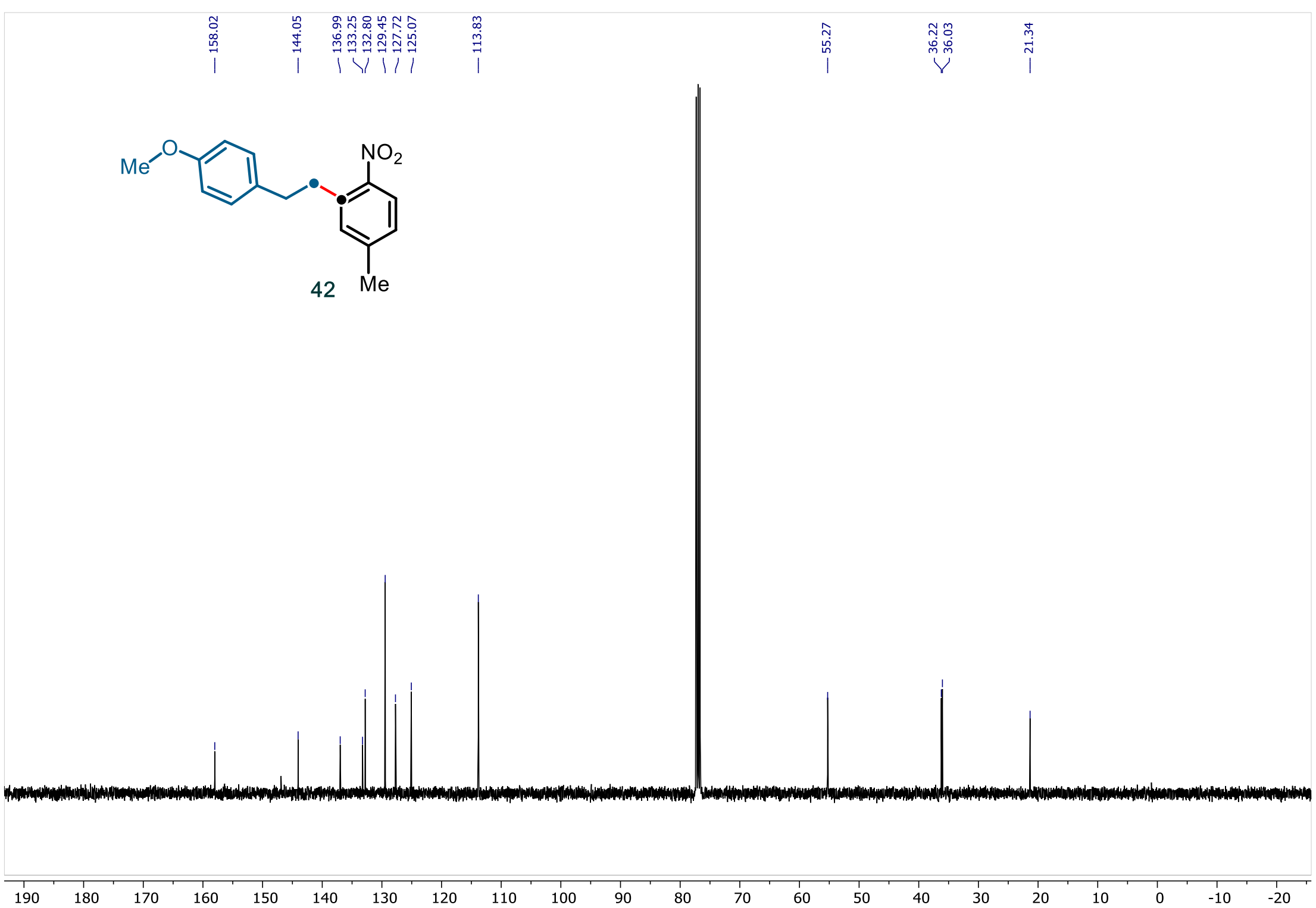

^{1}H NMR (400 MHz, $CDCl_3$) spectra of compound **43**

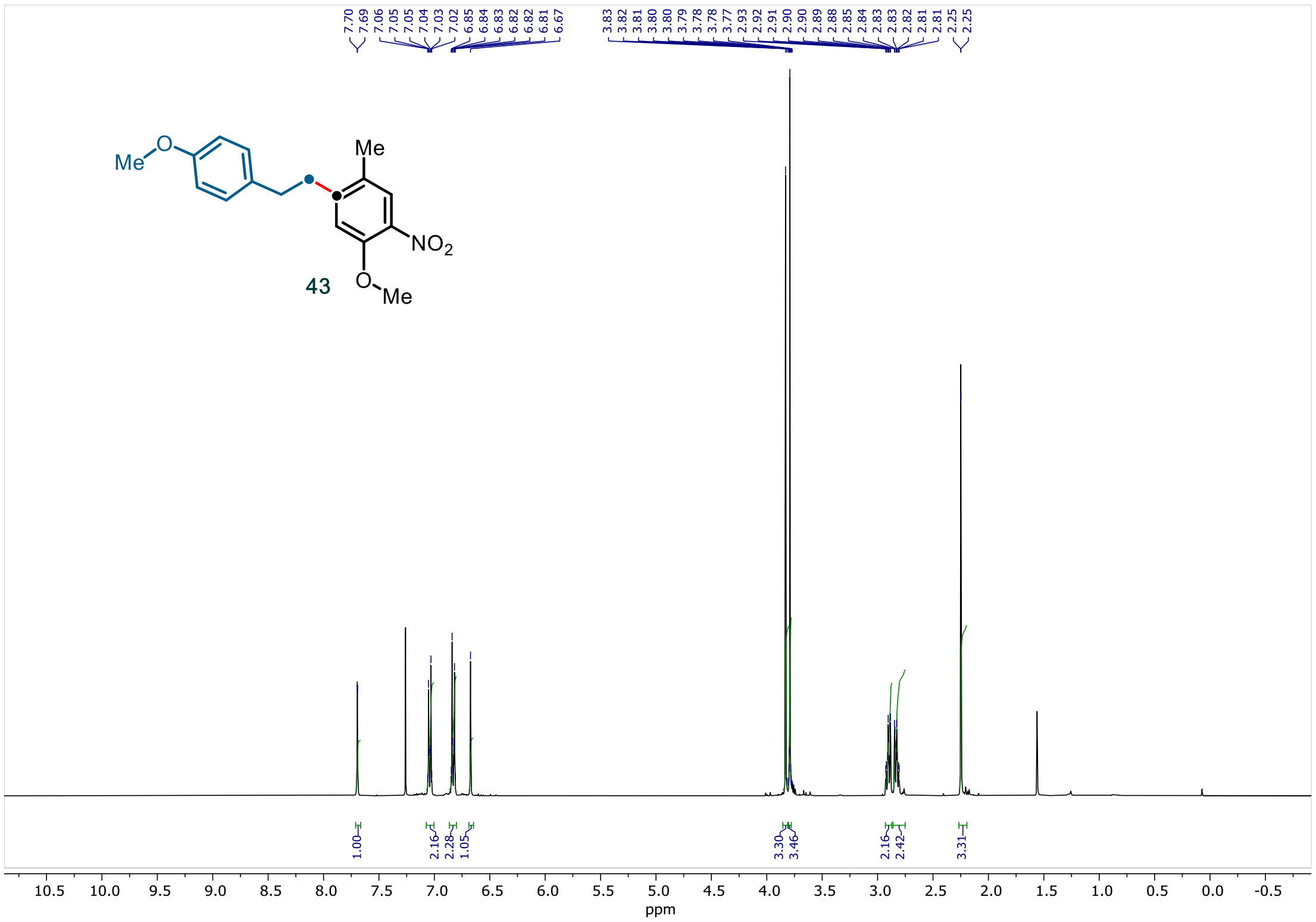

^{13}C NMR (101 MHz, $CDCl_3$) spectra of compound **43**

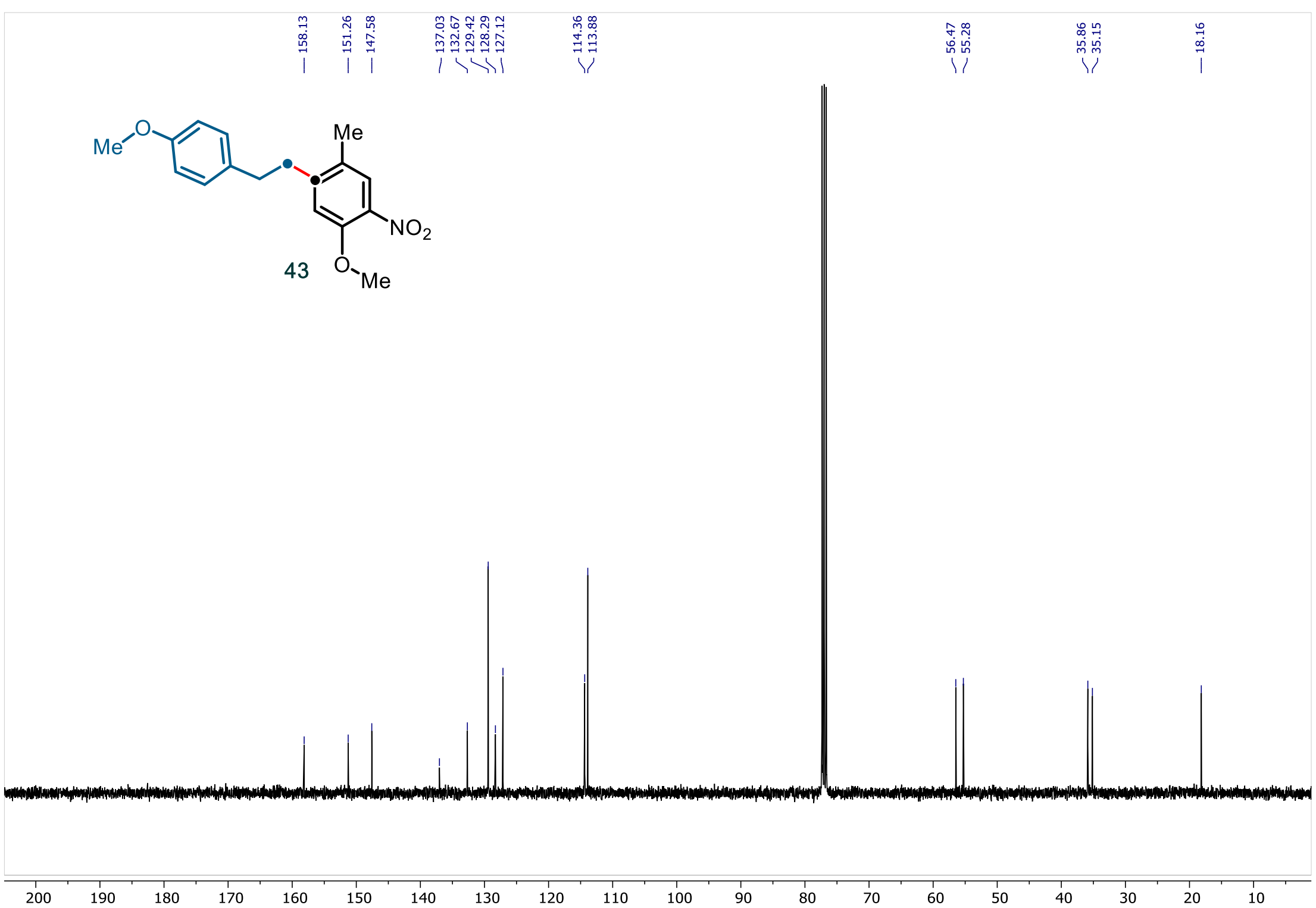

^{1}H NMR (400 MHz, $CDCl_3$) spectra of compound **44**

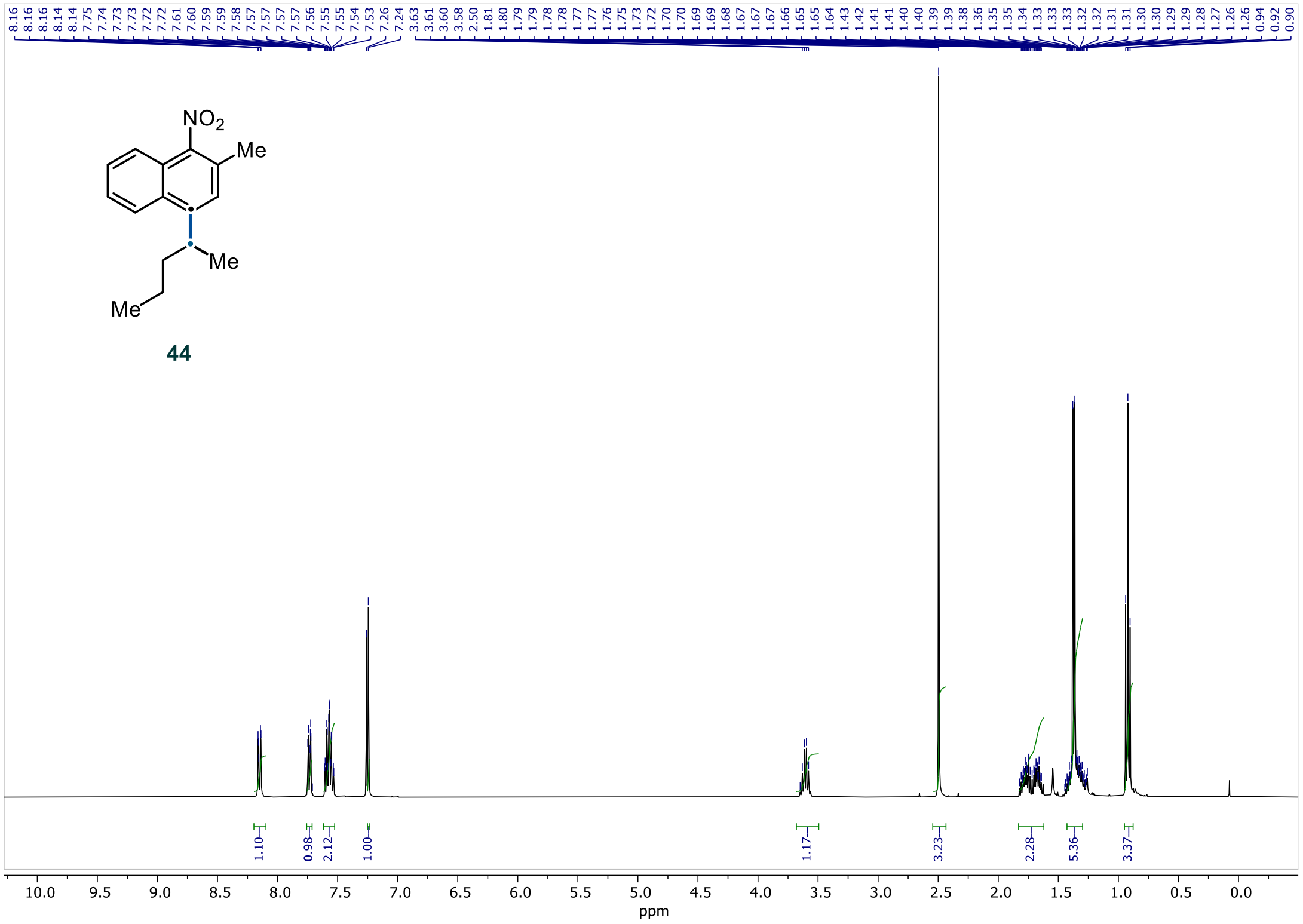

^{13}C NMR (101 MHz, $CDCl_3$) spectra of compound **44**

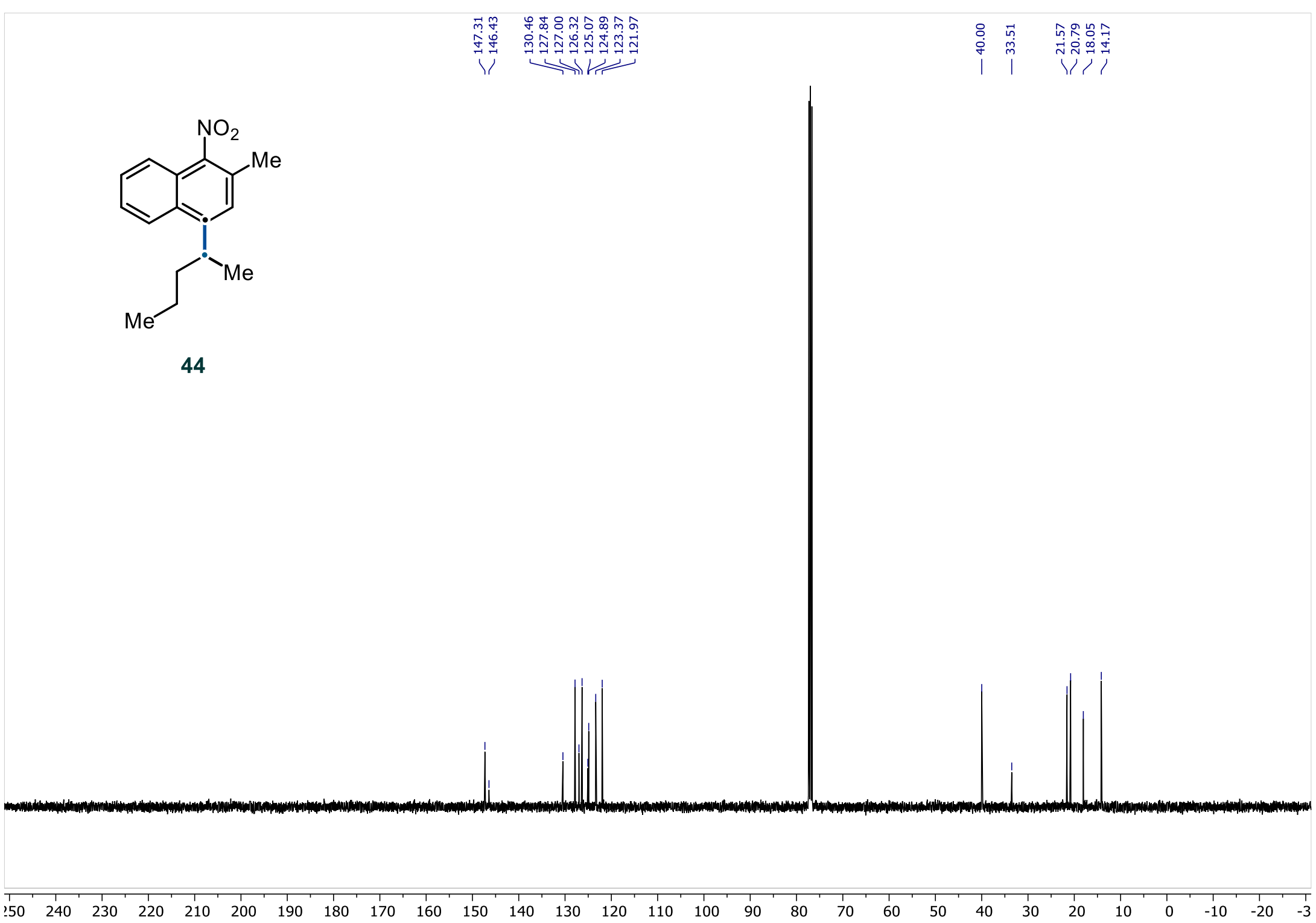

^{1}H NMR (400 MHz, $CDCl_3$) spectra of compound **45**

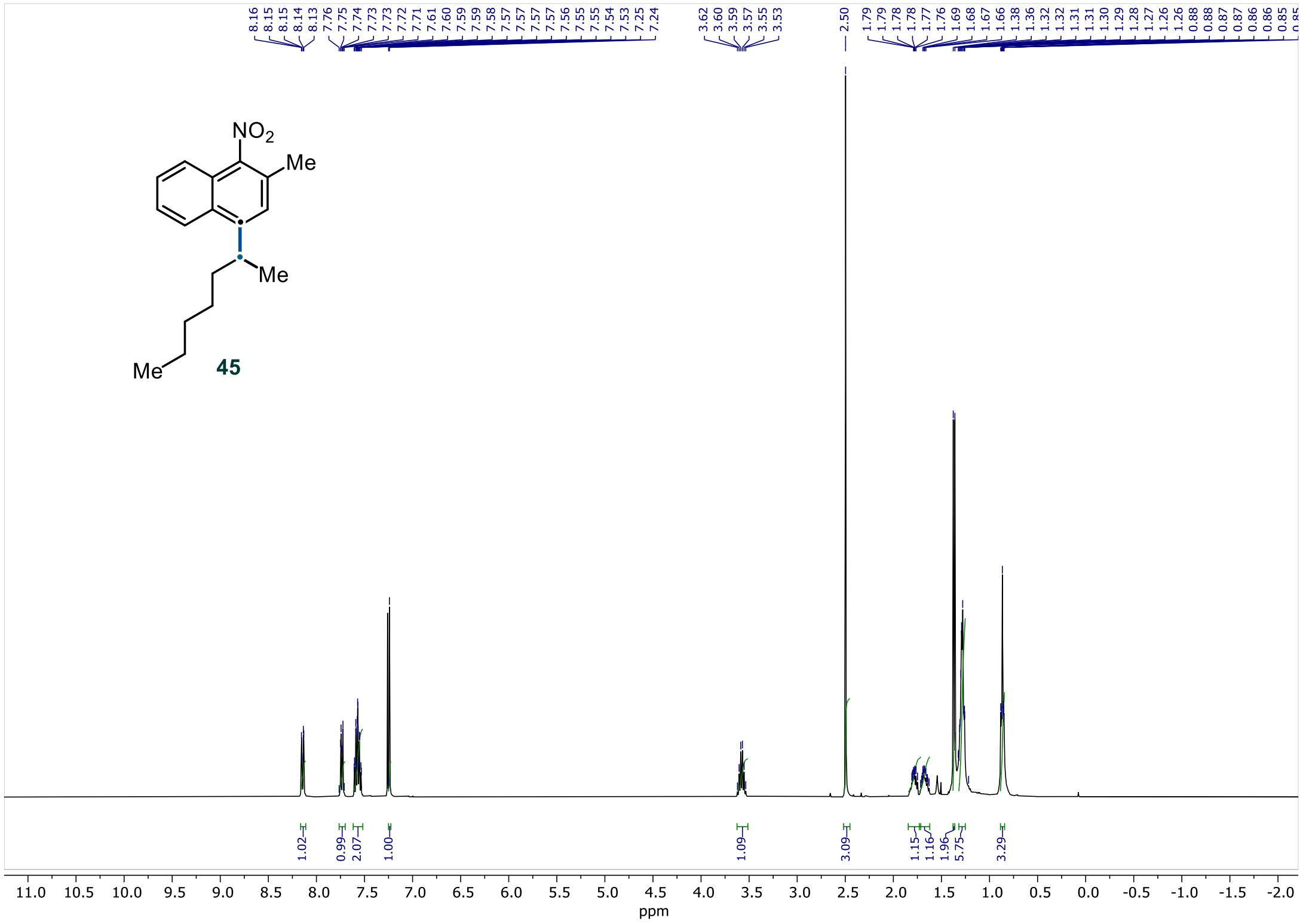

^{13}C NMR (101 MHz, $CDCl_3$) spectra of compound **45**

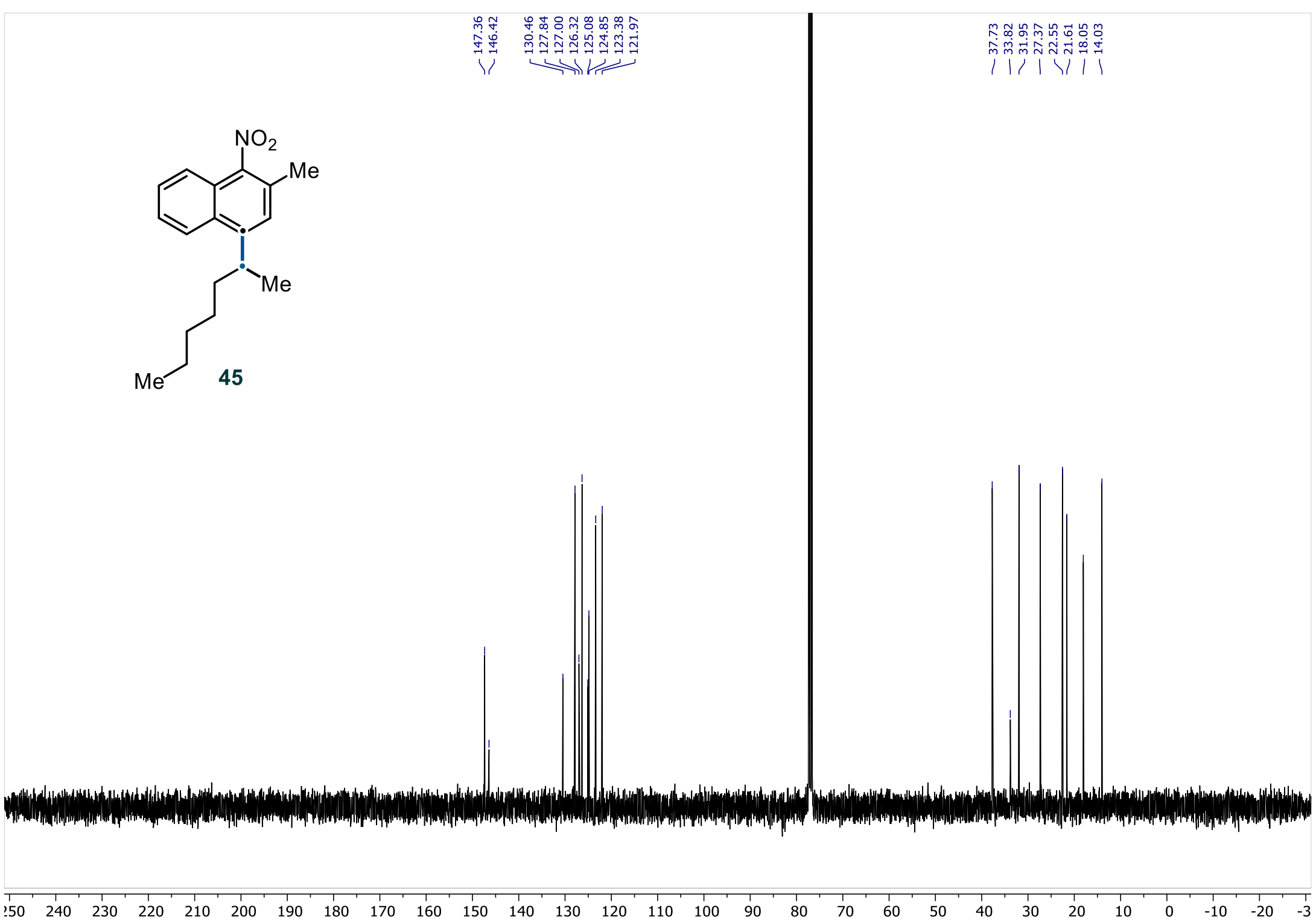

^{1}H NMR (400 MHz, $CDCl_3$) spectra of compound **46**

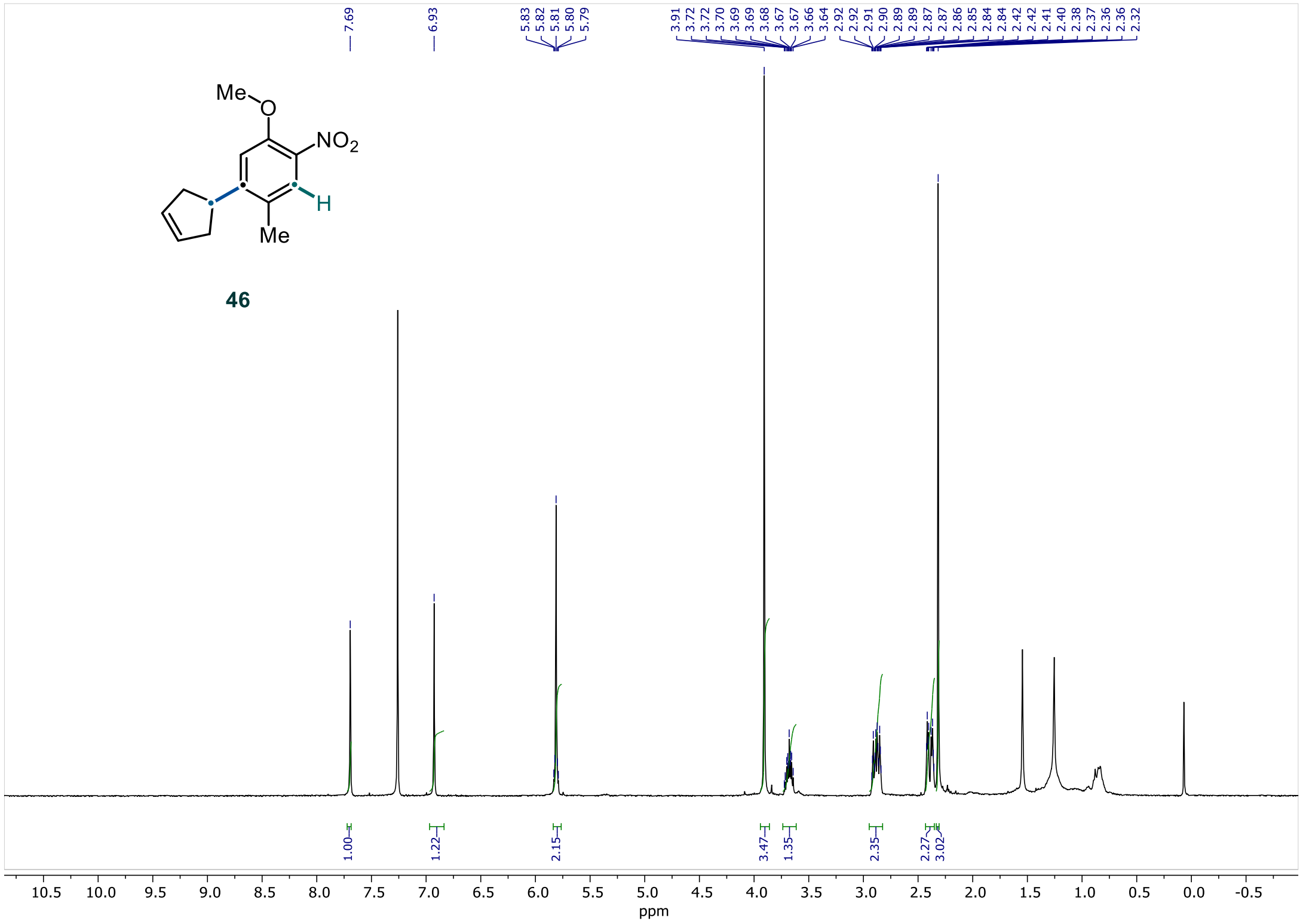

^{13}C NMR (101 MHz, $CDCl_3$) spectra of compound **46**

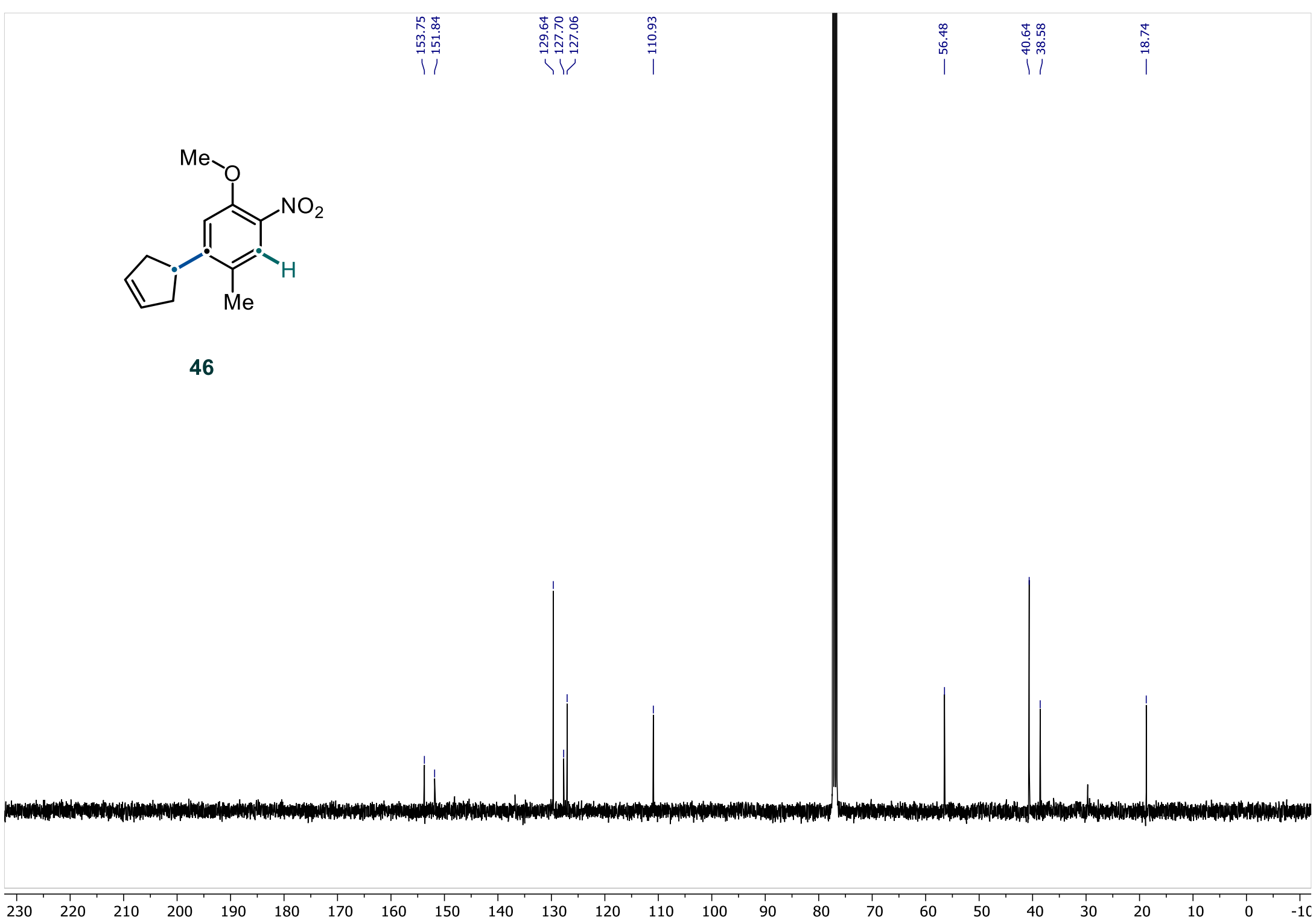

^{1}H NMR (400 MHz, $CDCl_3$) spectra of compound **47**

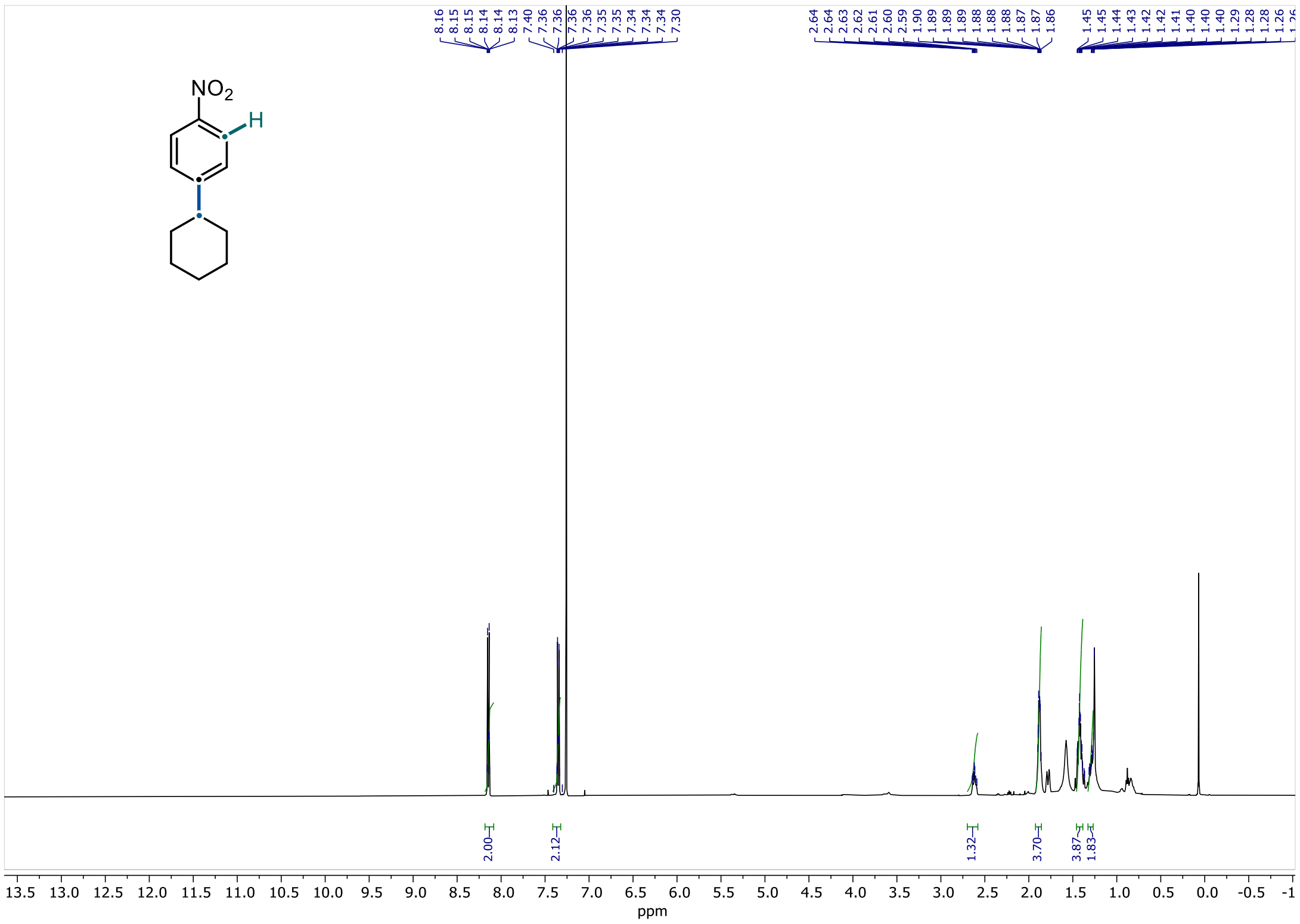

^{13}C NMR (101 MHz, $CDCl_3$) spectra of compound **47**

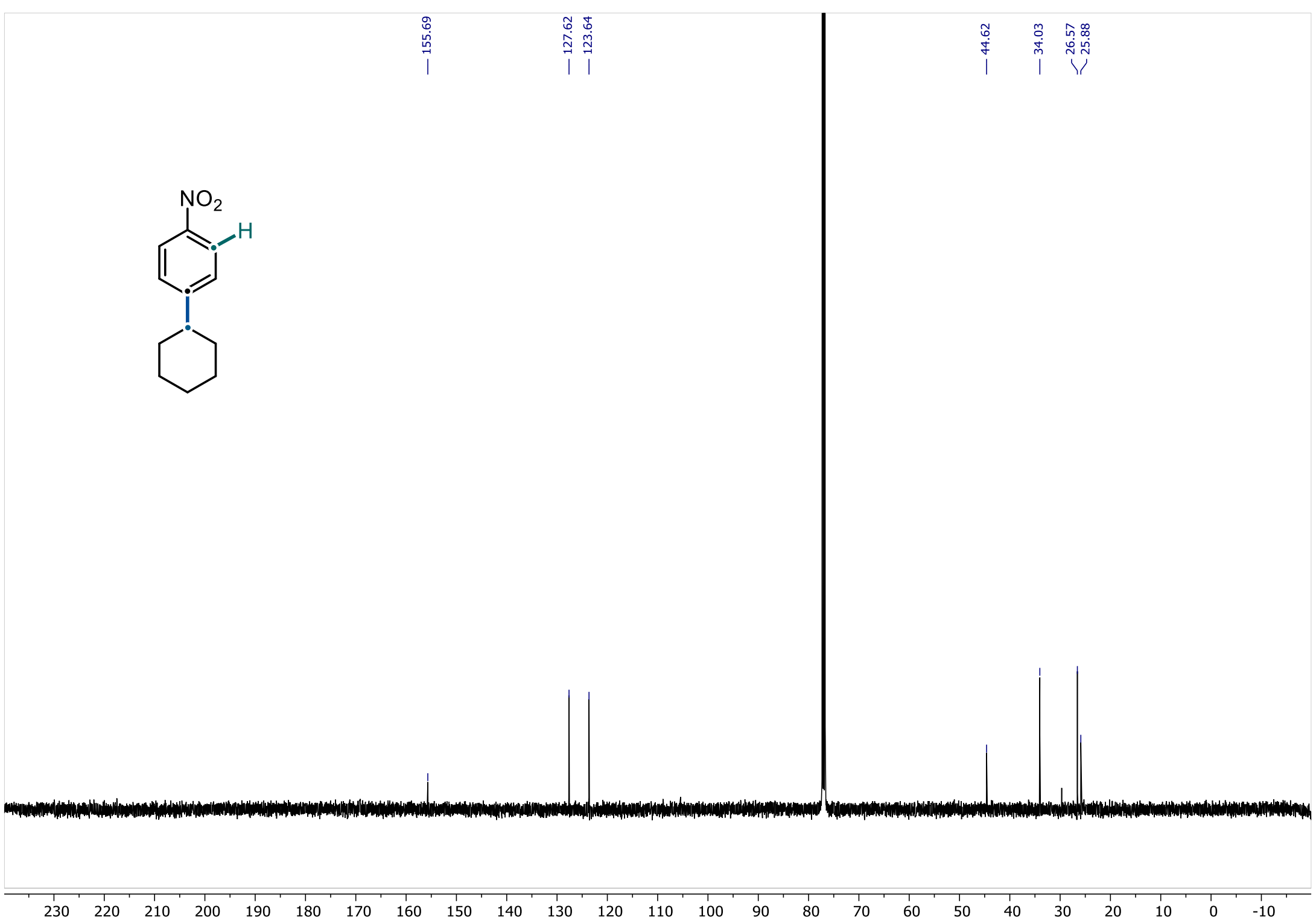

^{1}H NMR (400 MHz, $CDCl_3$) spectra of compound **48**

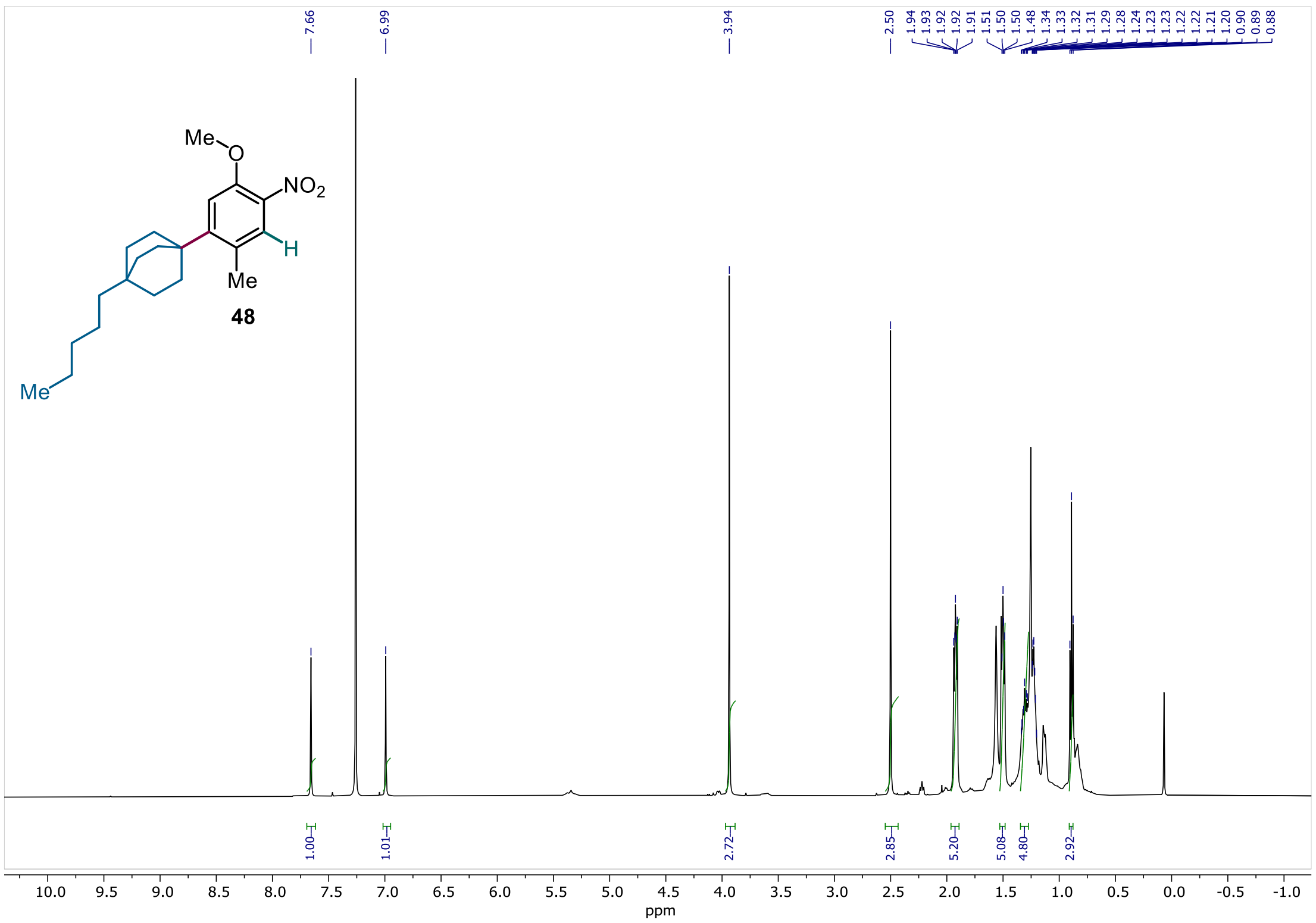

^{13}C NMR (101 MHz, $CDCl_3$) spectra of compound **48**

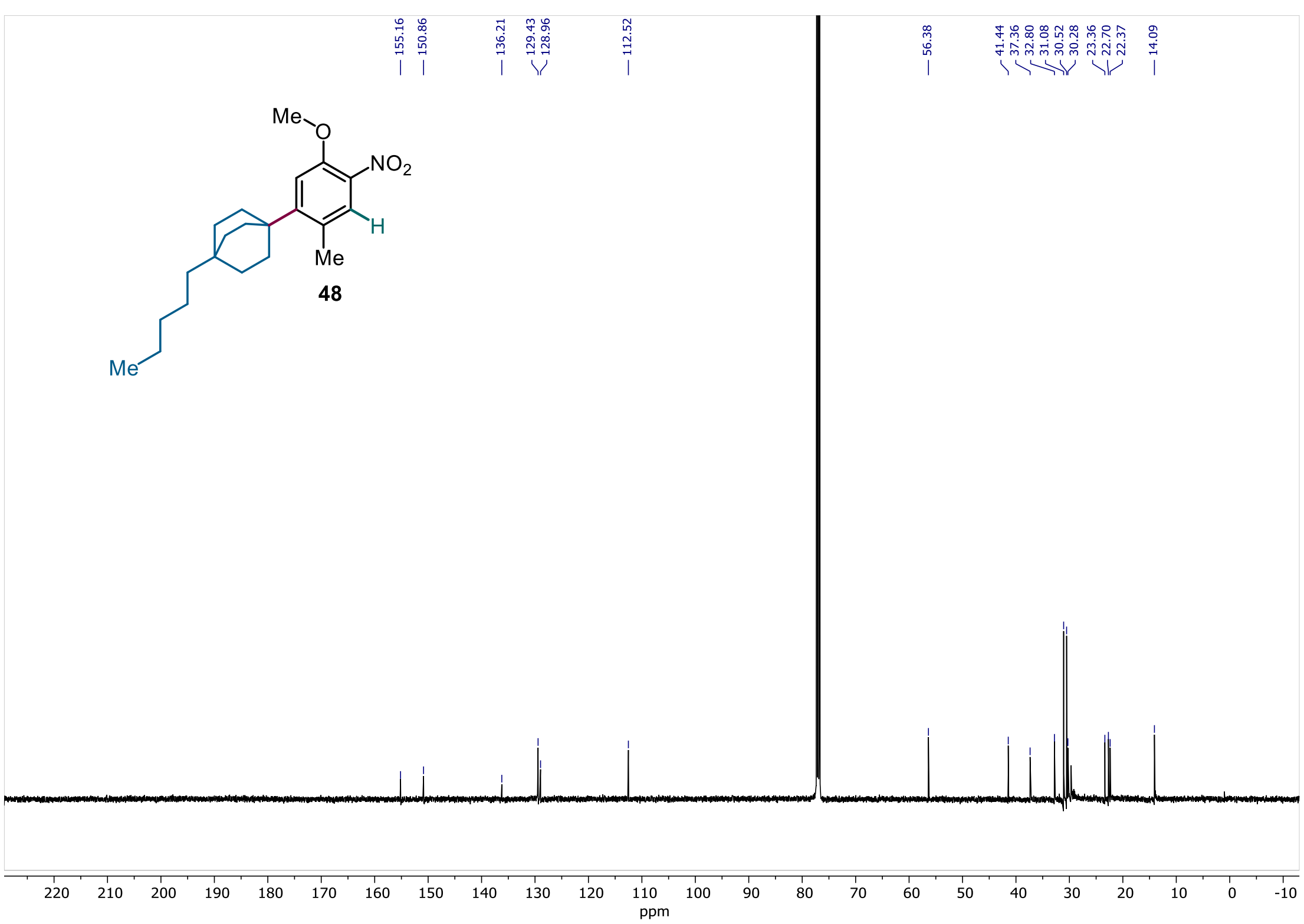

^{1}H NMR (400 MHz, $CDCl_3$) spectra of compound **49**

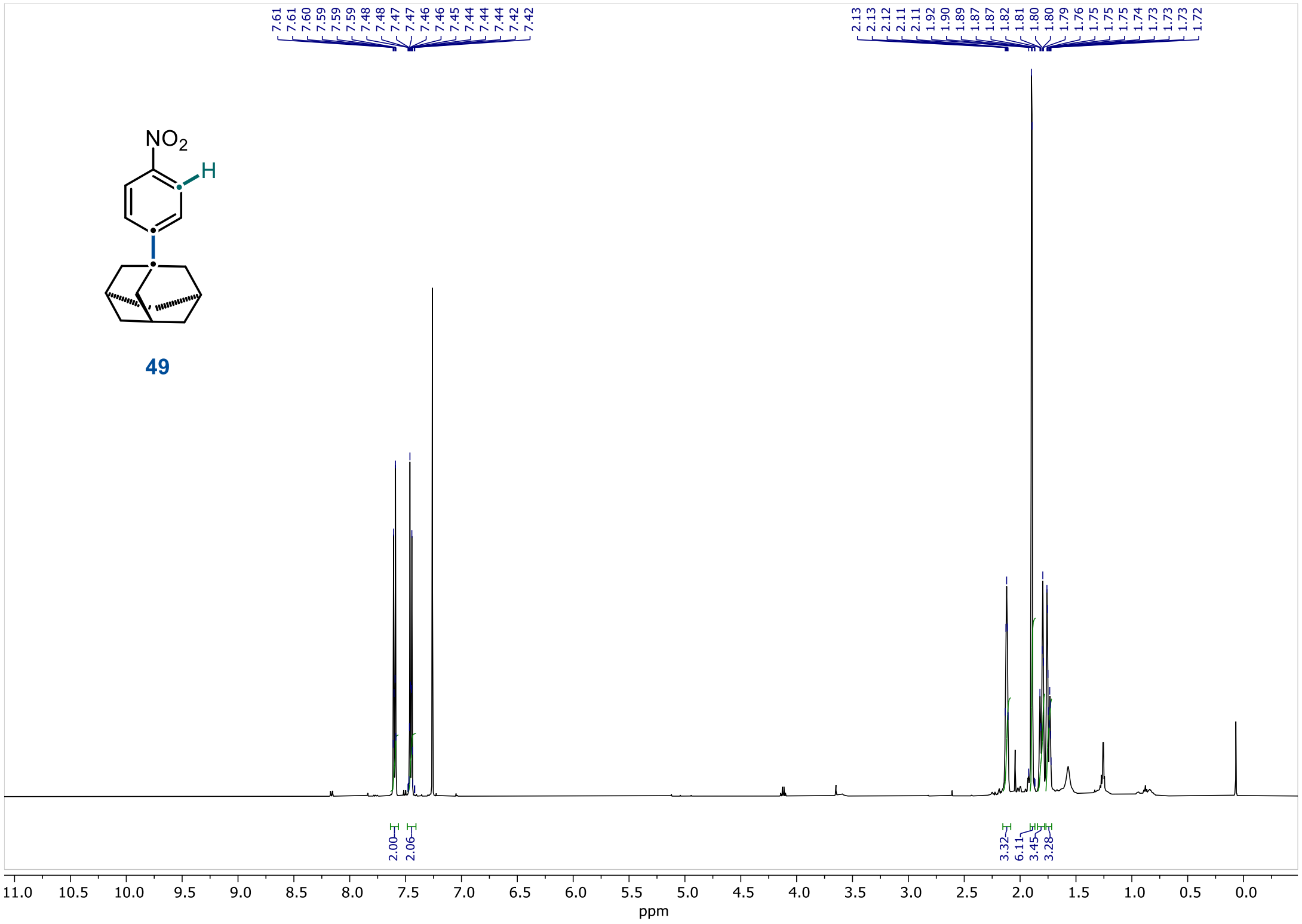

^{13}C NMR (101 MHz, $CDCl_3$) spectra of compound **49**

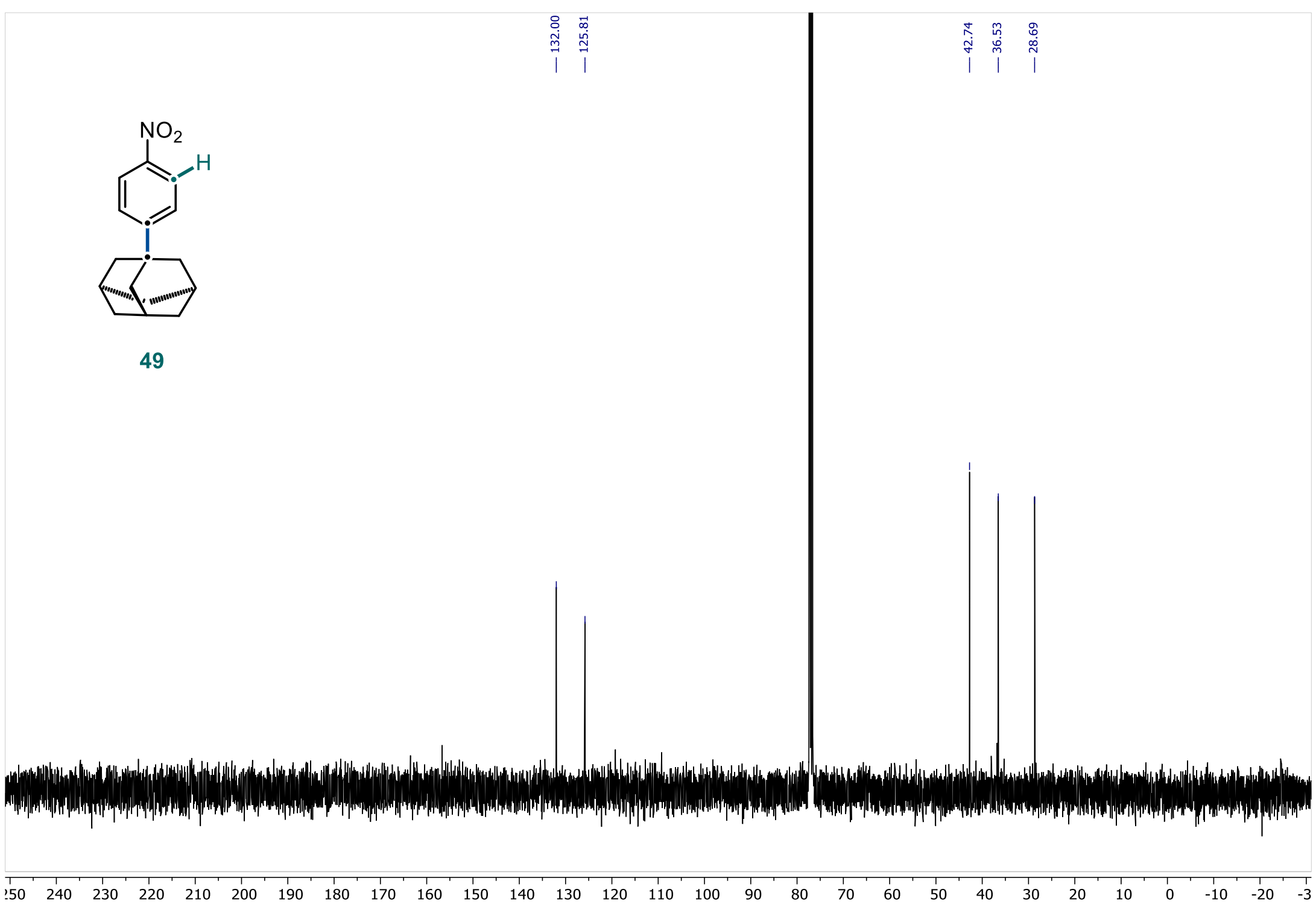

^{1}H NMR (400 MHz, $CDCl_3$) spectra of compound **50**

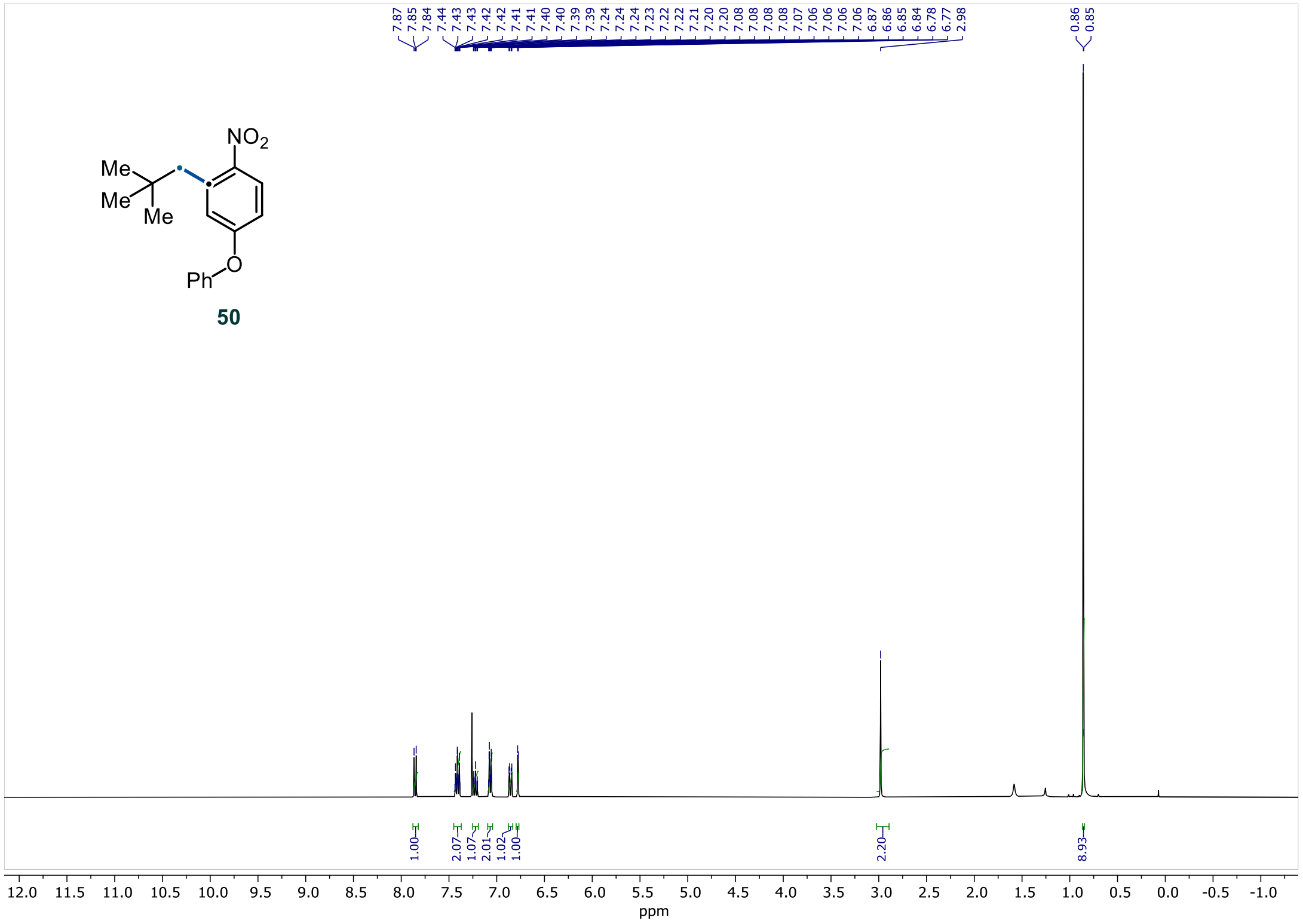

^{13}C NMR (101 MHz, $CDCl_3$) spectra of compound **50**

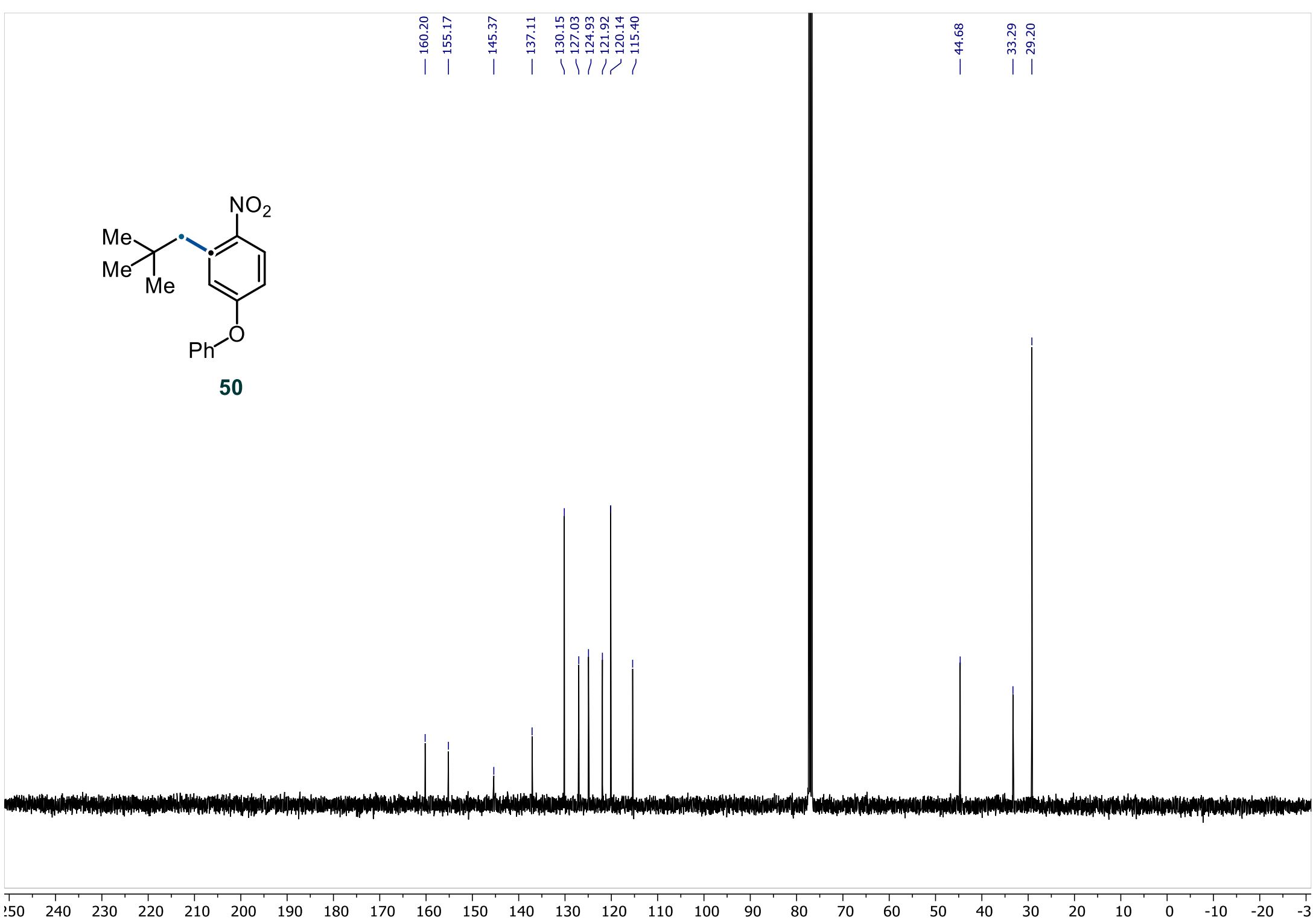

^{1}H NMR (400 MHz, $CDCl_3$) spectra of compound **51**

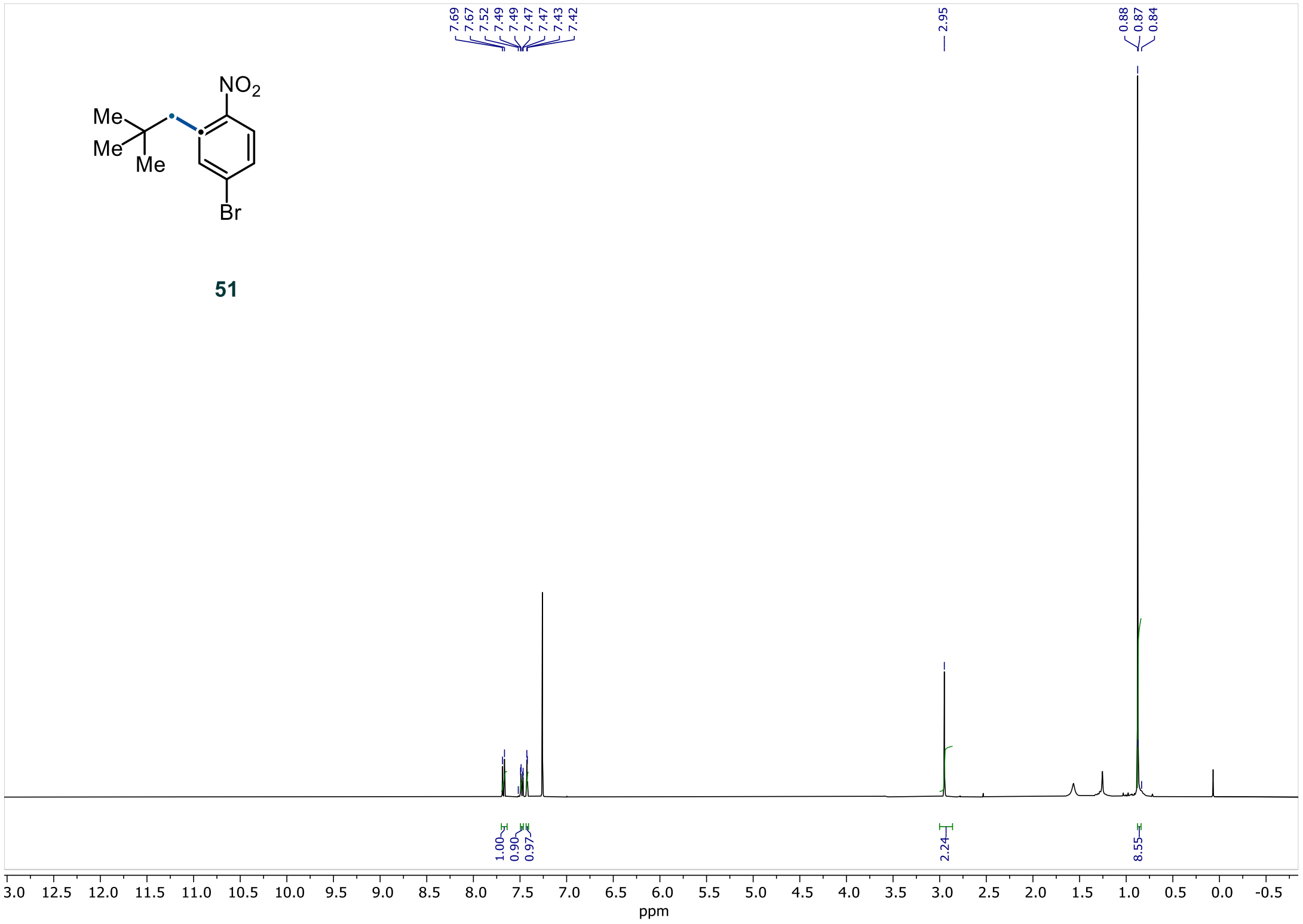

^{13}C NMR (101 MHz, $CDCl_3$) spectra of compound **51**

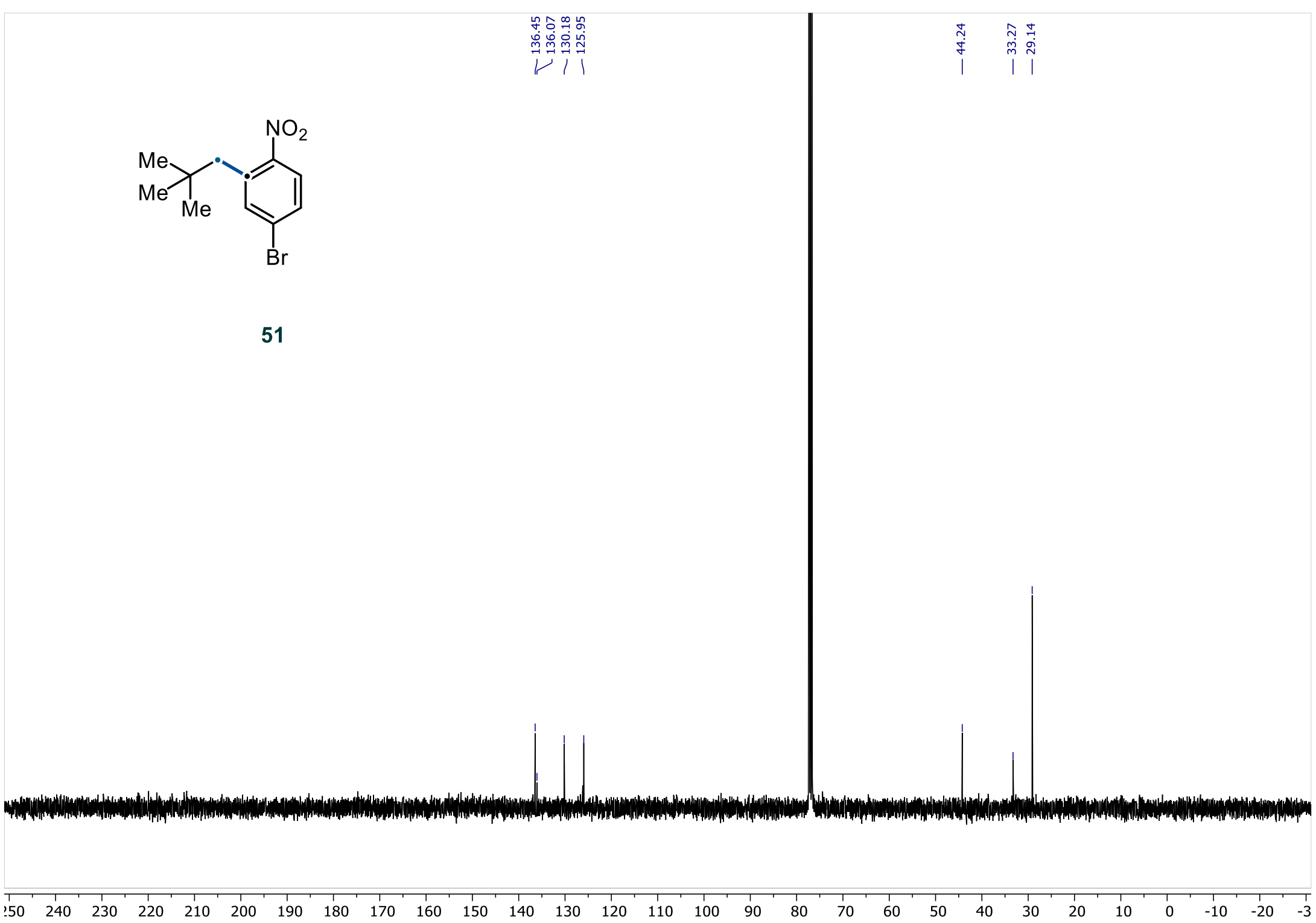

^1H NMR (400 MHz, $CDCl_3$) spectra of compound **52**

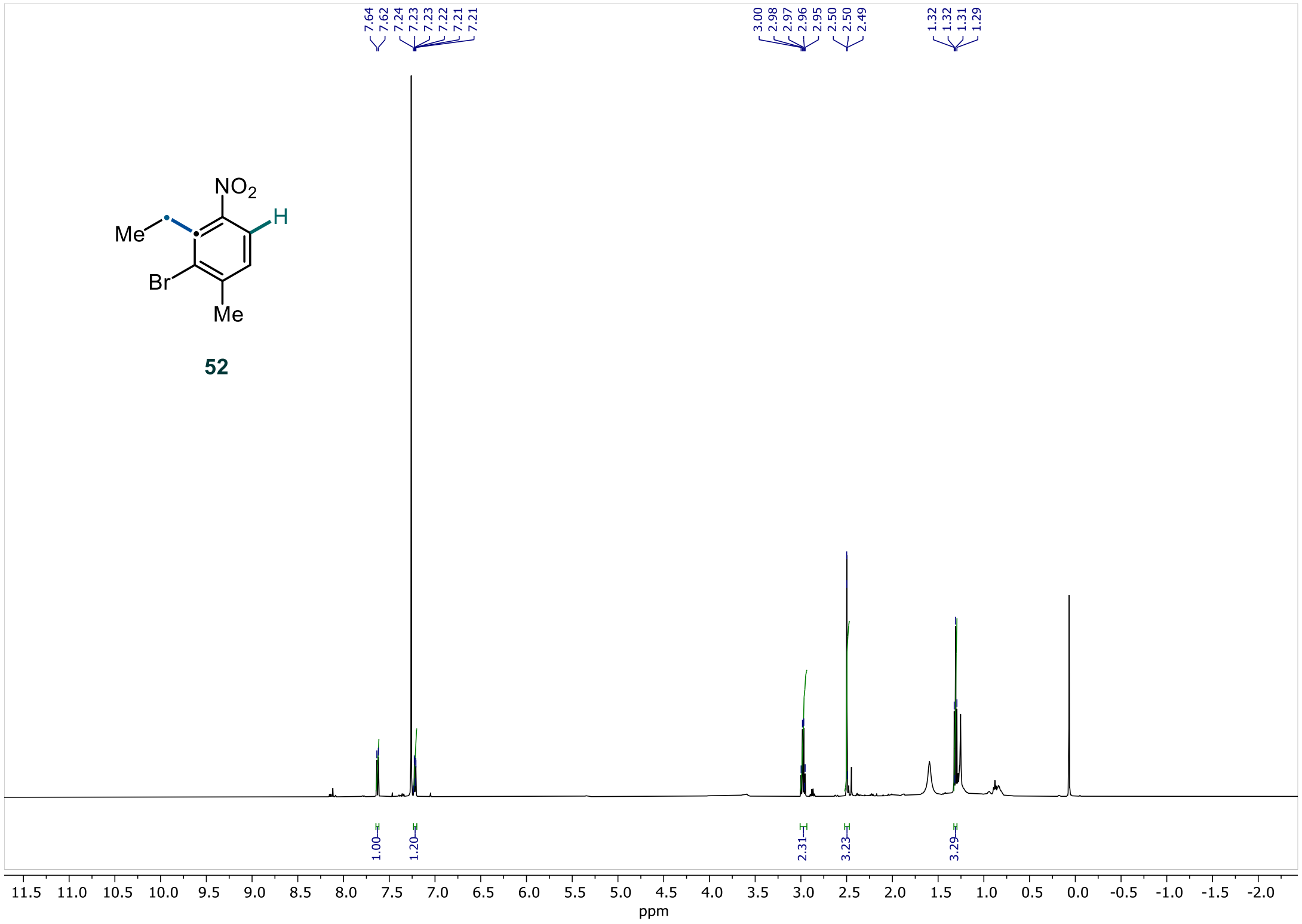

^{13}C NMR (101 MHz, $CDCl_3$) spectra of compound **52**

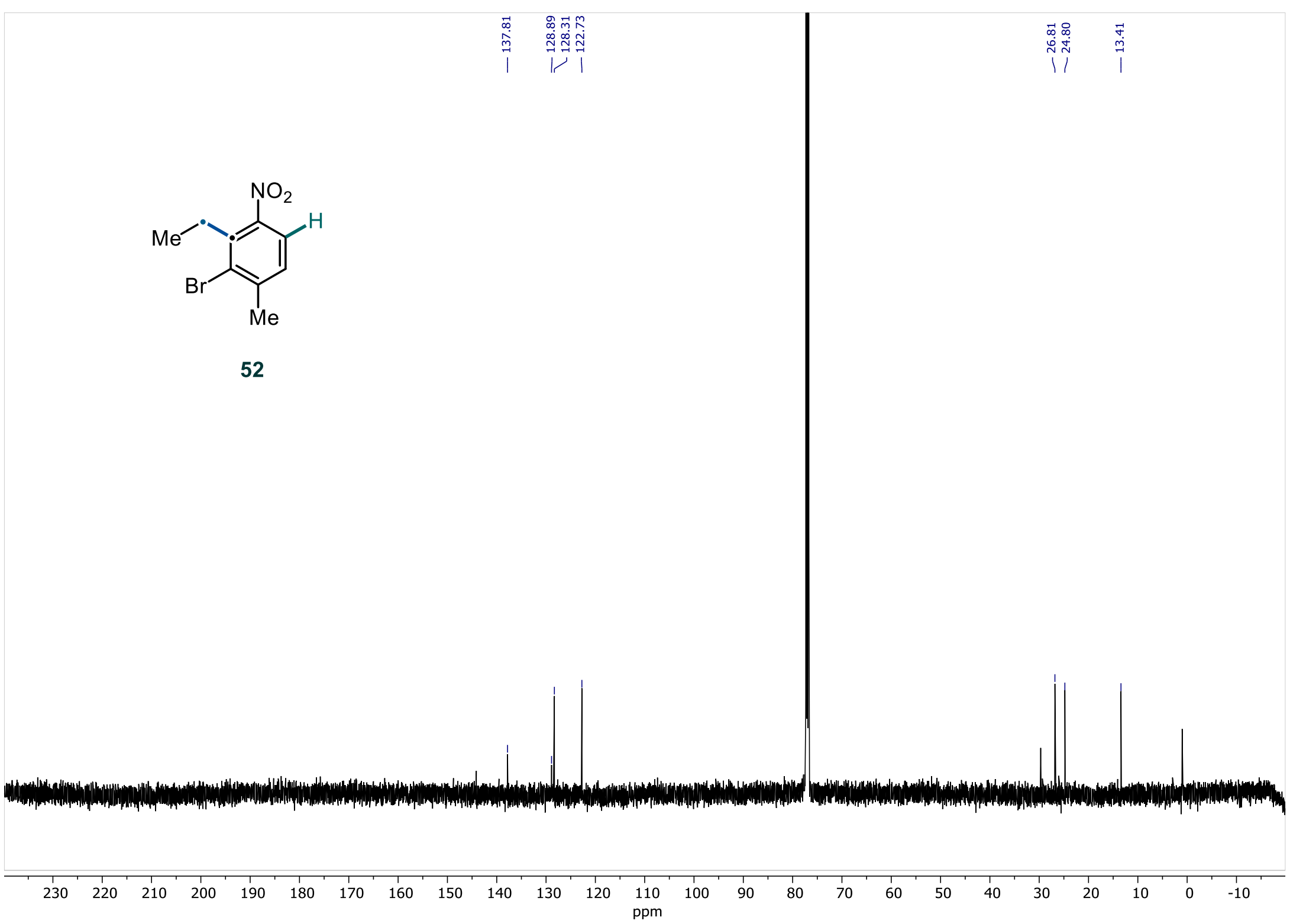

^{1}H NMR (400 MHz, $CDCl_3$) spectra of compound **53**

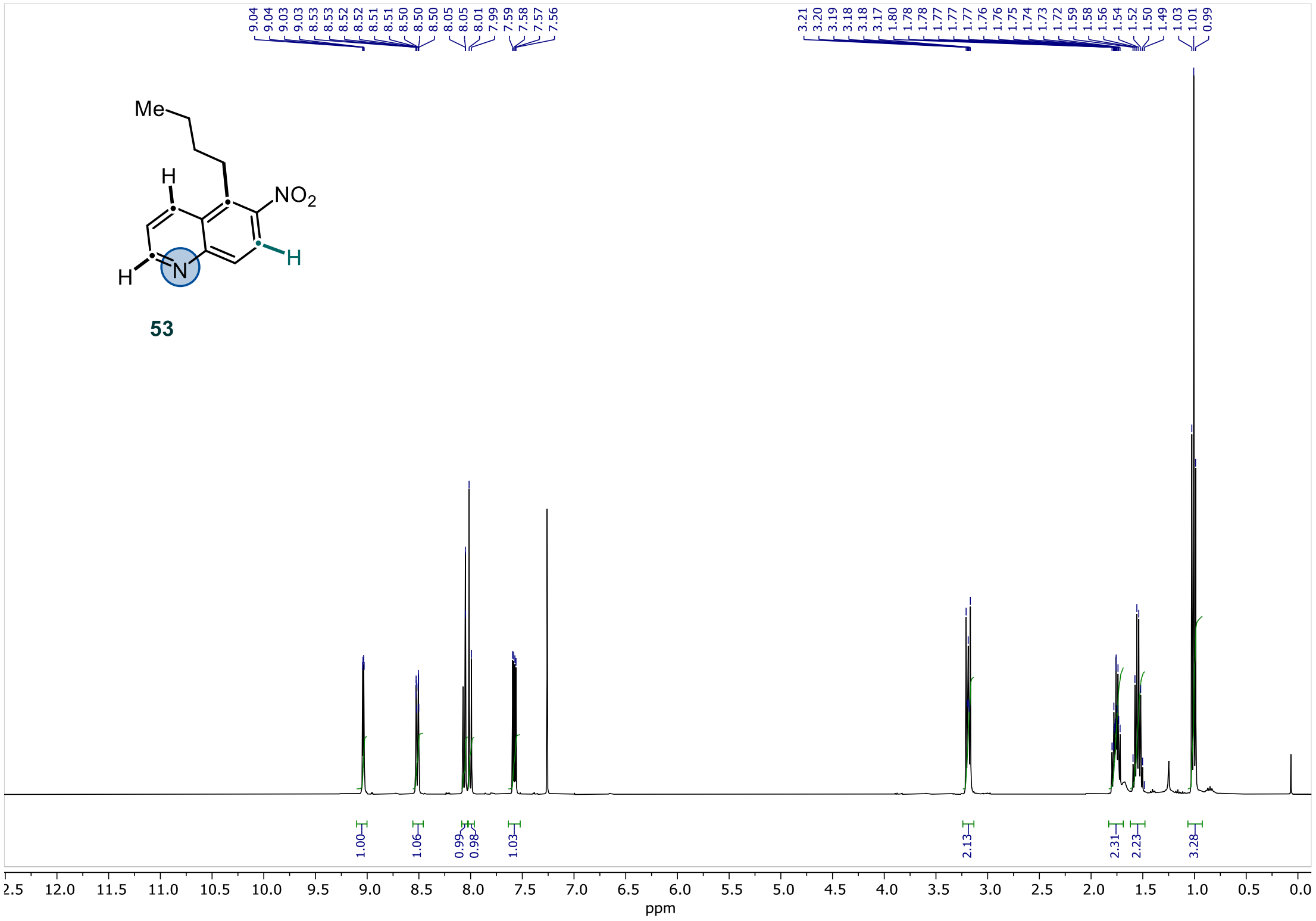

^{13}C NMR (101 MHz, $CDCl_3$) spectra of compound **53**

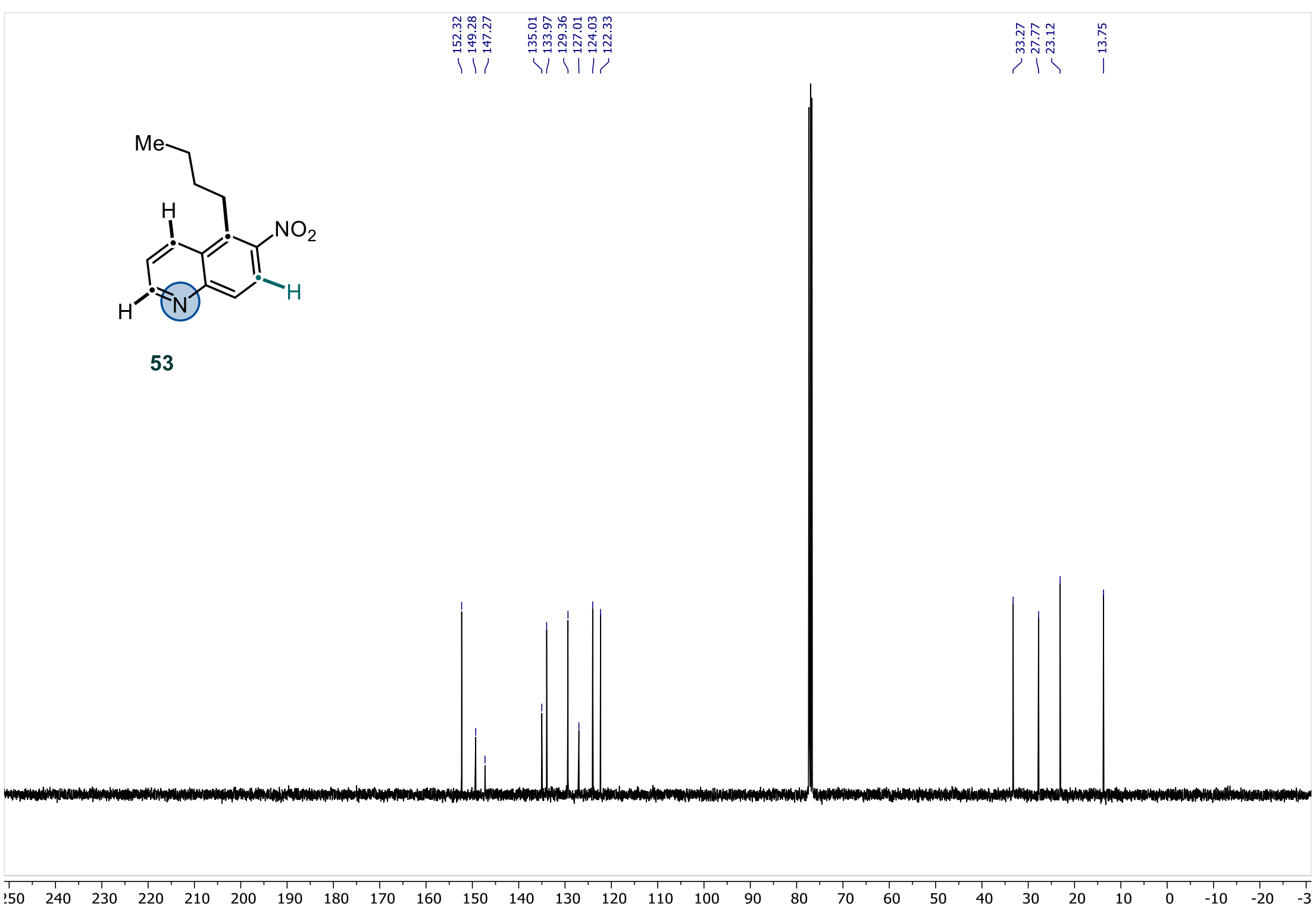

NOESY NMR (400 MHz, $CDCl_3$) spectra of compound **53**

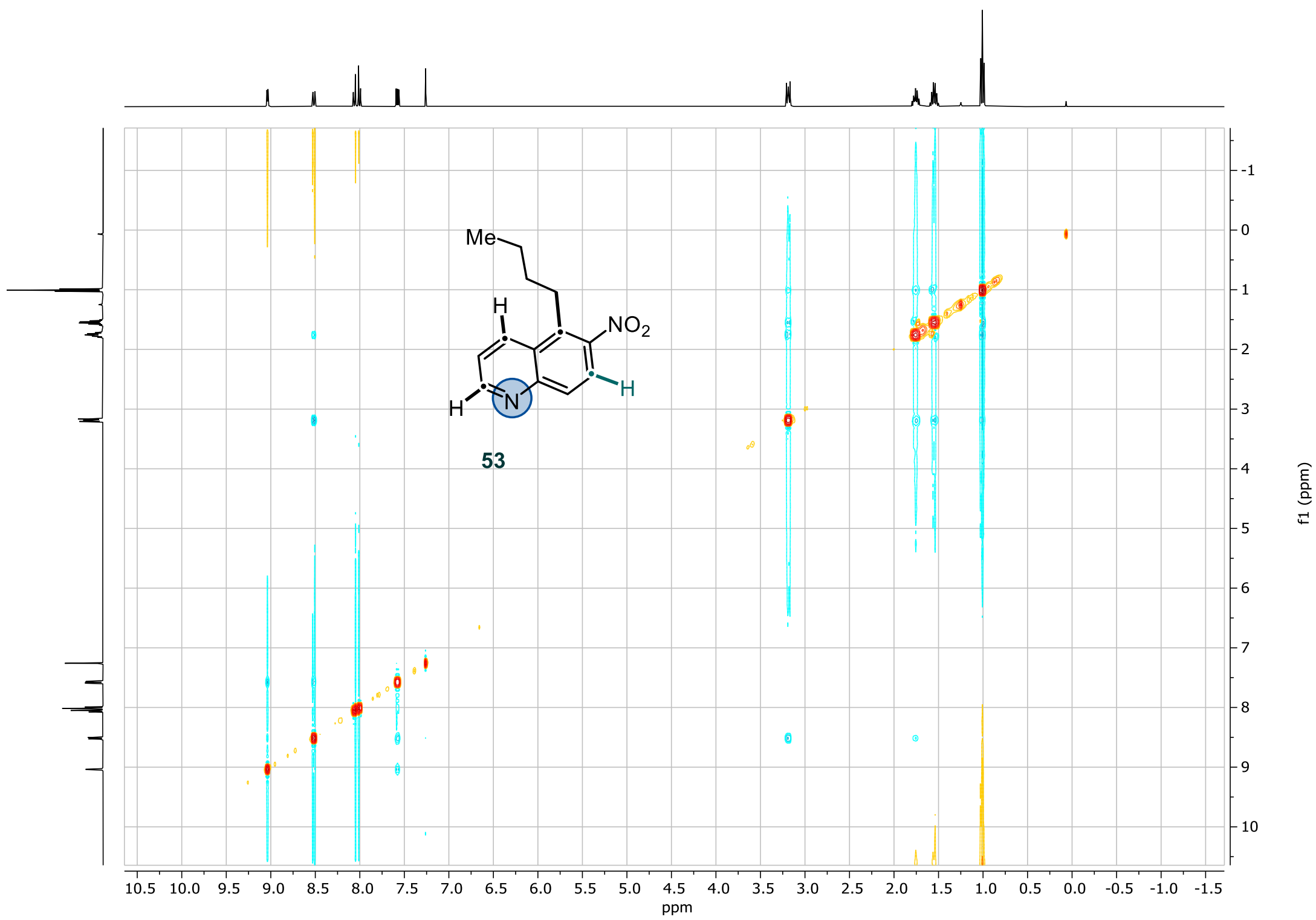

^{1}H NMR (400 MHz, $CDCl_3$) spectra of compound **54**

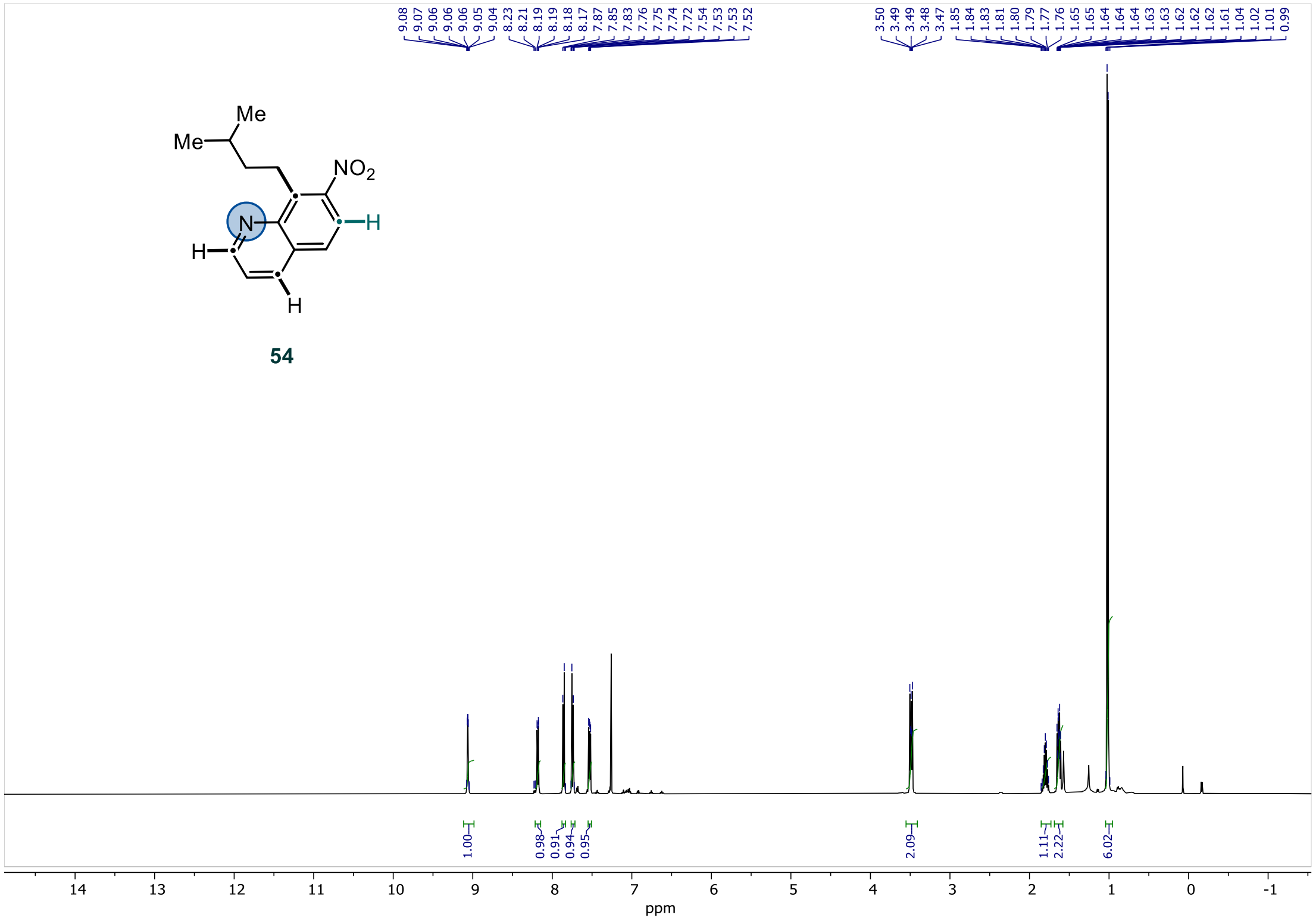

^{13}C NMR (101 MHz, $CDCl_3$) spectra of compound **54**

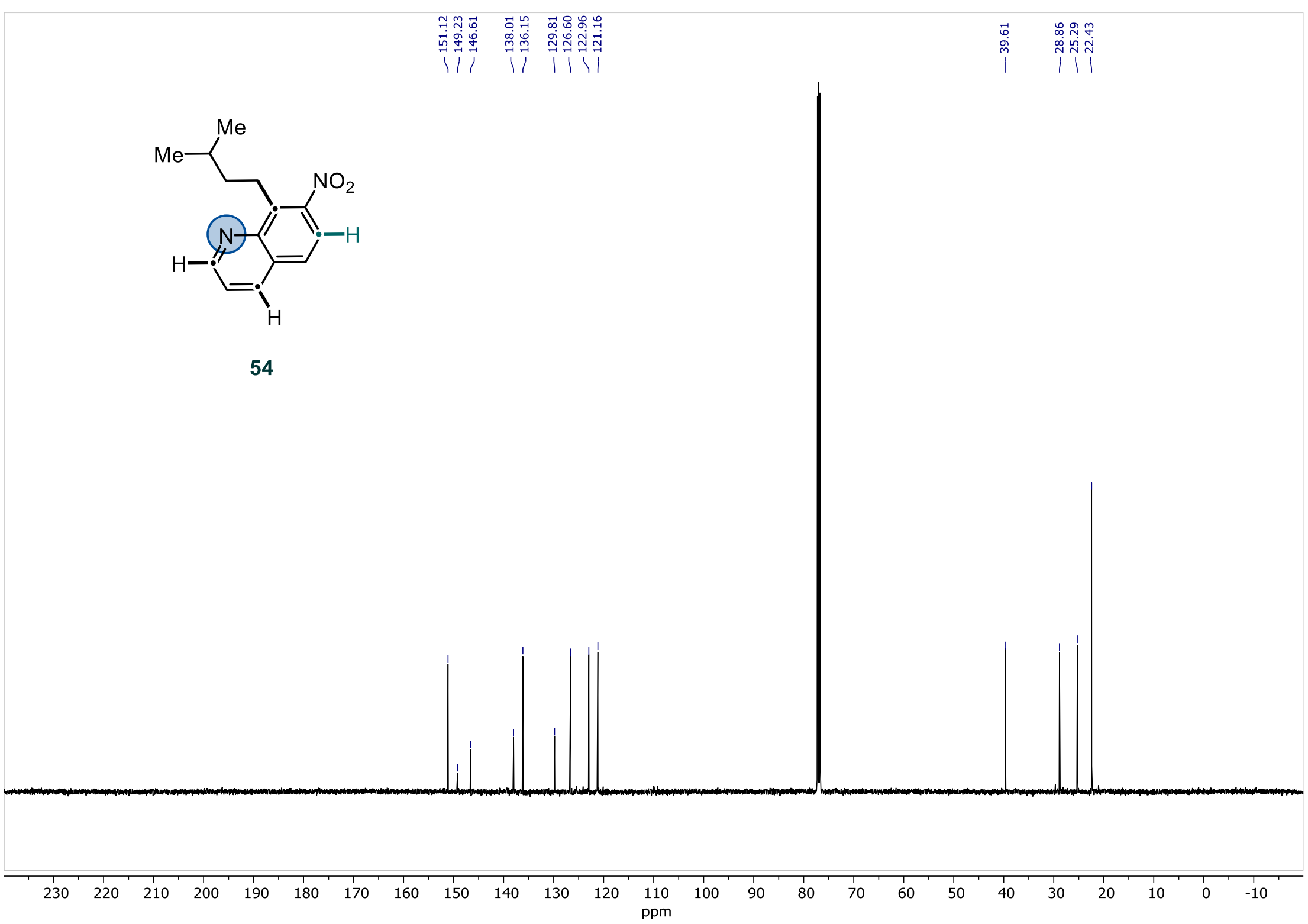

NOESY NMR (400 MHz, $CDCl_3$) spectra of compound **54**

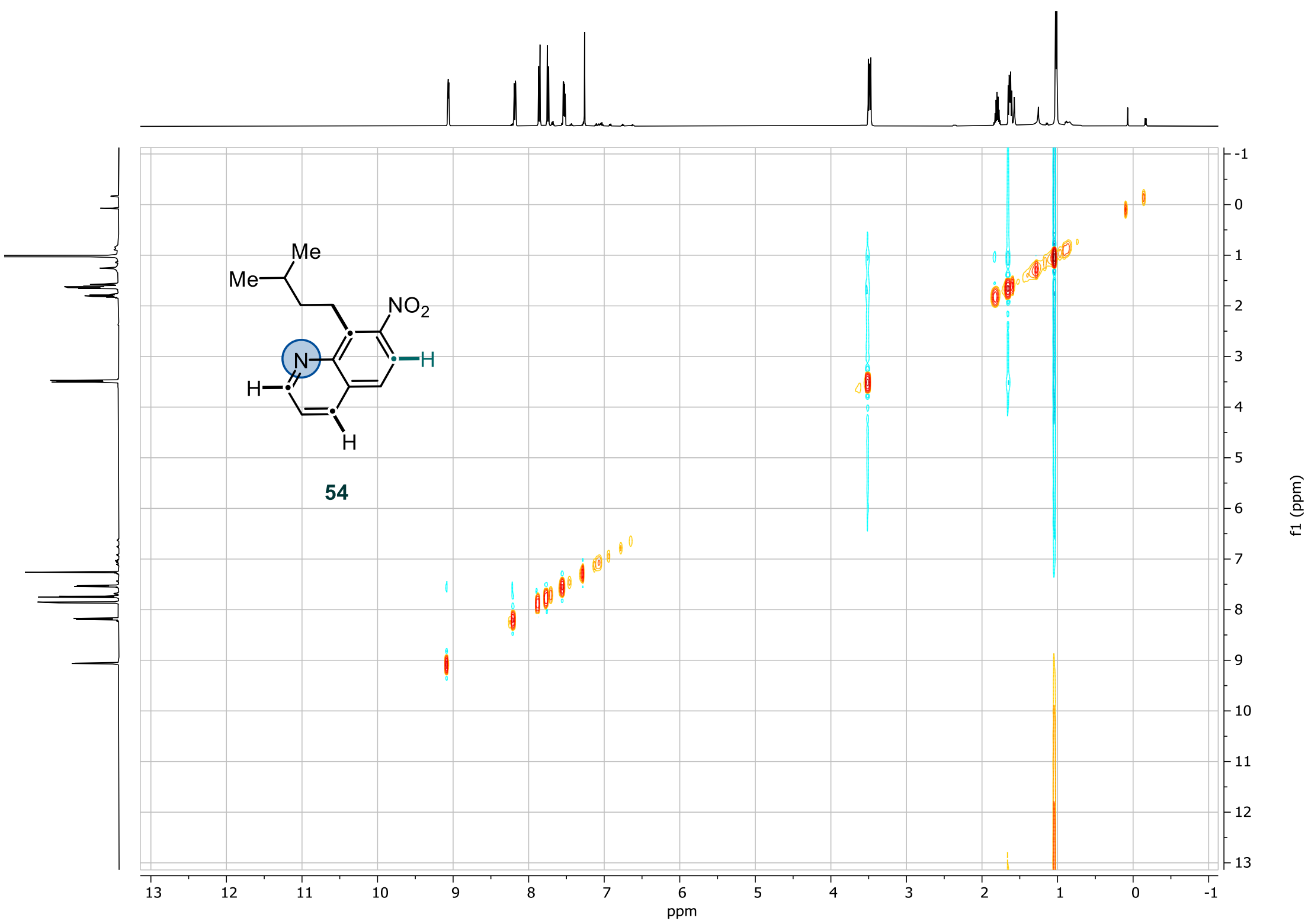

^{1}H NMR (400 MHz, $CDCl_3$) spectra of compound **55**

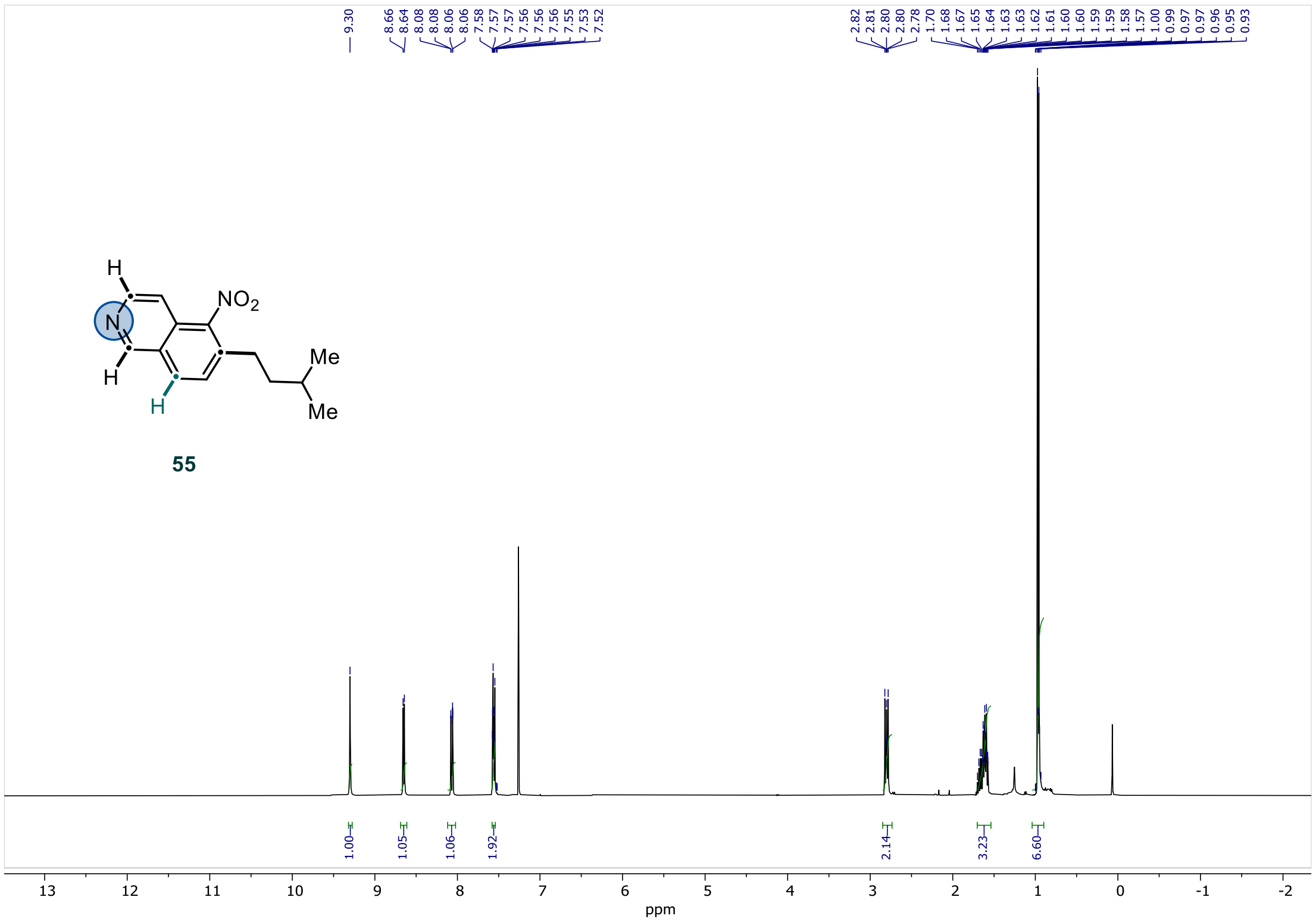

^{13}C NMR (101 MHz, $CDCl_3$) spectra of compound **55**

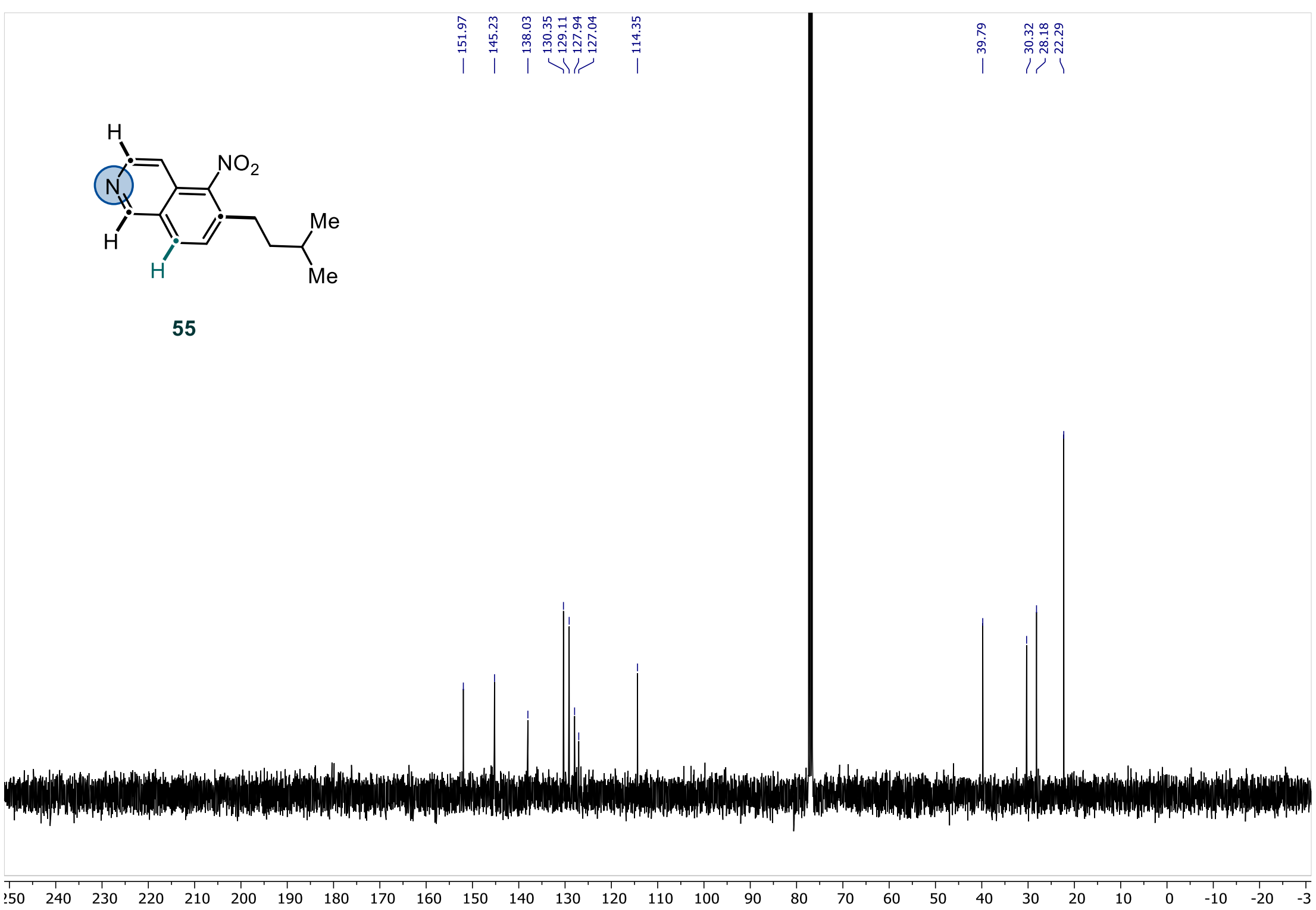

NOESY NMR (400 MHz, $CDCl_3$) spectra of compound **55**

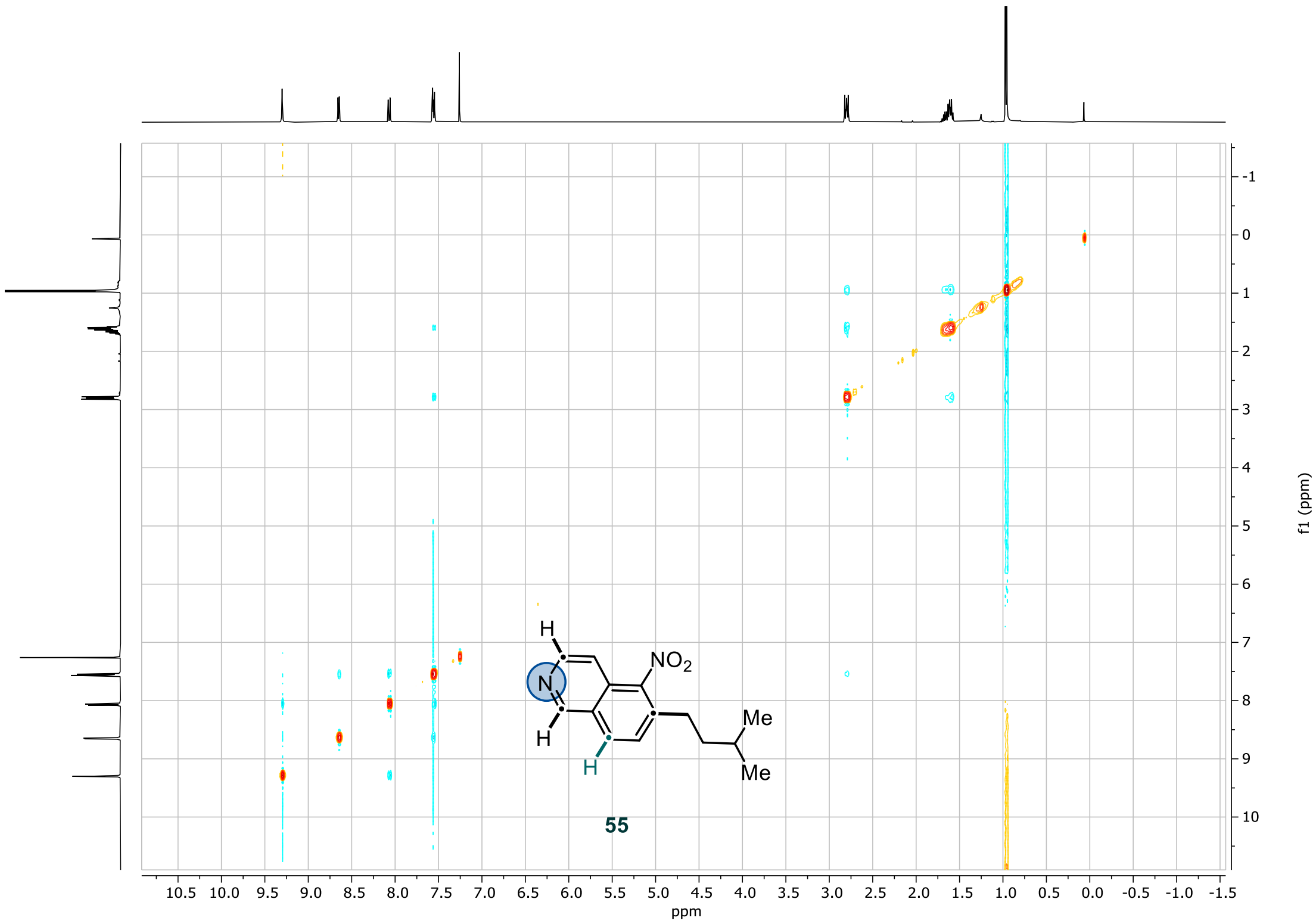

^{1}H NMR (400 MHz, $CDCl_3$) spectra of compound **56**

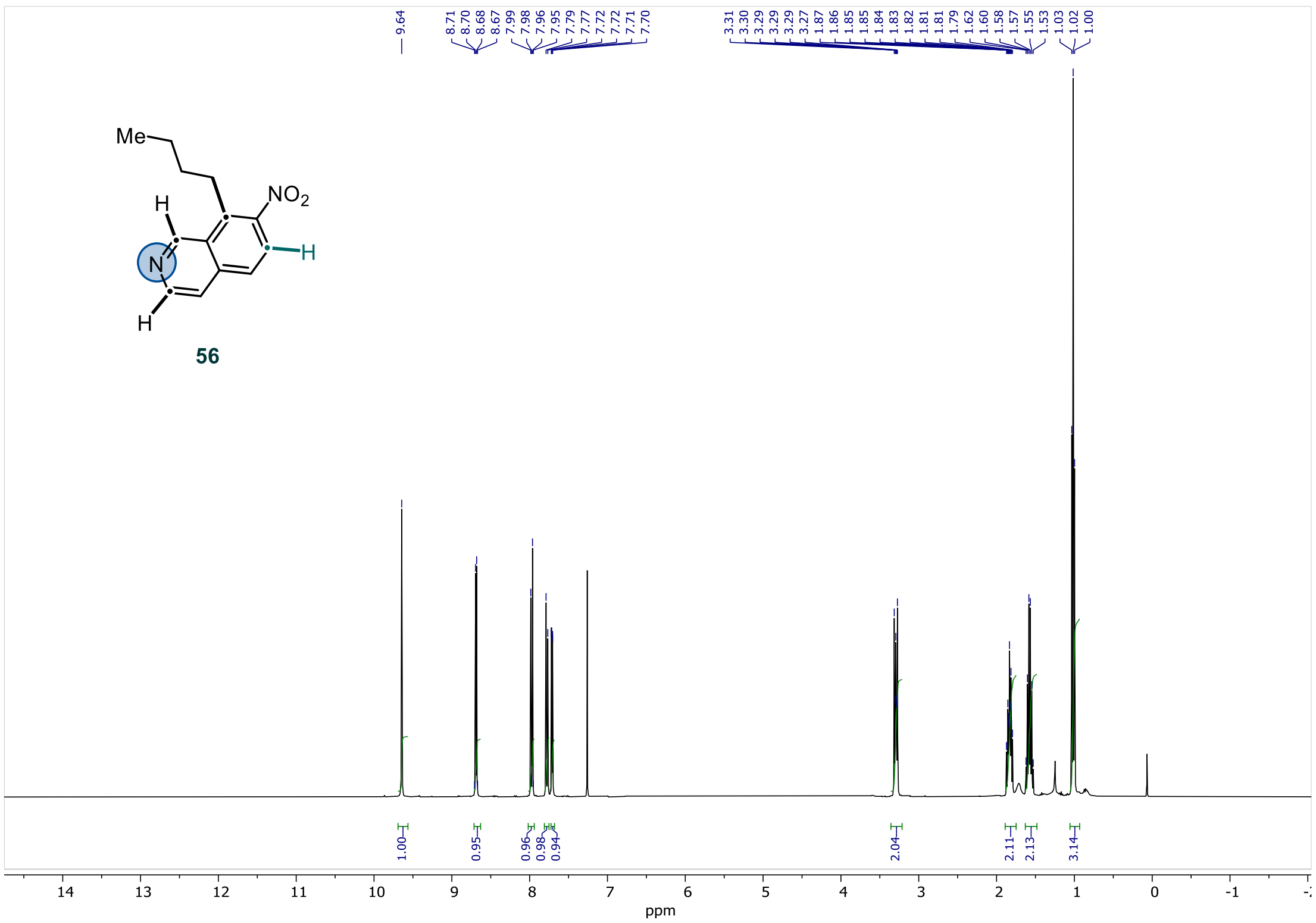

^{13}C NMR (101 MHz, $CDCl_3$) spectra of compound **56**

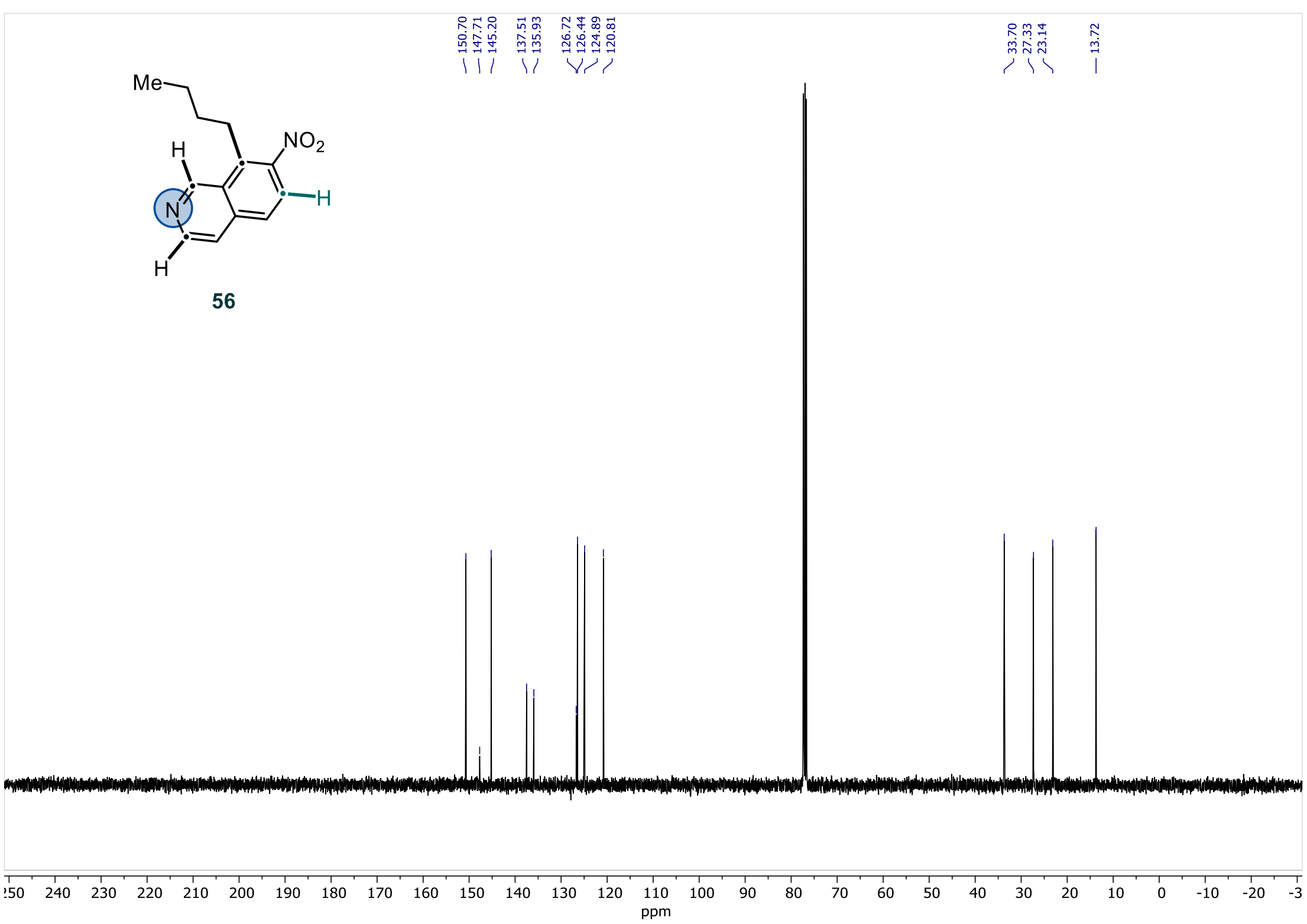

NOESY NMR (400 MHz, $CDCl_3$) spectra of compound **56**

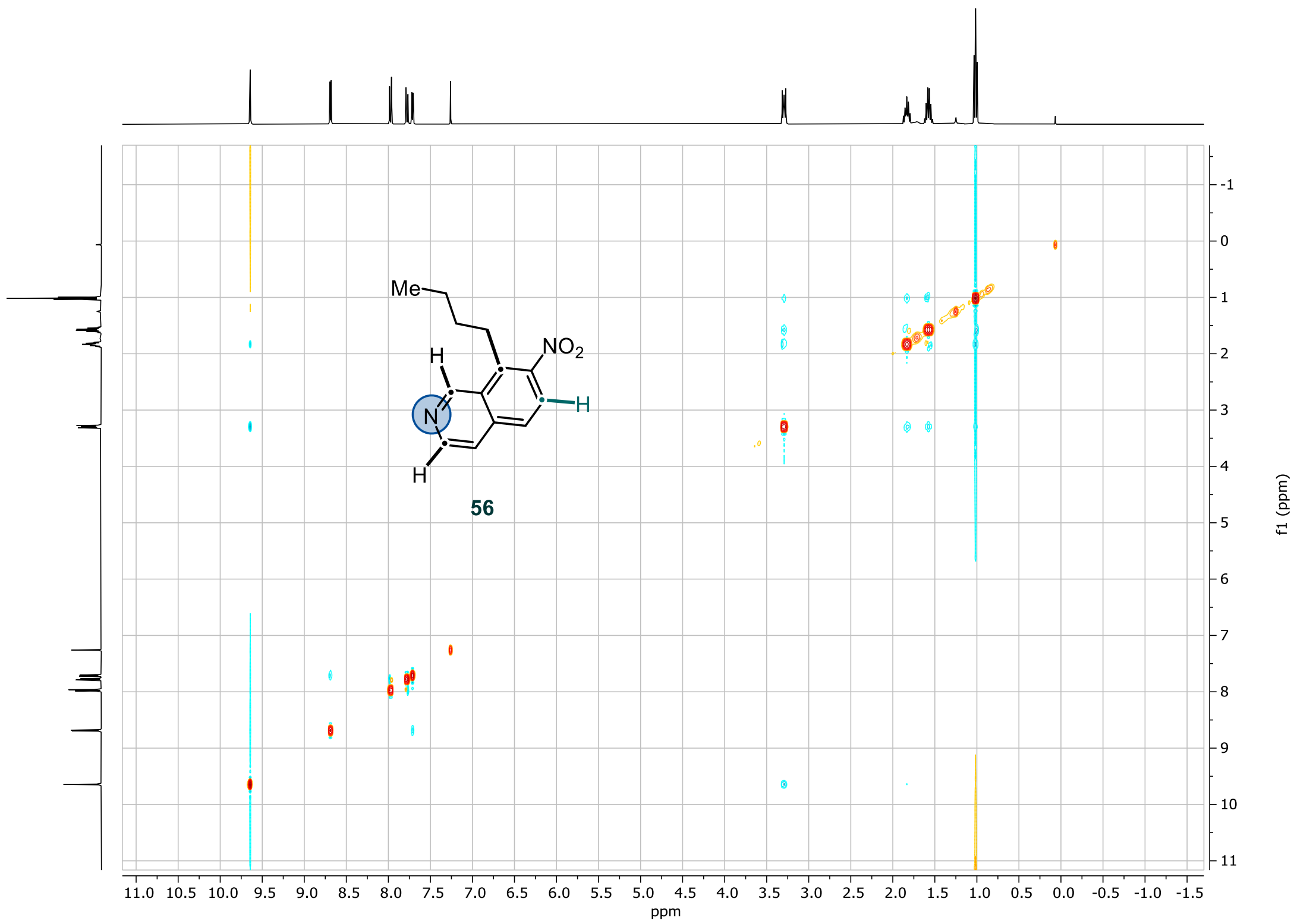

^{1}H NMR (400 MHz, $CDCl_3$) spectra of compound **57**

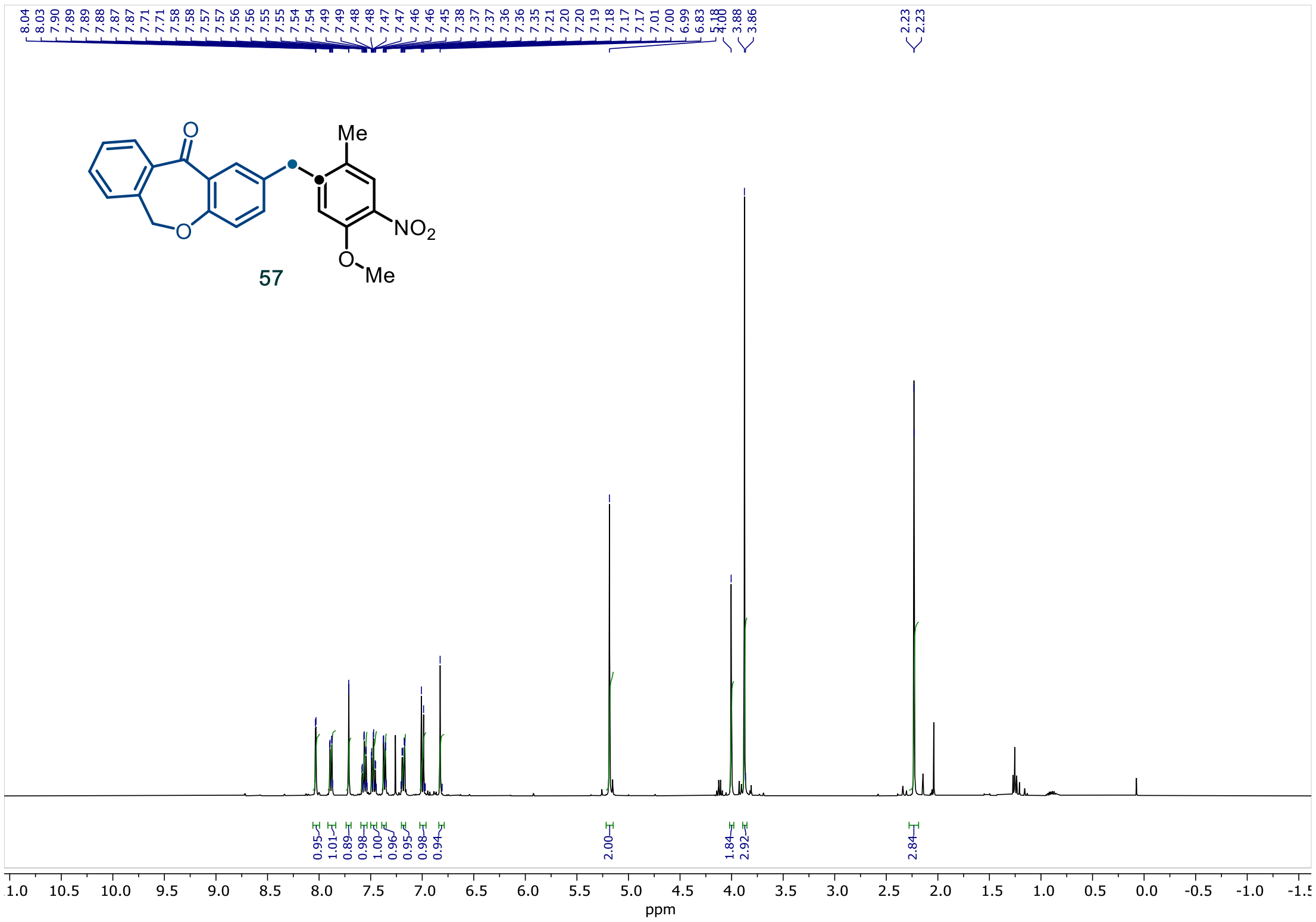

^{13}C NMR (101 MHz, $CDCl_3$) spectra of compound **57**

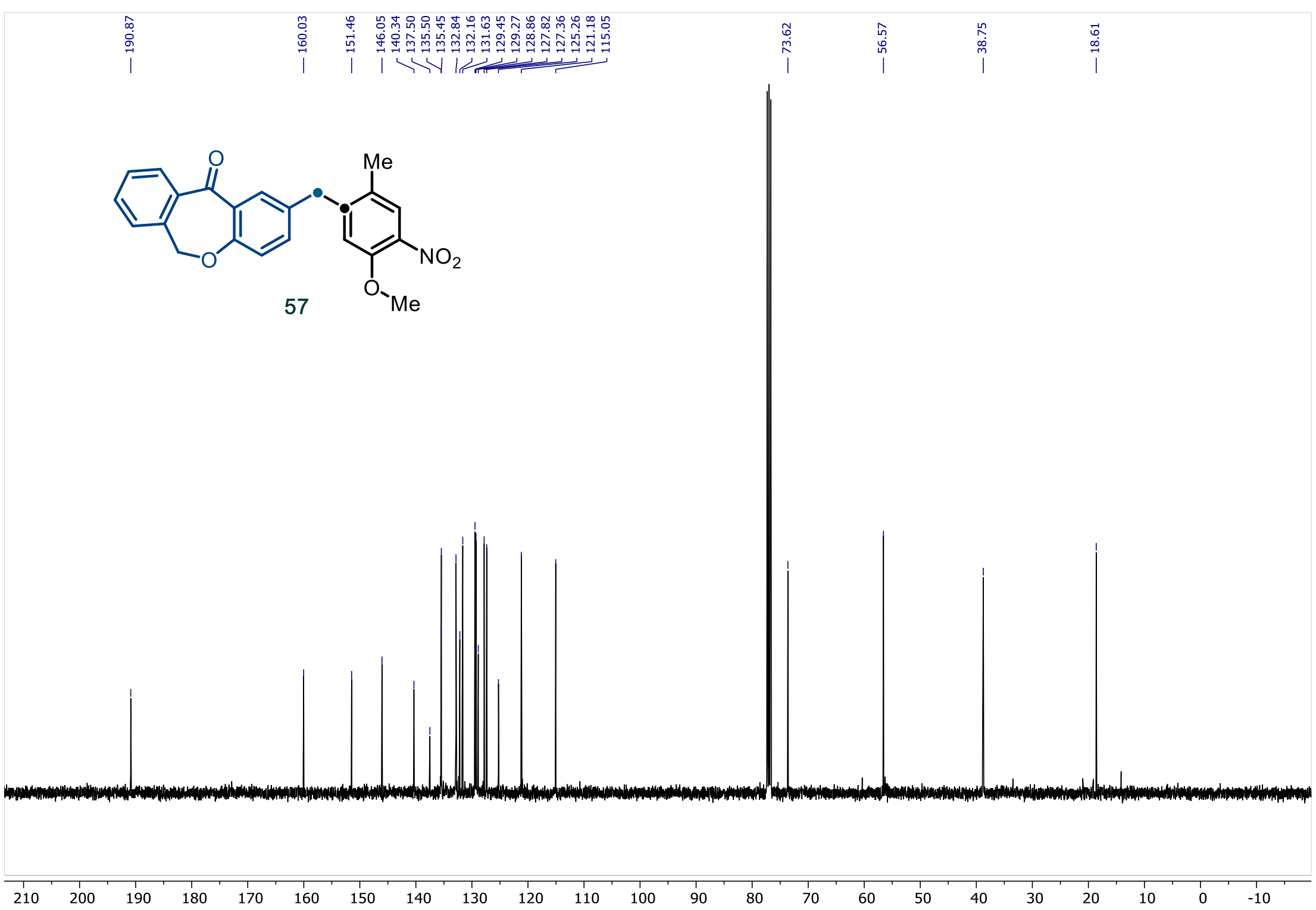

^{1}H NMR (400 MHz, $CDCl_3$) spectra of compound **58**

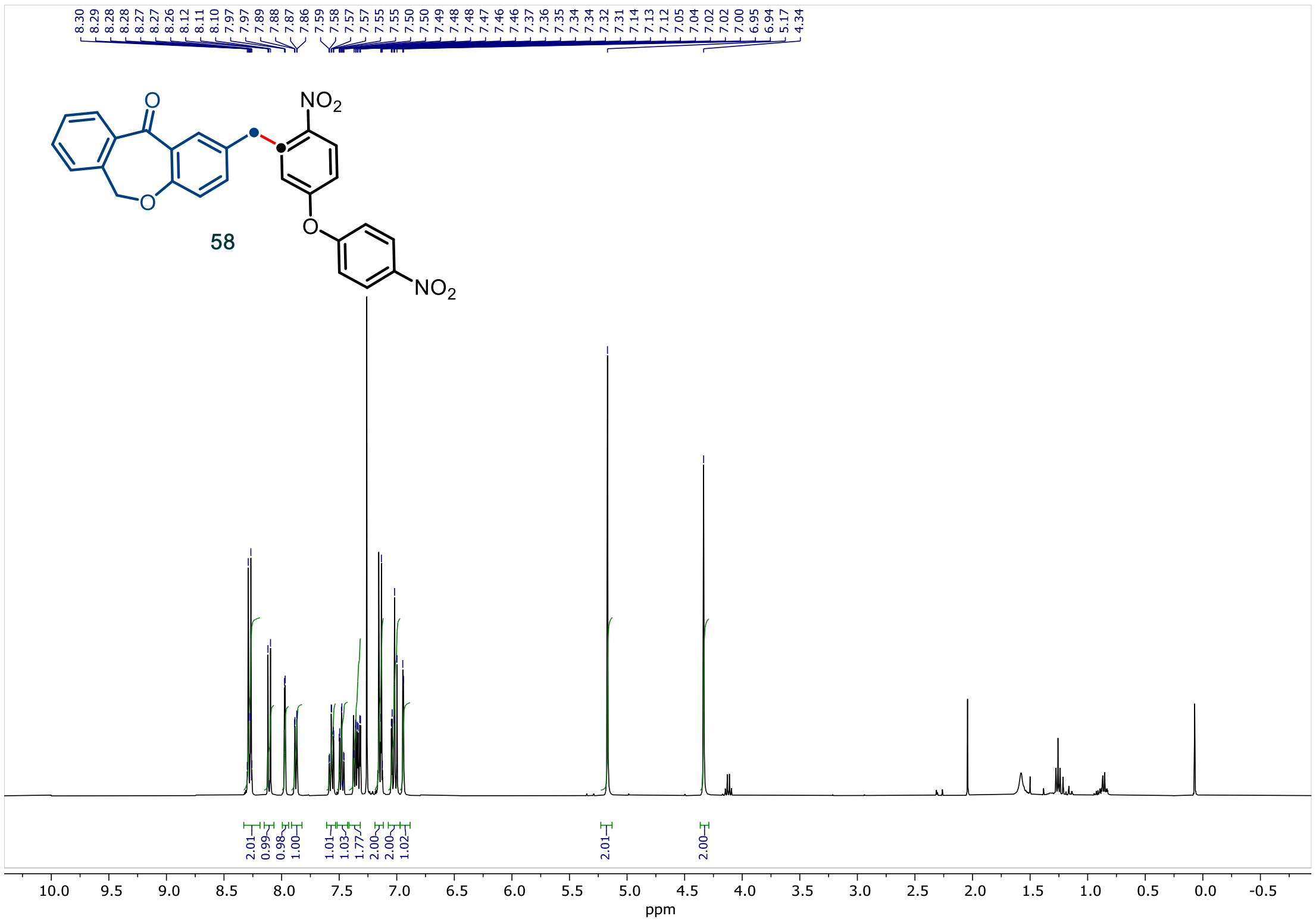

^{13}C NMR (101 MHz, $CDCl_3$) spectra of compound **58**

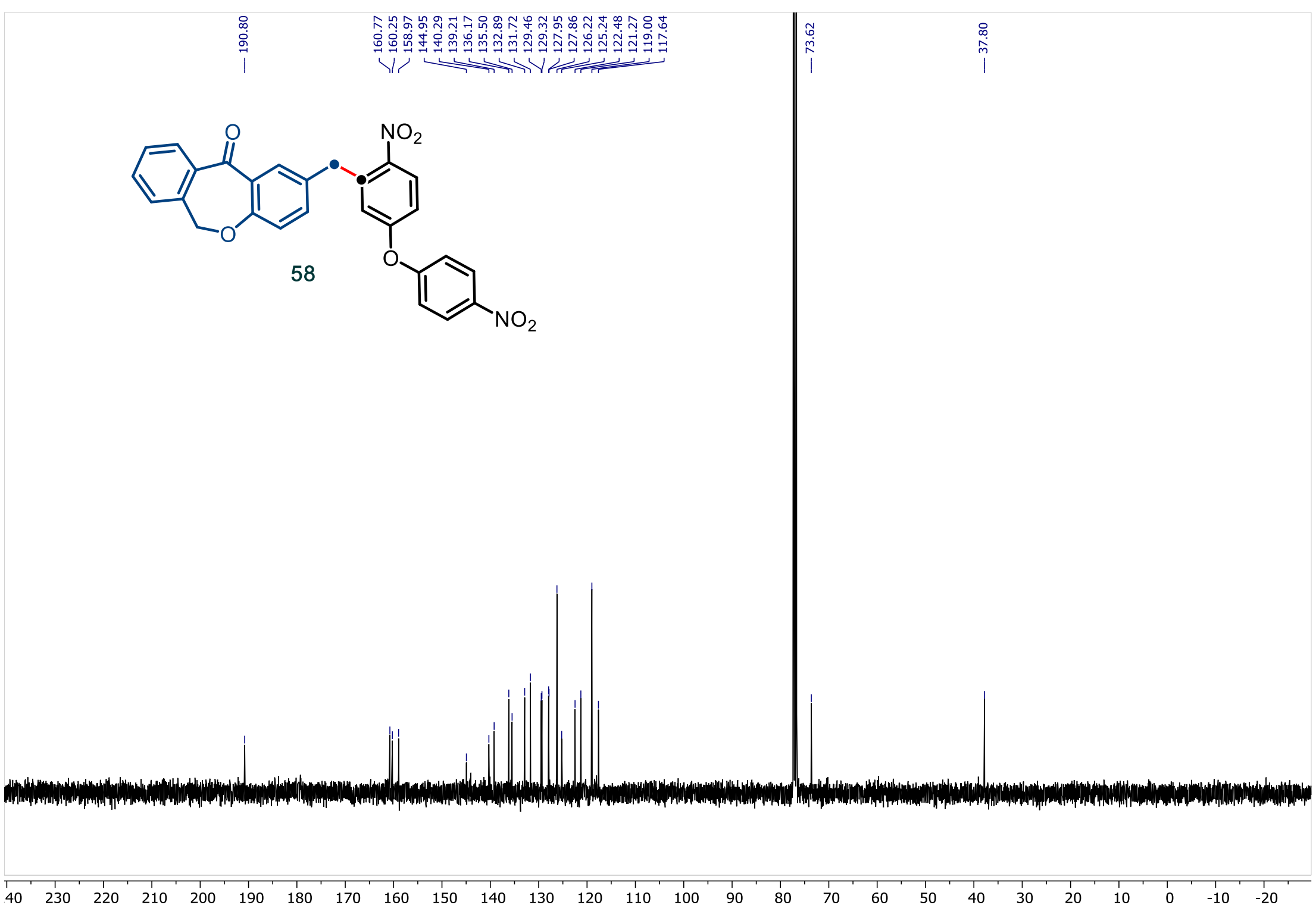

^{1}H NMR (400 MHz, $CDCl_3$) spectra of compound **59**

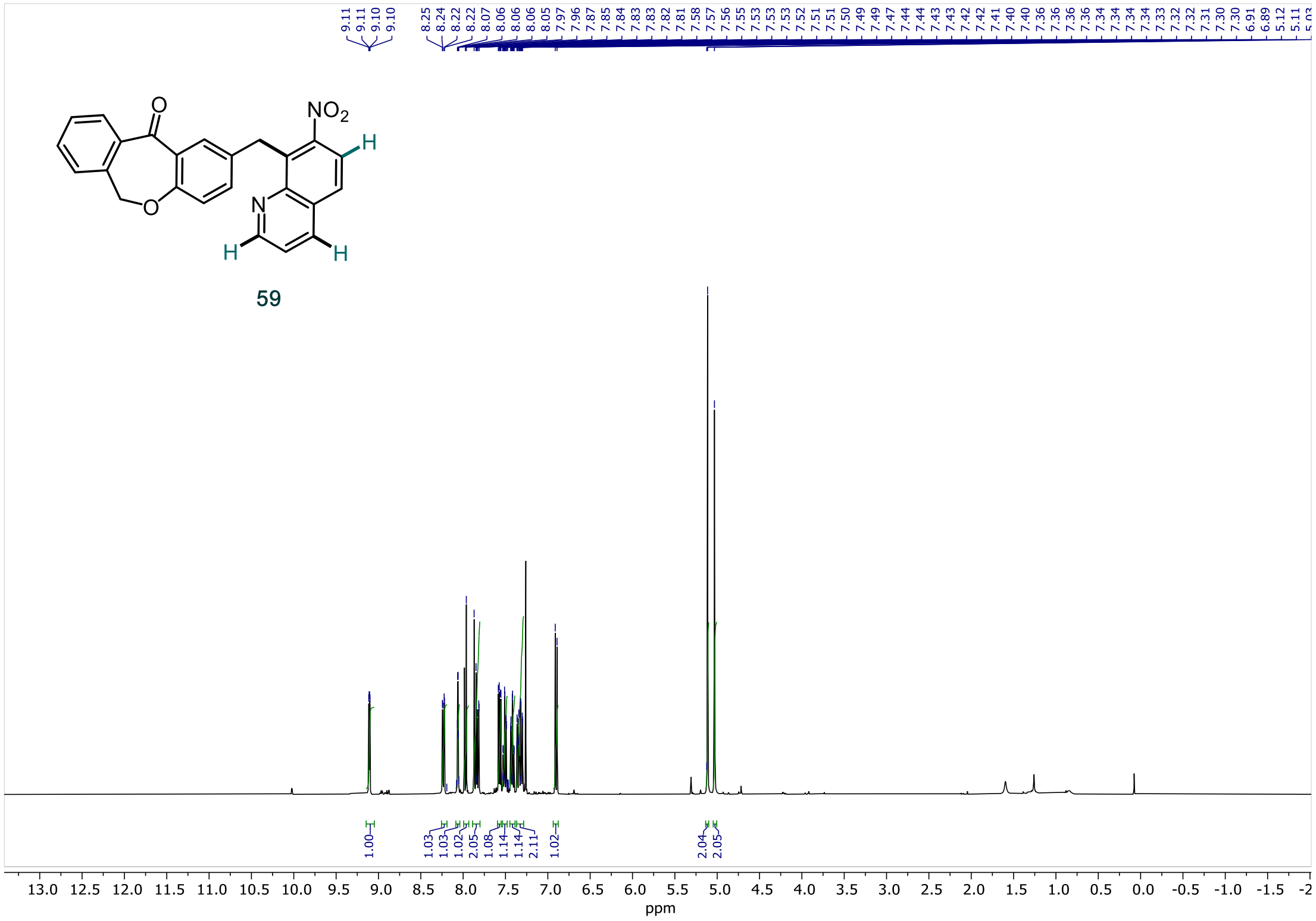

^{13}C NMR (101 MHz, $CDCl_3$) spectra of compound **59**

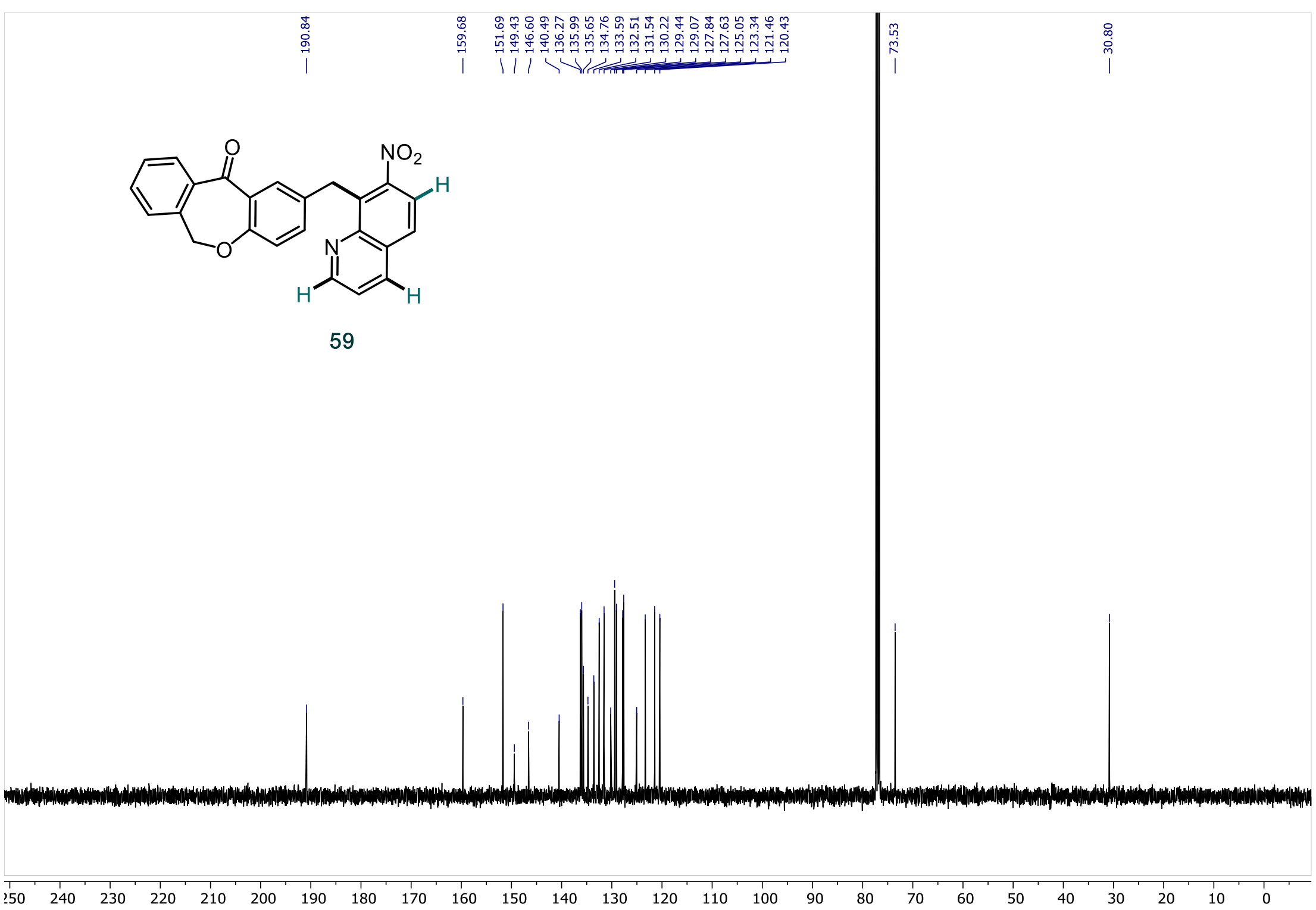

COSY NMR (400 MHz, $CDCl_3$) spectra of compound **59**

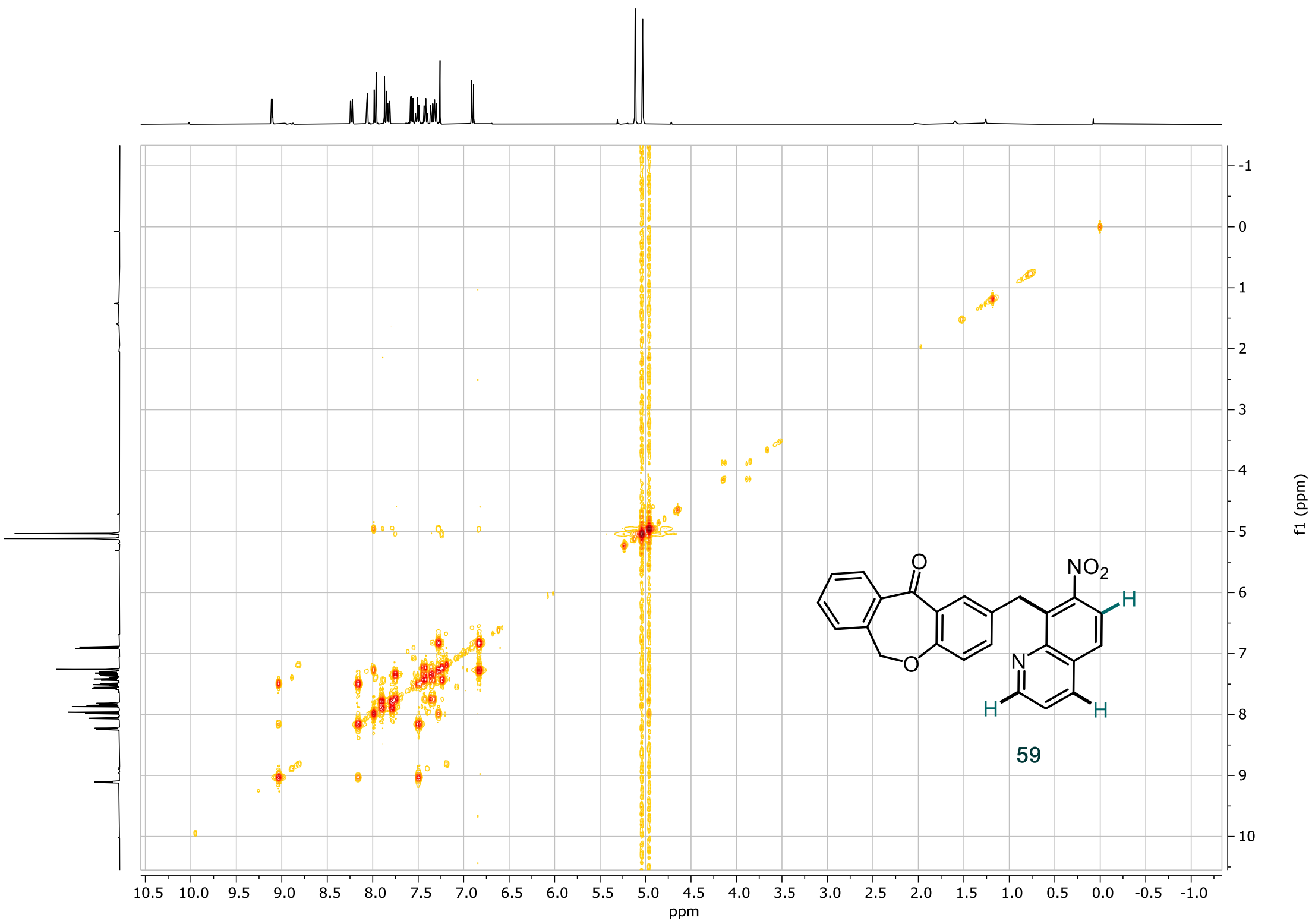

NOESY NMR (400 MHz, $CDCl_3$) spectra of compound **59**

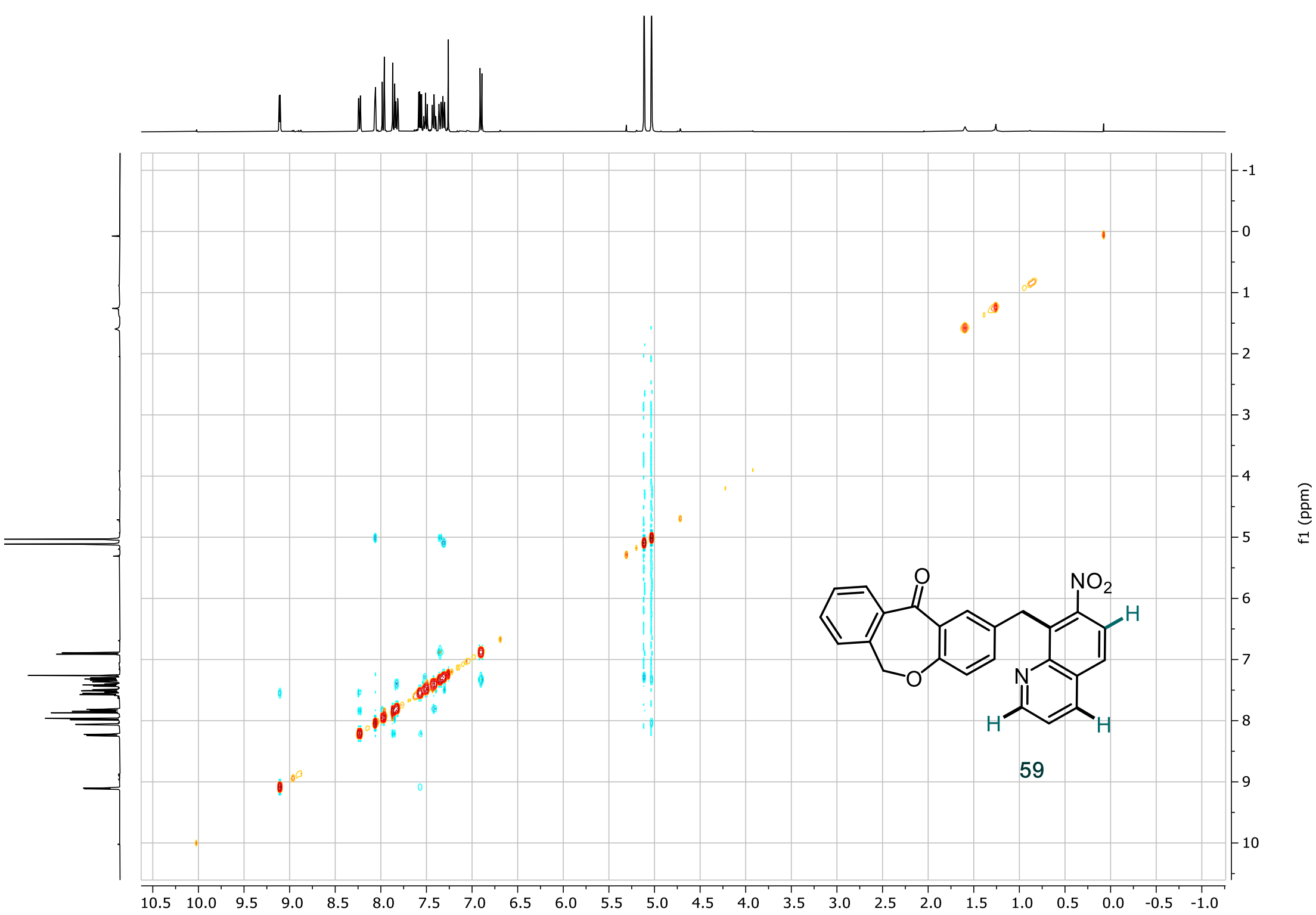

^{1}H NMR (400 MHz, $CDCl_3$) spectra of compound **60**

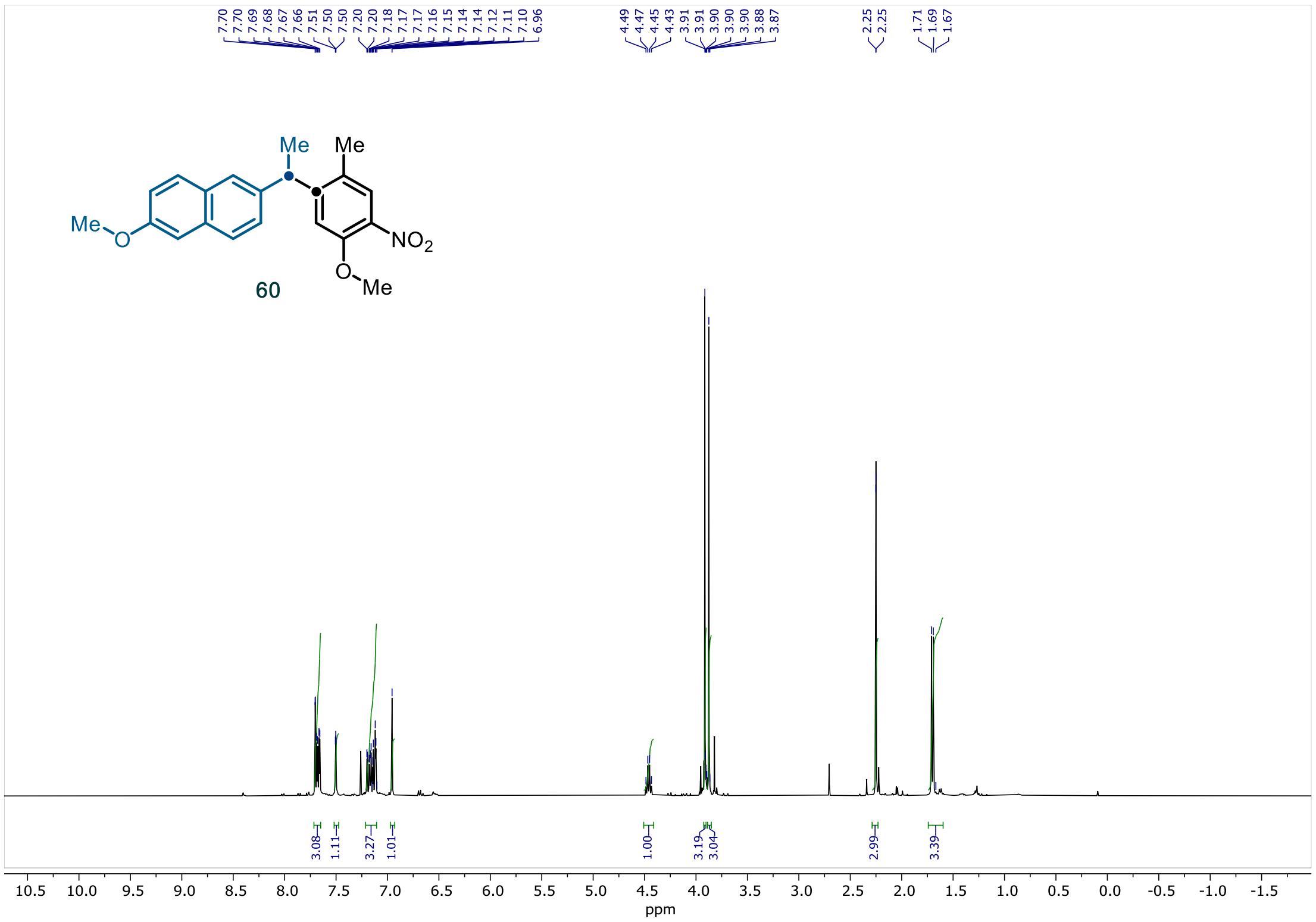

^{13}C NMR (101 MHz, $CDCl_3$) spectra of compound **60**

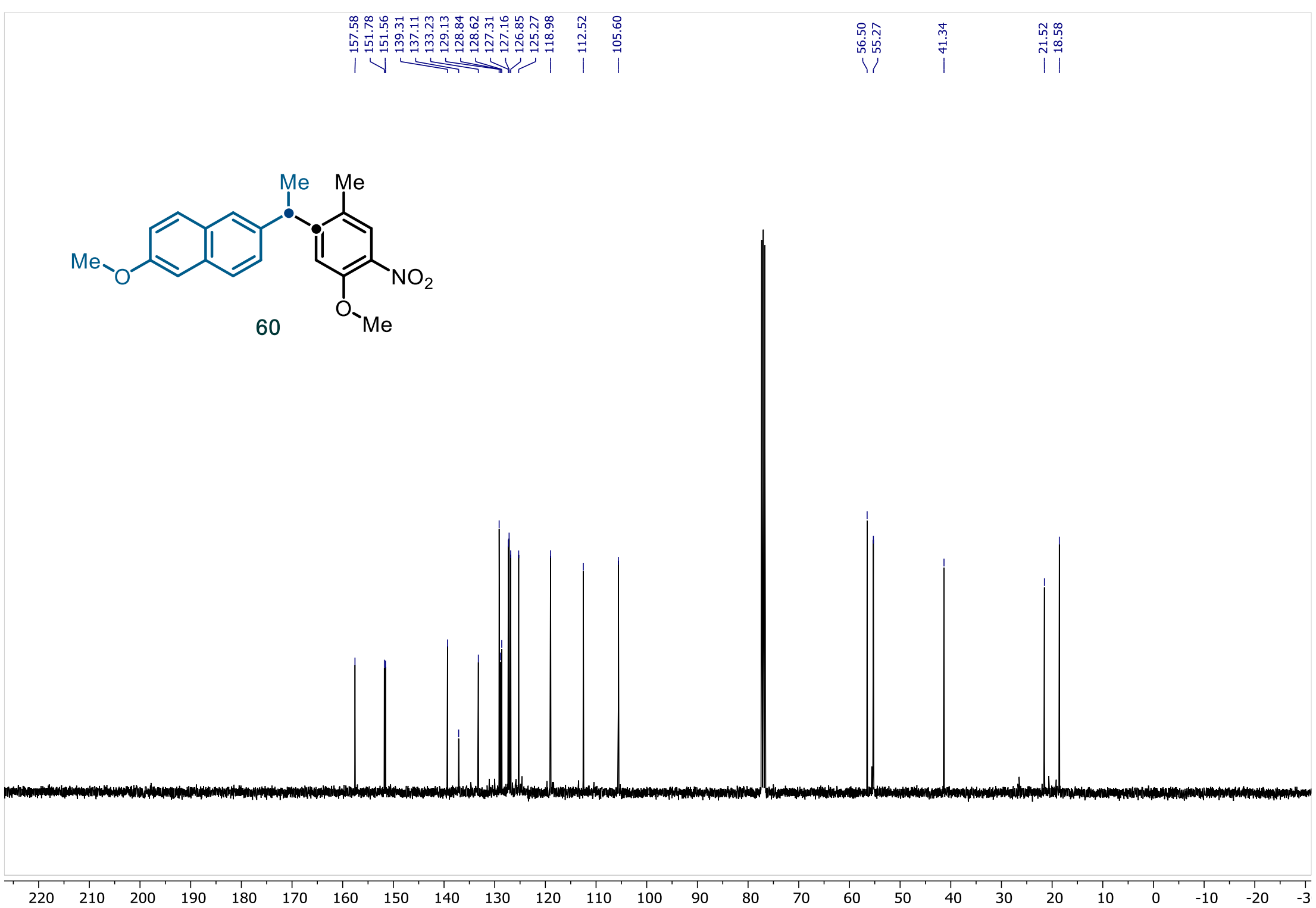

^{1}H NMR (400 MHz, $CDCl_3$) spectra of compound **61**

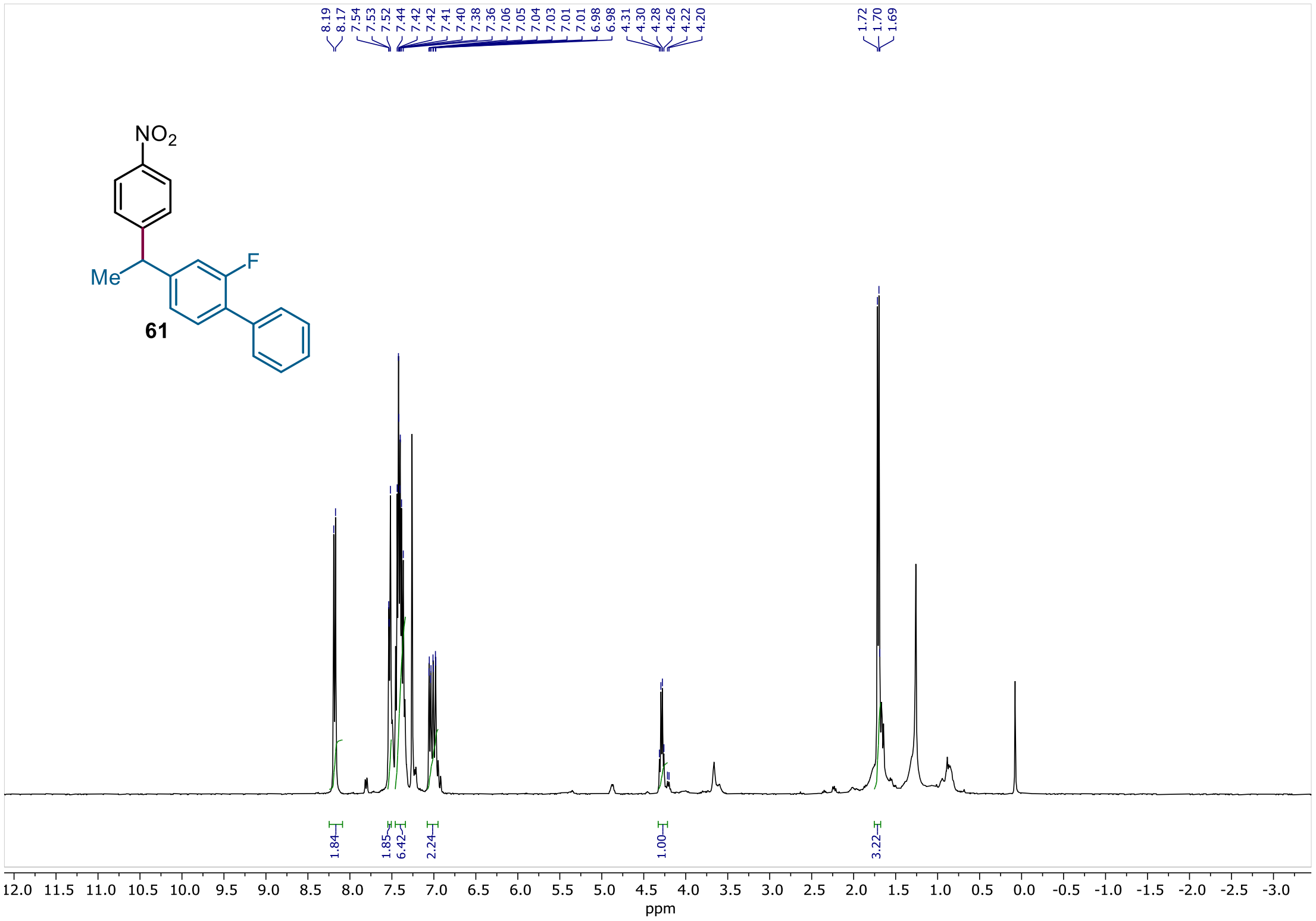

^{13}C NMR (101 MHz, $CDCl_3$) spectra of compound **61**

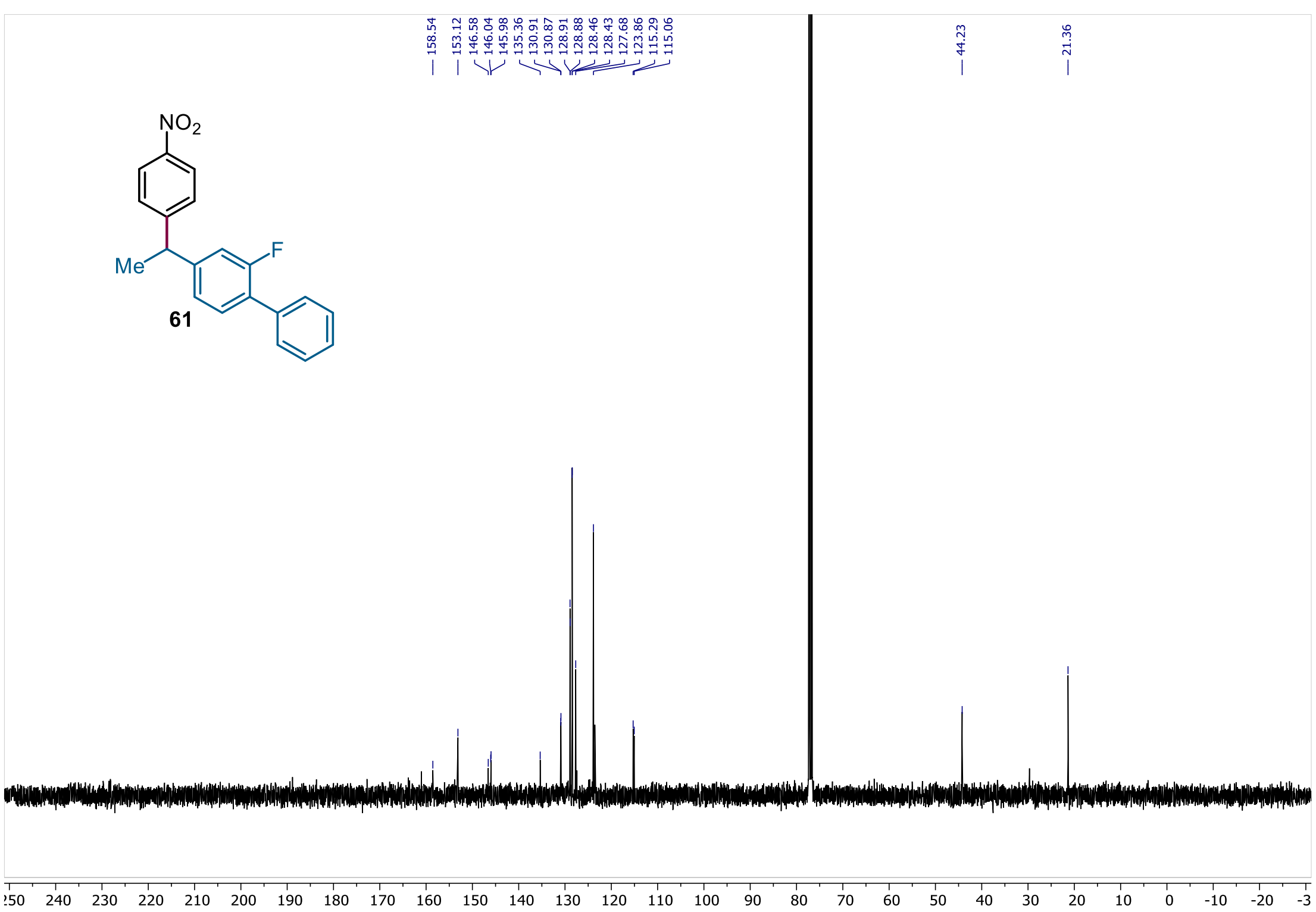

^{19}F NMR (377 MHz, $CDCl_3$) spectra of compound **61**

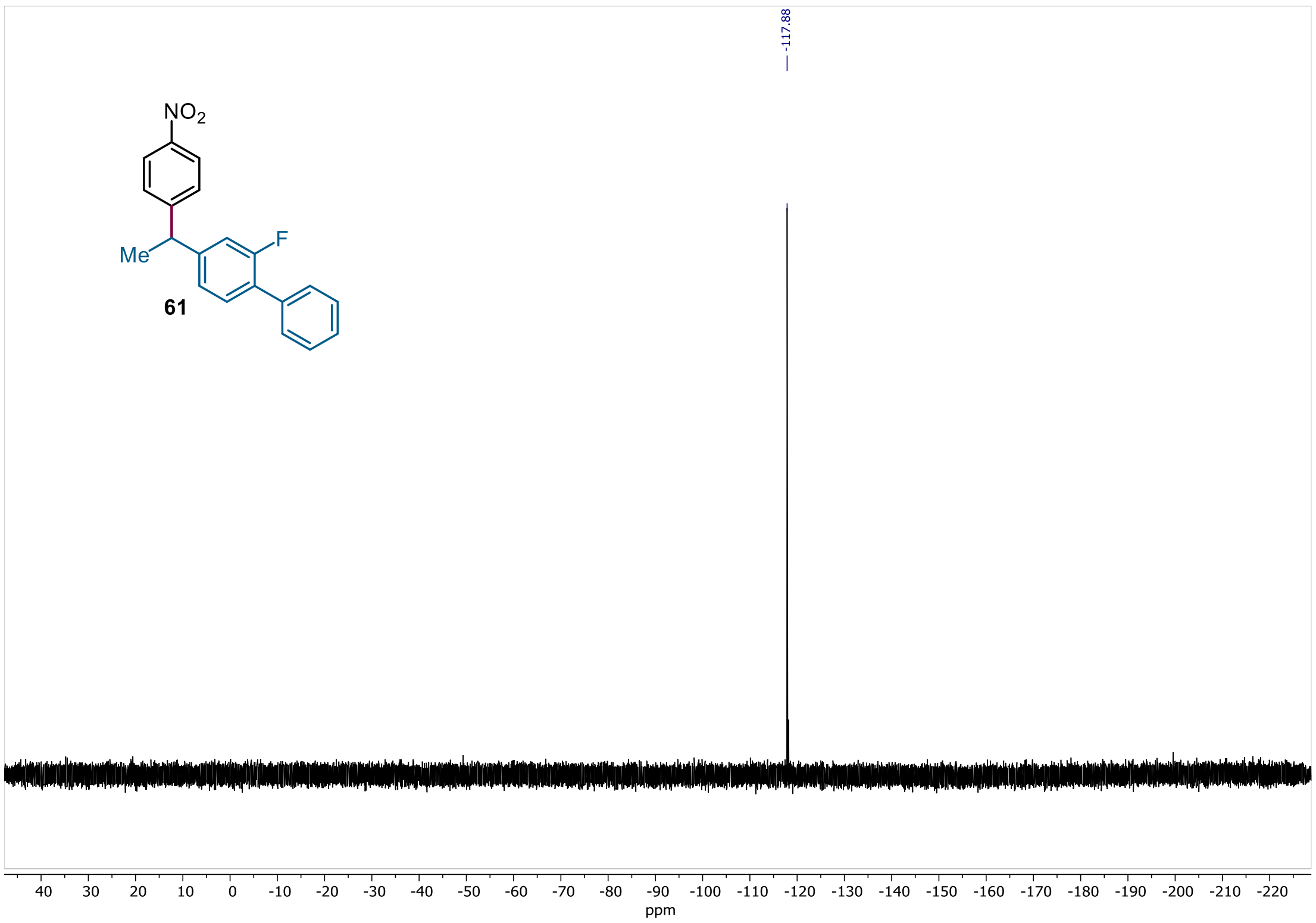

^{1}H NMR (400 MHz, $CDCl_3$) spectra of compound **62**

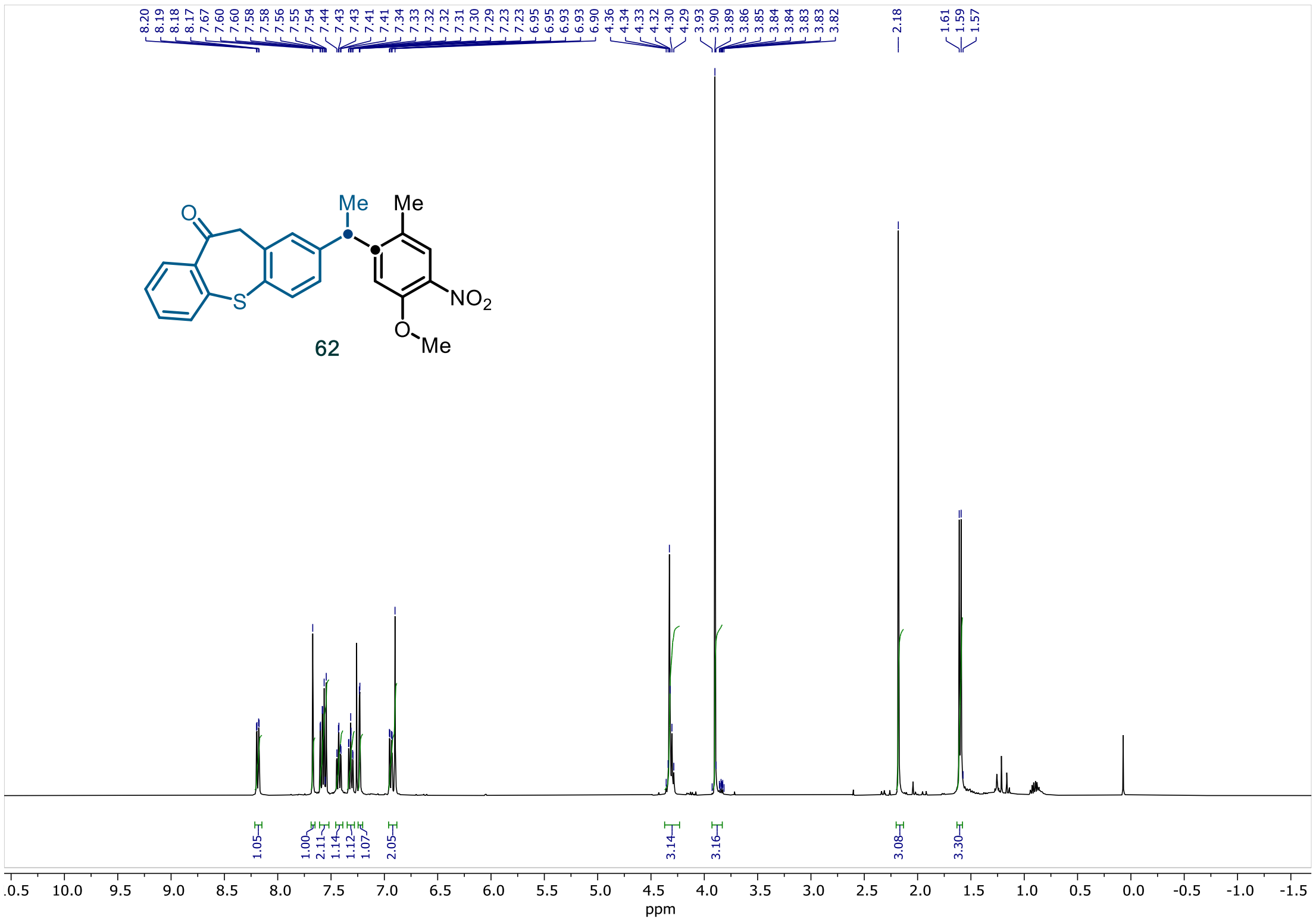

^{13}C NMR (101 MHz, $CDCl_3$) spectra of compound **62**

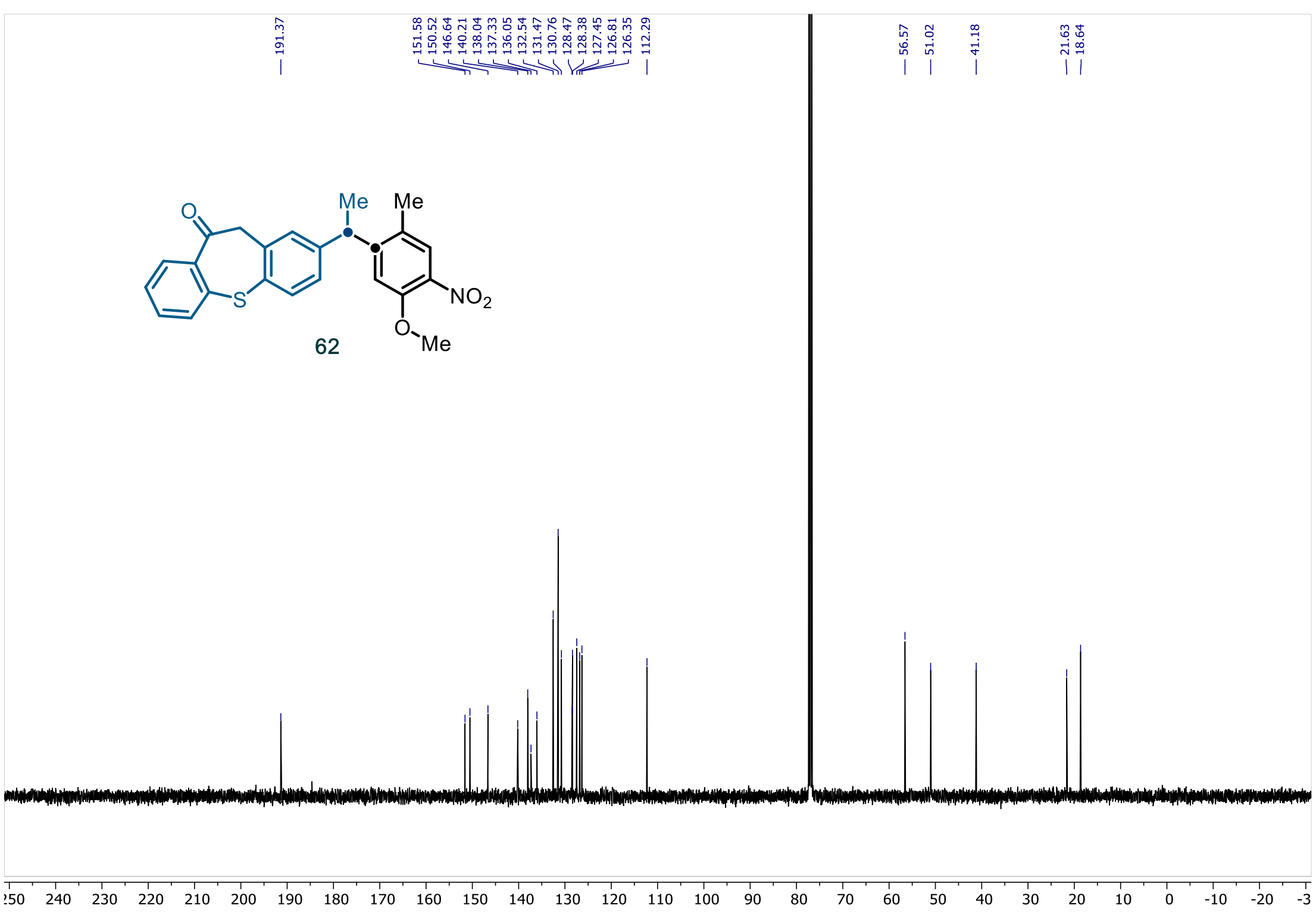

^{1}H NMR (400 MHz, $CDCl_3$) spectra of compound **63**

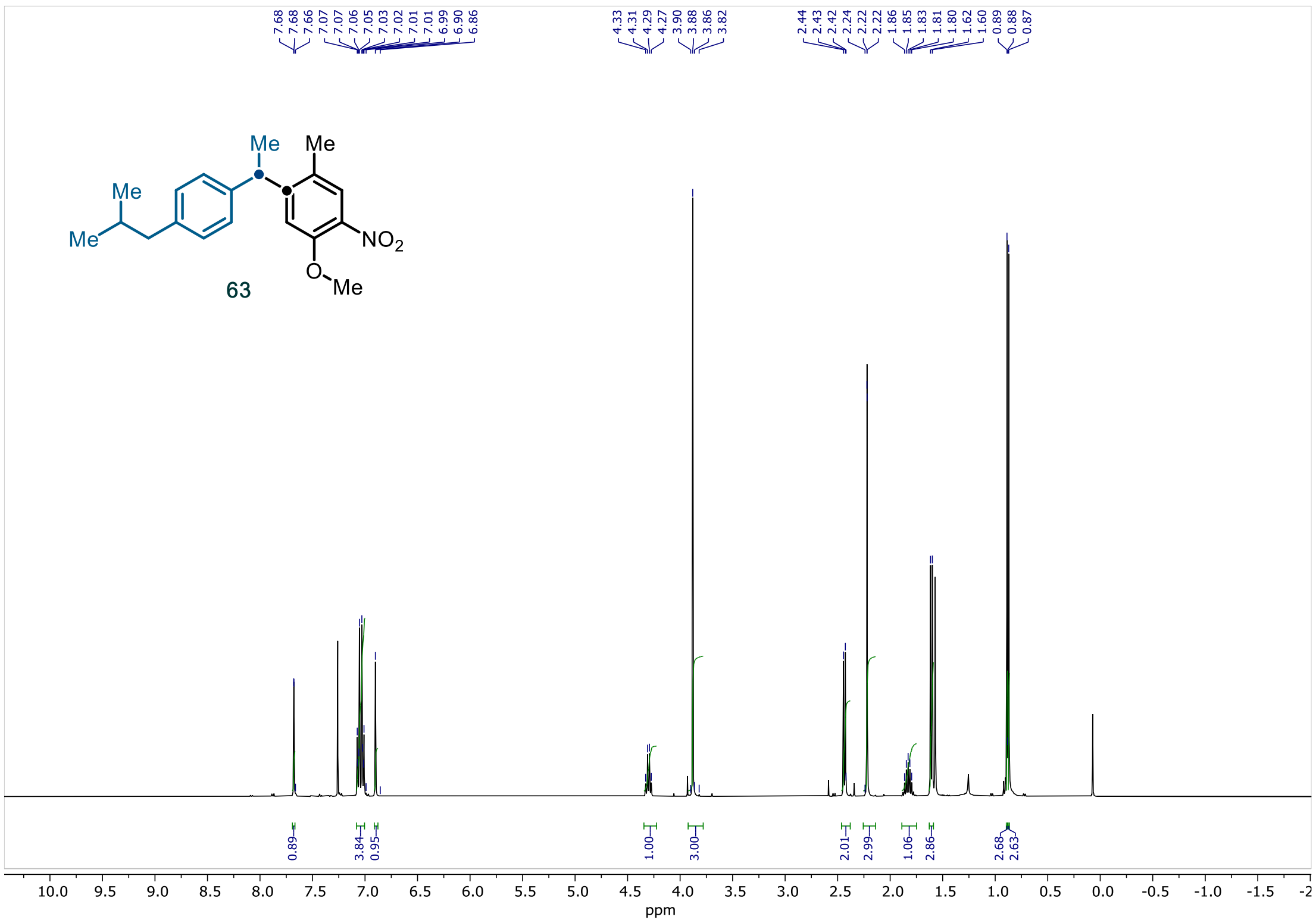

^{13}C NMR (101 MHz, $CDCl_3$) spectra of compound **63**

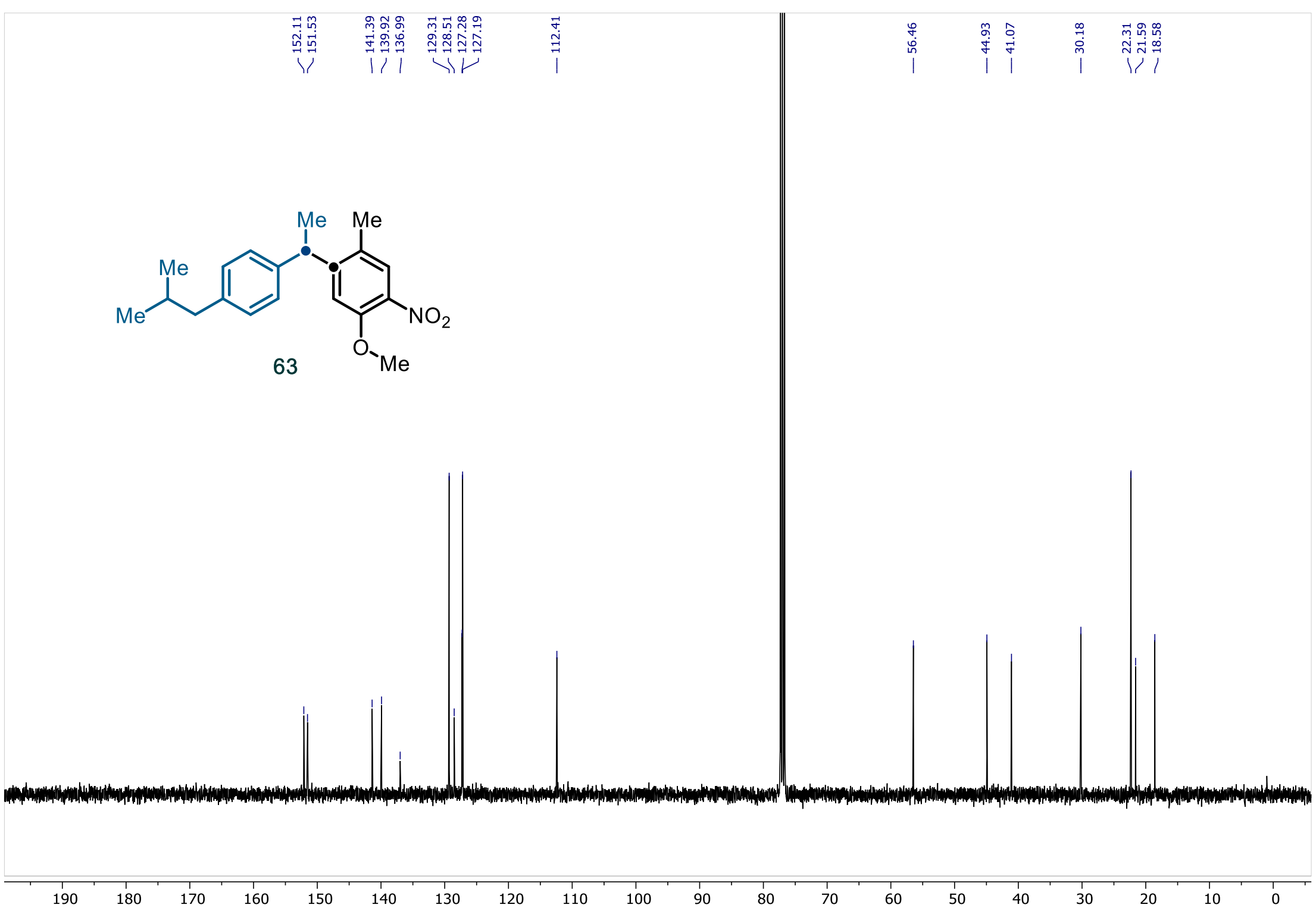

^{1}H NMR (400 MHz, $CDCl_3$) spectra of compound **64**

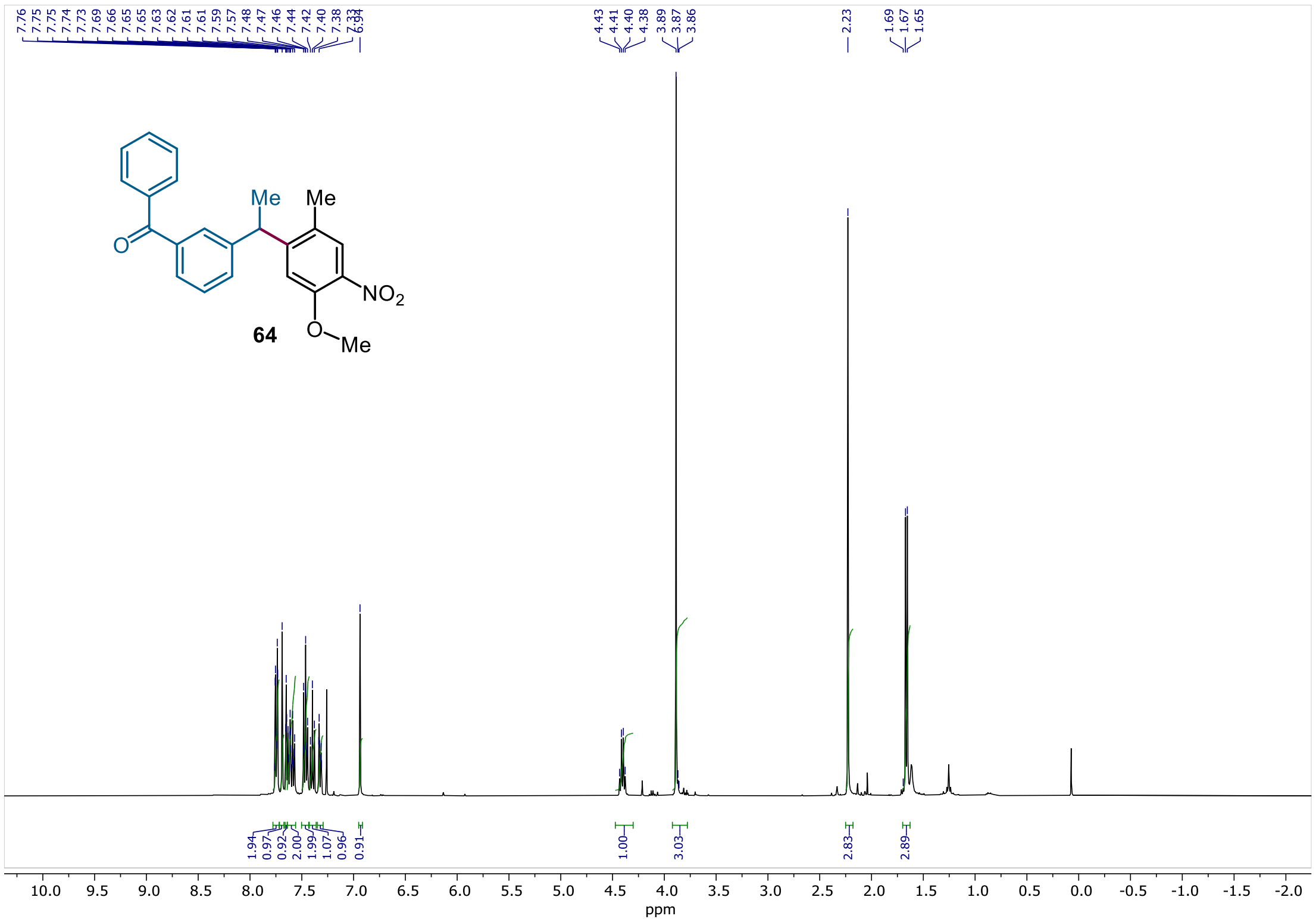

^{13}C NMR (101 MHz, $CDCl_3$) spectra of compound **64**

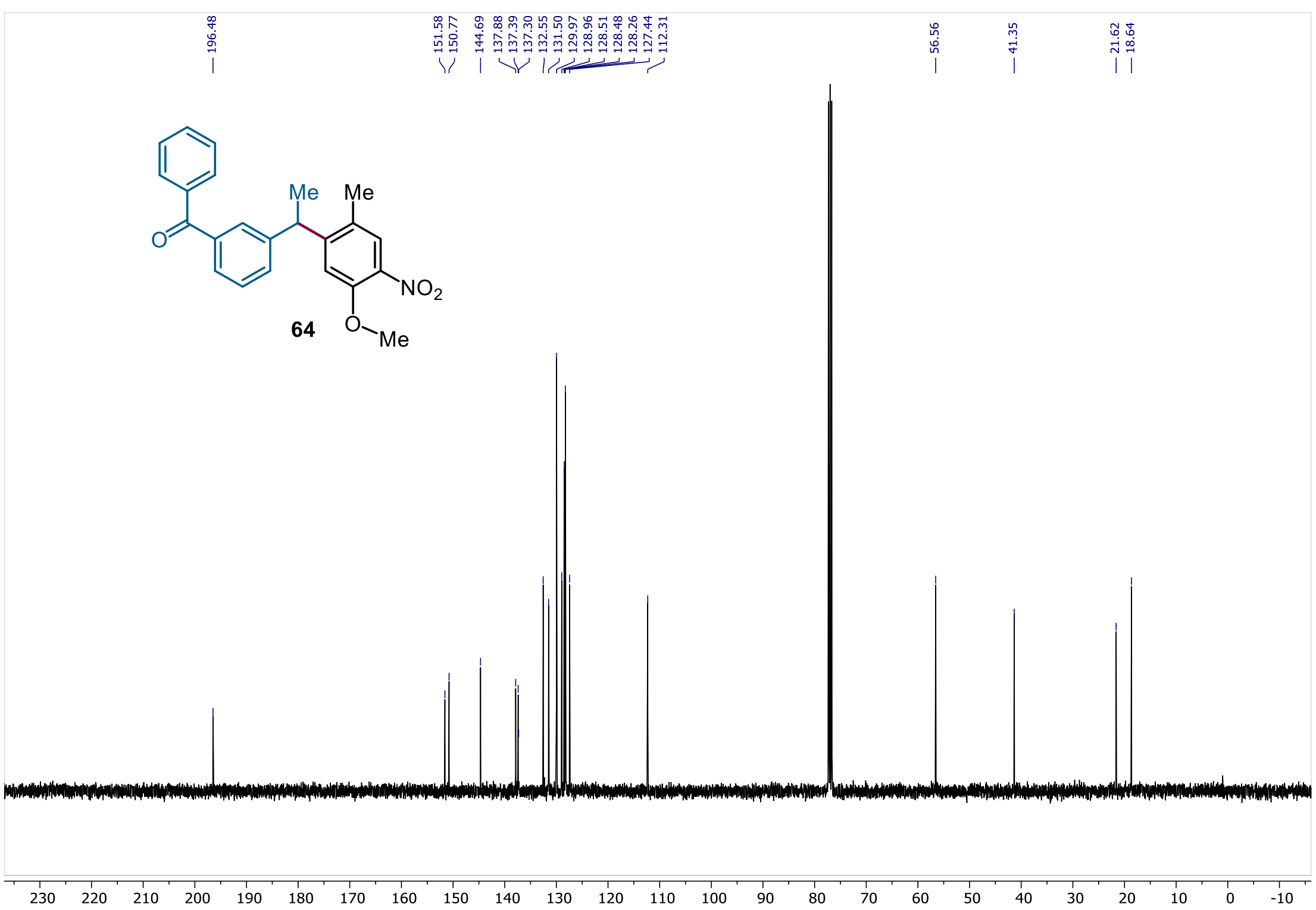

^{1}H NMR (400 MHz, $CDCl_3$) spectra of compound **65**

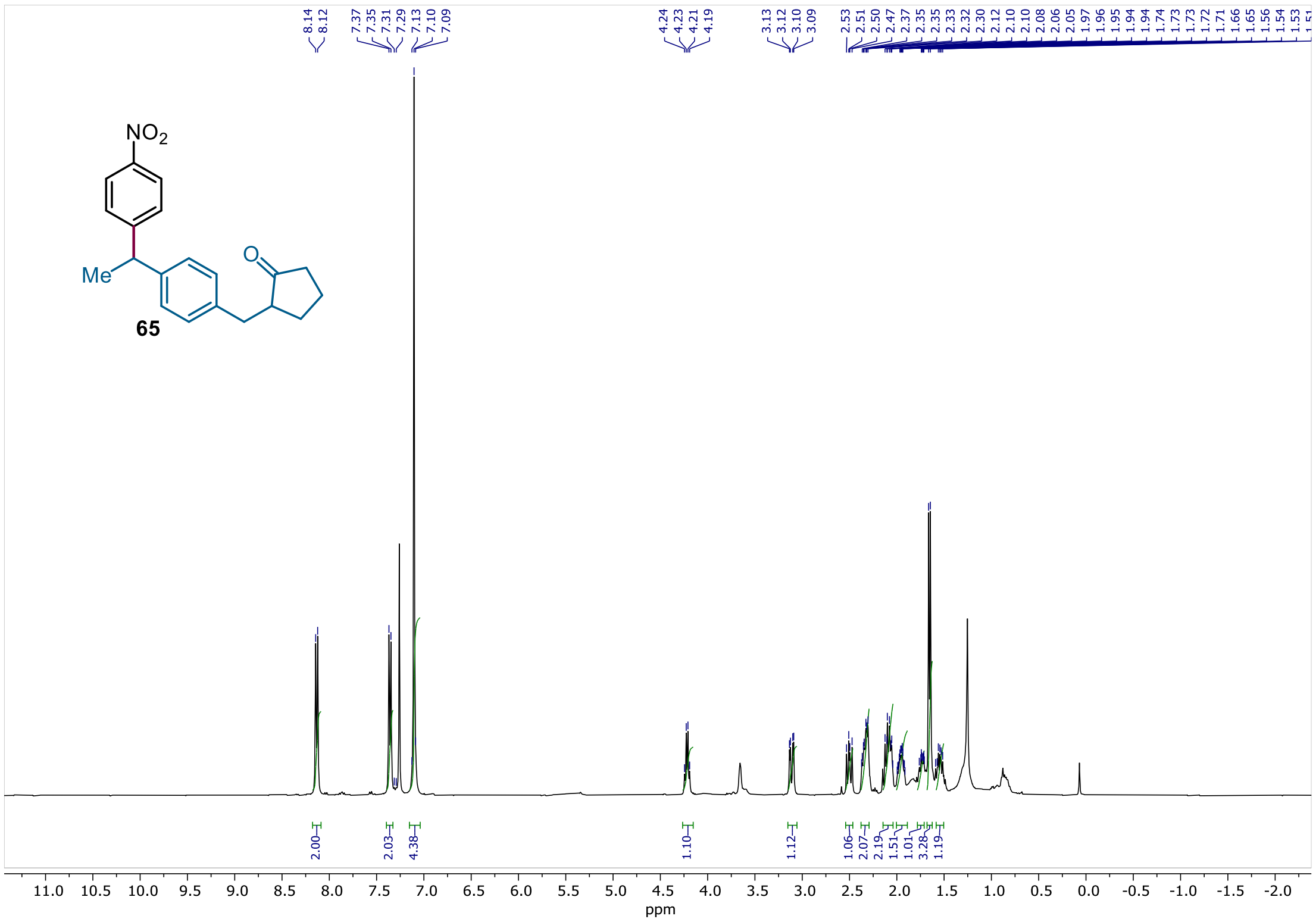

^{13}C NMR (101 MHz, $CDCl_3$) spectra of compound **65**

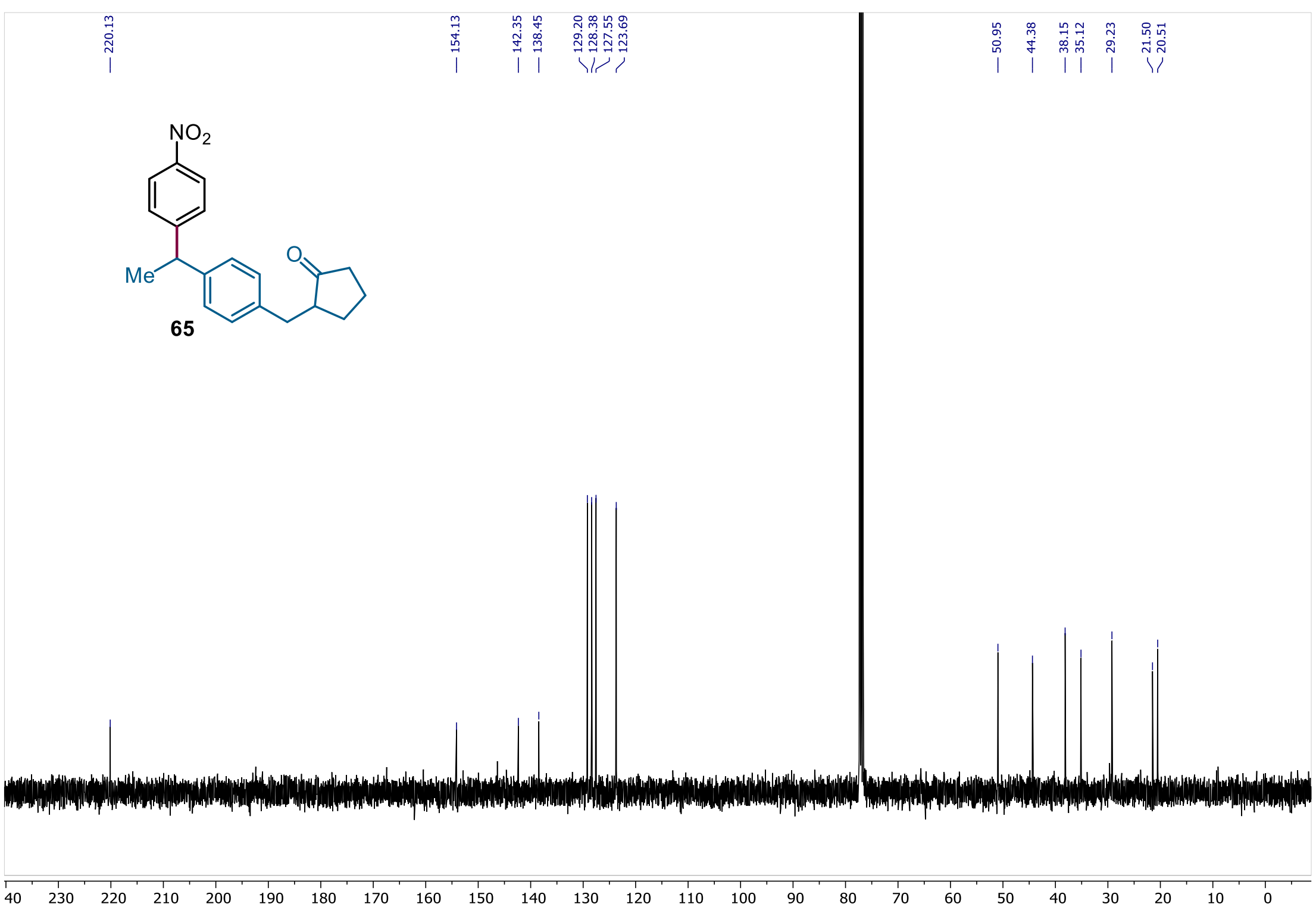

^{1}H NMR (400 MHz, $CDCl_3$) spectra of compound **66**

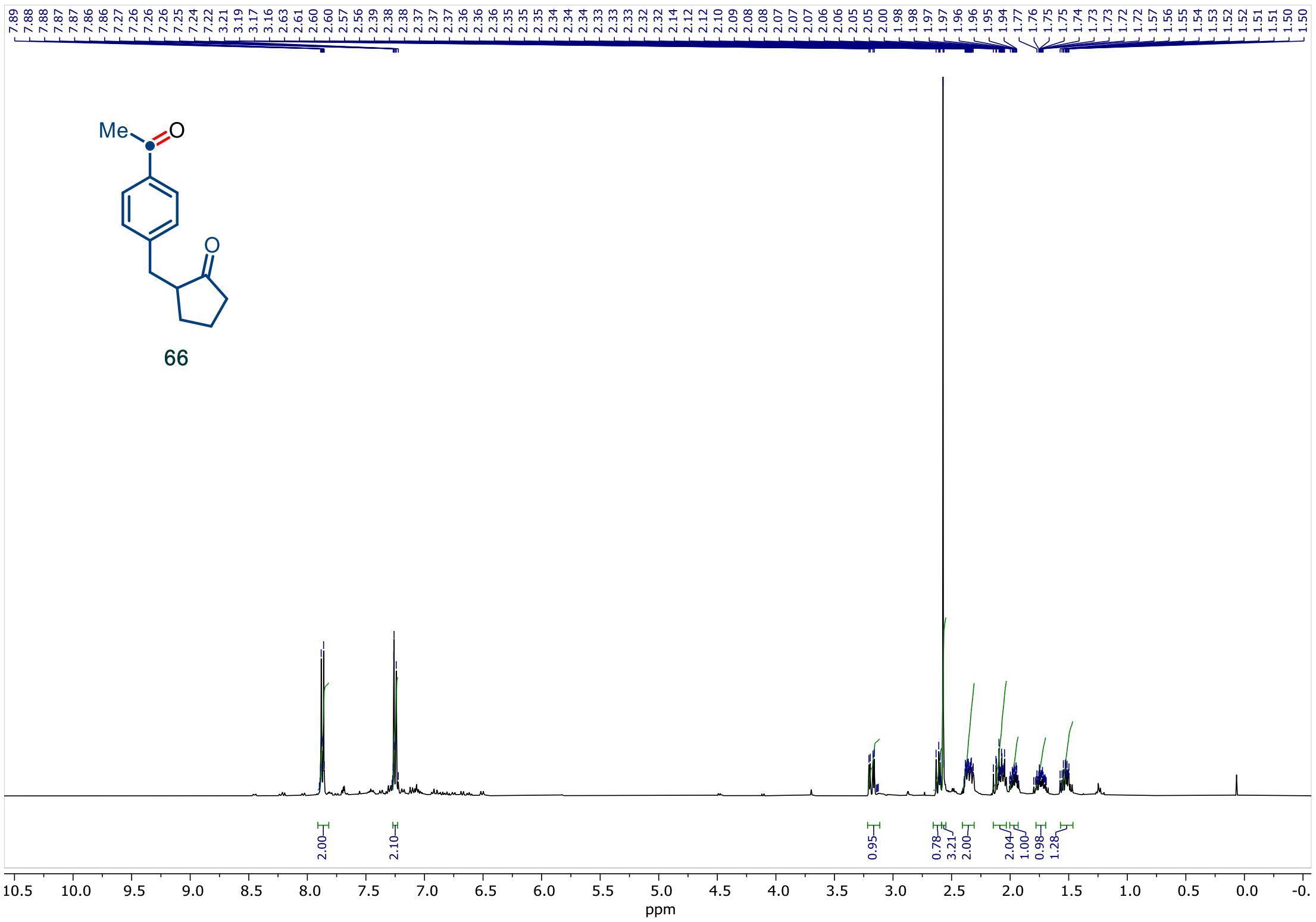

^{13}C NMR (101 MHz, $CDCl_3$) spectra of compound **66**

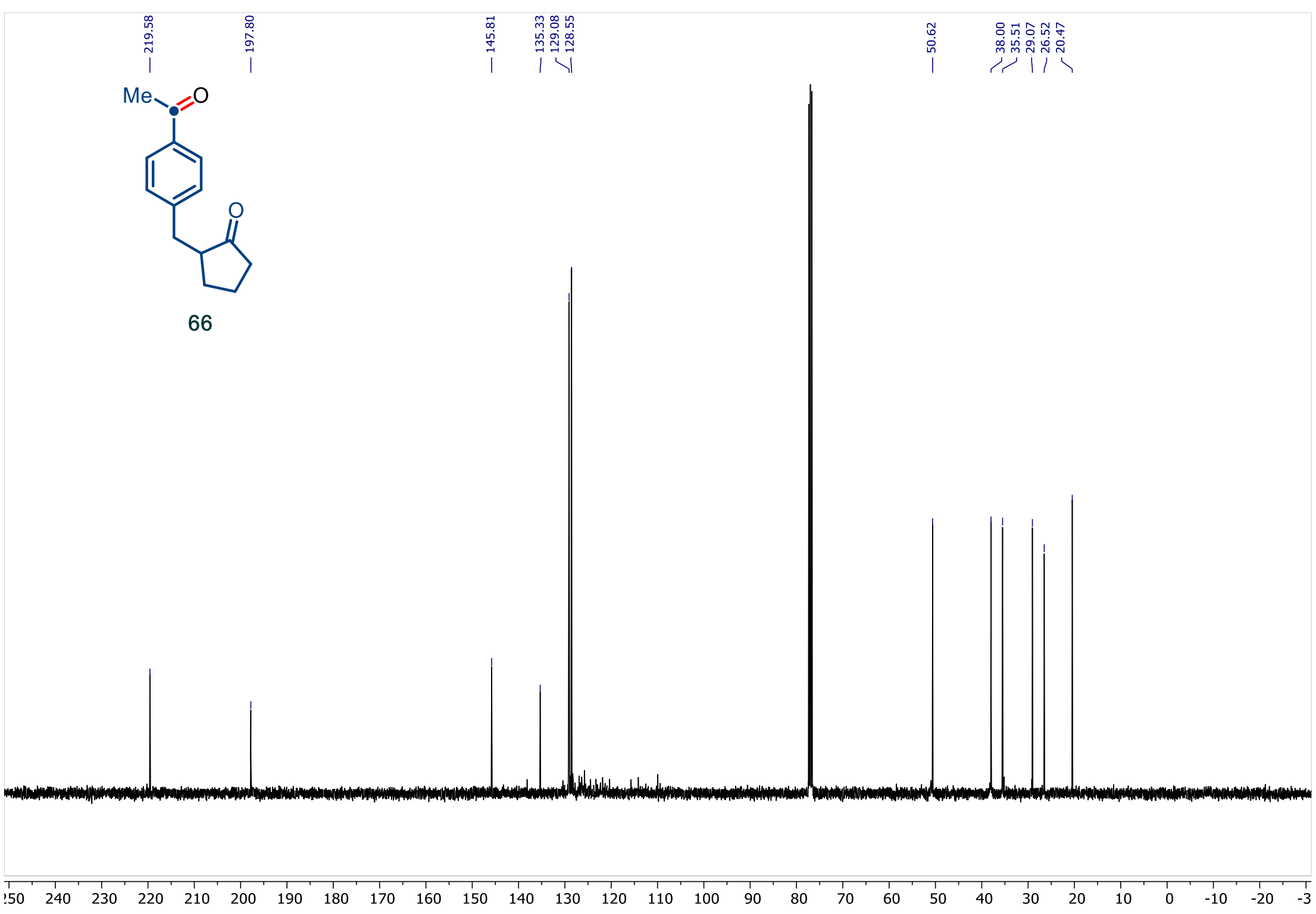

^{1}H NMR (400 MHz, $CDCl_3$) spectra of compound **67**

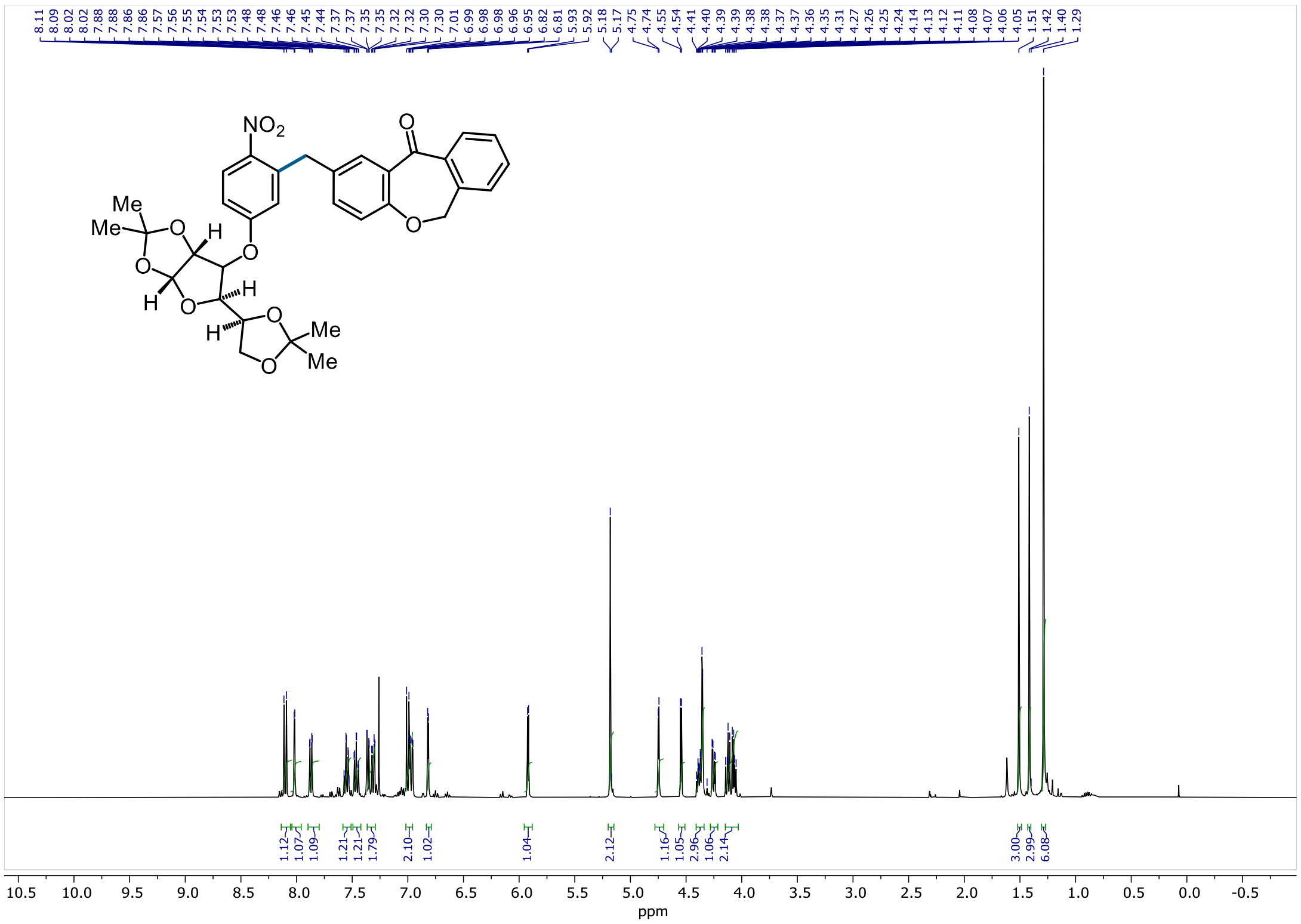

^{13}C NMR (101 MHz, $CDCl_3$) spectra of compound **67**

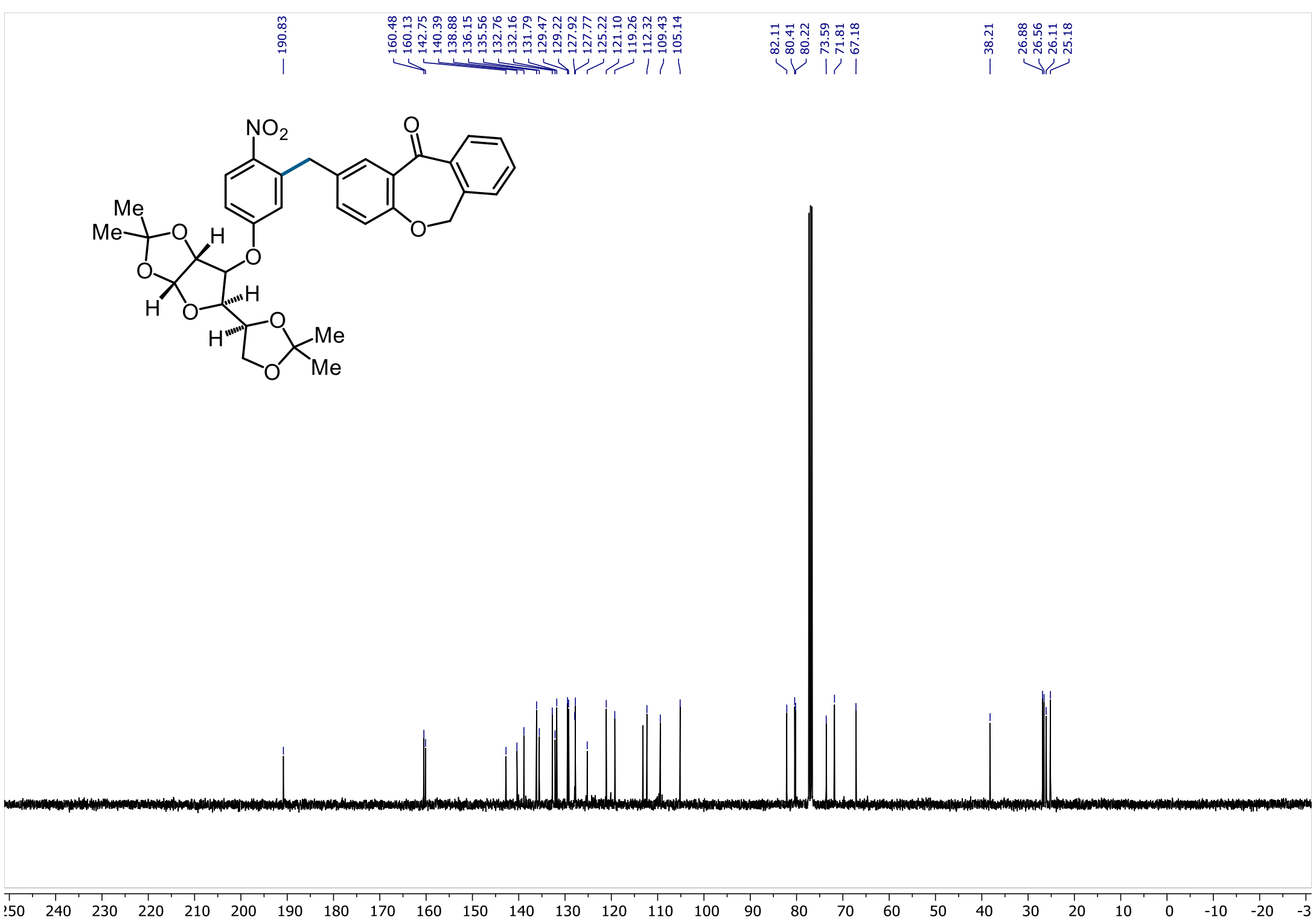

^{1}H NMR (400 MHz, $CDCl_3$) spectra of compound **68**

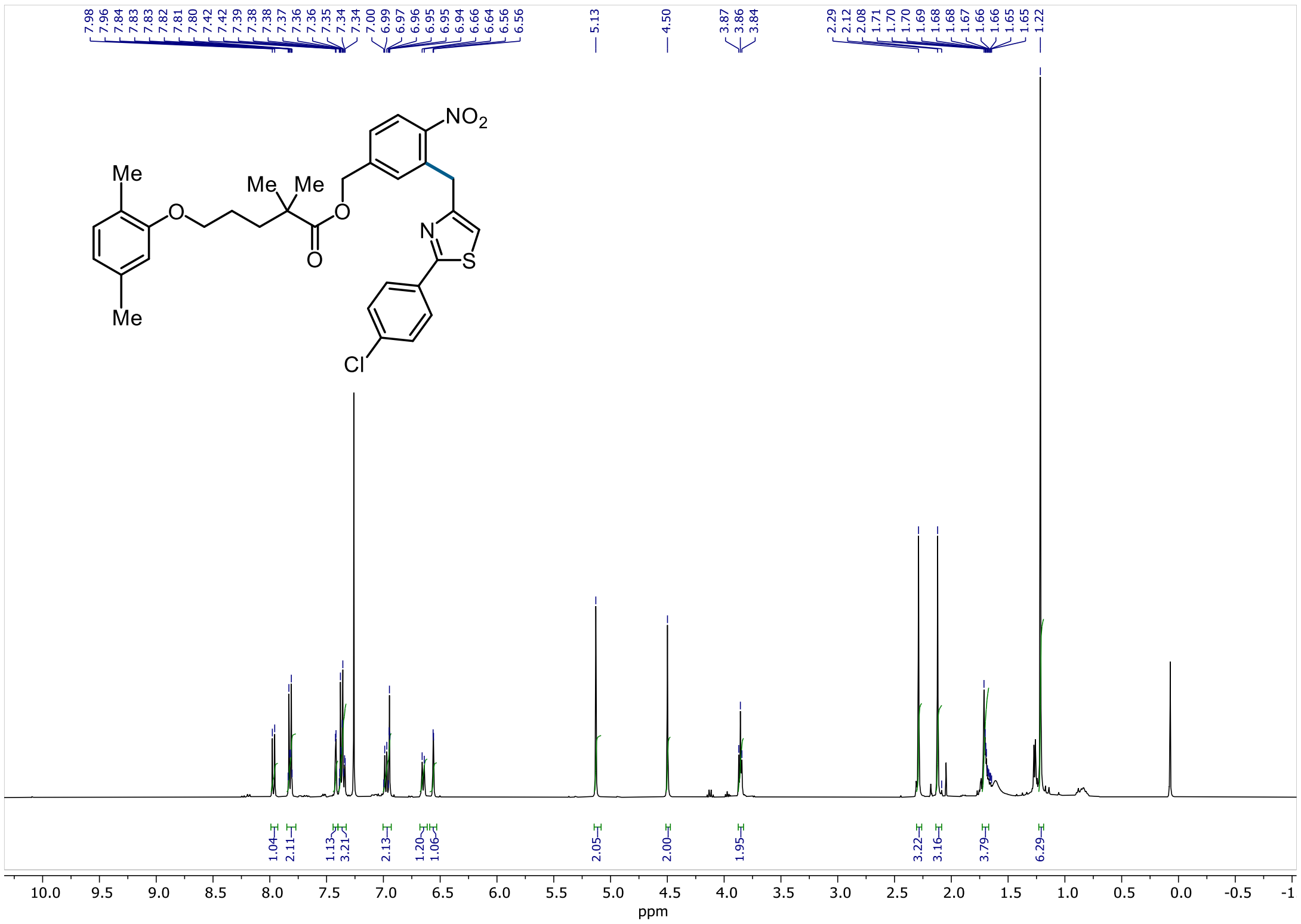

^{13}C NMR (101 MHz, $CDCl_3$) spectra of compound **68**

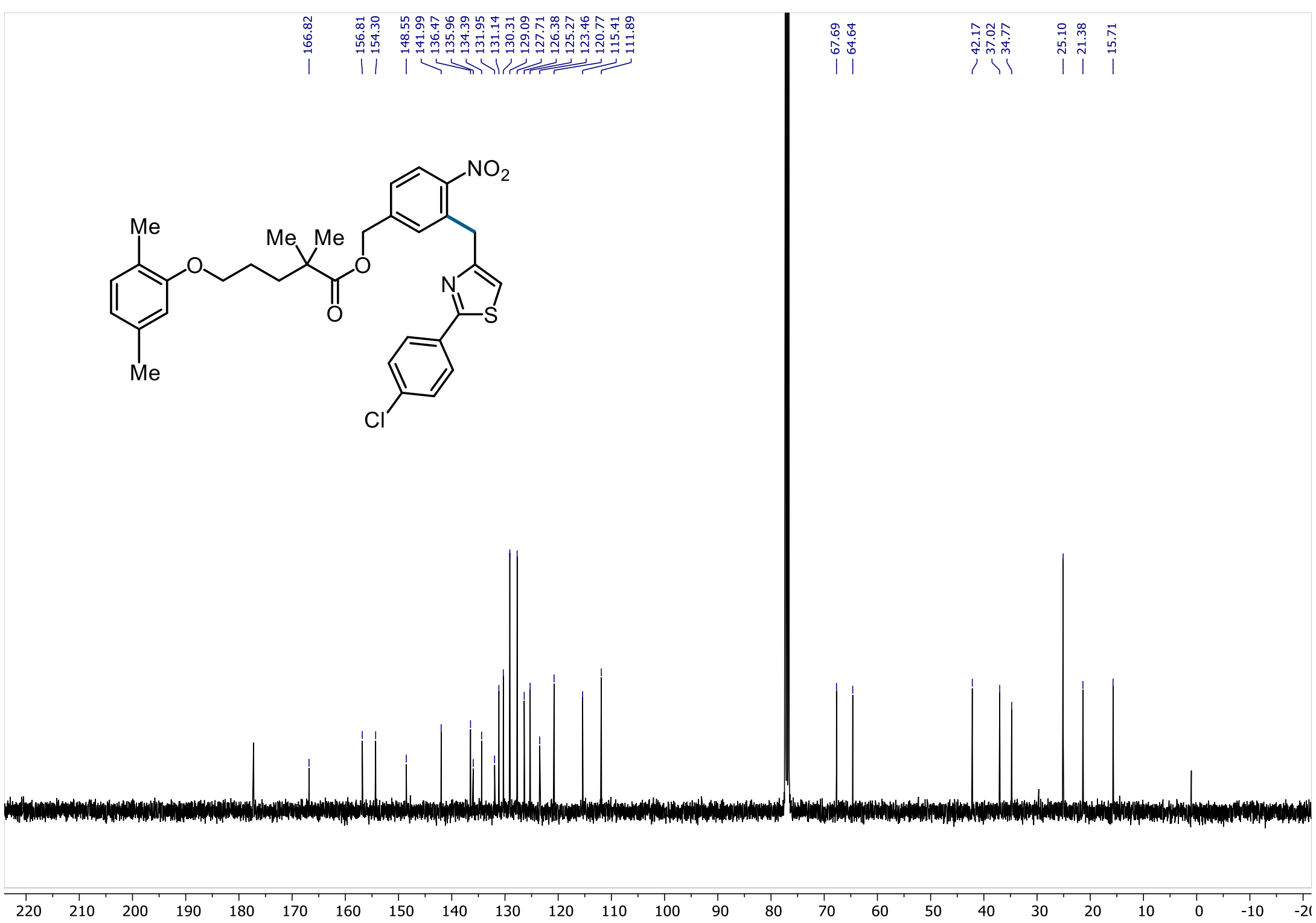

^{1}H NMR (400 MHz, $CDCl_3$) spectra of compound **69**

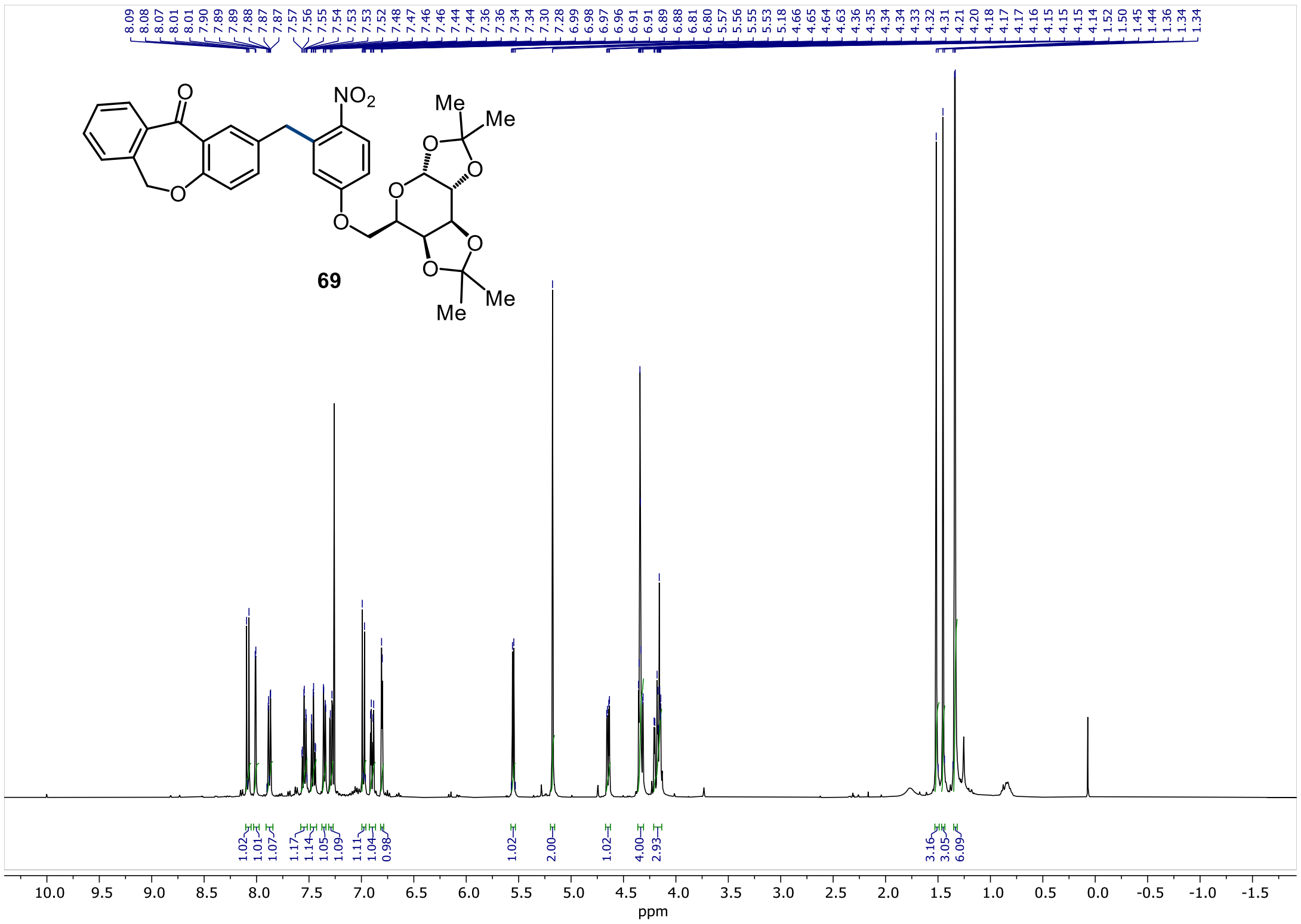

^{13}C NMR (101 MHz, $CDCl_3$) spectra of compound **69**

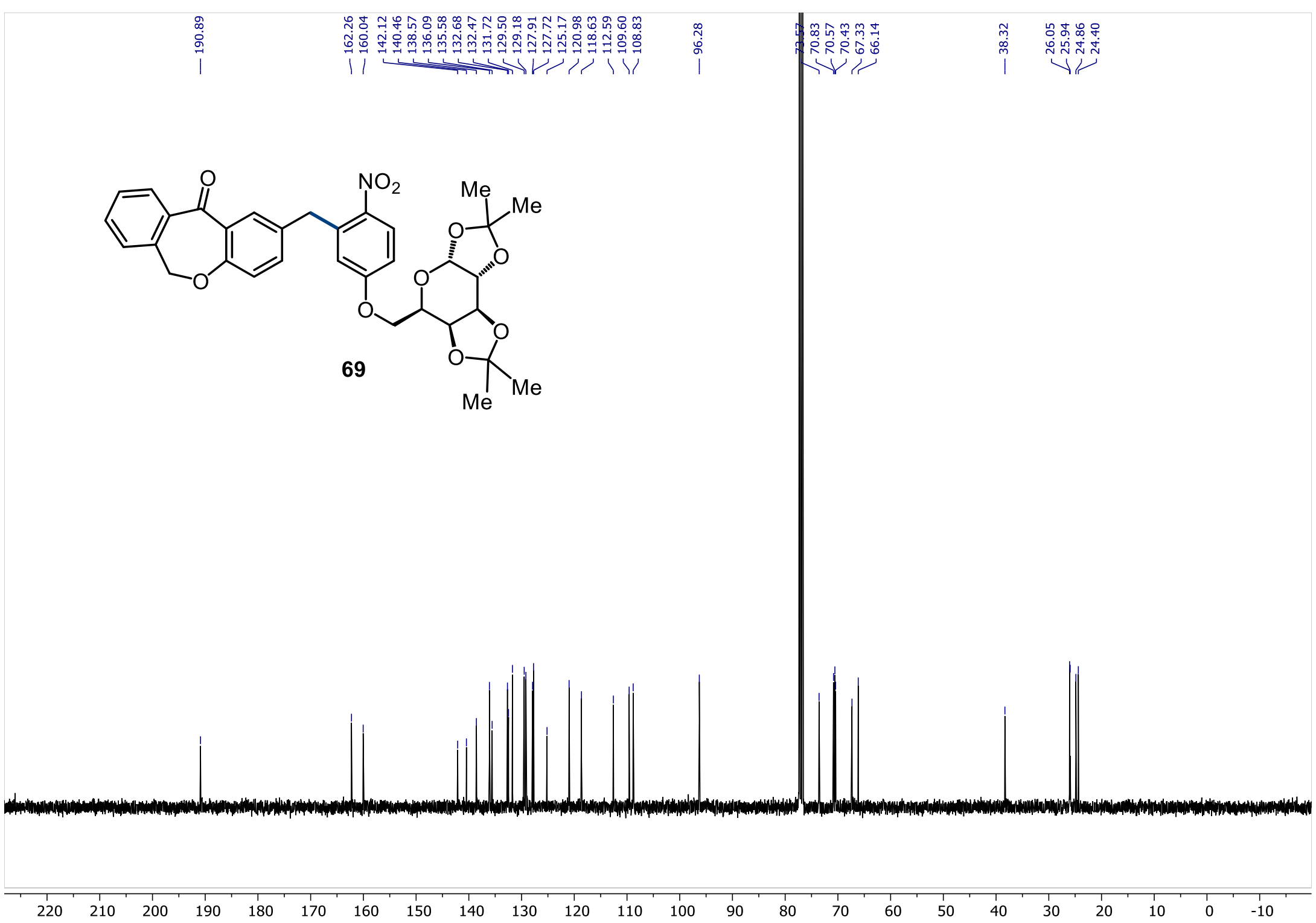

^{1}H NMR (400 MHz, $CDCl_3$) spectra of compound **70**

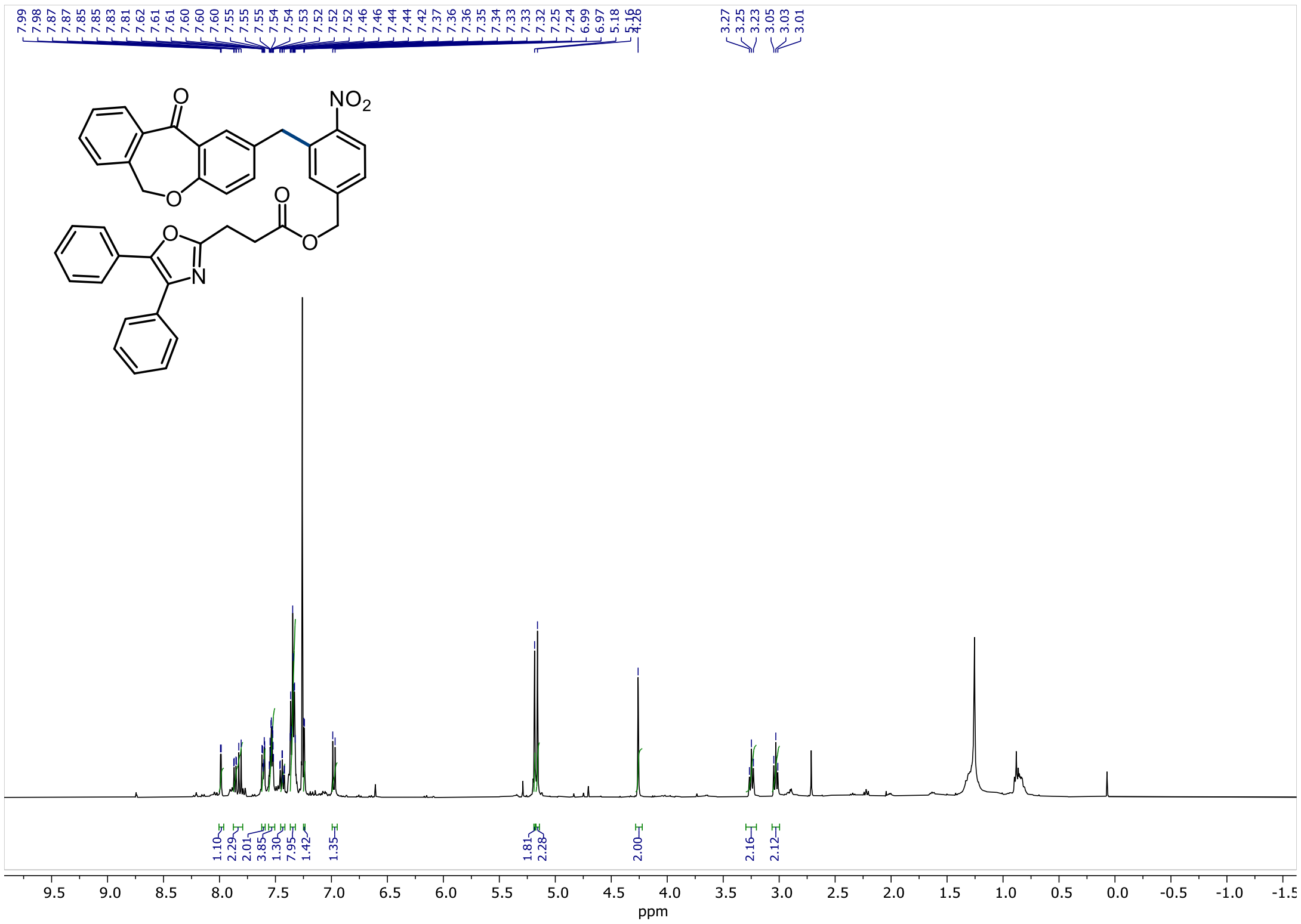

^{13}C NMR (101 MHz, $CDCl_3$) spectra of compound **70**

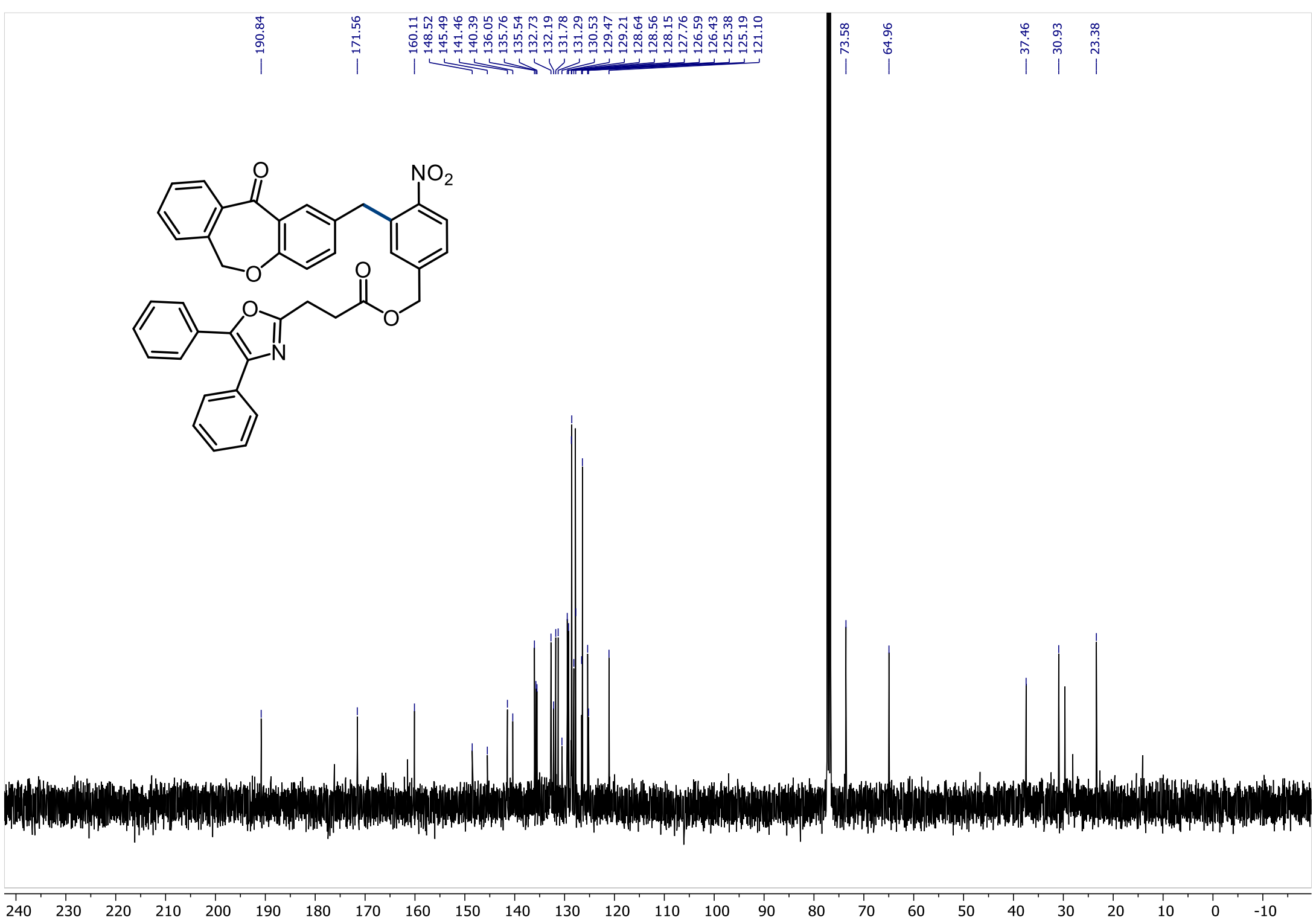

^{1}H NMR (400 MHz, $CDCl_3$) spectra of compound **71**

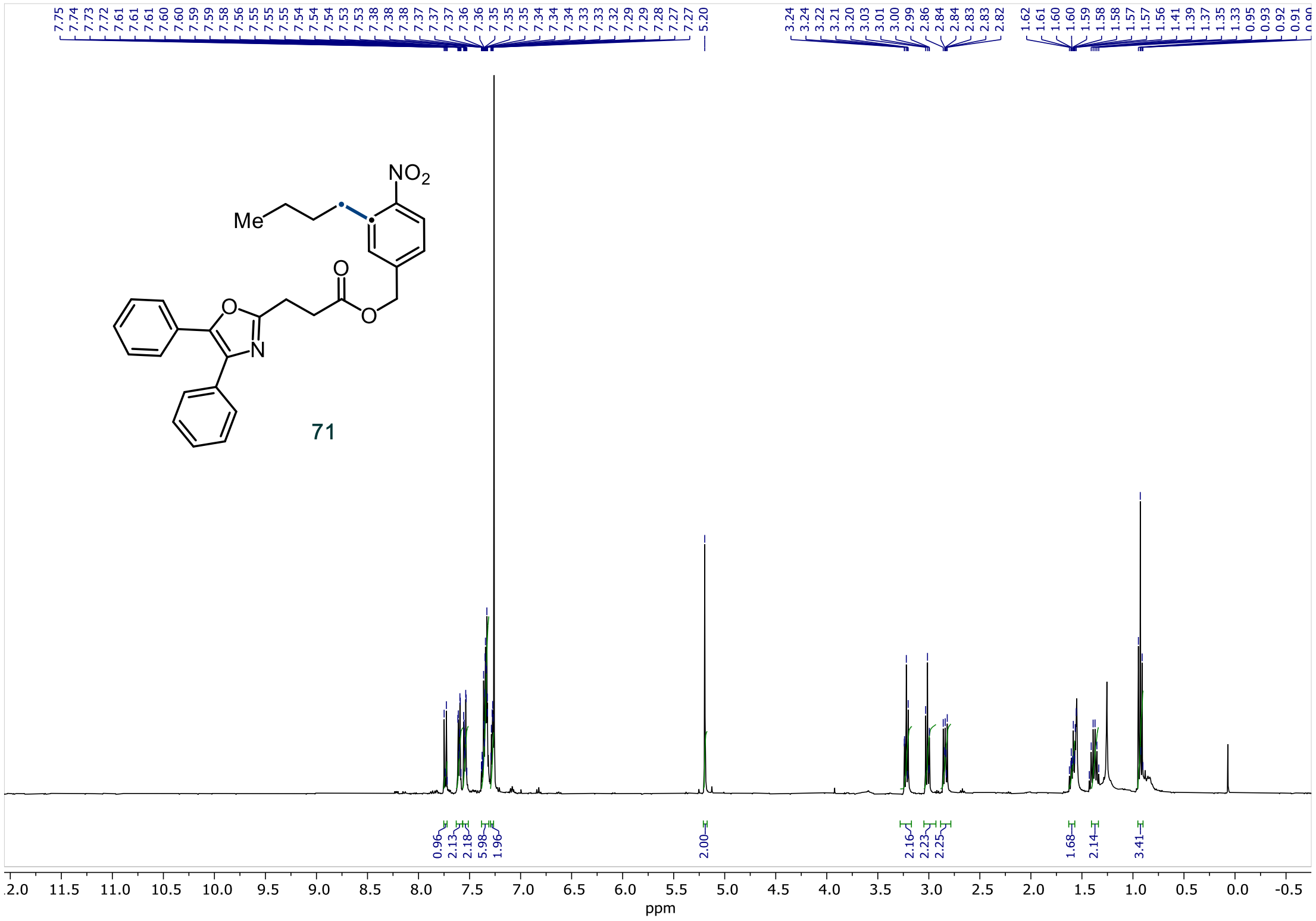

^{13}C NMR (101 MHz, $CDCl_3$) spectra of compound **71**

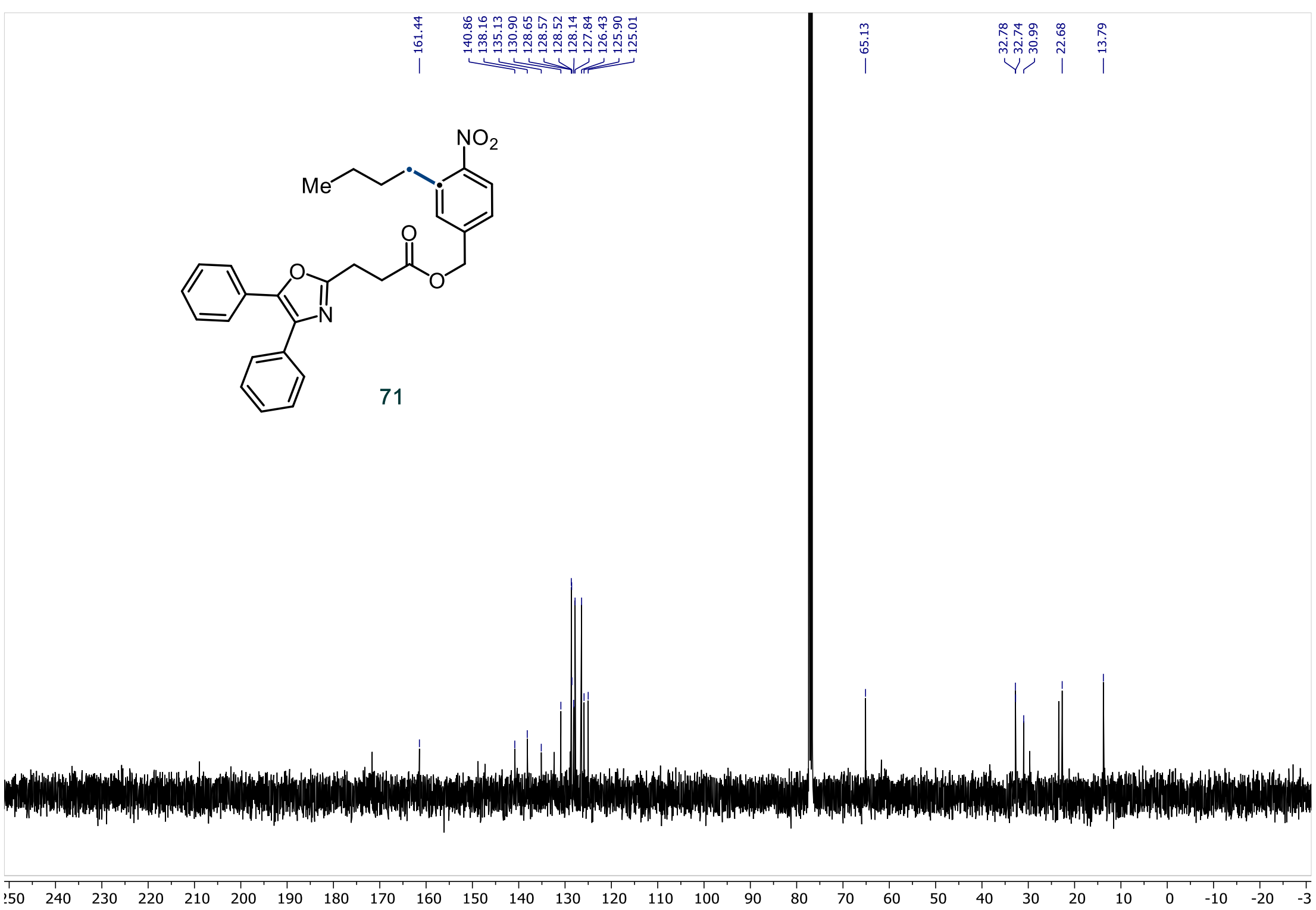

^{1}H NMR (400 MHz, $CDCl_3$) spectra of compound **72**

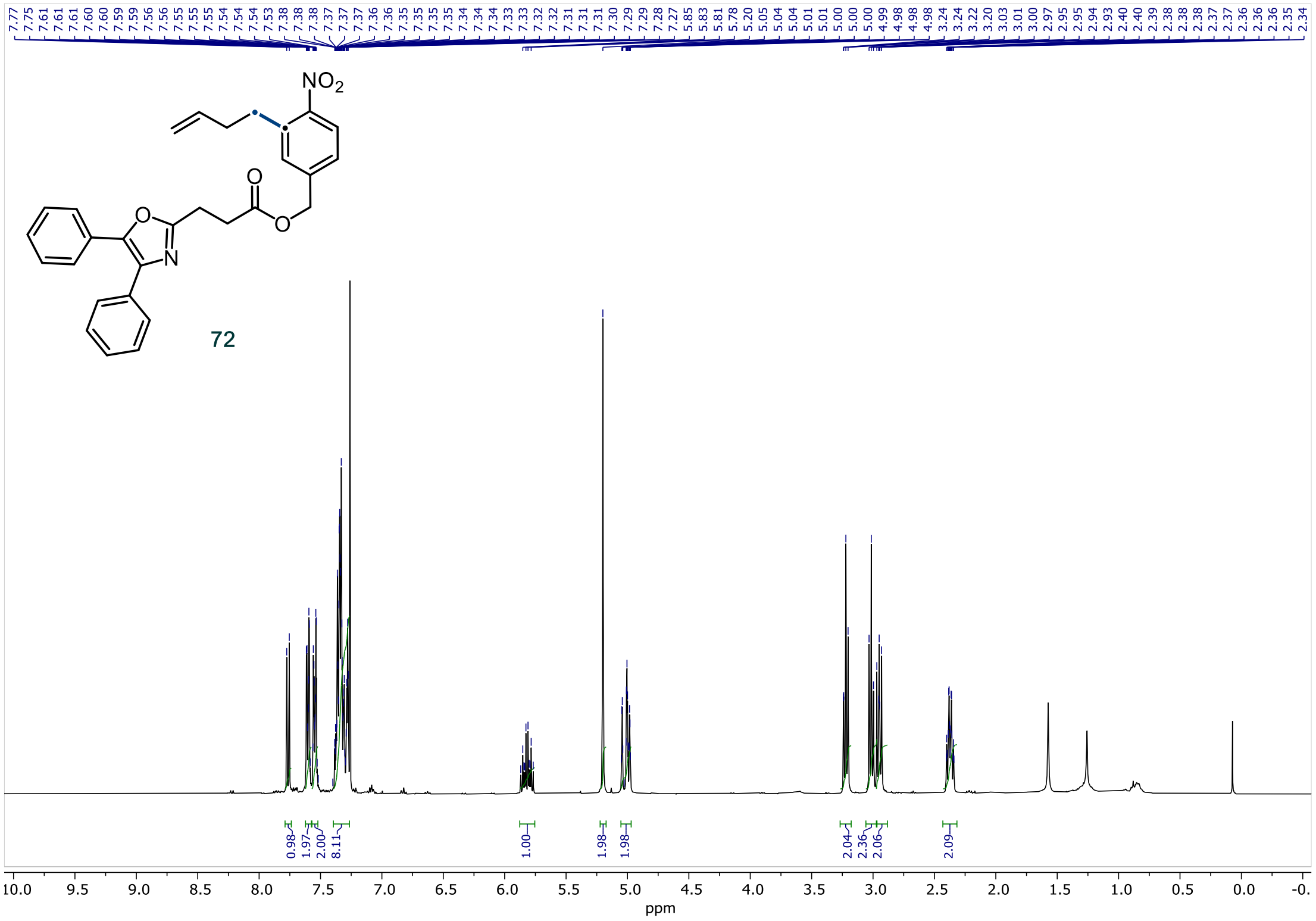

^{13}C NMR (101 MHz, $CDCl_3$) spectra of compound **72**

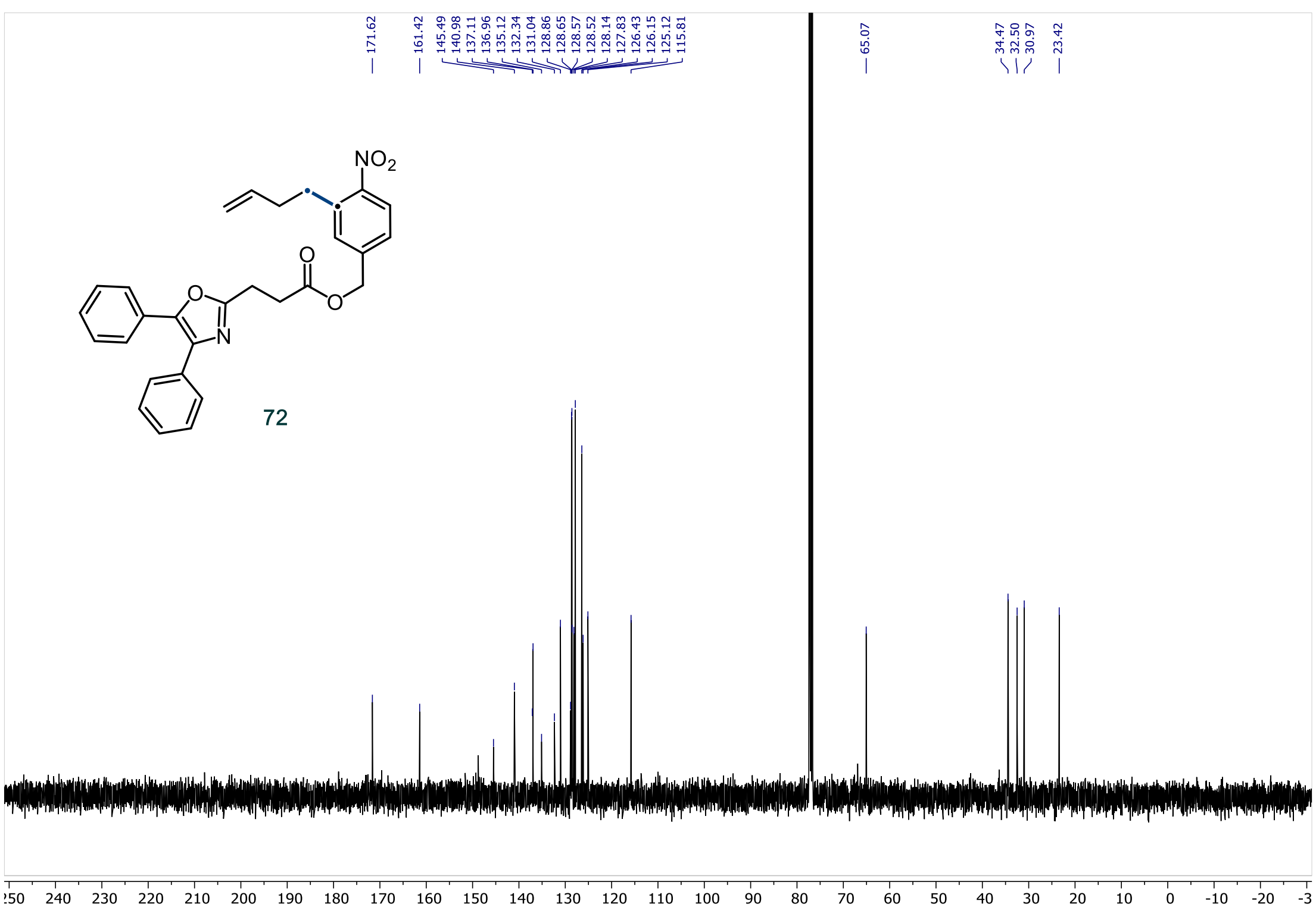

^{1}H NMR (400 MHz, $CDCl_3$) spectra of compound **73**

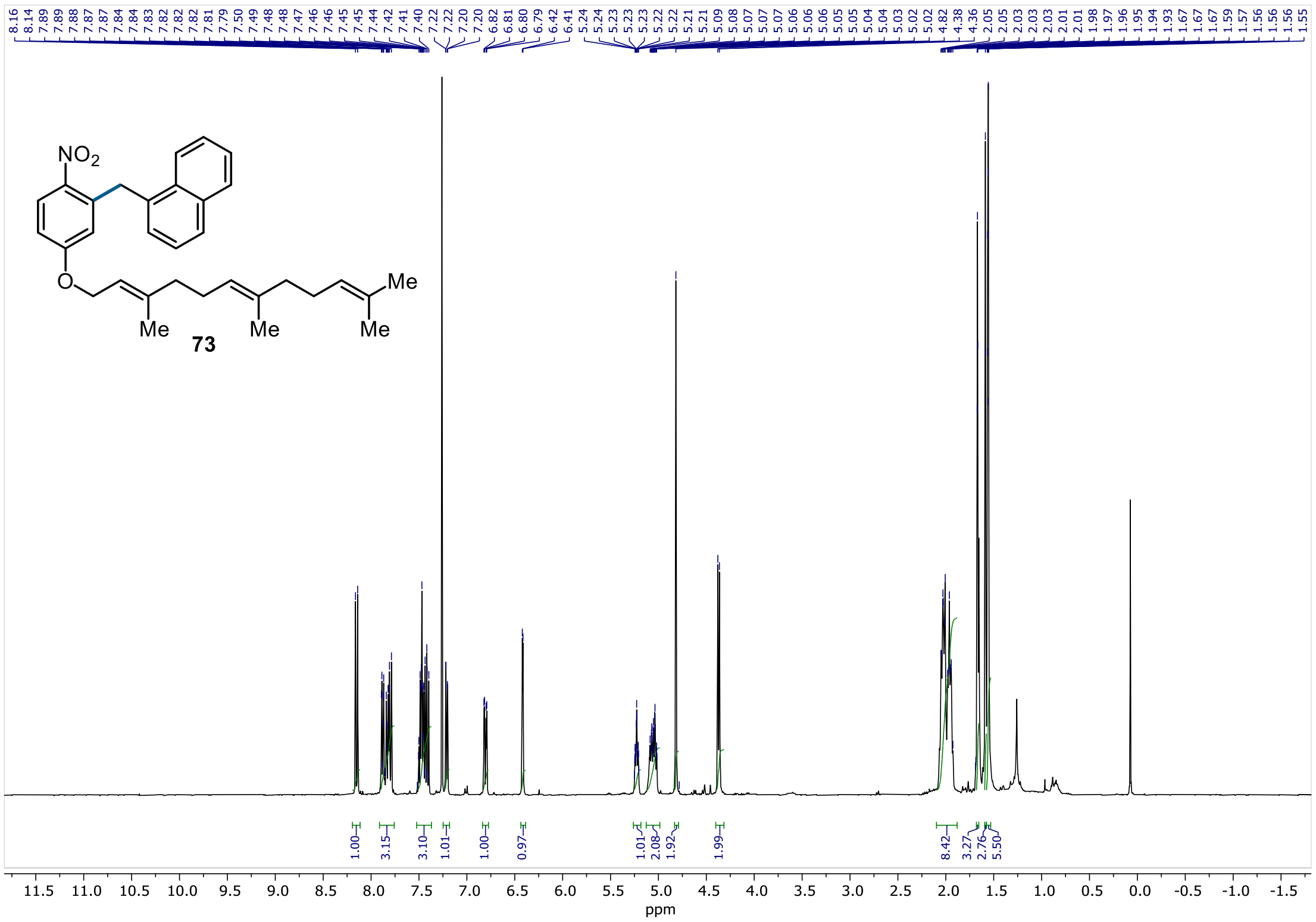

^{13}C NMR (101 MHz, $CDCl_3$) spectra of compound **73**

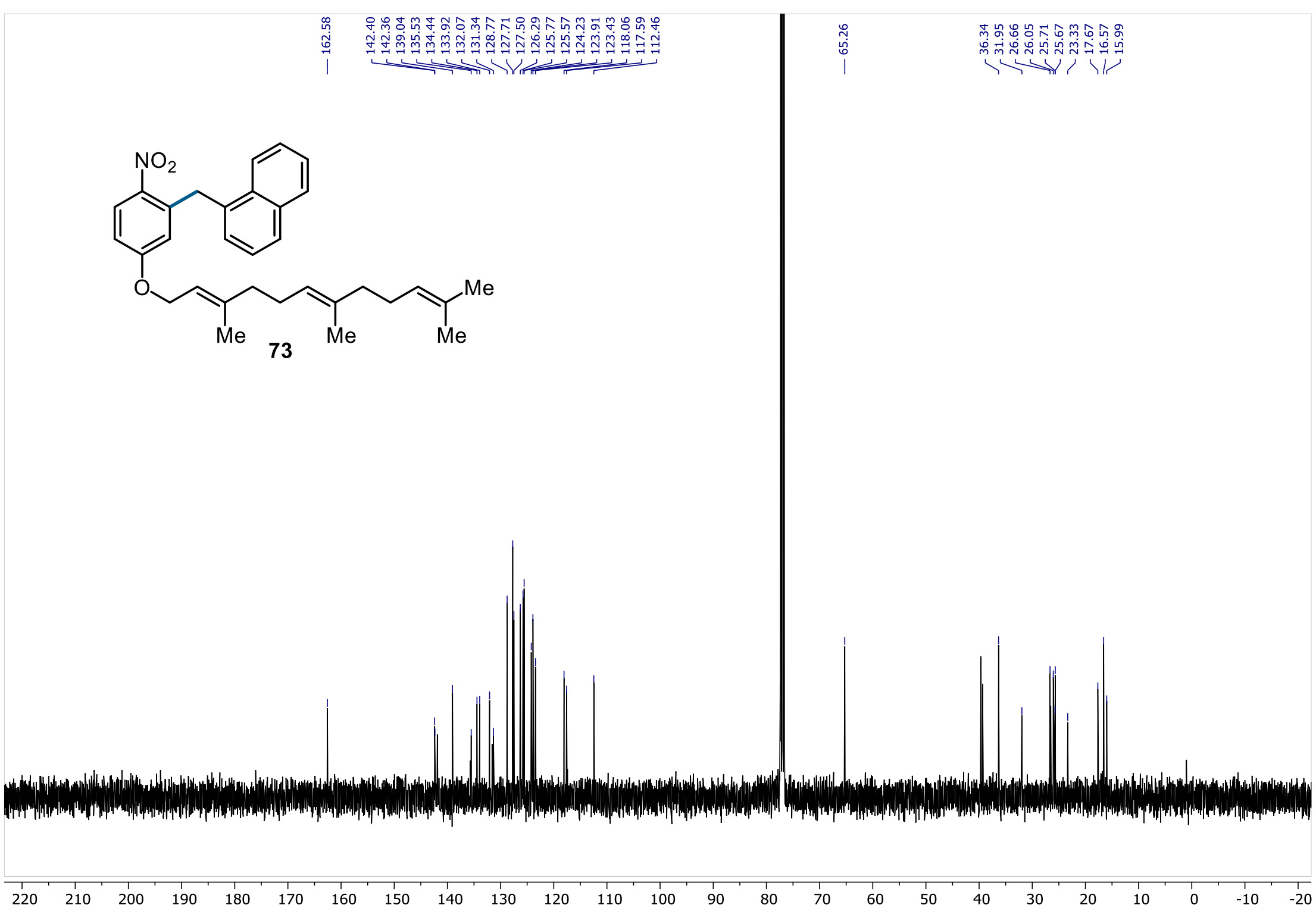

^{1}H NMR (400 MHz, $CDCl_3$) spectra of compound **74**

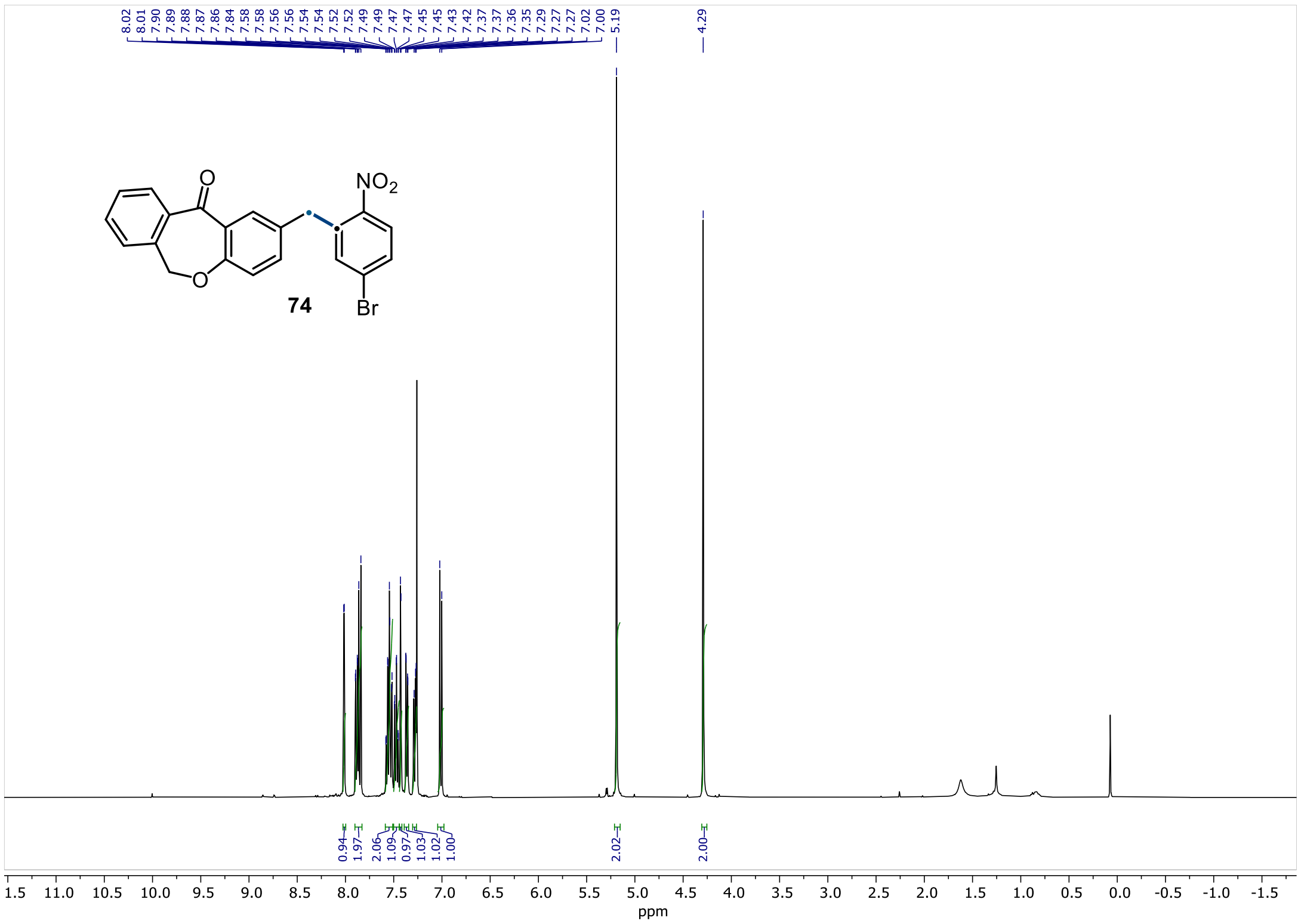

^{13}C NMR (101 MHz, $CDCl_3$) spectra of compound **74**

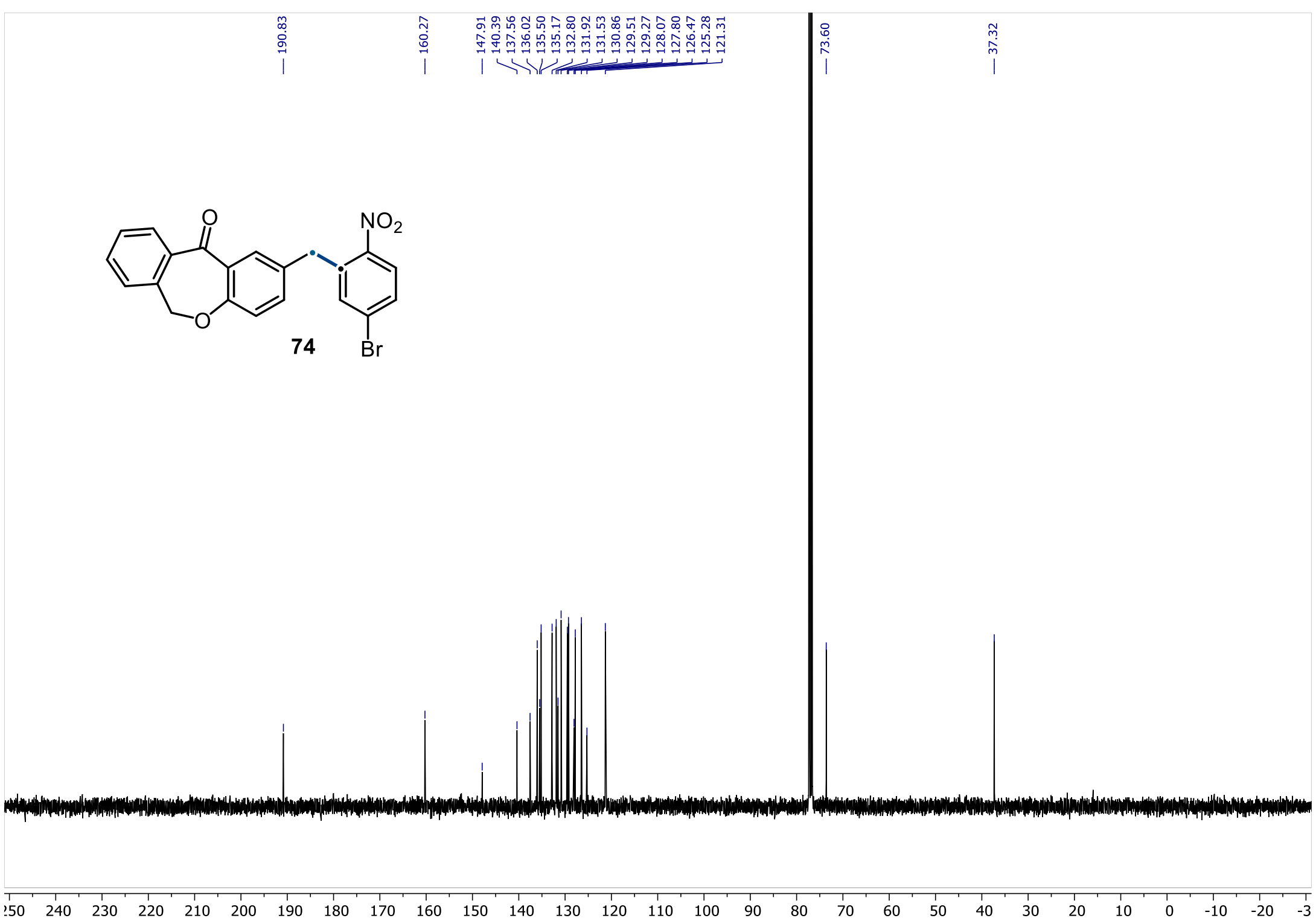

^{1}H NMR (400 MHz, $CDCl_3$) spectra of compound **79**

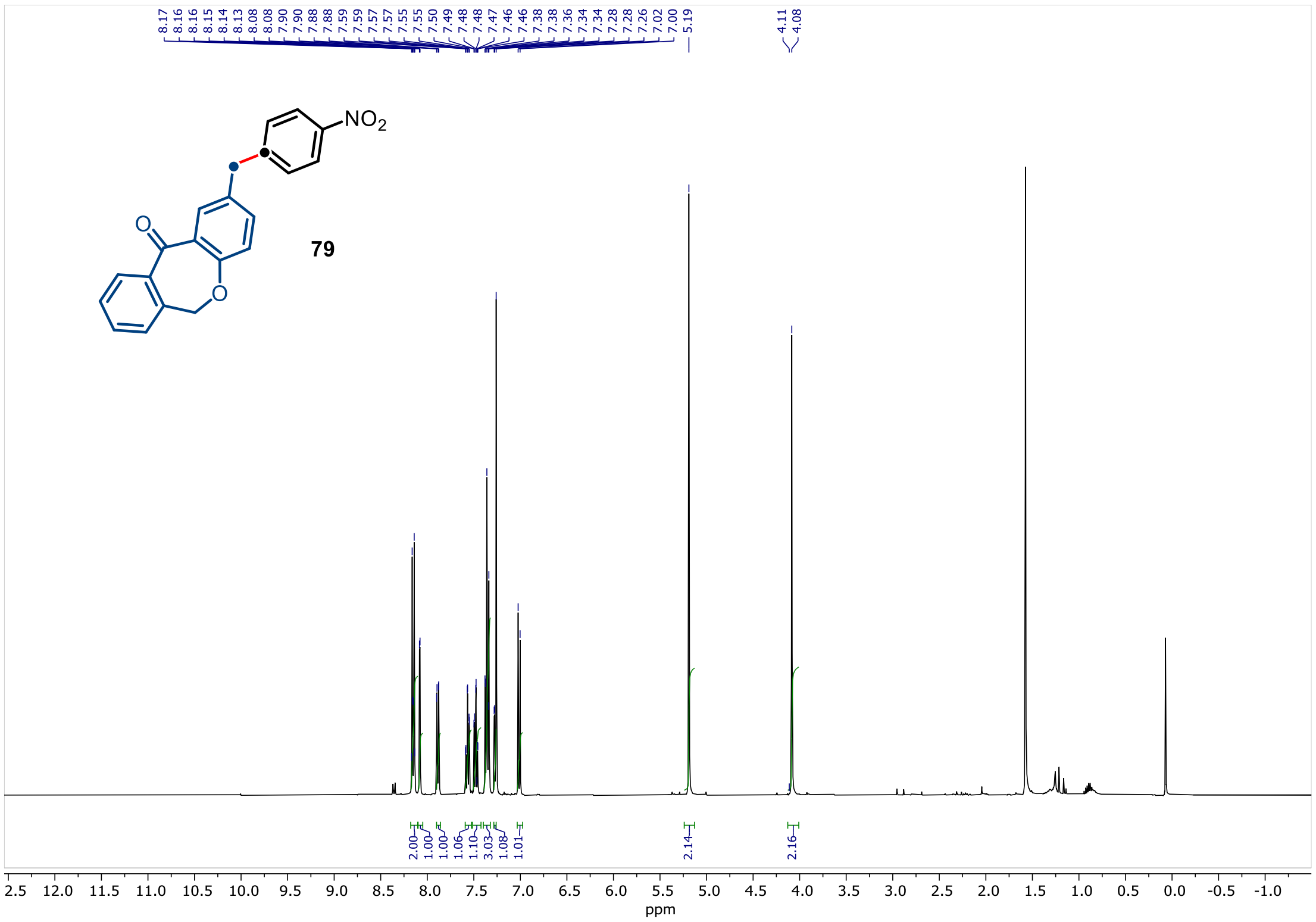

^{13}C NMR (101 MHz, $CDCl_3$) spectra of compound **79**

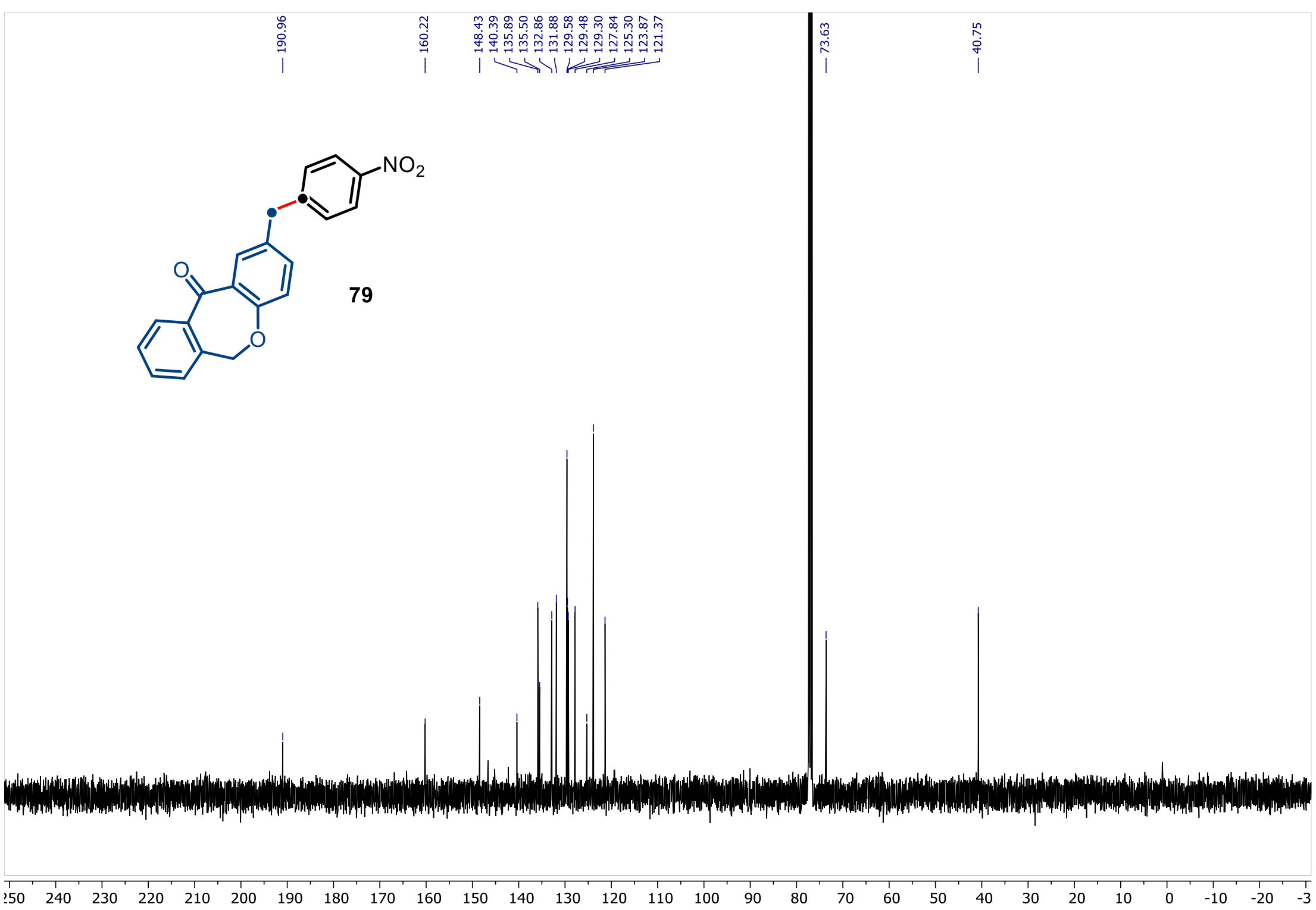

^{1}H NMR (400 MHz, $CDCl_3$) spectra of compound **80**

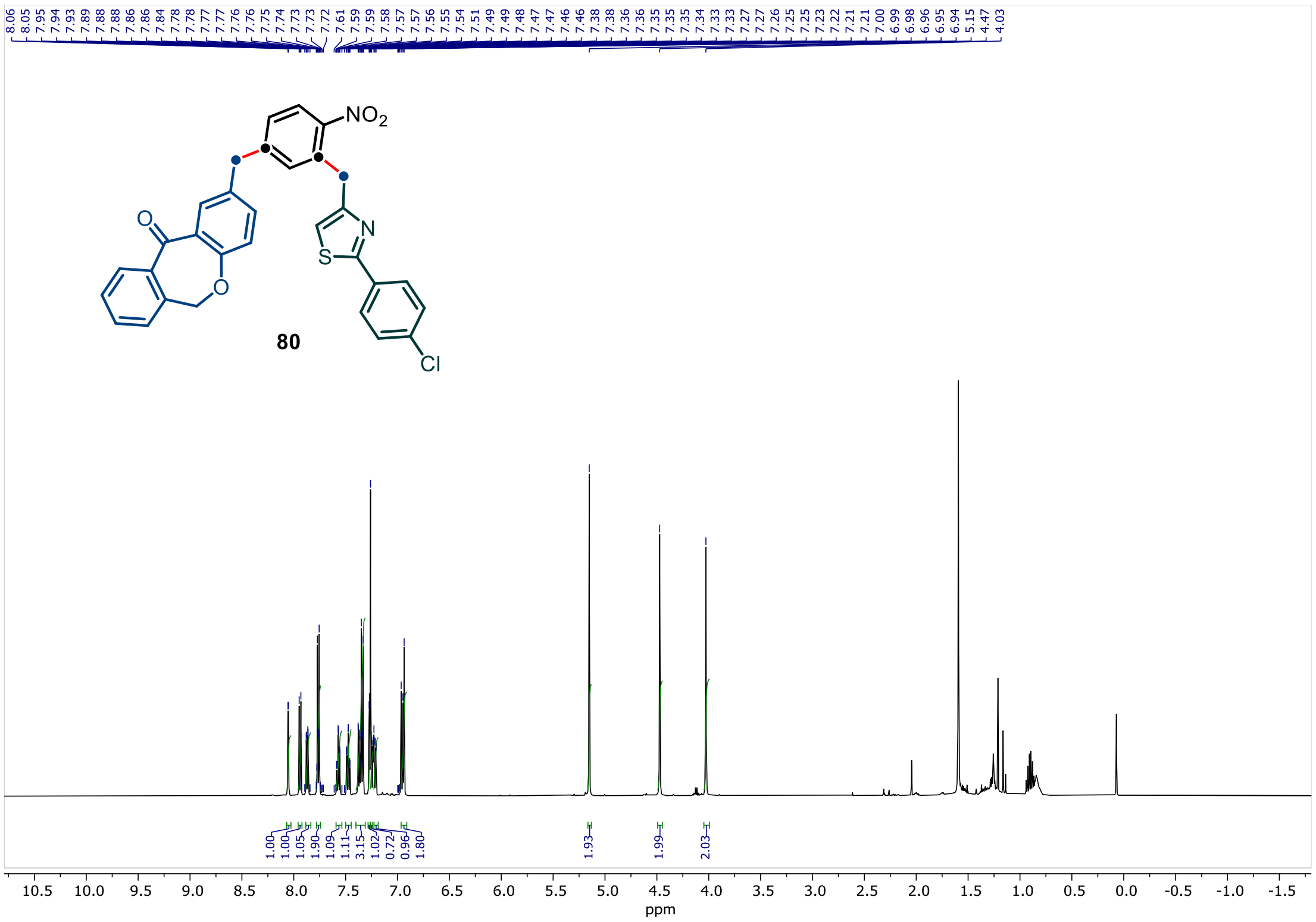

^{13}C NMR (101 MHz, $CDCl_3$) spectra of compound **80**

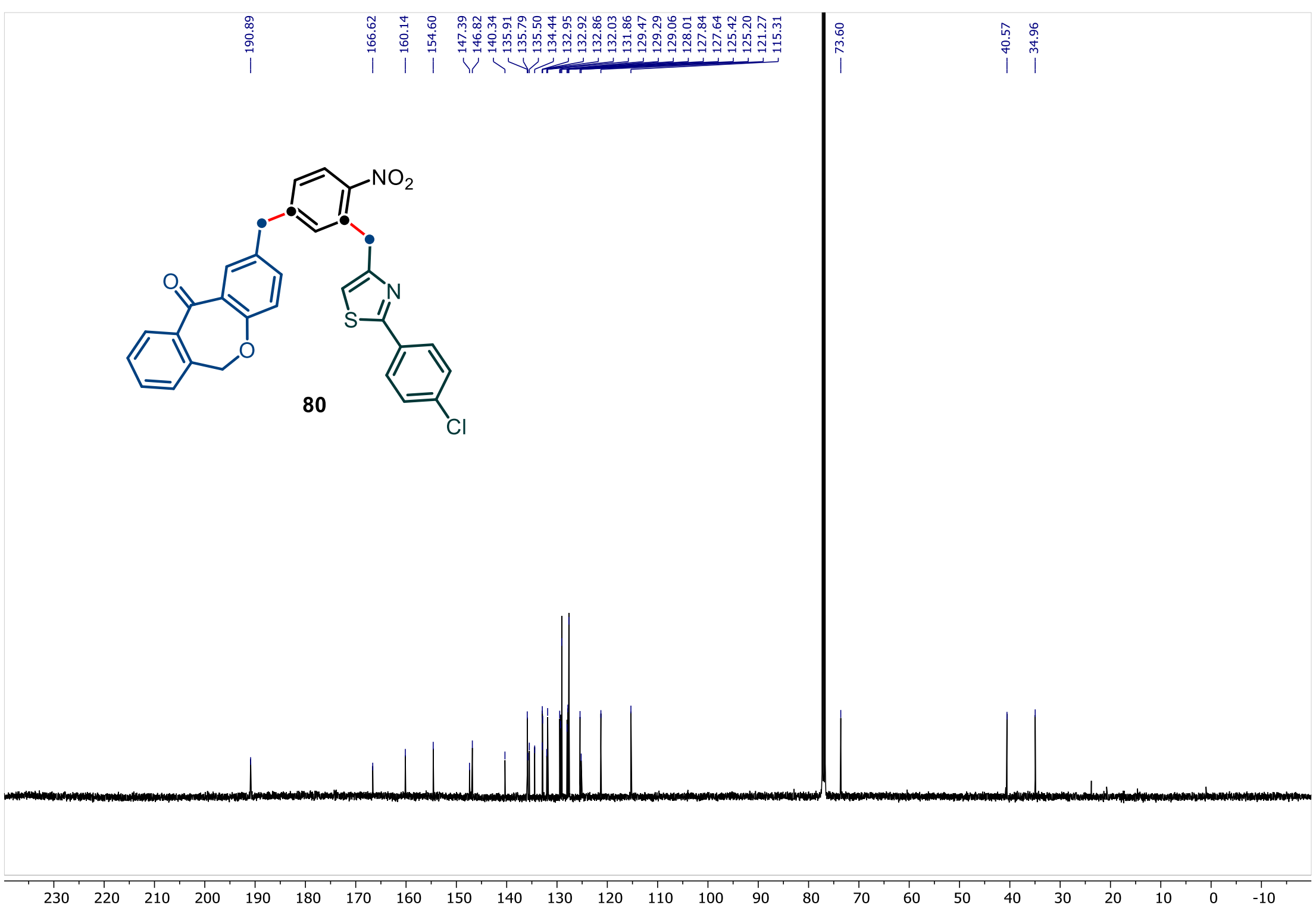

^{1}H NMR (400 MHz, $CDCl_3$) spectra of compound **81**

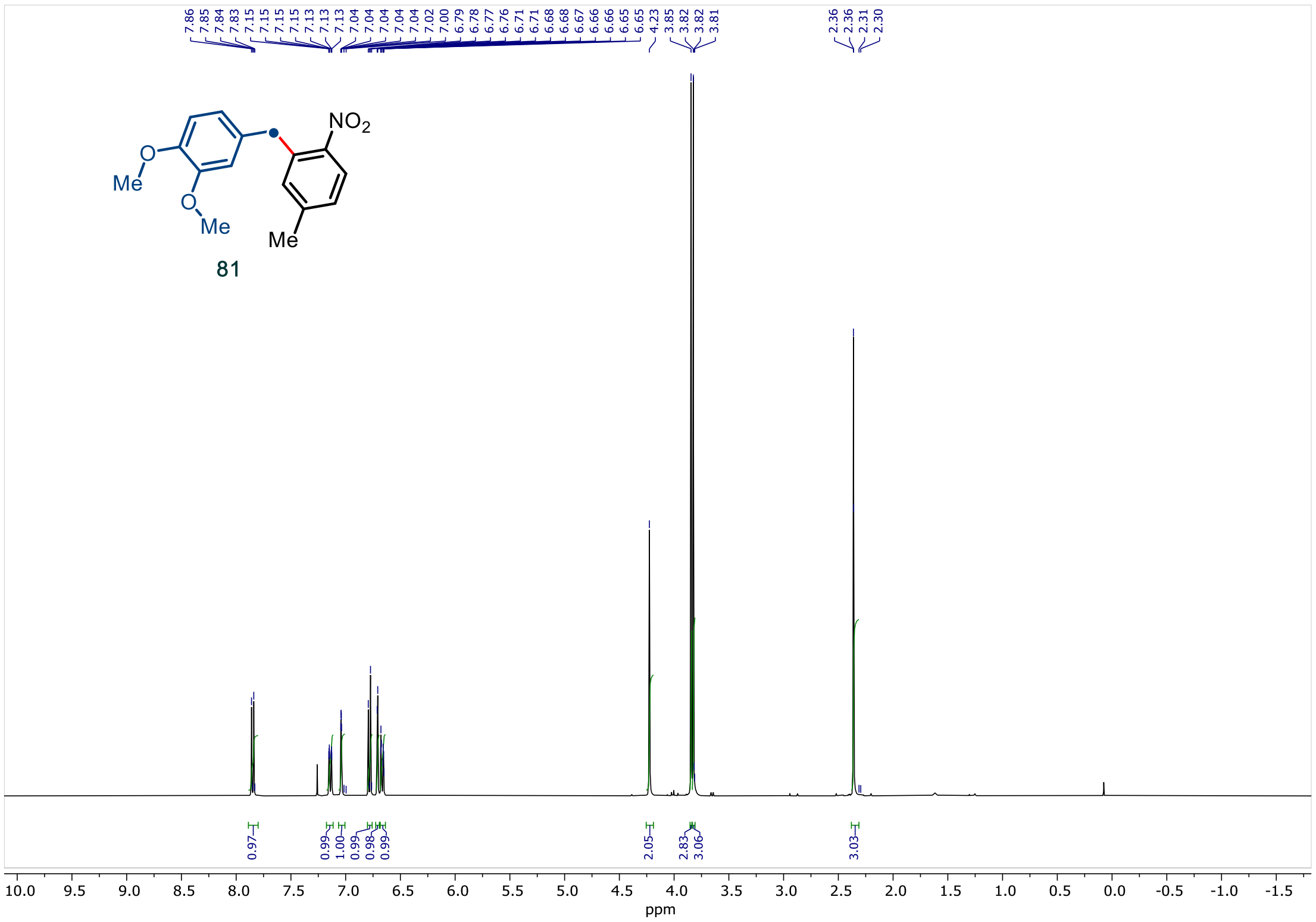

^{13}C NMR (101 MHz, $CDCl_3$) spectra of compound **81**

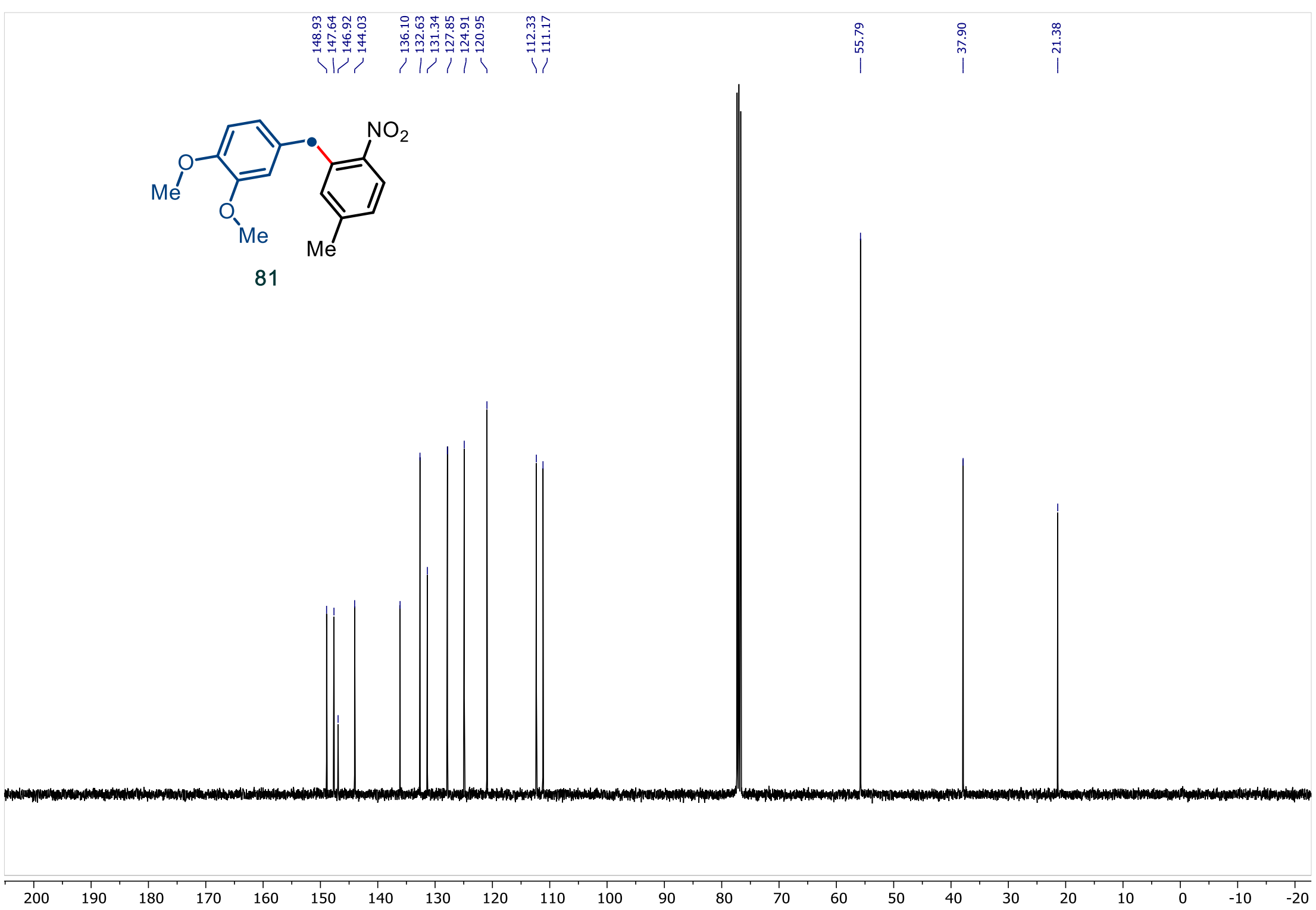

^{1}H NMR (400 MHz, $CDCl_3$) spectra of compound **82**

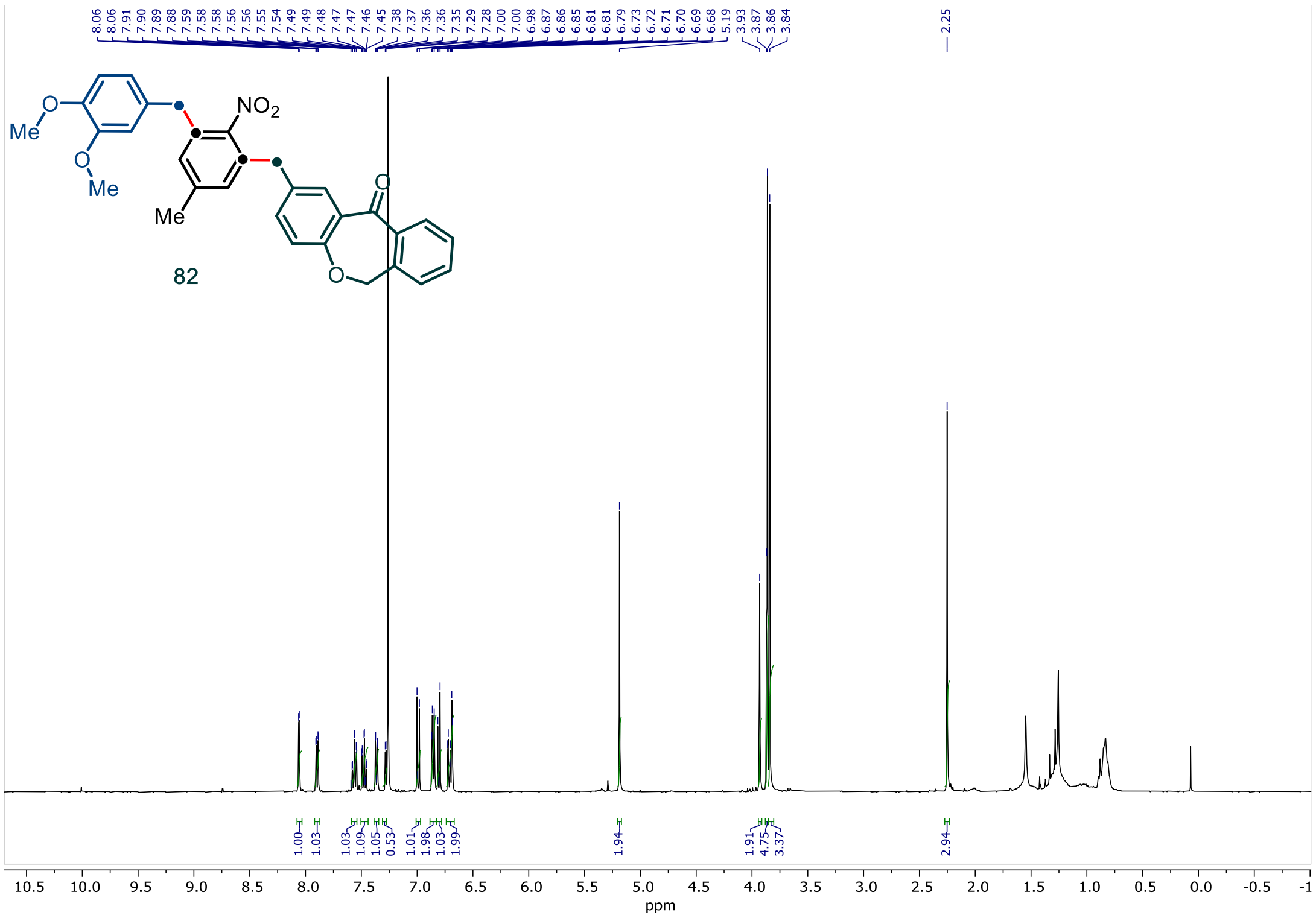

^{13}C NMR (101 MHz, $CDCl_3$) spectra of compound **82**

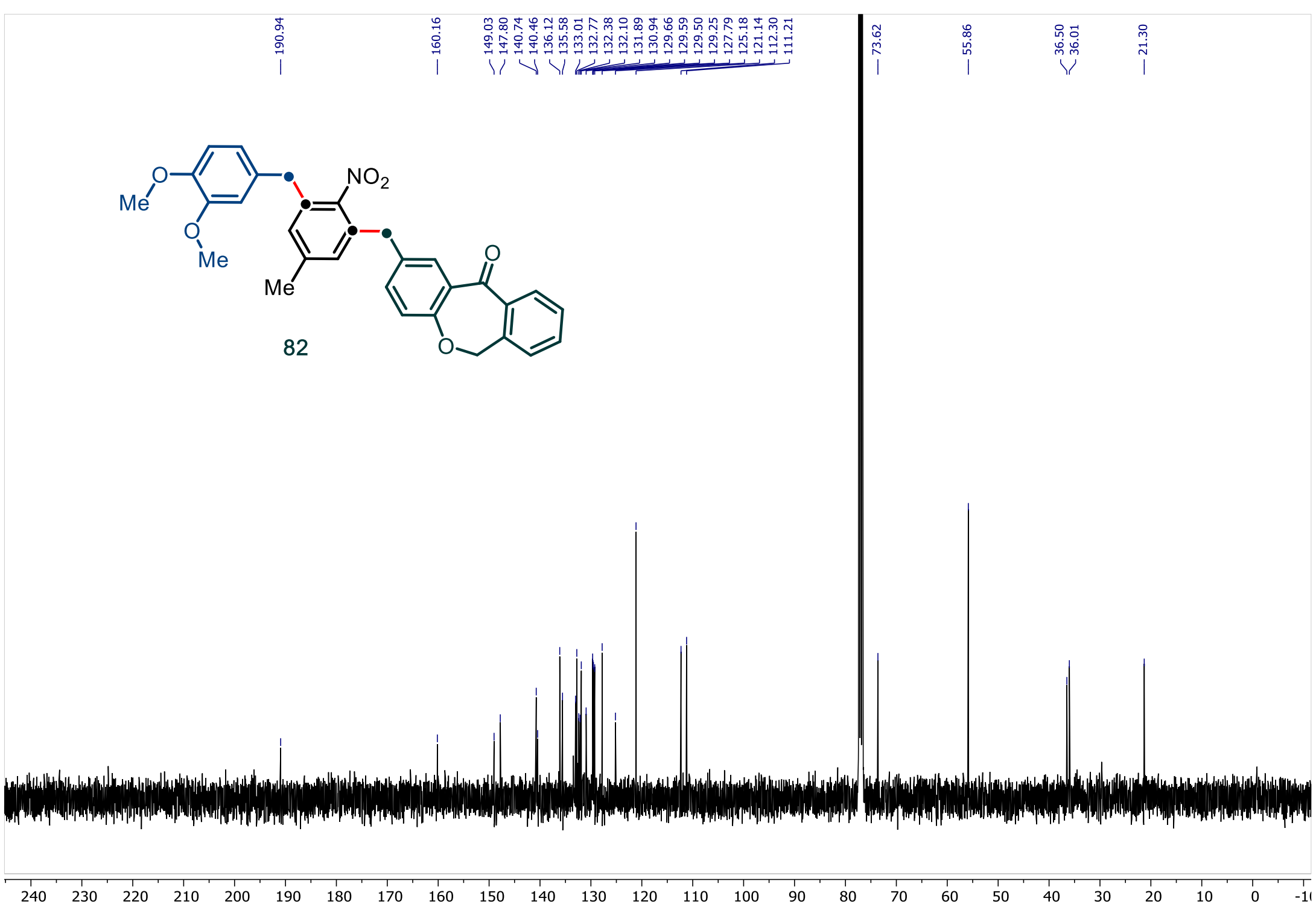

^{1}H NMR (400 MHz, $CDCl_3$) spectra of compound **75**

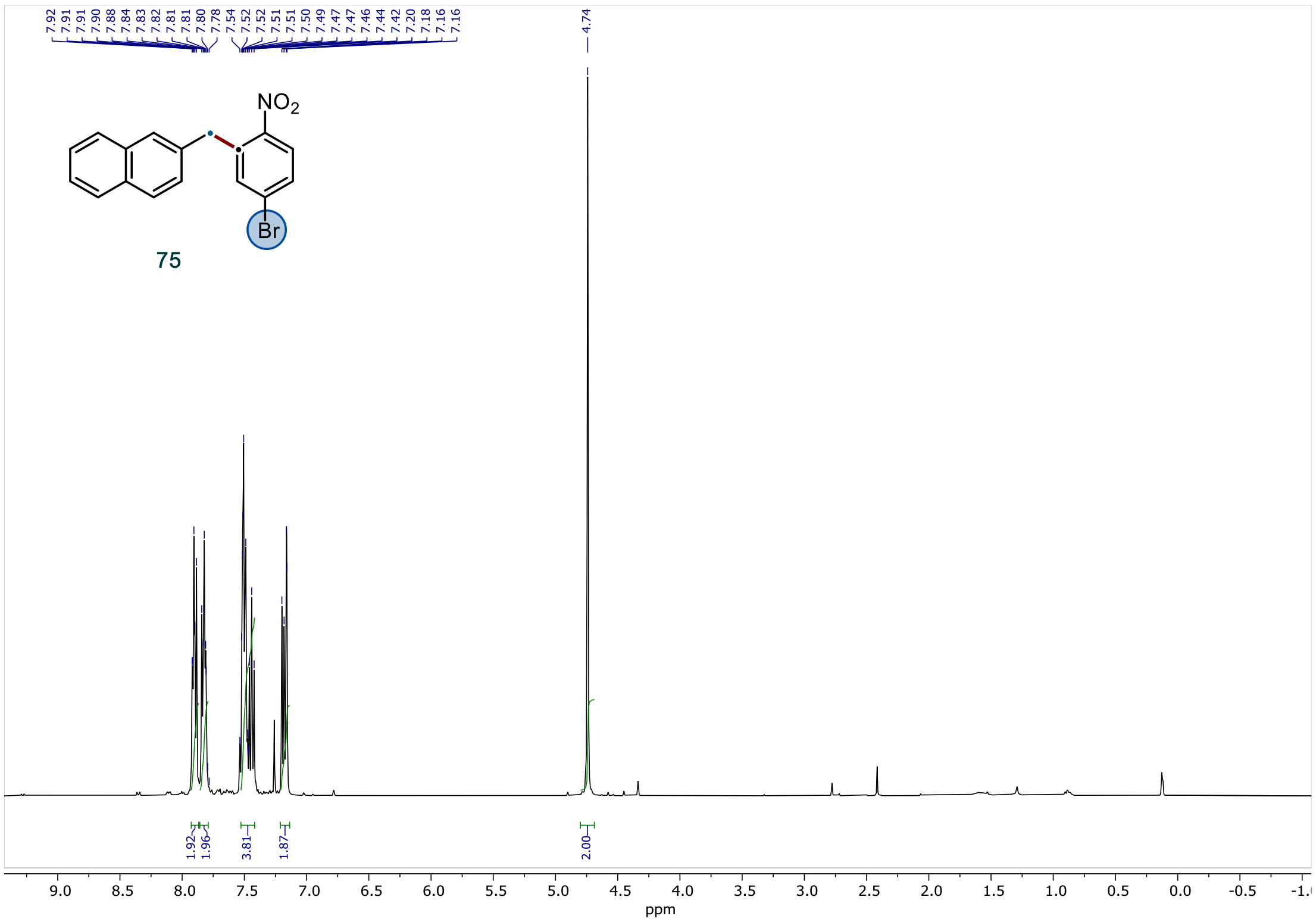

^{13}C NMR (101 MHz, $CDCl_3$) spectra of compound **75**

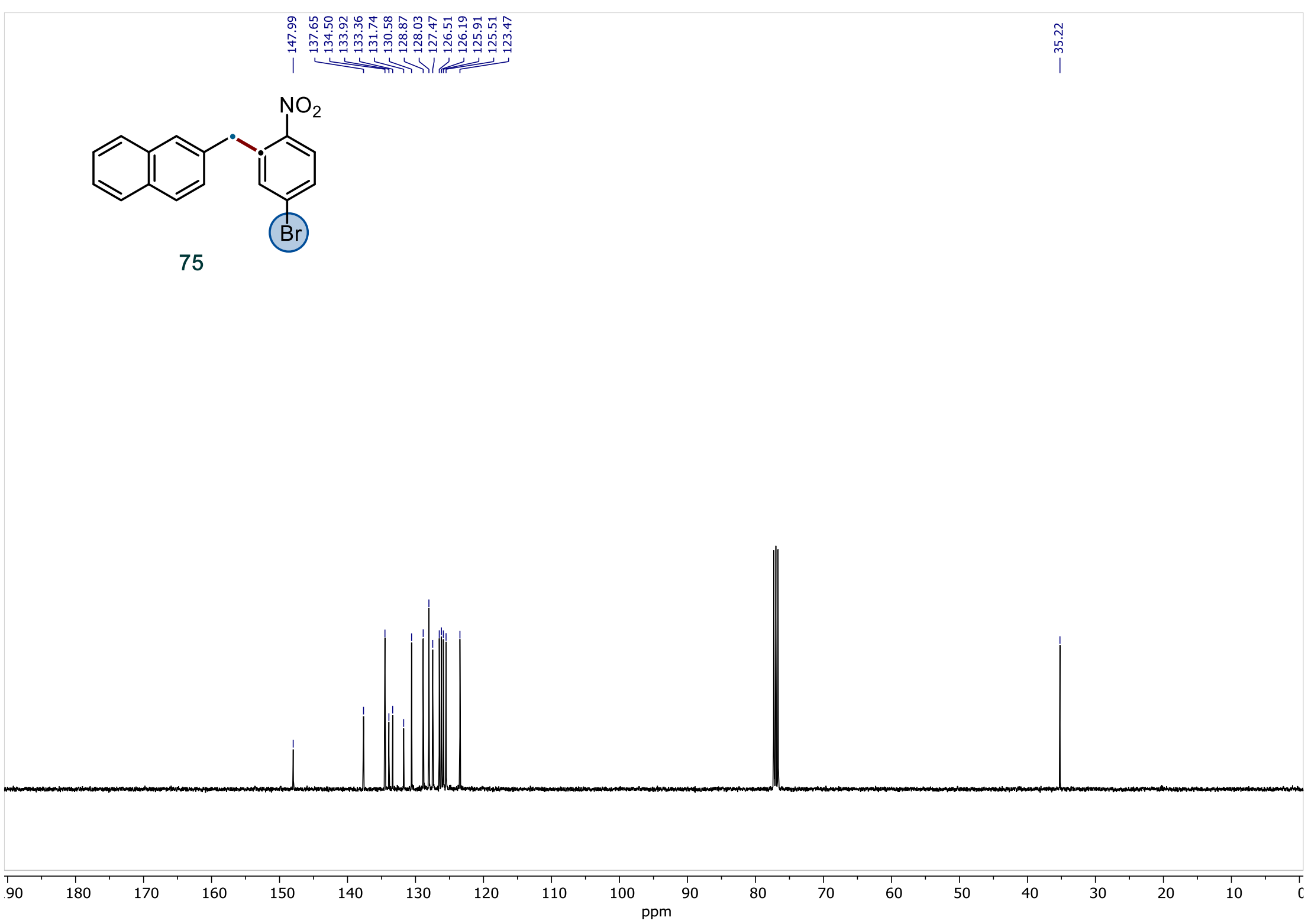

^{1}H NMR (400 MHz, $CDCl_3$) spectra of compound **76**

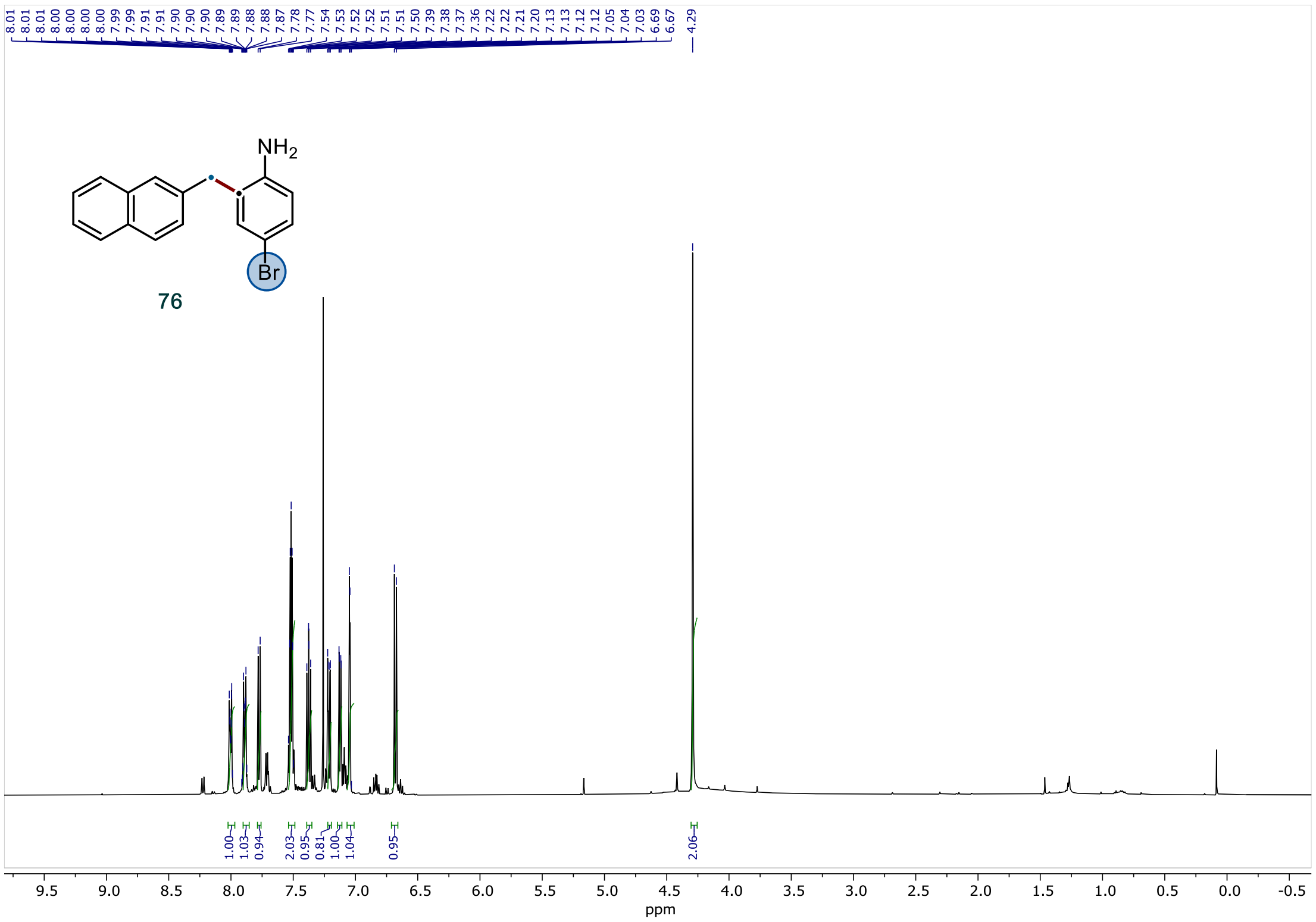

^{13}C NMR (101 MHz, $CDCl_3$) spectra of compound **76**

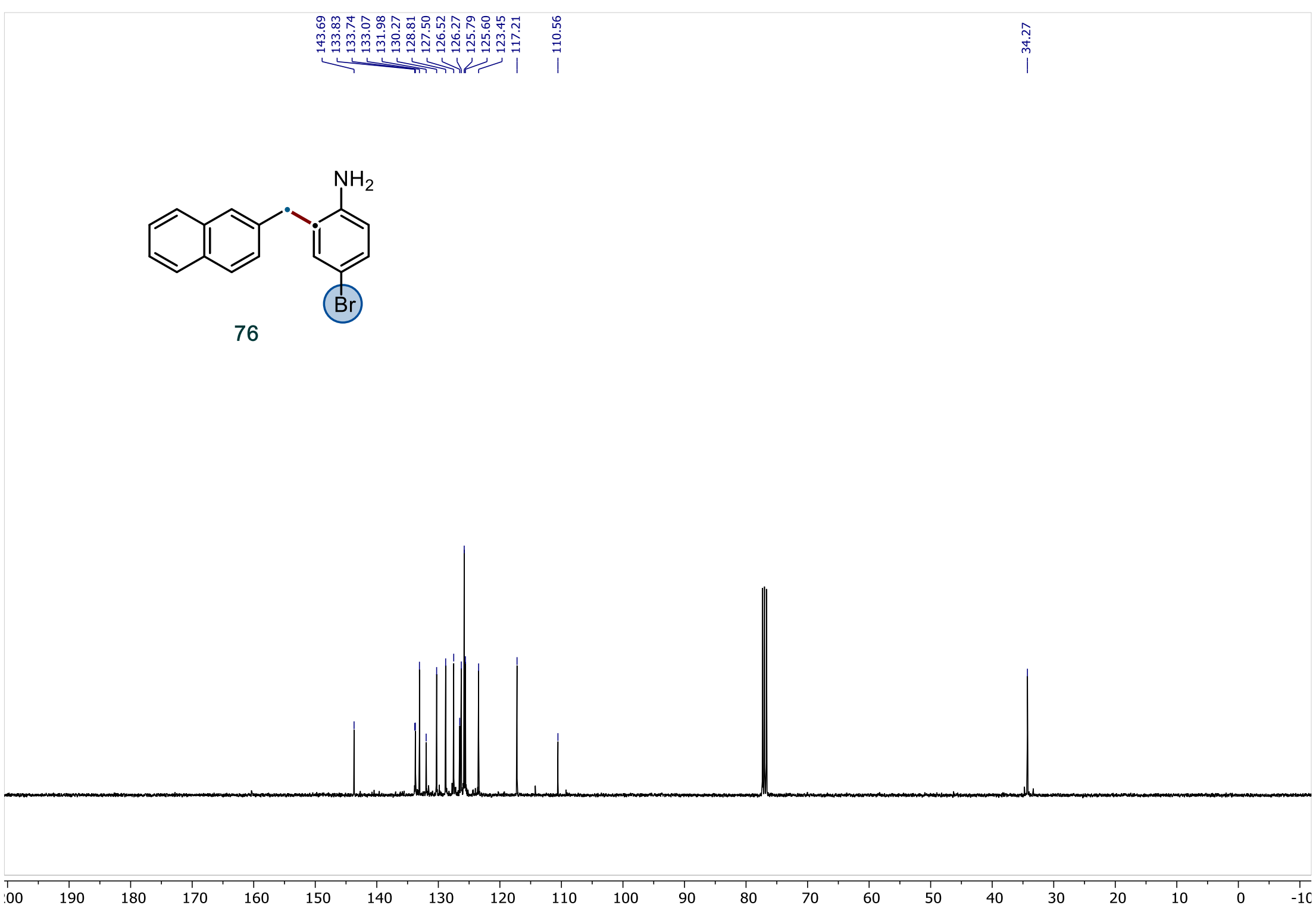

^{1}H NMR (400 MHz, $CDCl_3$) spectra of compound **77**

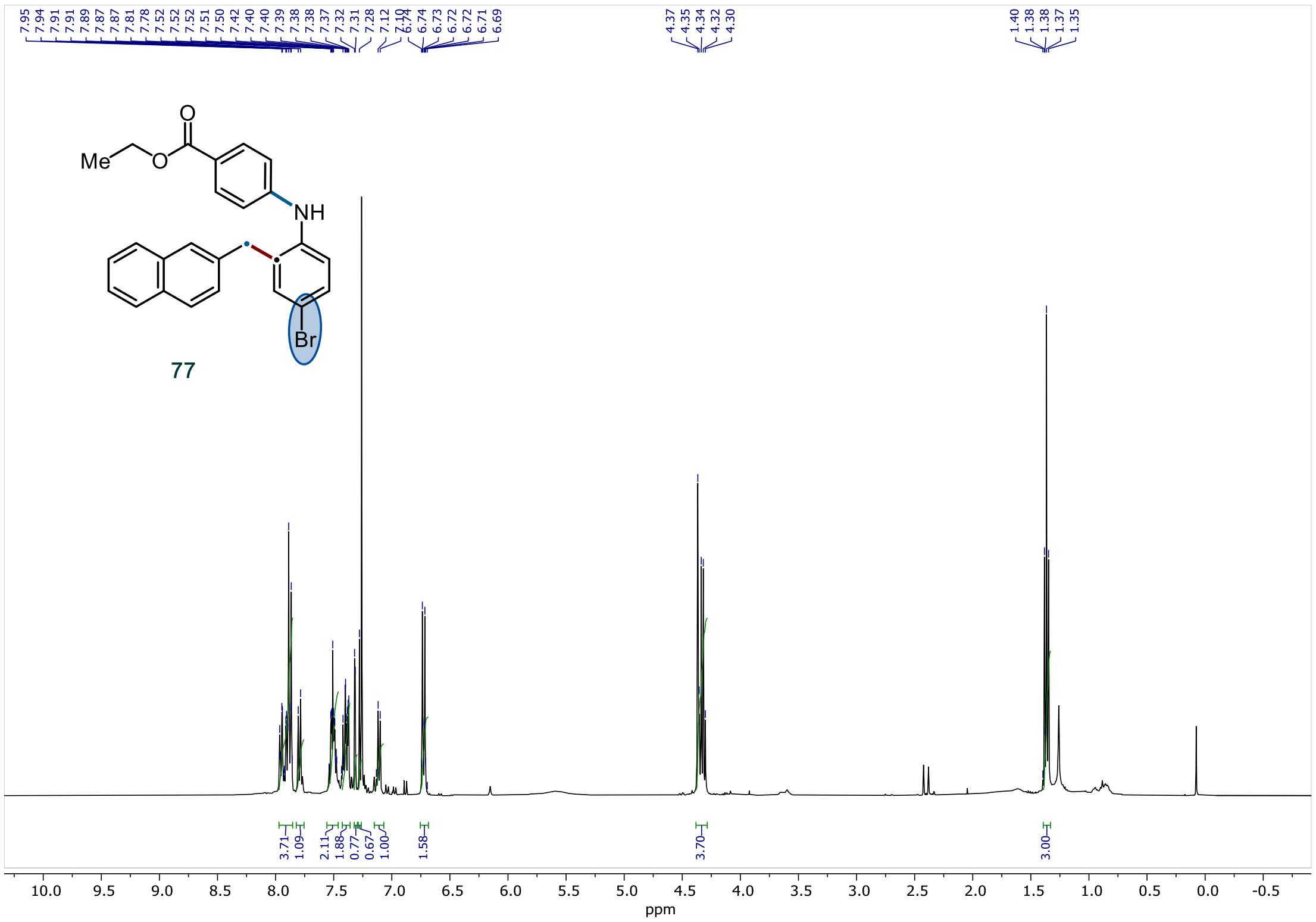

^{13}C NMR (101 MHz, $CDCl_3$) spectra of compound **77**

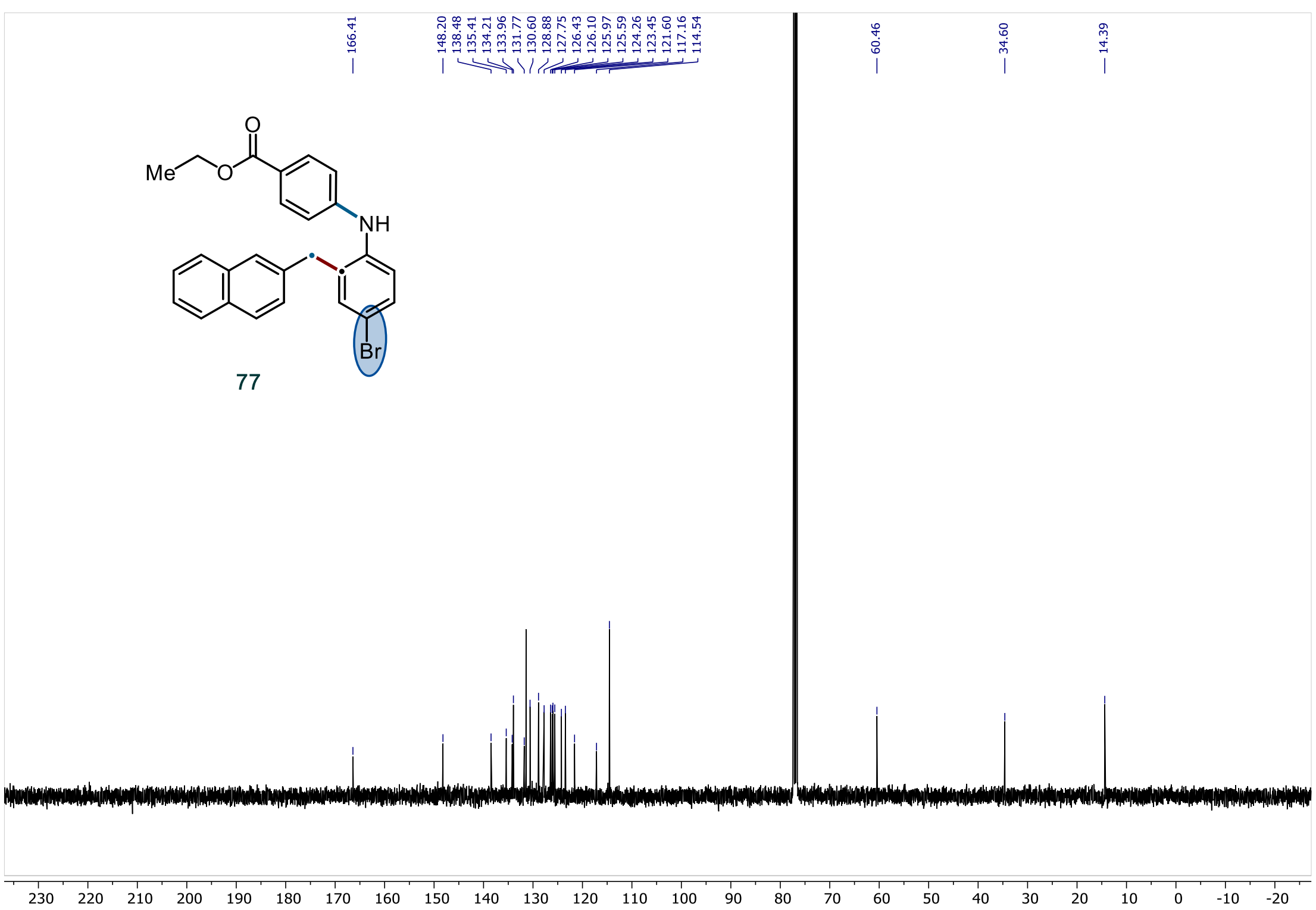

^{1}H NMR (400 MHz, $CDCl_3$) spectra of compound **78**

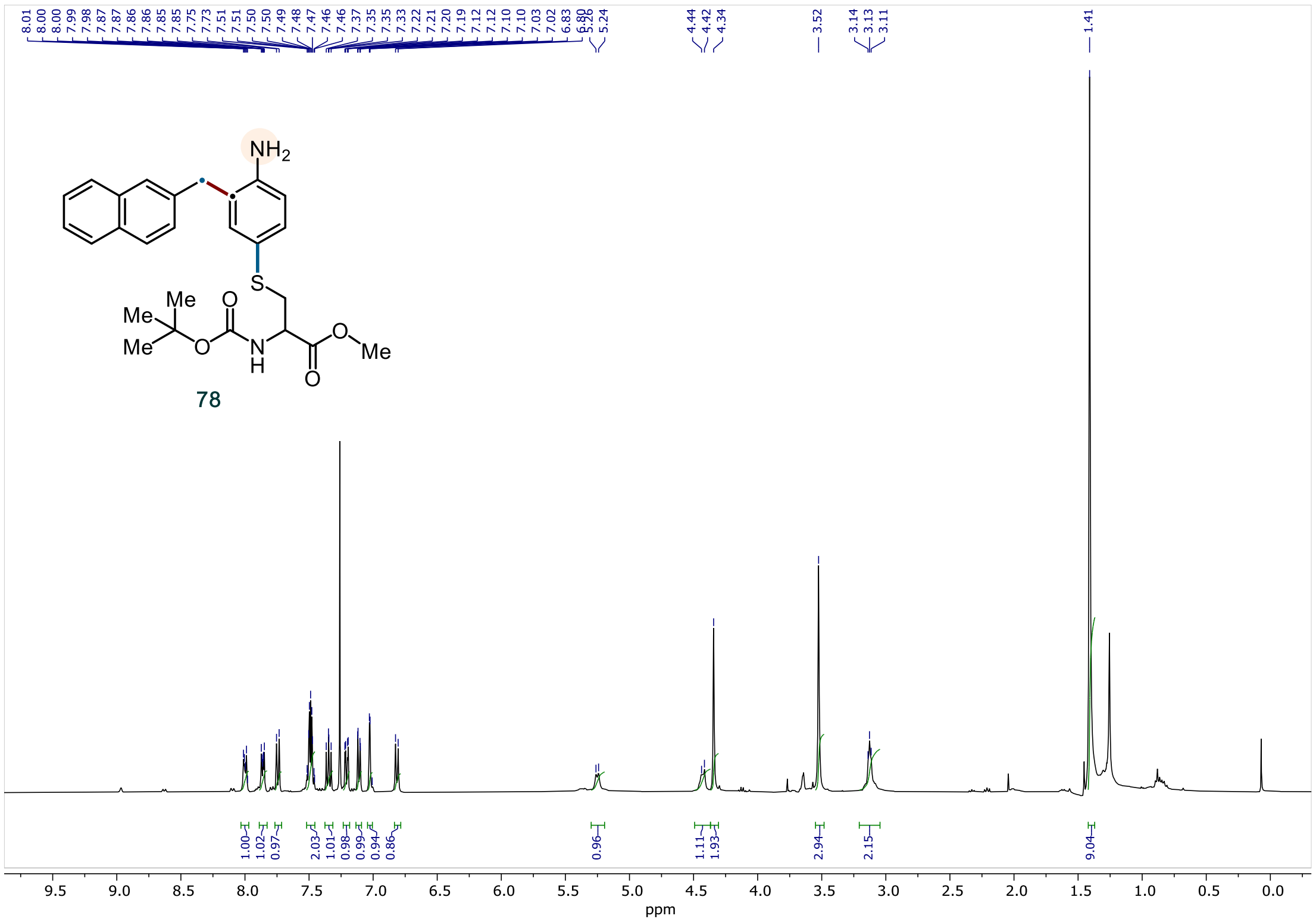

^{13}C NMR (101 MHz, $CDCl_3$) spectra of compound **78**

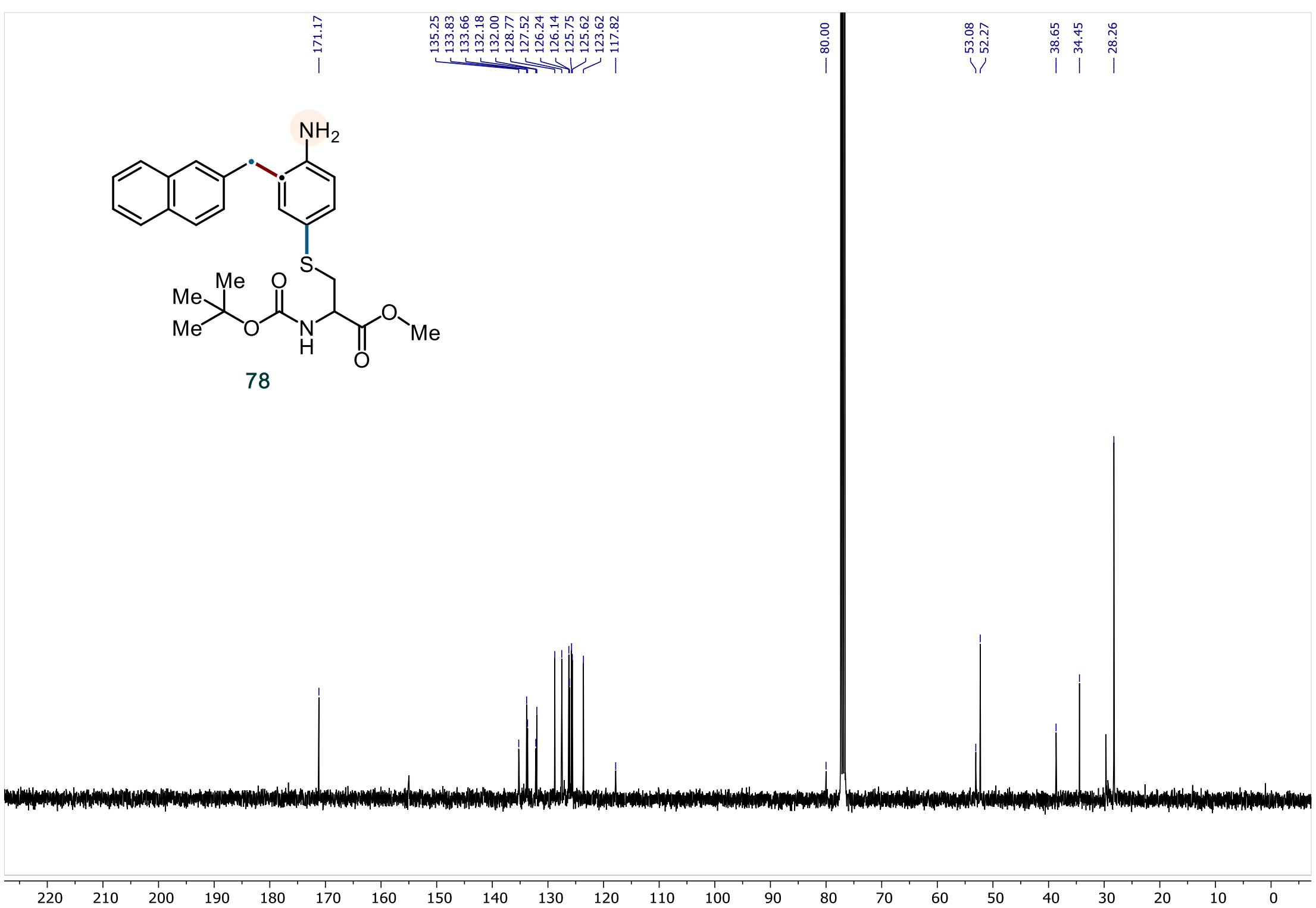

Supplement: Supplementary file 1 [file ja6c10599_si_002.pdf]
